# Evaluating the Viability of Successive Ring‐Expansions Based on Amino Acid and Hydroxyacid Side‐Chain Insertion

**DOI:** 10.1002/chem.202002164

**Published:** 2020-09-11

**Authors:** Aggie Lawer, Ryan G. Epton, Thomas C. Stephens, Kleopas Y. Palate, Mahendar Lodi, Emilie Marotte, Katie J. Lamb, Jade K. Sangha, Jason M. Lynam, William P. Unsworth

**Affiliations:** ^1^ Department of Chemistry University of York York YO10 5DD UK; ^2^ ENSICAEN 6 Boulevard Maréchal Juin, CS 45053 14050 Caen Cedex 04 France

**Keywords:** density functional theory, macrocycles, rearrangement, ring expansion, ring systems

## Abstract

The outcome of ring‐expansion reactions based on amino/hydroxyacid side‐chain insertion is strongly dependent on ring size. This manuscript, which builds upon our previous work on Successive Ring Expansion (SuRE) methods, details efforts to better define the scope and limitations of these reactions on lactam and β‐ketoester ring systems with respect to ring size and additional functionality. The synthetic results provide clear guidelines as to which substrate classes are more likely to be successful and are supported by computational results, using a density functional theory (DFT) approach. Calculating the relative Gibbs free energies of the three isomeric species that are formed reversibly during ring expansion enables the viability of new synthetic reactions to be correctly predicted in most cases. The new synthetic and computational results are expected to support the design of new lactam‐ and β‐ketoester‐based ring‐expansion reactions.

## Graphical abstract

**Does the ring fit?** Ring expansions based on amino/hydroxyacid side‐chain insertion are strongly dependent on ring size. Herein, guidelines are provided on which ring systems are more likely to be successful. Synthetic results are supported by computational chemistry. Calculating the relative Gibbs free energies of isomeric species formed during expansion helps predict the viability of new reactions.

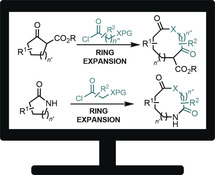

## Introduction

Rearrangements that allow ring‐enlarged products to be prepared from smaller cyclic systems have much utility in synthetic chemistry.^1^, ^2^ Ring expansions are particularly useful for the synthesis of medium‐sized rings (8‐ to 11‐membered) and macrocycles (12+ membered), as alternatives to direct end‐to‐end cyclisations.^3^ End‐to‐end cyclisations can be difficult and unpredictable processes due to competing intermolecular coupling and other side reactions, and they often necessitate the use of impractical high‐dilution (or pseudo‐high‐dilution) conditions.^4^ In contrast, high dilution can often be avoided completely in well‐designed ring‐expansion systems.^1^, ^2^, ^5^

**Scheme 1:**
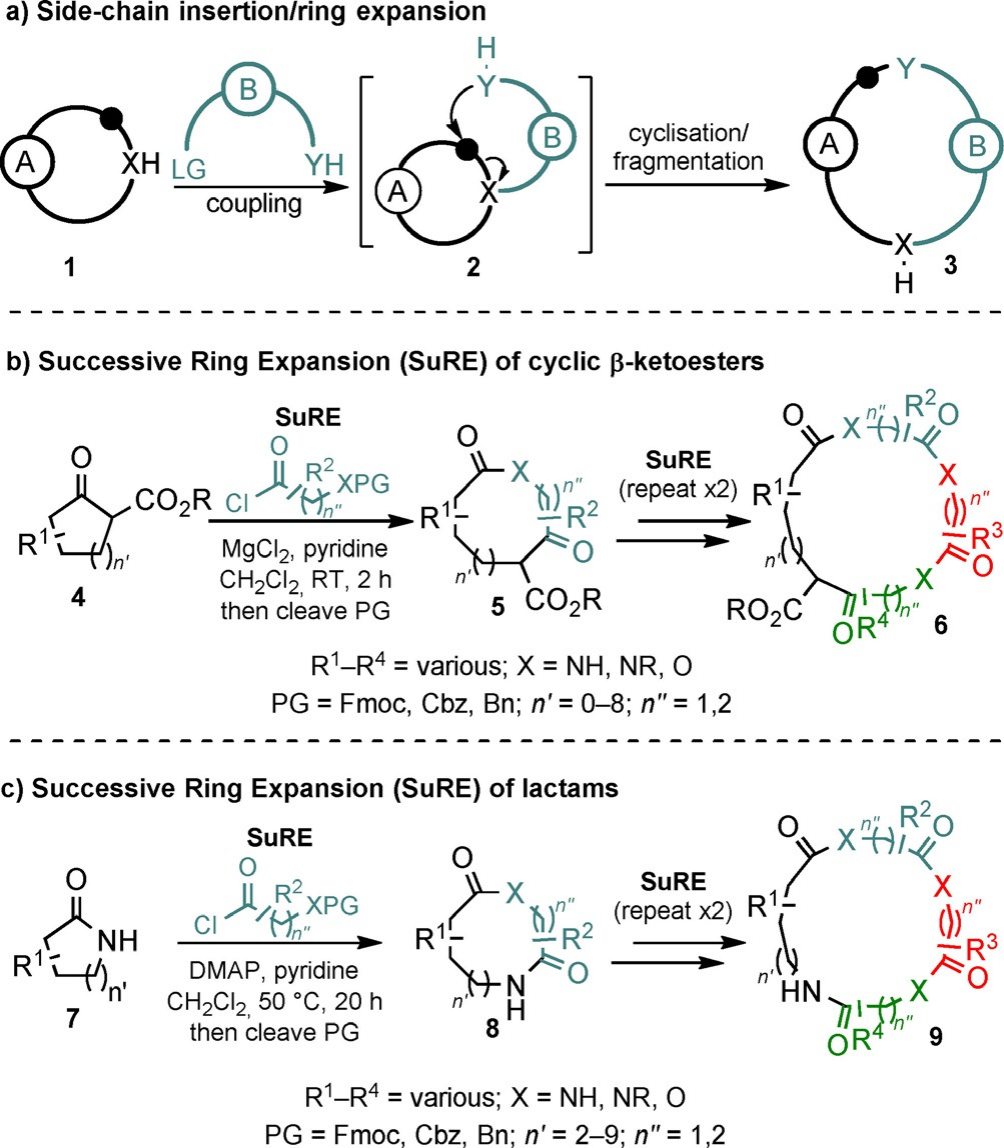
Side‐chain insertion ring‐expansion reactions and Successive Ring Expansion (SuRE).

Side‐chain insertion ring‐expansion reactions (Scheme 1 a) are a useful sub‐class of ring expansion, as the requisite precursors are generally straightforward to prepare. Various methods in which the ring expansion is accompanied by concomitant C−O, C−N and C−C bond formation are known, and this topic has been recently reviewed.1a Amongst this class of reaction, our group has developed a series side‐chain insertion ring expansion processes that can be performed iteratively. These methods, which we have termed “Successive Ring Expansion” (SuRE) reactions,^5^ enable the controlled, iterative insertion of amino acid or hydroxyacid‐derived linear sequences into cyclic β‐ketoesters (**4→6**, Scheme 1 b)5a, 5b or lactams (**7→9**, Scheme 1 c).5c, 5d

**Scheme 2:**
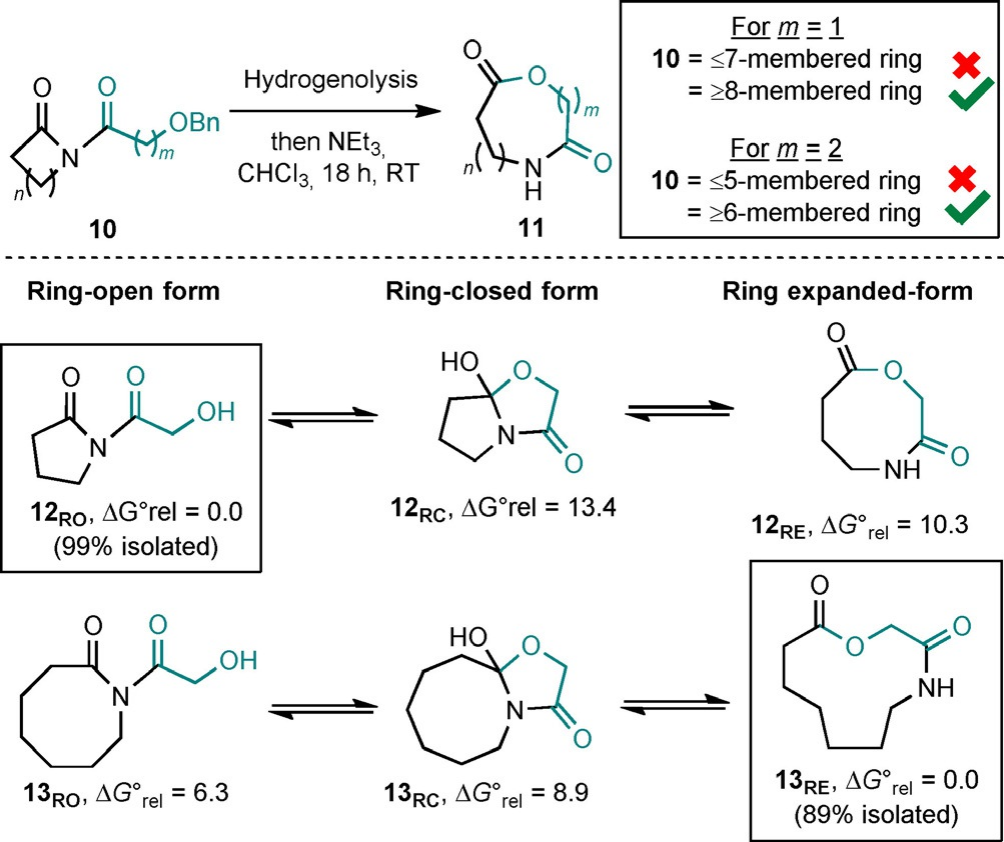
Ring‐size dependency on the outcome of the ring expansion of imides into aza‐lactones. ΔG∘rel values are given in kcal mol^−1^.

In our experience, the most important factor in determining the outcome of new ring‐expansion reactions of the types summarised in Scheme 1 b and c is ring size. This is well demonstrated by the outcomes of our published lactone‐forming ring expansions of imides of the form **10** (Scheme 2).5d Thus, for both α‐ and β‐hydroxyacid derived linear fragments (3‐ and 4‐atom ring expansions, respectively), there is a clear point at which ring expansion “switches on”; the reactions work for starting materials with rings that are eight‐membered or more for three‐atom expansions (*m*=1) and rings that are six‐membered or more for four‐atom expansions (*m*=2). The analogous reactions fail for smaller ring variants. We have previously postulated that these reactions are under thermodynamic control, and hence that the reaction outcomes depend on the relative Gibbs free energies of the three isomeric forms that the substrate must pass through for ring expansion to occur. This idea is supported by calculations performed at the DFT/B3LYP/6‐31G* level of theory;5d, 6, 7, 8 thus, five‐membered ring‐open form imide **12_RO_** (RO=ring‐opened) was calculated to be significantly lower in Gibbs free energy than its isomeric ring‐closed (**12_RC_**, RC=ring‐closed) and ring‐expanded forms (**12_RE_**, RE=ring‐expanded), and this was replicated in the synthetic results, with imide **12_RO_** being isolated in 99 % yield following hydrogenolysis of the parent benzyl protected imide (**10**, where *n*=2, *m*=1). Conversely, in the case of the analogous eight‐membered starting material (**10**, where *n*=5, *m*=1), the ring‐expanded form **13_RE_** was calculated to be the most stable isomer, and upon testing the reaction, **13_RE_** was isolated in 89 % yield, meaning that the calculations again were in line with the synthetic results.

These calculations, which drew inspiration from a similar approach used by Yudin and co‐workers,2d were done primarily to validate our ideas about the reactions being under thermodynamic control. In this work, we have explored the validity of using calculations of this type predictively. As we continue to develop this research programme, having a reliable predictive tool to inform the likelihood of new SuRE variants working before committing to labour‐intensive synthetic efforts will be of value. The utility of this approach is demonstrated herein; in total, 52 new ring‐expansion reactions have been attempted, with 48 successfully furnishing the desired ring‐expanded product. Our DFT/B3LYP/6‐31G* method correctly predicted the reaction outcome in almost all cases, and compared favourably when benchmarked against other alternative methods, including those that model solvation and dispersion interactions. Thus, we believe that this widely available DFT/B3LYP/6‐31G* approach will be useful to help assess the viability of new ring‐expansion reactions before committing to synthetic efforts.

## Results and Discussion

**Scheme 3:**
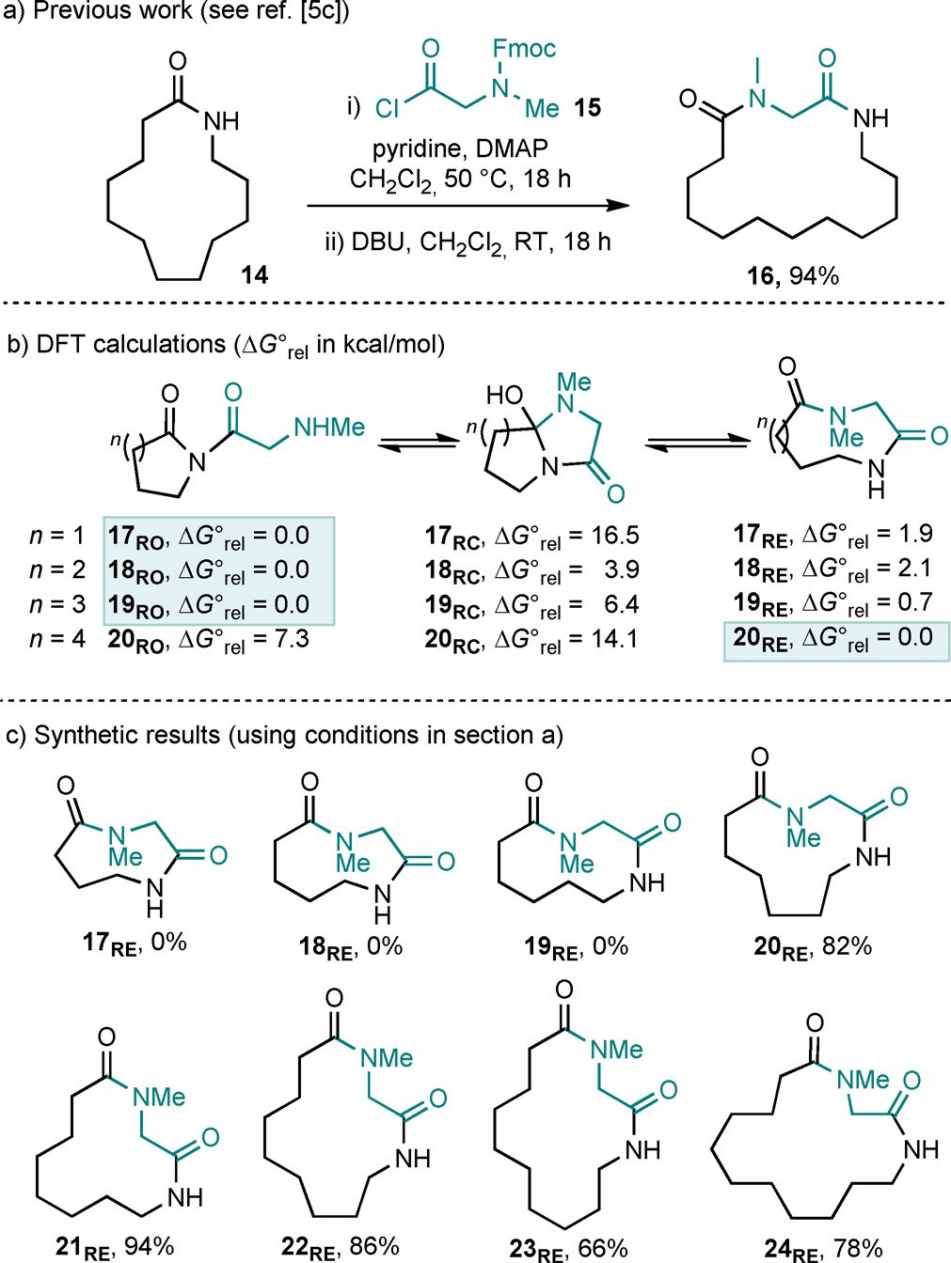
Ring‐size dependency on the outcome of the ring expansion of imides with N‐methyl sarcosine derivatives. ΔG∘rel values are given in kcal mol^−1^ with thermal corrections at 298 K.

We started by examining the ring expansion of simple lactams with sarcosine derivative **15**. We had already shown that this acid chloride is compatible with our standard lactam ring expansion method (**14**→**16**, Scheme 3 a), but prior to this work, 13‐membered lactam **14** was the smallest aliphatic lactam on which we have reported a successful ring expansion with any linear α‐amino acid chloride.

Prior to doing the synthetic chemistry, we ran DFT calculations based on the method used in our earlier study. To summarise this method, each of the three components of the equilibria deriving from five‐ to eight‐membered ring imide precursors **17_RO_**‐**20_RO_** were optimised at the DFT/B3LYP/6‐31G* level of theory in vacuum.^6^, ^7^, ^8^ Conformational searches of the optimised structures were performed at the Molecular Mechanics Force Field level. All the generated structures were retained, and their energies were calculated using DFT/B3LYP/6‐31G*. The lowest energy geometry in each case was selected, fully optimised and determined to be minima by the absence of negative vibrational modes, in vacuum using DFT/B3LYP/6‐31G*. In each case, the relative free energies of the imide (**17_RO_**–**20_RO_**), ring‐closed (**17_RC_**–**20_RC_**), and ring‐expanded (**17_RE_**–**20_RE_**) isomers were calculated, with ΔG∘rel values quoted in kcal mol^−1^ (Scheme 3 b). More information about the choice of this method and method effects are included later in the manuscript;^7^ until then, the discussion will focus on the synthetic aspects and DFT/B3LYP/6‐31G* calculations.

In the five‐ to seven‐membered series, the imide isomers **17_RO_**–**19_RO_** were calculated to be the most stable, thus suggesting that ring expansion is unlikely to proceed in these examples. This prediction was verified by synthetic results; thus, none of the ring‐expanded products **17_RO_**–**19_RO_** were obtained when attempts were made to prepare them using the standard conditions, with no tractable products isolated from these reactions (**17_RO_**–**19_RO_**_,_ Scheme 3 c). Conversely, the ring‐expanded isomer **20_RE_** was calculated to be the lowest in free energy in the eight‐membered ring series, and this again was borne out in the synthetic results, with **20_RE_** isolated in 82 % yield. Thus, the use of an eight‐membered ring starting material (or larger) appears to be the ‘switch on’ point for this series, as it was for the analogous lactone systems in Scheme 2. This is supported by the high yielding (66–94 %) ring expansions of 9–12‐membered lactam systems to form products **21_RE_**–**24_RE_** under the standard conditions.

**Scheme 4:**
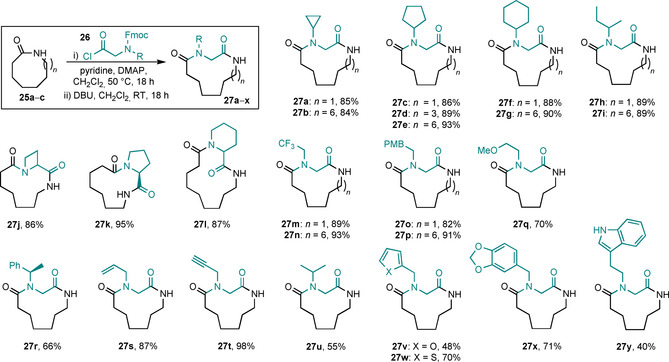
Scope of lactam ring‐expansion reactions with N‐functionalised amino acids.

Medicinal interest in medium‐sized rings and macrocycles has increased significantly in the last decade,^9^ and the reaction variant described in Scheme 3 appears to be well suited for use in the preparation of peptoid‐containing macrocycles,^10^ as long as the starting lactam is an eight‐membered ring or larger. Thus, to better demonstrate its potential utility, we went on to investigate the range of N‐substituents that can be tolerated on the linear unit **26**, with these results summarised in Scheme 4. In total, 24 new ring‐expansion reactions of this type have been performed, to make **27 a**–**y** (**27 k** was described previously)5c using various functionalised amino acid‐derived linear fragments (**26**). Most of the reactions proceeded in high yield (the yield quoted is for the full N‐acylation/protecting group cleavage/rearrangement sequence) under the standard reaction conditions, significantly expanding the range and diversity of amino acid derivatives that have been demonstrated in the SuRE method to date.

All the new SuRE reactions presented in Scheme 4 worked (at least to some degree), although there were a few outliers that were lower yielding (e.g., furan‐derivative **27 v**). In these cases, we believe that the lower yield is not caused by an inherent difference in the thermodynamics of the ring expansion equilibrium (i.e., the relative free energies of the analogous isomers **27 v_RO_**, **27 v_RC_** and **27 v_RE_** are in line with those for the methyl analogue **20**, see SI for full details)^11^ but can be explained by substrate‐dependent side reactions or problems with the preceding N‐acylation step. For example, in the case of furan derivative **27 v**, the lower yield is largely due to incomplete N‐acylation (step i), which in turn is likely to be a consequence of the relative instability of the acid‐sensitive furan motif. Unexpected side reactions/degradation also cannot be ruled out during the ring‐expansion reaction (step ii) in cases where more reactive functional groups are involved.

**Scheme 5:**
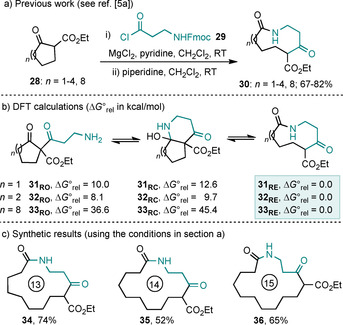
Ring‐size dependency of the outcome of the ring expansion of β‐ketoesters with β‐alanine‐derived acid chloride **29**. ΔG∘rel values are given in kcal mol^−1^.

Next, we examined the ring expansions of cyclic β‐ketoesters. These reactions were the subject of our first two publications in this area,5a, 5b which focused mainly on the insertion of β‐amino acid derived linear fragments; for example, five‐ to eight‐ and 12‐membered cyclic β‐ketoesters (**28**) were all found to undergo smooth ring expansion (to form products of the type **30**) upon reaction under the reported conditions with β‐alanine derived acid chloride **29** (Scheme 5 a).5a DFT/B3LYP/6‐31G* calculations were performed to measure the energies of the equilibrating isomers of the five‐, six‐, and 12‐membered ring systems **31**–**33** as before. Pleasingly, the calculations suggest that the ring‐expanded isomers are lowest in energy by a clear margin, suggesting that there is a strong thermodynamic driving force for ring expansion in this series (Scheme 5 b). To complete the synthetic series, we went on to perform the ring expansion of nine‐ to 11‐membered β‐ketoesters for the first time, with these new synthetic reactions proceeding well, affording lactams **34**–**36** (52–74 %, Scheme 5 c).

**Scheme 6:**
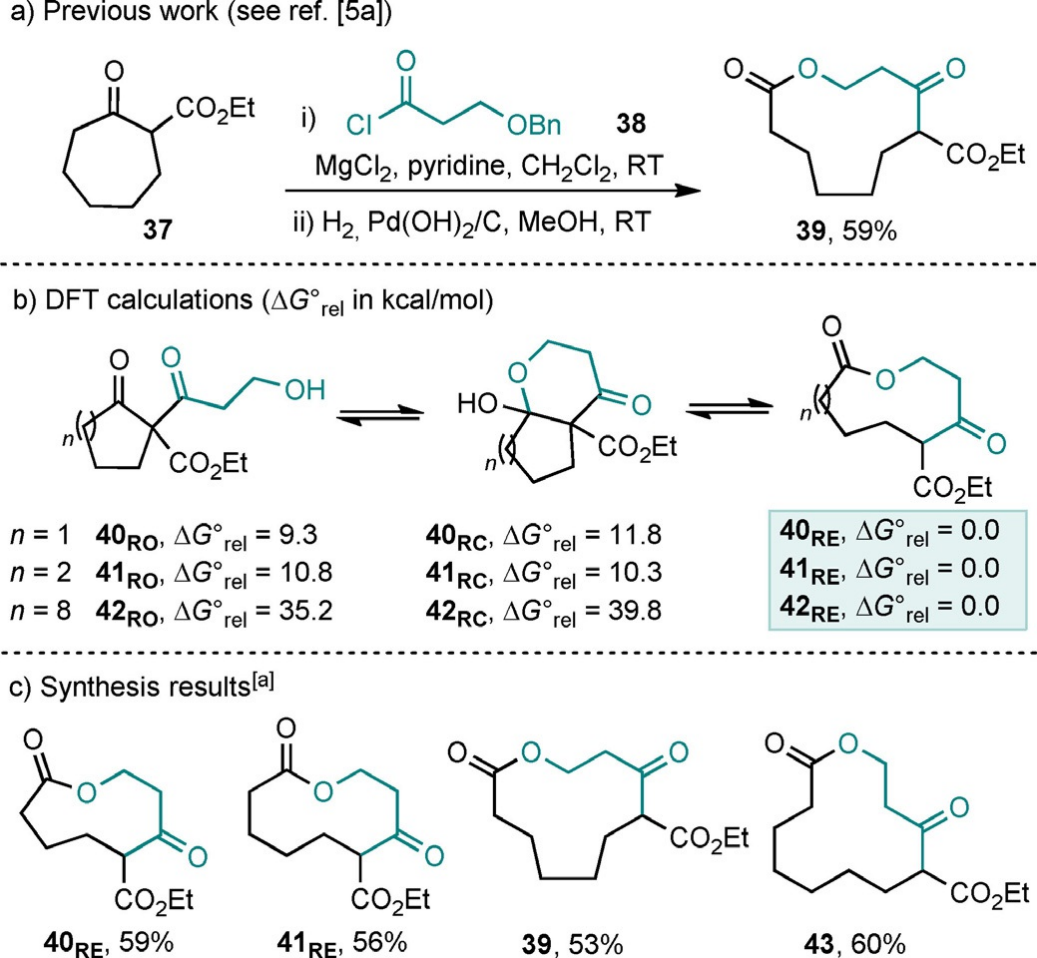
Ring‐size dependency of the outcome of the ring expansion of β‐ketoesters with β‐hydroxy acid chloride **38**. ΔG∘rel values are given in kcal mol^−1^. i) β‐ketoester, **38**, MgCl_2_, pyridine, CH_2_Cl_2_, RT; ii) Pd/C H_2_, EtOAc, 3 h, RT; NEt_3_, CHCl_3_, RT, 18 h.

The hydroxyacid‐based analogue of this cyclic β‐ketoester ring expansion was less well developed, with the expansion of seven‐membered **37** the only example of this type featured in our previous publications to have been performed on a simple cyclic β‐ketoester (Scheme 6 a). Given the importance of macrocyclic lactones in medicinal chemistry,^12^ we decided to test whether the scope of this variant could be expanded. As was done for the analogous amino acid system, DFT/B3LYP/6‐31G* calculations were performed to measure the energies of the equilibrating isomers of the five‐, six‐, and 12‐membered ring systems **40**–**42** (Scheme 6 b), which again suggested that there is a clear thermodynamic driving force for ring expansion. Pleasingly, the corresponding synthetic experiments all worked well, with five‐ to eight‐membered β‐ketoesters undergoing C‐acylation, hydrogenolysis and ring expansion to give ring‐expanded lactones **39**, **40_RE_**, **41_RE_** and **43** all in comparable yields (Scheme 6 c). In a small change to the published conditions shown in Scheme 6 a, we found that performing the hydrogenolysis in ethyl acetate (rather than methanol) and then stirring with triethylamine in chloroform led to superior reaction yields. The main reason the isolated yields are in the 50–60 % range (and not higher) is due to loss of material during the C‐acylation step (especially the work‐up, during which the magnesium salts can cause problems with phase separation) and these results are in line with typical yields in our previous papers.5a, 5b

**Scheme 7:**
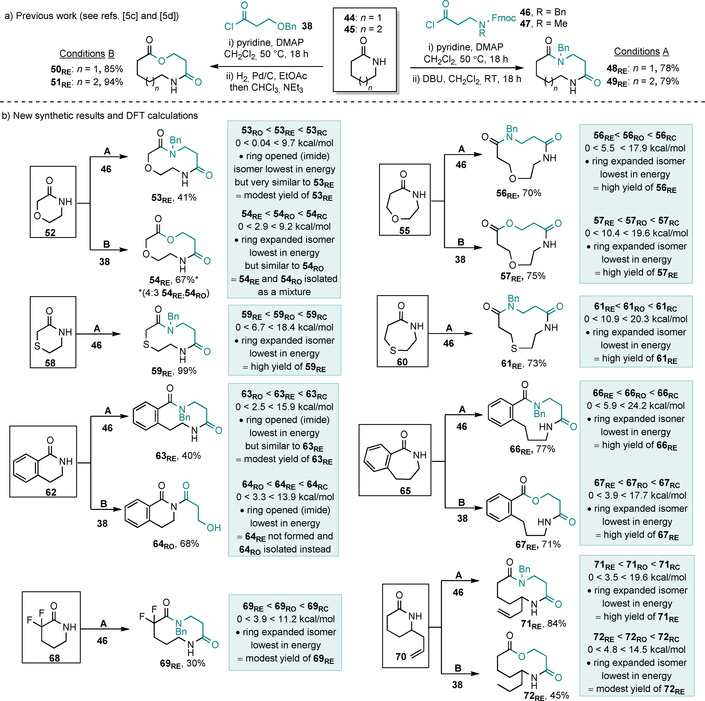
Lactam ring‐expansion reactions and DFT calculations. ΔG∘rel values are given in kcal mol^−1^.

We then went on to test other lactam‐based ring expansion systems with additional functionality present in the starting lactams. Hydroxyacid and amino acid derivatives **38** and **46** were used to exemplify the synthetic reactions, and in the calculations for **46**, a simplified N‐methyl (rather than N‐benzyl) derivative was used (i.e., from **47**) as this significantly reduced the computational time but was found to have very little impact on the calculations.^13^ Thus, we started by examining lactams containing α‐heteroatoms (**52**, **55**, **58** and **60**) with amino acid and hydroxyacid derivatives **38** and **46**. The analogous heteroatom‐free variants of these reactions had been tested in our earlier work (Scheme 7 a) and were shown to be high yielding. Therefore, based purely on our chemical intuition at this stage, we did not expect to see much variation upon switching to these new systems. However, starting from six‐membered lactam **52**, a much lower isolated yield (41 %) of the ring‐expanded product **53_RE_** was obtained in the amino acid series, while the ring‐expanded lactone **54_RE_** was isolated as an inseparable mixture with its ring‐opened imide form **54_RO_**. The calculations give clues as to why these reactions did not proceed well; for example, the ring‐opened and ring‐expanded isomers **53_RO_** and **53_RE_** were calculated to have very similar Gibbs free energies, thus suggesting that both may be formed in this reaction, although only the relatively non‐polar product **53_RE_** was isolated after chromatography, in modest yield. Compounds **54_RO_** and **54_RE_** were also calculated to be similar in free energy and in this case a mixture of products was isolated. Conversely, upon moving to seven‐membered starting material **55**, a clear preference for the ring‐expanded isomer was predicted by the calculations, which manifested in much improved synthetic yields for the desired ring‐expanded isomers (70 and 75 % for **56_RE_** and **57_RE_** respectively).

In contrast to oxygen‐containing **52** and **55**, sulfur‐containing lactams **58** and **60** both performed well in the synthetic ring‐expansion reactions with **46**;^14^ ring‐expanded products **59_RE_** and **61_RE_** were each formed in good yield. This was again mirrored in the calculations, with **59_RE_** and **61_RE_** calculated to be the lowest energy isomers in each case by clear margins. The difference in reactivity between **52** and **58**, which is presumably a result of some relatively subtle stereoelectronic effects and/or differences in bond lengths, is not something that we would have predicted without the calculations.

We also examined benzannulated, fluorinated and branched lactam starting materials **62**, **65**, **68** and **70**, and as before, the predictive ability of the calculations was retained. Indeed, the ability to predict when reactions will fail completely is also important; for example, the ring‐opened imide isomer **64_RO_** was calculated to be the most stable isomer in this series, and this was corroborated by the synthetic results.

In general, we have found that for systems in which the ring‐expanded isomer is calculated to be the lowest in energy by more than 3 kcal mol^−1^, then the reactions tend to work reliably. In cases where the free energy difference is less than 3 kcal mol^−1^, the reaction outcomes are less predictable, often giving low yields of ring‐expanded products and/or mixtures. The reactions to form ring‐expanded products **69_RE_** and **72_RE_**, which were isolated in modest 30 and 45 % yields, respectively, are outliers in terms of yield, but the lower yields in these cases simply reflect the fact that the N‐acylation step did not proceed to completion in either case. Indeed, an important caveat to keep in mind when using this DFT/B3LYP/6‐31G* method is that it only gives an indication of the chances of achieving a favourable equilibrium. It does not account for the efficiency of the synthetic steps that take place before the equilibrium, the possibility of off‐equilibrium side reactions or other kinetic effects.

**Figure 1:**
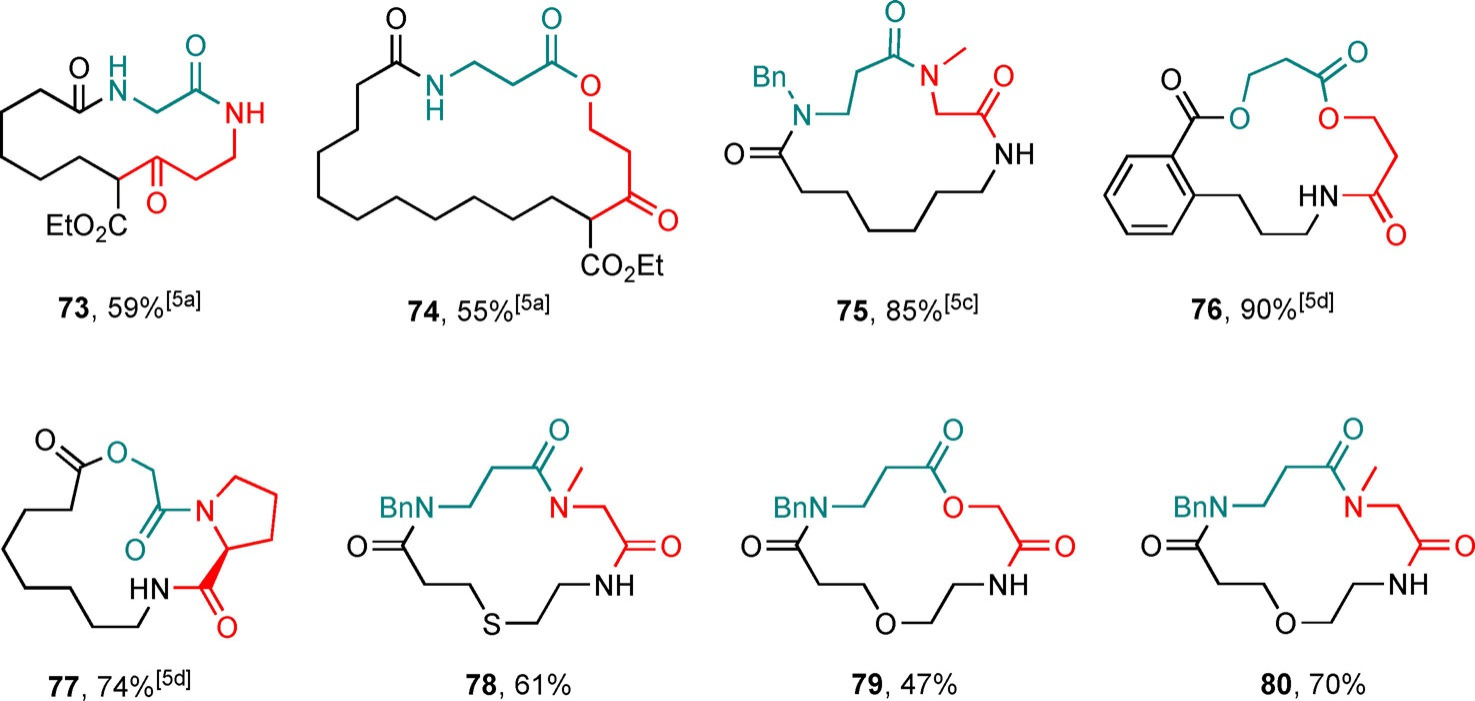
Successive ring expansion products.

As all the ring‐expanded products described in this manuscript were made using SuRE methods, they are all, in theory, potential starting materials for further ring‐expansion reactions. Representative examples of products (**73**–**77**) that have been expanded for a second time in our earlier work are shown in Figure 1, with the second linear fragment inserted highlighted in red. After undergoing one ring expansion, the rings should all be large enough that they are beyond the “switch on” point for any of the ring‐expansion reaction types that we have studied and calculated (not withstanding any effects resulting from the additionally added functional groups) and should therefore be thermodynamically favourable. This is corroborated by our work to date in which several successful successive ring‐expansion reactions are reported. This does not mean that performing additional iterations is always routine (e.g., in some cases, the acylation reactions can be more difficult on these more functionalised systems, sometimes requiring additional equivalents of acid chloride),5c, 5d but once acylation has been achieved, ring expansion is typically straightforward. Three new examples of doubly ring‐expanded products (**78**–**80**, see the Supporting Information for reaction conditions), based on new substrates made for the first time in this manuscript, have been performed and are reported here for completeness.

### Computational chemistry: Method evaluation

The DFT/B3LYP/6‐31G* methodology used has demonstrated, in both this and previous work,5d good success in predicting the outcome of SuRE reactions. Calculations at the B3LYP/6‐31G* level are relatively computationally efficient, but do not take into consideration effects such as solvation and dispersion. These additions are typically used to improve the accuracy of such calculations, therefore, we decided to benchmark their effects, along with a range of functionals, in order to determine any potential method‐effects in the calculations.

For this study general gradient approximation, GGA (BP86), hybrid (B3LYP and PBE0) and meta‐hybrid (M06 and M06‐2X) functionals were used. Solvation effects were applied using a PCM model with either dichloromethane or chloroform as relevant to simulate the reaction conditions. The effects of dispersion are inherently taken into consideration by the M06 and M06‐2X functionals.^15^ They were also applied using the Grimme's D3 method with Becke‐Johnson damping^16^ to a PBE0/def2‐TZVPP single‐point calculation, using the geometry and thermodynamic corrections from a BP86/SV(P) calculation; this method has been used successfully by our groups in previous projects,^17^ and also tests the effect of a large triple zeta basis set.^18^

Initially, a wide range of methods were benchmarked against structures **17**–**20**, by reoptimising the structures from the B3LYP/6‐31G* calculations and comparing the relative energies with the experimental outcomes (Table 1). Structures with which the ring‐closed isomer has a larger energy than the ring‐opened or ring‐expanded isomers (**17**, **19** and **20**), produced the most comparable results, with there being little difference when using GGA or hybrid functionals with the 6‐31G* basis set.

**Table 1:** Relative difference of Gibbs energies at 298 K for structures **17**–**20** at different levels of theory. Solvent corrections were applied using a PCM model. * Geometry from the BP86/SV(P) level.

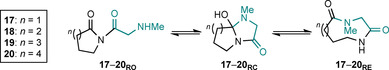								
Compound	Functional	Basis set	Solvent correction	Empirical dispersion correction	RO [kcal mol^−1^]	RC [kcal mol^−1^]	RE [kcal mol^−1^]	Yield RE [%]
**17** (*n*=1)	B3LYP	6‐31G*	N	N	0.0	16.5	1.9	0
	B3LYP	6‐31G*	PCM	N	0.0	14.9	0.2	
	BP86	6‐31G*	N	N	0.0	14.9	1.6	
	PBE0	6‐31G*	N	N	0.0	14.1	1.2	
	M06	6‐31G*	PCM	N	0.0	10.8	−2.1	
	M06‐2X	6‐31G*	PCM	N	0.0	8.7	−1.6	
	BP86	SV(P)	PCM	N	0.0	11.5	−2.1	
	PBE0*	def2‐TZVPP	PCM	D3(BJ)	0.0	9.2	−3.3	
								
**18** (*n*=2)	B3LYP	6‐31G*	N	N	0.0	3.9	2.1	0
	B3LYP	6‐31G*	PCM	N	0.0	2.2	−1.1	
	BP86	6‐31G*	N	N	0.0	0.5	−1.1	
	PBE0	6‐31G*	N	N	0.0	−0.6	−1.6	
	M06	6‐31G*	PCM	N	0.0	−3.0	−3.8	
	M06‐2X	6‐31G*	PCM	N	0.0	−4.5	−4.1	
	BP86	SV(P)	PCM	N	0.0	−1.2	−3.0	
	PBE0*	def2‐TZVPP	PCM	D3(BJ)	0.0	−3.0	−5.3	
								
**19** (*n*=3)	B3LYP	6‐31G*	N	N	0.0	6.4	0.7	0
	B3LYP	6‐31G*	PCM	N	0.0	6.2	−0.3	
	BP86	6‐31G*	N	N	0.0	5.5	−0.4	
	PBE0	6‐31G*	N	N	0.0	4.4	−1.3	
	M06	6‐31G*	PCM	N	0.0	1.1	−3.8	
	M06‐2X	6‐31G*	PCM	N	0.0	−0.5	−3.4	
	BP86	SV(P)	PCM	N	0.0	2.7	−1.8	
	PBE0*	def2‐TZVPP	PCM	D3(BJ)	0.0	0.7	−5.0	
								
**20** (*n*=4)	B3LYP	6‐31G*	N	N	7.3	14.1	0.0	82
	B3LYP	6‐31G*	PCM	N	9.9	16.1	0.0	
	BP86	6‐31G*	N	N	8.3	13.8	0.0	
	PBE0	6‐31G*	N	N	8.8	13.3	0.0	
	M06	6‐31G*	PCM	N	12.2	12.8	0.0	
	M06‐2X	6‐31G*	PCM	N	11.4	10.5	0.0	
	BP86	SV(P)	PCM	N	11.1	13.6	0.0	
	PBE0*	def2‐TZVPP	PCM	D3(BJ)	13.4	14.0	0.0	

Modelling the effects of solvation also had little effect on the relative energy differences when using the hybrid B3LYP functional. Comparable results are observed both with and without solvent corrections. However, this does not extend to the BP86/SV(P) calculations, with more significant relative energy differences observed when compared to the standard B3LYP/6‐31G* calculations, which appears to come from greater stabilisation of the ring‐closed and ring‐expanded isomers than the ring‐opened when solvent is included.

The effects of dispersion had the greatest impact on the expected outcomes of the experiments, with the M06, M06‐2X and D3(BJ)‐PBE0 calculations showing lower relative energies for the ring‐closed and ring‐expanded isomers, predicting that ring expansion should be comparatively more thermodynamically favourable in these examples, and in some cases contradicting the experimental results. We believe that due to the side chain present in the ring‐opened structures being directed away from the ring, there are fewer stabilising interactions present than compared to the ring‐closed or expanded isomers. As a consequence of these different molecule geometries, it appears that modelling the dispersion interactions may result in the stability of the ring‐expanded isomer being overpredicted when compared to the ring‐opened form. This alters the expected reaction outcome where the B3LYP/6‐31G* calculations predict these isomers to be similar in energy.

With dispersion effects having a large effect on the relative energy differences and the predicted thermodynamic outcomes on these examples, the study was extended to include these effects to several other systems, using the M06‐2X/6‐31G* methodology. A comparison between this method and B3LYP/6‐31G* is presented in Table 2. As observed with structures **17**–**20** (Table 1), the main difference between the two methods is that, when compared to the ring‐expanded form, the relative energies of the ring‐closed forms are lower at the M06‐2X/6‐31G* level (Δ_ave_=−5.2 kcal mol^−1^), and ring‐opened isomers increased (Δ_ave_=3.1 kcal mol^−1^). In most instances this doesn't change the expected outcome of the reaction, however, where there is a smaller difference in the energy of the ring‐opened and ring‐expanded isomers (see **53**, **63** and **64**), this does result in ring expansion being predicted to be favourable. Notably in some examples the intermediate ring‐closed isomer becomes lower in energy than the ring‐opened, however, this does not seem to correlate to any observable difference in how well the reaction proceeds experimentally (see **32**, **40** and **69** for examples).

**Table 2:** Relative difference of Gibbs energies at 298 K. Solvent corrections were applied using a PCM model with either dichloromethane or chloroform as relevant for the M06‐2X/6‐31G* calculations. See the Supporting Information for absolute energies. Blue numbers denotes the most significant differences between the two methods >3 kcal mol^−1^. Δ_ave_ is defined as the mean value of the energy at M06‐2X/6‐31G*—energy at B3LYP/6‐31G*.

Compound	Functional/	RO	RC	RE	Yield
	basis set	[kcal mol^−1^]	[kcal mol^−1^]	[kcal mol^−1^]	RE [%]
**31**	B3LYP/6‐31G*	10.0	12.6	0.0	675a
	M06‐2X/6‐31G*	12.5	10.2	0.0	
					
**32**	B3LYP/6‐31G*	8.1	9.7	0.0	825a
	M06‐2X/6‐31G*	10.5	3.7	0.0	
					
**33**	B3LYP/6‐31G*	36.6	45.4	0.0	805a
	M06‐2X/6‐31G*	38.0	34.7	0.0	
					
**40**	B3LYP/6‐31G*	9.3	11.8	0.0	59
	M06‐2X/6‐31G*	11.7	8.1	0.0	
					
**41**	B3LYP/6‐31G*	10.8	10.3	0.0	56
	M06‐2X/6‐31G*	10.3	3.7	0.0	
					
**42**	B3LYP/6‐31G*	35.2	39.8	0.0	–
	M06‐2X/6‐31G*	32.6	30.5	0.0	
					
**53**	B3LYP/6‐31G*	0.0	9.7	0.0	41
	M06‐2X/6‐31G*	6.9	7.1	0.0	
					
**54**	B3LYP/6‐31G*	2.9	9.2	0.0	67^[a]^
	M06‐2X/6‐31G*	5.0	4.5	0.0	
					
**56**	B3LYP/6‐31G*	5.5	17.9	0.0	70
	M06‐2X/6‐31G*	11.7	13.8	0.0	
					
**57**	B3LYP/6‐31G*	10.4	19.6	0.0	75
	M06‐2X/6‐31G*	11.2	13.8	0.0	
					
**59**	B3LYP/6‐31G*	6.7	18.4	0.0	99
	M06‐2X/6‐31G*	12.0	13.1	0.0	
					
**61**	B3LYP/6‐31G*	10.9	20.3	0.0	73
	M06‐2X/6‐31G*	14.5	15.1	0.0	
					
**63**	B3LYP/6‐31G*	−2.5	13.4	0.0	40
	M06‐2X/6‐31G*	2.8	10.0	0.0	
					
**64**	B3LYP/6‐31G*	−3.3	10.6	0.0	0
	M06‐2X/6‐31G*	0.5	6.8	0.0	
					
**66**	B3LYP/6‐31G*	5.9	24.2	0.0	77
	M06‐2X/6‐31G*	9.4	19.1	0.0	
					
**67**	B3LYP/6‐31G*	3.9	17.7	0.0	71
	M06‐2X/6‐31G*	6.0	12.8	0.0	
					
**69**	B3LYP/6‐31G*	3.9	11.2	0.0	30
	M06‐2X/6‐31G*	9.2	5.7	0.0	
					
**71**	B3LYP/6‐31G*	3.5	19.6	0.0	84
	M06‐2X/6‐31G*	9.8	14.5	0.0	
					
**72**	B3LYP/6‐31G*	4.8	14.5	0.0	45
	M06‐2X/6‐31G*	6.6	9.2	0.0	
					
	Δ_ave_	3.1	−5.2	0.0

[a] Isolated as a mixture (**54_RE_**/**54_RO_** 4:3).

Thus, for either method, both the B3LYP and M06‐2X functionals correctly predicts the expected reaction outcomes in the majority of cases, although on average, it is the B3LYP method that more closely correlates with the experimental findings, despite the fact that the M06‐2X functional usually performs better for organic molecules due to the inclusion of dispersion corrections.^15^, ^19^ Therefore, we believe that these results clearly demonstrate that the B3LYP/6‐31G* methodology is suitable as an aid for predicting the outcome of SuRE reactions, balancing computational efficiency with good prediction of reaction outcome. The observation that a greater than 3 kcal mol^−1^ energy difference between ring‐opened and ring‐expanded isomers is needed to more confidently predict the outcome of the reaction, is based upon the inherent computational accuracy of these calculations

## Conclusions

In summary, we have significantly expanded the scope of various classes of SuRE reaction, and have shown that the reaction outcomes can be predicted based on the relative Gibbs free energies of three isomeric species in equilibrium by using DFT calculations.^20^ Useful conclusions can also be drawn from the significantly expanded synthetic scoping reactions and a total of 48 new ring‐expanded products are reported in this manuscript. In most cases, the isomer calculated to be lowest in energy was the major product obtained in the corresponding synthetic results.

Of course, any computational predictive method of this type will never be 100 % accurate, especially given how difficult it is to model the properties and conformations of relatively flexible systems like macrocycles.^21^ In view of this, the approximations involved in the calculations and the possibility that kinetic effects might prevent equilibrium being reached in some reaction systems, we do not recommend using the calculations to make quantitative predictions on reaction yields or the Boltzmann distribution of the isomers in the presumed equilibria. The guideline that a free energy difference of more than 3 kcal mol^−1^ in favour of the ring‐expanded isomer when using the B3LYP/6‐31G* methodology usually leads to a successful reaction is a qualitative observation, that this was true in all such cases tested in which the preceding acylation step was efficient. It should not be considered a hard rule. However, as a guide to assessing the viability of new ring‐expansion reactions before embarking on synthetic effort, we do believe that this DFT/B3LYP/6‐31G* method, which is widely implemented across the vast majority of computational chemistry packages, has practical utility and will be useful in directing future synthetic efforts, in our group and others.

## Conflict of interest

The authors declare no conflict of interest.

## Supporting information

As a service to our authors and readers, this journal provides supporting information supplied by the authors. Such materials are peer reviewed and may be re‐organized for online delivery, but are not copy‐edited or typeset. Technical support issues arising from supporting information (other than missing files) should be addressed to the authors.

Supplementary
