# Supplementary material for: Evaluating the Viability of Successive Ring‐Expansions Based on Amino Acid and Hydroxyacid Side‐Chain Insertion
Source: Chemistry. 2020 Sep 11;26(55):12674–83. doi: 10.1002/chem.202002164 (PMC7589337; doi:10.1002/chem.202002164)
Supplement: Supplementary file 1 — Supplementary [file CHEM-26-12674-s001.pdf]

Chemistry–A European Journal

Supporting Information

Evaluating the Viability of Successive Ring-Expansions Based on Amino Acid and Hydroxyacid Side-Chain Insertion

Aggie Lawer,[a] Ryan G. Epton,[a] Thomas C. Stephens,[a] Kleopas Y. Palate,[a] Mahendar Lodi,[a] Emilie Marotte,[b] Katie J. Lamb,[a] Jade K. Sangha,[a] Jason M. Lynam,*[a] and William P. Unsworth*[a]

Table of Contents **Page**

**General information**	**1**
**List of starting materials**	**2**
**General procedure for acid chloride formation**	**4**
**Characterisation data, procedures and NMR spectra**	**4**
**Computational studies**	**127**
**References**	**466**

General information

Except where stated, all reagents were purchased from commercial sources and used without further purification. Except where stated, all experimental procedures were carried out under an atmosphere of argon. Anhydrous CH_2Cl_2 was obtained from an Innovative Technology Inc. PureSolv® solvent purification system. ^{1}H NMR and ^{13}C NMR spectra were recorded on a JEOL ECX400 or JEOL ECS400 spectrometer, operating at 400 MHz and 100 MHz, respectively. All spectral data was acquired at 295 K unless stated. Chemical shifts (δ) are quoted in parts per million (ppm). The residual solvent peak, δ_H 7.26 and δ_C 77.0 for $CDCl_3$ was used as a reference. Coupling constants (*J*) are reported in Hertz (Hz) to the nearest 0.1 Hz. The multiplicity abbreviations used are: s singlet, d doublet, t triplet, q quartet, m multiplet. Signal assignment was achieved by analysis of DEPT, COSY, HMBC and HMQC experiments where required. Infrared (IR) spectra were recorded on a PerkinElmer UATR two spectrometer as a thin film. Mass-spectra (low and high-resolution) were obtained by the University of York Mass Spectrometry Service, using electrospray ionisation (ESI) on a Bruker Daltonics, Micro-tof spectrometer. Melting points were determined using Gallenkamp apparatus and are uncorrected. Thin layer chromatography was carried out on Merck silica gel 60F254 pre-coated aluminium foil sheets and were visualised using UV light (254 nm) and stained with basic aqueous potassium permanganate. Flash column chromatography was carried out using slurry packed Fluka silica gel (SiO_2), 35–70 µm, 60 Å, under a light positive pressure, eluting with the specified solvent system.

List of starting materials

All the starting materials used in this publication are listed below. Commercially available starting materials (denoted with a *) were used as supplied, those with a reference number are were prepared via the cited literature method, while for all others, preparative details and spectroscopic characterisation data are provided.

Lactams

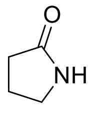
S1*

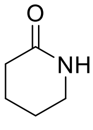
S2*

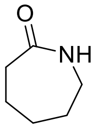
S3*

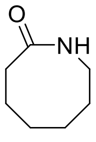
S4*

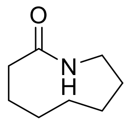
S5[1]

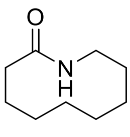
S6[1]

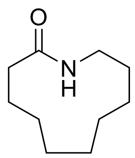
S7[1]

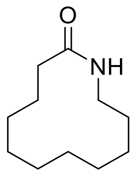
S8[1]

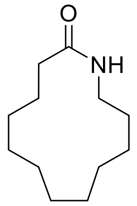
14*

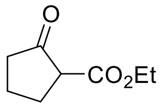
S9*

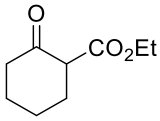
S10*

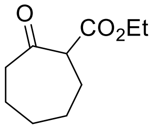
37[2]

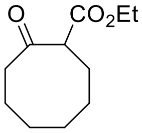
S11[2]

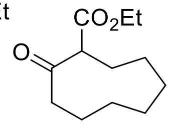
S12[1]

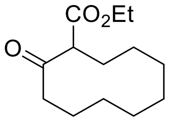
S13[1]

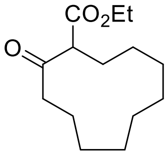
S14

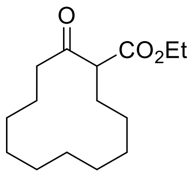
S15[2]

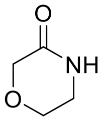
52[3]

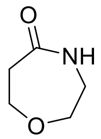
55[4]

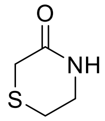
58[5]

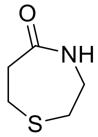
60

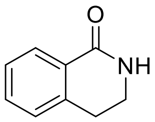
62*

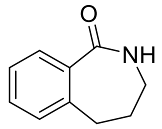
65*

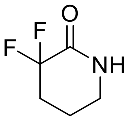
68[6]

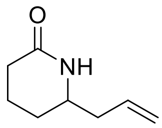
70[7]

Linear carboxylic acids S16−S37 used to make acid chlorides

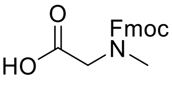

S16*
→ ROCl **15**

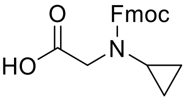

S17[1]
→ ROCl **26a**

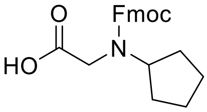

S18[1]
→ ROCl **26b**

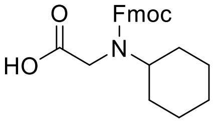

S19[1]
→ ROCl **26c**

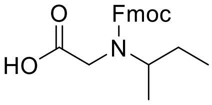

S20[1]
→ ROCl **26d**

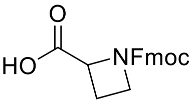

S21[1]
→ ROCl **26e**

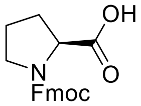

S22*
→ ROCl **26f**

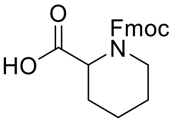

S23[1]
→ ROCl **26g**

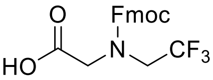

S24
→ ROCl **26h**

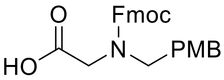

S25[1]
→ ROCl **26i**

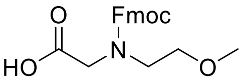

S26
→ ROCl **26j**

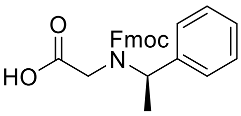

S27
→ ROCl **26k**

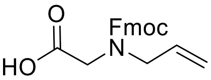

S28
→ ROCl **26l**

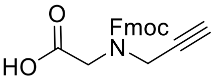

S29
→ ROCl **26m**

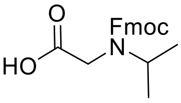

S30[8]
→ ROCl **26n**

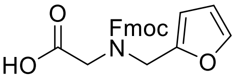

S31
→ ROCl **26o**

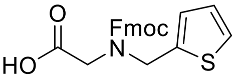

S32
→ ROCl **26p**

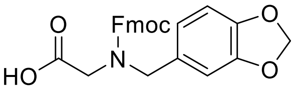

S33
→ ROCl **26q**

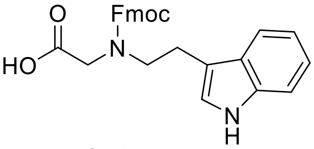

S34
→ ROCl **26r**

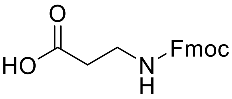

S35[1]
→ ROCl **29**

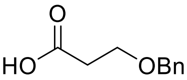

S36[1]
→ ROCl **38**

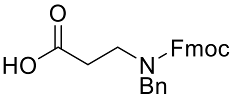

S37[1]
→ ROCl **46**

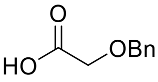

S38[1]
→ ROCl **S39**

General procedure for acid chloride formation

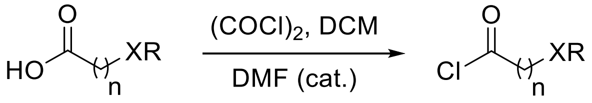

Oxalyl chloride (3 mmol) was added to a suspension of carboxylic acid (1 mmol) in DCM (5 mL), followed by a catalytic amount of DMF (1 drop/mmol of carboxylic acid). The resulting mixture was stirred at RT for 1 h and concentrated *in vacuo* to remove all the solvent and excess oxalyl chloride.

Characterisation data, procedures and NMR spectra

1,4-Oxazepan-5-one (55)

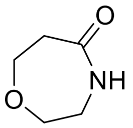

Hydroxylamine hydrochloride (2.70 g, 38.9 mmol) and sodium hydrogen carbonate (8.33 g, 99.0 mmol) were mixed in a two-neck flask. Ethanol (36 mL) and tetrahydro-4*H*-pyran-4-one (3.00 g, 30.0 mmol) were added dropwise at 0 °C. After completion of the reaction, ethanol was evaporated. Water was added and the aqueous layer was extracted with ethyl acetate (3 ×). The combined organic layers were dried over $MgSO_4$ and concentrated to give the crude oxime which was carried to the next step without further purification. Acetone (120 mL) was added to the crude product, followed by an aqueous solution of sodium carbonate (9.54 g, 90.0 mmol) in water (127 mL) at r.t. The reaction mixture was stirred for 5 mins. A solution of *p*-toluenesulfonyl chloride (8.58 g, 45.0 mmol) in acetone (32 mL) was added slowly and the reaction mixture was stirred at r.t. overnight. Acetone was removed and water was added. The mixture was extracted with DCM (5 ×). The combined organic layers were dried over $MgSO_4$, filtered and concentrated to give the title compound as a white solid (1.68 g, 48%); δ_H (400 MHz, $CDCl_3$) 3.67−3.64 (m, 2H, CH_2), 3.62−3.60 (m, 2H, CH_2), 3.20−3.17 (m, 2H, CH_2), 2.56−2.54 (m, 2H, CH_2). Data consistent with those reported in the literature.[5]

1,4-Thiazepan-5-one (60)

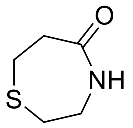

Hydroxylamine hydrochloride (2.30 g, 33.5 mmol) and sodium hydrogen carbonate (7.16 g, 85.1 mmol) were mixed in a two-neck flask. Ethanol (32 mL) and tetrahydro-4*H*-thiopyran-4-one (3.00 g, 25.8 mmol) were added dropwise at 0 °C. After completion of the reaction, ethanol was evaporated. Water was added and the aqueous layer was extracted with ethyl acetate (3 ×). The combined organic layers were dried over $MgSO_4$ and concentrated to give the crude oxime which was carried to the next step without further purification. Acetone (102 mL) was added to the crude product, followed by an aqueous solution of sodium carbonate (8.20 g, 77.4 mmol) in water (110 mL) at r.t. The reaction mixture was stirred for 5 mins. A solution of *p*-toluenesulfonyl chloride (7.40 g, 38.7 mmol) in acetone (28 mL) was added slowly and the reaction mixture was stirred at r.t. overnight. Acetone was removed and water was added. The mixture was extracted with DCM (5 ×). The combined organic layers were dried over $MgSO_4$, filtered and concentrated to give the title compound as a brown crystalline solid (3.23 g, 95%); m.p. 81−85 °C; ν_{max}/cm^{-1} (thin film) 3219, 3082, 2908, 2244, 1650, 1481, 1431, 1403, 1350, 1299, 1239, 1140, 1108, 1014; δ_H (400 MHz, $CDCl_3$) 7.53 (1H, br s, NH), 3.54 (2H, m, CH_2-NH), 2.85 (2H, t, *J* = 5.3 Hz, CH_2-CO), 2.69−2.63 (m, 4H, C$\mathbf{H_2}$-S-C$\mathbf{H_2}$); δ_C (100 MHz, $CDCl_3$) 177.8 (CO), 45.5 (CO-NH), 40.7 (**C**H_2-CO), 31.2 (SCH_2), 24.3 (S**C**H_2-CO); HRMS (ESI): calcd. for C_5H_9NNaOS, 154.0297. Found; $[MNa]^+$, 154.0303 (-4.1 ppm error).

Ethyl 2-oxocycloundecane-1-carboxylate (S14)

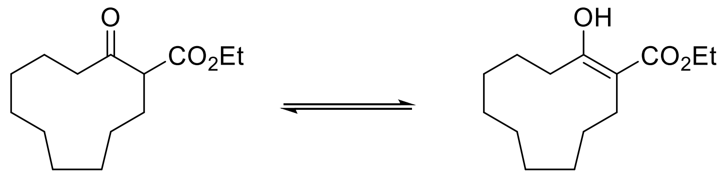

To a solution of cyclodecanone (4.86 g, 31.5 mmol) in Et_2O (60 mL) at 0 °C, was added a solution of $BF_3.OEt_2$ (5.93 mL, 47.3 mmol) in Et_2O (10 mL) over 5 mins. A solution of ethyl diazoacetate (87% wt. in CH_2Cl_2, 5.79 mL, 47.3 mmol) in Et_2O (10 mL), was then added over a period of 15 mins, causing a vigorous evolution of gas. The resulting solution was allowed to warm to RT under an argon atmosphere over 16 h, where the mixture was cooled to 0 °C and neutralized with sat. aq. solution of $NaHCO_3$. The resulting mixture was extracted with $CHCl_3$ (3 × 100 mL) and the combined organic extracts dried over $MgSO_4$ and concentrated *in vacuo*. Purification by flash column chromatography (SiO_2, 20:1 hexane:ethyl acetate) afforded the title compound (as a 2:1 mixture of ketone:enol tautomers) as a yellow oil (5.84 g, 77%); R_f 0.81&0.77(CH_2Cl_2); ν_{max}/cm^{-1} (thin film) 2929, 2865, 1745, 1710, 1638, 1603; δ_H (400 MHz, $CDCl_3$) 12.96 (1H, s, O**H**, enol tautomer), 4.26–4.07 (4H, m, C**H₂**O, both tautomers), 3.67 (1H, dd, *J* = 10.5, 2.9 Hz, C**H**CO_2Et, keto tautomer), 2.74 (1H, ddd, *J* = 16.4, 9.2, 3.2 Hz, C**H**H′, keto), 2.58 (1H, ddd, *J* = 16.4, 9.2, 3.2 Hz, CH**H′**, keto), 2.53–2.42 (2H, m, C**H**H′, both), 2.38 (1H, t, *J* = 6.3 Hz, CH**H′**, keto), 2.35–2.30 (1H, m, CH**H′**, enol), 2.14–2.02 (1H, m, C**H**H′, keto), 1.97–1.87 (1H, m, CH**H′**, keto), 1.87–1.14 (34H, m, both); δ_C (100 MHz, $CDCl_3$) data for the keto tautomer only: 208.6 (**C**O), 170.0 (**C**O), 61.4 (**C**H_2O), 58.2 (**C**HCO_2Et), 41.5 (**C**H_2CO), 26.5 (**C**H_2), 25.6 (**C**H_2), 25.0 (**C**H_2), 24.69 (**C**H_2), 24.67 (**C**H_2), 24.4 (**C**H_2), 24.3 (**C**H_2), 22.5 (**C**H_2), 14.2 (**C**H_3); HRMS (ESI): calcd. for $C_{14}H_{24}NaO_3$, 263.1618. Found: $[MNa]^+$, 263.1615 (1.1 ppm error).

Linear carboxylic acids **S17-S25** were synthesised according to the procedures described in the literature[1] to prepare similar type of linear carboxylic acids.

***N*-(((9*H*-Fluoren-9-yl)methoxy)carbonyl)-*N*-(2-methoxyethyl)glycine (S26)**

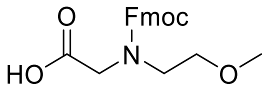

A solution of ethyl bromoacetate (10.0 mmol, 1.11 mL) in dry THF (5 mL) was added dropwise to a solution of 2-methoxyethylamine (22 mmol, 1.91 mL) in dry THF (5 mL) at 0 °C. The mixture was allowed to warm to r.t. and stirred for 3 h (following by TLC). The solvent was then removed *in vacuo* to afford the crude amino ester as a yellowish oil. NaOH (4N aq., 8.2 mL) was added to a solution of amino acetate in 1,4-dioxane (35 mL) and methanol (12.5 mL). The reaction was stirred at RT for 2.5 h and analysed by TLC. The solvent was removed in vacuo to yield crude the *N*-alkyl amino acid as the sodium salt. The *N*-alkyl amino acid salt was dissolved in a mixture of deionized water (30 mL), 1,4-dioxane (50 mL), and Na_2CO_3 (50 ml, 10% solution). The solution was cooled to 0 °C whilst stirring and a pre-made solution of 9-fluorenylmethyl chloroformate (8.67 g, 33.5 mmol) dissolved in 1,4-dioxane (28 mL) added slowly. The solution was allowed to warm to RT and stirred for 18 h, analysed by TLC. The mixture was diluted with water (100 mL) and acidified to pH 2 using 10% aq. HCl and extracted with EtOAc (3 × 50 mL). The combined organics were washed with brine (50 mL), dried over $MgSO_4$ and concentrated in vacuo. Column chromatography (SiO_2, 1:4 ethyl acetate: hexane → 1:1 ethyl acetate: hexane) afforded the *title compound* (as an 1:1 mixture of rotamers) as a yellow oil which crystalizes under vacuum, (2.40 g, 68% over 3 steps); R_f 0.23 (9:1 ethyl acetate: methanol) (streaky concentration spot with concentration dependence); ν_{max}/cm^{-1} (neat) 2937, 1700, 1450, 1118, 908, 727; δ_H (400 MHz, $CDCl_3$) 10.58 (2H, br s, COO**H**, both rotamers) 7.75 (2H, d, *J* = 7.5 Hz, Ar**H**, single rotamer), 7.72 (2H, d, *J* = 7.5 Hz, Ar**H**, single rotamer), 7.58 (2H, d, *J* = 7.4 Hz, Ar**H**, single rotamer), 7.54 (2H, d, *J* = 7.4 Hz, Ar**H**, single rotamer), 7.42 – 7.25 (8H, m, Ar**H**, both), 4.54 (2H, d, *J* = 6.1 Hz, CHC**H₂**CO₂N, rotamer A), 4.43 (2H, d, *J* = 6.5 Hz, CHC**H₂**CO₂N, rotamer B), 4.25 (1H, t, *J* = 6.1 Hz, C**H**CH₂CO₂N, rotamer A), 4.19 (1H, t, *J* = 6.5 Hz, C**H**CH₂CO₂N, rotamer B), 4.13 (2H, s, C**H₂**COOH, single rotamer), 4.06 (2H, s, C**H₂**COOH, single rotamer), 3.54 (4H, s, N**CH₂**CH₂OCH₃, and NCH₂**CH₂**OCH₃, overlapping protons signals from single rotamer), 3.36 (2H, t, *J* = 5.2 Hz N**CH₂**CH₂OCH₃, single rotamer), 3.29 (3H, s, NCH₂CH₂O**CH₃**, single rotamer), 3.25 (2H, t, *J*

= 5.2 Hz, NCH_2**CH_2**OCH_3, single rotamer), 3.22 (3H, s, NCH_2CH_2O**CH_3**, single rotamer); δ_C (100 MHz, $CDCl_3$) 174.7 (**C**OOH), 156.4 (N**C**OOCH$_2$CH), 156.0 (N**C**OOCH$_2$CH), 143.9 (Ar**C**), 143.8 (Ar**C**), 141.4 (Ar**C**), 141.3 (Ar**C**), 127.8 (Ar**C**H), 127.7 (Ar**C**H), 127.2 (Ar**C**H), 127.1 (Ar**C**H), 124.9 (Ar**C**H), 124.9 (Ar**C**H), 120.0 (Ar**C**H), 71.7 (**C**H$_2$), 71.4 (**C**H$_2$), 68.0 (NCOO**C**H$_2$), 67.6 (NCOO**C**H$_2$), 58.7 (O**C**H$_3$), 50.2 (N**C**H$_2$COOH), 50.1 (N**C**H$_2$COOH), 48.7 (**C**H$_2$), 48.2 (**C**H$_2$), 47.2 (**C**HCH$_2$CO$_2$N), 47.2 (**C**HCH$_2$CO$_2$N); HRMS (ESI): calcd. for $C_{20}H_{21}NNaO_5$, 378.1312. Found: $[MNa]^+$, 378.1310 (0.4 ppm error).

_N_-(((9_H_-Fluoren-9-yl)methoxy)carbonyl)-((1_R_)-1-phenylethyl)amino)acetic acid (S27)

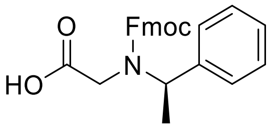

A solution of (*R*)-(+)-1-phenylethylamine (2.80 mL, 22 mmol) in dry THF (5 mL) was added dropwise to a stirring solution of ethyl bromoacetate (1.12 mL, 10 mmol) in dry THF (5 mL) at 0 °C. The reaction mixture was left stirring for 2 h at room temperature, staying a clear colourless solution throughout. This reaction mixture was concentrated *in vacuo* to afford a yellow oil. Diethyl ether (20 mL) was added to the oil and stirred to from a suspension (by causing precipitation of polar salts). The suspension was then filtered and washed with diethyl ether (3 x 10 mL). The collected solute was then concentrated *in vacuo* to afford the desired amino ester intermediate as yellow oil (1.84 g, 88%). Sodium hydroxide (4N aq., 2.5 mL, 10 mmol) was added to a mixture of the amino ester intermediate in 1,4-dioxane (35 mL) and methanol (12.5 mL) and left stirring at room temperature for 1 h. The reaction mixture was then concentrated *in vacuo* to afford the carboxylic acid sodium salt as a white solid (no mass was recorded or analysis performed for this product). The sodium salt intermediate was then dissolved in a mixture of water (30 mL), 1,4-dioxane (50 mL) and 10% aq. Na_2CO_3 (50 mL) and cooled to 0 °C. A pre-made solution of 9-fluorenylmethyl chloroformate (3.10 g, 12.0 mmol) dissolved in 1,4-dioxane (10 mL) was added dropwise to the sodium salt solution at 0 °C. The solution was then left to warm to room temperature and left stirring for 2 days. The reaction mixture was then diluted with water (50 mL), acidified to approx. pH 2 using 10% aq. HCl and extracted with ethyl acetate (3 × 50 mL). The combined organic layers were washed with sat. aq. brine (2 × 50 mL), dried ($MgSO_4$), filtered and concentrated *in vacuo* to afford a yellow oil (5.60 g). Purification by flash column chromatography (4:1→1:1 ethyl

acetate:hexane) afforded the *title compound* (as an 1.1:1 mixture of rotamers) as a white oil which dried to a white solid over time (2.13 g, 52% over three steps); R_f 0.42 (9:1 ethyl acetate:methanol); ν_{max}/cm^{-1} (neat) 3064, 3038, 2978, 2941, 1698, 1446, 1207, 1091, 758, 738; δ_H (400 MHz, $CDCl_3$) 7.75–7.68 (4H, m, Ar**H**), 7.54–7.50 (4H, m, Ar**H**), 7.41 – 7.23 (16H, m, Ar**H**, both), 7.14 – 7.10 (2H, m, Ar**H**, both), 5.59 (1H, q, *J* = 7.3 Hz, NC**H**(CH_3)Ar, rotamer A), 5.29 (1H, q, *J* = 7.1 Hz, NC**H**(CH_3)Ar, rotamer B), 4.62 – 4.49 (4H, m, CHC**H₂**CO₂N, both rotamers), 4.27 (1H, t, *J* = 6.3 Hz, C**H**CH_2CO_2N), 4.17 (1H, t, *J* = 6.3 Hz, C**H**CH_2CO_2N), 3.94 (1H, d, *J* = 17.9 Hz, C**H**H′COOH, rotamer B), 3.71 (1H, d, *J* = 18.6 Hz, C**H**H′COOH, rotamer A), 3.54 (1H, d, *J* = 17.9 Hz, CH**H′**COOH, rotamer B), 3.42 (1H, d, *J* = 18.6 Hz, CH**H′**COOH, rotamer A), 1.46 (6H, dd, *J* = 12.6, 7.1 Hz, C**H₃**, both rotamers); δ_C (100 MHz, $CDCl_3$) data for both rotamers, 175.4 (**C**OOH), 175.0 (**C**OOH), 156.5 (N**C**OOCH₂CH), 156.2 (N**C**OOCH₂CH), 143.8 (4C, Ar**C**), 141.4 (4C, Ar**C**), 140.2 (Ar**C**), 140.1 (Ar**C**), 128.8 (2C, ArCH), 128.7 (2C, Ar**C**H), 127.8 (4C, Ar**C**H), 127.7 (2C, Ar**C**H), 127.5 (2C, Ar**C**H), 127.21 (2C, Ar**C**H), 127.15 (2C, Ar**C**H), 127.0 (2C, Ar**C**H), 125.0 (4C, Ar**C**H), 120.1 (4C, Ar**C**H), 67.9 (NCOO**C**H₂), 67.7 (NCOO**C**H₂), 54.2 (N**C**HAr), 53.3 (N**C**HAr), 47.4 (2C, C**H**CH₂CO₂N), 44.9 (N**C**H₂COOH,), 44.1 (N**C**H₂COOH), 17.5 (**C**H₃), 16.6 (**C**H₃); HRMS (ESI): calcd. for $C_{25}H_{23}NNaO_4$, 424.1519. Found: $[MNa]^+$, 424.1519 (0.0 ppm error).

***N*-[(9*H*-fluoren-9-ylmethoxy)carbonyl]-*N*-2-propen-1-yl-glycine (S28)**

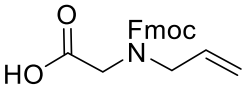

A solution of allylamine (1.65 mL, 22 mmol) in dry THF (5 mL) was added dropwise to a stirring solution of ethyl bromoacetate (1.12 mL, 10 mmol) in dry THF (5 mL) at 0 °C. The reaction mixture was left stirring for 2 h at room temperature, staying a clear colourless solution throughout. This reaction mixture was concentrated *in vacuo* to afford a yellow oil. Diethyl ether (20 mL) was added to the oil and stirred to from a suspension (by causing precipitation of polar salts). The suspension was then filtered and washed with diethyl ether (3 x 10 mL). The collected solute was then concentrated *in vacuo* to afford the desired amino ester intermediate as a pale yellow oil (1.27 g, 88%). Sodium hydroxide (4N aq., 2.5 mL, 10 mmol) was added to a mixture of the amino ester intermediate in 1,4-dioxane (35 mL) and methanol (12.5 mL) and left stirring at room temperature for 1 h. The reaction mixture was then

concentrated *in vacuo* to afford the carboxylic acid sodium salt as an orange solid (no mass was recorded or analysis performed for this product). The sodium salt intermediate was then dissolved in a mixture of water (30 mL), 1,4-dioxane (50 mL) and 10% aq. Na_2CO_3 (50 mL) and cooled to 0 °C. A pre-made solution of 9-fluorenylmethyl chloroformate (3.10 g, 12 mmol) dissolved in 1,4-dioxane (10 mL) was added dropwise to the sodium salt solution at 0 °C. The solution was then left to warm to room temperature and left stirring for 2 days. The reaction mixture was then diluted with water (50 mL), acidified to approx. pH 2 using 10% aq. HCl and extracted with ethyl acetate (3 × 50 mL). The combined organic layers were washed with sat. aq. brine (2 × 50 mL), dried ($MgSO_4$), filtered and concentrated *in vacuo* to afford a yellow oil (5.76 g). Purification by flash column chromatography (4:1 ethyl acetate:hexane → 1:1 ethyl acetate:hexane, ethyl acetate) afforded *N*-[(9H-fluoren-9-ylmethoxy)carbonyl]-*N*-2-propen-1-yl-glycine as a yellow oil which dried to a yellow solid overtime (1.98 g, 58% over three steps); R_f 0.16 (9:1 ethyl acetate:methanol); ν_{max}/cm^{-1} (neat) 3062-3014, 2941-2862, 1714, 1704, 1448, 1418, 1224, 761, 741; δ_H (400 MHz, $CDCl_3$) 9.02 (br s, 1 H), 7.78 (d, *J* = 7.5 Hz, 1 H), 7.75 (d, *J* = 7.8 Hz, 1 H), 7.61 (d, *J* = 7.5 Hz, 1 H), 7.57 (d, *J* = 7.4 Hz, 1 H), 7.44–7.29 (m, 4 H), 5.88–5.64 (m, 1 H), 5.21–5.12 (m, 2 H), 4.49 (d, *J* = 6.6 Hz, 2 H), 4.27 (t, *J* = 6.7 Hz, 0.5 H), 4.22 (t, *J* = 6.3 Hz, 0.5 H), 4.07 (s, 1 H), 4.02–3.98 (m, 2H), 3.92 (s, 1 H); δ_C (100 MHz, $CDCl_3$) 174.1, 174.0, 156.5, 156.0, 143.8, 141.3, 132.8, 132.6, 127.7, 127.0, 125.0, 124.9, 120.0, 118.3, 117.7, 68.0, 67.9, 50.7, 50.4, 47.8, 47.1; HRMS (ESI): calcd. for $C_{20}H_{19}NNaO_4$, 360.1206. Found: MNa^+, 360.1204 (1.5 ppm error).

_N_-(9-fluorenylmethoxycarbonyl)-_N_-(propargyl)glycine (S29)

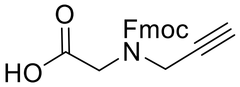

A solution of propargylamine (2.82 mL, 44 mmol) in dry THF (3 mL) was added dropwise to a stirring solution of ethyl bromoacetate (2.24 mL, 20 mmol) in dry THF (10 mL) at 0 °C. The reaction mixture was left stirring for 3 days at room temperature, forming an orange solution with a suspension within 1 h. This reaction mixture was concentrated *in vacuo* to afford an orange oil. Diethyl ether (20 mL) was added to the oil and stirred to from a suspension (by causing precipitation of polar salts). The suspension was then filtered and washed with diethyl ether (3 × 10 mL). The collected solute was then concentrated *in vacuo* to afford the desired amino ester intermediate as an orange oil (2.65 g, 93%). Sodium hydroxide (4N aq., 5 mL, 20

mmol) was added to a mixture of the amino ester intermediate in 1,4-dioxane (35 mL) and methanol (12.5 mL) and left stirring at room temperature for 1 h. The reaction mixture was then concentrated *in vacuo* to afford the carboxylic acid sodium salt as an orange solid (no mass was recorded or analysis performed for this product). The sodium salt intermediate was then dissolved in a mixture of water (30 mL), 1,4-dioxane (50 mL) and 10% aq. Na_2CO_3 (50 mL) and cooled to 0 °C. A pre-made solution of 9-fluorenylmethyl chloroformate (6.19 g, 24 mmol) dissolved in 1,4-dioxane (10 mL) was added dropwise to the sodium salt solution at 0 °C. The solution was then left to warm to room temperature and left stirring for 2 days. The reaction mixture was then diluted with water (50 mL), acidified to approx. pH 2 using 10% aq. HCl and extracted with ethyl acetate (3 × 50 mL). The combined organic layers were washed with sat. aq. brine (2 × 50 mL), dried ($MgSO_4$), filtered and concentrated *in vacuo* to afford a dark orange oil (10.5 g). Purification by column chromatography (4:1 ethyl acetate:hexane, 3:1 ethyl acetate:hexane, 2:1 ethyl acetate:hexane, 1:1 ethyl acetate:hexane, ethyl acetate) afforded *N*-(9-fluorenylmethoxycarbonyl)-*N*-(propargyl)glycine as a slightly orange oil which then solidified to a white solid with a hint of yellow over time (3.31 g, 49% over three steps). R_f = 0.39 (9:1 ethyl acetate:methanol); ν_{max}/cm^{-1} (neat) 3287, 3020-2952, 1703, 1450, 1234, 734; δ_H (400 MHz, $CDCl_3$) 10.14 (br s, 1 H), 7.78 (d, *J* = 7.5 Hz, 1 H), 7.73 (d, *J* = 7.5 Hz, 1 H), 7.63 (d, *J* = 7.5 Hz, 1 H), 7.52 (d, *J* = 7.5 Hz, 1 H), 7.44–7.27 (m, 4 H), 4.51–4.47 (m, 2 H), 4.32–4.20 (m, 4H), 4.09 (s, 1H), 2.35 (t, *J* = 2.5 Hz, 0.4 H), 2.29 (t, *J* = 2.5 Hz, 0.5 H); δ_C (100 MHz, $CDCl_3$) 175.1, 158.8, 155.5, 143.7, 141.4, 127.9, 127.9, 127.2, 127.2, 120.2, 120.1, 77.9, 73.7, 73.6, 47.3, 47.1, 46.6, 37.3, 37.2; HRMS (ESI): calcd. for $C_{20}H_{17}NNaO_4$, 358.1050. Found: MNa^+, 358.1038 (4.0 ppm error).

_N_-(((9_H_-Fluoren-9-yl)methoxy)carbonyl)-_N_-(furan-2-ylmethyl)glycine (S31)

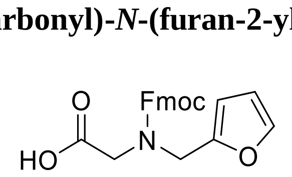

A solution of furfurylamine (1.94 mL, 22.0 mmol) in dry THF (5 mL) was added dropwise to a stirring solution of ethyl bromoacetate (1.12 mL, 10 mmol) in dry THF (5 mL) at 0 °C. The reaction mixture was left stirring for 2 h at r.t., staying a clear colourless solution throughout. This reaction mixture was concentrated *in vacuo* to afford a yellow oil. Diethyl ether (20 mL) was added to the oil and stirred to from a suspension (by causing precipitation of polar salts). The suspension was then filtered and washed with diethyl ether (3 × 10 mL). The collected

solute was then concentrated *in vacuo* to afford the desired amino ester intermediate as an orange oil (1.54 g, 83%). Sodium hydroxide (4N aq., 2.5 mL, 10 mmol) was added to a mixture of the amino ester intermediate in 1,4-dioxane (35 mL) and methanol (12.5 mL) and left stirring at room temperature for 1 h. The reaction mixture was then concentrated *in vacuo* to afford the carboxylic acid sodium salt as an orange solid (no mass was recorded or analysis performed for this product). The sodium salt intermediate was then dissolved in a mixture of water (30 mL), 1,4-dioxane (50 mL) and 10% aq. Na_2CO_3 (50 mL) and cooled to 0 °C. A pre-made solution of 9-fluorenylmethyl chloroformate (3.10 g, 12 mmol) dissolved in 1,4-dioxane (10 mL) was added dropwise to the sodium salt solution at 0 °C. The solution was then left to warm to room temperature and left stirring for 2 days. The reaction mixture was then diluted with water (50 mL), acidified to approx. pH 2 using 10% aq. HCl and extracted with ethyl acetate (3 x 50 mL). The combined organic layers were washed with sat. aq. brine (2 x 50 mL), dried ($MgSO_4$), filtered and concentrated *in vacuo* to afford an orange oil (8.81 g). Purification by flash column chromatography (4:1 ethyl acetate:hexane→ethyl acetate) afforded the title compound as an orange oil which dried to an orange solid overtime (1.87 g, 49 % over 3 steps); R_f 0.40 (9:1 ethyl acetate:methanol); ν_{max}/cm^{-1} (neat) 3062, 1737, 1673, 1478, 1269, 1174, 912, 745; δ_H (400 MHz, $CDCl_3$) 9.69 (2H, br s, COO**H**, both rotamers) 7.77 (2H, d, *J* = 7.5 Hz, Ar**H**, major), 7.72 (2H, d, *J* = 7.5 Hz, Ar**H**, minor), 7.61 (2H, dd, *J* = 7.5, 1.0 Hz, Ar**H**, major), 7.53 (2H, dd, *J* = 7.6, 1.1 Hz, Ar**H**, minor), 7.43–7.26 (10H, m, Ar**H** [fluorene and furan], both rotamers), [6.31–6.29 (2H, m), 6.27 (1H, dd, *J* = 3.3, 0.8 Hz), 6.06 (1H, dd, *J* = 3.3, 0.8 Hz), all furan Ar**H**, both rotamers], 4.57 (2H, s, N**CH₂**Ar), 4.53 (2H, d, *J* = 6.7 Hz, CHC**H₂**CO_2N, major), 4.51–4.48 (4H, m, N**CH₂**Ar and CHC**H₂**CO_2N), 4.31 (1H, t, *J* = 6.7 Hz, C**H**CH_2CO_2N, major), 4.21 (1H, t, *J* = 6.3 Hz, C**H**CH_2CO_2N, minor), 4.11 (2H, s, C**H₂**COOH, major), 3.93 (2H, s, C**H₂**COOH, minor); δ_C (100 MHz, $CDCl_3$) 175.00 (**C**OOH), 174.97 (**C**OOH), 156.3 (N**C**OOCH_2CH), 156.0 (N**C**OOCH_2CH), 150.1 (Ar**C**), 150.0 (Ar**C**), 143.9 (2C, Ar**C**), 143.8 (2C, Ar**C**), 142.90 (Ar**C**H), 142.85 (Ar**C**H), 141.5 (2C, Ar**C**), 141.4 (2C, Ar**C**), 127.9 (2C, Ar**C**H), 127.8 (2C, Ar**C**H), 127.3 (2C, Ar**C**H), 127.19 (2C, Ar**C**H), 125.2 (2C, Ar**C**H), 124.9 (2C, Ar**C**H), 120.13 (2C, Ar**C**H), 120.09 (2C, Ar**C**H), 110.6 (2C, Ar**C**H), 109.4 (Ar**C**H), 108.9 (Ar**C**H), 68.3 (NCOO**C**H_2), 68.1 (NCOO**C**H_2), 47.3 (N**C**H_2COOH), 47.2 (2C, **CH**CH_2CO_2N and N**C**H_2COOH), 44.3 (N**C**H_2Ar), 44.2 (N**C**H_2Ar); HRMS (ESI): calcd. for $C_{22}H_{19}NNaO_5$, 400.1155. Found: $[MNa]^+$, 400.1154 (0.4 ppm error).

N-(((9H-Fluoren-9-yl)methoxy)carbonyl)-N-(thiophen-2-ylmethyl)glycine (S32)

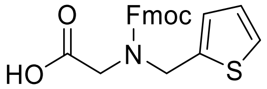

A solution of ethyl bromoacetate (10.0 mmol, 1.11 mL) in dry THF (5 mL) was added dropwise to a solution of 2-thiophenemethylamine (22.0 mmol, 2.26 mL) in dry THF (5 mL) at 0 °C. The mixture was allowed to warm to RT and stirred for 4 h (following by TLC). The solvent was then removed *in vacuo* and the mixture resuspended in diethyl ether (20 mL) at which point a white precipitate began to form. The mixture was filtered and the residue washed with diethyl ether (3 × 10 mL), the filtrate was then concentrated *in vacuo* to afford the amino ester as a colourless oil. NaOH (4N aq., 2.5 mL) was added to a solution of amino acetate in 1,4-dioxane (35 mL) and methanol (13 mL). The reaction was stirred at RT for 2.5 h and analysed by TLC. The solvent was removed *in vacuo* to yield crude the *N*-alkyl amino acid as the sodium salt. The *N*-Fmoc-*N*-alkyl amino acid salt was dissolved in a mixture of deionized water (30 mL), 1,4-dioxane (47 mL), and 10% aq. Na_2CO_3 (52 ml). The solution was cooled to 0 °C whilst stirring and a pre-made solution of 9-fluorenylmethyl chloroformate (3.10 g, 12.0 mmol) dissolved in 1,4-dioxane (10 mL) added slowly. The solution was allowed to warm to RT and stirred for 20 h, analysed by TLC. The mixture was diluted with water (50 mL) and acidified to pH 2 using 10% aq. HCl and extracted with EtOAc (3 × 50 mL). The combined organics were washed with brine, dried over $MgSO_4$ and concentrated in vacuo. Column chromatography (SiO_2, 1:4 ethyl acetate: hexane → 1:1 ethyl acetate: hexane) afforded the *title compound* (as an 1.1:1 mixture of rotamers) as a yellow oil which crystalizes under vacuum, (2.79 g, 71% over 3 steps); R_f 0.23 (9:1 ethyl acetate: methanol); ν_{max}/cm^{-1} (neat) 3067, 1702, 1450, 1221, 906, 726; δ_H (400 MHz, $CDCl_3$) 9.14 (2H, br s, COO**H**, both rotamers) 7.76 (2H, d, *J* = 7.5 Hz, Ar**H**, major), 7.73 (2H, d, *J* = 7.5 Hz, Ar**H**, minor), 7.58 (2H, d, *J* = 7.4 Hz, Ar**H**, major), 7.54 (2H, d, *J* = 7.5 Hz, Ar**H**, minor), 7.43 – 7.34 (4H, m, Ar**H**, both), 7.32–7.26 (4H, m, Ar**H**, both), [7.26–7.23 (2H, m), 6.94–6.92 (3H, m,), 6.81–6.78 (1H, m) all thiophene Ar**H**, both rotamers], 4.74 (2H, s, N**CH₂**Ar, minor), 4.67 (2H, s, N**CH₂**Ar, major), 4.56 (2H, d, *J* = 6.6 Hz, CHC**H₂**CO₂N, major), 4.52 (2H, d, *J* = 6.4 Hz, CHC**H₂**CO₂N, minor) 4.31 (1H, t, *J* = 6.6 Hz, C**H**CH₂CO₂N, major), 4.23 (1H, t, *J* = 6.3 Hz, C**H**CH₂CO₂N, minor), 4.07 (2H, s, C**H₂**COOH, major), 3.89 (2H, s, C**H₂**COOH, minor); δ_C (100 MHz, $CDCl_3$) 175.1 (**C**OOH), 175.0 (**C**OOH), 156.3 (N**C**OOCH₂CH), 156.0 (N**C**OOCH₂CH), 143.8 (Ar**C**), 141.5 (Ar**C**), 141.4 (Ar**C**), 139.0 (Ar**C**), 139.0 (Ar**C**), 127.9 (Ar**C**H), 127.8 (Ar**C**H), 127.4 (Ar**C**H), 127.3 (Ar**C**H), 127.2 (Ar**C**H), 127.1 (Ar**C**H), 127.0 (Ar**C**H), 126.9 (Ar**C**H), 126.2 (Ar**C**H), 126.0

(Ar**C**H), 125.1 (Ar**C**H), 125.0 (Ar**C**H), 120.2 (Ar**C**H), 120.1 (Ar**C**H), 68.3 (NCOO**C**H_2), 68.1 (NCOO**C**H_2), 47.4 (N**C**H_2Ar), 47.3 (**C**HCH_2CO_2N), 47.2 (**C**HCH_2CO_2N), 46.8 (N**C**H_2Ar), 46.2 (N**C**H_2COOH), 46.1 (N**C**H_2COOH); HRMS (ESI): calcd. for $C_{22}H_{19}NNaO_4S$, 416.0927. Found: $[MNa]^+$, 416.0924 (0.7 ppm error).

_N_-(((9_H_-fluoren-9-yl)methoxy)carbonyl)-_N_-(benzo[d][1,3]dioxol-5-ylmethyl)glycine (S33)

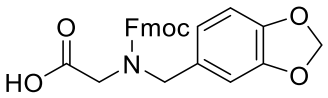

A solution of ethyl bromoacetate (10.0 mmol, 1.11 mL) in dry DCM (5 mL) was added dropwise to a solution of piperonyl amine (17 mmol, 2.15 mL) in dry DCM (10 mL) at 0 °C. The mixture was allowed to warm to RT and stirred for 0.5 h (following by TLC). After 10 min the reaction mixture formed a thick white paste which made stirring difficult. The solvent was then removed *in vacuo* and the mixture resuspended in diethyl ether (20 mL). The mixture was filtered and the residue washed with diethyl ether (3 × 5 mL), the filtrate was then concentrated *in vacuo* to afford the crude amino ester. NaOH (4N aq., 2.5 mL) was added to a solution of amino acetate in 1,4-dioxane (35 mL) and methanol (12.5 mL). The reaction was stirred at RT for 2.5 h and analysed by TLC. The solvent was removed in vacuo to yield crude the *N*-alkyl amino acid as the sodium salt. The *N*-alkyl amino acid salt was dissolved in a mixture of deionized water (30 mL), 1,4-dioxane (50 mL), and Na_2CO_3 (50 mL, 10% solution). The solution was cooled to 0 °C whilst stirring and a pre-made solution of 9-fluorenylmethyl chloroformate (3.2 g, 12.4 mmol) dissolved in 1,4-dioxane (10 mL) added slowly. The solution was allowed to warm to RT and stirred for 21 h, analysed by TLC. The mixture was diluted with water (30 mL) and acidified to pH 1 using 10% aq. HCl and extracted with EtOAc (3 × 50 mL). The combined organics were washed with brine (50 mL), dried over $MgSO_4$ and concentrated in vacuo. Column chromatography (SiO_2, 1:4 ethyl acetate: hexane → 1:1 ethyl acetate: hexane) afforded the *title compound* (as a 1.08:1 mixture of rotamers) as a beige solid, (3.25 g, 75% over 3 steps); m.p. 110–114 °C; R_f 0.17 (9:1 ethyl acetate: methanol) (streaky concentration spot with concentration dependence); ν_{max}/cm^{-1} (neat) 2895, 1700, 1489, 1444, 1232, 1127, 1038, 908, 727; δ_H (400 MHz, $CDCl_3$) 9.91 (2H, br s, COO**H**, both rotamers), 7.73 (4H, t, J = 7.4 Hz, Ar**H**), 7.56–7.51 (4H, m, Ar**H**), 7.41–7.33 (4H, m, Ar**H**), 7.28 (4H, tdd, J = 7.4, 2.8, 1.2 Hz, Ar**H**), 6.74–6.69 (3H, m, Ar**H**), 6.65–6.61 (1H, m, Ar**H**), 6.54–6.49 (2H, m,

Ar**H**), 5.94 (2H, s, OC**H**$_2$O, major rotamer), 5.92 (2H, s, OC**H**$_2$O, minor rotamer), 4.57 (2H, d, *J* = 6.3 Hz, CHC**H**$_2$CO$_2$N, major rotamer), 4.52 (2H, d, *J* = 6.2 Hz, CHC**H**$_2$CO$_2$N, minor rotamer), 4.46 (2H, s, NC**H**$_2$Ar, minor rotamer), 4.36 (2H, s, NC**H**$_2$Ar, major rotamer), 4.27 (1H, t, *J* = 6.3 Hz, C**H**CH$_2$CO$_2$N, major rotamer), 4.21 (1H, t, *J* = 6.2 Hz, C**H**CH$_2$CO$_2$N, minor rotamer), 3.96 (2H, s, NC**H**$_2$COOH, major rotamer), 3.76 (2H, s, NC**H**$_2$COOH, minor rotamer); δ_C (100 MHz, CDCl$_3$) 174.83 (**C**OOH), 174.76 (**C**OOH), 156.6 (N**C**OOCH$_2$CH), 156.4 (N**C**OOCH$_2$CH), 148.2 (2C, Ar**C**), 147.4 (Ar**C**), 147.3 (Ar**C**), 143.84 (2C, Ar**C**), 143.82 (2C, Ar**C**), 141.45 (2C, Ar**C**), 141.43 (2C, Ar**C**), 130.3 (Ar**C**), 130.2 (Ar**C**), 127.9 (2C, Ar**C**H), 127.8 (2C, Ar**C**H), 127.24 (2C, Ar**C**H), 127.19 (2C, Ar**C**H), 125.0 (2C, Ar**C**H), 124.9 (2C, Ar**C**H), 121.8 (Ar**C**H), 121.3 (Ar**C**H), 120.14 (2C, Ar**C**H), 120.08 (2C, Ar**C**H), 108.8 (Ar**C**H), 108.4 (Ar**C**H), 108.30 (Ar**C**H), 108.25 (Ar**C**H), 101.3 (O**C**H$_2$O), 101.2 (O**C**H$_2$O), 68.0 (N**C**H$_2$COOH), 67.9 (N**C**H$_2$COOH), 51.3 (**C**H$_2$), 51.0 (**C**H$_2$), 47.5 (**C**H$_2$), 47.4 (C**H**CH$_2$CO$_2$N), 47.3 (C**H**CH$_2$CO$_2$N), 46.7 (**C**H$_2$); HRMS (ESI): calcd. for $C_{25}H_{21}NNaO_6$, 454.1261. Found: $[MNa]^+$, 454.1263 (–0.4 ppm error).

_N_-(2-(1_H_-inden-2-yl)ethyl)-_N_-(((9_H_-fluoren-9-yl)methoxy)carbonyl)glycine (S34)

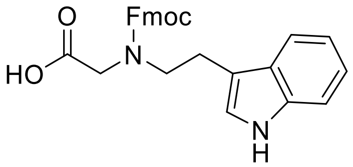

A solution of ethyl bromoacetate (1.11 mL, 10.0 mmol) in dry DCM (5 mL) was added dropwise to a solution of tryptamine (3.53 g, 22 mmol) in dry DCM (10 mL) at 0 °C. The mixture was allowed to warm to RT and stirred for 1.5 h (following by TLC). The solvent was then removed *in vacuo* and the mixture resuspended in diethyl ether (20 mL). The mixture was filtered and the residue washed with diethyl ether (4 × 5 mL), the filtrate was then concentrated *in vacuo* to afford the crude amino ester as a dark orange oil. NaOH (4N aq., 2.5 mL) was added to a solution of amino acetate in 1,4-dioxane (35 mL) and methanol (12 ml). The reaction was stirred at RT for 1.5 h and analysed by TLC. The solvent was removed in vacuo to yield crude the *N*-alkyl amino acid as the sodium salt. The *N*-Fmoc-*N*-alkyl amino acid salt was dissolved in a mixture of deionized water (30 mL), 1,4-dioxane (45 mL), and Na_2CO_3 (52 mL, 10% solution). The solution was cooled to 0 °C whilst stirring and a pre-made solution of 9-fluorenylmethyl chloroformate (3.20 g, 12.4 mmol) dissolved in 1,4-dioxane (10 mL) added

slowly. The solution was allowed to warm to RT and stirred for 18 h, analysed by TLC. The mixture was diluted with water (30 mL) and acidified to pH 1 using 10% aq. HCl and extracted with EtOAc (4 × 50 mL). The combined organics were washed with brine (50 mL), dried over $MgSO_4$ and concentrated in vacuo. Column chromatography (SiO_2, 1:4 ethyl acetate: hexane → ethyl acetate) afforded the *title compound* (as a 3:2 mixture of rotamers) as a light brown crystalline solid, (1.57 g, 36% over 3 steps); m.p. 146–148 °C; R_f 0.17 (9:1 ethyl acetate: methanol) (streaky spot with concentration dependence); ν_{max}/cm^{-1} (neat) 3356, 2926, 1686, 1450, 1248, 1119, 908, 738; δ_H (400 MHz, $CDCl_3$) 8.61 (2H, br s, COO**H**, both rotamers) 8.14–8.11 (2H, m, N**H**, both rotamers), 7.72 (2H, d, *J* = 7.5 Hz, Ar**H**, major rotamer), 7.71 (2H, d, *J* = 7.3 Hz, Ar**H**, minor rotamer), 7.61–7.45 (6H, m, Ar**H**), 7.39–7.23 (10H, m, Ar**H**), 7.19–7.14 (2H, m, Ar**H**), 7.11–7.04 (2H, m, Ar**H**), 6.92 (1H, d, *J* = 2.1 Hz, Ar**H**, minor rotamer), 6.80 (1H, d, *J* = 2.1 Hz, Ar**H**, major rotamer), 4.52–4.44 (4H, m, $CHC\mathbf{H_2}CO_2N$, both rotamers), 4.20–4.13 (2H, m, $C\mathbf{H}CH_2CO_2N$, both rotamers), 3.88 (2H, s, $C\mathbf{H_2}COOH$, major rotamer), 3.77 (2H, s, $C\mathbf{H_2}COOH$, minor rotamer), 3.63 (2H, t, *J* = 7.4 Hz, $N\mathbf{CH_2}CH_2Ar$, minor rotamer), 3.55 (2H, t, *J* = 7.1 Hz, $N\mathbf{CH_2}CH_2Ar$, major rotamer), 3.00 (2H, t, *J* = 7.4 Hz, $NCH_2\mathbf{CH_2}Ar$, minor rotamer), 2.82 (2H, t, *J* = 7.1 Hz, $NCH_2\mathbf{CH_2}Ar$, major rotamer); δ_C (100 MHz, $CDCl_3$) 174.9 (**C**OOH), 174.7 (**C**OOH), 156.9 (N**C**$OOCH_2CH$), 156.0 (N**C**$OOCH_2CH$), 143.93 (Ar**C**), 143.92 (Ar**C**), 141.5 (Ar**C**), 141.4 (Ar**C**), 136.4 (Ar**C**), 127.8 (Ar**C**H), 127.7 (Ar**C**H), 127.4 (Ar**C**), 127.2 (Ar**C**H), 127.1 (Ar**C**H), 124.99 (Ar**C**H), 124.96 (Ar**C**H), 122.3 (Ar**C**H), 122.22 (Ar**C**H), 122.20 (Ar**C**H), 122.1 (Ar**C**H), 120.11 (Ar**C**H), 120.06 (Ar**C**H), 119.6 (Ar**C**H), 119.5 (Ar**C**H), 118.7 (Ar**C**H), 118.6 (Ar**C**H), 112.7 (Ar**C**H), 112.5 (Ar**C**H), 111.4 (Ar**C**H), 111.3 (Ar**C**H), 67.7 (N**C**H_2COOH), 67.1 (N**C**H_2COOH), 49.9 (**C**H_2), 49.8 (**C**H_2), 49.3 (**C**H_2), 49.2 (**C**H_2), 47.3 (C**H**CH_2CO_2N), 24.5 (**C**H_2), 24.1 (**C**H_2); HRMS (ESI): calcd. for $C_{27}H_{25}N_2O_4$, 441.1809. Found: $[MH]^+$, 441.1816 (1.6 ppm error).

4-Methyl-1,4-diazacycloundecane-2,5-dione (20_{RE})

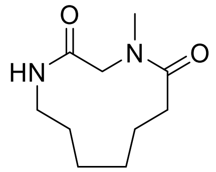

A mixture of 1-aza-2-cyclooctanone (64.4 mg, 0.505 mmol), DMAP (10.2 mg, 0.082 mmol) and pyridine (0.240 mL, 3.00 mmol) in DCM (5 mL) under an argon atmosphere was stirred

at RT for 30 mins. Next, a solution of acid chloride **15** (0.758 mmol, 1.50 equiv., prepared using the general procedure) in DCM (5 mL) was added and the resulting mixture was refluxed at 50 °C for 19 h. The mixture was then diluted with DCM (30 mL) and washed with 10% aq. HCl (15 mL). The aqueous layer was then extracted with DCM (3 × 15 mL) and the combined organic extracts dried over $MgSO_4$ and concentrated *in vacuo*. The crude material was then re-dissolved in DCM (5 mL) and DBU (0.75 mL, 5.00 mmol) was added, followed by stirring at RT for 18 h, before the solvent was removed *in vacuo*. Purification by flash column chromatography (SiO_2, 1:9 ethyl acetate: hexane → 1:1 ethyl acetate: hexane → ethyl acetate → 19:1 ethyl acetate: methanol) afforded the *title compound* (as a 3:1 ratio of rotamers) as a brown yellow solid (81.6 mg, 82%); m.p. 118–121 °C; R_f 0.18 (9:1 ethyl acetate: methanol); ν_{max}/cm^{-1} (neat) 3333, 2931, 1640, 1530, 1464, 1394, 1262, 1094, 799, 728, 647, 584); δ_H (400 MHz, $CDCl_3$, 55 °C) 6.06 (1H, br s, N**H**, major rotamer), 3.92 (2H, s, NC**H$_2$**CO, major rotamer), 3.49–3.33 (2H, m, C**H$_2$**NH, major rotamer), 3.04 (3H, s, C**H$_3$**, major rotamer), 2.27 (2H, m, C**H$_2$**CON, major rotamer), 1.92–1.18 (16H, m, 4 × C**H$_2$**, both rotamers); Diagnostic ^{1}H NMR resonance of minor rotamer at reported at room temperature: δ_H (400 MHz, $CDCl_3$) 6.53 (1H, br d, *J* = 9.8 Hz, N**H**, minor rotamer), 4.93 (1H, d, *J* = 12.9 Hz, C**H**H′NMe, minor rotamer), 3.11 (3H, s, C**H$_3$**, minor rotamer), 2.90 (1H, d, *J* = 12.9 Hz, CH**H′**NMe, minor rotamer); δ_C (100 MHz, $CDCl_3$) data for major rotamer, 175.2 (**C**O), 169.7 (**C**O), 55.2 (N**C**H$_2$CO), 39.40 (**C**H$_2$NH), 36.7 (N**C**H$_3$), 30.4 (**C**H$_2$CON), 28.7 (**C**H$_2$), 25.7 (**C**H$_2$), 24.0 (**C**H$_2$), 22.7 (**C**H$_2$); data for minor rotamer, 176.7 (**C**O), 169.9 (**C**O), 53.9 (N**C**H$_2$CO), 39.42 (**C**H$_2$NH), 37.7 (N**C**H$_3$), 33.1 (**C**H$_2$CON), 27.0 (**C**H$_2$), 26.9 (**C**H$_2$), 23.6 (**C**H$_2$), 22.8 (**C**H$_2$); HRMS (ESI): calcd. for $C_{10}H_{18}N_2NaO_2$, 221.1260. Found: $[MNa]^+$, 221.1260 (0.3 ppm error).

4-Methyl-1,4-diazacyclododecane-2,5-dione (21$_{RE}$)

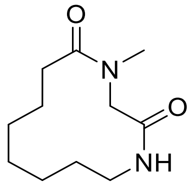

A mixture of azonan-2-one (100 mg, 0.710 mmol), DMAP (8.67 mg, 0.0710 mmol), pyridine (0.340 mL, 4.26 mmol, 6 eq.) in DCM (5.0 mL) under an argon atmosphere was stirred at RT for 30 mins. Next, a solution of acid chloride **15** (1.07 mmol, 1.50 equiv., prepared using the general procedure) in DCM (3.2 mL) was added and the resulting mixture was heated, at reflux,

at 50 °C for 16 h. The mixture was then diluted with DCM (25 mL) and washed with 10% aq. HCl (25 mL). The aqueous layer was then extracted with DCM (3 × 20 mL) and the combined organic extracts dried over $MgSO_4$ and concentrated *in vacuo*. The crude mixture was then re-dissolved in DCM (7.23 mL) and DBU (1.08 mL, 7.24 mmol) was added, followed by stirring at RT overnight, before the solvent was removed *in vacuo*. Purification by flash column chromatography (SiO_2, 6:4 ethyl acetate: hexane → 95:5 ethyl acetate: methanol) afforded the *title compound* (as a mixture of two rotamers in an approximately 1.3:1 ratio) as a white solid (133 mg, 94%); m.p. 125−130 °C; R_f 0.33 (95:5 ethyl acetate: methanol); ν_{max}/cm^{-1} (thin film) 3343, 2979, 2941, 2919, 2899, 2861, 2847, 1632, 1533, 1474, 1448, 1435; δ_H (400 MHz, $CDCl_3$) 6.75 (1H, s, NH, minor), 6.09 (1H, s, NH, major), 4.87–4.83 (1H, m, C**H**HNCH$_3$, minor), 3.89 (2H, s, C**H$_2$**NCH$_3$, major), 3.50–3.46 (1H, m, C**H**HNH, minor), 3.37–3.26 (2H, m, C**H$_2$**NH, major), 3.09 (3H, s, CH$_3$, minor), 2.99 (3H, s, CH$_3$, major), 2.89–2.85 (1H, m, CH**H**NCH$_3$, minor), 2.70–2.68 (1H, m, CH**H**NH, minor), 2.58–2.51 (1H, m, C**H**HCO, minor), 2.20–2.17 (2H, m, CH$_2$CO, major), 2.09–2.04 (1H, m, CH**H**CO, minor), 1.84–1.75 (1H, m, C**H**HCH$_2$CO, minor) 1.72–1.64 (2H, m, C**H$_2$**CH$_2$CO, major), 1.51–1.50 (2H, m, C**H$_2$**CH$_2$NH, major), 1.44–0.97 (15H , m, 7 × CH$_2$ and CH**H**CH$_2$CO, minor); δ_c (100 MHz, $CDCl_3$) 175.1 (**C**ONCH$_3$, minor), 174.6 (**C**ONCH$_3$, major), 169.3 (CONH, minor), 168.3 (CONH, major), 54.6 (**C**H$_2$NCH$_3$, major), 53.4 (**C**H$_2$NCH$_3$, minor), 39.9 (CH$_2$NH, major), 38.9 (CH$_2$NH, minor), 37.7 (CH$_3$, minor), 36.3 (CH$_3$, major), 32.8 (**C**H$_2$CO, minor), 29.1 (**C**H$_2$CO, major), 27.5 (CH$_2$), 27.1 (CH$_2$), 26.6 (CH$_2$), 24.7 (CH$_2$), 24.6 (CH$_2$), 24.5 (CH$_2$), 24.4 (CH$_2$), 24.3 (CH$_2$), 23.5 (CH$_2$), 23.3 (CH$_2$). HRMS (ESI): calcd. for $C_{11}H_{20}N_2O_2$, 235.1417. Found; $[MNa]^+$, 235.1413 (1.8 ppm error).

2,5-Methyl-1,4-diazacyclotridecane-2,5-dione (22$_{RE}$)

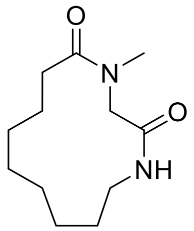

A mixture of octahydro-azecin-2-one (100 mg, 0.640 mmol), DMAP (7.80 mg, 0.064 mmol), pyridine (0.310 mL, 3.84 mmol) in DCM (4.5 mL) under an argon atmosphere was stirred at RT for 30 mins. Next, a solution of acid chloride **15** (0.960 mmol, 1.50 equiv., prepared using

the general procedure) in DCM (2.9 mL) was added and the resulting mixture was heated, at reflux, at 50 °C for 16 h. The mixture was then diluted with DCM (25 mL) and washed with 10% aq. HCl (25 mL). The aqueous layer was then extracted with DCM (3 × 20 mL) and the combined organic extracts dried over $MgSO_4$ and concentrated *in vacuo*. The crude mixture was then re-dissolved in DCM (6.50 mL) and DBU (0.98 mL, 6.57 mmol) was added, followed by stirring at RT overnight, before the solvent was removed *in vacuo*. Purification by flash column chromatography (SiO_2, 6:4 ethyl acetate: hexane → ethyl acetate) afforded the *title compound* (as a mixture of two rotamers in an approximately 3.6:1 ratio) as a white solid (124.5 mg, 86%); m.p. 103−105 °C; R_f 0.38 (95:5 ethyl acetate: methanol); ν_{max}/cm^{-1} (thin film) 3345, 2921, 2849, 1639, 1525, 1479, 1463, 1451, 1437; δ_H (400 MHz, $CDCl_3$) 6.76 (1H, br s, NH, major), 6.41 (1H, br s, NH, minor), 4.91 (1H, d, *J* = 13.7 Hz, C**H**HNCH$_3$, major), 3.93 (2H, s, minor, C**H**$_2$NCH$_3$), 3.95–3.62 (1H, m, C**H**HNH, major), 3.31–3.24 (2H, m, CH$_2$NH, minor), 3.16 (3H, s, NCH$_3$, major), 3.04 (3H, s, NCH$_3$, minor), 2.98 (1H, d, *J* = 13.7 Hz, CH**H**NCH$_3$, major), 2.77–2.71 (1H, m, CH**H**NH, minor), 2.66–2.59 (1H, m, C**H**HCO, major), 2.24 (2H, m, CH$_2$CO, minor), 2.18–2.12 (1H, m, CH**H**CO, major), 1.83–1.22 (24 H, m, 12 × CH$_2$, both); δ_c (100 MHz, $CDCl_3$) 174.8 (CONCH$_3$, major), 173.8 (CONCH$_3$, minor), 168.9 (CONH, major), 168.4 (CONH, minor), 54.1 (**C**H$_2$NCH$_3$, minor), 53.3 (**C**H$_2$NCH$_3$, major), 39.0 (CH$_2$NH, minor), 38.9 (CH$_2$NH, major), 37.5 (NCH$_3$, major), 35.9 (NCH$_3$, minor), 32.3 (major, **C**H$_2$CO), 30.2 (minor, **C**H$_2$CO), 26.6 (CH$_2$, minor), 26.3 (CH$_2$, major), 26.2 (CH$_2$, minor), 25.3 (CH$_2$, major), 25.2 (CH$_2$, major), 25.1 (CH$_2$, major), 25.0 (CH$_2$, minor), 23.9 (CH$_2$, major), 23.6 (CH$_2$, major), 23.5 (CH$_2$, minor), 23.1 (CH$_2$, minor), 22.9 (CH$_2$, minor). HRMS (ESI): calcd. for $C_{12}H_{22}N_2O_2$, 249.1573. Found; $[MNa]^+$, 249.1568 (2.2 ppm error).

4-Methyl-1,4-diazacyclotetradecane-2,5-dione (23$_{RE}$)

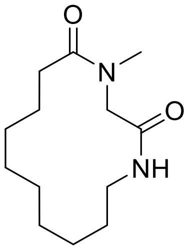

A mixture of azacycloundecan-2-one (100 mg, 0.590 mmol), DMAP (7.21 mg, 0.0590 mmol), pyridine (0.280 mL, 3.54 mmol) in DCM (4.1 mL) under an argon atmosphere was stirred at RT for 30 mins. Next, a solution of acid chloride **15** (0.885 mmol, 1.50 equiv., prepared using

the general procedure) in DCM (2.7 mL) was added and the resulting mixture was heated, at reflux, at 50 °C for 16 h. An additional solution of acid chloride **15** (0.885 mmol, 1.50 equiv. prepared using the general procedure) in DCM (2.7 mL) was added and the reaction heated, at reflux at 50 °C for another 12 h to achieve reaction completion. The mixture was then diluted with DCM (25 mL) and washed with 10% aq. HCl (25 mL). The aqueous layer was then extracted with DCM (3 × 20 mL) and the combined organic extracts dried over $MgSO_4$ and concentrated *in vacuo*. The crude mixture was then re-dissolved in DCM (6 mL) and DBU (0.90 mL, 6.03 mmol) was added, followed by stirring at RT overnight, before the solvent was removed *in vacuo*. Purification by flash column chromatography (SiO_2, 6:4 ethyl acetate: hexane → ethyl acetate) afforded the *title compound* (as a mixture of two rotamers in an approximately 4.3:1 ratio) as a white solid (93.6 mg, 66%); m.p. 125−130 °C; R_f 0.47 (95:5 ethyl acetate: methanol); ν_{max}/cm^{-1} (thin film) 3386, 3261, 3088, 3926, 2861, 2847, 1655, 1634, 1564, 1522, 1484, 1458, 1438, 1424; δ_H (400 MHz, $CDCl_3$) 6.58 (1H, br s, NH, major), 6.32 (1H, br s, NH, minor), 3.93–3.90 (4H, m, C**H**$_2$NCH$_3$, both), 3.31–3.28 (2H, m, C**H**$_2$NH, minor), 3.22–3.16 (2H, m, C**H**$_2$NH, major), 3.12 (3H, s, NCH$_3$, major), 2.99 (3H, s, NCH$_3$, minor), 2.39 (2H, t, J = 5.9 Hz, CH$_2$CO, major), 2.19 (2H, t, J = 7.1 Hz, CH$_2$CO, minor), 1.71–1.63 (4H, m, C**H**$_2$CH$_2$CO, both), 1.49–1.42 (4H, m, **C**H$_2$CH$_2$NH, both), 1.83–1.22 (20 H, m, 10 × CH$_2$, both); δ_C (100 MHz, $CDCl_3$) 174.1 (CONCH$_3$, major), 173.7 (CONCH$_3$, minor), 169.2 (CONH, major), 168.2 (CONH, minor), 53.7 (**C**H$_2$NCH$_3$, minor), 53.3 (**C**H$_2$NCH$_3$, major), 38.7 (CH$_2$NH, minor), 37.7 (CH$_2$NH, major), 37.3 (NCH$_3$, major), 35.6 (NCH$_3$, minor), 31.0 (major, **C**H$_2$CO), 30.7 (minor, **C**H$_2$CO), 27.5 (**C**H$_2$CH$_2$NH, minor), 27.2 (**C**H$_2$CH$_2$NH, major), 25.9 (CH$_2$, minor), 25.7 (CH$_2$, minor), 25.4 (CH$_2$, major), 25.3 (CH$_2$, major), 25.2 (CH$_2$, major), 25.1 (CH$_2$, major), 24.6 (**C**H$_2$CH$_2$CO, minor), 24.2 (**C**H$_2$CH$_2$CO, major), 23.9 (CH$_2$, minor), 23.1 (CH$_2$, minor), 22.9 (CH$_2$, major), 22.2 (CH$_2$, minor). HRMS (ESI): calcd. for $C_{13}H_{24}N_2O_2$, 263.1730. Found; $[MNa]^+$, 263.1728 (0.8 ppm error).

4-Methyl-1,4-diazacyclopentadecane-2,5-dione (24$_{RE}$)

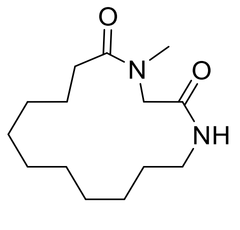

A mixture of azacyclododecan-2-one (100 mg, 0.550 mmol), DMAP (6.72 mg, 0.0550 mmol), pyridine (0.260 mL, 3.30 mmol) in DCM (3.8 mL) under an argon atmosphere was stirred at RT for 30 mins. Next, a solution of acid chloride **15** (0.825 mmol, 1.50 equiv., prepared using the general procedure) in DCM (2.5 mL) was added and the resulting mixture was heated, at reflux, at 50 °C for 16 h. The mixture was then diluted with DCM (25 mL) and washed with 10% aq. HCl (25 mL). The aqueous layer was then extracted with DCM (3 × 20 mL) and the combined organic extracts dried over $MgSO_4$ and concentrated *in vacuo*. The crude mixture was then re-dissolved in DCM (5.6 mL) and DBU (0.84 mL, 5.63 mmol) was added, followed by stirring at RT overnight, before the solvent was removed *in vacuo*. Purification by flash column chromatography (SiO_2, 6:4 ethyl acetate: hexane → ethyl acetate) afforded the *title compound* (as a mixture of three rotamers in an approximately 3.0:1.9:1.4 ratio) as a white solid (108.8 mg, 78%); m.p. 141−145 °C; R_f 0.50 (95:5 ethyl acetate: methanol); ν_{max}/cm^{-1} (thin film) 3295, 3091, 2926, 2848, 1657, 1628, 1548,1484, 1454, 1440; δ_H (400 MHz, $CDCl_3$) 6.59 (2H, br m, 2 × NH), 6.38 (1H, s, NH), 3.92–3.89 (6H, m, 3 × C**H₂**NCH₃), 3.29–3.25 (2H, m, C**H₂**NH), 3.21–3.17 (4H, m, 2 × C**H₂**NH), 3.11 (3H, s, CH_3), 3.09 (3H, s, CH_3), 2.95 (3H, s, CH_3), 2.39–2.32 (4H, m, 2 × CH_2CO), 2.19–2.15 (2H, m, CH_2CO), 1.72–1.48 (6H, m, 3 × C**H₂**CH₂CO), 1.48–1.13 (42 H, m, 21 × CH_2); δ_C (100 MHz, $CDCl_3$) 174.5 (**C**ONCH₃), 174.3 (**C**ONCH₃), 173.8 (**C**ONCH₃), 169.5 (CONH), 169.4 (CONH), 168.3 (CONH), 53.8 (**C**H₂NCH₃), 53.7 (**C**H₂NCH₃), 53.6 (**C**H₂NCH₃), 39.0 (CH_2NH), 38.7 (CH_2NH), 37.9 (CH_2NH), 37.5 (NCH_3), 37.3 (NCH_3), 35.6 (NCH_3), 31.9 (**C**H₂CO), 31.3 (**C**H₂CO), 30.8 (**C**H₂CO), 28.5 (**C**H₂CH₂NH), 28.4 (**C**H₂CH₂NH), 28.2 (**C**H₂CH₂NH), 27.4 (CH_2) 26.8 (CH_2), 26.6 (CH_2), 26.5 (CH_2), 26.4 (CH_2), 26.3 (CH_2), 26.0 (CH_2), 25.7 (CH_2), 25.6 (CH_2), 25.5 (CH_2), 25.4 (CH_2), 25.3 (CH_2), 25.2 (CH_2), 24.8 (CH_2), 24.5 (CH_2), 24.2 (CH_2), 23.9 (CH_2), 23.6 (CH_2), 23.5 (**C**H₂CH₂CO), 23.1 (**C**H₂CH₂CO), 21.7 (**C**H₂CH₂CO). HRMS (ESI): calcd. for $C_{14}H_{26}N_2O_2$, 277.1886. Found; $[MNa]^+$, 277.1885 (0.5 ppm error).

4-Cyclopropyl-1,4-diazacycloundecane-2,5-dione (27a)

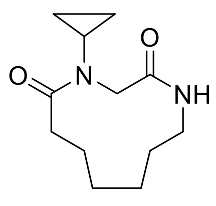

A mixture of 1-aza-2-cyclooctanone (100 mg, 0.786 mmol), DMAP (10 mg, 0.078 mmol) and pyridine (0.380 mL, 4.72 mmol) in DCM (5.5 mL) under an argon atmosphere was stirred at RT for 30 mins. Next, a solution of acid chloride **26a** (1.18 mmol, 1.50 equiv., prepared using the general procedure) in DCM (3 mL) was added and the resulting mixture was refluxed at 50 °C for 24 h. The mixture was then diluted with DCM (30 mL) and washed with 10% aq. HCl (30 mL). The aqueous layer was then extracted with DCM (3 × 30 mL) and the combined organic extracts dried over $MgSO_4$ and concentrated *in vacuo*. The crude material was then re-dissolved in DCM (8 mL) and DBU (1.2 mL, 7.86 mmol) was added, followed by stirring at RT overnight, before the solvent was removed *in vacuo*. Purification by flash column chromatography (SiO_2, 1:4 ethyl acetate: hexane → 1:1 ethyl acetate: hexane) afforded the *title compound* (as a 6:1 mixture of rotamers) as a white solid (151 mg, 85%); m.p. 88–90 °C; R_f 0.30 (1:1 ethyl acetate: hexane); ν_{max}/cm^{-1} (thin film) 3300, 2927, 2854, 1649, 1456; δ_H (400 MHz, $CDCl_3$) 6.46 (1H, br s, N**H**, major), 5.87 (1H, br s, N**H**, minor), 4.82 (2H, d, *J* = 13.0 Hz, NC**H**H'CO, both rotamers), 3.70–3.52 (2H, m, NHC**H**H', both), 3.20 (2H, d, *J* = 13.0 Hz, NCH**H**'CO, both), 2.98–2.84 (2H, m, COC**H**H', both), 2.83–2.70 (4H, m, CH_2C**H**N and NHCH**H**', both), 2.24–2.14 (2H, m, COCH**H**', both), 2.03–1.90 (2H, m, NHCH$_2$C**H**H', both), 1.79–1.63 (2H, m, NHCH$_2$CH**H**', both), 1.60–1.18 (12H, m, 3 × C**H**$_2$, both), 1.15–0.50 (8H, m, 2 × C**H**$_2$, both); δ_C (100 MHz, $CDCl_3$) data for the major rotamer only: 179.6 (**C**O), 170.0 (**C**O), 53.1 (**C**H$_2$), 39.1 (**C**H$_2$), 33.8 (**C**H$_2$), 31.2 (**C**H$_2$), 27.0 (2 × **C**H$_2$), 23.4 (**C**H$_2$), 22.9 (**C**H$_2$), 12.4 (**C**H$_2$), 8.3 (**C**H$_2$); HRMS (ESI): calcd. for $C_{12}H_{20}N_2NaO_2$, 247.1417 Found: $[M+Na]^+$, 247.1416 (0.7 ppm error).

4-Cyclopropyl-1,4-diazacyclohexadecane-2,5-dione (27b)

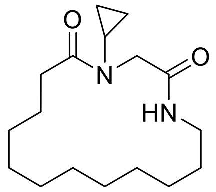

A mixture of laurolactam (155 mg, 0.786 mmol), DMAP (10 mg, 0.078 mmol) and pyridine (0.380 mL, 4.72 mmol) in DCM (5.5 mL) under an argon atmosphere was stirred at RT for 30 mins. Next, a solution of acid chloride **26a** (1.18 mmol, 1.50 equiv., prepared using the general

procedure) in DCM (3 mL) was added and the resulting mixture was refluxed at 50 °C for 24 h. The mixture was then diluted with DCM (30 mL) and washed with 10% aq. HCl (30 mL). The aqueous layer was then extracted with DCM (3 × 30 mL) and the combined organic extracts dried over $MgSO_4$ and concentrated *in vacuo*. The crude material was then re-dissolved in DCM (8 mL) and DBU (1.2 mL, 7.86 mmol) was added, followed by stirring at RT overnight, before the solvent was removed *in vacuo*. Purification by flash column chromatography (SiO_2, 1:2 ethyl acetate: hexane → 1:1 ethyl acetate: hexane) afforded the *title compound* as a white solid (339 mg, 84%); m.p. 102–104 °C; R_f 0.66 (9:1 ethyl acetate: methanol); ν_{max}/cm^{-1} (thin film) 3282, 2925, 2858, 1642, 1564; δ_H (400 MHz, $CDCl_3$) 6.52 (1H, br s, N**H**), 3.93 (2H, s, C**H**$_2$), 3.25–3.13 (2H, m, C**H**$_2$), 2.83–2.73 (1H, m, C**H**), 2.56 (2H, t, J = 6.1 Hz, C**H**$_2$), 1.70–1.55 (2H, m, C**H**$_2$), 1.50–1.10 (16H, m, 8 × C**H**$_2$), 0.85–0.55 (4H, m, 2 × CH_2); δ_C (100 MHz, $CDCl_3$): 177.0 (**C**O), 170.1 (**C**O), 52.8 (N**C**H$_2$CO), 38.8 (NH**C**H$_2$), 32.5 (**C**H$_2$CON), 31.8 (**C**H$_2$), 28.5 (**C**H$_2$), 26.9 (**C**H$_2$), 26.7 (**C**H$_2$), 26.0 (2 × **C**H$_2$), 25.7 (**C**H$_2$), 24.9 (**C**H$_2$), 24.6 (**C**H$_2$), 23.6 (**C**H$_2$), 9.5 (2 × **C**H$_2$). HRMS (ESI): calcd. for $C_{17}H_{31}N_2O_2$: 295.2380 Found: $[M+H]^+$, 295.2378 (0.5 ppm error).

4-Cyclopentyl-1,4-diazacycloundecane-2,5-dione (27c)

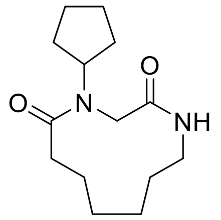

A mixture of 1-aza-2-cyclooctanone (155 mg, 0.786 mmol), DMAP (10 mg, 0.078 mmol) and pyridine (0.380 mL, 4.72 mmol) in DCM (5.50 mL) under an argon atmosphere was stirred at RT for 30 mins. Next, a solution of acid chloride **26b** (1.18 mmol, 1.50 equiv.; prepared using the general procedure) in DCM (3 mL) was added and the resulting mixture was refluxed at 50 °C for 24 h. The mixture was then diluted with DCM (30 mL) and washed with 10% aq. HCl (30 mL). The aqueous layer was then extracted with DCM (3 × 30 mL) and the combined organic extracts dried over $MgSO_4$ and concentrated *in vacuo*. The crude material was then re-dissolved in DCM (8 mL) and DBU (1.2 mL, 7.86 mmol) was added, followed by stirring at RT overnight, before the solvent was removed *in vacuo*. Purification by flash column chromatography (SiO_2, 1:4 ethyl acetate: hexane → 1:1 ethyl acetate: hexane) afforded the *title*

compound as a white solid (170 mg, 86%); m.p. 125–127 °C; R_f 0.30 (1:1 ethyl acetate: hexane); ν_{max}/cm^{-1} (thin film) 3300, 2927, 2854, 1649, 1456; δ_H (400 MHz, $CDCl_3$) N.B. all signals are broad, presumably due to rotamer interconversion on the NMR timescale: 5.93 (1H, br s, N**H**), 5.20–4.93 (1H, m, C**H**), 4.06–3.67 (2H, m, C**H**$_2$), 2.51–2.12 (2H, m, C**H**$_2$), 2.08–0.71 (18H, m, 9 × C**H**$_2$); δ_C (100 MHz, $CDCl_3$): 175.3 (**C**O), 170.2 (**C**O), 54.5 (**C**H), 47.8 (**C**H$_2$), 39.3 (**C**H$_2$), 30.7 (**C**H$_2$), 29.1 (**C**H$_2$), 28.9 (**C**H$_2$), 26.0 (**C**H$_2$), 24.2 (**C**H$_2$), 23.9 (**C**H$_2$), 22.3 (**C**H$_2$); HRMS (ESI): calcd. for $C_{14}H_{25}N_2O_2$, 253.1911 Found: $[M+H]^+$, 253.1915 (−1.5 ppm error).

4-Cyclopentyl-1,4-diazacyclotridecane-2,5-dione (27d)

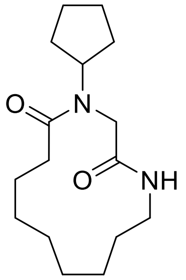

A mixture of octahydro-azecin-2-one (61 mg, 0.394 mmol), DMAP (5 mg, 0.039 mmol) and pyridine (0.19 mL, 2.36 mmol) in DCM (3 mL) under an argon atmosphere was stirred at RT for 30 mins. Next, a solution of acid chloride **26b** (0.590 mmol, 1.50 equiv., prepared using the general procedure) in DCM (2 mL) was added and the resulting mixture was refluxed at 50 °C for 24 h. The mixture was then diluted with DCM (10 mL) and washed with 10% aq. HCl (10 mL). The aqueous layer was then extracted with DCM (3 × 10 mL) and the combined organic extracts dried over $MgSO_4$ and concentrated in vacuo. The crude material was then re-dissolved in DCM (4 mL) and DBU (0.6 mL, 3.94 mmol) was added, followed by stirring at RT overnight, before the solvent was removed in vacuo. Purification by flash column chromatography (SiO_2, 1:4 ethyl acetate:hexane → ethyl acetate) afforded the *title compound* (as a 3:1 mixture of rotamers) as a white solid (98 mg, 89%); m.p. 128–130 °C; R_f 0.30 (ethyl acetate); ν_{max}/cm^{-1} (thin film) 3312, 2931, 2863, 1649, 1534; δ_H (400 MHz, $CDCl_3$) 7.17 (1H, br s, N**H**, major rotamer), 6.36 (1H, br s, N**H**, minor), 5.00–4.86 (2H, m, C**H**$_2$, minor), 4.72–4.54 (1H, m, CH'**H**, major), 4.35–4.16 (1H, m, C**H**'H, major), 3.90–3.73 (1H, m, C**H**, major), 3.70–3.53 (2H, m, C**H**'H, both), 3.38–3.10 (3H, m, C**H**'H, both and C**H**, minor), 2.93–2.62 (4H, m, CH'**H**, both and CH'**H**, both), 2.38–1.10 (40H, m, 10 × C**H**$_2$, both); δ_C (100 MHz,

$CDCl_3$): data for the major rotamer: 175.3 (**C**O), 170.5 (**C**O), 59.8 (**C**H), 46.5 (**C**H_2), 39.1 (**C**H_2), 33.2 (**C**H_2), 28.9 (**C**H_2), 26.2 (**C**H_2), 26.0 (**C**H_2), 25.8 (**C**H_2), 25.7 (**C**H_2), 24.8 (**C**H_2), 24.2 (**C**H_2), 23.6 (**C**H_2), 23.5 (**C**H_2). Diagnostic ^{13}C NMR resonances for the minor rotamer: 174.1 (**C**O), 169.3 (**C**O), 55.2 (**C**H), 47.4 (**C**H_2), 39.2 (**C**H_2), 31.3 (**C**H_2), 29.7 (**C**H_2), 29.6 (**C**H_2), 26.6 (**C**H_2), 26.4 (**C**H_2), 25.0 (**C**H_2), 23.9 (**C**H_2), 23.3 (**C**H_2), 22.8 (**C**H_2); HRMS (ESI): calcd. for $C_{16}H_{28}N_2O_2Na$, 303.2043 Found: $[M+Na]^+$, 303.2044 (−0.6 ppm error).

4-Cyclopentyl-1,4-diazacyclohexadecane-2,5-dione (27e)

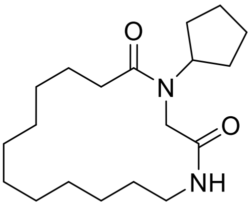

A mixture of laurolactam (155 mg, 0.786 mmol), DMAP (10 mg, 0.078 mmol) and pyridine (0.380 mL, 4.72 mmol) in DCM (5.50 mL) under an argon atmosphere was stirred at RT for 30 mins. Next, a solution of acid chloride **26b** (1.18 mmol, 1.50 equiv., prepared using the general procedure) in DCM (3 mL) was added and the resulting mixture was refluxed at 50 °C for 24 h. The mixture was then diluted with DCM (30 mL) and washed with 10% aq. HCl (30 mL). The aqueous layer was then extracted with DCM (3 × 30 mL) and the combined organic extracts dried over $MgSO_4$ and concentrated *in vacuo*. The crude material was then re-dissolved in DCM (8 mL) and DBU (1.2 mL, 7.86 mmol) was added, followed by stirring at RT overnight, before the solvent was removed *in vacuo*. Purification by flash column chromatography (SiO_2, 1:4 ethyl acetate: hexane → 1:1 ethyl acetate: hexane) afforded the *title compound* (as a 6:1 mixture of rotamers) as a white solid (240 mg, 93%); m.p. 110–112 °C; R_f 0.30 (1:1 ethyl acetate: hexane); ν_{max}/cm^{-1} (thin film) 3300, 2927, 2854, 1649, 1456; δ_H (400 MHz, $CDCl_3$) 6.85 (1H, br s, N**H**, major), 6.12 (1H, br s, N**H**, minor), 4.90–4.75 (1H, m, C**H**, minor), 4.25–4.15 (1H, m, C**H**, major), 3.84 (4H, s, C**H**$_2$, both rotamers), 3.40–3.28 (2H, m, C**H**$_2$, minor), 3.27–3.17 (2H, m, C**H**$_2$, major), 2.43 (2H, t, J = 6.4 Hz, C**H**$_2$, major), 2.17 (2H, t, J = 6.4 Hz, C**H**$_2$, minor), 1.85–1.36 (24H, m, 6 × C**H**$_2$, both rotamers), 1.35–1.10 (28H, m, 7 × C**H**$_2$, both rotamers); δ_C (100 MHz, $CDCl_3$) data for the major rotamer only: 174.7 (**C**O), 170.7 (**C**O), 59.1 (**C**H), 46.9 (**C**H_2), 38.8 (**C**H_2), 32.5 (**C**H_2), 29.6 (**C**H_2), 28.4 (**C**H_2), 26.9 (**C**H_2), 26.7 (**C**H_2), 26.2 (**C**H_2), 25.8 (**C**H_2), 25.7 (**C**H_2), 24.8 (**C**H_2), 24.7 (**C**H_2), 24.5 (**C**H_2), 23.7 (**C**H_2). Diagnostic ^{13}C NMR resonances for the minor rotamer: 174.3 (**C**O), 169.3 (**C**O),

55.3 (**C**H), 47.3 (**C**H_2), 40.0 (**C**H_2), 33.5 (**C**H_2), 29.3 (**C**H_2), 28.6 (**C**H_2), 26.6 (**C**H_2), 26.4 (**C**H_2), 25.4 (**C**H_2), 25.0 (**C**H_2), 23.4 (**C**H_2); HRMS (ESI): calcd. for $C_{19}H_{35}N_2O_2$, 323.2693 Found: $[M+H]^+$, 323.2698 (−2.0 ppm error).

4-Cyclohexyl-1,4-diazacycloundecane-2,5-dione (27f)

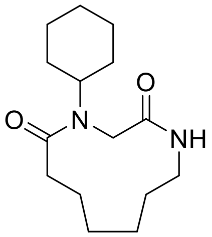

A mixture of 1-aza-2-cyclooctanone (50 mg, 0.393 mmol), DMAP (5 mg, 0.039 mmol) and pyridine (0.190 mL, 1.77 mmol) in DCM (3 mL) under an argon atmosphere was stirred at RT for 30 mins. Next, a solution of acid chloride **26c** (0.59 mmol, 1.50 equiv., prepared using the general procedure) in DCM (1 mL) was added and the resulting mixture was refluxed at 50 °C for 24 h. The mixture was then diluted with DCM (10 mL) and washed with 10% aq. HCl (10 mL). The aqueous layer was then extracted with DCM (3 × 10 mL) and the combined organic extracts dried over $MgSO_4$ and concentrated *in vacuo*. The crude material was then re-dissolved in DCM (4 mL) and DBU (0.60 mL, 3.93 mmol) was added, followed by stirring at RT overnight, before the solvent was removed *in vacuo*. Purification by flash column chromatography (SiO_2, 1:4 ethyl acetate: hexane → 1:1 ethyl acetate: hexane) afforded the *title compound* as a white solid (116 mg, 88%); m.p. 152–154 °C; R_f 0.30 (100% ethyl acetate); ν_{max}/cm^{-1} (thin film) 3333, 2929, 2854, 1630, 1465; δ_H (400 MHz, $CDCl_3$) 5.97 (1H, br s, N**H**), 4.65–4.49 (1H, m, C**H**), 3.88 (2H, s, C**H**$_2$), 2.44–2.11 (2H, m, C**H**$_2$), 1.92–0.94 (20H, m, 10 × C**H**$_2$); δ_C (100 MHz, $CDCl_3$): 174.9 (**C**O), 170.4 (**C**O), 52.6 (**C**H), 47.5 (**C**H_2), 39.3 (**C**H_2), 30.7 (**C**H_2), 30.6 (**C**H_2), 28.9 (**C**H_2), 26.1 (**C**H_2), 25.5 (**C**H_2), 25.2 (**C**H_2), 24.3 (**C**H_2), 22.4 (**C**H_2); HRMS (ESI): calcd. for $C_{15}H_{26}N_2NaO_2$, 289.1886 Found: $[M+H]^+$, 289.1886 (0.2 ppm error).

4-Cyclohexyl-1,4-diazacyclohexadecane-2,5-dione (27g)

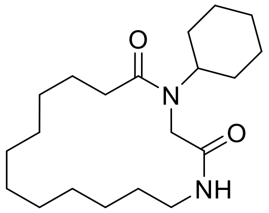

A mixture of laurolactam (155 mg, 0.786 mmol), DMAP (10 mg, 0.078 mmol) and pyridine (0.380 mL, 4.72 mmol) in DCM (5.50 mL) under an argon atmosphere was stirred at RT for 30 mins. Next, a solution of acid chloride **26c** (1.18 mmol, 1.50 equiv., prepared using the general procedure) in DCM (3 mL) was added and the resulting mixture was refluxed at 50 °C for 24 h. The mixture was then diluted with DCM (30 mL) and washed with 10% aq. HCl (30 mL). The aqueous layer was then extracted with DCM (3 × 30 mL) and the combined organic extracts dried over $MgSO_4$ and concentrated *in vacuo*. The crude material was then re-dissolved in DCM (8 mL) and DBU (1.2 mL, 7.86 mmol) was added, followed by stirring at RT overnight, before the solvent was removed *in vacuo*. Purification by flash column chromatography (SiO_2, 1:4 ethyl acetate: hexane → 1:1 ethyl acetate: hexane) afforded the *title compound* (as a 8:1 mixture of rotamers) as a white solid (242 mg, 90%); m.p. 123–125 °C; R_f 0.30 (1:1 ethyl acetate: hexane); ν_{max}/cm^{-1} (thin film) 3300, 2925, 2854, 1664, 1543; δ_H (400 MHz, $CDCl_3$) 6.94 (1H, br s, N**H**, major), 6.10 (1H, br s, N**H**, minor), 3.89 (2H, s, C**H**$_2$, major), 3.85 (2H, s, C**H**$_2$, minor), 3.68–3.50 (2H, m, C**H**, both rotamers), 3.37–3.26 (2H, m, C**H**$_2$, minor), 3.25–3.15 (2H, m, C**H**$_2$, major), 2.40 (2H, t, J = 6.1 Hz, C**H**$_2$, major), 2.17 (2H, t, J = 6.1 Hz, C**H**$_2$, minor), 1.89–1.77 (8H, m, 4 × C**H**$_2$, minor), 1.70–1.48 (8H, m, 4 × C**H**$_2$, major), 1.47–1.00 (40H, m, 10 × C**H**$_2$, both); δ_C (100 MHz, $CDCl_3$) data for the major rotamer only: 174.5 (**C**O), 171.0 (**C**O), 57.9 (**C**H), 46.8 (**C**H$_2$), 38.8 (**C**H$_2$), 32.5 (**C**H$_2$), 31.0 (**C**H$_2$), 28.4 (**C**H$_2$), 26.9 (**C**H$_2$), 26.7 (**C**H$_2$), 26.2 (**C**H$_2$), 25.8 (**C**H$_2$), 25.7 (**C**H$_2$), 25.6 (**C**H$_2$), 24.9 (**C**H$_2$), 24.8 (**C**H$_2$), 24.7 (**C**H$_2$), 24.5 (**C**H$_2$). Diagnostic ^{13}C NMR resonance for the minor rotamer 173.9 (**C**O), 169.4 (**C**O), 53.3 (**C**H), 47.0 (**C**H$_2$), 39.0 (**C**H$_2$), 33.5 (**C**H$_2$), 33.5 (**C**H$_2$), 29.3 (**C**H$_2$), 26.4(**C**H$_2$), 25.5 (**C**H$_2$), 25.2 (**C**H$_2$), 25.0 (**C**H$_2$), 24.6 (**C**H$_2$); HRMS (ESI): calcd. for $C_{20}H_{37}N_2O_2$, 337.2850 Found: [M+H]$^+$, 337.2853 (−1.4 ppm error).

4-(*Sec*-Butyl)-1,4-diazacycloundecane-2,5-dione (27h)

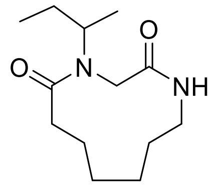

A mixture of 1-aza-2-cyclooctanone (50 mg, 0.393 mmol), DMAP (5 mg, 0.039 mmol) and pyridine (0.190 mL, 2.36 mmol) in DCM (3 mL) under an argon atmosphere was stirred at RT for 30 mins. Next, a solution of acid chloride **26d** (0.590 mmol, 1.50 equiv., prepared using the general procedure) in DCM (2 mL) was added and the resulting mixture was refluxed at 50 °C for 24 h. The mixture was then diluted with DCM (30 mL) and washed with 10% aq. HCl (30 mL). The aqueous layer was then extracted with DCM (3 × 30 mL) and the combined organic extracts dried over $MgSO_4$ and concentrated *in vacuo*. The crude material was then re-dissolved in DCM (4 mL) and DBU (0.6 mL, 3.94 mmol) was added, followed by stirring at RT overnight, before the solvent was removed *in vacuo*. Purification by flash column chromatography (SiO_2, 1:4 ethyl acetate: hexane → 1:1 ethyl acetate: hexane) afforded the *title compound* (10:1 mixture of rotamers) as a white solid (85 mg, 89%); m.p. 92–94 °C; R_f 0.30 (1:1 ethyl acetate: hexane); ν_{max}/cm^{-1} (thin film) 3300, 2927, 2854, 1649, 1456; δ_H (400 MHz, $CDCl_3$): data for the major rotamer 6.01 (1H, br s, NH), 4.82–4.68 (1H, m, NC**H**H-CO), 3.97–3.22 (4H, m, NCH**H**-CO, C**H₂**-NH and CHN), 2.42-2.09 (2H, m, CH_2CO), 1.91-0.84 (16H, m, 5 × CH_2 and 2 × CH_3); δ_C (100 MHz, $CDCl_3$): data for the major rotamer 175.2 (**C**O), 170.2 (**C**O), 50.2 (**C**H), 46.8 (**C**H₂), 39.2 (**C**H₂), 30.4 (**C**H₂), 28.8 (**C**H₂), 27.5 (**C**H₂), 26.1 (**C**H₂), 24.2 (**C**H₂), 22.3 (**C**H₂), 18.0 (**C**H₃), 10.9 (**C**H₃); Diagnostic ^{13}C NMR resonances for the minor rotamer 177.1 (**C**O), 170.1 (**C**O), 53.2 (**C**H), 48.3 (**C**H₂), 45.1 (**C**H₂), 41.7 (**C**H₂), 33.6 (**C**H₂), 29.4 (**C**H₂), 28.1 (**C**H₂), 27.2 (**C**H₂), 25.2 (**C**H₂), 23.5 (**C**H₃), 19.4 (**C**H₃); HRMS (ESI): calcd. for $C_{13}H_{24}N_2NaO_2$, 263.1730 Found: $[M+Na]^+$, 263.1733 (−1.2 ppm error).

4-Cyclopentyl-1,4-diazacyclohexadecane-2,5-dione (27i)

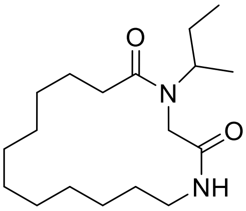

A mixture of laurolactam (155 mg, 0.786 mmol), DMAP (10 mg, 0.078 mmol) and pyridine (0.380 mL, 4.72 mmol) in DCM (5.50 mL) under an argon atmosphere was stirred at RT for 30 mins. Next, a solution of acid chloride **26d** (1.18 mmol, 1.50 equiv prepared using the general procedure) in DCM (3 mL) was added and the resulting mixture was refluxed at 50 °C for 24 h. The mixture was then diluted with DCM (30 mL) and washed with 10% aq. HCl (30 mL). The aqueous layer was then extracted with DCM (3 × 30 mL) and the combined organic extracts dried over $MgSO_4$ and concentrated *in vacuo*. The crude material was then re-dissolved in DCM (8 mL) and DBU (1.2 mL, 7.86 mmol) was added, followed by stirring at RT overnight, before the solvent was removed *in vacuo*. Purification by flash column chromatography (SiO_2, 1:4 ethyl acetate: hexane → 1:1 ethyl acetate: hexane) afforded the *title compound* (as a 10:1 mixture of rotamers) as a white solid (220 mg, 89%); m.p. 38–40 °C; R_f 0.30 (1:1 ethyl acetate: hexane); ν_{max}/cm^{-1} (thin film) 3301, 2925, 2856, 1621, 1455; δ_H (400 MHz, $CDCl_3$) 7.08 (1H, br s, N**H**, major), 6.27 (1H, br s, N**H**, minor), 4.60–4.52 (3H, m, C**H**$_2$ and C**H**, minor), 3.90–3.65 (3H, m, C**H**$_2$ and C**H**, major), 3.25–3.10 (2H, m, C**H**$_2$, major), 2.85–2.65 (2H, m, C**H**$_2$, minor), 2.45–2.30 (2H, m, C**H**$_2$, major), 2.18–2.07 (2H, m, C**H**$_2$, minor), 1.68–1.41 (8H, m, 2 × C**H**$_2$, both rotamers), 1.40–1.16 (32H, m, 8 × C**H**$_2$, both rotamers), 1.13 (3H, d, J = 6.9 Hz, C**H**$_3$, major), 0.99 (3H, d, J = 6.9 Hz, C**H**$_3$, minor), 0.78 (6H, t, J = 6.9 Hz, C**H**$_3$, both rotamers); δ_C (100 MHz, $CDCl_3$) data for the major rotamer only: 174.7 (**C**O), 170.6 (**C**O), 55.1 (**C**H), 45.9 (**C**H$_2$), 38.8 (**C**H$_2$), 32.4 (**C**H$_2$), 28.4 (**C**H$_2$), 27.3 (**C**H$_2$), 26.8 (**C**H$_2$), 26.7 (**C**H$_2$), 26.0 (**C**H$_2$), 25.7 (**C**H$_2$), 25.6 (**C**H$_2$), 24.9 (**C**H$_2$), 24.5 (**C**H$_2$), 24.4 (**C**H$_2$), 19.0 (**C**H$_3$), 10.9 (**C**H$_3$). Diagnostic ^{13}C NMR resonances for the minor rotamer: 174.1 (**C**O), 169.1 (**C**O), 50.6 (**C**H), 46.1 (**C**H$_2$), 33.3 (**C**H$_2$), 29.2 (**C**H$_2$), 27.0 (**C**H$_2$), 26.7 (**C**H$_2$), 26.6 (**C**H$_2$), 26.3 (**C**H$_2$), 26.2 (**C**H$_2$), 25.5 (**C**H$_2$), 24.8 (**C**H$_2$), 22.9 (**C**H$_2$), 17.4 (**C**H$_3$), 10.8 (**C**H$_3$); HRMS (ESI): calcd. for $C_{18}H_{35}N_2O_2$, 311.2693 Found: $[M+H]^+$, 311.2698 (−0.2 ppm error).

1,9-Diazabicyclo[9.2.0]tridecane-2,10-dione (27j)

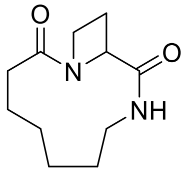

A mixture of 1-aza-2-cyclooctanone (100 mg, 0.786 mmol), DMAP (10 mg, 0.078 mmol) and pyridine (0.380 mL, 4.72 mmol) in DCM (5.5 mL) under an argon atmosphere was stirred at RT for 30 mins. Next, a solution of acid chloride **26e** (1.18 mmol, 1.50 equiv. prepared using the general procedure) in DCM (3 mL) was added and the resulting mixture was refluxed at 50 °C for 24 h. The mixture was then diluted with DCM (30 mL) and washed with 10% aq. HCl (30 mL). The aqueous layer was then extracted with DCM (3 × 30 mL) and the combined organic extracts dried over $MgSO_4$ and concentrated *in vacuo*. The crude material was then re-dissolved in DCM (8 mL) and DBU (1.2 mL, 7.86 mmol) was added, followed by stirring at RT overnight, before the solvent was removed *in vacuo*. Purification by flash column chromatography (SiO_2, 1:4 ethyl acetate: hexane → 1:1 ethyl acetate: hexane → ethyl acetate) afforded the *title compound* as a white solid (142 mg, 86%); m.p. 150–152 °C; R_f 0.20 (ethyl acetate); ν_{max}/cm^{-1} (thin film) 3283, 2928, 1622, 1551; δ_H (400 MHz, $CDCl_3$) 6.86 (1H, br s, N**H**), 4.92–4.70 (1H, m, CONC**H**), 4.09–3.86 (2H, m, CONC**H**$_2$), 3.72–3.51 (1H, m, CONCH'**H**), 3.26–3.02 (1H, m, CONC**H**'H), 2.59–2.38 (1H, m, CONHCH'**H**), 2.36–2.10 (2H, m, CONHC**H**'H and COCH'**H**), 2.00–1.82 (1H, m, COC**H**'H), 1.81–1.25 (6H, m, 3 × C**H**$_2$), 1.14–0.87 (2H, m, C**H**$_2$); δ_C (100 MHz, $CDCl_3$): 175.1 (**C**O), 170.9 (**C**O), 64.1 (**C**H), 46.1 (**C**H$_2$), 39.3 (**C**H$_2$), 29.0 (**C**H$_2$), 28.3 (**C**H$_2$), 25.1 (**C**H$_2$), 23.4 (**C**H$_2$), 21.6 (**C**H$_2$), 19.7 (**C**H$_2$); HRMS (ESI): calcd. for $C_{11}H_{19}N_2O_2$, 211.1441 Found: $[M+H]^+$, 211.1441 (0 ppm error).

Dodecahydropyrido[1,2-a][1,4]diazacyclotridecine-1,11(2*H*,13*H*)-dione (27l)

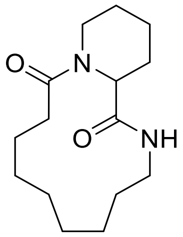

A mixture of octahydro-azecin-2-one (61 mg, 0.394 mmol), DMAP (5 mg, 0.039 mmol) and pyridine (0.19 mL, 2.360 mmol) in DCM (3 mL) under an argon atmosphere was stirred at RT for 30 mins. Next, a solution of acid chloride **26g** (0.590 mmol, 1.50 equiv. prepared using the general procedure) in DCM (2 mL) was added and the resulting mixture was refluxed at 50 °C for 24 h. The mixture was then diluted with DCM (10 mL) and washed with 10% aq. HCl (10 mL). The aqueous layer was then extracted with DCM (3 × 10 mL) and the combined organic extracts dried over $MgSO_4$ and concentrated *in vacuo*. The crude material was then re-dissolved in DCM (4 mL) and DBU (0.6 mL, 3.94 mmol) was added, followed by stirring at RT overnight, before the solvent was removed *in vacuo*. Purification by flash column chromatography (SiO_2, 1:4 ethyl acetate: hexane→ethyl acetate) afforded the *title compound* (as a 10:1 mixture of rotamers) as a white solid (91 mg, 87%); m.p. 65–67 °C; R_f 0.30 (ethyl acetate); ν_{max}/cm^{-1} (thin film) 3320, 2932, 2860, 1619, 1540; δ_H (400 MHz, $CDCl_3$) 6.44 (1H, br s, N**H**, major rotamer), 6.24 (1H, br s, N**H**, minor), 5.24–5.11 (1H, m, NC**H**, major), 4.72–4.58 (1H, m, NC**H**, minor), 4.54–4.40 (1H, m, NCH'**H**, minor), 3.87–3.72 (2H, m, NC**H**'H, major and NHCH'H, minor), 3.70–3.55 (1H, m, NHC**H**'H, major), 3.31–3.24 (1H, m, NHCH'**H**, minor), 3.14–2.98 (1H, m, NCH'**H**, major), 2.84–2.59 (3H, m, NHCH'**H**, major and COC**H**'H, both), 2.56–2.36 (2H, m, 2 × C**H**'H, minor), 2.80–1.87 (3H, m, 3 × C**H**'H, major), 1.83–1.07 (36H, m, 9 × C**H**$_2$, both); δ_C (100 MHz, $CDCl_3$): data for the major rotamer: 174.1 (**C**O), 170.8 (**C**O), 50.6 (**C**H), 44.3 (**C**H$_2$), 38.4 (**C**H$_2$), 32.7 (**C**H$_2$), 26.1 (**C**H$_2$), 25.6 (**C**H$_2$), 24.9 (**C**H$_2$), 24.8 (**C**H$_2$), 24.6 (**C**H$_2$), 24.5 (**C**H$_2$), 23.6 (**C**H$_2$), 23.4 (**C**H$_2$), 19.9 (**C**H$_2$). Diagnostic ^{13}C NMR resonances for the minor rotamer: 172.9 (**C**O), 170.3 (**C**O), 57.7 (**C**H), 39.3 (**C**H$_2$), 38.8 (**C**H$_2$), 30.5 (**C**H$_2$), 26.6 (**C**H$_2$), 25.1 (**C**H$_2$), 24.4 (**C**H$_2$), 23.7 (**C**H$_2$), 22.7 (**C**H$_2$), 20.3 (**C**H$_2$); HRMS (ESI): calcd. for $C_{15}H_{26}N_2NaO_2$, 289.1886 Found: $[M+Na]^+$, 289.1891 (−2.1 ppm error).

4-(2,2,2-Trifluoroethyl)-1,4-diazacycloundecane-2,5-dione (27m)

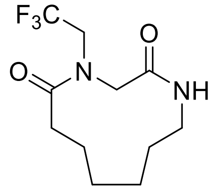

A mixture of 1-aza-2-cyclooctanone (100 mg, 0.786 mmol), DMAP (10 mg, 0.078 mmol) and pyridine (0.380 mL, 4.72 mmol) in DCM (5.50 mL) under an argon atmosphere was stirred at RT for 30 mins. Next, a solution of acid chloride **26h** (1.18 mmol, 1.50 equiv. prepared using the general procedure) in DCM (3 mL) was added and the resulting mixture was refluxed at 50 °C for 24 h. The mixture was then diluted with DCM (30 mL) and washed with 10% aq. HCl (30 mL). The aqueous layer was then extracted with DCM (3 × 30 mL) and the combined organic extracts dried over $MgSO_4$ and concentrated *in vacuo*. The crude material was then re-dissolved in DCM (8 mL) and DBU (1.2 mL, 7.86 mmol) was added, followed by stirring at RT overnight, before the solvent was removed *in vacuo*. Purification by flash column chromatography (SiO_2, 1:4 ethyl acetate: hexane → 1:1 ethyl acetate: hexane) afforded the *title compound* as a white solid (186 mg, 89%); m.p. 188–190 °C; R_f 0.30 (1:1 ethyl acetate: hexane); ν_{max}/cm^{-1} (thin film) 3308, 1657, 1469; δ_H (400 MHz, $CDCl_3$) 8.14 (1H, br s, N**H**), 4.38–4.14 (2H, m, C**H**$_2$), 4.08 (2H, s, C**H**$_2$), 3.29–3.07 (2H, m, C**H**$_2$), 2.23 (2H, t, *J* = 6.9 Hz, C**H**$_2$), 1.60–1.40 (4H, m, 2 × C**H**$_2$), 1.35–1.12 (4H, m, 2 × C**H**$_2$); δ_F (376 MHz, $CDCl_3$) -69.93 (3F, t, *J* = 9.3 Hz, C**F**$_3$); δ_C (100 MHz, $CDCl_3$): 174.6 (**C**O), 169.0 (**C**O), 124.0 (CF_3, q, *J* = 280.8 Hz), 53.0 (**C**H_2), 47.9 (**C**H_2, q, *J* = 64.2 and 32.6 Hz), 37.9 (**C**H_2), 29.4 (**C**H_2), 27.2 (**C**H_2), 24.4 (**C**H_2), 23.0 (**C**H_2), 21.3 (**C**H_2); HRMS (ESI): calcd. for $C_{11}H_{17}F_3N_2NaO_2$, 289.1134 Found: $[M+H]^+$, 289.1137 (−0.6 ppm error).

4-(2,2,2-Trifluoroethyl)-1,4-diazacyclohexadecane-2,5-dione (27n)

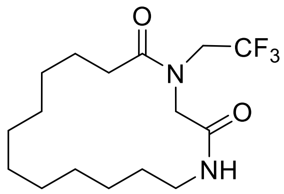

A mixture of laurolactam (155 mg, 0.786 mmol), DMAP (10 mg, 0.078 mmol) and pyridine (0.380 mL, 4.720 mmol) in DCM (5.50 mL) under an argon atmosphere was stirred at RT for

30 mins. Next, a solution of acid chloride **26h** (1.18 mmol, 1.50 equiv. prepared using the general procedure) in DCM (3 mL) was added and the resulting mixture was refluxed at 50 °C for 24 h. The mixture was then diluted with DCM (30 mL) and washed with 10% aq. HCl (30 mL). The aqueous layer was then extracted with DCM (3 × 30 mL) and the combined organic extracts dried over $MgSO_4$ and concentrated *in vacuo*. The crude material was then re-dissolved in DCM (8 mL) and DBU (1.20 mL, 7.860 mmol) was added, followed by stirring at RT overnight, before the solvent was removed *in vacuo*. Purification by flash column chromatography (SiO_2, 1:4 ethyl acetate: hexane → 1:1 ethyl acetate: hexane) afforded the *title compound* (as a 2:1 mixture of rotamers) as a white solid (251 mg, 93%); m.p. 192–194 °C; R_f 0.66 (9:1 ethyl acetate: methanol); ν_{max}/cm^{-1} (thin film) 3335, 2932, 2858, 1675, 1554; δ_H (400 MHz, $CDCl_3$) 6.28 (1H, br s, N**H**, major), 5.80 (1H, br s, N**H**, minor), 4.23–3.94 (8H, m, 2 × C**H**$_2$, both), 3.44–3.34 (2H, m, C**H**$_2$, minor), 3.33–3.19 (2H, m, C**H**$_2$, major), 2.43 (2H, t, *J* = 7.6 Hz, C**H**$_2$, major), 2.29 (2H, t, *J* = 7.6 Hz, C**H**$_2$, minor), 1.80–1.60 (4H, m, 2 × C**H**$_2$, major), 1.59–1.42 (4H, m, 2 × C**H**$_2$, minor), 1.40–1.16 (28H, m, 7 × C**H**$_2$, both); δ_C (100 MHz, $CDCl_3$): 174.7 (**C**O, major), 174.5 (**C**O, minor), 168.7 (**C**O, major), 167.3 (**C**O, minor), 123.5 (**C**F$_3$, q, *J* = 279.9 Hz, both), 52.1 (**C**H$_2$, major), 51.8 (**C**H$_2$, minor), 50.4 (**C**H$_2$, q, *J* = 33.5 Hz, major), 47.3 (**C**H$_2$, q, *J* = 33.5 Hz, minor), 39.1 (NH**C**H$_2$, both rotamers), 32.3 (**C**H$_2$, minor), 31.6 (**C**H$_2$, major), 29.1 (**C**H$_2$, minor), 28.9 (**C**H$_2$, major), 27.2 (**C**H$_2$, major), 27.0 (**C**H$_2$, minor), 26.8 (**C**H$_2$, minor), 26.7 (**C**H$_2$, major), 26.6 (**C**H$_2$, minor), 26.6 (**C**H$_2$, minor), 26.4 (**C**H$_2$, minor), 26.2 (**C**H$_2$, major), 26.1 (**C**H$_2$, major), 25.9 (**C**H$_2$, both), 25.2 (**C**H$_2$, minor), 25.0 (**C**H$_2$, major), 24.9 (**C**H$_2$, minor), 24.7 (**C**H$_2$, major), 23.9 (**C**H$_2$, major), 22.9 (**C**H$_2$, minor). HRMS (ESI): calcd. for $C_{16}H_{28}F_3N_2O_2$, 337.2097 Found: $[M+H]^+$, 337.2097 (−0.2 ppm error).

4-(4-Methoxybenzyl)-1,4-diazacycloundecane-2,5-dione (27o)

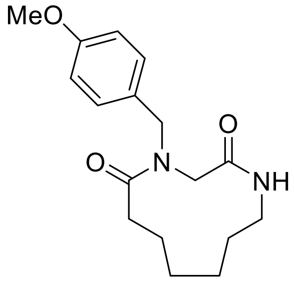

A mixture of 1-aza-2-cyclooctanone (100 mg, 0.786 mmol), DMAP (10 mg, 0.0780 mmol) and pyridine (0.380 mL, 4.72 mmol) in DCM (5.50 mL) under an argon atmosphere was stirred at RT for 30 mins. Next, a solution of acid chloride **26i** (1.18 mmol, 1.50 equiv. prepared using the general procedure) in DCM (3 mL) was added and the resulting mixture was refluxed at 50 °C for 24 h. The mixture was then diluted with DCM (30 mL) and washed with 10% aq. HCl (30 mL). The aqueous layer was then extracted with DCM (3 × 30 mL) and the combined organic extracts dried over $MgSO_4$ and concentrated *in vacuo*. The crude material was then re-dissolved in DCM (8 mL) and DBU (1.20 mL, 7.86 mmol) was added, followed by stirring at RT overnight, before the solvent was removed *in vacuo*. Purification by flash column chromatography (SiO_2, 1:4 → 1:1 ethyl acetate: hexane) afforded the *title compound* (20:1 mixture of rotamers) as a white solid (194 mg, 82%); m.p. 92–94 °C; R_f 0.30 (1:1 ethyl acetate: hexane); ν_{max}/cm^{-1} (thin film) 3308, 1657, 1469; δ_H (400 MHz, $CDCl_3$) 7.26 (2H, d, J = 8.4 Hz, Ar-H, major rotamer), 7.14 (2H, d, J = 8.4 Hz, Ar-H, minor rotamer), 6.86 (2H, d, J = 8.4 Hz, Ar-H, major), 6.63 (2H, d, J = 8.4 Hz, Ar-H, minor), 5.82 (1H, br s, N**H**, minor), 5.54 (1H, br s, N**H**, major), 4.89–4.46 (4H, m, C**H**$_2$, both), 4.43–4.30 (2H, m, C**H**$_2$, minor), 4.08–3.85 (2H, m, C**H**$_2$, major), 3.77 (6H, s, C**H**$_3$, both), 3.40–3.13 (4H, m, C**H**$_2$, both), 2.61–2.14 (4H, m, C**H**$_2$, both), 2.07–0.90 (16H, m, 4 × C**H**$_2$, both); δ_C (100 MHz, $CDCl_3$) data for the major rotamer: 174.8 (**C**O), 169.6 (**C**O), 159.4 (**C**), 129.9 (**C**H), 129.3 (**C**), 114.4 (**C**H), 55.2 (**C**H$_3$), 52.8 (**C**H$_2$), 51.0 (**C**H$_2$), 38.9 (**C**H$_2$), 30.2 (**C**H$_2$), 28.8 (**C**H$_2$), 26.0 (**C**H$_2$), 24.4 (**C**H$_2$), 22.4 (**C**H$_2$). Diagnostic ^{13}C NMR resonances for the minor rotamer: 176.5 (**C**O), 170.0 (**C**O), 159.2 (**C**), 128.5 (**C**H), 114.2 (**C**H), 51.8 (**C**H$_2$), 50.2 (**C**H$_2$), 39.3 (**C**H$_2$), 33.0 (**C**H$_2$), 26.9 (**C**H$_2$), 26.8 (**C**H$_2$), 23.6 (**C**H$_2$), 23.2 (**C**H$_2$); HRMS (ESI): calcd. for $C_{17}H_{24}N_2NaO_3$, 327.1679 Found: $[M+Na]^+$, 327.1682 (0.5 ppm error).

4-(4-Methoxybenzyl)-1,4-diazacyclohexadecane-2,5-dione (27p)

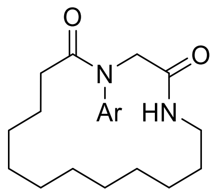

Ar = PMB

A mixture of laurolactam (155 mg, 0.786 mmol), DMAP (10 mg, 0.078 mmol) and pyridine (0.380 mL, 4.72 mmol) in DCM (5.5 mL) under an argon atmosphere was stirred at RT for 30 mins. Next, a solution of acid chloride **26i** (1.18 mmol, 1.50 equiv. prepared using the general procedure) in DCM (3 mL) was added and the resulting mixture was refluxed at 50 °C for 24 h. The mixture was then diluted with DCM (30 mL) and washed with 10% aq. HCl (30 mL). The aqueous layer was then extracted with DCM (3 × 30 mL) and the combined organic extracts dried over $MgSO_4$ and concentrated *in vacuo*. The crude material was then re-dissolved in DCM (8 mL) and DBU (1.2 mL, 7.86 mmol) was added, followed by stirring at RT overnight, before the solvent was removed *in vacuo*. Purification by flash column chromatography (SiO_2, 1:4 ethyl acetate: hexane → 1:1 ethyl acetate: methanol) afforded the *title compound* (as a 3:1 mixture of rotamers) as a yellow solid (274 mg, 91%); m.p. 148–150 °C; R_f 0.66 (9:1 ethyl acetate: methanol); ν_{max}/cm^{-1} (thin film) 3302, 2927, 2855, 1652, 1545; δ_H (400 MHz, $CDCl_3$) 7.22 (2H, d, *J* = 8.4 Hz, Ar, minor), 7.09 (2H, d, *J* = 9.2 Hz, Ar, major), 6.87 (2H, d, *J* = 9.2 Hz, Ar, major), 6.84 (2H, d, *J* = 8.4 Hz, Ar, minor), 6.67 (1H, br s, N**H**, major), 5.73 (1H, br s, N**H**, minor), 4.61 (2H, s, C**H**$_2$, major), 4.57 (2H, s, C**H**$_2$, minor), 3.93 (4H, s, C**H**$_2$, both), 3.79 (3H, s, OC**H**$_3$, major), 3.78 (3H, s, OC**H**$_3$, minor), 3.30–3.15 (4H, s, C**H**$_2$, both), 2.46 (2H, t, *J* = 6.1 Hz, C**H**$_2$, major), 2.26 (2H, t, *J* = 7.6 Hz, C**H**$_2$, minor), 1.75–1.60 (4H, m, C**H**$_2$, both), 1.15–1.10 (32H, m, 8 × C**H**$_2$, both); δ_C (100 MHz, $CDCl_3$): 174.9 (**C**O, major), 174.0 (**C**O, minor), 169.7 (**C**O, major), 168.4 (**C**O, minor), 159.4 (**C,** minor), 159.3 (**C,** major), 130.1 (**C,** major), 129.2 (**C,** minor), 128.1 (**C**H, major), 127.9 (**C**H, minor), 114.4 (**C**H, major), 114.3 (**C**H, minor), 55.4 (O**C**H$_3$, both), 52.5 (**C**H$_2$Ar, major), 51.6 (N**C**H$_2$CO, major), 51.4 (**C**H$_2$Ar, minor), 50.1 (N**C**H$_2$CO, minor), 39.2 (NH**C**H$_2$, both), 32.9 (**C**H$_2$CONAr, minor), 32.2 (**C**H$_2$CONAr, major), 29.3 (**C**H$_2$, minor), 28.7 (**C**H$_2$, major), 27.1 (**C**H$_2$, both), 27.0 (**C**H$_2$, major), 26.9 (**C**H$_2$, minor), 26.5 (**C**H$_2$, minor), 26.4 (2 × **C**H$_2$, major), 26.2 (**C**H$_2$, both), 26.0 (**C**H$_2$, both), 25.5 (2 × **C**H$_2$, minor), 25.2 (**C**H$_2$, major), 25.0 (**C**H$_2$, major),

24.9 (**C**H_2, major), 24.8 (**C**H_2, minor), 24.7 (**C**H_2, major). HRMS (ESI): calcd. for $C_{22}H_{35}N_2O_3$, 375.2642. Found: $[M+H]^+$, 375.2644 (0.4 ppm error).

4-(2-Methoxyethyl)-1,4-diazacycloundecane-2,5-dione (27q)

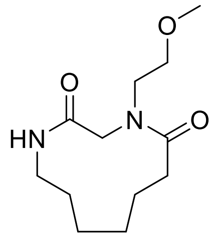

A mixture of 1-aza-2-cyclooctanone (64.2 mg, 0.505 mmol), DMAP (9.0 mg, 0.074 mmol) and pyridine (0.240 mL, 3.00 mmol) in DCM (5 mL) under an argon atmosphere was stirred at RT for 30 mins. Next, a solution of acid chloride **26j** (0.776 mmol, 1.50 equiv. prepared using the general procedure) in DCM (5 mL) was added and the resulting mixture was refluxed at 50 °C for 18 h. The mixture was then diluted with DCM (30 mL) and washed with 10% aq. HCl (15 mL). The aqueous layer was then extracted with DCM (3 × 15 mL) and the combined organic extracts dried over $MgSO_4$ and concentrated *in vacuo*. The crude material was then re-dissolved in DCM (5 mL) and DBU (0.750 mL, 5.00 mmol) was added, followed by stirring at RT for 18 h, before the solvent was removed *in vacuo*. Purification by flash column chromatography (SiO_2, 1:9 ethyl acetate: hexane → 19:1 ethyl acetate: methanol) afforded the *title compound* as a pasty orange solid (85.4 mg, 70%); R_f 0.20 (9:1 ethyl acetate: methanol); m.p. 104–105 °C; ν_{max}/cm^{-1} (neat) 3368, 2915, 1667, 1624, 1524, 1470, 1412, 1364, 1213, 1113, 1046, 990, 672); All ^1H signals broadened due to rotamer interconversion. δ_H (400 MHz, DMSO-d_6, 80 °C) 7.64 (1H, br s, N**H**), 3.97 (2H, s, NC**H**$_2$CO), 3.60 (2H, t, J = 5.6 Hz, C**H**$_2$OCH_3,), 3.52 (2H, t, J = 5.6 Hz, C**H**$_2$CH_2OCH_3,), 3.29 (3H, s, CH_2OC**H**$_3$), 3.28–3.19 (2H, m, C**H**$_2$NH), 2.18 (2H, t, J = 6.8 Hz, C**H**$_2$CON), 1.59–1.45 (4H, m, (C**H**$_2$)$_2$), 1.32–1.21 (4H, m, (C**H**$_2$)$_2$); δ_C (100 MHz, $CDCl_3$): 175.8 (**C**O), 170.7 (**C**O), 69.2 (**C**H_2OCH_3), 58.3 (O**C**H_3), 55.3 (N**C**H_2), 49.1 (N**C**H_2), 38.9 (**C**H_2NH), 30.4 (**C**H_2CON), 29.0 (**C**H_2), 26.9 (**C**H_2), 25.2 (**C**H_2), 22.9 (**C**H_2); HRMS (ESI): calcd. for $C_{12}H_{22}N_2NaO_3$, 265.1523. Found: $[MNa]^+$, 265.1527 (–1.6 ppm error).

(*R*)-4-(1-Phenylethyl)-1,4-diazacycloundecane-2,5-dione (27r)

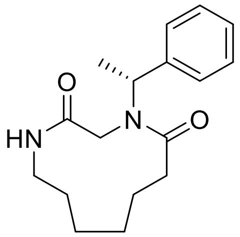

A mixture of 1-aza-2-cyclooctanone (66.0 mg, 0.519 mmol), DMAP (10.4 mg, 0.085 mmol) and pyridine (0.240 mL, 3.00 mmol) in DCM (5 mL) under an argon atmosphere was stirred at RT for 30 mins. Next, a solution of acid chloride **26k** (0.771 mmol, 1.50 equiv. prepared using the general procedure) in DCM (5 mL) was added and the resulting mixture was refluxed at 50 °C for 19 h. The mixture was then diluted with DCM (30 mL) and washed with 10% aq. HCl (30 mL). The aqueous layer was then extracted with DCM (3 × 15 mL) and the combined organic extracts dried over $MgSO_4$ and concentrated *in vacuo*. The crude material was then re-dissolved in DCM (5 mL) and DBU (0.75 mL, 5.00 mmol) was added, followed by stirring at RT for 18 h, before the solvent was removed *in vacuo*. Purification by flash column chromatography (SiO_2, 1:9 ethyl acetate: hexane → 1:1 ethyl acetate: hexane → ethyl acetate → 19:1 ethyl acetate: methanol) afforded the *title compound* as an off white solid (99.4 mg, 66%); R_f 0.33 (ethyl acetate); m.p. 189–193 °C; ν_{max}/cm^{-1} (neat) 3334, 2933, 1640, 1539, 1450, 1395, 1179, 1151, 748, 697); All ^{1}H signals broadened due to rotamer interconversion. δ_H (400 MHz, $CDCl_3$, 55 °C) 7.39–7.23 (5H, m, Ar**H**), 6.25 (1H, t, *J* = 7.1 Hz, C**H**CH_3), 5.35–5.16 (1H, m, N**H**), 3.81–3.69 (2H, m, NC$\mathbf{H_2}$CO), 3.48–3.26 (1H, m, C**H**H′NH), 3.05–2.76 (1H, m, CH**H′**NH), 2.36–2.18 (2H, m, C$\mathbf{H_2}$CON), 1.91–1.55 (3H, m, 3 from C$\mathbf{H_2}$), 1.48 (3H, d, *J* = 7.1 Hz, CHC$\mathbf{H_3}$), 1.43–1.05 (5H, m, 5 from C$\mathbf{H_2}$); δ_C (100 MHz, $CDCl_3$) 175.1 (**C**O), 170.0 (**C**O), 140.6 (Ar**C**), 129.3 (2C, Ar**C**H), 128.4 (Ar**C**H), 127.4 (2C, Ar**C**H), 50.6 (**C**HCH_3), 47.6 (N**C**H_2CO), 38.9 (**C**H_2NH), 30.7 (**C**H_2CON), 29.1 (**C**H_2), 26.5 (**C**H_2), 24.8 (**C**H_2), 22.6 (**C**H_2), 16.1 (**C**H_3); HRMS (ESI): calcd. for $C_{17}H_{25}N_2O_2$, 289.1911. Found: $[MH]^+$, 289.1911 (0.1 ppm error).

4-(Prop-2-en-1-yl)-1,4-diazacycloundecane-2,5-dione (27s)

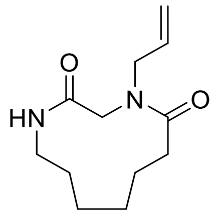

A mixture of 1-aza-2-cyclooctanone (65.0 mg, 0.511 mmol), DMAP (6.1 mg, 0.050 mmol) and pyridine (0.240 mL, 3.00 mmol) in DCM (5 mL) under an argon atmosphere was stirred at RT for 30 mins. Next, a solution of acid chloride **26l** (0.831 mmol, 1.50 equiv prepared using the general procedure) in DCM (5 mL) was added and the resulting mixture was refluxed at 50 °C for 18 h. The mixture was then diluted with DCM (30 mL) and washed with 10% aq. HCl (15 mL). The aqueous layer was then extracted with DCM (3 × 15 mL) and the combined organic extracts dried over $MgSO_4$ and concentrated *in vacuo*. The crude material was then re-dissolved in DCM (5 mL) and DBU (0.750 mL, 5.00 mmol) was added, followed by stirring at RT for 18 h, before the solvent was removed *in vacuo*. Purification by flash column chromatography (SiO_2, 1:9 ethyl acetate: hexane → 19:1 ethyl acetate: methanol) afforded the *title compound* (as an 7:1 mixture of rotamers) as an off white solid (99.5 mg, 87%); m.p. 122–125 °C; R_f 0.41 (9:1 ethyl acetate: methanol); ν_{max}/cm^{-1} (neat) 3291, 2933, 1627, 1547, 1465, 1408, 913, 727); δ_H (400 MHz, $CDCl_3$) data for the major rotamer only; 6.03 (1H, br m, N**H**), 5.74 (1H, ddt, *J* = 16.8, 10.2, 6.8 Hz, C**H**CH_2), 5.19–5.12 (2H, m, CHC**H₂**), 4.09–3.97 (2H, m, NC**H₂**CH), 3.92–3.84 (2H, s, NC**H₂**CO), 3.46–3.35 (2H, m, C**H₂**NH), 2.32–2.20 (2H, m, C**H₂**CON), 1.71–1.15 (8H, m, (C**H₂**)₄); Diagnostic ^{1}H NMR resonances for the minor rotamer: 6.53 (1H, br d, N**H**, *J* = 10.2 Hz), 4.81 (1H, dd, NC**H₂**CO, *J* = 13.0, 1.3 Hz), 2.99 (1H, d, NC**H₂**CO, *J* = 13.0 Hz); δ_C (100 MHz, $CDCl_3$): Major rotamer: 174.8 (**C**O), 169.8 (**C**O), 132.6 (**C**HCH_2), 119.4 (CH**C**H_2), 52.3 (N**C**H_2CO), 50.8 (N**C**H_2CH), 39.3 (**C**H_2NH), 30.5 (**C**H_2CON), 28.9 (**C**H_2), 25.9 (**C**H_2), 24.2 (**C**H_2), 22.5 (**C**H_2); Minor rotamer: 176.7 (**C**O), 170.4 (**C**O), 132.4 (**C**HCH_2), 117.7 (CH**C**H_2), 51.7 (N**C**H_2CH), 51.2 (N**C**H_2CO), 39.4 (**C**H_2NH), 32.8 (**C**H_2CON), 27.1 (**C**H_2), 26.9 (**C**H_2), 23.6 (**C**H_2), 23.3 (**C**H_2); HRMS (ESI): calcd. for $C_{12}H_{21}N_2O_2$, 225.1598. Found: $[MH]^+$, 225.1597 (0.1 ppm error).

4-(Prop-2-yn-1-yl)-1,4-diazacycloundecane-2,5-dione (27t)

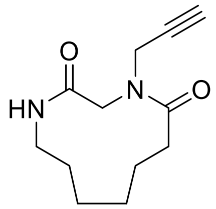

A mixture of 1-aza-2-cyclooctanone (65.6 mg, 0.516 mmol), DMAP (12.0 mg, 0.0960 mmol) and pyridine (0.240 mL, 3.00 mmol) in DCM (5 mL) under an argon atmosphere was stirred at RT for 30 mins. Next, a solution of acid chloride **26m** (0.750 mmol, 1.50 equiv prepared using the general procedure) in DCM (5 mL) was added and the resulting mixture was refluxed at 50 °C for 18 h. The mixture was then diluted with DCM (30 mL) and washed with 10% aq. HCl (15 mL). The aqueous layer was then extracted with DCM (3 × 15 mL) and the combined organic extracts dried over $MgSO_4$ and concentrated *in vacuo*. The crude material was then re-dissolved in DCM (5 mL) and DBU (0.75 mL, 5.00 mmol) was added, followed by stirring at RT for 18 h, before the solvent was removed *in vacuo*. Purification by flash column chromatography (SiO_2, 1:9 ethyl acetate: hexane → 1:1 ethyl acetate: hexane → ethyl acetate → 9:1 ethyl acetate: methanol) afforded the *title compound* as an off white solid (112.6 mg, 98%); m.p. 148–150 °C; R_f 0.36 (9:1 ethyl acetate: methanol); ν_{max}/cm^{-1} (neat) 3293, 2944, 1642, 1560, 1458, 1410, 1222, 1186, 914, 762, 596); All ^1H signals broadened due to rotamer interconversion. δ_H (400 MHz, $CDCl_3$, 55 °C) 6.16 (1H, br s, N**H**), 4.43–4.21 (2H, m, NC**H₂**CCH), 4.09 (2H, br s, NC**H₂**CO), 3.62–3.26 (2H, m, C**H₂**NH), 2.53–2.23 (3H, m, C**H₂**CON and NCH₂CC**H**), 1.83–1.17 (8H, m, (C**H₂**)₄); δ_C (100 MHz, $CDCl_3$): 174.9 (**C**O), 169.7 (**C**O), 78.9 (CH₂**C**CH), 73.6 (CH₂C**C**H), 53.1 (N**C**H₂CO), 39.1 (**C**H₂NH), 37.3 (N**C**H₂CCH), 30.3 (**C**H₂CON), 29.0 (**C**H₂), 26.4 (**C**H₂), 24.6 (**C**H₂), 22.6 (**C**H₂); HRMS (ESI): calcd. for $C_{12}H_{18}N_2NaO_2$, 245.1260. Found: $[MNa]^+$, 245.1262 (−0.7 ppm error).

4-Isopropyl-1,4-diazacycloundecane-2,5-dione (27u)

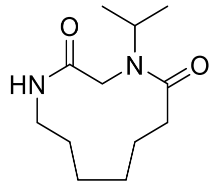

A mixture of 1-aza-2-cyclooctanone (64.2 mg, 0.505 mmol), DMAP (9.60 mg, 0.079 mmol) and pyridine (0.240 mL, 3.00 mmol) in DCM (5 mL) under an argon atmosphere was stirred

at RT for 30 mins. Next, a solution of acid chloride **26n** (0.766 mmol, 1.50 equiv prepared using the general procedure) in DCM (5 mL) was added and the resulting mixture was refluxed at 50 °C for 18 h. The mixture was then diluted with DCM (30 mL) and washed with 10% aq. HCl (15 mL). The aqueous layer was then extracted with DCM (3 × 15 mL) and the combined organic extracts dried over $MgSO_4$ and concentrated *in vacuo*. The crude material was then re-dissolved in DCM (5 mL) and DBU (0.75 mL, 5.00 mmol) was added, followed by stirring at RT for 18 h, before the solvent was removed *in vacuo*. Purification by flash column chromatography (SiO_2, 1:9 ethyl acetate: hexane → 1:1 ethyl acetate: hexane → ethyl acetate → 19:1 ethyl acetate: methanol) afforded the *title compound* as a light brown solid (63.2 mg, 55%); m.p. 132 – 136 °C; R_f 0.38 (9:1 ethyl acetate: methanol); ν_{max}/cm^{-1} (neat) 3321, 2938, 1642, 1540, 1466, 1397, 1196, 1146, 1008, 756, 715, 614, 587); All ^{1}H signals broadened due to rotamer interconversion. δ_H (400 MHz, $CDCl_3$, 55 °C) 5.93 (1H, br s, N**H**), 5.00–4.85 (1H, m, C**H**(CH_3)$_2$), 3.81 (2H, s, NC**H₂**CO), 3.58–3.18 (2H, m, C**H₂**NH), 2.30–2.16 (2H, m, C**H₂**CON), 1.87–1.18 (8H, m, (C**H₂**)$_4$), 1.09 (6H, d, *J* = 6.8 Hz, CH(C**H₃**)$_2$); δ_C (100 MHz, $CDCl_3$): 175.1 (**C**O), 170.4 (**C**O), 46.6 (N**C**H_2CO), 44.6 (N**C**HCH_2), 39.5 (**C**H_2NH), 30.8 (**C**H_2CON), 29.0 (**C**H_2), 26.2 (**C**H_2), 24.4 (**C**H_2), 22.5 (**C**H_2), 20.4 (2C, **C**H_3); HRMS (ESI): calcd. for $C_{12}H_{22}N_2NaO_2$, 249.1573. Found: [MNa]$^+$, 249.1573 (0.3 ppm error); Diagnostic ^{1}H NMR resonances reported at room temperature: δ_H (400 MHz, $CDCl_3$) 4.94 (1H, hept, *J* = 6.8 Hz, C**H**(CH_3)$_2$), 1.07 (6H, d, *J* = 6.8 Hz, CH(C**H₃**)$_2$).

4-(Furan-2-ylmethyl)-1,4-diazacycloundecane-2,5-dione (27v)

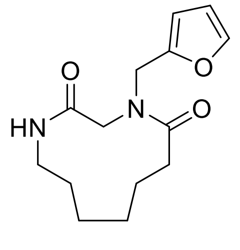

A mixture of 1-aza-2-cyclooctanone (67.0 mg, 0.527 mmol), DMAP (6.10 mg, 0.050 mmol) and pyridine (0.240 mL, 3.00 mmol) in DCM (5 mL) under an argon atmosphere was stirred at RT for 30 mins. Next, a solution of acid chloride **26o** (0.773 mmol, 1.50 equiv prepared using the general procedure) in DCM (5 mL) was added and the resulting mixture was refluxed at 50 °C for 18 h. The mixture was then diluted with DCM (30 mL) and washed with 10% aq. HCl (15 mL). The aqueous layer was then extracted with DCM (3 × 15 mL) and the combined

organic extracts dried over $MgSO_4$ and concentrated *in vacuo*. The crude material was then re-dissolved in DCM (5 mL) and DBU (0.75 mL, 5.00 mmol) was added, followed by stirring at RT for 21 h, before the solvent was removed *in vacuo*. Purification by flash column chromatography (SiO_2, 1:9 ethyl acetate: hexane → 1:1 ethyl acetate: hexane → ethyl acetate → 19:1 ethyl acetate: methanol) afforded the *title compound* as a beige solid (66.4 mg, 48%); R_f 0.21 (ethyl acetate); m.p. 71–87 °C; ν_{max}/cm^{-1} (thin film) 3318, 2934, 1639, 1536, 1463, 1147, 1010, 734, 599); All ^1H signals broadened due to rotamer interconversion. δ_H (400 MHz, $CDCl_3$, 55 °C) 7.34–7.32 (1H, m, Ar**H**), 6.32–6.28 (2H, m, Ar**H**), 5.73 (1H, br s, N**H**), 4.65 (2H, s, NC**H₂**Ar), 3.99 (2H, s, NC**H₂**CO), 3.40–3.18 (2H, m, C**H₂**NH), 2.37–2.21 (2H, m, C**H₂**CON), 1.77–1.14 (8H, m, (C**H₂**)₄); δ_C (100 MHz, $CDCl_3$) 175.0 (**C**O), 169.7 (**C**O), 150.4 (Ar**C**), 142.7 (Ar**C**H), 111.1 (Ar**C**H), 109.8 (Ar**C**H), 53.0 (N**C**H₂CO), 44.1 (N**C**H₂Ar), 39.2 (**C**H₂NH), 30.3 (**C**H₂CON), 29.0 (**C**H₂), 26.3 (**C**H₂), 24.6 (**C**H₂), 22.6 (**C**H₂); HRMS (ESI): calcd. for $C_{14}H_{20}N_2NaO_3$, 287.1366. Found: $[MNa]^+$, 287.1357 (3.0 ppm error).

4-[(Thiophen-2-yl)methyl]-1,4-diazacycloundecane-2,5-dione (27w)

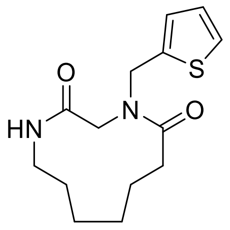

A mixture of 1-aza-2-cyclooctanone (63.8 mg, 0.502 mmol), DMAP (8.0 mg, 0.066 mmol) and pyridine (0.240 mL, 3.00 mmol) in DCM (5 mL) under an argon atmosphere was stirred at RT for 30 mins. Next, a solution of acid chloride **26p** (0.795 mmol, 1.50 equiv prepared using the general procedure) in DCM (5 mL) was added and the resulting mixture was refluxed at 50 °C for 18 h. The mixture was then diluted with DCM (30 mL) and washed with 10% aq. HCl (15 mL). The aqueous layer was then extracted with DCM (3 × 15 mL) and the combined organic extracts dried over $MgSO_4$ and concentrated *in vacuo*. The crude material was then re-dissolved in DCM (5 mL) and DBU (0.75 mL, 5.00 mmol) was added, followed by stirring at RT for 18 h, before the solvent was removed *in vacuo*. Purification by flash column chromatography (SiO_2, 1:9 ethyl acetate: hexane→19:1 ethyl acetate: methanol) afforded the *title compound* as an off white solid (98.6 mg, 70%); m.p. 123–128 °C; R_f 0.50 (9:1 ethyl acetate: methanol); ν_{max}/cm^{-1} (neat) 3316, 2933, 1633, 1536, 1463, 1186, 911, 702, 591; All ^1H signals broadened

due to rotamer interconversion. δ_H (400 MHz, $CDCl_3$, 55 °C) 7.23 (1H, dd, *J* = 5.1, 1.1 Hz, Ar**H**), 7.02–6.98 (1H, m, Ar**H**), 6.92 (1H, dd, *J* = 5.1, 3.4 Hz, Ar**H**), 5.56 (1H, br s, N**H**), 4.78 (2H, s, NC**H₂**Ar), 4.00 (2H, s, NC**H₂**CO), 3.32–3.12 (2H, m, C**H₂**NH), 2.32–2.24 (2H, m, C**H₂**CON), 1.76–1.14 (8H, m, (C**H₂**)₄); δ_C (100 MHz, $CDCl_3$) 174.8 (**C**O), 169.6 (**C**O), 139.4 (Ar**C**), 127.9 (Ar**C**H), 127.2 (Ar**C**H), 126.7 (Ar**C**H), 53.5 (N**C**H_2CO), 46.7 (N**C**H_2Ar), 39.3 (**C**H_2NH), 30.3 (**C**H_2CON), 28.9 (**C**H_2), 26.1 (**C**H_2), 24.5 (**C**H_2), 22.6 (**C**H_2); HRMS (ESI): calcd. for $C_{14}H_{21}N_2O_2S$, 281.1318. Found: $[MH]^+$, 281.1321 (–1.1 ppm error).

4-(Benzo[d][1,3]dioxol-5-ylmethyl)-1,4-diazacycloundecane-2,5-dione (27x)

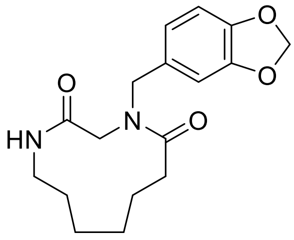

A mixture of 1-aza-2-cyclooctanone (64.1 mg, 0.504 mmol), DMAP (5.2 mg, 0.043 mmol) and pyridine (0.240 mL, 3.00 mmol) in DCM (5 mL) under an argon atmosphere was stirred at RT for 30 mins. Next, a solution of acid chloride **26q** (0.750 mmol, 1.50 eqv prepared using the general procedure) in DCM (5 mL) was added and the resulting mixture was refluxed at 50 °C for 18 h. The mixture was then diluted with DCM (30 mL) and washed with 10% aq. HCl (15 mL). The aqueous layer was then extracted with DCM (3 × 15 mL) and the combined organic extracts dried over $MgSO_4$ and concentrated *in vacuo*. The crude material was then re-dissolved in DCM (5 mL) and DBU (0.75 mL, 5.00 mmol) was added, followed by stirring at RT for 18 h, before the solvent was removed *in vacuo*. Purification by flash column chromatography (SiO_2, 1:9 ethyl acetate: hexane → 1:1 ethyl acetate: hexane → ethyl acetate → 19:1 ethyl acetate: methanol) afforded the *title compound* as an off white solid (113.5 mg, 71%); m.p. 134–136 °C; R_f 0.53 (9:1 ethyl acetate: methanol); ν_{max}/cm^{-1} (neat) 3318, 2934, 1634, 1536, 1502, 1489, 1443, 1370, 1244, 1176, 1100, 1037, 926, 808, 730, 578, 492; All ^1H signals broadened due to rotamer interconversion. δ_H (400 MHz, $CDCl_3$, 55 °C) 6.85–6.64 (3H, m, Ar**H**), 5.91 (2H, s, OC**H₂**O), 5.63 (1H, br s, N**H**), 4.59–4.38 (2H, s, NC**H₂**Ar), 3.98–3.71 (2H, s, NC**H₂**CO), 3.41–3.13 (2H, m, C**H₂**NH), 2.36–2.21 (2H, m, C**H₂**CON), 1.81–1.10 (8H, m, (C**H₂**)₄); δ_C (100 MHz, $CDCl_3$) 175.1 (**C**O), 169.7 (**C**O), 148.4 (Ar**CO**CH_2), 147.6 (Ar**CO**CH_2), 131.1 (Ar**C**), 122.1 (Ar**C**H), 109.0 (Ar**C**H), 108.7 (Ar**C**H), 101.3 (O**C**H_2O), 52.8

(N**C**H_2CO), 51.5 (N**C**H_2Ar), 39.2 (**C**H_2NH), 30.4 (**C**H_2CON), 29.0 (**C**H_2), 26.1 (**C**H_2), 24.5 (**C**H_2), 22.6 (**C**H_2); HRMS (ESI): calcd. for $C_{17}H_{22}N_2NaO_4$, 341.1472. Found: $[MNa]^+$, 341.1471 (0.1 ppm error).

4-(2-(1*H*-Indol-3-yl)ethyl)-1,4-diazacycloundecane-2,5-dione (27y)

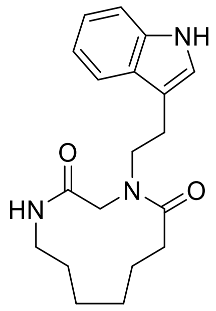

A mixture of 1-aza-2-cyclooctanone (64.4 mg, 0.506 mmol), DMAP (6.2 mg, 0.050 mmol) and pyridine (0.240 mL, 3.00 mmol) in DCM (5 mL) under an argon atmosphere was stirred at RT for 30 mins. Next, a solution of acid chloride **26r** (0.756 mmol, 1.50 eqv prepared using the general procedure) in DCM (5 mL) was added and the resulting mixture was refluxed at 50 °C for 18 h. The mixture was then diluted with DCM (30 mL) and washed with 10% aq. HCl (15 mL). The aqueous layer was then extracted with DCM (3 × 15 mL) and the combined organic extracts dried over $MgSO_4$ and concentrated *in vacuo*. The crude material was then re-dissolved in DCM (5 mL) and DBU (0.75 mL, 5.00 mmol) was added, followed by stirring at RT for 18 h, before the solvent was removed *in vacuo*. Purification by flash column chromatography (SiO_2, 1:9 ethyl acetate: hexane → 1:1 ethyl acetate: hexane → ethyl acetate → 19:1 ethyl acetate: methanol) afforded the *title compound* as an orange white solid (65.4 mg, 40%); m.p. 180–182 °C; R_f 0.33 (9:1 ethyl acetate: methanol); ν_{max}/cm^{-1} (neat) 3301, 2931, 1635, 1535, 1447, 1101, 908, 731, 646; ^{1}H signals broadened due to rotamer interconversion. δ_H (400 MHz, $CDCl_3$, 55 °C) 8.20 (1H, br s, ArN**H**), 7.68–7.04 (5H, m, Ar**H**), 5.47 (1H, br s, N**H**), 4.03–3.77 (2H, s, NC**H$_2$**CO), 3.55–3.26 (2H, m, C**H$_2$**NH), 3.09 (2H, t, J = 6.6 Hz, NCH$_2$**CH$_2$**Ar), 2.45–2.37 (2H, m, C**H$_2$**NH), 2.28–2.13 (2H, m, C**H$_2$**CON), 1.95–0.87 (8H, m, (C**H$_2$**)$_4$); δ_C (100 MHz, $CDCl_3$) 175.7 (**C**O), 169.7 (**C**O), 136.4 (Ar**C**), 127.3 (Ar**C**), 122.8 (ArCH), 122.6 (Ar**C**H), 119.9 (Ar**C**H), 118.6 (Ar**C**H), 111.6 (Ar**C**), 111.4 (Ar**C**H), 54.1 (N**C**H_2CO), 47.6 (N**C**H_2Ar), 39.0 (**C**H_2NH), 30.3 (**C**H_2CON), 29.0 (**C**H_2), 26.0 (**C**H_2), 24.7 (**C**H_2), 23.5 (Ar**C**H_2), 22.5 (**C**H_2); HRMS (ESI): calcd. for $C_{19}H_{25}N_3NaO_2$, 350.1839. Found: $[MNa]^+$, 350.1836 (1.0 ppm error).

Ethyl 4,13-dioxoazacyclotridecane-5-carboxylate (34)

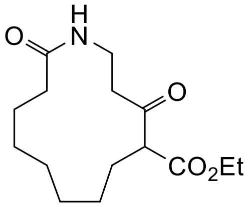

A mixture of ethyl 2-oxocyclononane-1-carboxylate (84 mg, 0.393 mmol), $MgCl_2$ (75 mg, 0.780 mmol) and pyridine (0.190 mL, 2.36 mmol) in CH_2Cl_2 (3 mL) under an argon atmosphere was stirred at RT for 30 mins. Next, a solution of acid chloride **29** (1.18 mmol, prepared using the general procedure) in CH_2Cl_2 (1 mL) was added and the reaction mixture was stirred for 2 h at RT. The mixture was then diluted with CH_2Cl_2 (20 mL) and washed with 10% aq. HCl (25 mL). The aqueous layer was extracted with CH_2Cl_2 (3 × 25 mL) and the combined organic extracts were dried over $MgSO_4$ and concentrated *in vacuo*. The crude material was then re-dissolved in CH_2Cl_2 (4 mL) and piperidine (0.390 mL, 3.93 mmol) was added. The resulting mixture was stirred for 2 h at RT, before the solvent was removed *in vacuo*. Purification by column chromatography (SiO_2, 5:1 hexane:ethyl acetate→ethyl acetate) afforded the *title compound* as a white solid (83.0 mg, 74%); m.p. 121–123 °C (chloroform); R_f 0.18 (1:1 hexane:ethyl acetate); ν_{max}/cm^{-1} (thin film) 3395, 3288, 3072, 2932, 2862, 1742, 1709, 1640, 1539; δ_H (400 MHz, $CDCl_3$) 6.38 (1H, br s, N**H**), 4.07 (2H, q, *J* = 6.9 Hz, OC**H₂**CH₃), 3.63–3.50 (1H, m, C**H**H′NH), 3.43–3.30 (2H, m, CH**H′**NH and C**H**CO₂Et), 2.95–2.74 (2H, m, C**H₂**COCH), 2.18–1.99 (2H, m, C**H₂**CONH), 1.88–1.71 (2H, m, C**H₂**CH), 1.67–0.96 (13H, m, 5 × C**H₂** and C**H₃**); δ_C (100 MHz, $CDCl_3$) 207.1 (**C**O), 173.7 (**C**O), 169.5 (**C**O), 61.3 (O**C**H₂), 58.3 (**C**HCO₂Et), 42.2 (**C**H₂COCH), 36.5 (**C**H₂CONH), 34.0 (**C**H₂NH), 27.6 (**C**H₂CH), 26.1 (**C**H₂), 25.6 (**C**H₂), 25.5 (**C**H₂), 24.4 (**C**H₂), 24.1 (**C**H₂), 14.1 (**C**H₃); HRMS (ESI): calcd. for $C_{15}H_{25}NNaO_4$, 306.1676. Found: $[MNa]^+$, 306.1679 (−1.1 ppm error).

Ethyl 4,14-dioxoazacyclotetradecane-5-carboxylate (35)

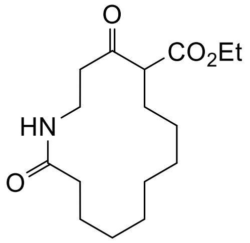

A mixture of ethyl 2-oxocyclodecane-1-carboxylate (89 mg, 0.393 mmol), $MgCl_2$ (75.0 mg, 0.780 mmol) and pyridine (0.190 mL, 2.36 mmol) in CH_2Cl_2 (3 mL) under an argon atmosphere was stirred at RT for 30 mins. Next, a solution of acid chloride **29** (1.18 mmol, prepared using the general procedure) in CH_2Cl_2 (1 mL) was added and the reaction mixture was stirred for 2 h at RT. The mixture was then diluted with CH_2Cl_2 (20 mL) and washed with 10% aq. HCl (25 mL). The aqueous layer was extracted with CH_2Cl_2 (3 × 25 mL) and the combined organic extracts were dried over $MgSO_4$ and concentrated *in vacuo*. The crude material was then re-dissolved in CH_2Cl_2 (4 mL) and piperidine (0.390 mL, 3.93 mmol) was added. The resulting mixture was stirred for 2 h at RT, before the solvent was removed *in vacuo*. Purification by column chromatography (SiO_2, 5:1 hexane:ethyl acetate→ethyl acetate) afforded the *title compound* as a colourless oil (60.0 mg, 52%); R_f 0.28 (1:1 hexane:ethyl acetate); ν_{max}/cm^{-1} (thin film) 3300, 3064, 2929, 2859, 1738, 1709, 1643, 1607; δ_H (400 MHz, $CDCl_3$) 6.14 (1H, br s, N**H**), 4.12 (2H, q, *J* = 6.9 Hz, OC**H₂**), 3.59–3.48 (2H, m, C**H₂**NH), 3.44 (1H, dd, *J* = 10.7, 3.8 Hz, C**H**CO_2Et), 2.91–2.66 (2H, m, C**H₂**COCH), 2.12 (2H, t, *J* = 6.1 Hz, C**H₂**CONH), 2.04–1.90 (1H, m, C**H**H′CH), 1.85–1.72 (1H, m, CH**H′**CH), 1.70–1.02 (15H, m, 6 × C**H₂** and CH_2C**H₃**); δ_C (100 MHz, $CDCl_3$) 206.6 (**C**O), 172.9 (**C**O), 169.6 (**C**O), 61.5 (O**C**H_2), 58.3 (**C**HCO_2Et), 41.5 (**C**H_2COCH), 34.7 (**C**H_2CONH), 33.3 (**C**H_2NH), 28.7 (**C**H_2CH), 26.7 (**C**H_2), 26.2 (**C**H_2), 25.3 (**C**H_2), 25.0 (**C**H_2), 24.8 (**C**H_2), 24.5 (**C**H_2), 14.2 (**C**H_3); HRMS (ESI): calcd. for $C_{16}H_{27}NNaO_4$, 320.1832. Found: $[MNa]^+$, 320.1826 (1.7 ppm error).

Ethyl 4,15-dioxoazacyclopentadecane-5-carboxylate (36)

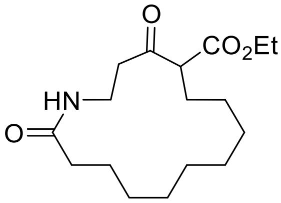

A mixture of ethyl 2-oxocycloundecane-1-carboxylate (94.0 mg, 0.393 mmol), $MgCl_2$ (75 mg, 0.780 mmol) and pyridine (0.190 mL, 2.36 mmol) in CH_2Cl_2 (3 mL) under an argon atmosphere was stirred at RT for 30 mins. Next, a solution of acid chloride **29** (1.18 mmol, prepared using the general procedure) in CH_2Cl_2 (1 mL) was added and the reaction mixture was stirred for 2 h at RT. The mixture was then diluted with CH_2Cl_2 (20 mL) and washed with 10% aq. HCl (25 mL). The aqueous layer was extracted with CH_2Cl_2 (3 × 25 mL) and the combined organic extracts were dried over $MgSO_4$ and concentrated *in vacuo*. The crude material was then re-dissolved in CH_2Cl_2 (4 mL) and piperidine (0.390 mL, 3.93 mmol) was added. The resulting mixture was stirred for 2 h at RT, before the solvent was removed *in vacuo*. Purification by column chromatography (SiO_2, 5:1 hexane:ethyl acetate → 1:1 hexane:ethyl acetate → ethyl acetate) afforded the *title compound* (as a 3:1 mixture of rotamers) as a colourless oil (79 mg, 65%); R_f 0.21 (1:1 hexane:ethyl acetate); ν_{max}/cm^{-1} (thin film) 3300, 2928, 2857, 1740, 1710, 1642, 1538; δ_H (400 MHz, $CDCl_3$) 6.20 (1H, br t, *J* = 5.5 Hz, N**H**, major rotamer), 6.12 (1H, br t, *J* = 5.3 Hz, N**H**, minor rotamer), 4.19–4.08 (4H, m, C**H**$_2$CH$_3$, both rotamers), 3.71–3.62 (1H, m, C**H**H′NH, major), 3.56–3.50 (1H, m, C**H**H′NH, minor), 3.45 (2H, dd, *J* = 10.3, 4.0 Hz, C**H**CO$_2$Et, both), 3.35–3.24 (2H, m, CH**H′**NH, both), 2.91–2.79 (4H, m, C**H**$_2$COCH, both), 2.37–1.93 (8H, m, 2 × C**H**$_2$, both), 1.87–1.05 (34H, m, 7 × C**H**$_2$ and C**H**$_3$, both); δ_C (100 MHz, $CDCl_3$) data for the major rotamer only: 206.7 (**C**O), 173.5 (**C**O), 169.6 (**C**O), 61.7 (**C**H$_2$CH$_3$), 58.0 (**C**HCO$_2$Et), 43.7 (**C**H$_2$NH), 36.2 (**C**H$_2$CO), 34.0 (**C**H$_2$CO), 28.5 (**C**H$_2$), 28.0 (**C**H$_2$), 27.6 (**C**H$_2$), 27.2 (**C**H$_2$), 26.9 (**C**H$_2$), 26.8 (**C**H$_2$), 26.6 (**C**H$_2$), 25.0 (**C**H$_3$); HRMS (ESI): calcd. for $C_{17}H_{29}NNaO_4$, 334.1989. Found: $[MNa]^+$, 334.1983 (1.8 ppm error).

Ethyl 4,9-dioxooxonane-5-carboxylate & Ethyl 4-hydroxy-9-oxo-2,3,6,7,8,9-hexahydrooxonine-5-carboxylate (40_{RE})

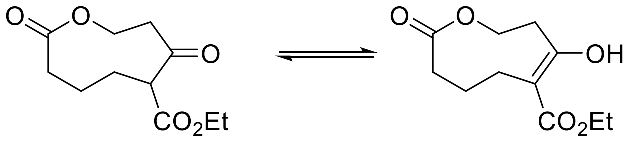

A mixture of ethyl 2-oxocyclopentane-1-carboxylate (61 mg, 0.393 mmol), $MgCl_2$ (75 mg, 0.780 mmol) and pyridine (0.190 mL, 2.36 mmol) in CH_2Cl_2 (3 mL) under an argon atmosphere was stirred at RT for 30 mins. Next, a solution of acid chloride **38** (1.18 mmol, prepared using the general procedure) in CH_2Cl_2 (1 mL) was added and the reaction mixture was stirred for 2 h at RT. The solvent was then concentrated *in vacuo*, loaded onto a short silica plug and eluted with ethyl acetate, to remove the majority of excess carboxylic acid and pyridine residues, and concentrated *in vacuo*. This material was re-dissolved in ethyl acetate (3.9 mL) and placed under an argon atmosphere. Palladium on carbon (39 mg, Pd 10% on carbon) was then added and the reaction vessel was backfilled with hydrogen (via balloon) several times, then stirred at RT under a slight positive pressure of hydrogen (balloon) for 18 h. The reaction was then purged with argon, filtered through Celite, washed with ethyl acetate and the solvent was removed *in vacuo*. The crude material was then re-dissolved in chloroform (3.9 mL) and triethylamine (82 µL, 0.590 mmol) added, and stirred at RT for 18 h, then reduced *in vacuo*. Purification by flash column chromatography (SiO_2, 2:1 hexane:ethyl acetate) afforded the *title compound* (as a 2:3 mixture of keto:enol tautomeric forms) as a colourless oil (53 mg, 59%); R_f 0.45 (1:1 hexane:ethyl acetate); ν_{max}/cm^{-1} (thin film) 2979, 1738, 1641, 1613; δ_H (400 MHz, $CDCl_3$) 12.81 (1H, s, O**H**, enol tautomer), 4.93–4.84 (1H, m, OC**H**H′CH_2, keto tautomer), 4.36–4.28 (1H, m, OCH**H′**CH_2, keto), 4.27–4.08 (6H, m, OC**H₂**CH_3, both tautomers and OC**H₂**CH_2, enol), 3.57 (1H, dd, *J* = 9.9, 4.0 Hz, C**H**CO_2Et, keto), 3.06–2.98 (1H, m, C**H**H′CO, keto), 2.90–2.81 (1H, m, CH**H′**CO, keto), 2.50–2.27 (6H, m, 3 × C**H₂**, both), 2.21–1.93 (8H, m, 4 × C**H₂**, both), 1.34–1.18 (6H, m, C**H₃**, both); δ_C (100 MHz, $CDCl_3$) 205.4 (**C**O), 175.2 (**C**O), 174.5 (**C**O), 173.1 (**C**O), 170.8 (**C**O), 169.1 (**C**O), 103.5 (**C**CO_2Et, enol), 61.7 (**C**H_2O), 61.5 (**C**H_2O), 60.8 (**C**H_2O), 60.6 (**C**H_2O), 57.8 (**C**HCO_2Et, keto), 41.2 (**C**H_2), 38.2 (**C**H_2), 34.8 (**C**H_2), 33.3 (**C**H_2), 27.5 (**C**H_2), 26.8 (**C**H_2), 23.4 (**C**H_2), 23.3 (**C**H_2), 14.4 (**C**H_3), 14.2 (**C**H_3); HRMS (ESI): calcd. for $C_{11}H_{16}NaO_5$, 251.0890. Found: $[MNa]^+$, 251.0886 (1.6 ppm error).

Ethyl 4,10-dioxooxecane-5-carboxylate & Ethyl (Z)-4-hydroxy-10-oxo-3,6,7,8,9,10-hexahydro-2H-oxecine-5-carboxylate (41_{RE})

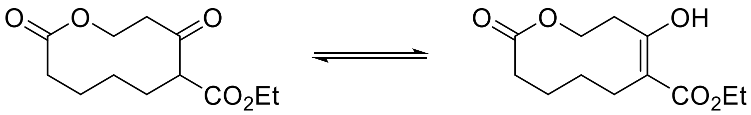

A mixture of ethyl 2-oxocyclohexane-1-carboxylate (67 mg, 0.393 mmol), $MgCl_2$ (75 mg, 0.780 mmol) and pyridine (0.190 mL, 2.36 mmol) in CH_2Cl_2 (3 mL) under an argon atmosphere was stirred at RT for 30 mins. Next, a solution of acid chloride **38** (1.18 mmol, prepared using the general procedure) in CH_2Cl_2 (3 mL) was added and the reaction mixture was stirred for 2 h at RT. The solvent was then concentrated *in vacuo*, loaded onto a short silica plug and eluted with 2:1 hexane:ethyl acetate, to remove the majority of excess carboxylic acid and pyridine residues, and concentrated *in vacuo*. This material was re-dissolved in ethyl acetate (3.9 mL) and placed under an argon atmosphere. Palladium on carbon (39 mg, Pd 10% on carbon) was then added and the reaction vessel was backfilled with hydrogen (via balloon) several times, then stirred at RT under a slight positive pressure of hydrogen (balloon) for 16 h. The reaction was then purged with argon, filtered through Celite, washed with ethyl acetate and the solvent was removed *in vacuo*. The crude material was then re-dissolved in chloroform (3.9 mL) and triethylamine (82.0 µL, 0.590 mmol) added, and stirred at RT for 16 h, then reduced *in vacuo*. Purification by flash column chromatography (SiO_2, 2:1 hexane:ethyl acetate → ethyl acetate) afforded the *title compound* (as a 3:2 mixture of keto:enol tautomeric forms) as a colourless oil (53 mg, 56%); R_f 0.49 (1:1 hexane:ethyl acetate); ν_{max}/cm^{-1} (thin film) 2943, 2874, 1732, 1715, 1642; δ_H (400 MHz, $CDCl_3$) 12.74 (1H, s, O**H**, enol), 4.72–4.62 (1H, m, C**H**H′O, keto), 4.32–4.07 (7H, m, CH**H′**O, keto, and C**H₂**O, enol and CH_3C**H₂**O, both), 3.68 (1H, dd, *J* = 9.9, 4.6 Hz, C**H**CO), 2.99–2.90 (1H, m, C**H**H′COCH, keto), 2.85–2.75 (1H, m, CH**H′**COCH, keto), 2.46–2.36 (1H, m, C**H**H′COO, keto), 2.34–2.10 (5H, m, CH**H′**COO, keto, and C**H₂**COO, enol and C**H₂**COH, enol), 2.02–1.33 (12H, m, 3 × C**H₂**, both), 1.29 (3H, t, *J* = 6.9 Hz, C**H₃**, enol), 1.24 (3H, t, *J* = 6.9 Hz, keto); δ_C (100 MHz, $CDCl_3$) 206.2 (**C**O, keto), 174.6 (**C**OH, enol), 173.2 (**C**O, enol), 173.0 (**C**O, keto), 170.7 (**C**O, enol), 169.4 (**C**O, keto), 102.5 (**C**), 61.53 (CH_3**C**H_2O, keto/enol), 61.49 (CH_3**C**H_2O, keto/enol), 61.0 (**C**H_2O, enol), 60.5 (**C**H_2O, keto), 58.3 (**C**H, keto), 39.8 (**C**H_2CO, keto), 35.9 (**C**H_2COO, enol), 34.6 (**C**H_2COO, keto), 31.1 (**C**H_2COH, enol), 27.9 (**C**H_2, enol), 26.6 (**C**H_2, keto), 24.5 (**C**H_2, enol), 24.0 (**C**H_2, keto), 23.3 (**C**H_2, enol), 22.1 (**C**H_2, keto), 14.3 (**C**H_3, enol, 14.2 (**C**H_3, keto); HRMS (ESI): calcd. for $C_{12}H_{18}NaO_5$, 265.1046. Found: $[MNa]^+$, 265.1035 (1.1 ppm error).

Ethyl 4,11-dioxooxacycloundecane-5-carboxylate (39)

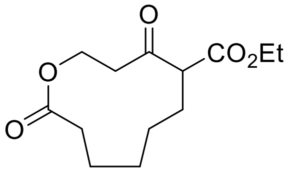

A mixture of ethyl 2-oxocycloheptane-1-carboxylate (72.0 mg, 0.393 mmol), $MgCl_2$ (75.0 mg, 0.780 mmol) and pyridine (0.190 mL, 2.36 mmol) in CH_2Cl_2 (3 mL) under an argon atmosphere was stirred at RT for 30 mins. Next, a solution of acid chloride **38** (1.18 mmol, prepared using the general procedure) in CH_2Cl_2 (1 mL) was added and the reaction mixture was stirred for 2 h at RT. The mixture was then loaded onto a short silica plug and eluted with 2:1 hexane:ethyl acetate to remove excess pyridine and carboxylic acid residues. The crude material was then dissolved in ethyl acetate (3.9 mL) and placed under an argon atmosphere. Palladium on carbon (39 mg, Pd 10% on carbon) was then added and the reaction vessel was backfilled with hydrogen (via balloon) several times, then stirred at RT under a slight positive pressure of hydrogen (balloon) for 3 h. The reaction was then purged with argon, filtered through Celite, washed with methanol before the solvent was removed *in vacuo*. The crude material was dissolved in chloroform (3.9 mL) and triethylamine (82.0 µL, 0.590 mmol) added and then stirred at RT for 18 h, where the solvent was removed *in vacuo*. Purification by flash column chromatography (2:1 hexane:ethyl acetate) afforded the *title compound* as a colourless oil (54.0 mg, 53%); R_f 0.55 (solvent); δ_H (400 MHz, $CDCl_3$) 4.68–4.62 (1H, m), 4.24–4.11 (3H, m), 3.43 (1H, dd, J = 12.0, 3.0 Hz), 3.09 (1H, ddd, J = 14.5, 10.1, 3.7 Hz), 2.58–2.52 (1H, m), 2.46–2.41 (1H, m), 2.18–2.04 (2H, m), 1.82–1.71 (4H, m), 1.53–1.47 (1H, m), 1.43–1.38 (1H, m), 1.30– 1.21 (4H, m). Data is consistent with those reported in the literature.[9]

Ethyl 4,12-dioxooxacyclododecane-5-carboxylate & Ethyl 4-hydroxy-12-oxooxacyclododec-4-ene-5-carboxylate (43)

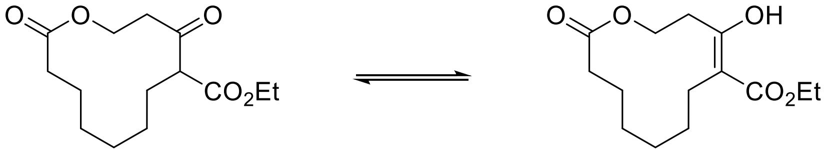

A mixture of ethyl 2-oxocyclooctane-1-carboxylate (78.0 mg, 0.393 mmol), $MgCl_2$ (75.0 mg, 0.780 mmol) and pyridine (0.190 mL, 2.36 mmol) in CH_2Cl_2 (3 mL) under an argon atmosphere

was stirred at RT for 30 mins. Next, a solution of acid chloride **38** (1.18 mmol, prepared using the general procedure) in CH_2Cl_2 (3 mL) was added and the reaction mixture was stirred for 2 h at RT. The solvent was then concentrated *in vacuo*, loaded onto a short silica plug and eluted with 2:1 hexane:ethyl acetate, to remove the majority of excess carboxylic acid and pyridine residues, and concentrated *in vacuo*. This material was re-dissolved in ethyl acetate (3.9 mL) and placed under an argon atmosphere. Palladium on carbon (39 mg, Pd 10% on carbon) was then added and the reaction vessel was backfilled with hydrogen (via balloon) several times, then stirred at RT under a slight positive pressure of hydrogen (balloon) for 16 h. The reaction was then purged with argon, filtered through Celite, washed with ethyl acetate and the solvent was removed *in vacuo*. The crude material was then re-dissolved in chloroform (3.9 mL) and triethylamine (82.0 µL, 0.590 mmol) added, and stirred at RT for 18 h, then reduced *in vacuo*. Purification by flash column chromatography (SiO_2, 2:1 hexane:ethyl acetate → ethyl acetate) afforded the *title compound* (as a 3:1 mixture of keto:enol tautomers) as a yellow oil (64 mg, 60%); R_f 0.76 (ethyl acetate); ν_{max}/cm^{-1} (thin film) 2928, 2857, 1745, 1706, 1642; δ_H (400 MHz, $CDCl_3$) 12.60 (1H, s, O**H**, enol tautomer), 4.28–4.09 (8H, m, 2 × C**H₂**O, both tautomers), 3.55 (1H, dd, *J* = 11.1, 5.0 Hz, C**H**CO_2Et, keto tautomer), 2.68–2.32 (8H, m, 2 × C**H₂**CO, both), 2.17–2.06 (4H, m, C**H₂**, both), 1.97–1.65 (8H, m, 2 × C**H₂**, both), 1.56–1.19 (14H, m, 2 × C**H₂** and C**H₃**, both); δ_C (100 MHz, $CDCl_3$) data for the ketone tautomer only: 212.2 (**C**O), 175.9 (**C**O), 170.4 (**C**O), 61.3 (**C**H_2O), 60.3 (**C**H_2O), 57.2 (**C**HCO_2Et), 41.8 (**C**H_2CO), 30.0 (**C**H_2CO), 29.1 (**C**H_2), 27.1 (**C**H_2), 25.6 (**C**H_2), 25.4 (**C**H_2), 24.7 (**C**H_2), 14.2 (**C**H_3); HRMS (ESI): calcd. for $C_{14}H_{22}NaO_5$, 293.1359. Found: $[MNa]^+$, 293.1357 (0.7 ppm error).

4-Benzyl-1,4,8-oxadiazecane-3,7-dione (53$_{RE}$)

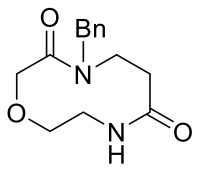

A mixture of morpholine-3-one (126 mg, 1.25 mmol), DMAP (23.2 mg, 0.188 mmol) and pyridine (0.604 mL, 7.50 mmol) in DCM (8.5 mL) under an argon atmosphere was stirred at RT for 30 mins. A solution of acid chloride **46** (1.88 mmol, 1.50 equiv., freshly prepared using the general procedure) in DCM (4.5 mL) was added and the resulting mixture was refluxed at 50 °C overnight. The mixture was then diluted with DCM (30 mL) and washed with 10% aq.

HCl (30 mL). The aqueous layer was then extracted with DCM (3 × 30 mL). The combined organic layers were dried over $MgSO_4$ and concentrated *in vacuo*. The crude material was then re-dissolved in DCM (12.6 mL) and DBU (1.87 mL, 12.5 mmol) was added, followed by stirring at RT overnight, before the solvent was removed *in vacuo*. Purification by flash column chromatography (SiO_2, ethyl acetate/hexane 1:1 → 9:1 ethyl acetate/MeOH) afforded the *title compound* a yellow oil (91.6 mg, 41%); R_f 0.62 (3:2 ethyl acetate : MeOH); ν_{max} (thin film)/cm^{-1} 3287, 3064, 2933, 2242, 1626, 2430, 1106, 729; Data for major rotamer only: δ_H (400 MHz, $CDCl_3$) 7.35−7.25 (4H, m, ArH), 7.17−7.15 (1H, m, ArH), 5.82 (1H, m, NH), 5.10 (1H, d, *J* = 16.9 Hz, NC**H**H-Ph), 4.51 (1H, d, *J* = 13.1 Hz, OC**H**H-CO), 4.34 (1H, d, *J* = 16.9 Hz, NCH**H**-Ph), 4.21−4.07 (3H, m, C**H**H-NBn and OC**H₂**), 3.85−3.82 (1H, m, C**H**HNH), 3.71 (1H, d, *J* = 13.1 Hz, OCH**H**-CO), 3.46−3.43 (1H, m, CH**H**NH), 3.14−3.06 (1H, m, CH**H**-NBn), 2.95−2.86 (1H, m, C**H**H-CO), 2.48−2.42 (1H, m, CH**H**-CO); δ_C (100 MHz, $CDCl_3$) 172.7 (CO-NBn), 171.5 (CO-NH), 136.9 (ArC), 128.9 (ArC), 128.8 (ArC), 128.2 (ArC), 127.6 (ArC), 126.5 (ArC), 73.8 (O**C**H_2-CO), 67.9 (OCH_2), 53.9 (NCH_2-Ph), 43.8 (CH_2-NBn), 41.4 (CH_2NH), 34.7 (**C**H_2-CO); Diagnostic ^{13}C NMR resonances for the minor rotamer: 168.9 (CO), 165.9 (CO), 72.7 (O**C**H_2-CO), 63.2 (OCH_2), 42.8, 41.2, 40.7, 30.5 (**C**H_2-CO); HRMS (ESI$^+$): calcd. for $C_{14}H_{18}N_2NaO_3$, 285.1210. Found: [MNa^+], 285.1211 (−0.5 ppm error).

4-(3-Hydroxypropanoyl)morpholin-3-one (54_{RO}) and 1,4,7-dioxazecane-2,8-dione (54_{RE})

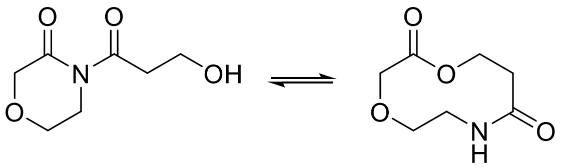

A mixture of morpholine-3-one (63.4 mg, 0.630 mmol), DMAP (23.2 mg, 0.188 mmol) and pyridine (0.604 mL, 7.50 mmol) in DCM (8.5 mL) under an argon atmosphere was stirred at RT for 30 mins. A solution of acid chloride **38** (1.88 mmol, 1.60 equiv., freshly prepared using the general procedure) in DCM (4.5 mL) was added and the resulting mixture was refluxed at 50 °C overnight. The mixture was then diluted with DCM (30 mL) and washed with 10% aq. HCl (30 mL). The aqueous layer was then extracted with DCM (3 × 30 mL). The combined organic layers were dried over $MgSO_4$ and concentrated *in vacuo*. The crude material was then re-dissolved in DCM (12.6 mL) and DBU (1.87 mL, 12.5 mmol) was added, followed by stirring at RT overnight. The solvent was removed *in vacuo* to give the crude mixture of the

title compounds in a ratio of 4:3. Purification by flash column chromatography (SiO_2, ethyl acetate/hexane 1:1 → 9:1 ethyl acetate/MeOH) afforded a mixture of the *title compounds* as a colourless oil (72.9 mg, 67%). Data for 4-(3-hydroxypropanoyl)morpholin-3-one: R_f 0.71 (4:1 ethyl acetate/MeOH); ν_{max} (thin film)/cm^{-1} 3328, 2914, 1662, 1349, 1125; δ_H (400 MHz, $CDCl_3$) 4.28 (2H, s, OCH_2), 3.95–3.91 (4H, m, C**H**$_2$OH and CH_2C**H**$_2$O), 3.80 (2H, m, C**H**$_2$NH), 3.23 (2H, t, J = 5.3 Hz, CH_2CO), 2.53 (1H, t, J = 6.5 Hz, OH); δ_C (100 MHz, $CDCl_3$) 175.3 (**C**O-CH_2-CH_2), 169.5 (**C**O-CH_2O), 68.9 (OCH_2), 63.9 (**C**H_2-CH_2-N), 58.1 (CH_2OH), 43.3 (CH_2-N), 42.3 (**C**H_2-CO-N); Diagnostic ^{1}H NMR resonances for 1,4,7-dioxazecane-2,8-dione: δ_H (400 MHz, $CDCl_3$) 5.92 (1H, m, NH), 4.57 (2H, br m, COO-CH_2), 4.05 (2H, br m, OCH_2), 3.63 (2H, br m, C**H**$_2$-CH_2-N), 3.48–3.45 (2H, m, CH_2N), 2.59 (2H, m, CH_2-CO); HRMS (ESI$^+$): calcd. for $C_7H_{11}NNaO_4$, 196.0580. Found: [MNa$^+$], 196.0582 (-0.9 ppm error).

8-Benzyl-1-oxa-4,8-diazacycloundecane-5,9-dione (56$_{RE}$)

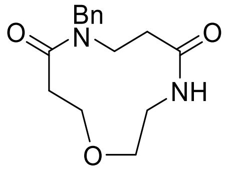

A mixture of 1,4-oxazepan-5-one (133 mg, 1.16 mmol), DMAP (21.3 mg, 0.174 mmol) and pyridine (0.560 mL, 6.96 mmol) in DCM (8.0 mL) under an argon atmosphere was stirred at RT for 30 mins. A solution of acid chloride **46** (1.74 mmol, 1.50 equiv., freshly prepared using the general procedure) in DCM (8.5 mL) was added and the resulting mixture was refluxed at 50 °C overnight. The mixture was then diluted with DCM (30 mL) and washed with 10% aq. HCl (30 mL). The aqueous layer was then extracted with DCM (3 × 20 mL). The combined organic layers were dried over $MgSO_4$ and concentrated *in vacuo*. The crude material was then re-dissolved in DCM (12 mL) and DBU (1.73 mL, 11.6 mmol) was added, followed by stirring at RT overnight, before the solvent was removed *in vacuo*. Purification by flash column chromatography (SiO_2, ethyl acetate/hexane 1:1 → 9:1 ethyl acetate/MeOH) afforded the *title compound* as a mixture of three rotamers in an approximately 1:1:1 ratio (224 mg, 70%); R_f 0.50 (9:1 ethyl acetate: methanol); ν_{max} (thin film)/cm^{-1} 3299, 3086, 2930, 1629, 1555, 1451; δ_H (400 MHz, $CDCl_3$) 7.31–7.12 (15H, m, ArH, all rotamers), 6.45–6.38 (2H, m, NH, rotamer A and B), 5.95–5.93 (1H, m, NH, rotamer C), 5.11–5.07 (2H, 2 × C**H**H-Ph, rotamer A and B), 4.92 (1H, d, J = 16.8 Hz, C**H**H-Ph, rotamer C), 4.45–4.33 (3H, m, C**H**H-NBn, OC**H**H and

CH**H**-Ph, rotamer C), 4.23−4.19 (2H, m, 2 × CH**H**-Ph, rotamer A and B), 4.02−3.91 (2H, m, C**H**H-NBn and OC**H**H-CH_2-NH), 3.84−3.72 (5H, m, OC**H**H, C**H**H-NH, OCH**H**-CH_2-NH and OC**H$_2$**-CH_2-NH), 3.67−3.59 (4H, m, 2 × OC**H$_2$**), 3.50−3.47 (2H, m, C**H$_2$**-NH), 3.39−3.24 (5H, m, OC**H$_2$**-CH_2-NH, 1 × CH_2-NBn and CH**H**-NBn) 3.02−2.65 (7H, m, 1 × CH_2-CO, C**H**H-CO, C**H$_2$**-NH, CH**H**-NBn and CH**H**-NH), 2.43−1.91 (9H, m, 4 × CH_2-CO and CH**H**-CO); δ_C (100 MHz, $CDCl_3$) 179.2 (CO), 177.6 (CO), 173.5 (CO), 171.7 (CO), 171.6 (CO), 171.5 (CO), 137.8 (ArC), 137.1 (ArC), 128.8 (ArC), 128.7 (ArC), 128.0 (ArC), 127.6 (ArC), 127.4 (ArC), 126.2 (ArC), 71.4 (O**C**H_2-CH_2-NH), 71.1 (O**C**H_2-CH_2-NH), 70.5 (O**C**H_2-CH_2-NH), 67.4 (OCH_2), 65.5 (OCH_2), 65.3 (OCH_2), 53.6 (CH_2-Ph), 48.6 (2 × CH_2-Ph), 44.8 (CH_2-NBn), 43.9 (CH_2-NBn), 42.8 (CH_2-NBn), 41.0 (CH_2-NH), 40.8 (CH_2-NH), 40.1 (CH_2-NH), 36.6 (**C**H_2-CO), 36.3 (**C**H_2-CO), 35.1 (**C**H_2-CO), 34.7 (**C**H_2-CO), 30.5 (**C**H_2-CO), 29.5 (**C**H_2-CO); HRMS (ESI^+): calcd. for $C_{15}H_{20}N_2NaO_3$, 299.1366. Found: [MNa^+], 299.1369 (−0.9 ppm error).

4-Benzyl-1,4,8-thiadiazecane-3,7-dione (59$_{RE}$)

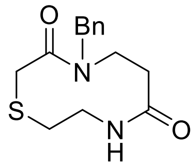

A mixture of thiomorpholin-3-one (75.6 mg, 0.645 mmol), DMAP (12.0 mg, 0.0900 mmol) and pyridine (0.310 mL, 3.90 mmol) in DCM (4.2 mL) under an argon atmosphere was stirred at RT for 30 mins. A solution of acid chloride **46** (0.968 mmol, 1.5 equiv., freshly prepared using the general procedure) in DCM (2.5 mL) was added and the resulting mixture was refluxed at 50 °C overnight. The mixture was then diluted with DCM (15 mL) and washed with 10% aq. HCl (15 mL). The aqueous layer was then extracted with DCM (3 × 15 mL). The combined organic layers were dried over $MgSO_4$ and concentrated *in vacuo*. The crude material was then re-dissolved in DCM (6.5 mL) and DBU (0.970 mL, 6.50 mmol) was added, followed by stirring at RT overnight, before the solvent was removed *in vacuo*. Purification by flash column chromatography (SiO_2, ethyl acetate/hexane 1:1 → 9:1 ethyl acetate:MeOH) afforded the *title compound* as a yellow oil (180 mg, 99%); R_f 0.66 (7:3 ethyl acetate:MeOH); ν_{max} (thin film)/cm^{-1} 3298, 3085, 2926, 1622, 1552, 1432, 1301, 1203, 730; Data for major rotamer only: δ_H (400 MHz, $CDCl_3$) 7.34−7.14 (5H, m, ArH), 6.12 (1H, br s, NH), 4.99 (1H, d, J = 16.8

Hz, NC**H**H-Ph), 4.47−4.40 (m, 1H, C**H**H-NBn), 4.38 (1H, d, J = 16.8 Hz, NCH**H**-Ph), 3.62−3.56 (1H, m, C**H**H-NH), 3.37−3.26 (2H, m, CO-C**H**HS and CH**H**-NH), 3.12 (1H, d, J = 13.0 Hz, CO-CH**H**S), 2.98−2.85 (3H, m, CH**H**-NBn and SC**H$_2$**-CH$_2$), 2.74−2.69 (1H, m, CH$_2$-C**H**H-CO), 2.52−2.45 (1H, m, CH$_2$-CH**H**-CO); δ_C (100 MHz, CDCl$_3$) 172.2 (CO-NBn), 170.6 (CO-NH), 136.2 (ArC), 128.9 (ArC), 128.8 (ArC), 128.1 (ArC), 127.8 (ArC), 126.3 (ArC), 52.7 (NCH$_2$-Ph), 42.4 (CH$_2$-NBn), 39.3 (CH$_2$-NH), 35.3 (CH$_2$-**C**H$_2$-CO), 34.5 (CO-**C**H$_2$S), 32.1 (S**C**H$_2$-CH$_2$); Diagnostic ^{13}C NMR resonances for the minor rotamer: 171.7 (CO), 171.3 (CO), 137.1 (ArC), 128.4 (ArC), 127.9 (ArC), 128.0 (ArC), 127.9 (ArC), 127.8 (ArC), 49.1, 45.2, 43.0, 36.8, 34.3, 30.5; HRMS (ESI$^+$): calcd. for $C_{14}H_{18}N_2NaO_2S$, 301.0981. Found: [MNa$^+$], 301.0981 (0.1 ppm error).

8-Benzyl-1-thia-4,8-diazacycloundecane-5,9-dione-(61$_{RE}$)

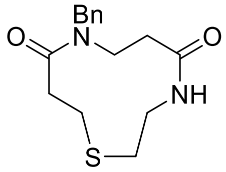

A mixture of 1,4-thiazepan-5-one (139 mg, 1.06 mmol), DMAP (19.4 mg, 0.160 mmol) and pyridine (0.510 mL, 6.36 mmol) in DCM (8.0 mL) under an argon atmosphere was stirred at RT for 30 mins. A solution of acid chloride **46** (1.59 mmol, 1.5 equiv., freshly prepared using the general procedure) in DCM (8.0 mL) was added and the resulting mixture was refluxed at 50 °C overnight. The mixture was then diluted with DCM (30 mL) and washed with 10% aq. HCl (30 mL). The aqueous layer was then extracted with DCM (3 × 20 mL). The combined organic layers were dried over MgSO$_4$ and concentrated *in vacuo*. The crude material was then re-dissolved in DCM (11 mL) and DBU (1.58 mL, 10.6 mmol) was added, followed by stirring at RT overnight, before the solvent was removed *in vacuo*. Purification by flash column chromatography (SiO$_2$, ethyl acetate/hexane 1:1 → 9:1 ethyl acetate/MeOH) afforded the *title compound* as a mixture of two rotamers in an approximately 5:1 ratio (228 mg, 73%); R$_f$ 0.71 (7:3 ethyl acetate: methanol); ν_{max} (thin film)/cm^{-1} 3299, 3086, 2924, 1628, 1553, 1427; Data for major rotamer only: δ_H (400 MHz, CDCl$_3$, 50 °C) 7.31−7.16 (5H, ArH), 6.30 (1H, br s, NH), 4.67 (2H, s, CH$_2$-Ph), 3.60−3.59 (2H, m, CH$_2$-NBn), 3.36−3.16 (2H, m, CH$_2$-NH), 2.85−2.80 (2H, m, CH$_2$S), 2.77−2.75 (2H, m, SC**H$_2$**-CH$_2$-NH), 2.57−2.54 (2H, m, C**H$_2$**-CH$_2$S), 2.21 (2H, t, J = 5.3 Hz, CH$_2$-CO); δ_C (100 MHz, CDCl$_3$) 171.7 (CO), 171.3 (CO), 171.3 (CO), 138.1 (ArC), 129.0 (ArC), 128.8 (ArC), 128.1 (ArC), 127.8 (ArC), 126.3 (ArC), 49.1 (CH$_2$-

Ph), 44.9 (CH_2-NBn), 40.6 (CH_2-NH), 36.6 (**C**H_2-CO), 31.1 (**C**H_2-CH_2S), 30.4 (S**C**H_2-CH_2-NH), 29.6 (CH_2S); **HRMS (ESI$^+$)**: calcd. for $C_{15}H_{20}N_2NaO_2S$, 315.1138. Found: [MNa^+], 315.1139 (-0.5 ppm error).

2-Benzyl-3,4,7,8-tetrahydro-2,6-benzodiazecine-1,5-(2*H*,6*H*)-dione (63$_{RE}$)

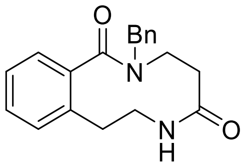

A mixture of 3,4-dihydroisoquinolin-1(2*H*)-one (100 mg, 0.680 mmol), DMAP (8.31 mg, 0.0680 mmol) and pyridine (0.330 mL, 4.08 mmol) in DCM (4.8 mL) under an argon atmosphere was stirred at RT for 30 mins. A solution of acid chloride **46** (1.02 mmol, 1.50 equiv., freshly prepared using the general procedure) in DCM (3.1 mL) was added and the resulting mixture was refluxed at 50 °C overnight. An additional solution of acid chloride (1.02 mmol, 1.50 equiv. prepared using the general procedure) in DCM (3.1 mL) was added and the reaction heated, at reflux at 50 °C for another 12 h to achieve reaction completion. The mixture was then diluted with DCM (30 mL) and washed with 10% aq. HCl (30 mL). The aqueous layer was then extracted with DCM (3 × 20 mL). The combined organic layers were dried over $MgSO_4$ and concentrated *in vacuo*. The crude material was then re-dissolved in DCM (6.9 mL) and DBU (1.04 mL, 6.97 mmol) was added, followed by stirring at RT overnight, before the solvent was removed *in vacuo*. Purification by flash column chromatography (SiO_2, ethyl acetate/hexane 4:1 → 98:2 ethyl acetate:MeOH) afforded the *title compound* as an oil (83.5 mg, 40%); R_f 0.46 (9:1 ethyl acetate: methanol); ν_{max}/cm^{-1} (neat) 3287, 3064, 2936, 2239, 1730, 1655, 1614, 1597, 1551, 1494, 1418 ; δ_H (400 MHz, $CDCl_3$) 7.39–7.24 (7H, m, ArH), 7.20 (1H, t, *J* = 8.0 Hz, ArH), 7.10–7.08 (1H, m, ArH), 5.75 (1H, s, NH), 5.44 (1H, d, *J* = 14.9 Hz, C**H**HPh), 4.13 (1H, d, *J* = 14.9 Hz, CH**H**Ph), 3.90–3.83 (1H, m, C**H**HNBn), 3.48–3.13 (4H, m, CH_2NH and CH**H**NBn and C**H**HCH_2NH), 2.62–2.58 (1H, m, CH**H**CH_2NH), 2.28–2.23 (1H, m, C**H**HCO), 1.97−1.93 (1H, m, CH**H**CO); δ_C (100 MHz, $CDCl_3$) 172.4 (CO), 171.6 (CO), 137.8 (ArC), 137.6 (ArC), 131.6 (ArC), 129.9 (ArC), 129.2 (ArC), 128.4 (ArC), 128.0 (ArC), 126.4 (ArC), 126.3 (ArC), 47.0 (CH_2Ph), 46.9 (CH_2NBn), 40.7 (CH_2NH), 36.2 (**C**H_2CO), 33.1 (**C**$H_2$$CH_2$NH). HRMS (ESI): calcd. for $C_{19}H_{20}N_2O_2$, 331.1417. Found; [MNa]$^+$, 331.1416 (1.0 ppm error).

2-(3-Hydroxypropanoyl)-3,4-dihydroisoquinolin-1(2*H*)-one (64$_{RO}$)

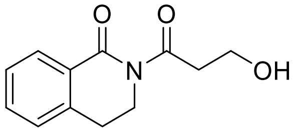

A mixture of 3,4-dihydroisoquinolin-1(2*H*)-one (100 mg, 0.680 mmol), DMAP (12.4 mg, 0.102 mmol) and pyridine (0.328 mL, 4.07 mmol) in DCM (4.5 mL) under an argon atmosphere was stirred at RT for 30 mins. A solution of acid chloride **38** (1.02 mmol, 1.5 equiv., freshly prepared using the general procedure) in DCM (3.2 mL) was added and the resulting mixture was refluxed at 50 °C overnight. Further two portions of acid chloride (1.02 mmol, 1.50 equiv. prepared using the general procedure) in DCM (3.1 mL) were added and the reaction heated, at reflux at 50 °C for another 2 × 12 h to achieve reaction completion. The solvent was concentrated *in vacuo*, loaded onto a short silica plug and eluted with 9:1 hexane:ethyl acetate → 4:1 hexane:ethyl acetate to remove the excess carboxylic acid and pyridine, and concentrated *in vacuo.* This material was re-dissolved in ethyl acetate (6.8 mL) and placed under an argon atmosphere. Palladium on carbon (68.0 mg, Pd 10% on carbon) was then added and the reaction vessel was backfilled with hydrogen (*via* balloon) several times, then stirred at RT under a slight positive pressure of hydrogen (balloon) for 16 h. The reaction was then purged with argon, filtered through Celite, washed with methanol and the solvent was removed *in vacuo*. The crude material was re-dissolved in chloroform (6.8 mL) and triethylamine (0.179 mL, 1.28 mmol) added, and stirred for 16 h. After removal of solvent under reduced pressure, the mixture was purified by flash column chromatography (SiO_2, 2:3 → 1:4 hexane:ethyl acetate) to afford the *title compound* as a colourless oil (68.3 mg, 68%); R_f (7:3 ethyl acetate:MeOH); ν_{max} (thin film)/cm^{-1} 3417, 1681, 1458, 1379, 1363, 1310, 1221; δ_H (400 MHz, $CDCl_3$) 8.11 (1H, dd, *J* = 7.9, 0.9 Hz, ArH), 7.50 (1H, dt, *J* = 7.5, 1.4 Hz, ArH), 7.36 (1H, t, *J* = 7.8 Hz, ArH), 7.25−7.22 (1H, m, ArH), 4.10 (2H, t, *J* = 6.4 Hz, CH_2N), 3.94 (2H, t, *J* = 5.3 Hz, CH_2OH), 3.29 (2H, t, *J* = 5.3 Hz, CO-CH_2), 2.98 (2H, t, *J* = 6.4 Hz, CH_2-CH_2N), 2.84 (1H, br s, OH); δ_C (100 MHz, $CDCl_3$) 176.2 (CO), 165.7 (CO), 140.1 (ArC), 133.5 (ArC), 129.5 (ArC), 128.8 (ArC), 127.4 (ArC), 127.3 (ArC), 58.6 (CH_2OH), 42.0 (**C**H_2-CO), 41.7 (CH_2N), 27.9 (CH_2-CH_2N); HRMS (ESI$^+$): calcd. for $C_{12}H_{13}NNaO_3$, 242.0788. Found: [MNa$^+$], 242.0787 (0.1 ppm error).

2-Benzyl-3,4,6,7,8,9-hexahydro-1*H*-benzo[*g*][1,5]diazacycloundecine-1,5(2*H*)-dione (66$_{\mathbf{RE}}$)

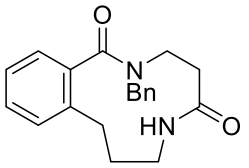

A mixture of 2,3,4,5-tetrahydro-1*H*-benzo[c]azepin-1-one (127 mg, 0.786 mmol), DMAP (10.0 mg, 0.0786 mmol) and pyridine (0.380 mL, 4.72 mmol) in DCM (5.5 mL) under an argon atmosphere was stirred at RT for 30 mins. A solution of acid chloride **46** (1.18 mmol, 1.50 equiv., freshly prepared using the general procedure) in DCM (3.0 mL) was added and the resulting mixture was refluxed at 50 °C overnight. The mixture was then diluted with DCM (30 mL) and washed with 10% aq. HCl (30 mL). The aqueous layer was then extracted with DCM (3 × 20 mL). The combined organic layers were dried over $MgSO_4$ and concentrated *in vacuo*. The crude material was then re-dissolved in DCM (8.0 mL) and DBU (1.20 mL, 7.86 mmol) was added, followed by stirring at RT overnight, before the solvent was removed *in vacuo*. Purification by flash column chromatography (SiO_2, 5:1 → 2:1 → 1:1 hexane:ethyl acetate) afforded the *title compound* as a yellow oil (189 mg, 77%), and as a mixture of two rotamers in an approximately 3:1 ratio. R_f 0.54 (98:2 ethyl acetate: methanol); ν_{max}/cm^{-1} (neat) 3299, 1646, 1615, 1549, 1495, 1443, 1421, 1351; Data for the major rotamers only: δ_H (400 MHz, $CDCl_3$, 50 °C) 7.40−7.05 (9H, m, ArH), 6.81 (1H, br m, NH), 4.50 (1H, d, *J* = 16.0 Hz, C**H**H-Ph), 4.38−4.28 (2H, m, CH**H**-Ph and C**H**H-NBn), 3.99−3.90 (1H, m, C**H**H-NH), 3.13−3.03 (2H, m, CH**H**-NBn and C**H**H-CO), 2.80−2.72 (2H, m, CH**H**-NH and CH_2-C**H**H-CH_2), 2.67−2.64 (1H, m, CH_2-CH**H**-CH_2), 2.58−2.52 (1H, m, CH**H**-CO), 1.86−1.60 (2H, m, C**H₂**-CH_2-CH_2); Data for major rotamer only: δ_c (100 MHz, $CDCl_3$) 172.9 (CO-NBn), 169.9 (CO-NH), 139.2 (ArC), 136.3 (ArC), 136.0 (ArC), 130.0 (ArC), 129.3 (ArC), 128.9 (ArC), 28.4 (ArC), 127.8 (ArC), 127.1 (ArC), 53.8 (CH_2-Ph), 41.0 (CH_2-NBn), 39.1 (CH_2-NH), 34.9 (**C**H_2-CO), 32.2 (**C**H_2-CH_2-CH_2), 29.1 (CH_2-**C**H_2-CH_2); Diagnostic ^{1}H NMR resonances for the minor rotamer: 5.59 (d, *J* = 14.7 Hz, C**H**H-Ph), 5.77 (1H, br m, NH), 4.19 (1H, d, *J* = 14.7 Hz, CH**H**-Ph), 3.85−3.81 (1H, m, C**H**H-NH), 3.22−3.15 (1H, m, CH**H**-NH); Diagnostic ^{13}C NMR resonances for the minor rotamer: 174.9 (CO), 171.6 (CO-NBn), 45.8 (CH_2-Ph), 39.0 (CH_2-NH); HRMS (ESI): calcd. for $C_{20}H_{22}N_2NaO_2$, 345.1573. Found; $[MNa]^+$ 345.1574 (-0.2 ppm error).

5-Benzyl-7,7-difluoro-1,5-diazecane-2,6-dione (69$_{RE}$)

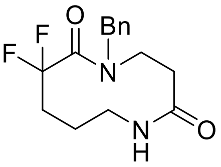

A mixture of 3,3-difluoropiperidin-2-one (52.8 mg, 0.390 mmol), DMAP (7.00 mg, 0.0590 mmol) and pyridine (0.188 mL, 2.34 mmol) in DCM (2.6 mL) under an argon atmosphere was stirred at RT for 30 mins. A solution of acid chloride **46** (0.590 mmol, 1.5 equiv., freshly prepared using the general procedure) in DCM (2.0 mL) was added and the resulting mixture was refluxed at 50 °C overnight. The mixture was then diluted with DCM (10 mL) and washed with 10% aq. HCl (10 mL). The aqueous layer was then extracted with DCM (3 × 10 mL). The combined organic layers were dried over $MgSO_4$ and concentrated *in vacuo*. The crude material was then re-dissolved in DCM (4.0 mL) and DBU (0.580 mL, 3.90 mmol) was added, followed by stirring at RT overnight, before the solvent was removed *in vacuo*. Purification by flash column chromatography (SiO_2, ethyl acetate/hexane 1:1 → 9:1 ethyl acetate/MeOH) afforded the *title compound* as a yellow oil (33.5 mg, 30%); R_f 0.63 (4:1 ethyl acetate:MeOH); ν_{max} (thin film)/cm^{-1} 3299, 2938, 1667, 1445, 1342, 1197, 1121, 988, 699; δ_H (400 MHz, $CDCl_3$) 7.38−7.22 (10H, m, ArH, both), 6.88 (1H, br s, NH, minor), 5.83 (1H, d, *J* = 10.7 Hz, NH, major), 5.11 (1H, d, *J* = 15.7 Hz, C**H**H-Ph, major), 4.23 (1H, d, *J* = 15.7 Hz, CH**H**-Ph, major), 4.07−4.01 (1H, m, C**H**H-NBn, major), 3.96−3.93 (1H, m, C**H**H-NH, major), 3.58−3.39 (3H, m, C**H$_2$**-Ph, minor, C**H**H-NBn minor and C**H$_2$**NH, minor), 3.23−3.19 (1H, m, CH**H**-NBn, minor), 3.12−3.04 (1H, m, CH**H**-NBn, major), 2.92−2.83 (2H, m, C**H**H-CO, major and CH**H**-NH, major), 2.53−2.42 (3H, m, CH**H**-CO, major, C**H$_2$**-CH_2-NH, major), 2.35−2.10 (3H, m, C**H$_2$**-CF_2, minor and C**H**H-CO, minor), 2.09−2.01 (2H, m, C**H$_2$**-CH_2-NH, minor), 1.90−1.83 (2H, m, C**H$_2$**-CH_2-NH, major), 1.71−1.63 (3H, m, C**H$_2$**-CF_2, major and CH**H**-CO, minor); δ_C (100 MHz, $CDCl_3$) 170.5 (CO-NH, major), 165.7 (CO-NBn, major), 165.1 (CO, minor), 163.7 (minor), 135.7 (ArC), 129.1 (ArC), 128.9 (ArC), 128.5 (ArC), 128.2 (ArC), 128.1 (ArC), 127.5 (ArC), 127.4 (ArC), 112.6 (m, CF_2), 54.1 (CH_2-Ph, major), 46.6 (CH_2-Ph, minor), 43.7 (CH_2-NBn, major), 42.9 (CH_2-NBn, minor), 41.9 (CH_2-NH, minor), 39.3 (CH_2-NH, major), 34.4 (**C**H_2-CO, major), 31.9 (t, *J* = 23.0 Hz, **C**H_2-CF_2, minor), 30.7 (**C**H_2-CO, minor), 29.8 (m, **C**H_2-CF_2, major), 22.3 (t, *J* = 3.4 Hz, **C**H_2-CH_2-NH, major), 19.6 (t, *J* = 5.3 Hz, **C**H_2-CH_2-NH, minor); δ_F −101.3 (t, *J* = 14.5 Hz, 1F, minor), −102.1

(dt, J = 14.5, 2.9 Hz, 1F, major); HRMS (ESI$^+$): calcd. for $C_{15}H_{18}F_2N_2NaO_2$, 319.1229. Found: [MNa$^+$], 319.1229 (−0.1 ppm error).

10-Allyl-5-benzyl-1,5-diazecane-2,6-dione (71$_{RE}$)

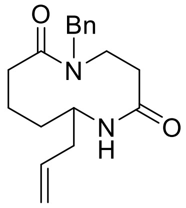

A mixture of (S)-6-allylpiperidin-2-one (100 mg, 0.720 mmol), DMAP (13.2 mg, 0.110 mmol) and pyridine (0.350 mL, 4.32 mmol) in DCM (4.8 mL) under an argon atmosphere was stirred at RT for 30 mins. A solution of acid chloride **46** (1.08 mmol, 1.5 equiv., freshly prepared using the general procedure) in DCM (4.5 mL) was added and the resulting mixture was refluxed at 50 °C overnight. The mixture was then diluted with DCM (30 mL) and washed with 10% aq. HCl (30 mL). The aqueous layer was then extracted with DCM (3 × 20 mL). The combined organic layers were dried over $MgSO_4$ and concentrated *in vacuo*. The crude material was then re-dissolved in DCM (7.5 mL) and DBU (1.10 mL, 7.20 mmol) was added, followed by stirring at RT overnight, before the solvent was removed *in vacuo*. Purification by flash column chromatography (SiO_2, ethyl acetate/hexane 1:1 → 9:1 ethyl acetate/MeOH) afforded the *title compound* as a colourless oil (181.4 mg, 84%); R_f 0.71 (4:1 ethyl acetate: methanol); ν_{max} (thin film)/cm^{-1} 3283, 3073, 2933, 1616, 1553, 1449, 1422, 914, 728, 699; δ_H (400 MHz, $CDCl_3$) 7.32−7.23 (5H, m, ArH), 5.73−5.62 (1H, m, CH_2=C**H**), 5.14 (1H, d, J = 9.9 Hz, NH), 5.03−4.96 (2H, m, C**H₂**=CH), 4.73 (1H, d, J = 14.1 Hz, C**H**H-Ph), 4.50 (1H, d, J = 14.1 Hz, CH**H**-Ph), 3.99−3.87 (2H, m, C**H**H-NBn and C**H**-NH), 3.26 (1H, dt, J = 15.3, 3.8 Hz, CH**H**-NBn), 2.67−2.59 (1H, m, CH_2-C**H**H-CH_2), 2.21−2.05 (5H, m, CH_2-CH**H**-CH_2, CH_2=CH-C**H₂**, C**H**H-CO-NH and C**H**H-CH-NH), 1.99−1.92 (1H, m, CH**H**-CO-NH), 1.66−1.55 (2H, m, CH**H**-CH-NH and C**H**H-CO-NBn), 1.39 (1H, m, CH**H**-CO-NBn); δ_C (100 MHz, $CDCl_3$) 173.8 (CO-NBn), 170.3 (CO-NH), 138.3 (ArC), 133.9 (CH_2=**C**H), 128.9 (ArC), 127.9 (ArC), 128.0 (ArC), 117.9 (**C**H_2=CH), 49.8 (CH_2-Ph), 49.1 (CH-NH), 45.6 (CH_2-NBn), 40.9 (CH_2=CH-**C**H_2), 37.7 (**C**H_2-CO-NH), 30.9 (**C**H_2-CO-NBn), 27.8 (CH_2-**C**H_2-CH_2), 23.2 (**C**H_2-CH-NH); HRMS (ESI$^+$): calcd. for $C_{18}H_{24}N_2NaO_2$, 323.1730. Found: [MNa$^+$], 323.1730 (0.1 ppm error).

6-Allyl-1,5-oxazecane-4,10-dione (72_{RE})

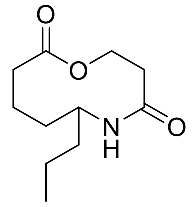

A mixture of (S)-6-allylpiperidin-2-one (120 mg, 0.860 mmol), DMAP (10.5 mg, 0.086 mmol) and pyridine (0.420 mL, 5.16 mmol) in DCM (6.0 mL) under an argon atmosphere was stirred at RT for 30 mins. A solution of acid chloride (1.29 mmol, 1.5 equiv., freshly prepared using the general procedure) in DCM (4.5 mL) was added and the resulting mixture was refluxed at 50 °C overnight. An additional solution of acid chloride **38** (1.29 mmol, 1.50 equiv. prepared using the general procedure) in DCM (3.1 mL) was added and the reaction heated, at reflux at 50 °C for another 12 h to achieve reaction completion. The solvent was concentrated *in vacuo*, loaded onto a short silica plug and eluted with 7:3 → 6:4 hexane:ethyl acetate to remove the excess carboxylic acid and pyridine, and concentrated *in vacuo* to give the *N*-acylated intermediate (57.4 mg, 67%). This material was re-dissolved in ethyl acetate (6.0 mL) and placed under an argon atmosphere. Palladium on carbon (57.4 mg, Pd 10% on carbon) was then added and the reaction vessel was backfilled with hydrogen (*via* balloon) several times, then stirred at RT under a slight positive pressure of hydrogen (balloon) for 16 h. The reaction was then purged with argon, filtered through Celite, washed with methanol and the solvent was removed *in vacuo*. The crude material was re-dissolved in chloroform (6.0 mL) and triethylamine (0.179 mL, 1.29 mmol) added, and stirred for 16 h. After removal of solvent under reduced pressure, the mixture was purified by flash column chromatography (SiO_2, 2:3 → 1:4 hexane:ethyl acetate) to afford the *title compound* as a colourless oil and as a mixture of two rotamers in an approximately 2:1 ratio (81.7 mg, 67% from the isolated acylated product); m.p. 127−130 °C; ν_{max} (thin film)/cm^{-1} 3285, 2955, 2931, 1732, 1648, 1549; δ_H (400 MHz, $CDCl_3$) 5.87 (1H, d, *J* = 11.4 Hz, NH, minor), 5.26 (1H, d, *J* = 9.2 Hz, NH, major), 4.82−4.78 (1H, m, OC**H**H, minor), 4.55−4.48 (1H, m, OC**H**H, major), 4.43−4.38 (1H, m, OCH**H**, major), 4.13−4.06 (1H, m, OCH**H**, minor), 3.89−3.86 (1H, m, C**H**-NH, major), 3.37−3.28 (1H, m, C**H**-NH, minor), 3.05−2.97 (1H, OCH_2-C**H**H, minor), 2.72−2.67 (1H, m, OCH_2-C**H**H, major), 2.58−2.50 (1H, C**H**H-CO, minor), 2.50−2.44 (1H, C**H**H-CO, major), 2.38 (1H, ddd, *J* = 12.2, 6.1, 1.5 Hz, OCH_2-CH**H**, major), 2.17−2.11 (5H, m, CH**H**-CO, both, and OCH_2-CH**H**, minor, C**H₂**-CH_2-CO, minor), 1.92−1.81 (3H, m, C**H₂**-CH_2-CO, major and C**H**H-CH-NH, major),

1.68−1.55 (2H, m, C**H**$_2$-CH-NH, minor), 1.41−1.22 (9H, m, CH$_3$-C**H**$_2$-C**H**$_2$ both, and CH**H**-CH-NH, major), 0.89−0.85 (6H, m, CH$_3$, both); δ_C (100 MHz, CDCl$_3$) 174.7 (COO, major), 174.3 (COO, minor), 172.8 (CO-NH, minor), 169.3 (CO-NH, major), 60.7 (OCH$_2$, major), 60.6 (OCH$_2$, minor), 51.6 (CH-NH, minor), 50.3 (CH-NH, major), 40.5 (CH$_3$-CH$_2$-**C**H$_2$, minor), 37.7 (CH$_3$-CH$_2$-**C**H$_2$, major), 36.9 (OCH$_2$-**C**H$_2$, major), 36.5 (**C**H$_2$-CO, major), 36.2 (**C**H$_2$-CH-NH, minor), 35.2 (**C**H$_2$-CO, minor), 33.9 (**C**H$_2$-CH$_2$-NH, major), 31.3 (OCH$_2$-**C**H$_2$, minor), 22.8 (**C**H$_2$-CH$_2$-CO, major), 21.4 (**C**H$_2$-CH$_2$-CO, minor), 19.1 (CH$_3$-**C**H$_2$, major), 18.9 (CH$_3$-**C**H$_2$, minor), 13.8 (CH$_3$, both); HRMS (ESI$^+$): calcd. for $C_{11}H_{19}NNaO_3$, 236.1257. Found: [MNa$^+$], 236.1256 (0.6 ppm error).

11-Benzyl-7-methyl-1-thia-4,7,11-triazacyclotetradecane-5,8,12-trione (78)

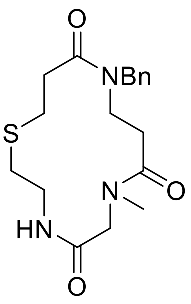

A mixture of 8-benzyl-1-thia-4,8-diazacycloundecane-5,9-dione (65.1 mg, 0.220 mmol), DMAP (2.8 mg, 0.022 mmol) and pyridine (0.110 mL, 1.32 mmol) in DCM (1.6 mL) under an argon atmosphere was stirred at RT for 30 mins. A solution of acid chloride **15** (0.660 mmol, 3.0 equiv., freshly prepared using the general procedure) in DCM (0.9 mL) was added and the resulting mixture was refluxed at 50 °C overnight. An additional solution of acid chloride (0.660 mmol, 3.0 equiv. prepared using the general procedure) in DCM (0.9 mL) was added and the reaction heated, at reflux at 50 °C for another 12 h to achieve reaction completion. The mixture was then diluted with DCM (30 mL) and washed with 10% aq. HCl (30 mL). The aqueous layer was then extracted with DCM (3 × 20 mL). The combined organic layers were dried over $MgSO_4$ and concentrated *in vacuo*. The crude material was then re-dissolved in DCM (2.2 mL) and DBU (0.330 mL, 2.20 mmol) was added, followed by stirring at RT overnight, before the solvent was removed *in vacuo*. Purification by flash column chromatography (SiO_2, ethyl acetate/hexane 9:1 → 9:1 ethyl acetate/MeOH) afforded the *title compound* as a yellow solid (48.4 mg, 61%), and as a mixture of two rotamers in an approximately 3:1 ratio. R_f 0.39 (4:1 ethyl acetate: methanol); m.p. 135−138 °C; ν_{max} (thin

film)/cm^{-1} 3313, 2928, 2471, 1630, 1535, 1478, 1449, 1363, 1279, 1208; δ_H (400 MHz, $CDCl_3$) 7.45 (1H, t, *J* = 5.3 Hz, NH, major), 7.39−7.14 (10H, m, ArH, both rotamers), 6.85 (1H, t, *J* = 5.0 Hz, NH, minor), 4.85 (2H, s, CH_2-Ph, minor), 4.67 (2H, s, CH_2-Ph, major), 3.95−3.63 (8H, m, C**H₂**-NCH_3 and CH_2-NBn, both), 3.56−3.52 (m, 2H, C**H**$_2$-NH, major), 3.50−3.46 (m, 2H, C**H₂**-NH, minor), 3.10 (3H, s, NCH_3, major), 3.00 (3H, s, NCH_3, minor), 2.86−2.75 (8H, m, 2 × SCH_2, both), 2.62−2.42 (8H, m, 2 × C**H₂**-CO, both); δ_C (100 MHz, $CDCl_3$) 173.0 (CO, major), 172.6 (CO, minor), 172.0 (CO, minor), 171.5 (CO, major), 168.5 (CO, major), 167.4 (CO, minor), 137.6 (ArC, minor), 136.0 (ArC, major), 129.0 (ArC, major), 128.8 (ArC, minor), 128.3 (ArC, minor), 128.1 (ArC, minor), 127.9 (ArC, major), 127.7 (ArC, minor), 127.3 (ArC, minor), 126.4 (ArC, major), 126.1 (ArC, major), 54.9 (**C**H_2-Ph, minor), 53.9 (CH_2-NCH_3, minor), 52.1 (CH_2-NCH_3, major), 51.8 (**C**H_2-Ph, major), 45.2 (CH_2-NBn, minor), 43.8 (CH_2-NBn, major), 36.8 (NCH_3, major), 35.6 (CH_2-NH, minor), 35.3 (CH_2-NH, major), 35.1 (NCH_3,minor), 33.2 (S**C**H_2-CH_2-NH, minor), 32.9 (**C**H_2-CO, minor), 32.4 (**C**H_2-CO, major), 32.2 (**C**H_2-CO, major), 32.1 (S**C**H_2-CO, minor), 31.9 (S**C**H_2-CH_2-NH, major), 26.8 (S**C**H_2-CH_2-CO, minor), 23.9 (S**C**H_2-CH_2-CO, major); **HRMS (ESI$^+$)**: calcd. for $C_{18}H_{25}N_3NaO_3S$ 386.1509. Found: [MNa^+], 386.1510 (−0.3 ppm error).

11-Benzyl-1,7-dioxa-4,11-diazacyclotetradecane-3,10,14-trione (79)

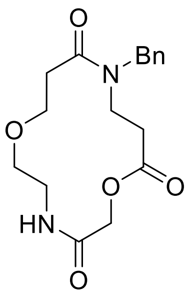

A mixture of 8-benzyl-1-oxa-4,8-diazacycloundecane-5,9-dione (80.0 mg, 0.290 mmol) was diluted in dry THF (2.7 mL) and *i*PrMgCl (0.220 mL) was added dropwise at 0 °C. Next, a solution of acid chloride **S39** (0.435 mmol, 1.5 eq., freshly prepared from acid using the general procedure) in dry THF (1.4 mL) was added and the resulting mixture was stirred at RT overnight. The mixture was then diluted with EtOAc (~30 mL) and washed with $NaHCO_3$ (15 mL). The aqueous layer was then extracted with EtOAc (2 × 20 mL) and the combined organic layer extracts dried over $MgSO_4$. The solvent was concentrated *in vacuo*, loaded onto a short silica plug and eluted with 6:4 → 3:7 hexane:ethyl acetate to remove the excess carboxylic

acid and pyridine, and concentrated *in vacuo*. This material was then re-dissolved in THF (2.4 mL) and water (0.44 mL), and placed under an argon atmosphere. Palladium on carbon (24.0 mg, Pd 10% on carbon) was then added and the reaction vessel was backfilled with hydrogen (via balloon) several times, then stirred at RT under a slight positive pressure of hydrogen (balloon) for 16 h. The reaction was then purged with argon, filtered through Celite, washed with methanol and concentrated *in vacuo*. This material was re-dissolved in chloroform (2.4 mL) and triethylamine (48.0 µL, 0.350 mmol) added, and stirred at RT for 16 h, then reduced *in vacuo*. Purification by flash column chromatography (SiO_2, ethyl acetate → 9:1 ethyl acetate: methanol) afforded the *title compound* as an yellow oil (45.2 mg, 47%); R_f 0.41 (9:1 ethyl acetate: methanol); ν_{max}/cm^{-1} (thin film) 3328, 2937, 1745, 1674, 1631, 1541, 1429; δ_H (400 MHz, $CDCl_3$) 7.37–7.26 (3H, m, ArH), 7.13 (2H, d, *J* = 6.9 Hz, ArH), 6.84 (1H, s, NH), 4.69–4.64 (4H, m, C**H**$_2$Ph and OC**H**$_2$CO), 3.78 (2H, t, *J* = 5.3 Hz, OC**H**$_2$CH$_2$NH), 3.75–3.72 (2H, m, COCH$_2$C**H**$_2$O), 3.60 (2H, t, *J* = 5.3 Hz, NCH$_2$), 3.48–3.44 (2H, m, NHC**H**$_2$), 2.76–2.73 (2H, m, COC**H**$_2$CH$_2$O), 2.55 (2H, t, *J* = 5.3 Hz, COC**H**$_2$CH$_2$N); δ_C (100 MHz, $CDCl_3$) 173.3 (CO), 171.3 (CO), 167.4 (CO), 136.5 (ArC), 129.1 (2 × ArC), 127.7 (ArC), 126.3 (2 × ArC), 68.2 (O**C**H$_2$CO), 66.3 (O**C**H$_2$), 62.9 (OCH$_2$), 53.0 (CH$_2$Ph), 44.1 (CH$_2$NBn), 38.3 (CH$_2$NH), 33.8 (2 peaks, 2 × CH$_2$CO). HRMS (ESI): calcd. for $C_{17}H_{22}N_2O_5$, 357.1421. Found; $[MNa]^+$, 357.1418 (0.8 ppm).

11-Benzyl-7-methyl-1-oxa-4,7,11-triazacyclotetradecane-5,8,12-trione (80)

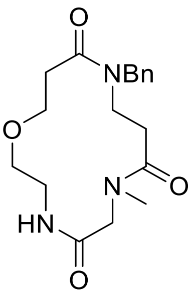

A mixture of 8-benzyl-1-oxa-4,8-diazacycloundecane-5,9-dione (76.2 mg, 0.280 mmol), DMAP (5.1 mg, 0.0420 mmol) and pyridine (0.140 mL, 1.68 mmol) in DCM (2.0 mL) under an argon atmosphere was stirred at RT for 30 mins. A solution of acid chloride **15** (0.410 mmol, 1.5 equiv., freshly prepared using the general procedure) in DCM (1.8 mL) was added and the resulting mixture was refluxed at 50 °C overnight. The mixture was then diluted with DCM (30 mL) and washed with 10% aq. HCl (30 mL). The aqueous layer was then extracted with

DCM (3 × 20 mL). The combined organic layers were dried over $MgSO_4$ and concentrated *in vacuo*. The crude material was then re-dissolved in DCM (3.0 mL) and DBU (0.420 mL, 2.80 mmol) was added, followed by stirring at RT overnight, before the solvent was removed *in vacuo*. Purification by flash column chromatography (SiO_2, ethyl acetate/hexane 3:2 → 4:1 ethyl acetate/MeOH) afforded the *title compound* as a colourless oil, and as a mixture of two rotamers in an approximately 2:1 ratio (48.4 mg, 70%); ν_{max} (thin film)/cm^{-1} 3314, 2930, 1630, 1531, 1125, 800, 731, 698; δ_H (400 MHz, CD_3OD) 7.40−7.22 (10H, m, ArH, both), 4.80 (2H, s, CH_2-Ph, major), 4.73 (2H, s, CH_2-Ph, minor), 4.05−3.97 (4H, m, C$\mathbf{H_2}$-NCH_3, both), 3.83−3.73 (8H, m, CH_2-NBn and OCH_2, both), 3.58−3.50 (4H, OCH_2, both), 3.44−3.42 (4H, m, C$\mathbf{H_2}$-NH, both), 3.15 (3H, NCH_3, major), 3.08 (3H, NCH_3, minor), 2.77−2.56 (8H, m, 2 × **C**H_2-CO, both); δ_C (100 MHz, $CDCl_3$) 175.7 (CO), 175.3 (CO), 174.8 (CO), 174.7 (CO), 171.1 (CO), 170.9 (CO), 139.2 (CO), 138.5 (CO), 130.2 (ArC), 130.0 (ArC), 129.8 (ArC), 129.0 (ArC), 128.8 (ArC), 128.6 (ArC), 127.9 (ArC), 127.7 (ArC), 70.7 (OCH_2), 69.1 (OCH_2), 69.0 (OCH_2), 66.1 (OCH_2), 54.3 (CH_2-NCH_3), 54.2 (CH_2-NCH_3), 53.5 (CH_2-Ph, minor), 53.3 (CH_2-Ph, major), 44.8 (CH_2-NBn), 43.9 (CH_2-NBn), 40.4 (CH_2-NH), 39.2 (CH_2-NH), 37.5 (NCH_3), 36.6 (NCH_3), 34.9 (**C**H_2-CO), 34.5 (**C**H_2-CO), 33.5 (**C**H_2-CO), 33.3 (**C**H_2-CO); HRMS (ESI): calcd. for $C_{18}H_{25}N_3NaO_4$, 370.1737. Found; $[MNa]^+$, 370.1732 (1.5 ppm).

Compound 60

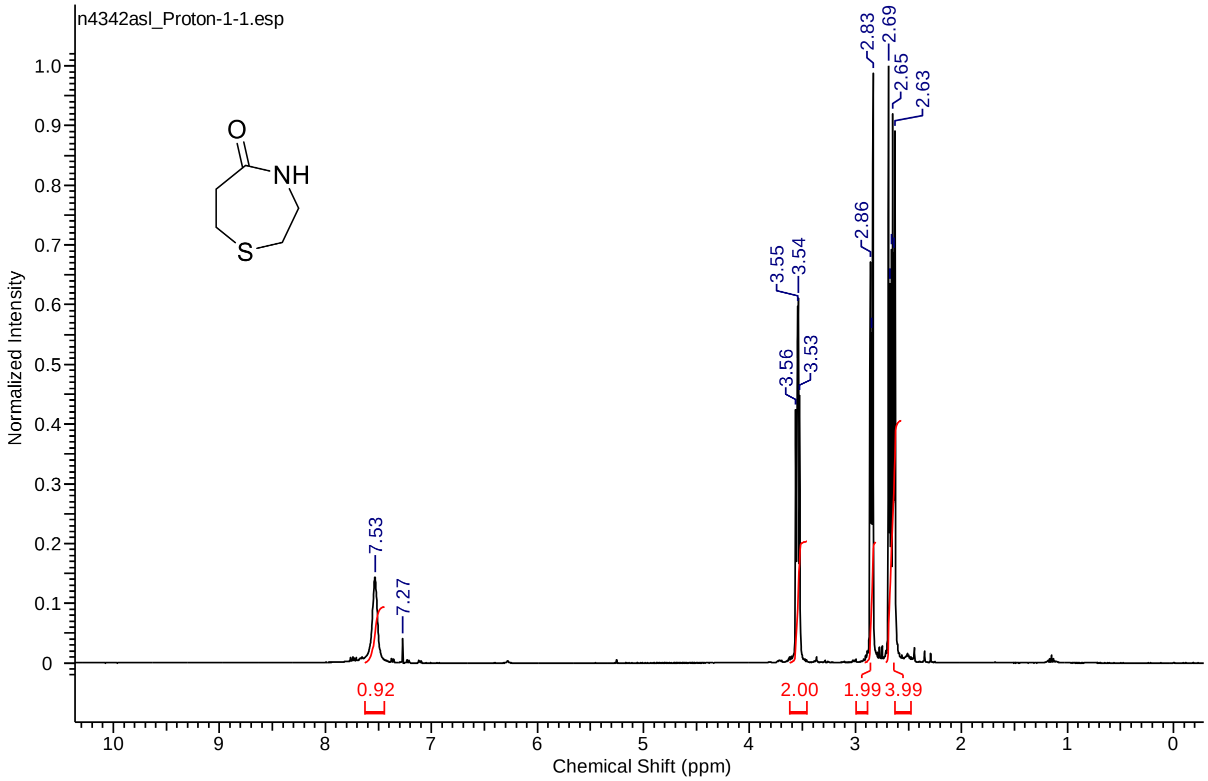
n4342asl_Proton-1-1.esp
O
NH
S
Normalized Intensity
Chemical Shift (ppm)
7.53
7.27
3.56
3.55
3.54
3.53
2.86
2.83
2.69
2.65
2.63
0.92
2.00
1.99
3.99

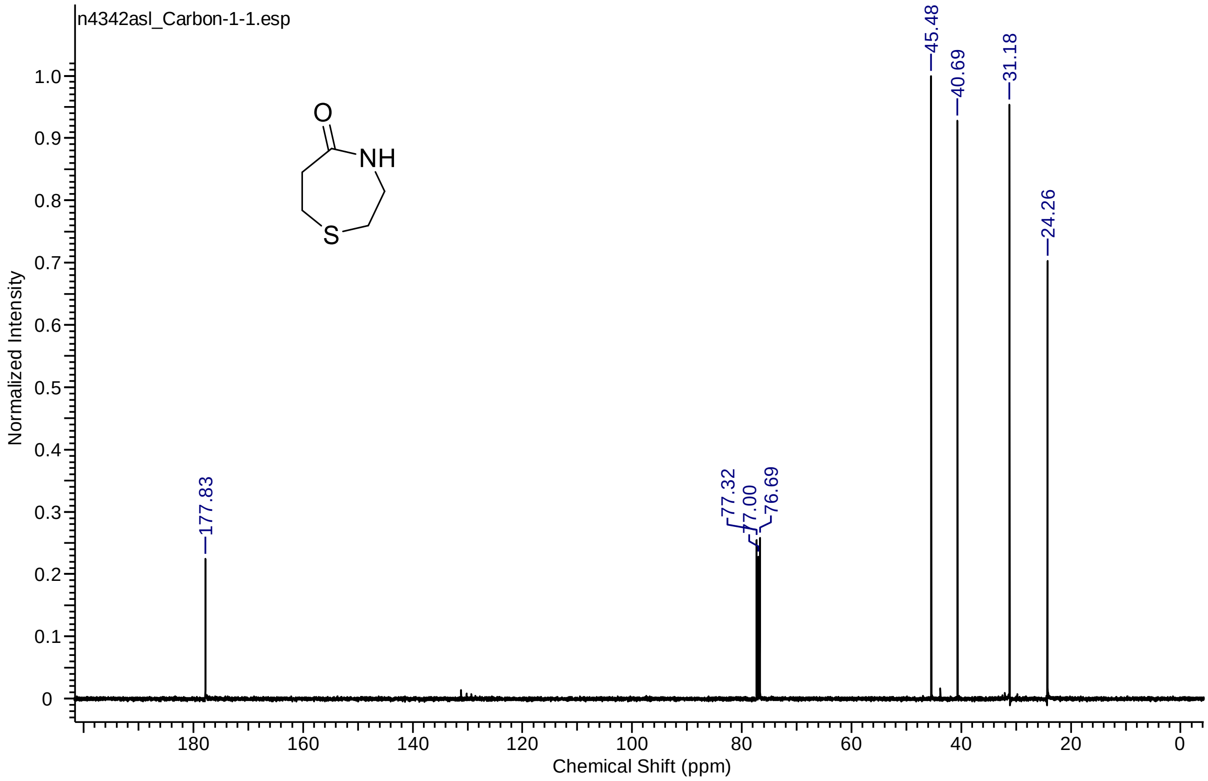
n4342asl_Carbon-1-1.esp
O
NH
S
Normalized Intensity
Chemical Shift (ppm)
177.83
77.32
77.00
76.69
45.48
40.69
31.18
24.26

Compound S14

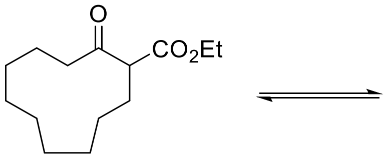

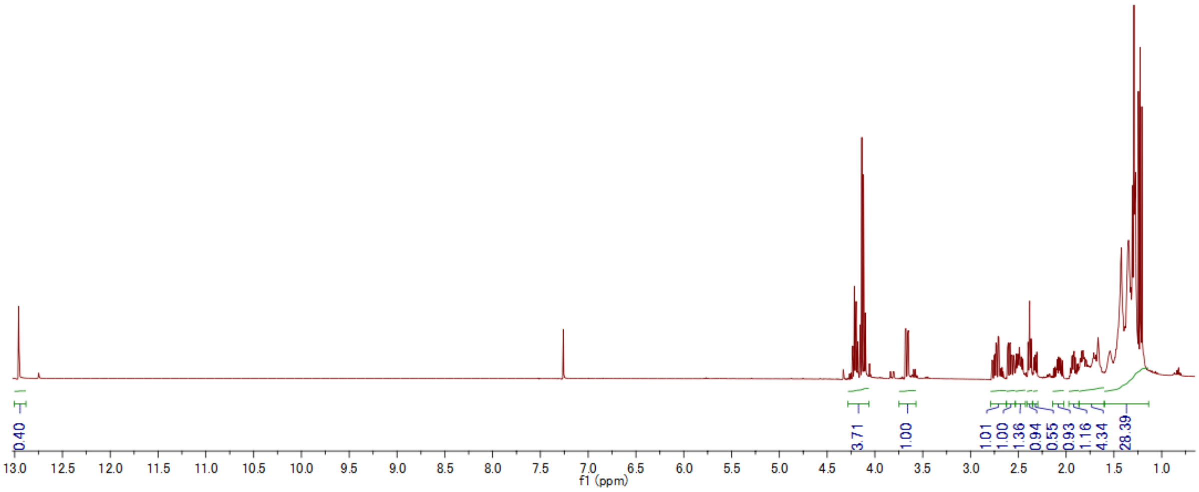

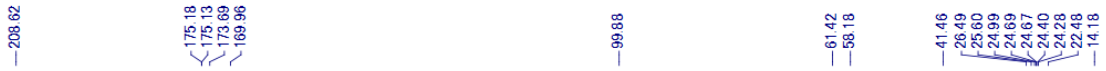

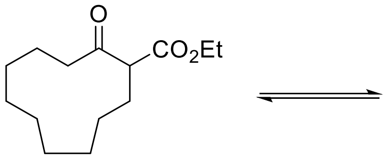

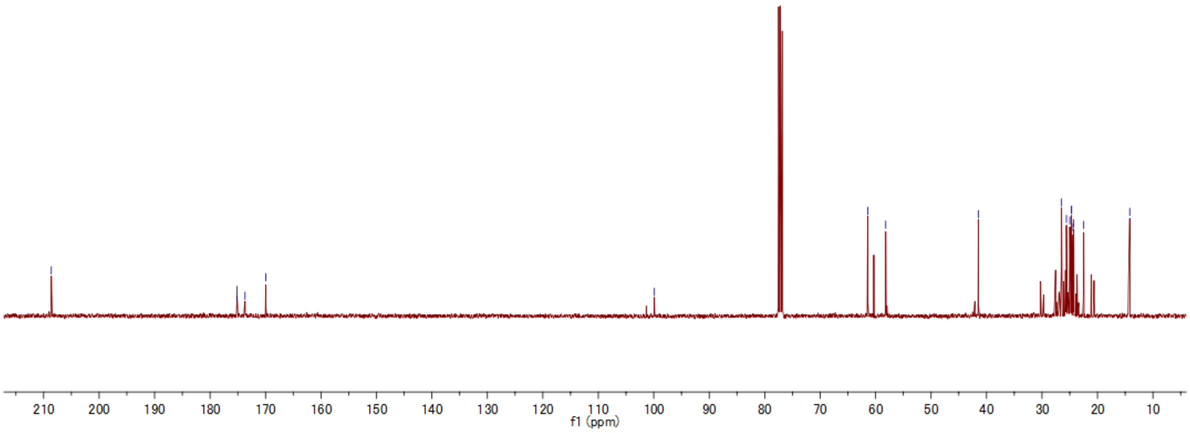

Compound S26

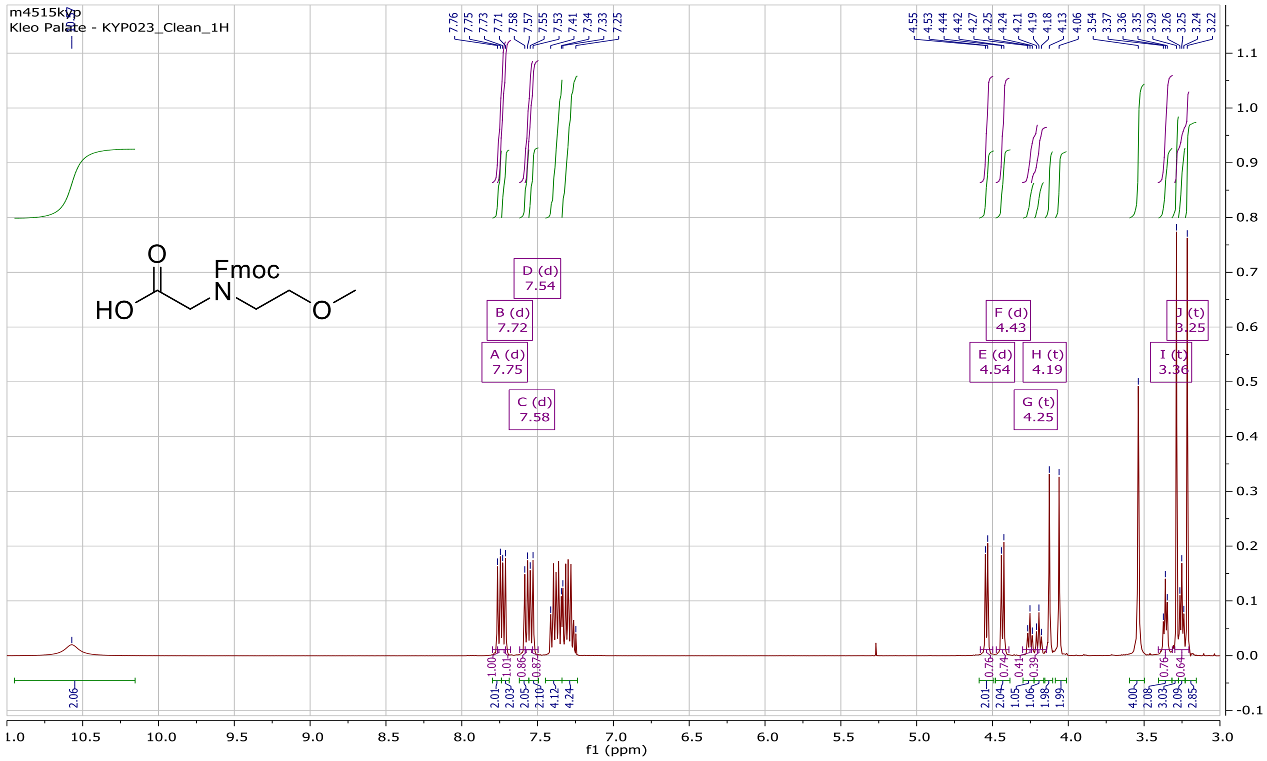

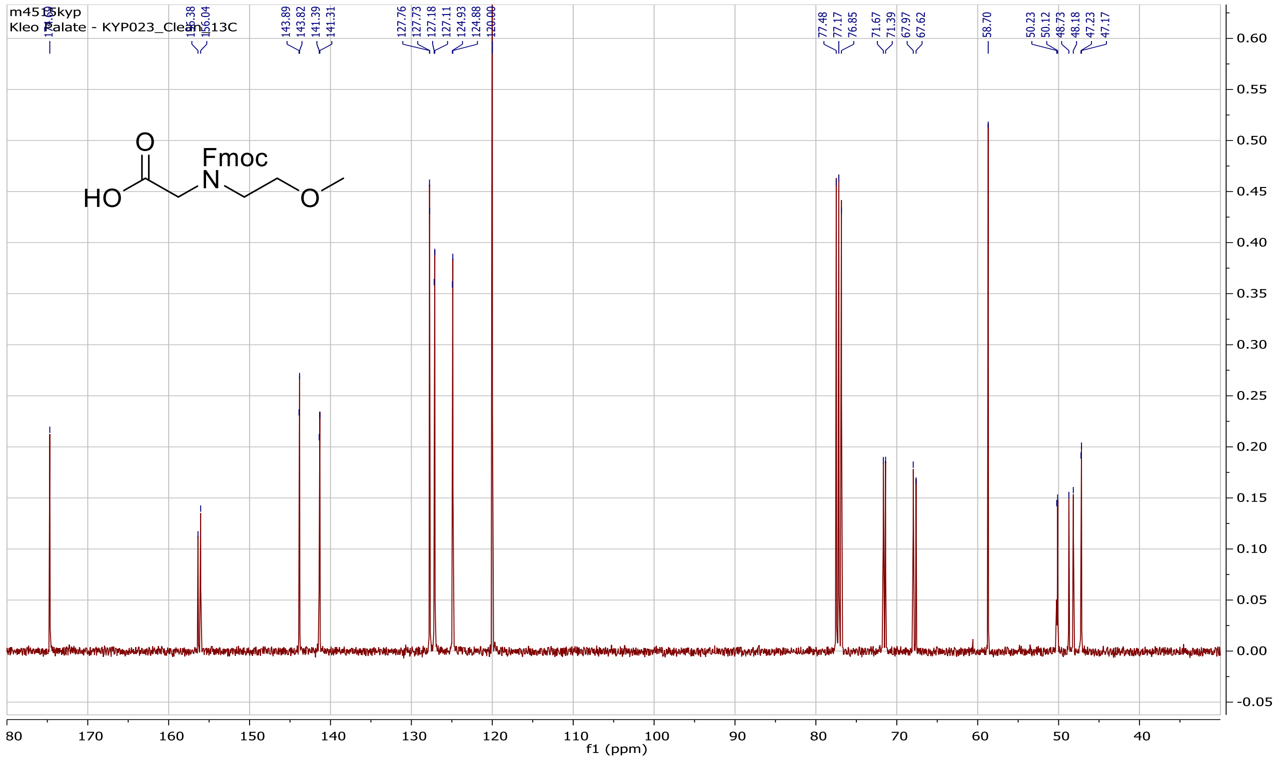

Compound S27

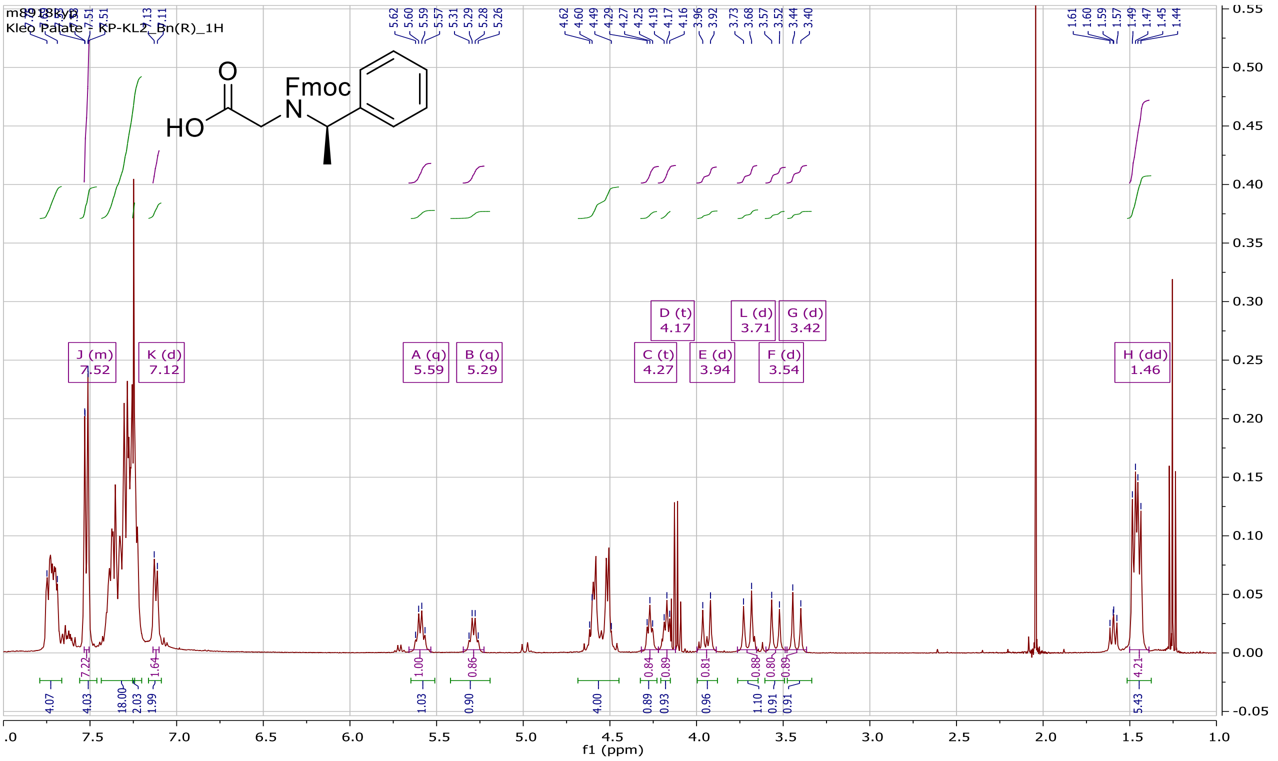

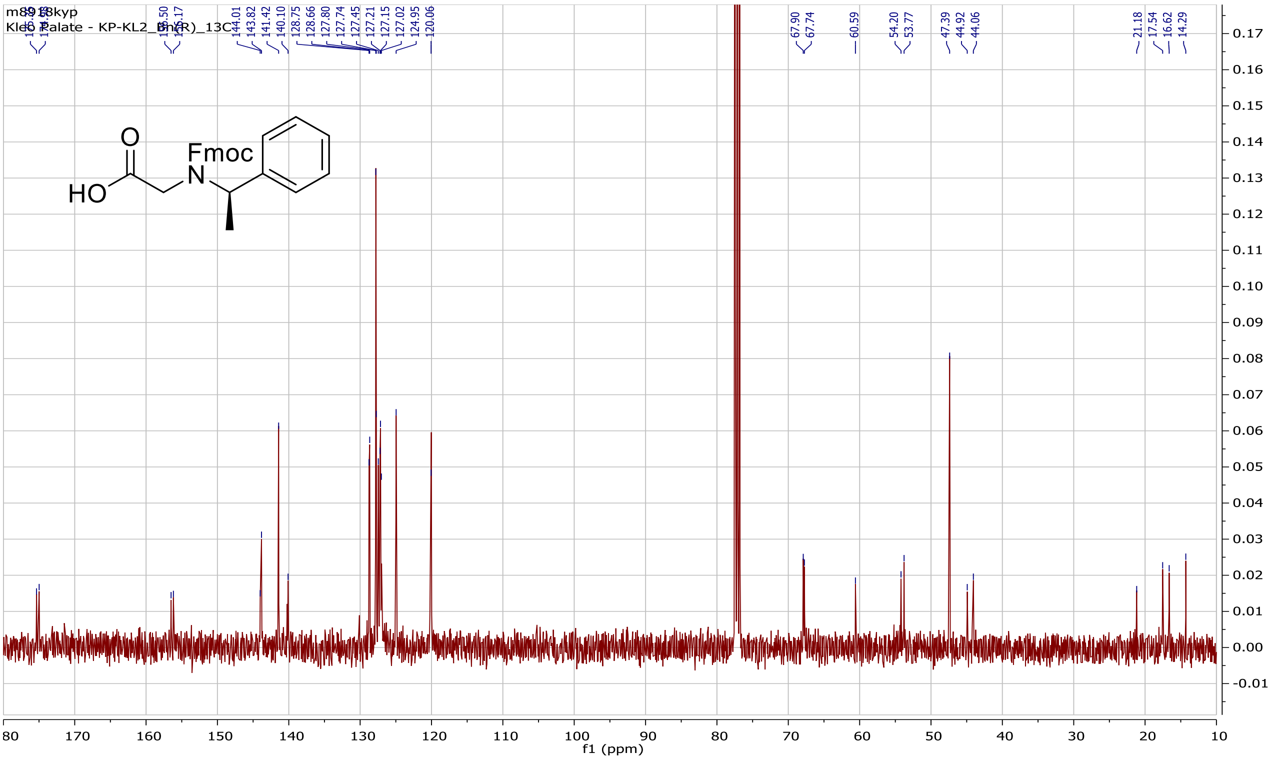

Compound S28

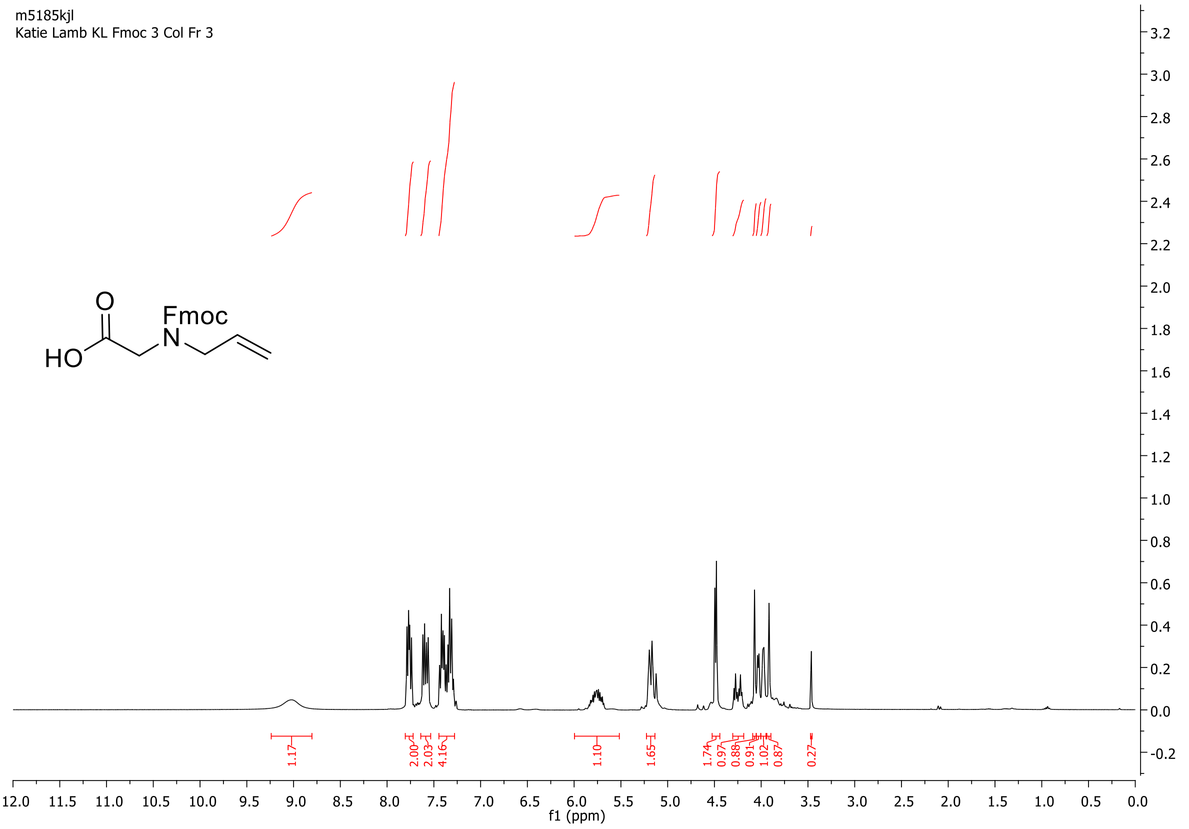

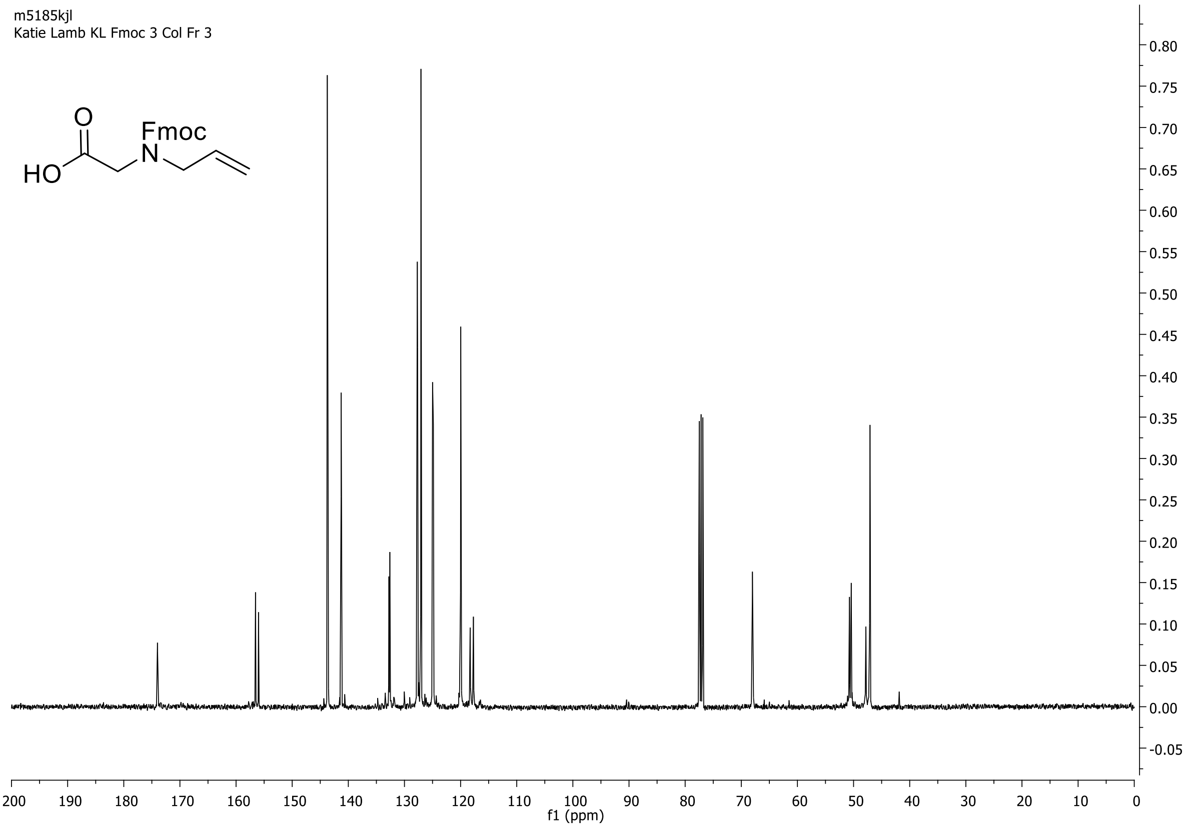

Compound S29

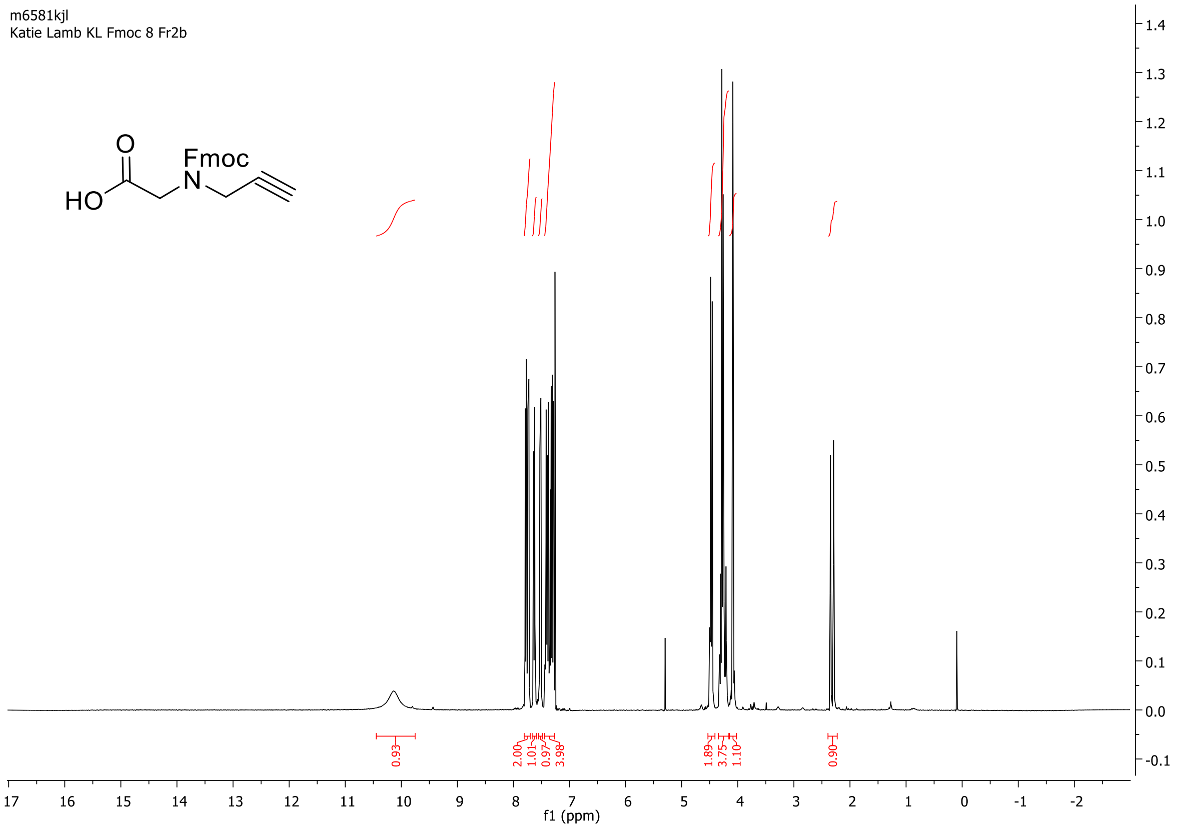

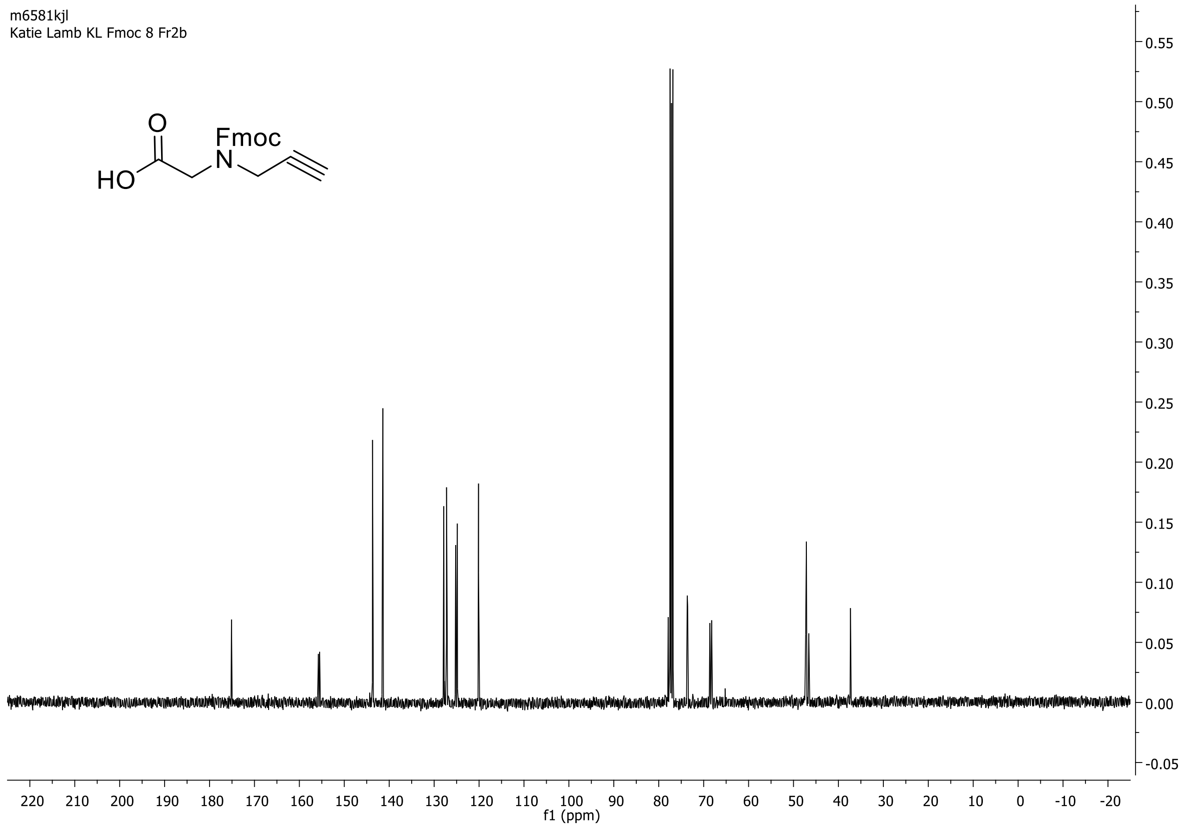

Compound S31

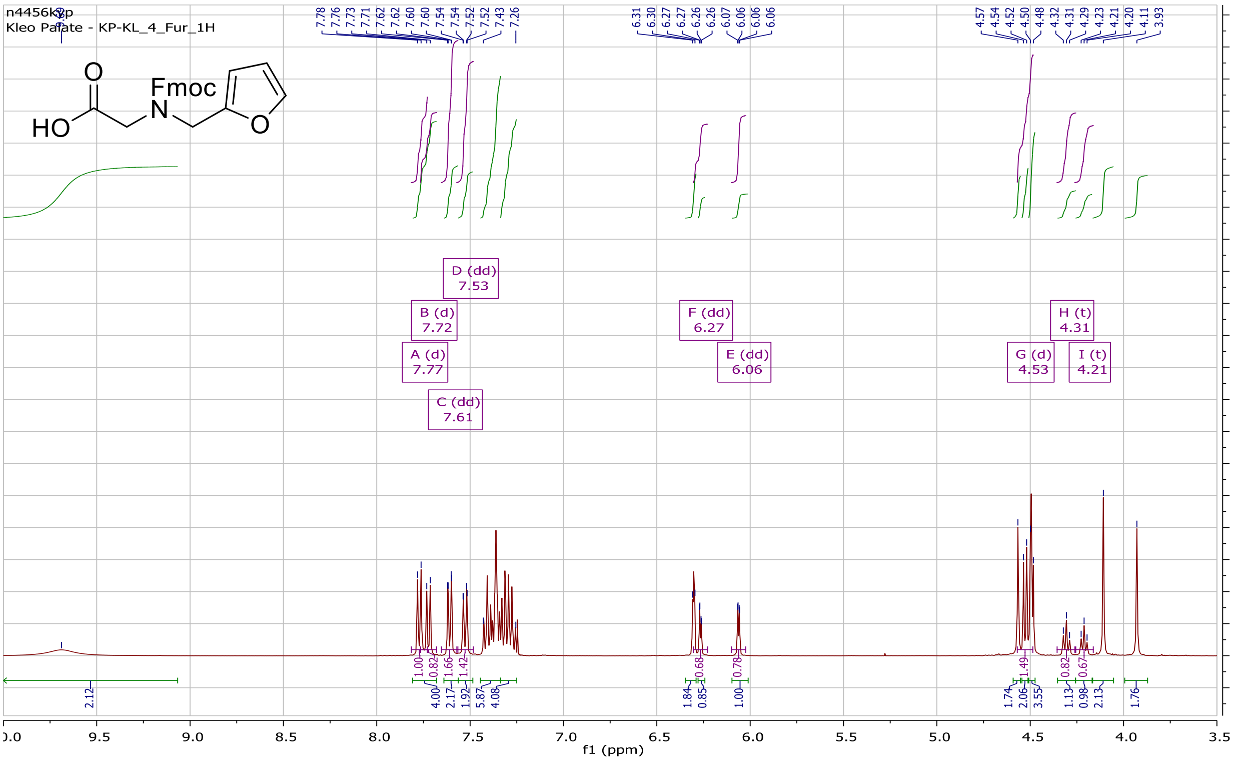

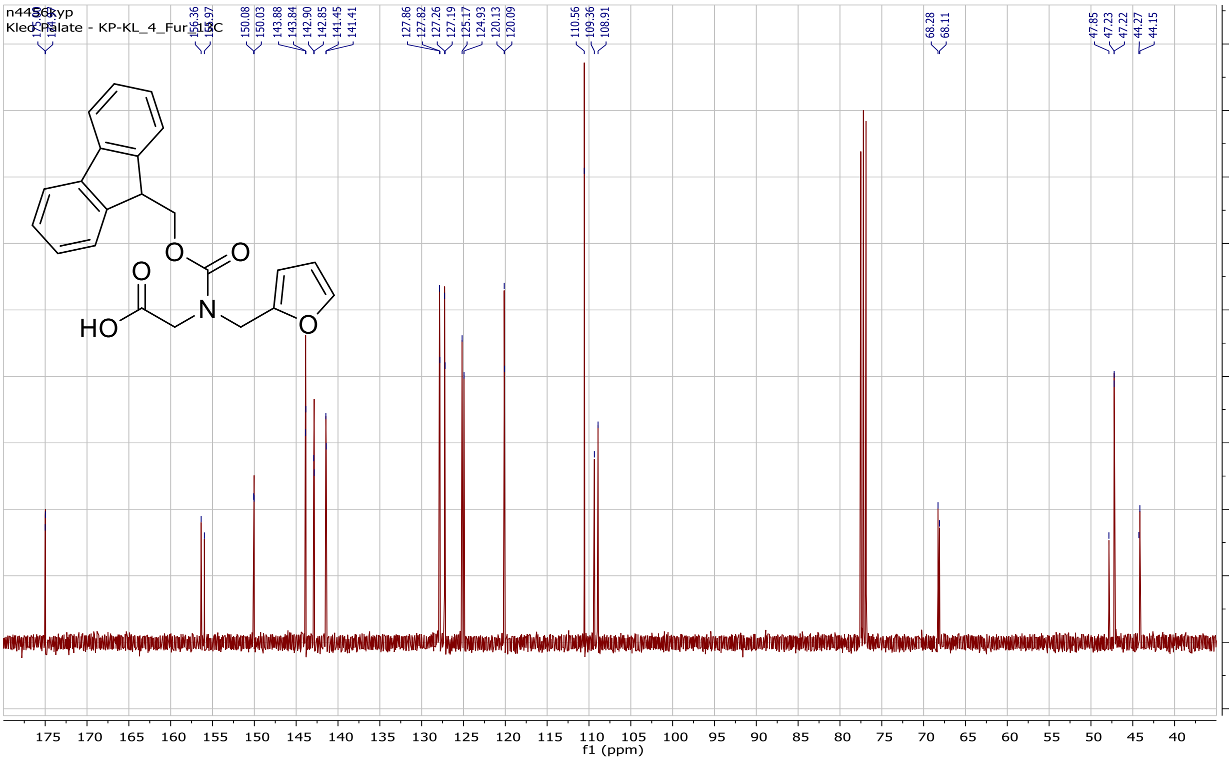

Compound S32

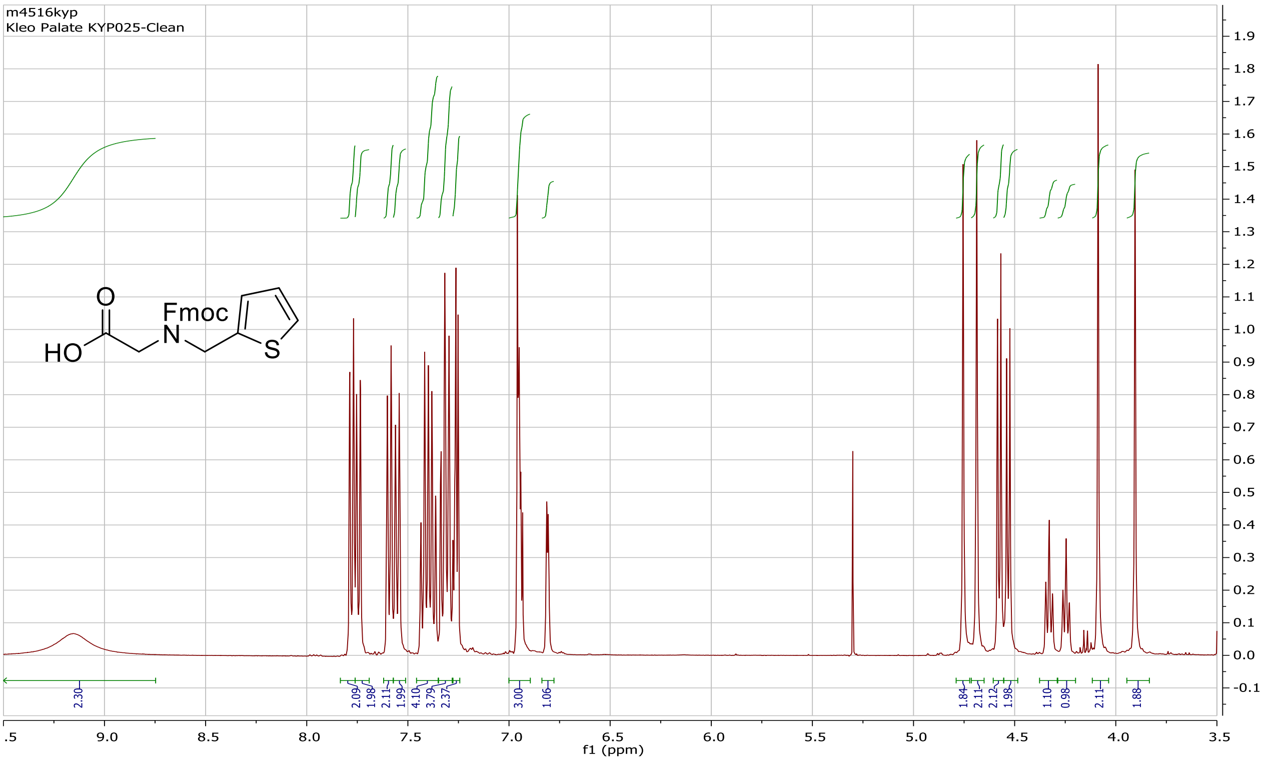

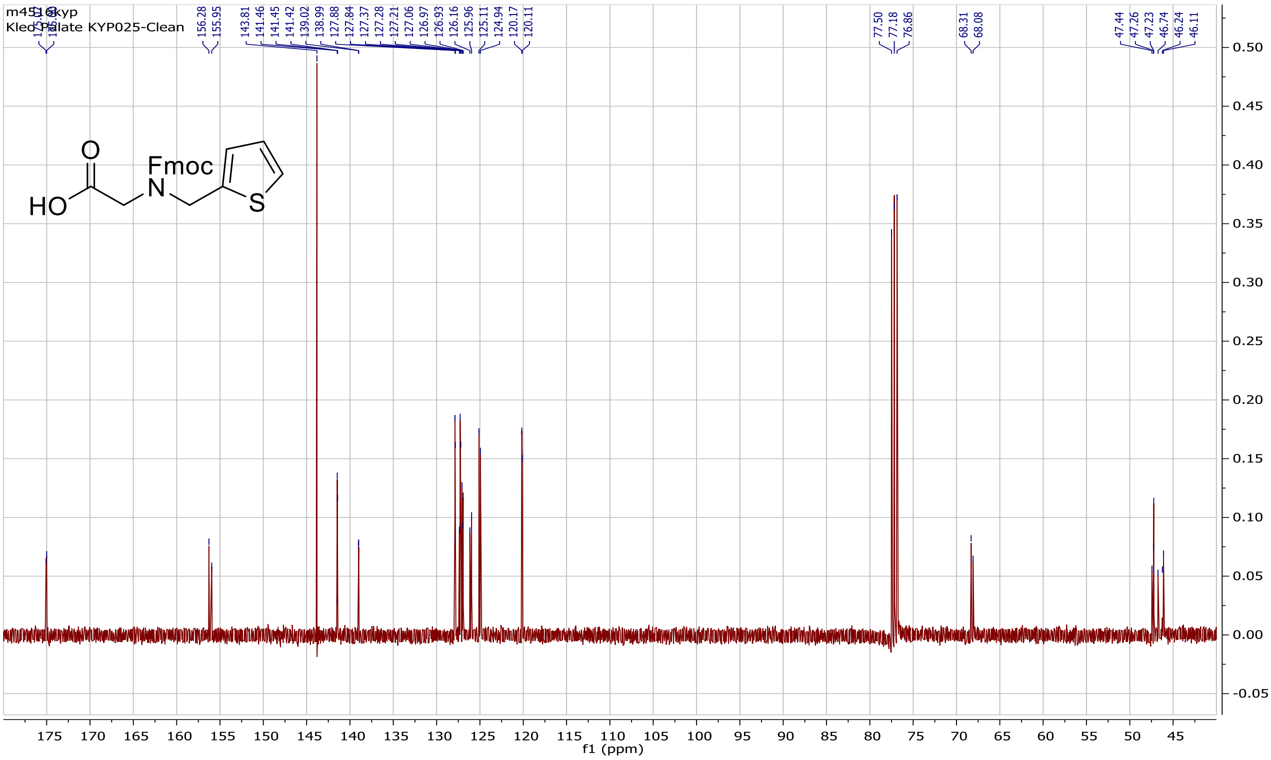

Compound S33

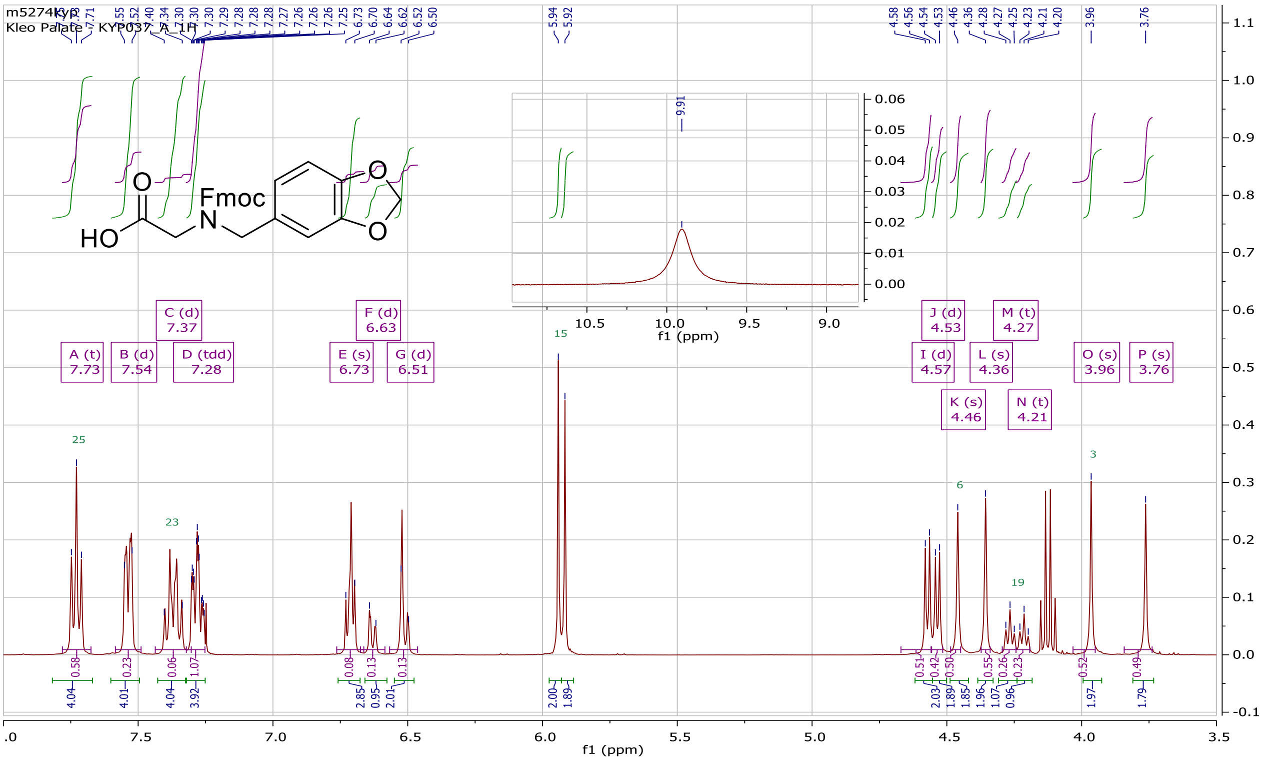

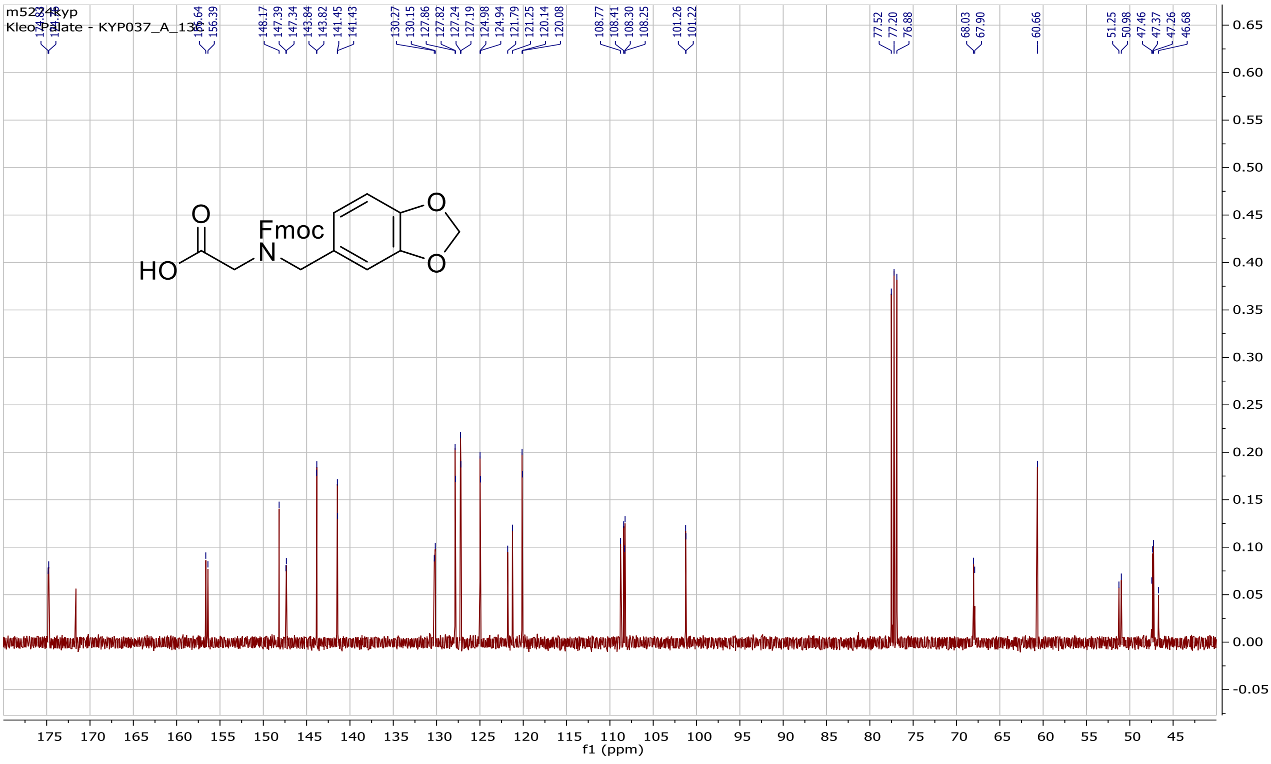

Compound S34

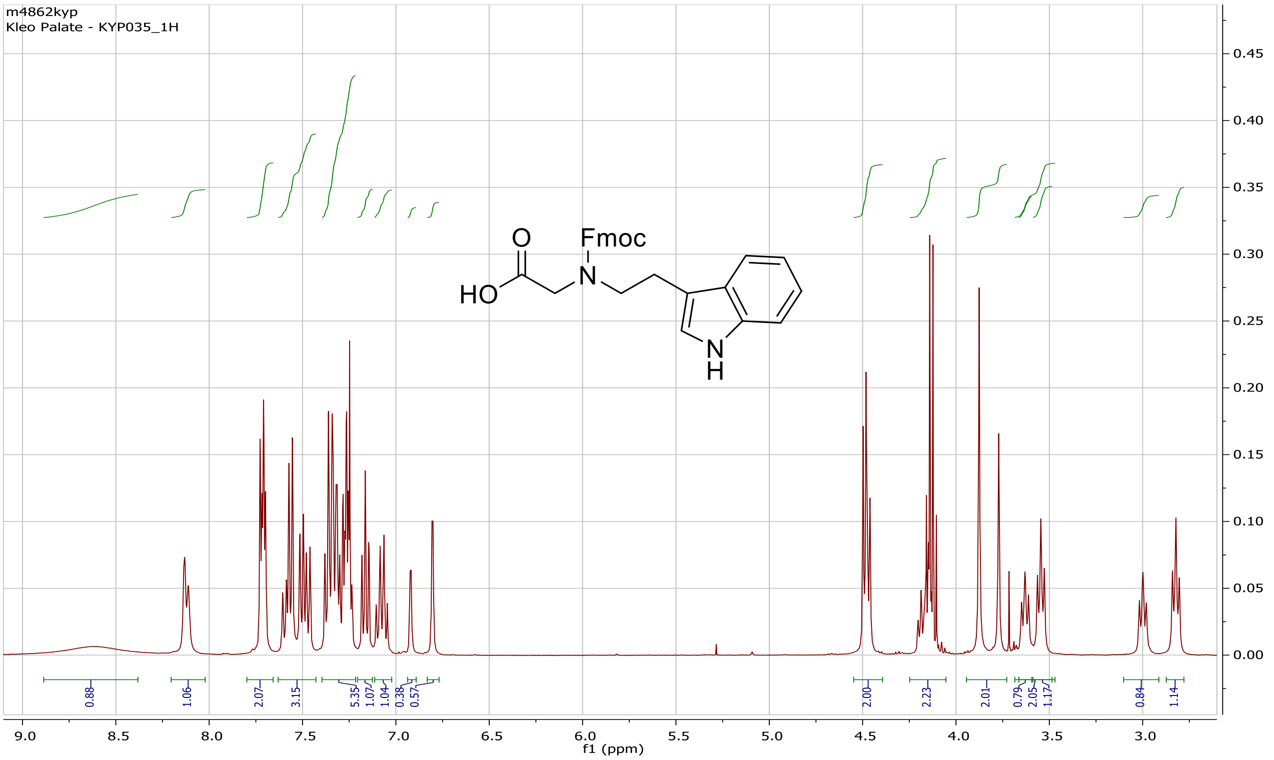

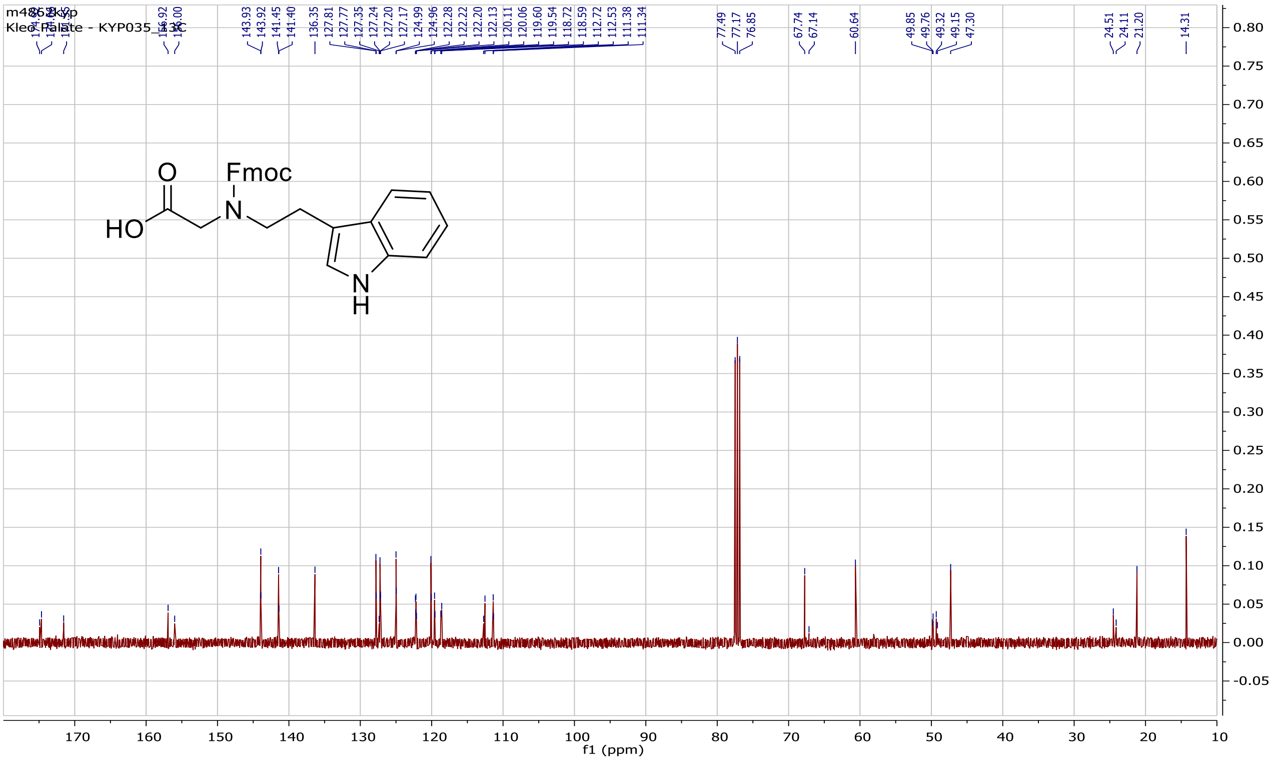

Compound 20RE

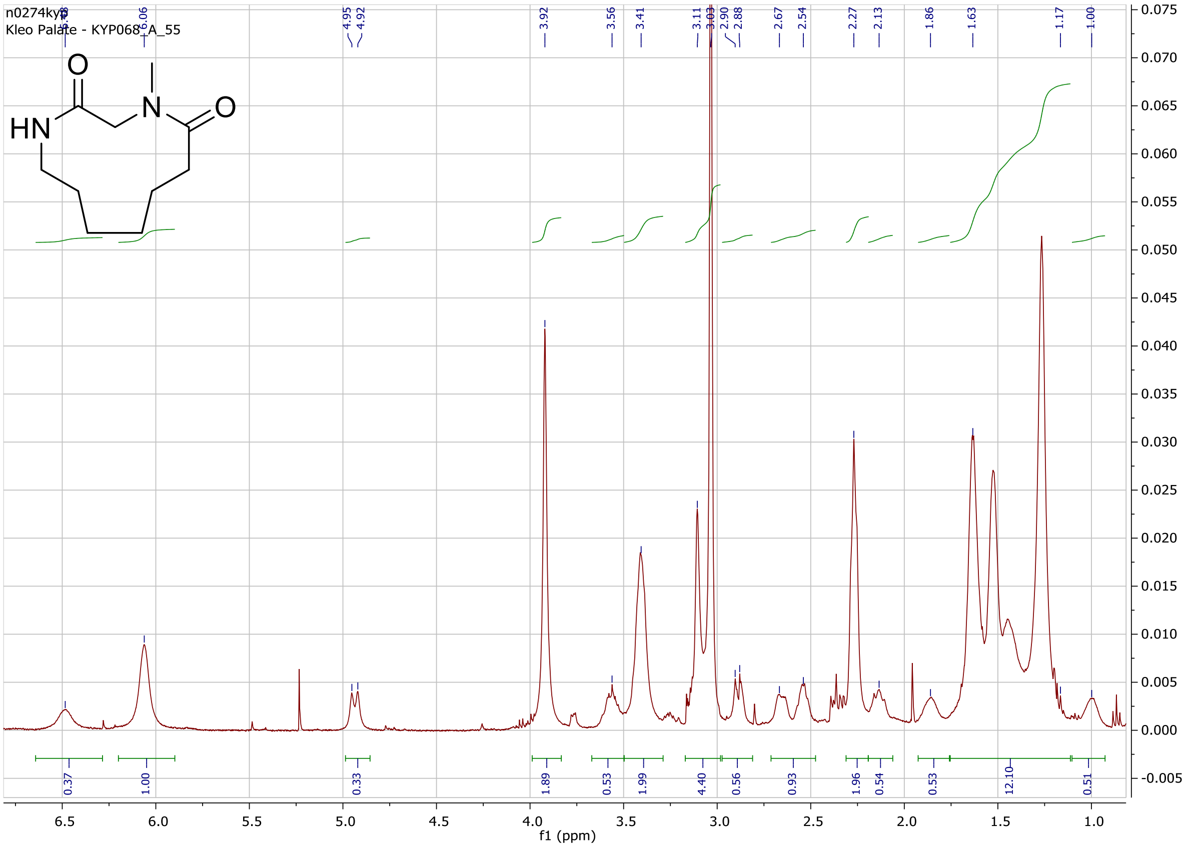

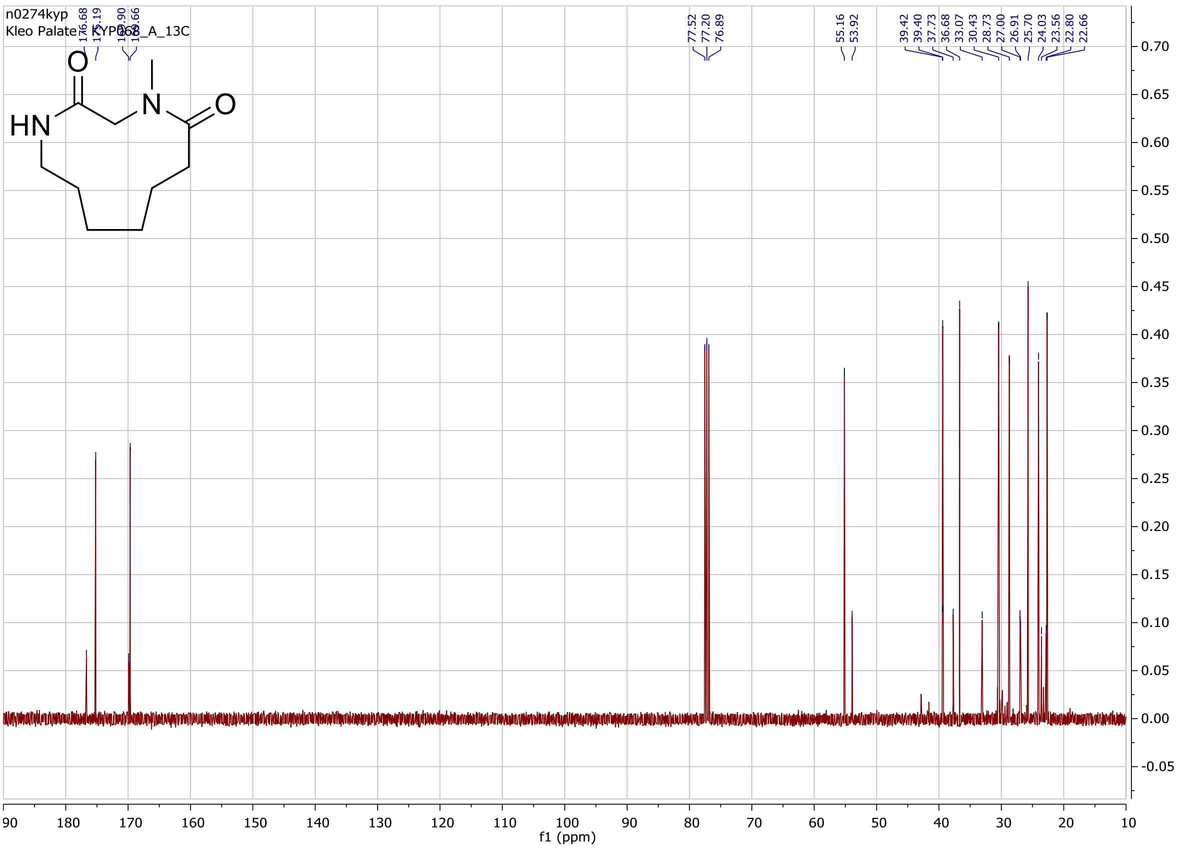

Compound 21RE

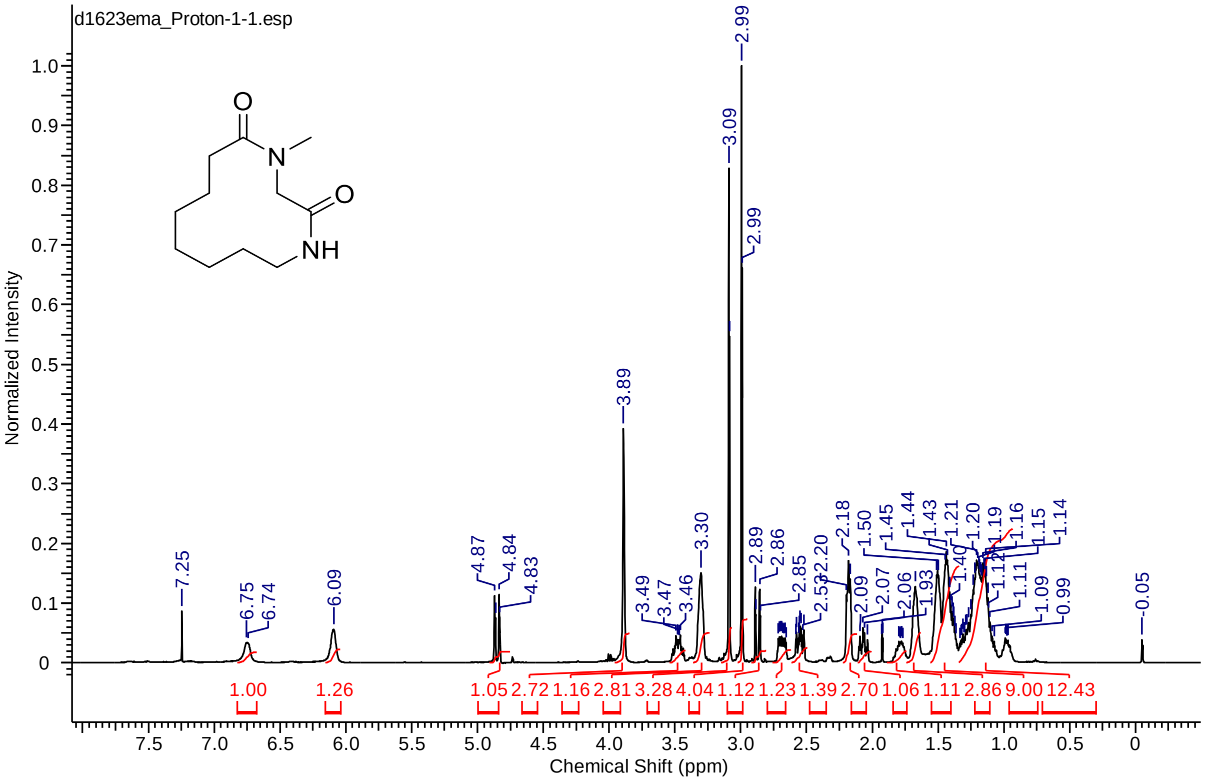

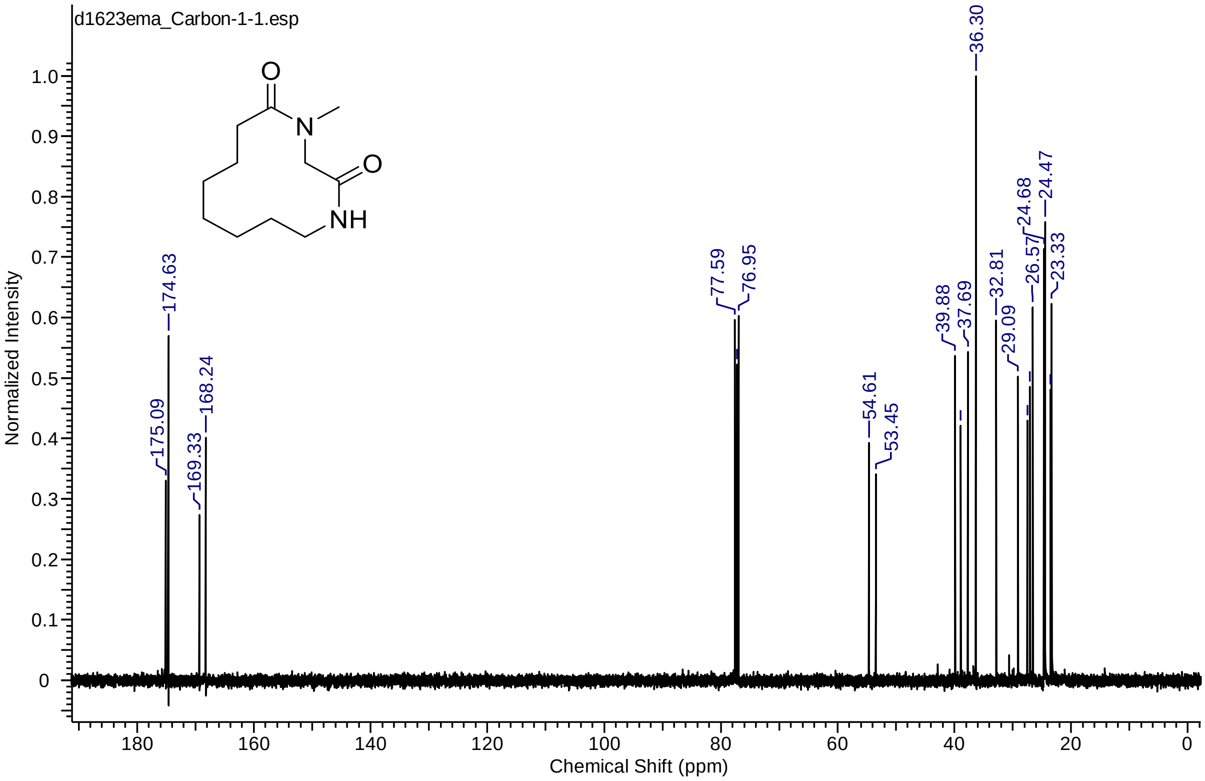

Compound 22RE

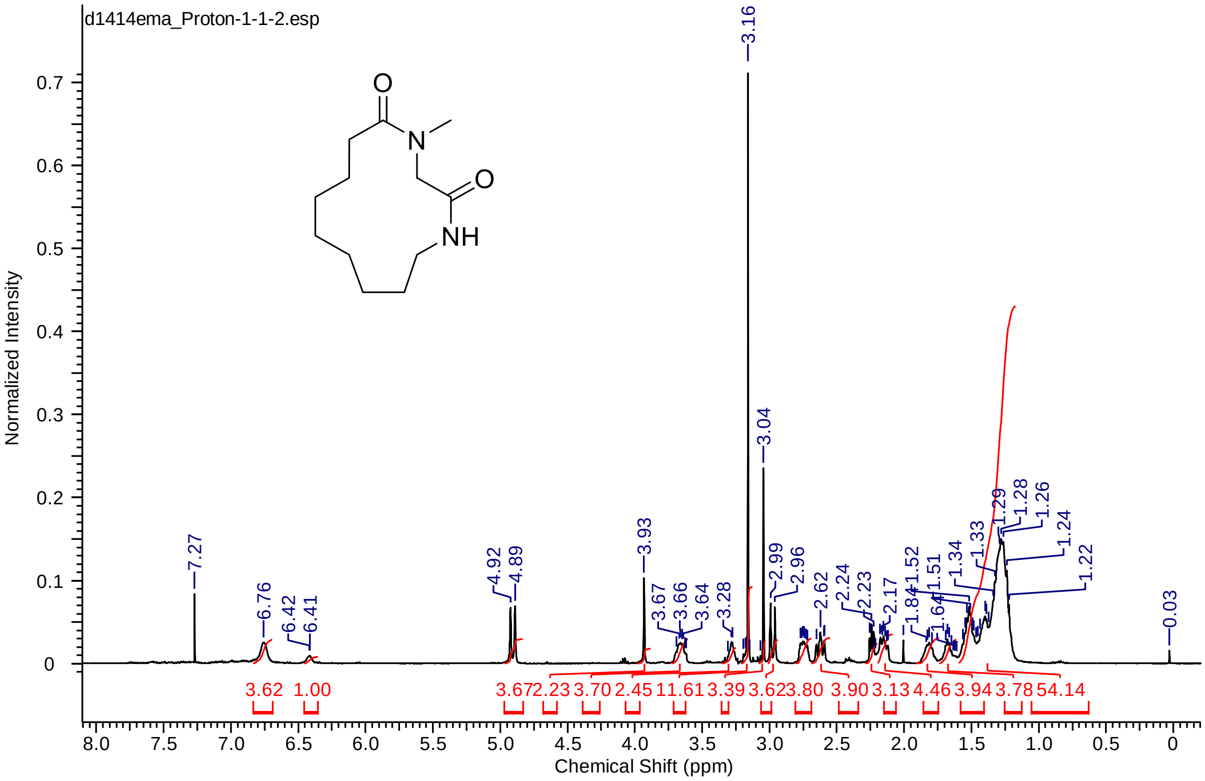

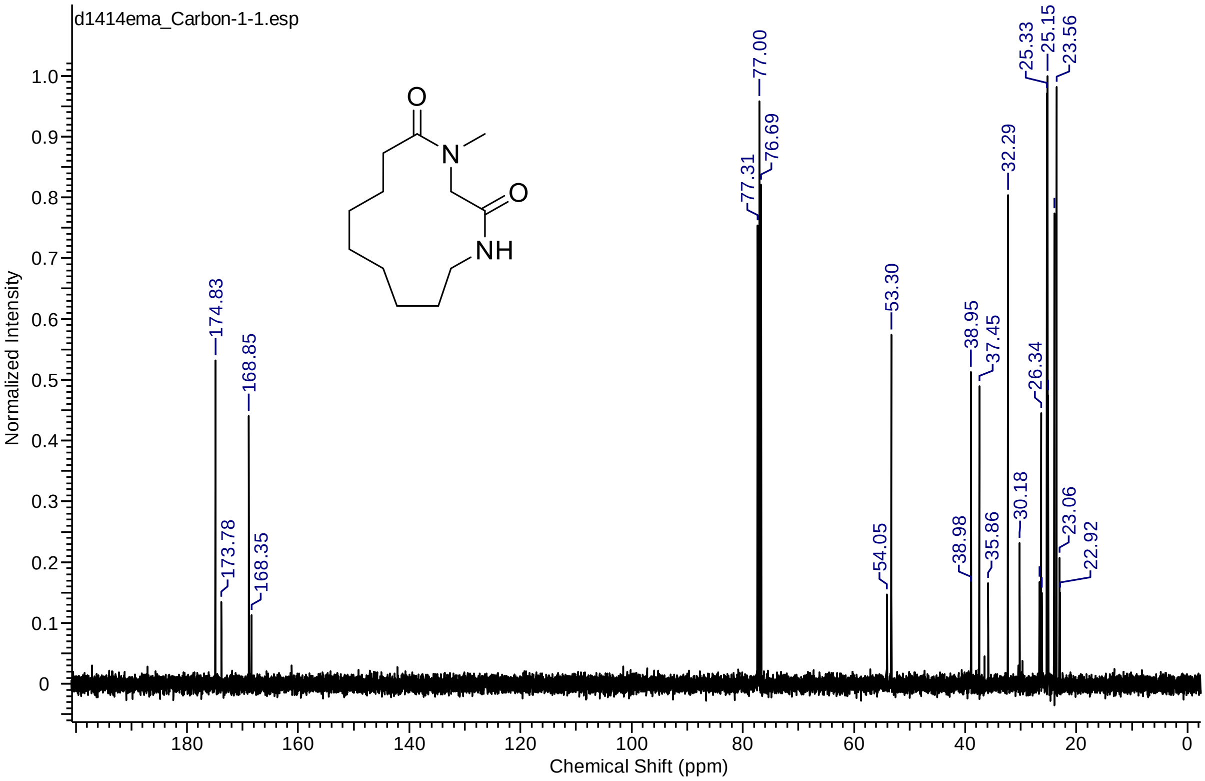

Compound 23RE

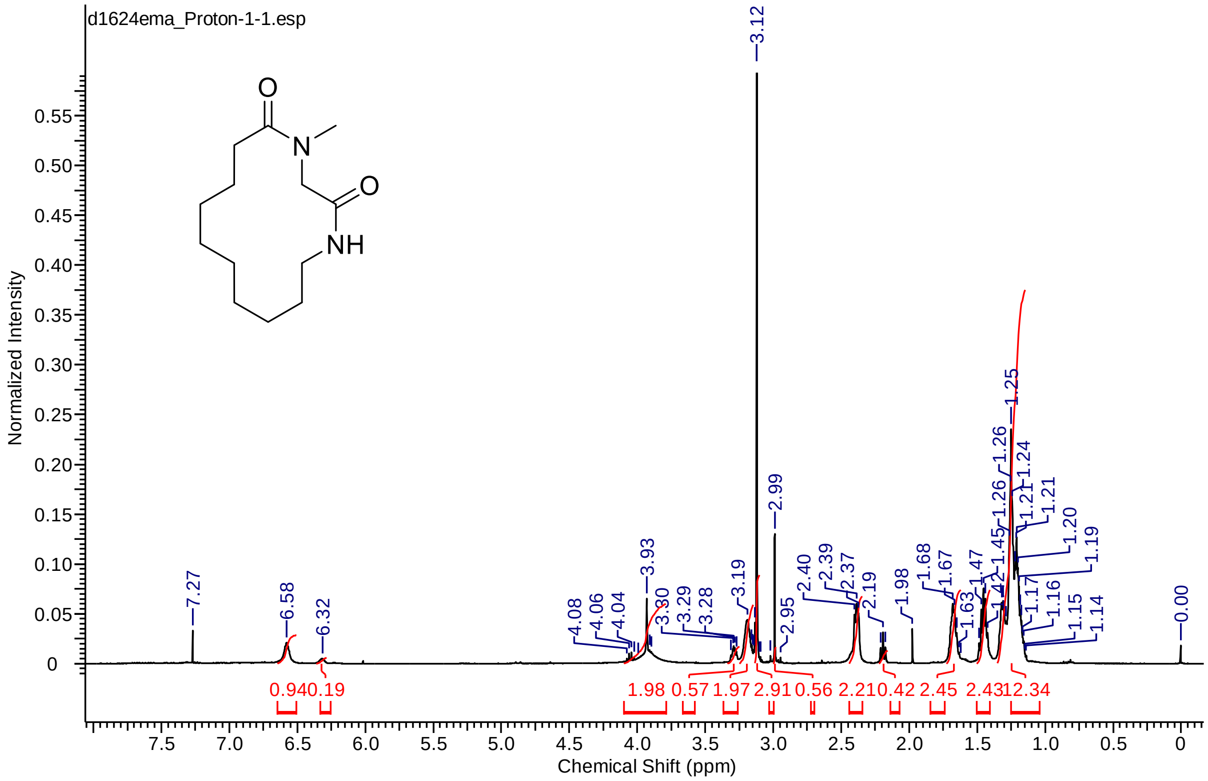

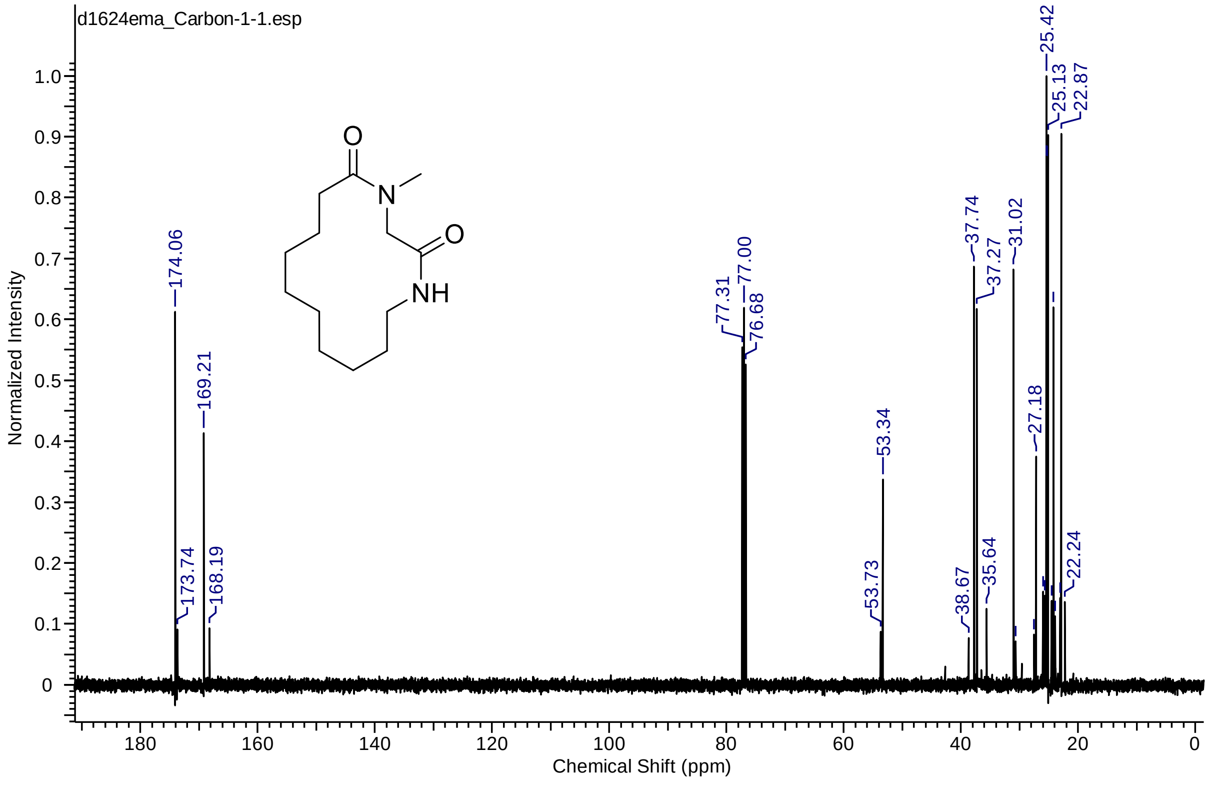

Compound 24RE

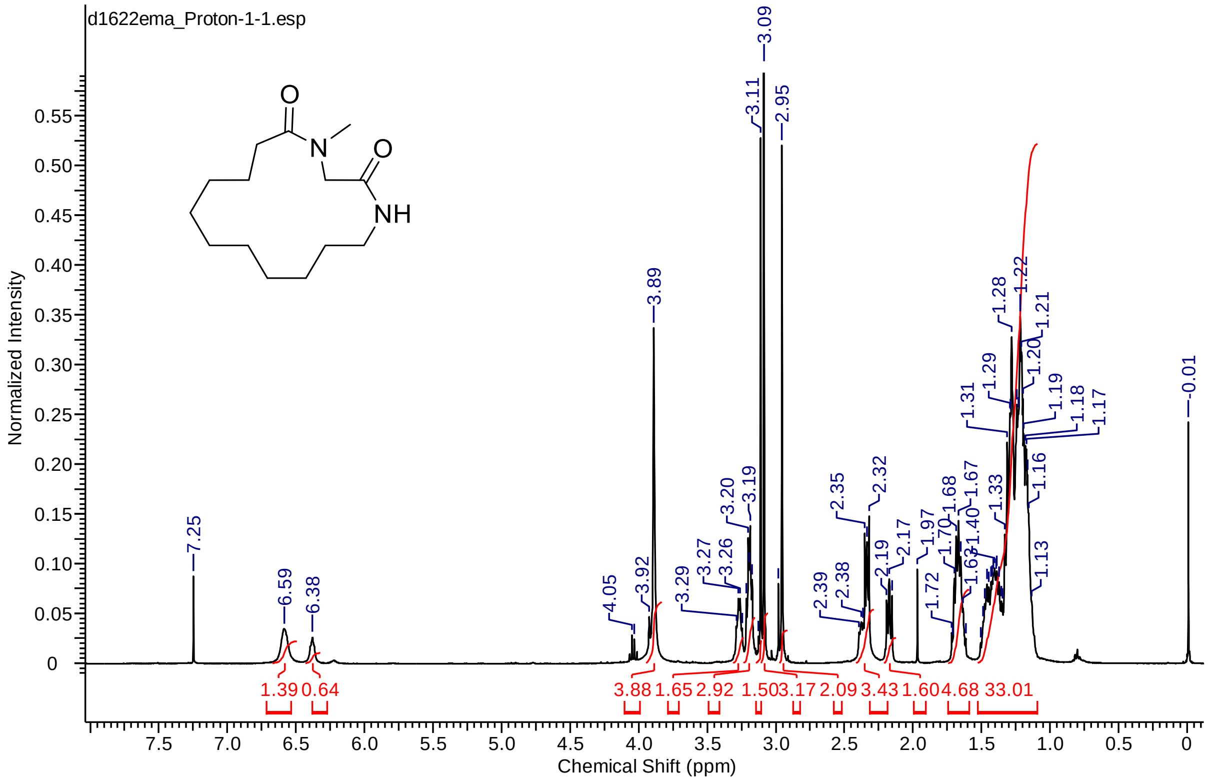

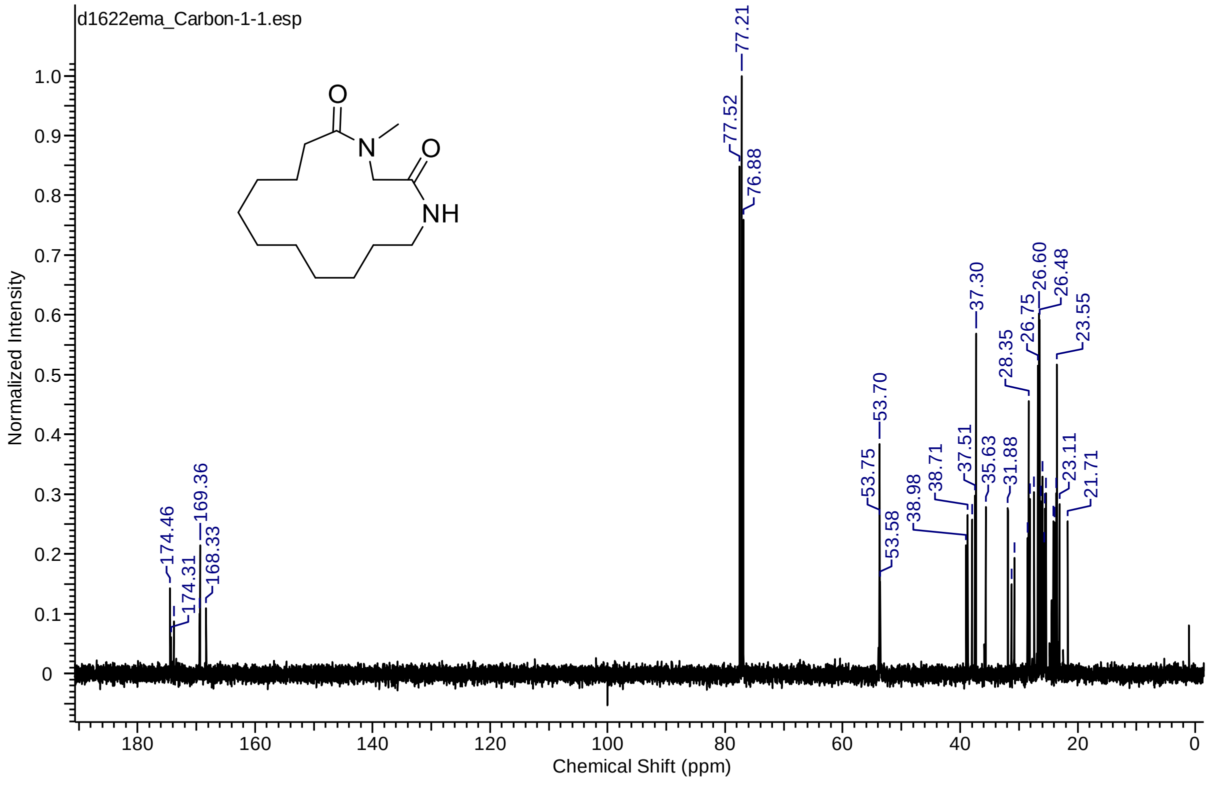

Compound 27a

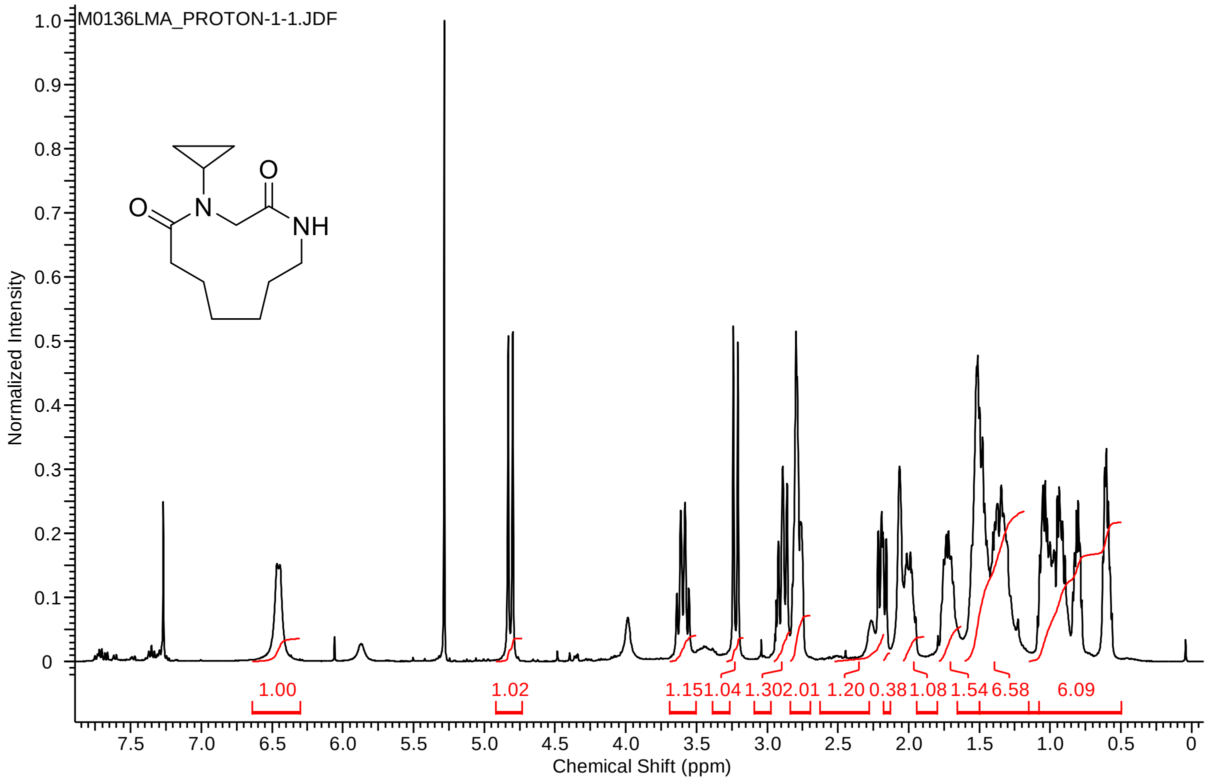

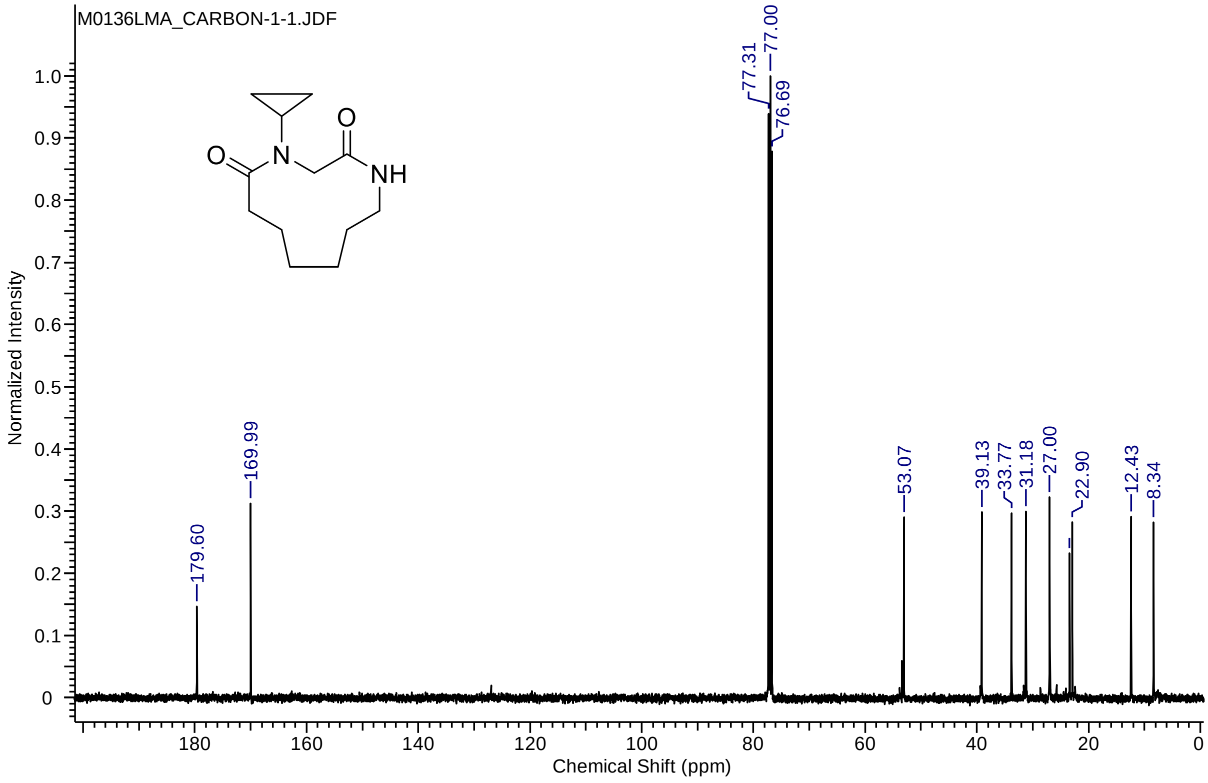

Compound 27b

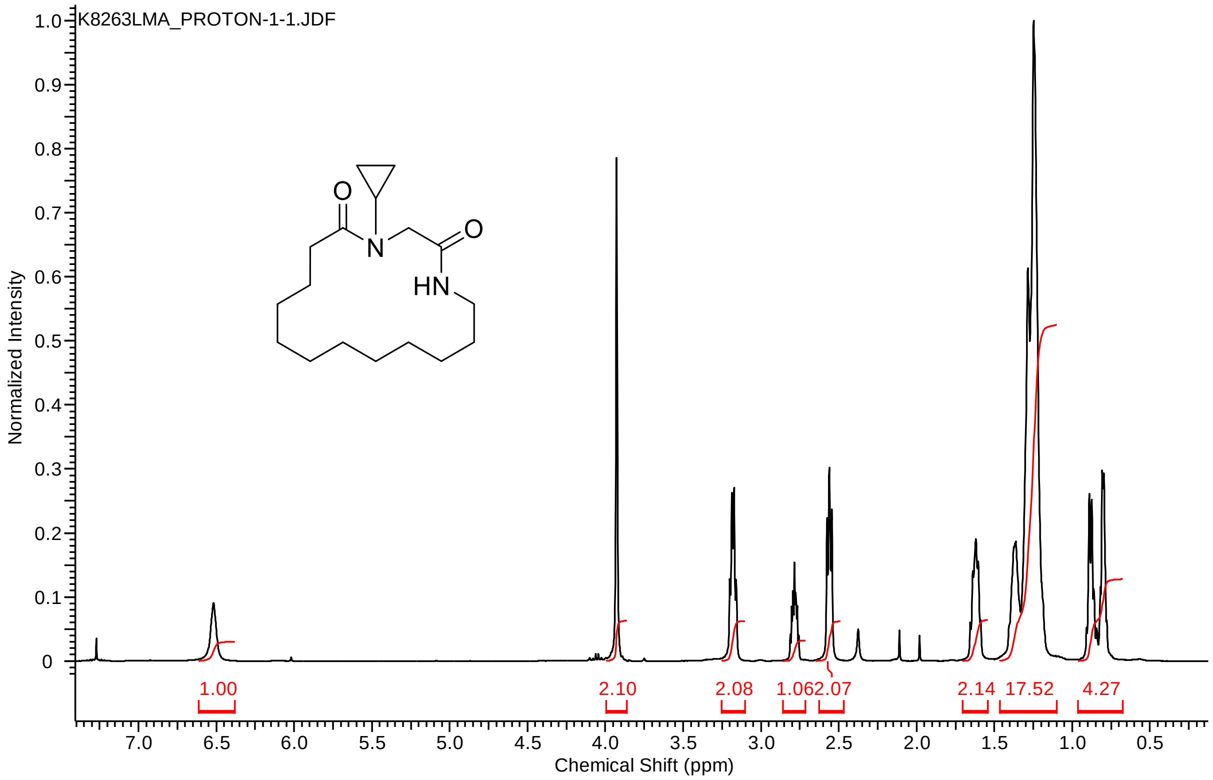

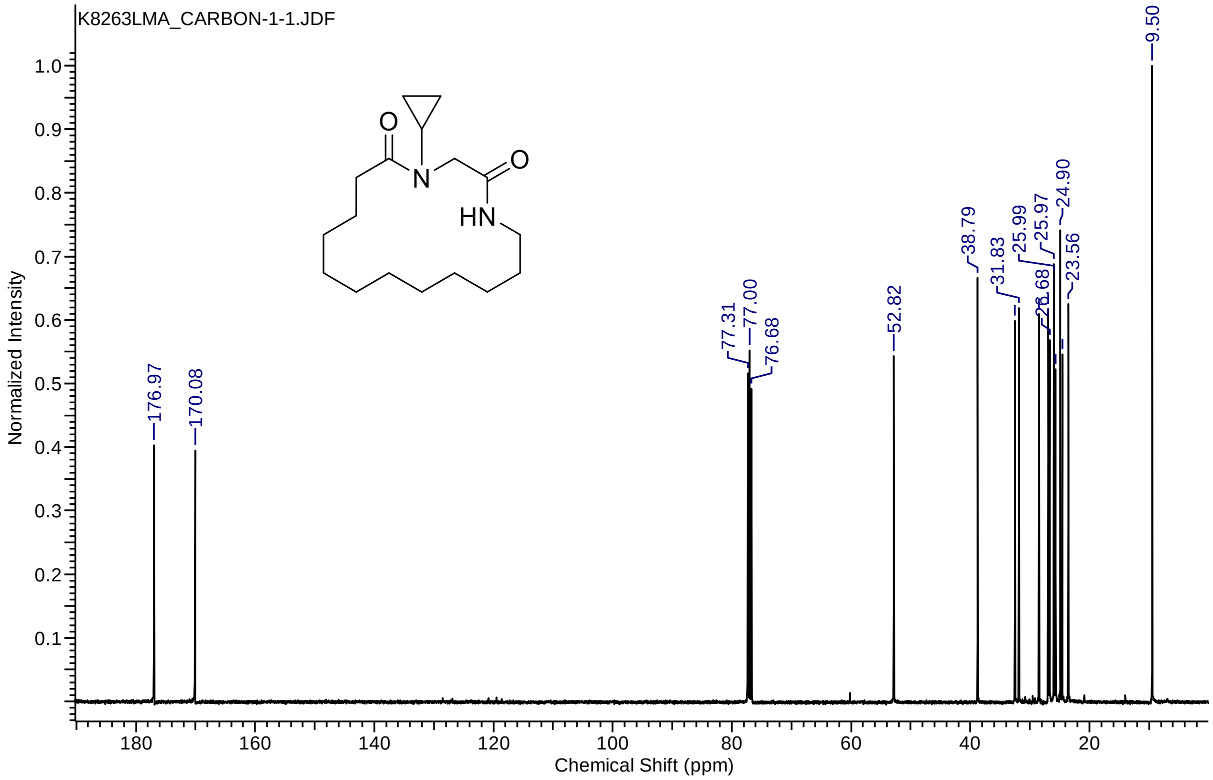

Compound 27c

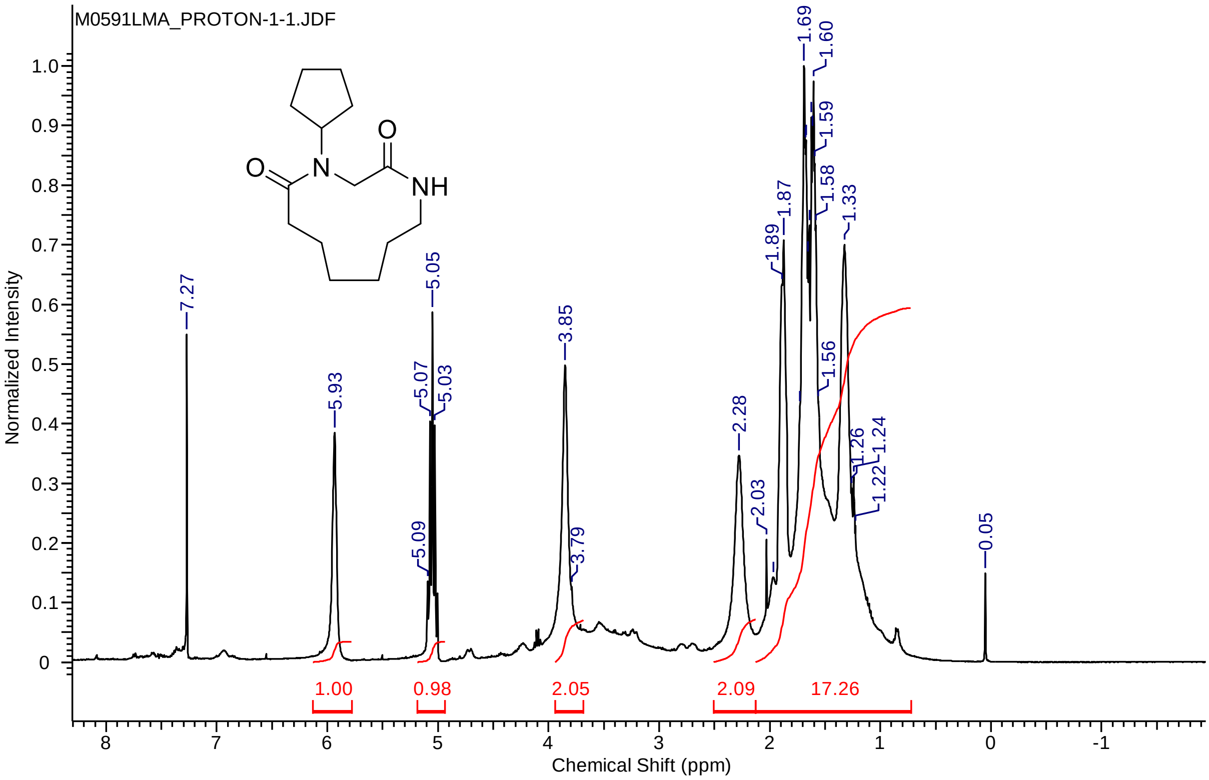

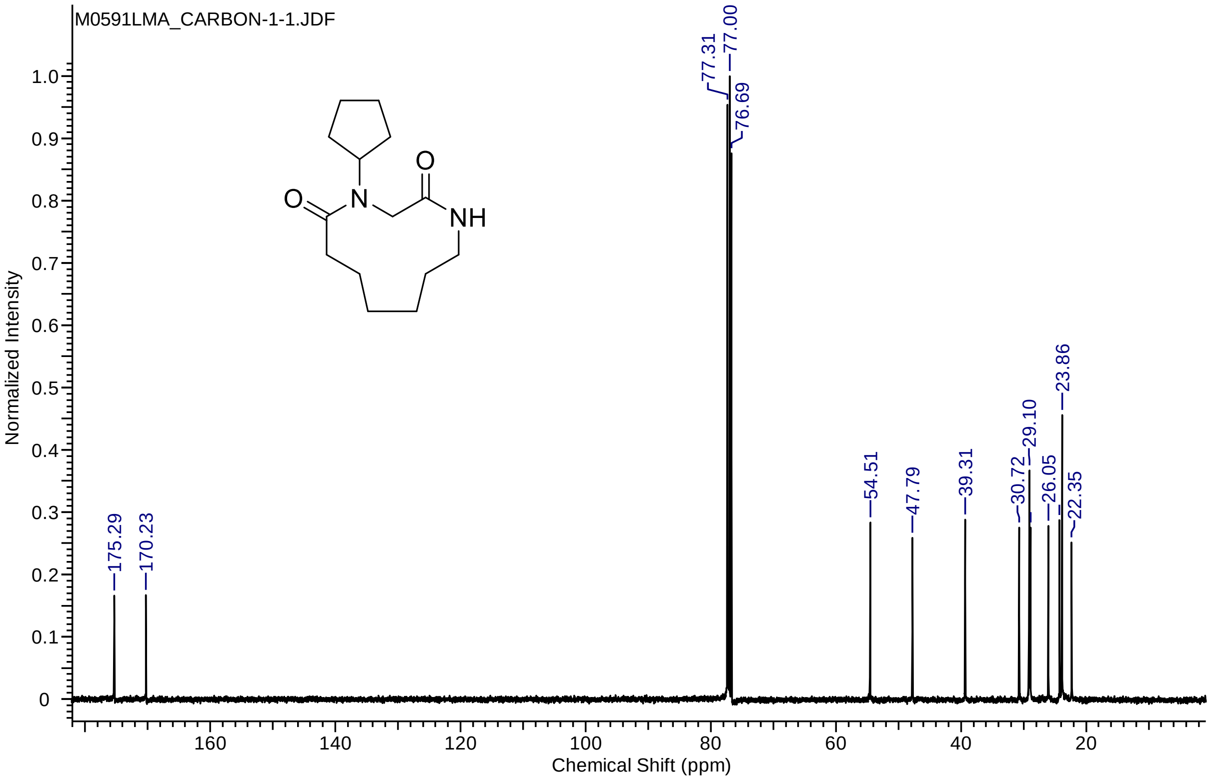

Compound 27d

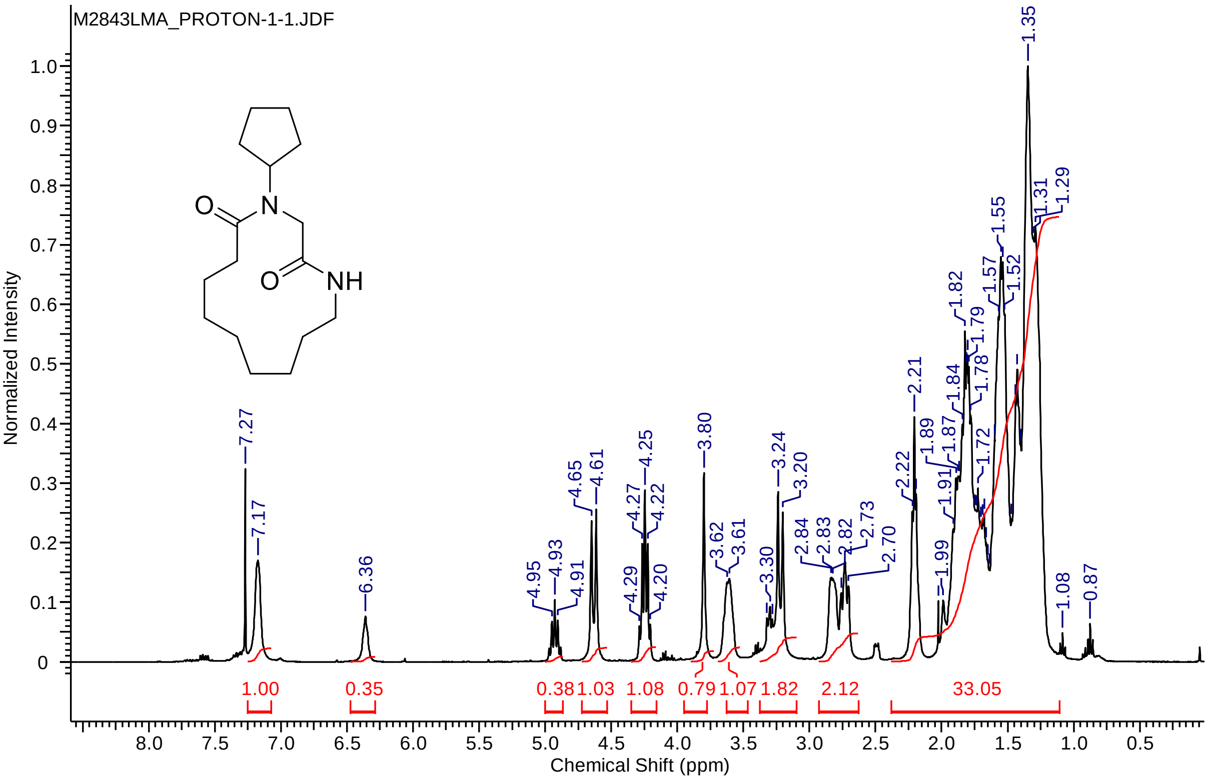

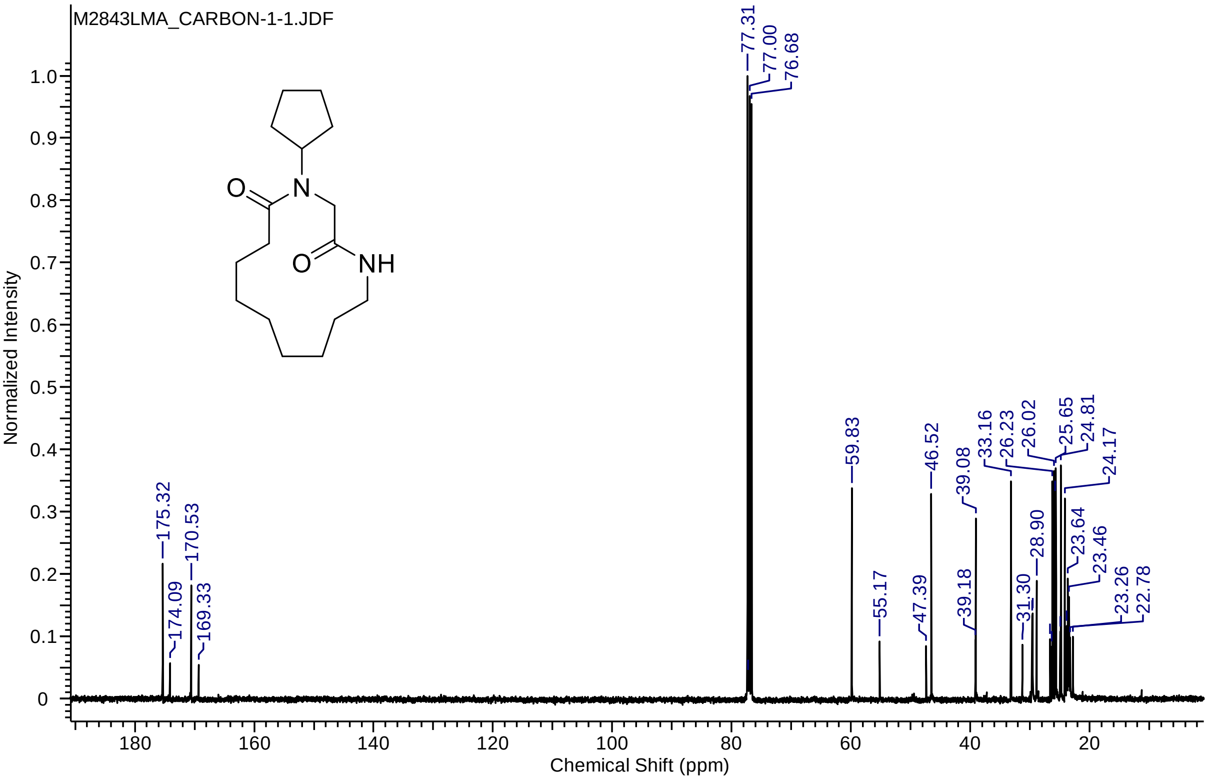

Compound 27e

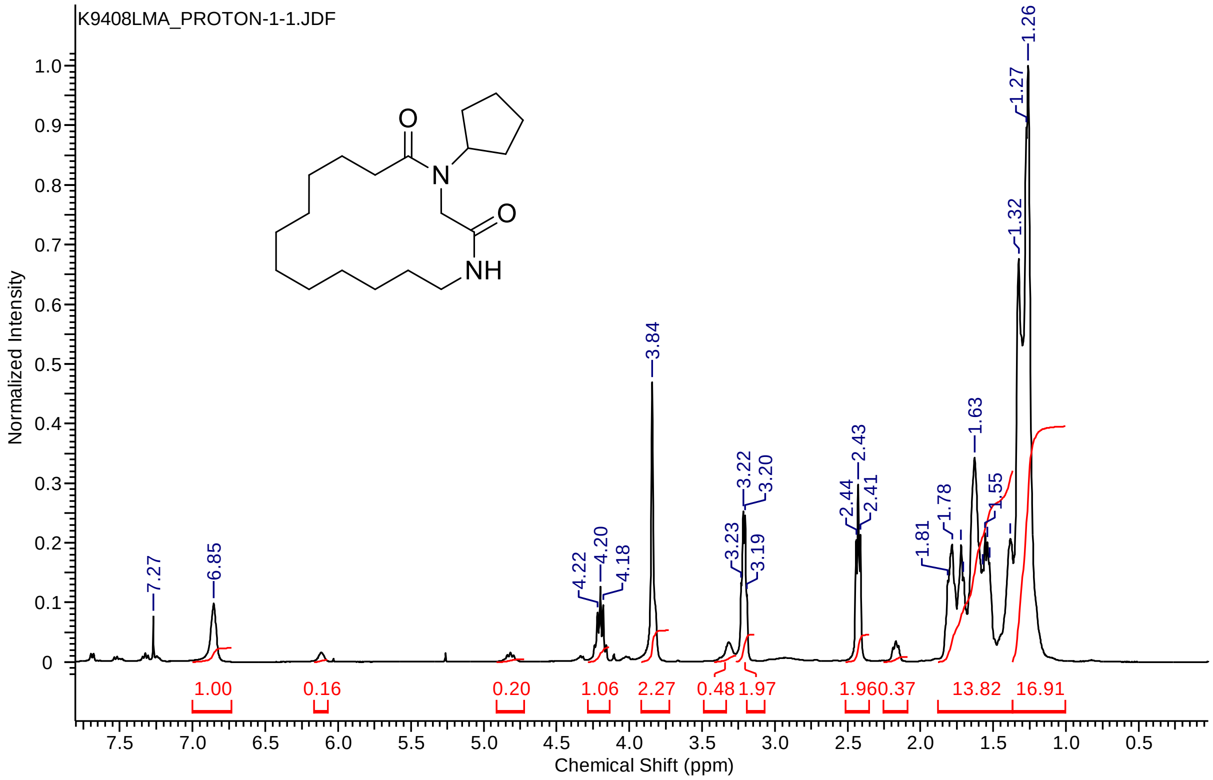

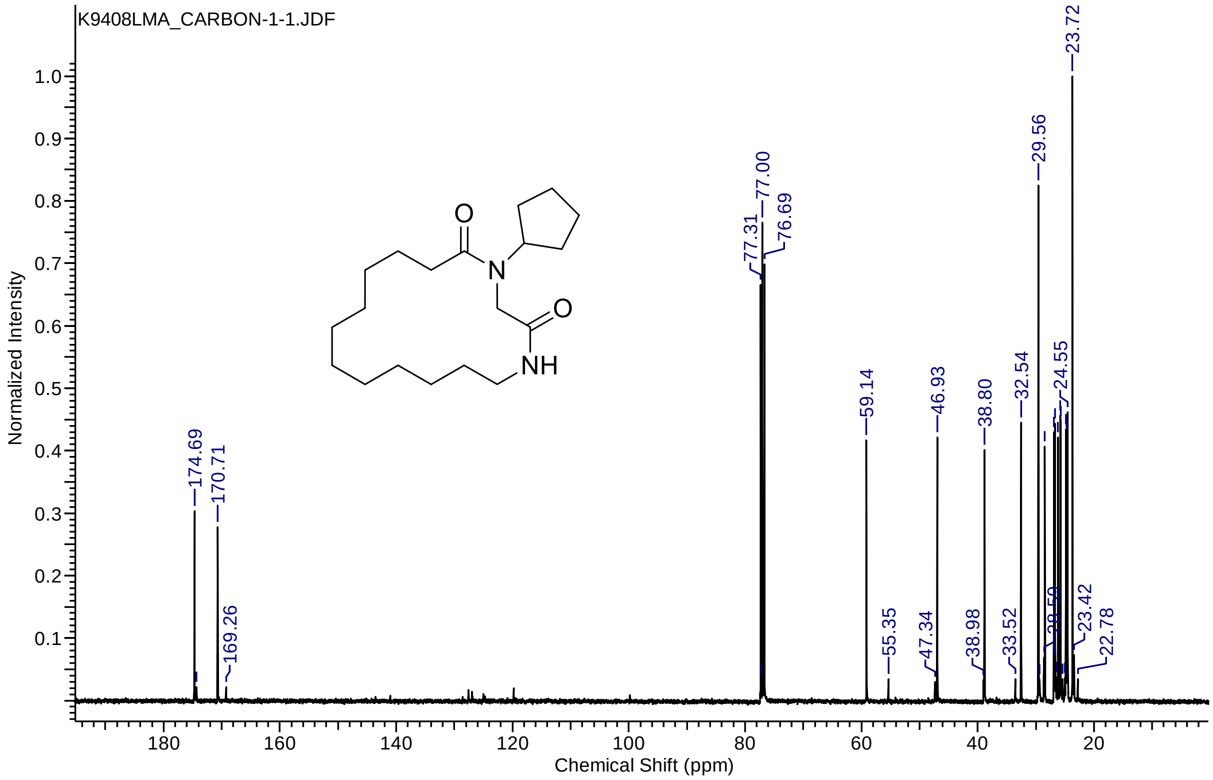

Compound 27f

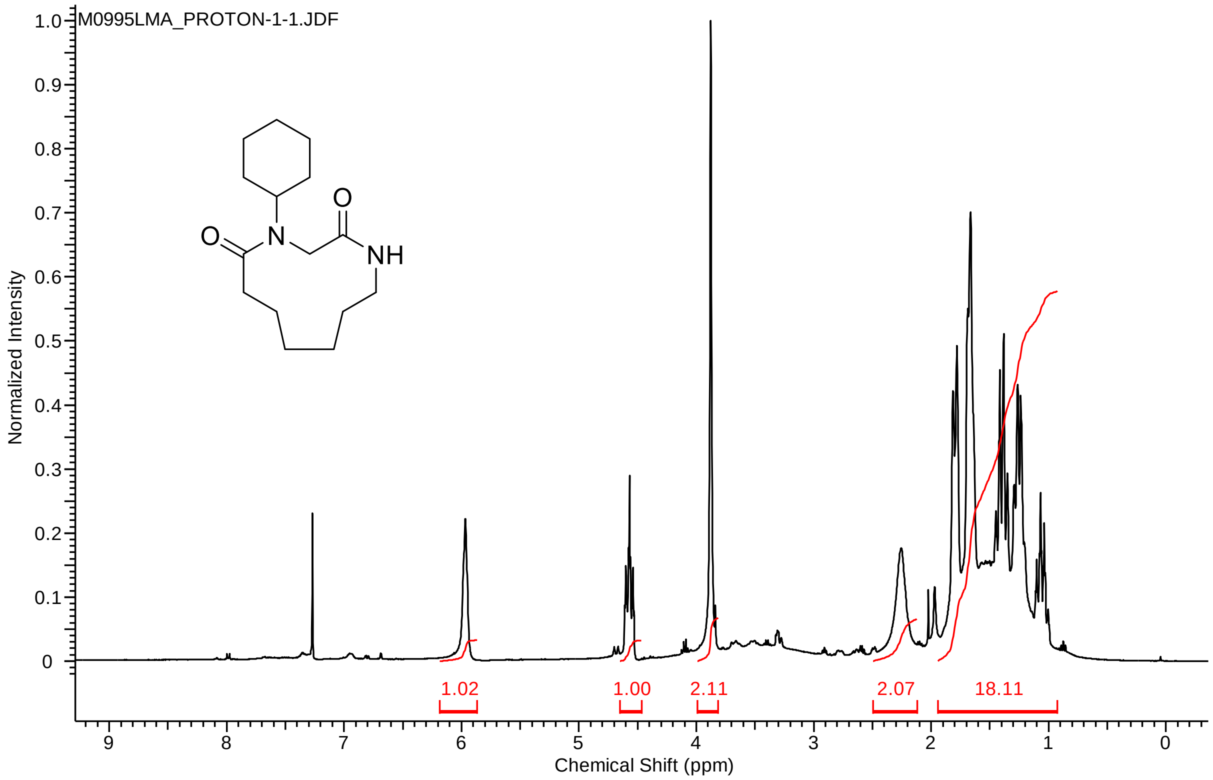

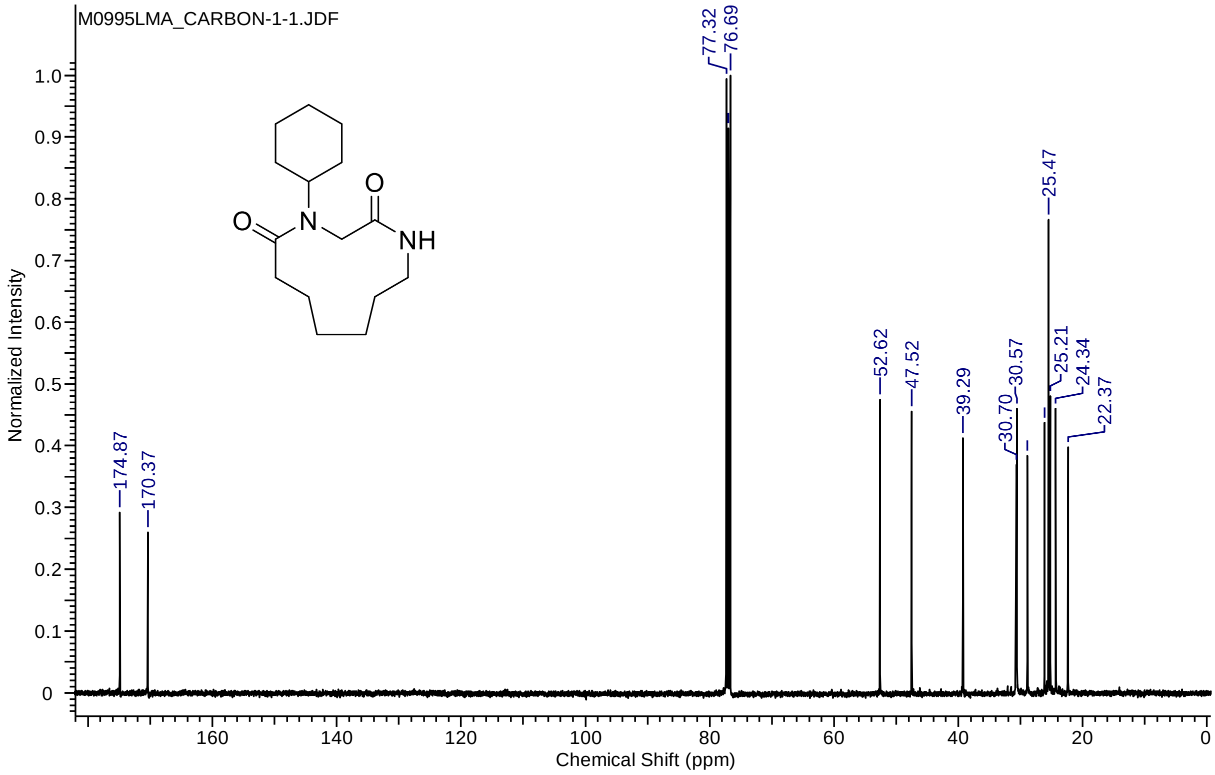

Compound 27g

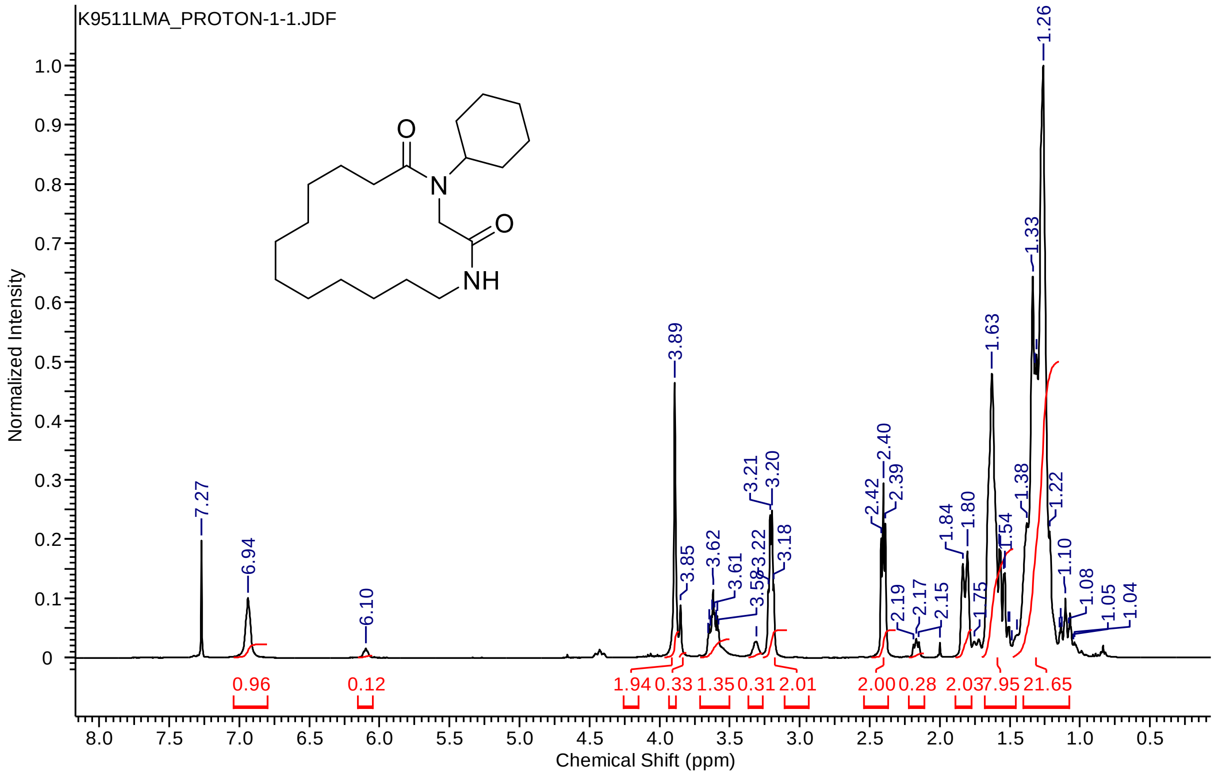

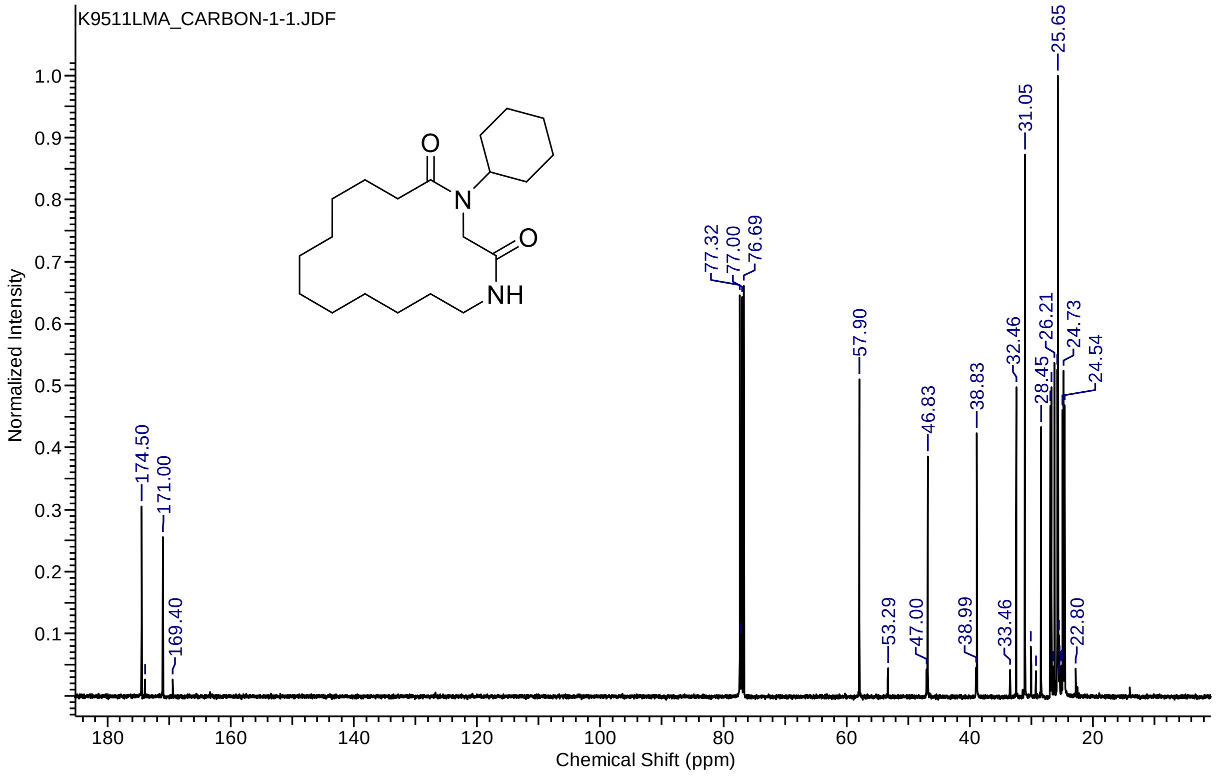

Compound 27h

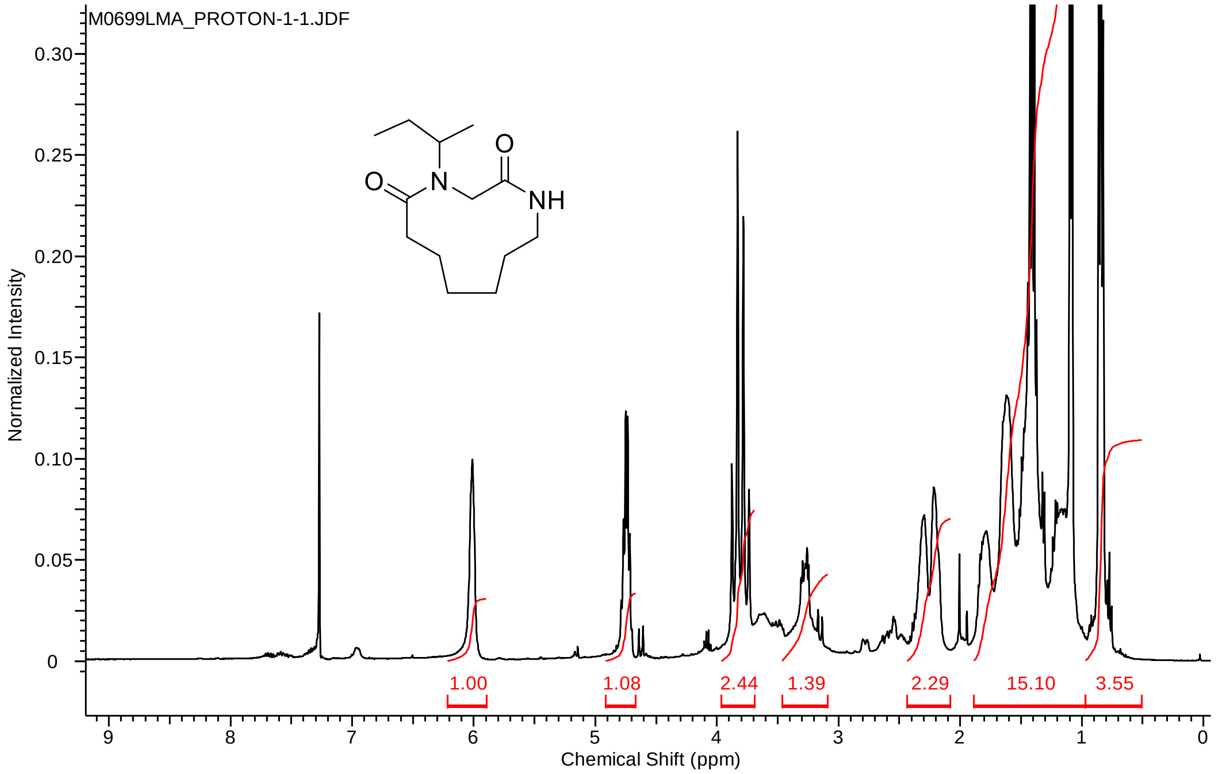

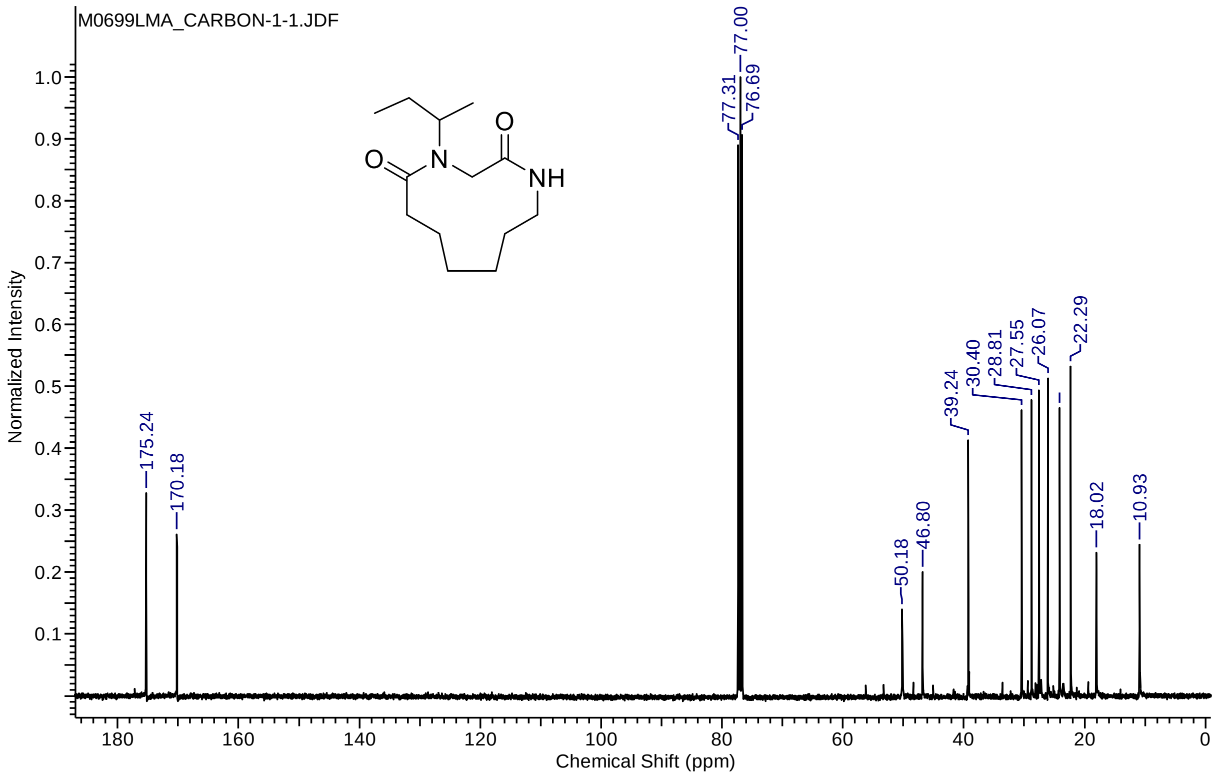

Compound 27i

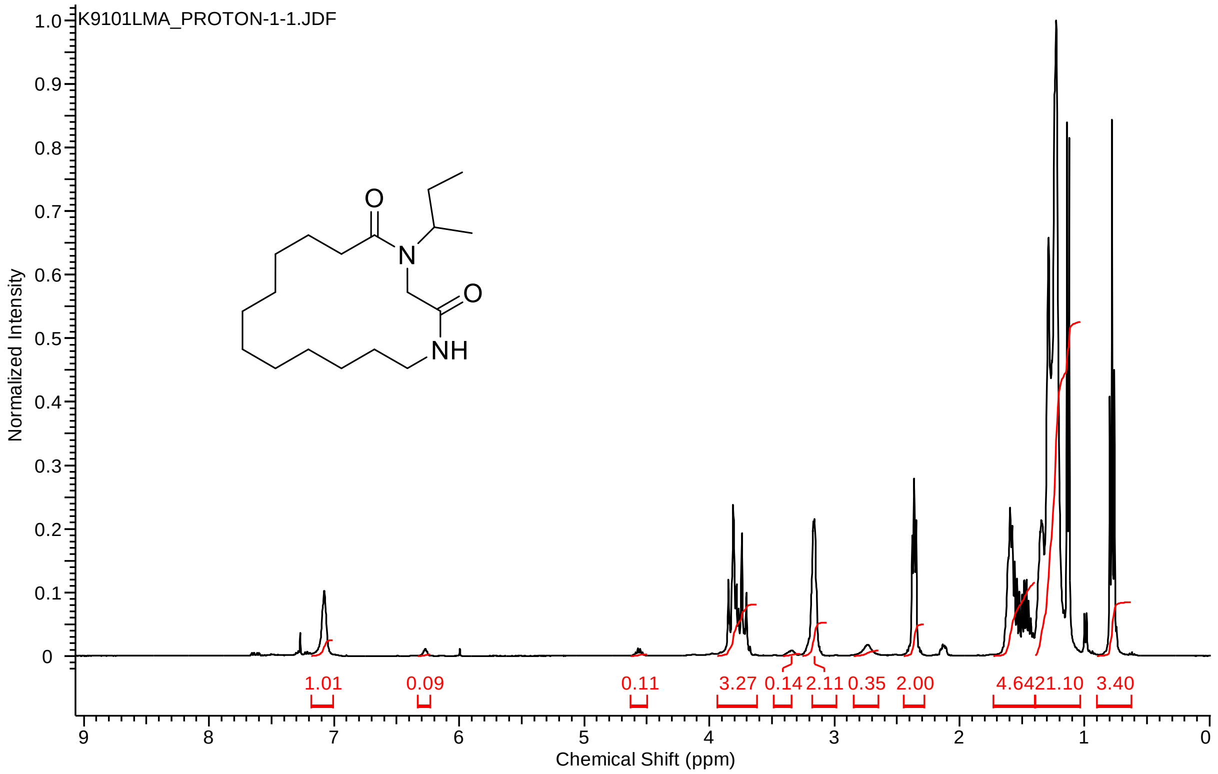

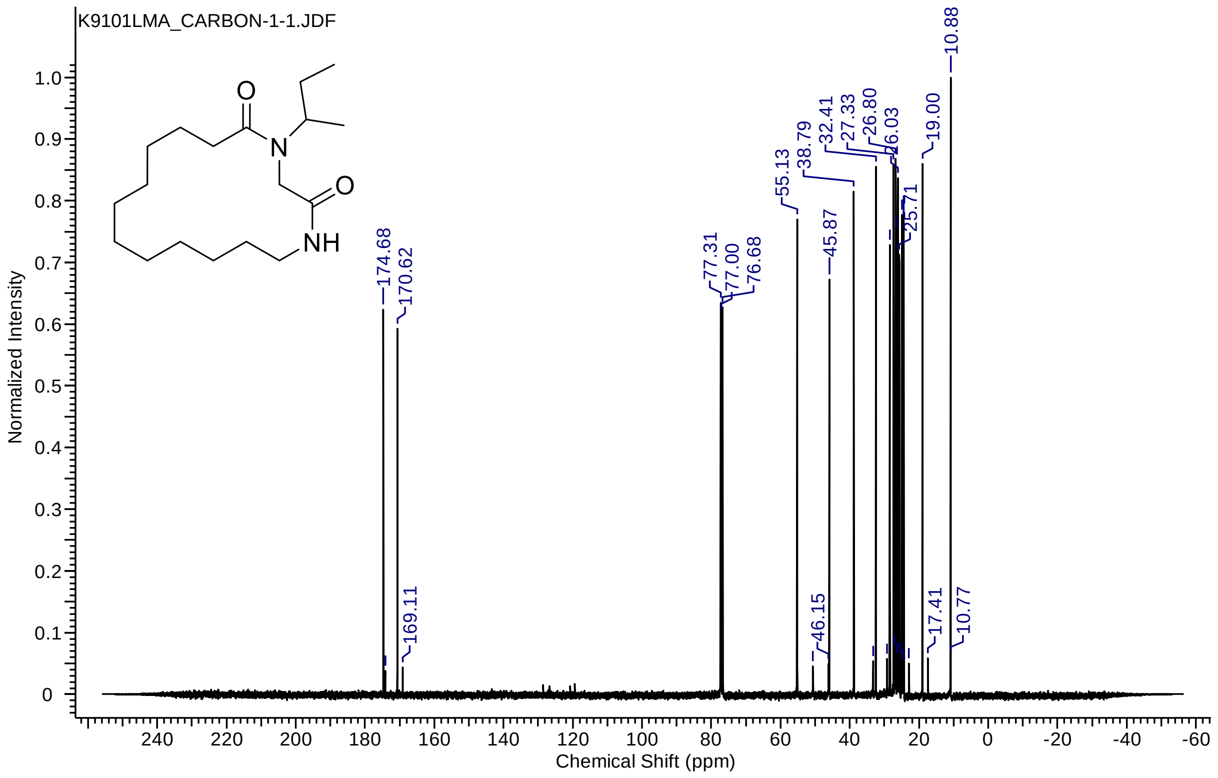

Compound 27j

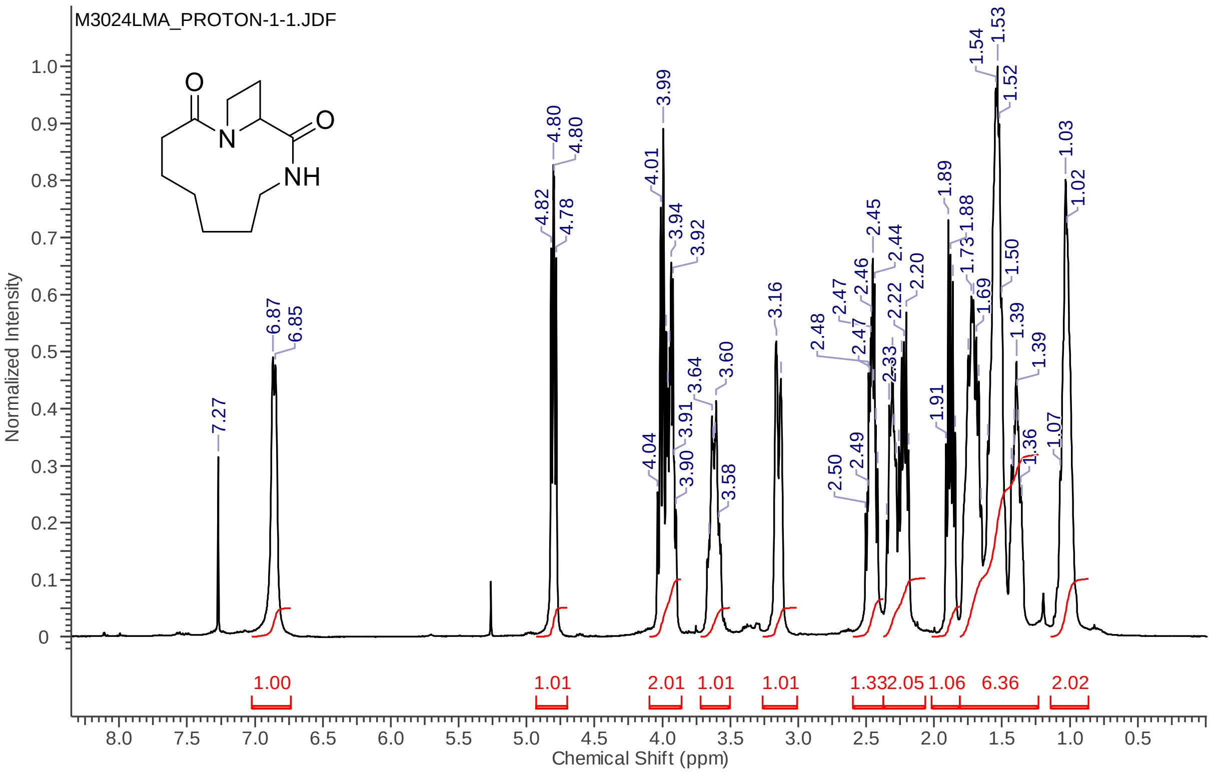

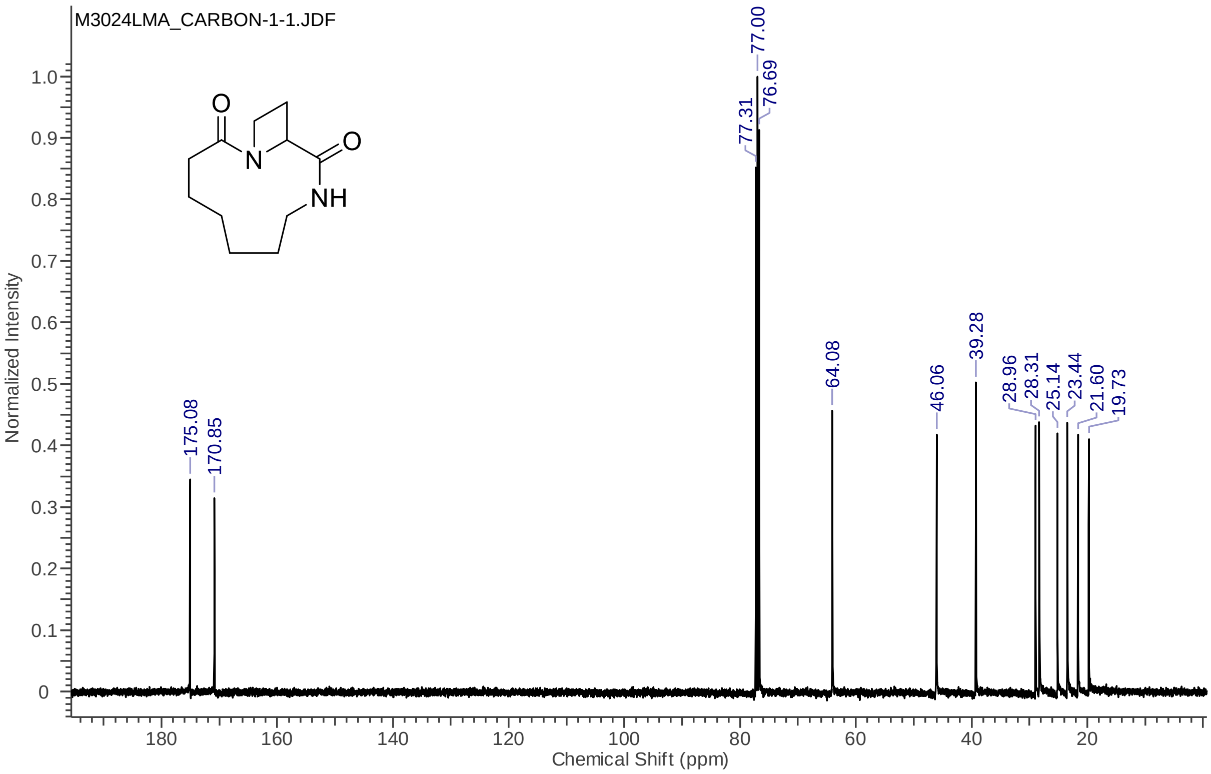

Compound 27l

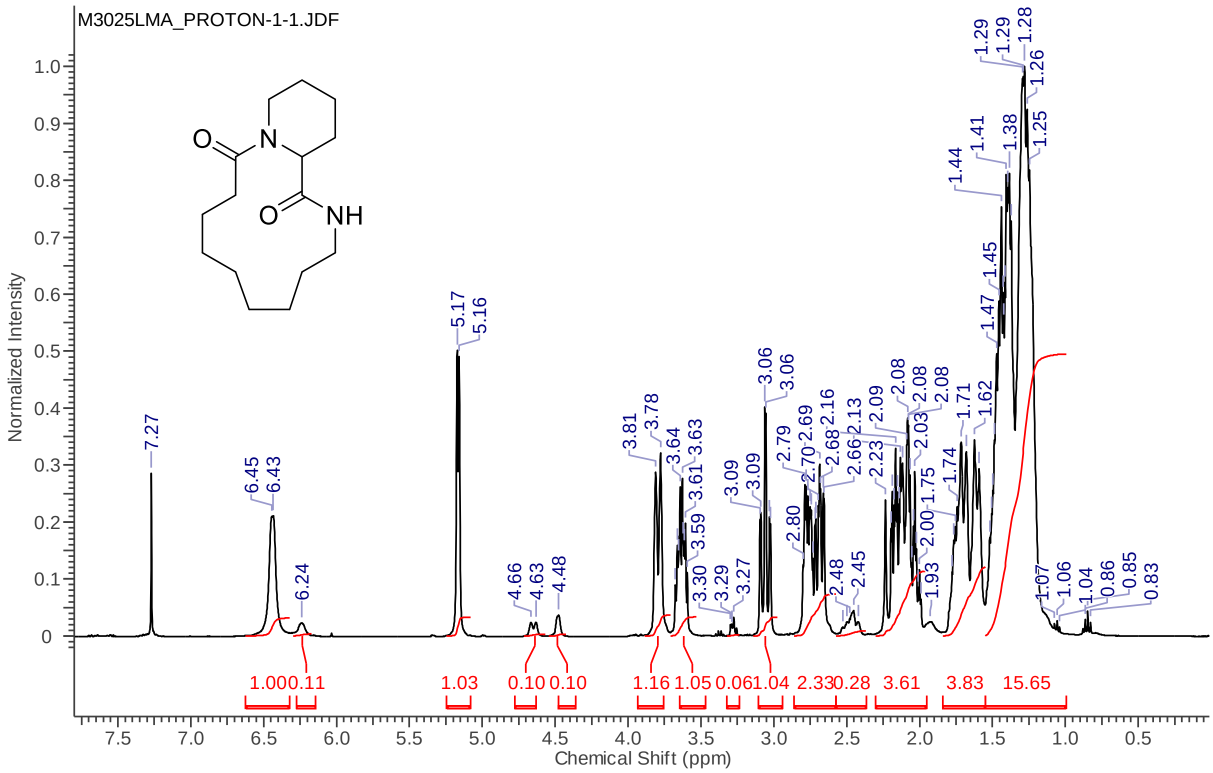

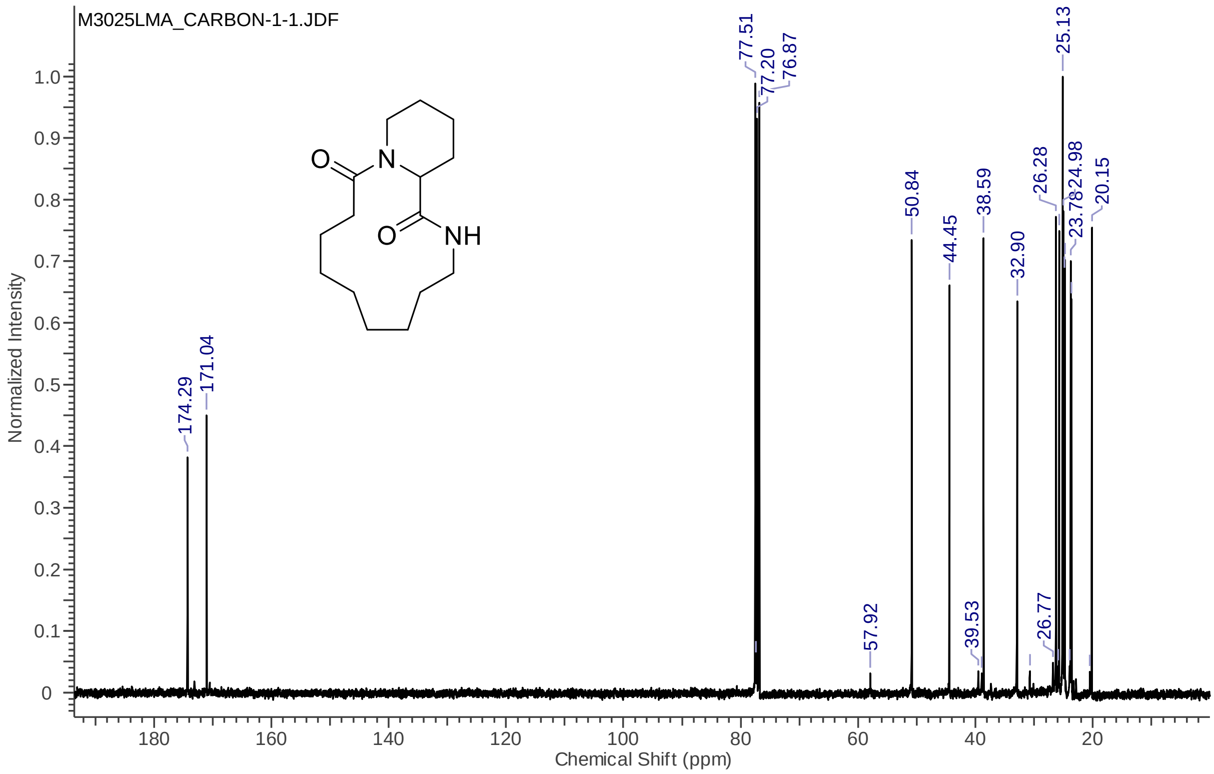

Compound 27m

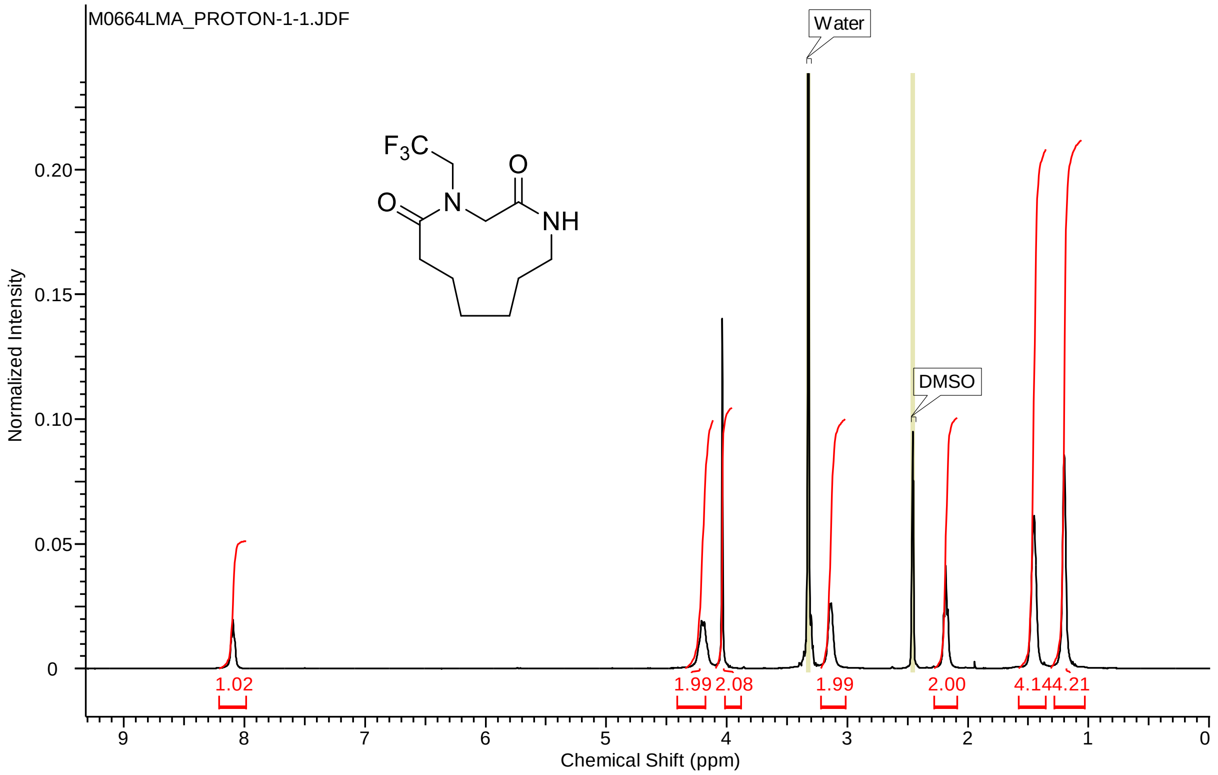
M0664LMA_PROTON-1-1.JDF
Water
DMSO
1.02
1.99
2.08
1.99
2.00
4.14
4.21
Normalized Intensity
Chemical Shift (ppm)

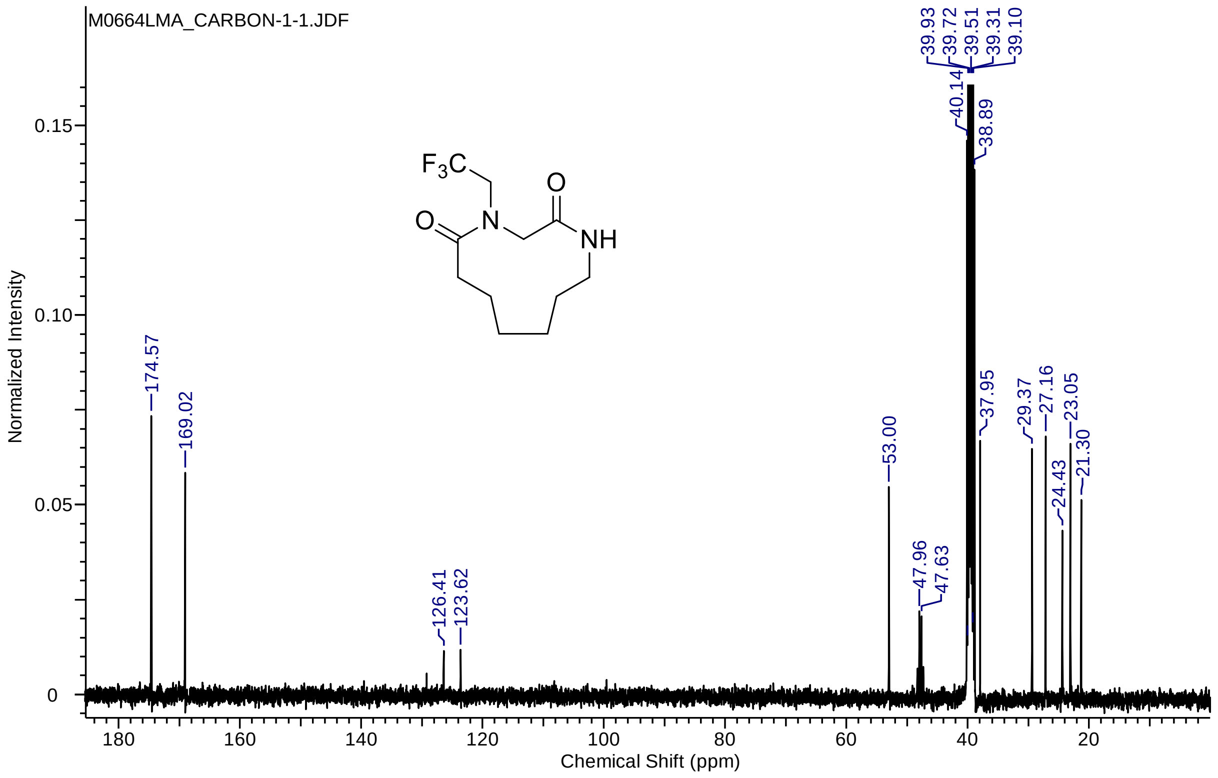
M0664LMA_CARBON-1-1.JDF
174.57
169.02
126.41
123.62
53.00
47.96
47.63
40.14
39.93
39.72
39.51
39.31
39.10
38.89
37.95
29.37
27.16
24.43
23.05
21.30
Normalized Intensity
Chemical Shift (ppm)

Compound 27n

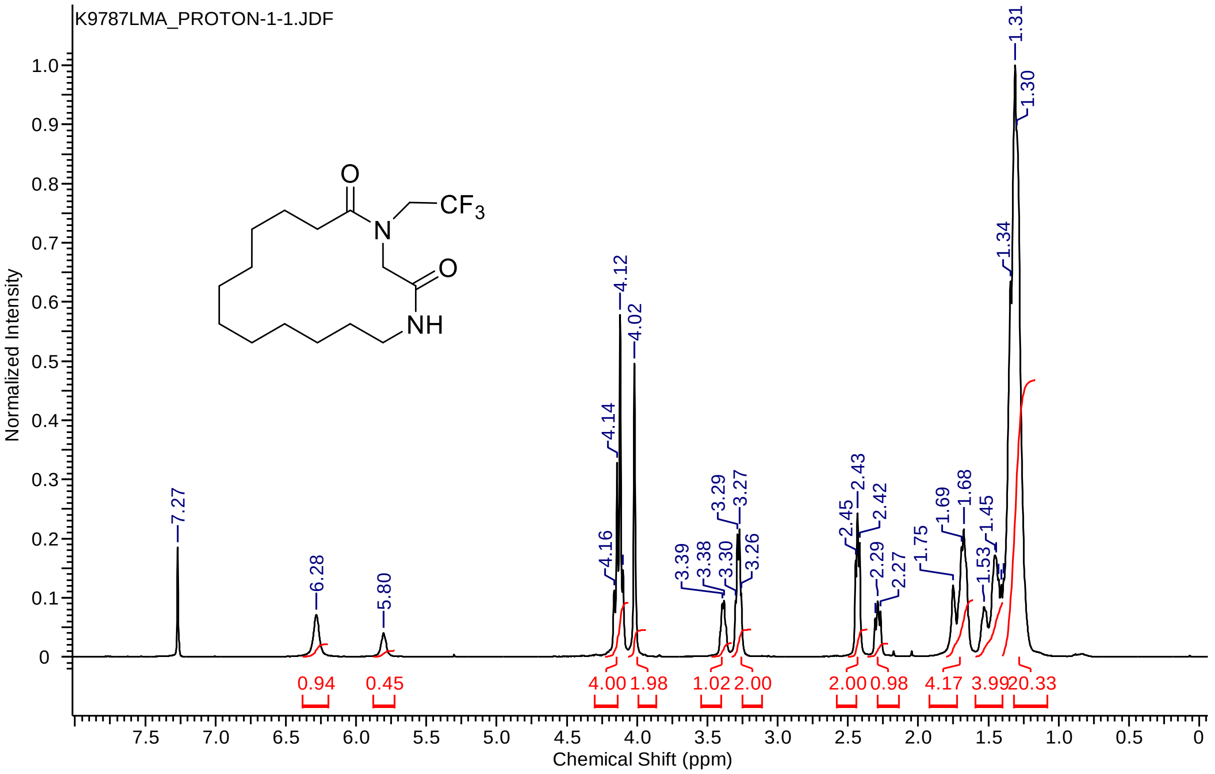

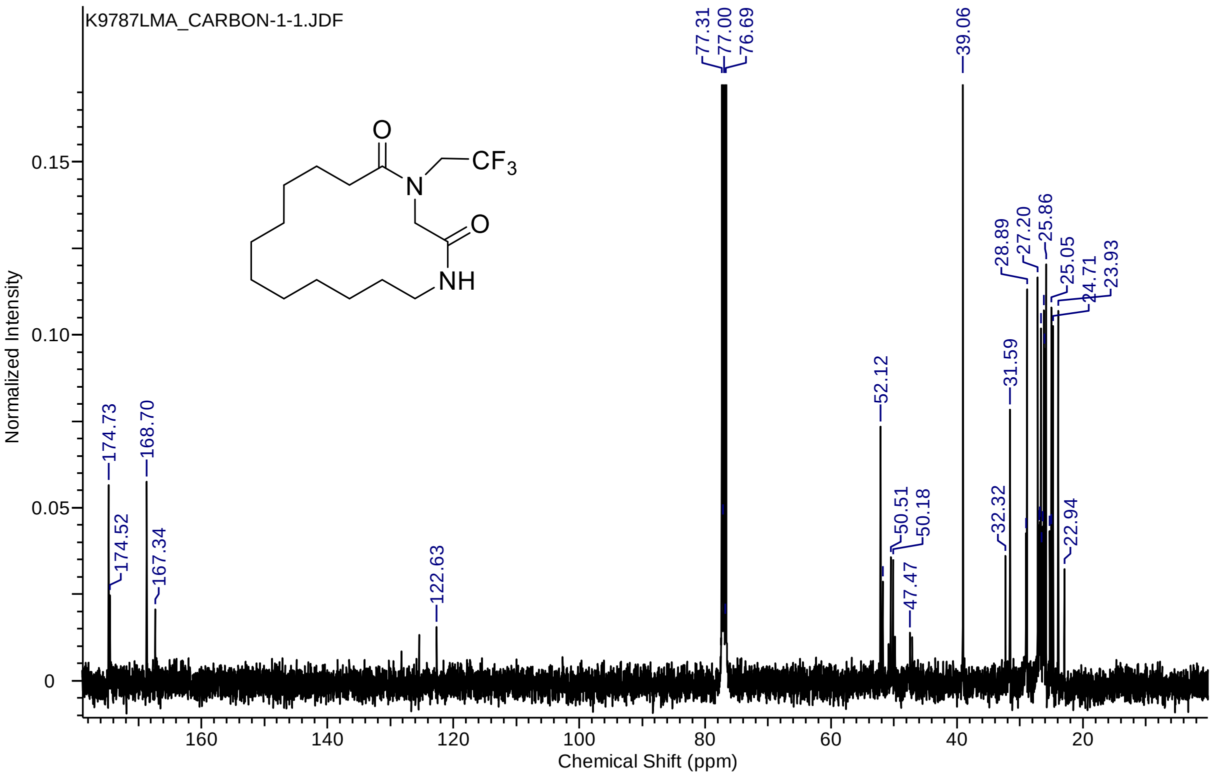

Compound 27o

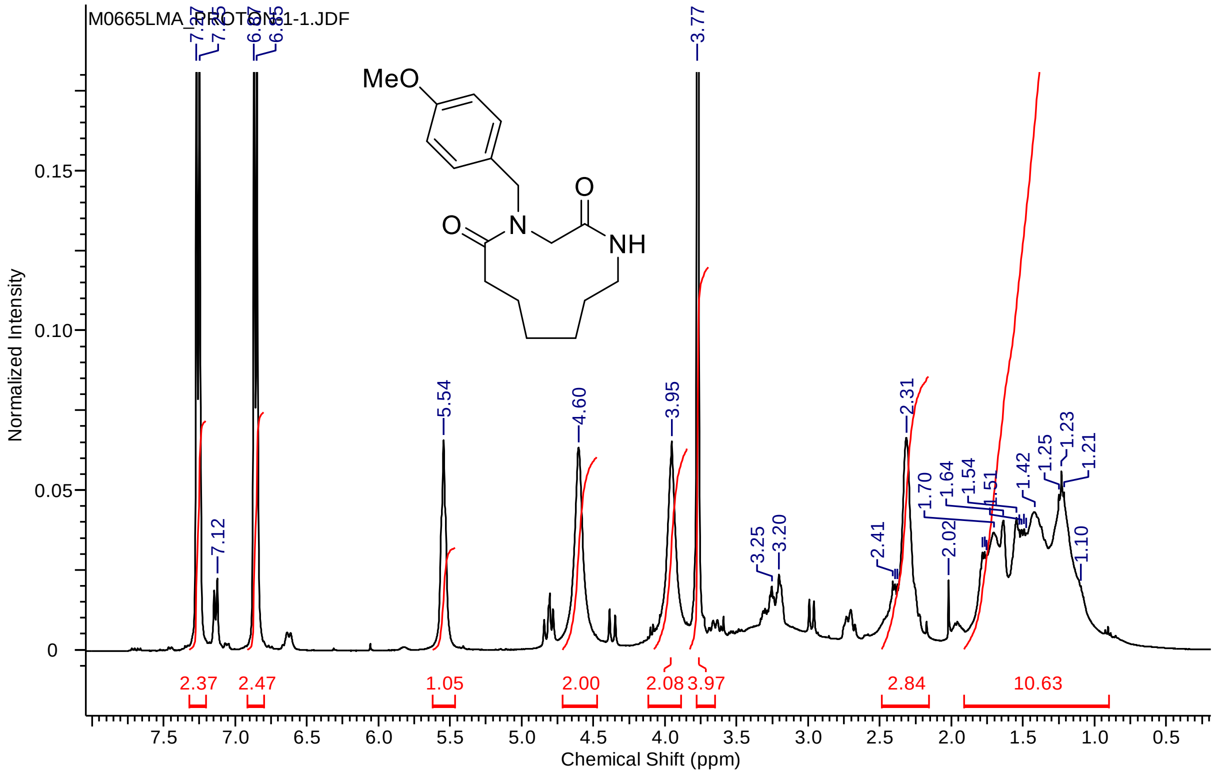

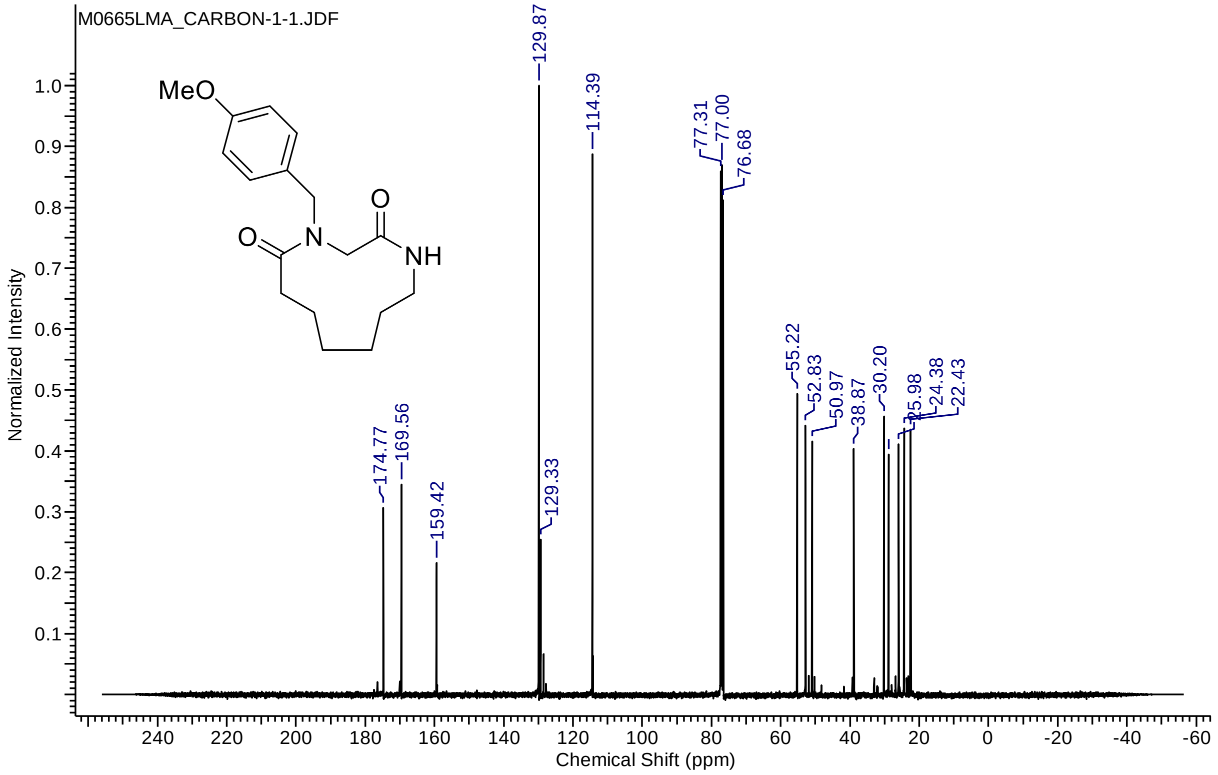

Compound 27p

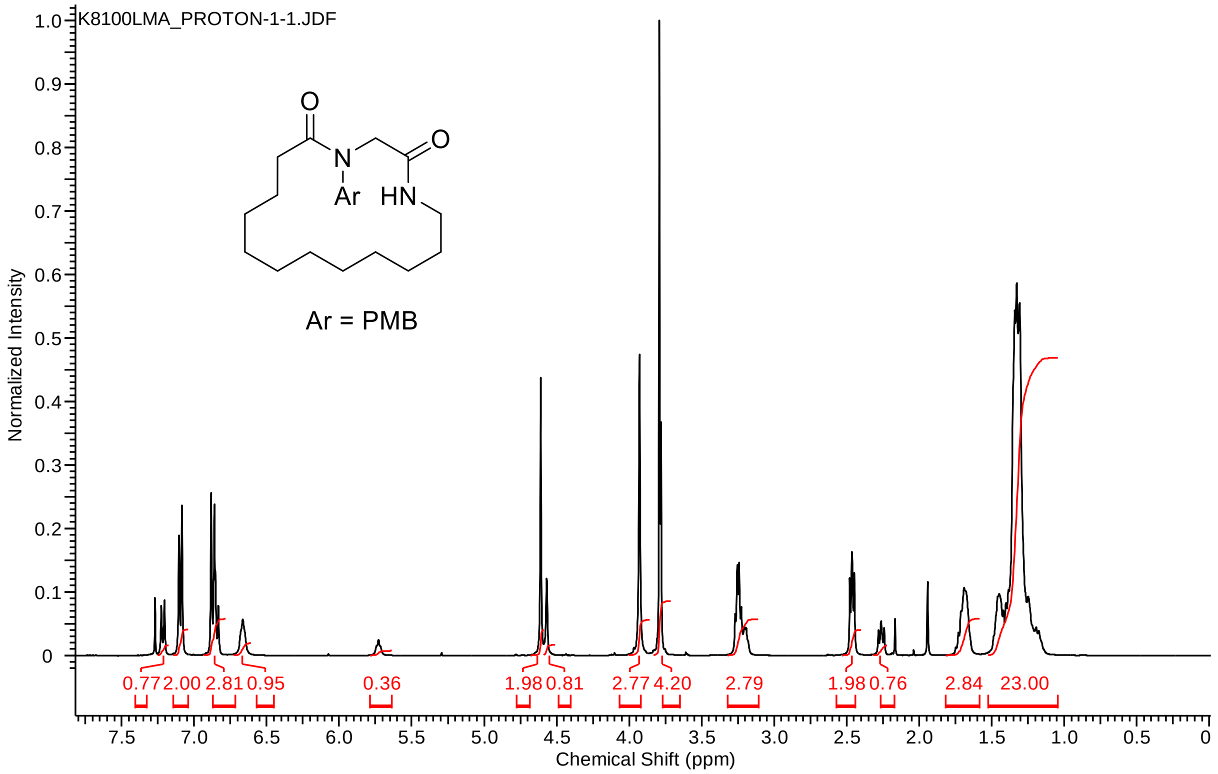

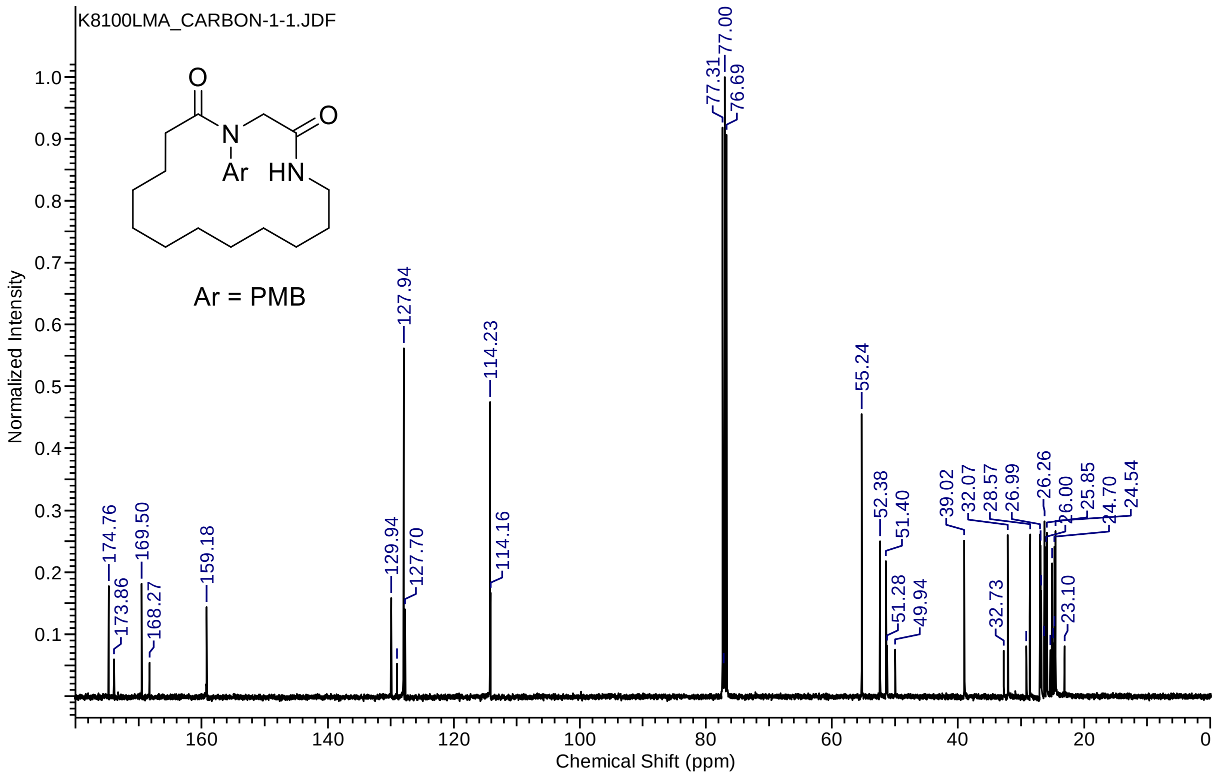

Compound 27q

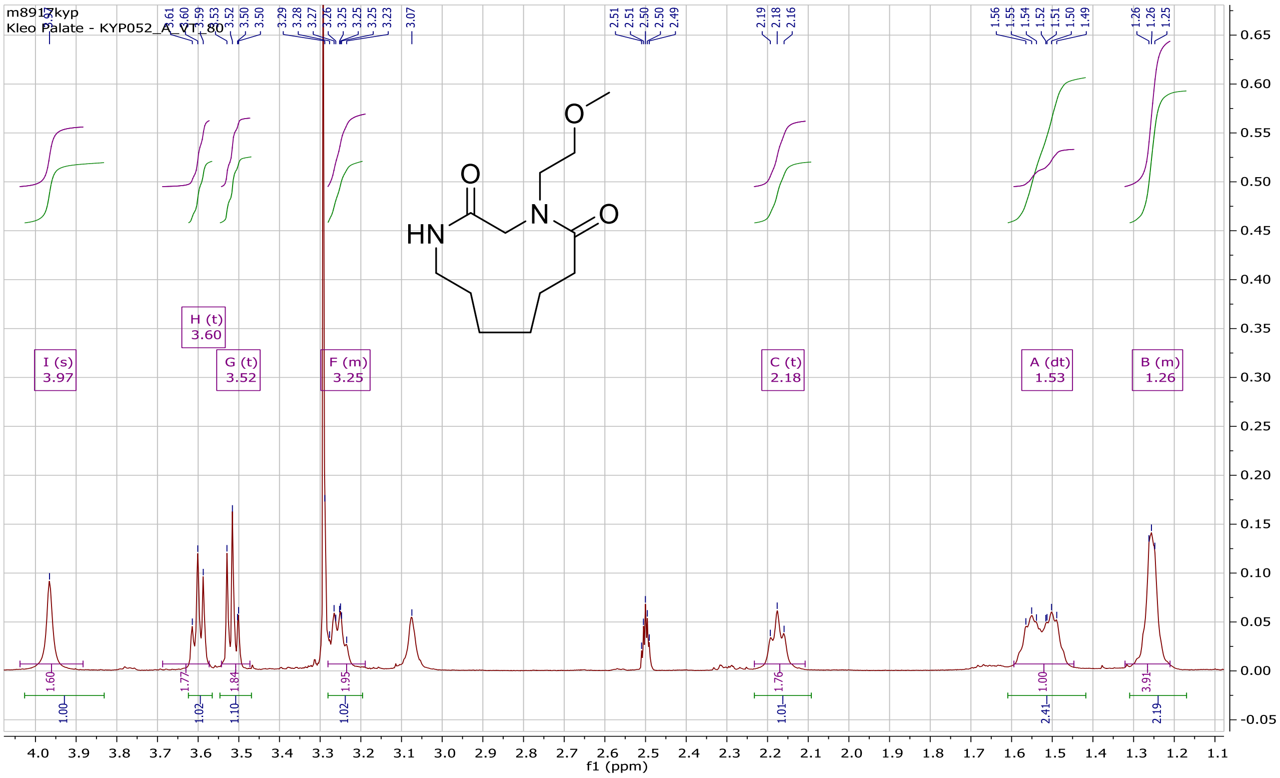

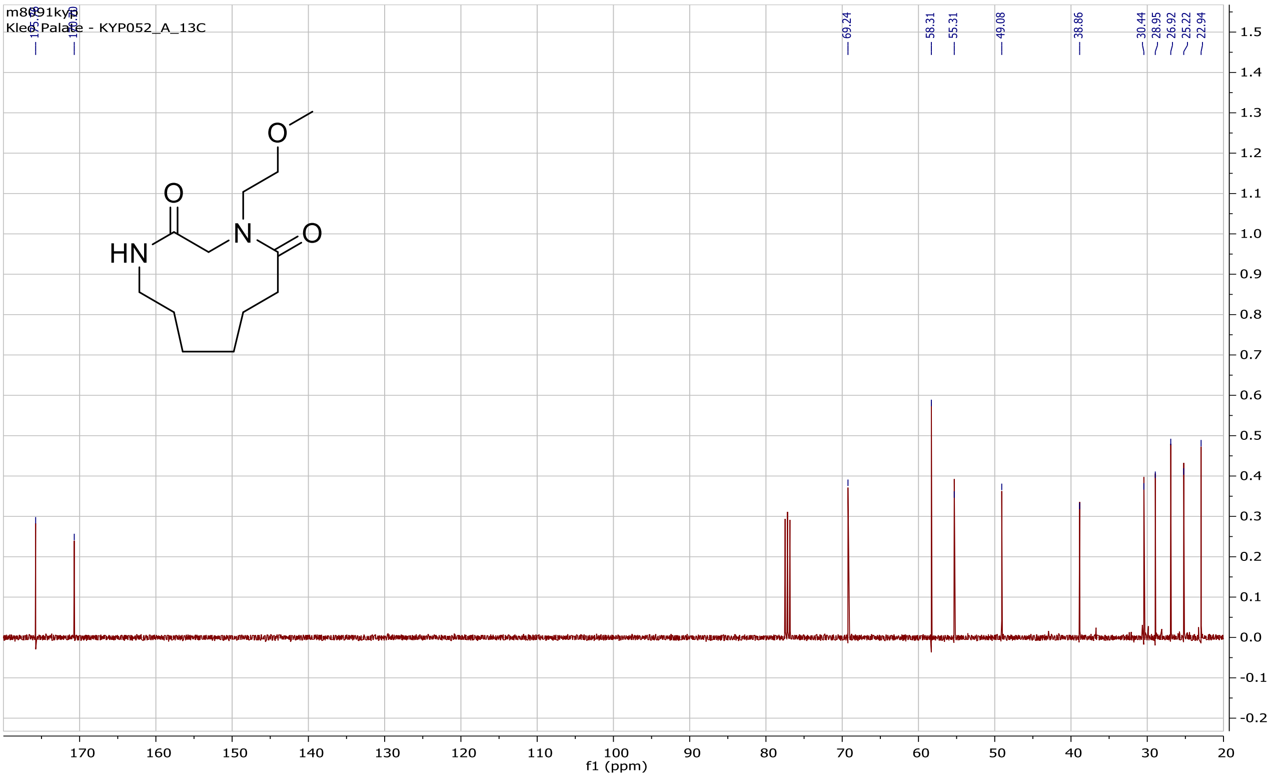

Compound 27r

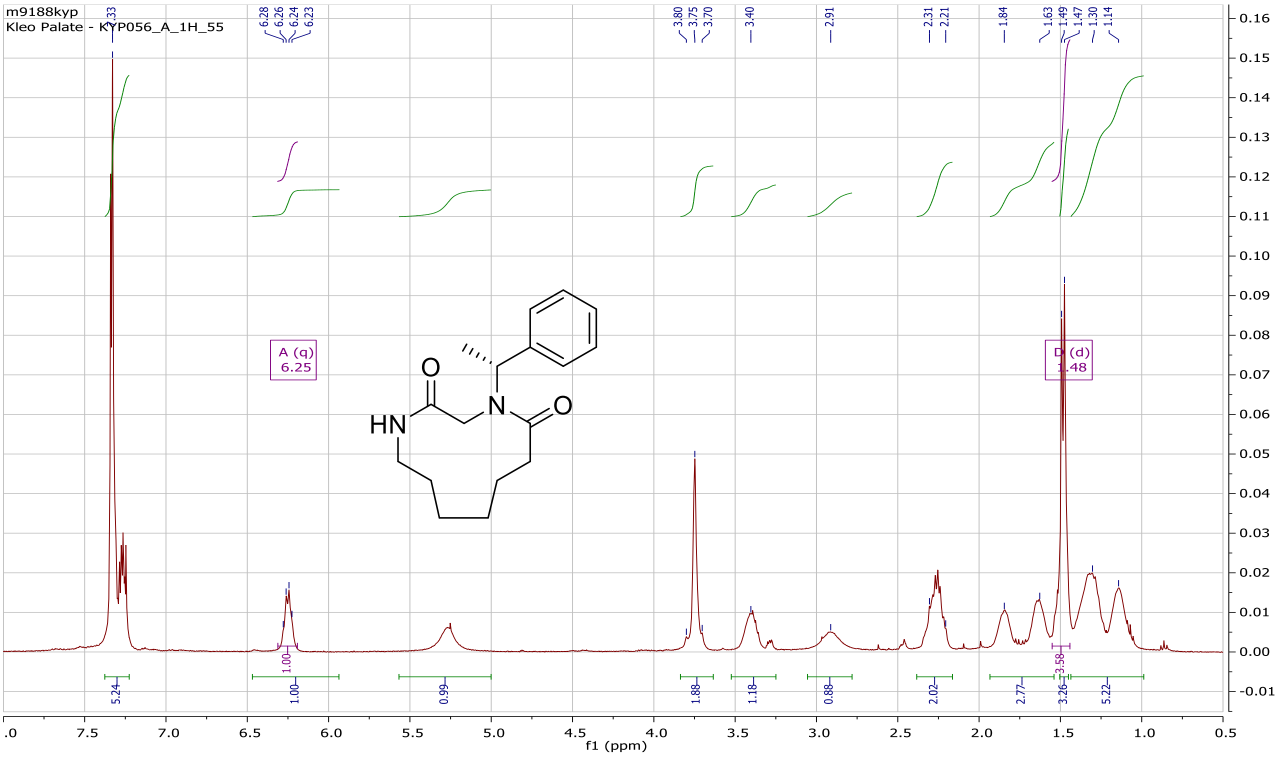

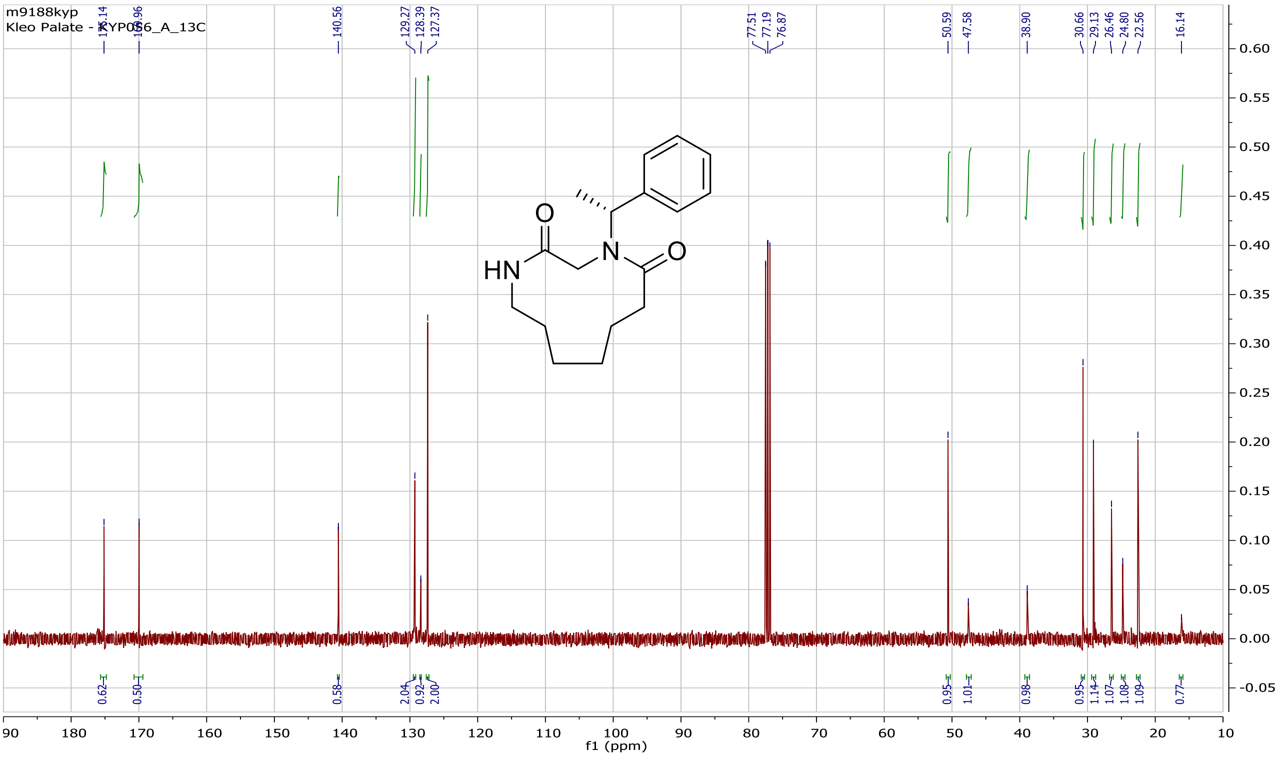

Compound 27s

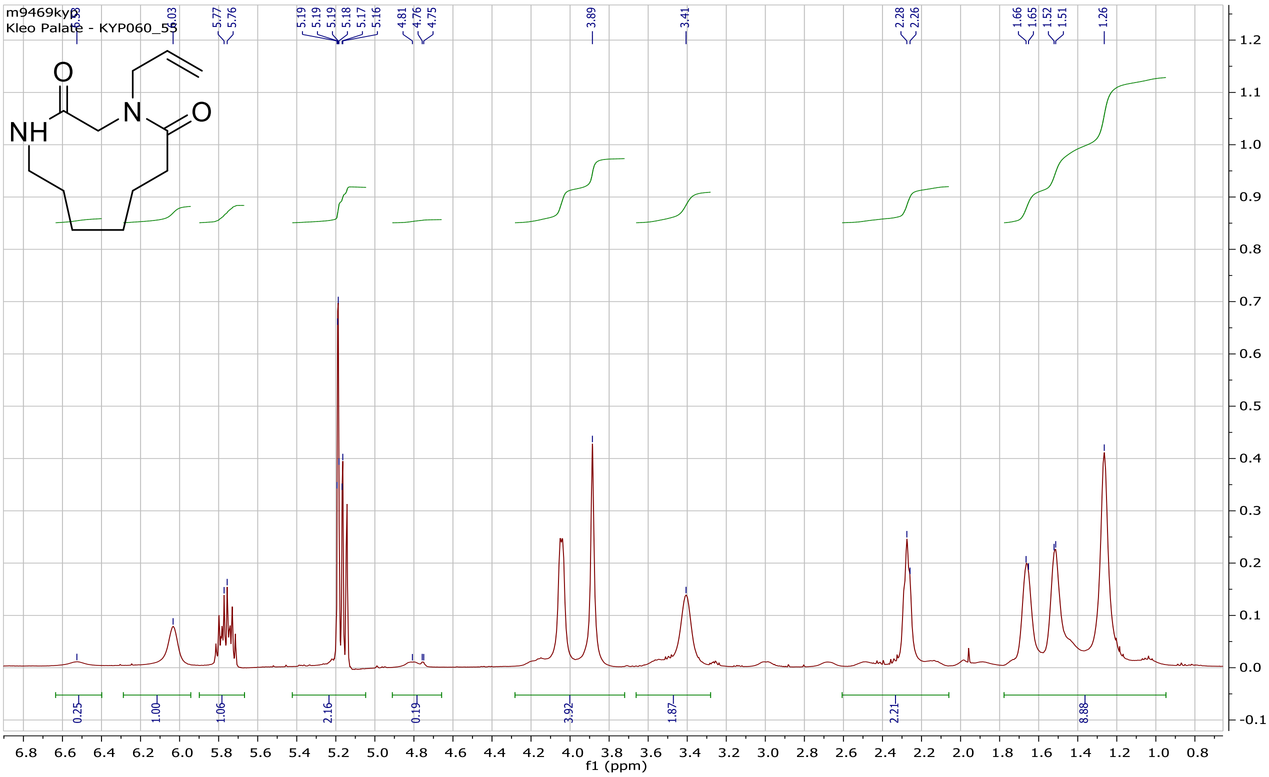

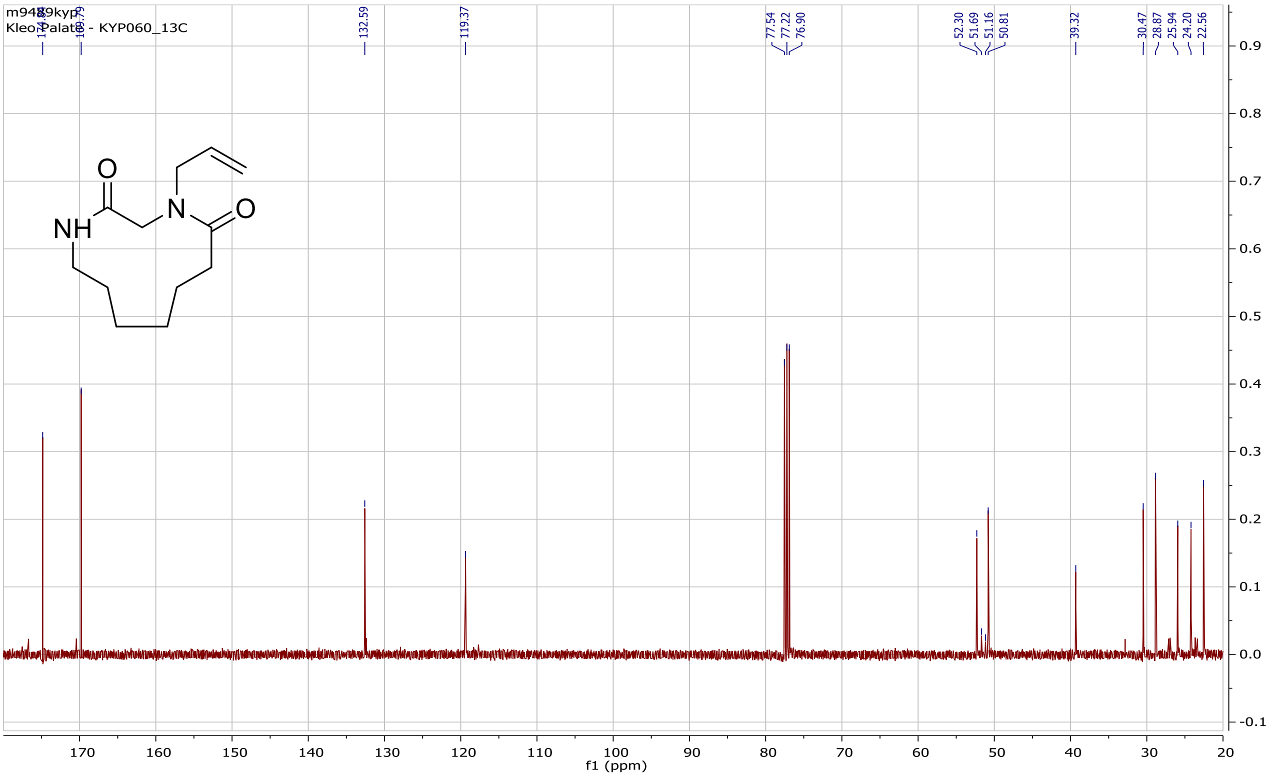

Compound 27t

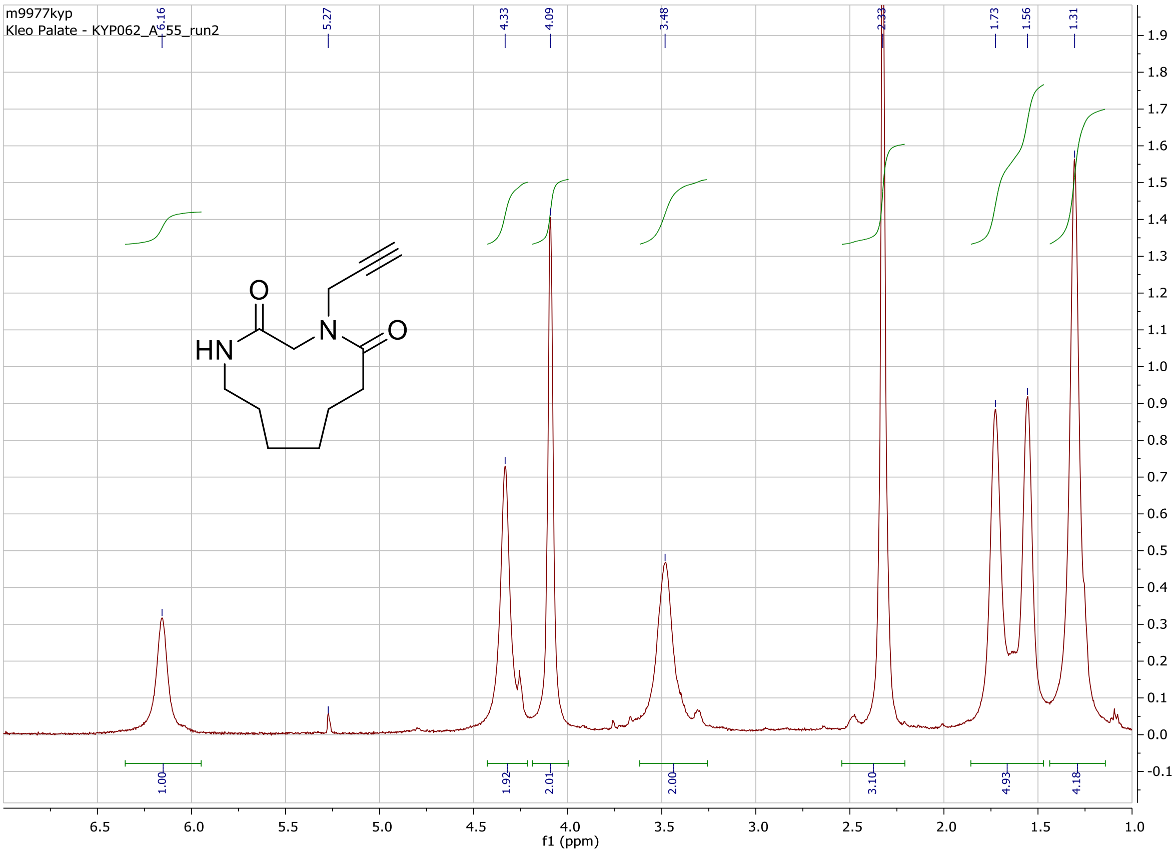

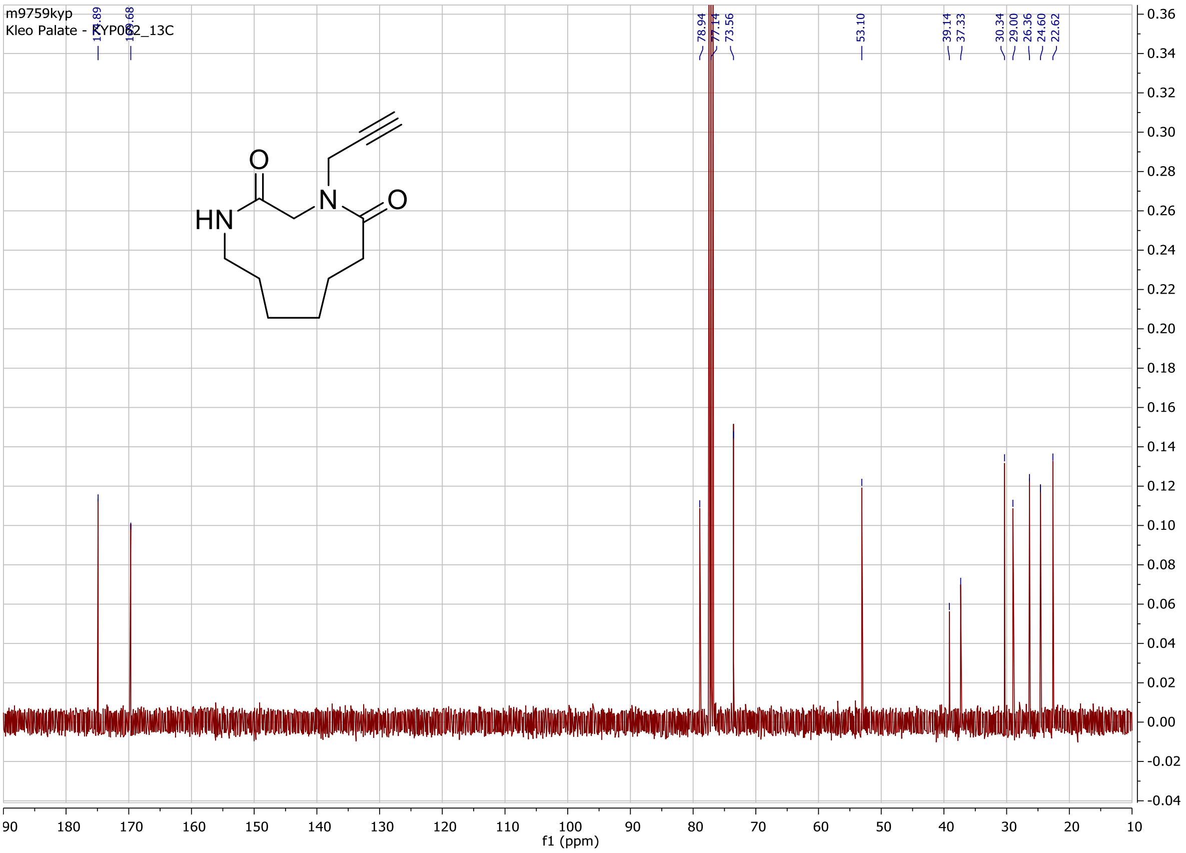

Compound 27u

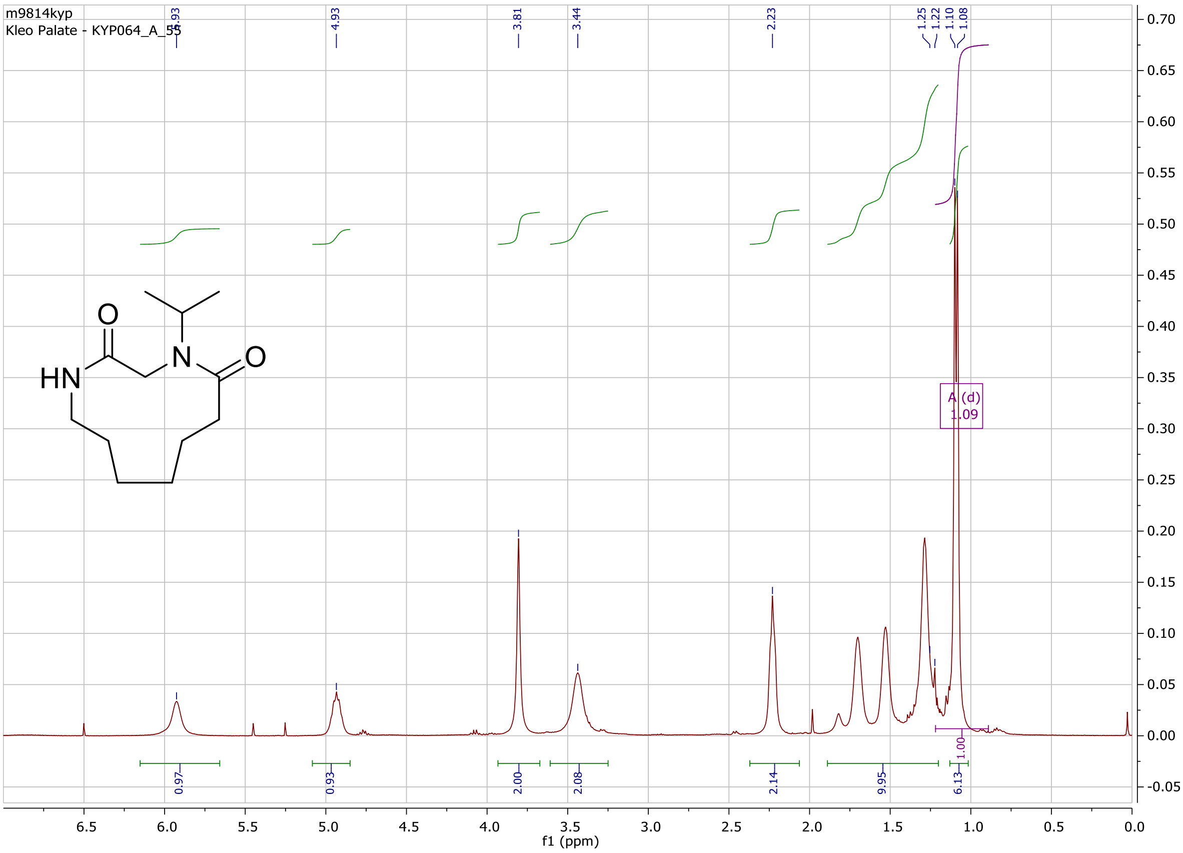

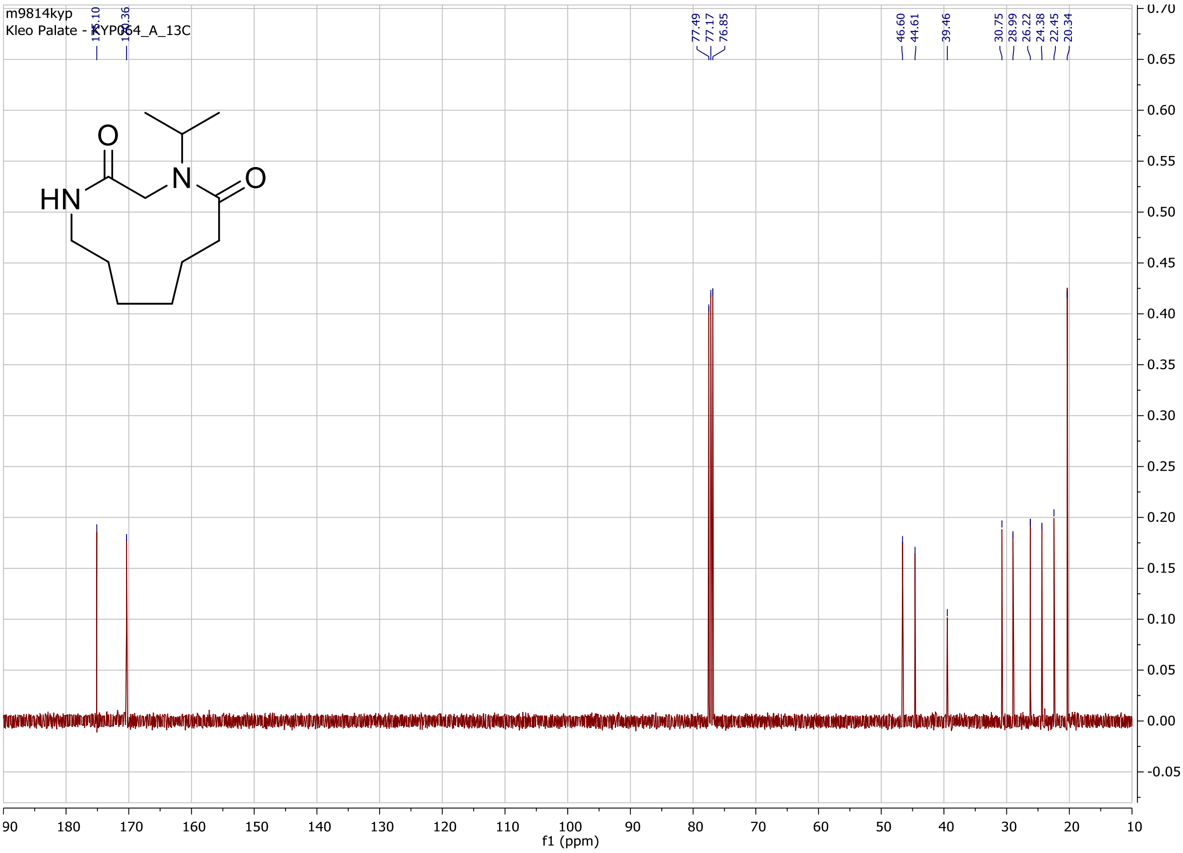

Compound 27v

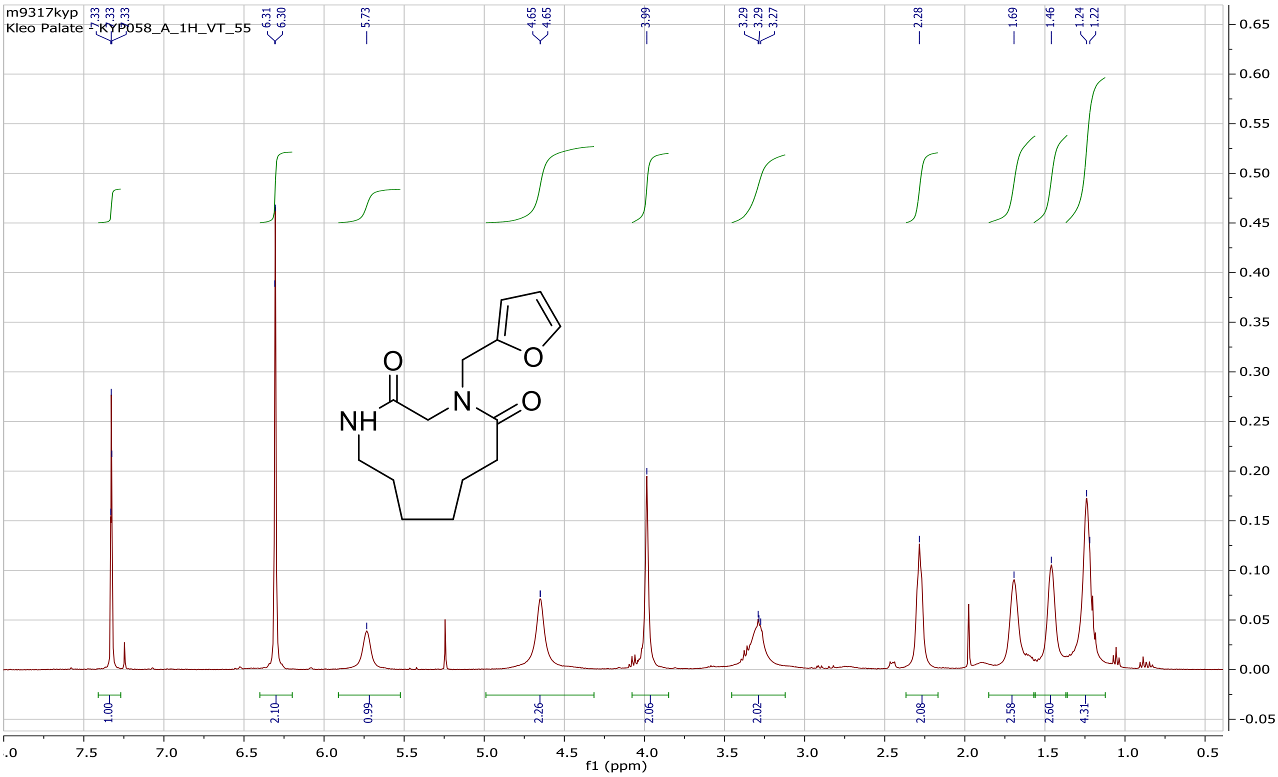

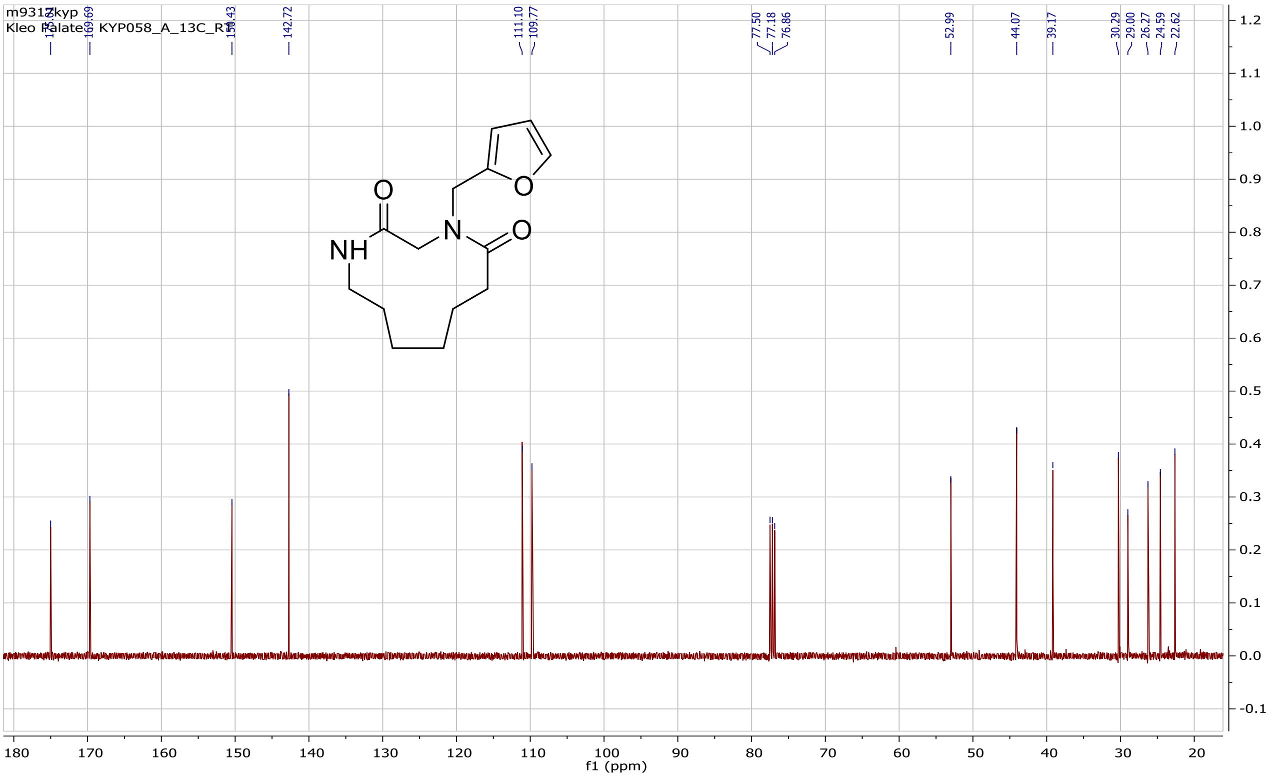

Compound 27w

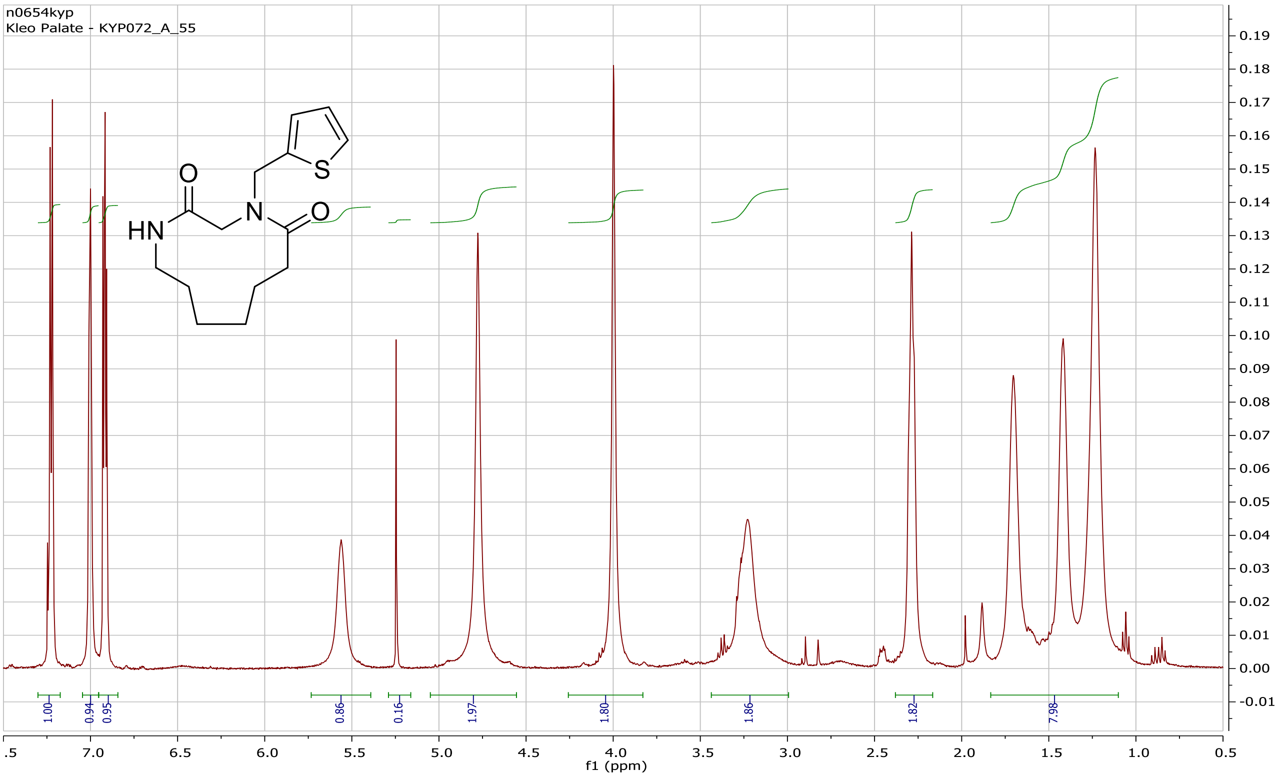

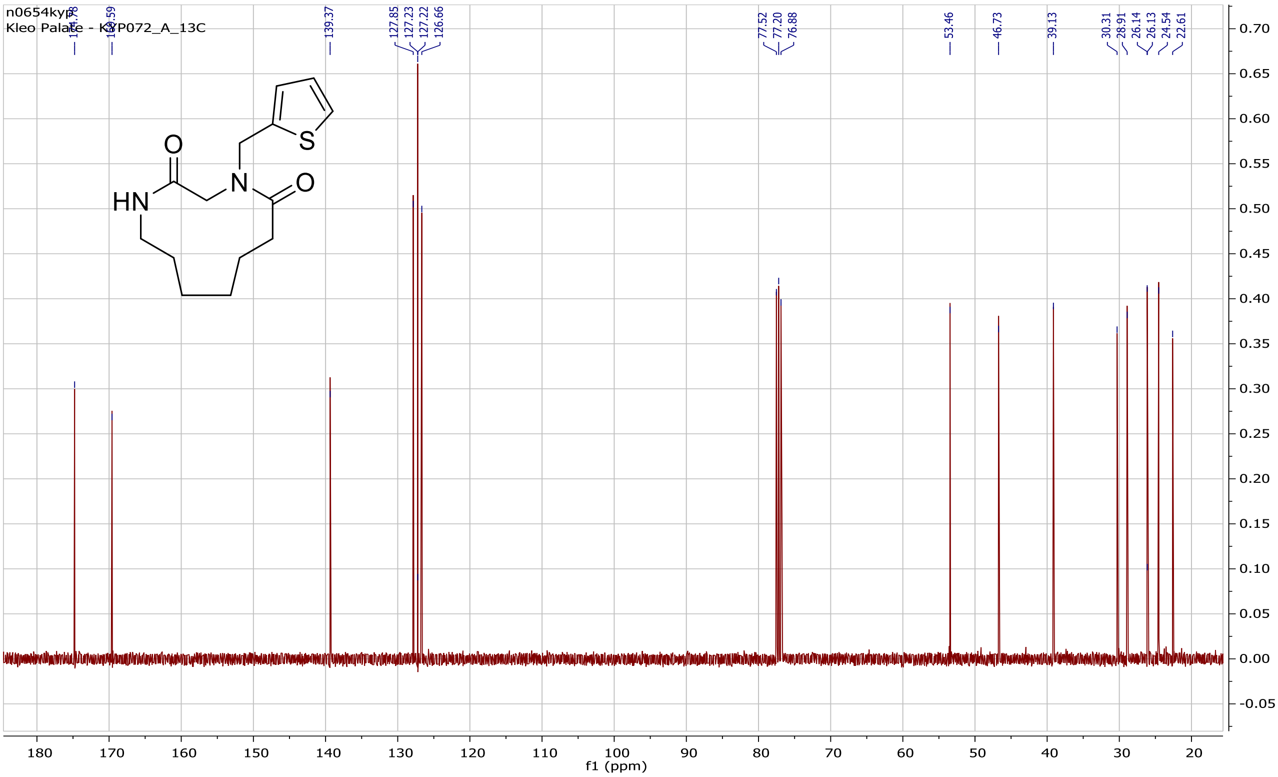

Compound 27x

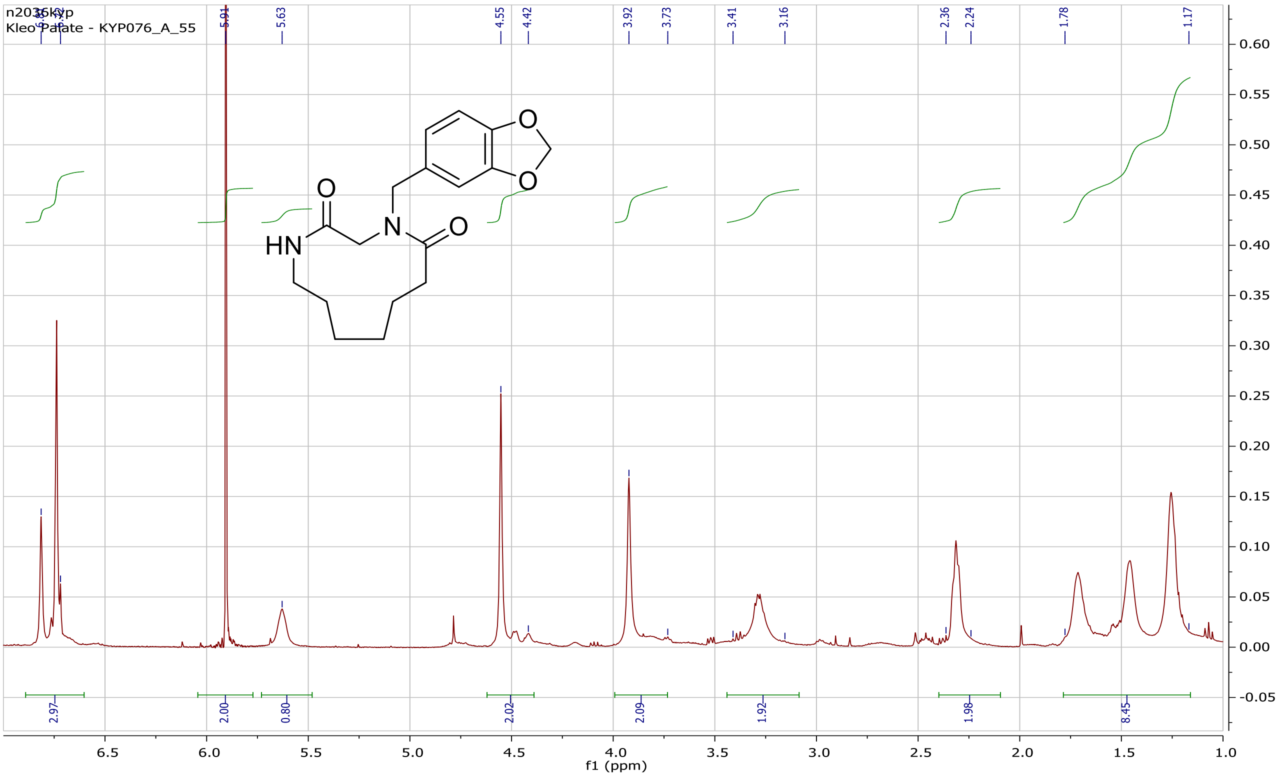

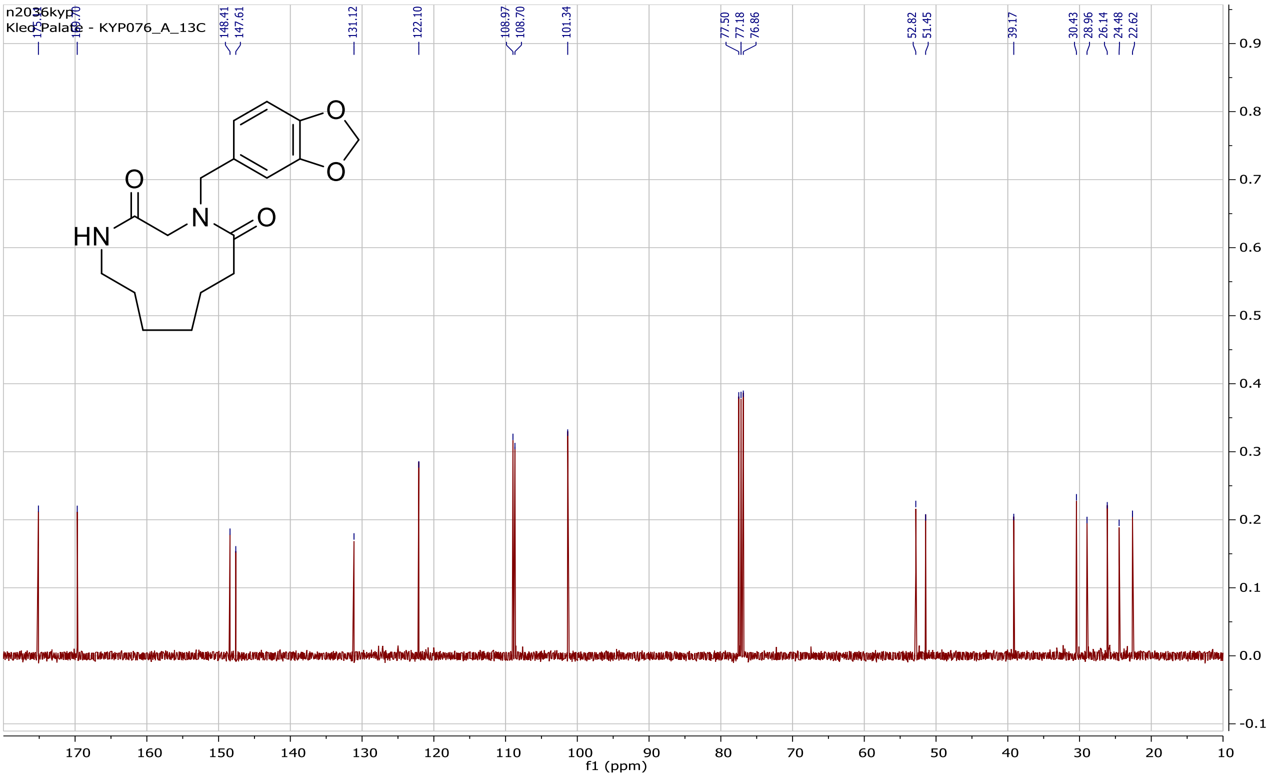

Compound 27y

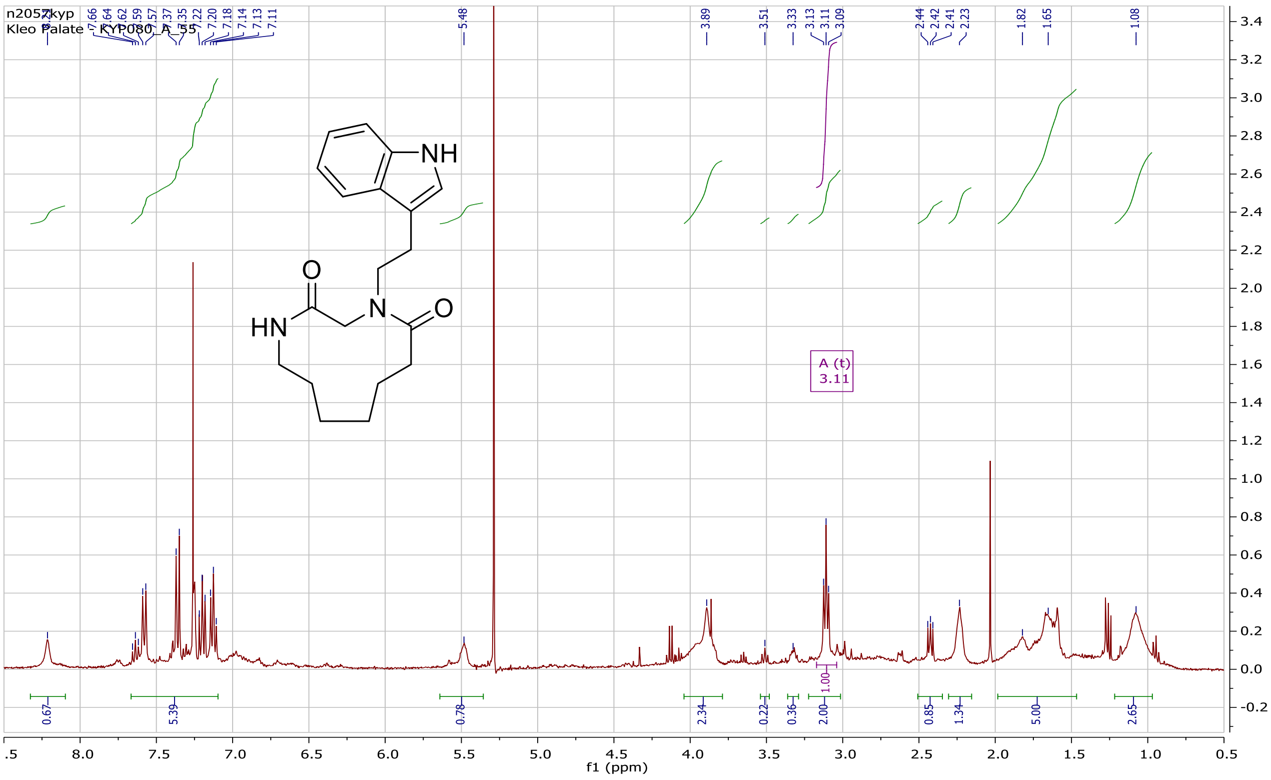

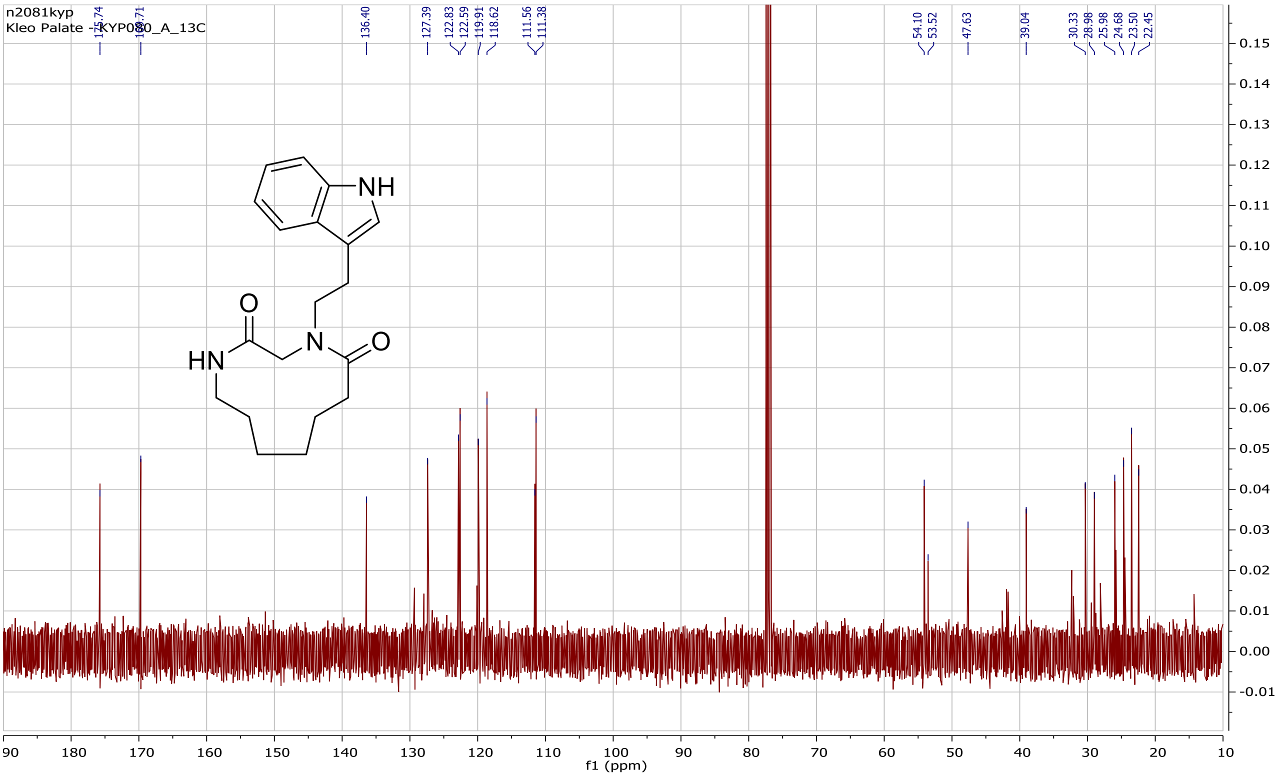

Compound 34

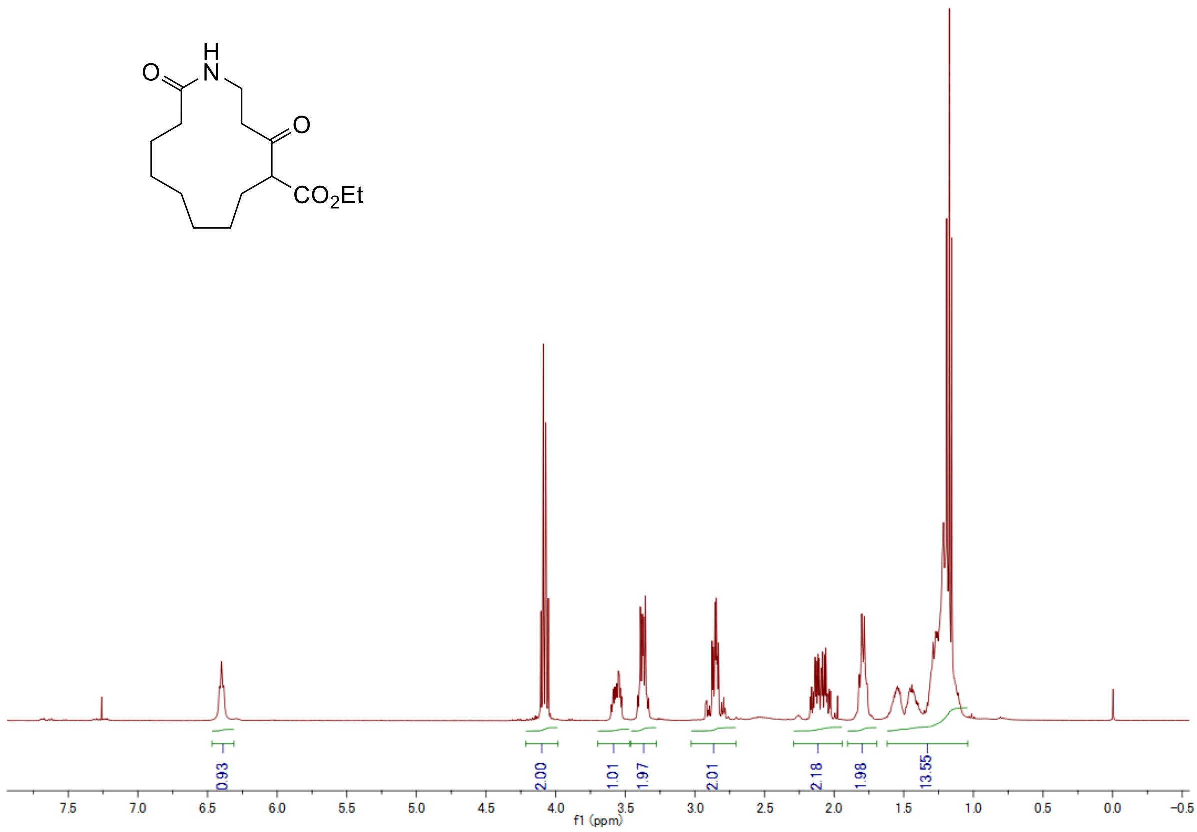

H
N
O
O
CO2Et
0.93
2.00
1.01
1.97
2.01
2.18
1.98
13.55
7.5
7.0
6.5
6.0
5.5
5.0
4.5
4.0
3.5
3.0
2.5
2.0
1.5
1.0
0.5
0.0
−0.5
f1 (ppm)

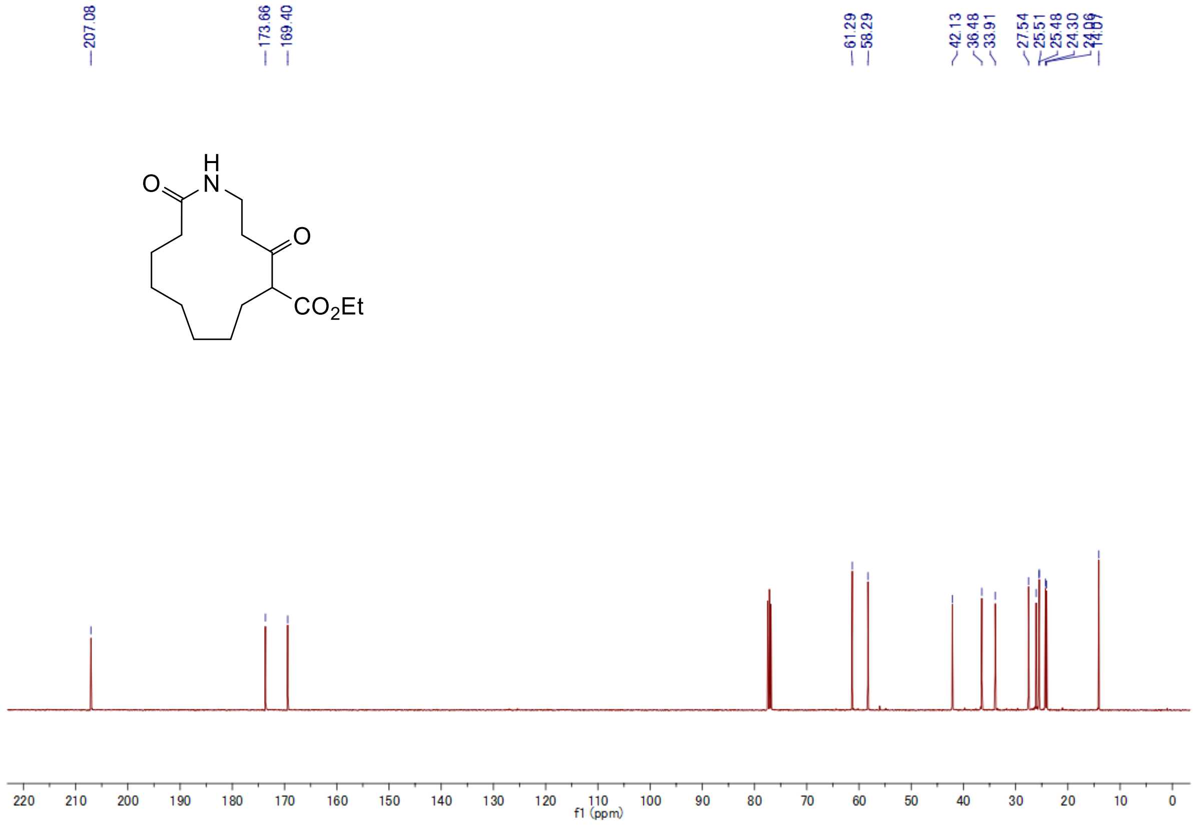

207.08
173.66
169.40
61.29
58.29
42.13
36.48
33.91
27.54
25.51
25.48
24.30
24.06
14.07
H
N
O
O
CO2Et
220
210
200
190
180
170
160
150
140
130
120
110
100
90
80
70
60
50
40
30
20
10
0
f1 (ppm)

Compound 35

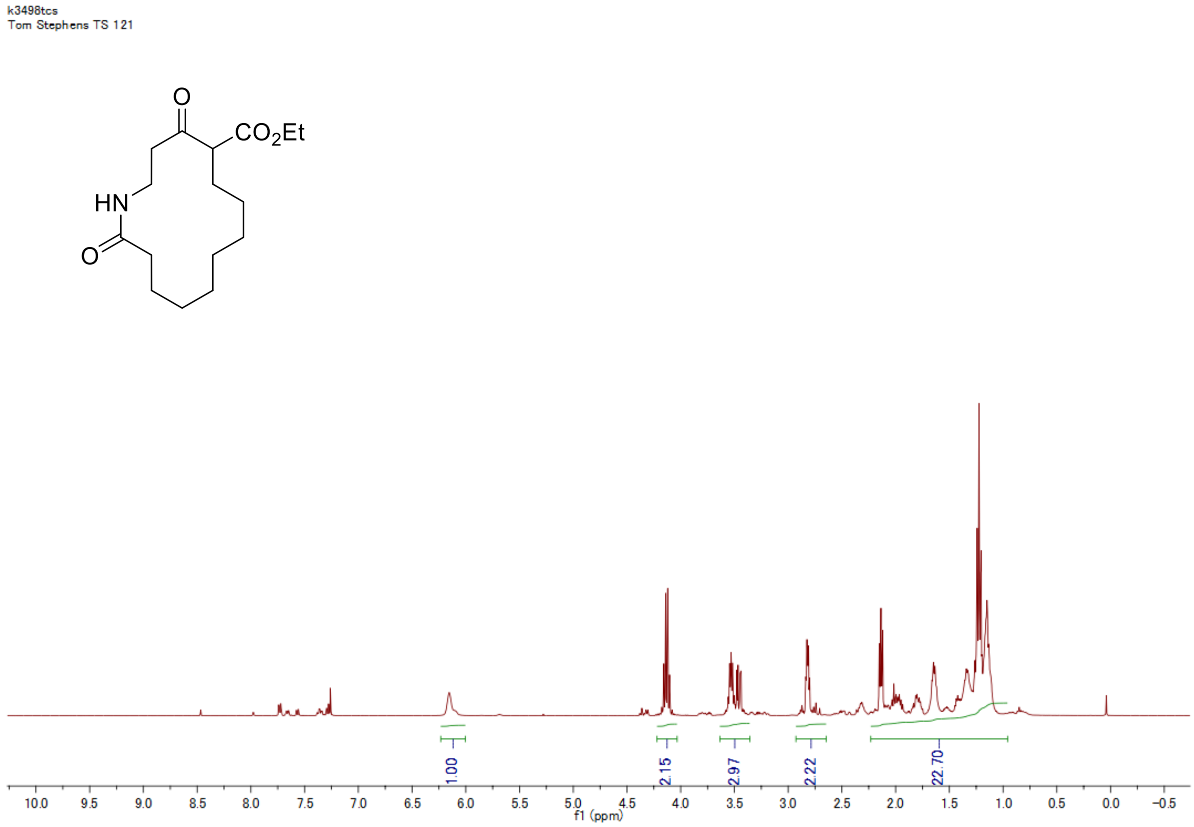
k3498tcs
Tom Stephens TS 121
O
CO_2Et
HN
O
1.00
2.15
2.97
2.22
22.70
10.0 9.5 9.0 8.5 8.0 7.5 7.0 6.5 6.0 5.5 5.0 4.5 4.0 3.5 3.0 2.5 2.0 1.5 1.0 0.5 0.0 −0.5
f1 (ppm)

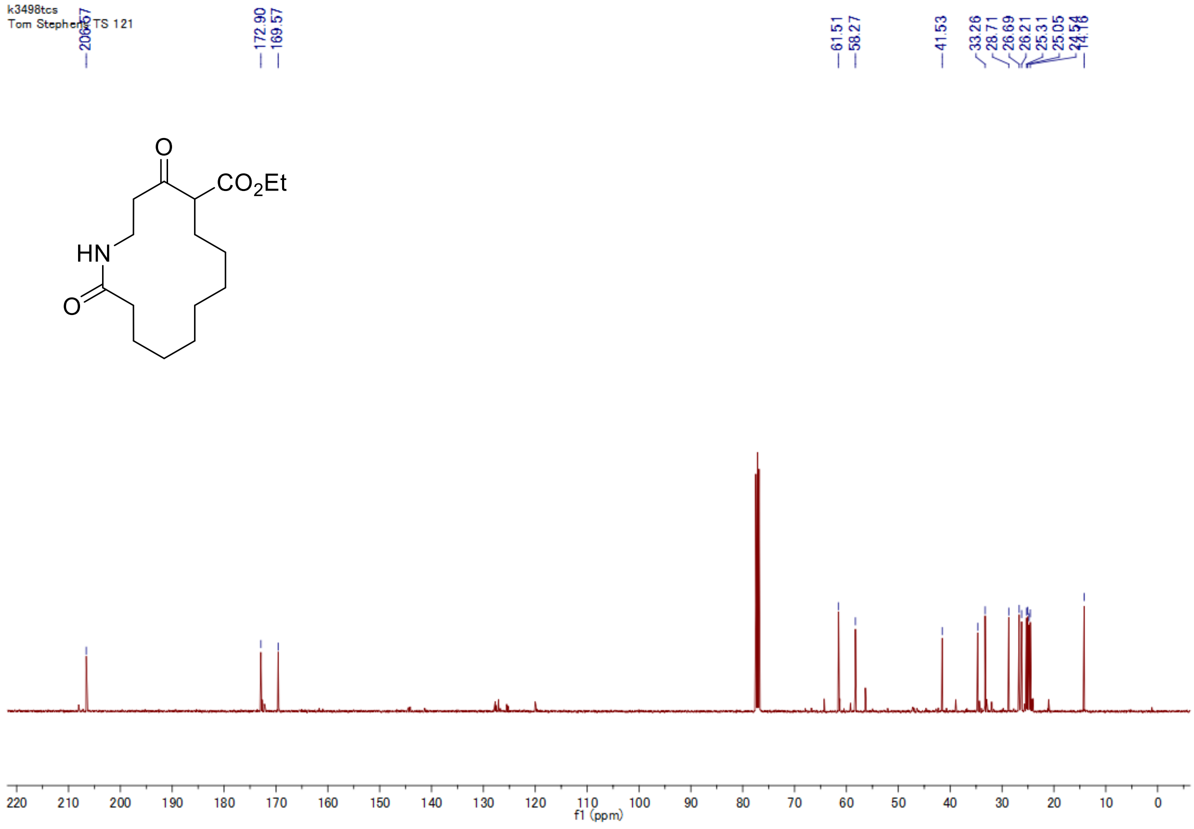
k3498tcs
Tom Stephens TS 121
172.90
169.57
61.51
58.27
41.53
33.26
28.71
26.69
26.21
25.31
25.05
24.54
14.16
O
CO_2Et
HN
O
220 210 200 190 180 170 160 150 140 130 120 110 100 90 80 70 60 50 40 30 20 10 0
f1 (ppm)

Compound 36

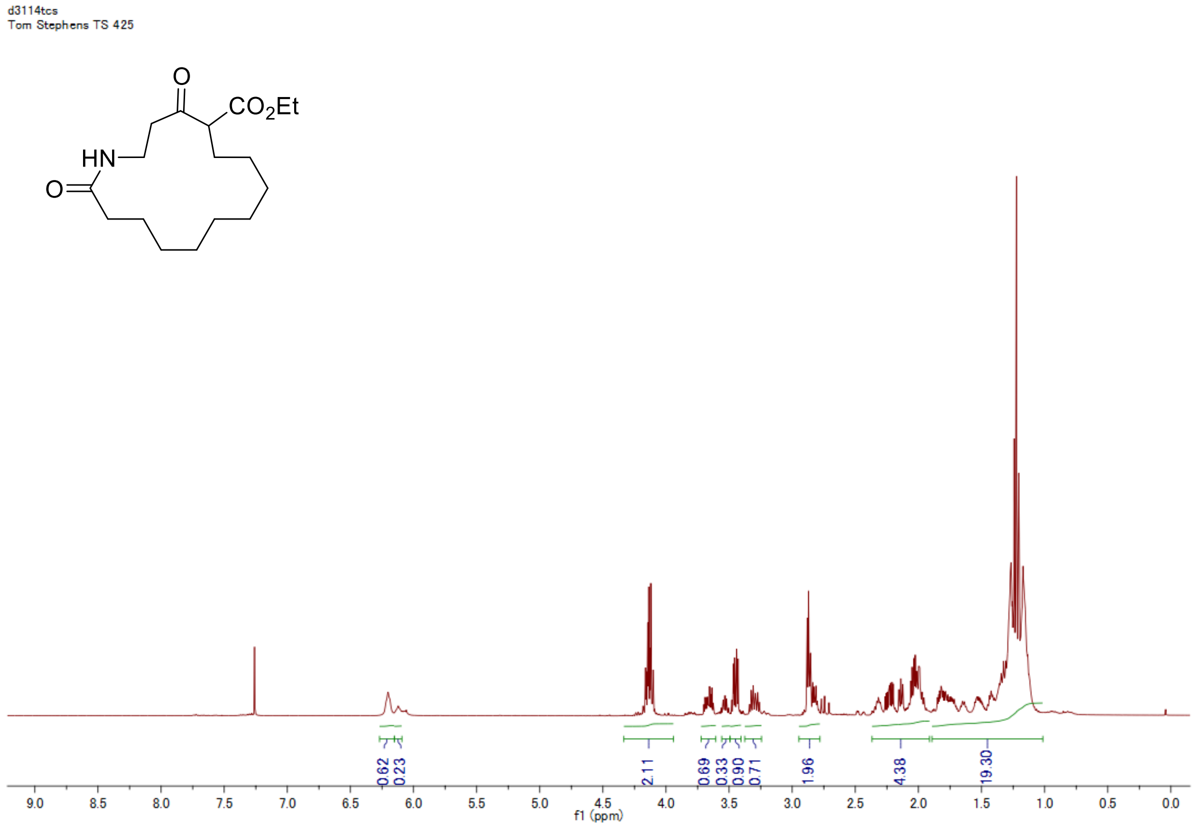
d3114tcs
Tom Stephens TS 425
O
CO2Et
HN
O
0.62
0.23
2.11
0.69
0.33
0.90
0.71
1.96
4.38
19.30
9.0 8.5 8.0 7.5 7.0 6.5 6.0 5.5 5.0 4.5 4.0 3.5 3.0 2.5 2.0 1.5 1.0 0.5 0.0
f1 (ppm)

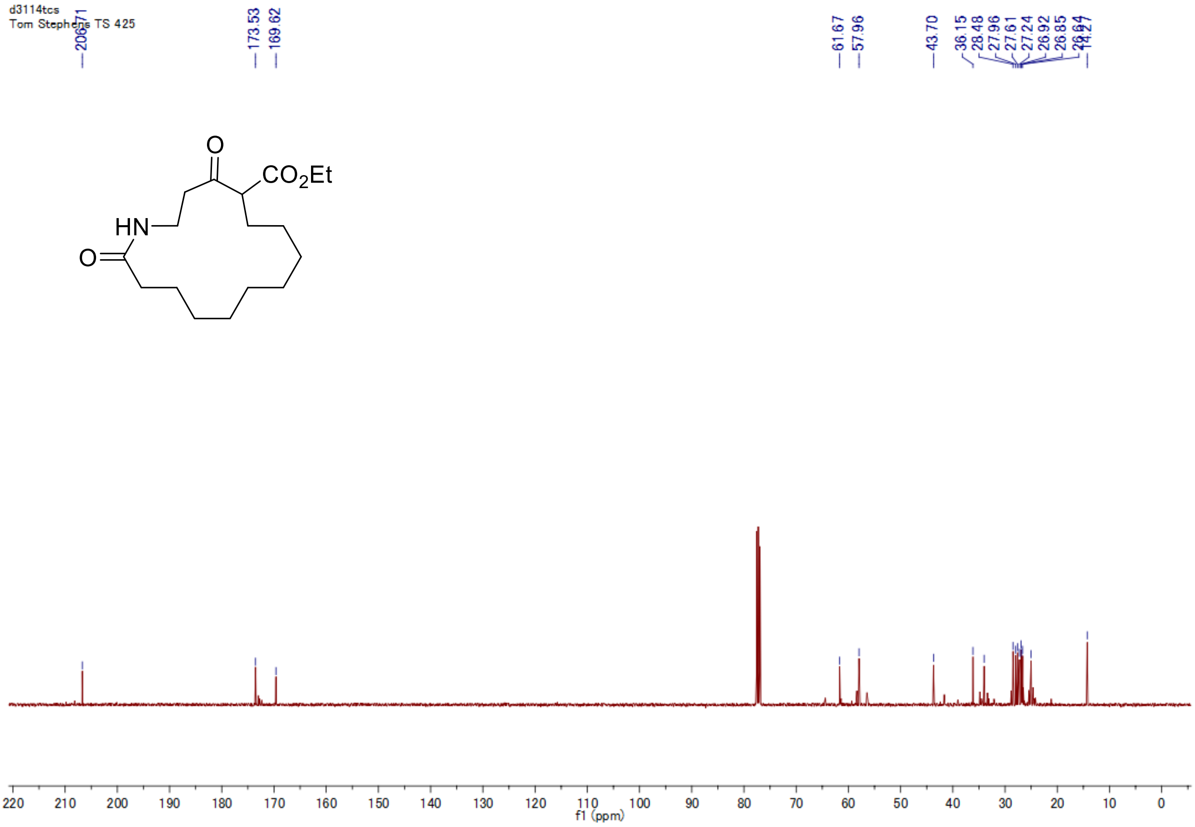
d3114tcs
Tom Stephens TS 425
173.53
169.62
61.67
57.96
43.70
36.15
28.48
27.96
27.61
27.24
26.92
26.85
O
CO2Et
HN
O
220 210 200 190 180 170 160 150 140 130 120 110 100 90 80 70 60 50 40 30 20 10 0
f1 (ppm)

Compound 40RE

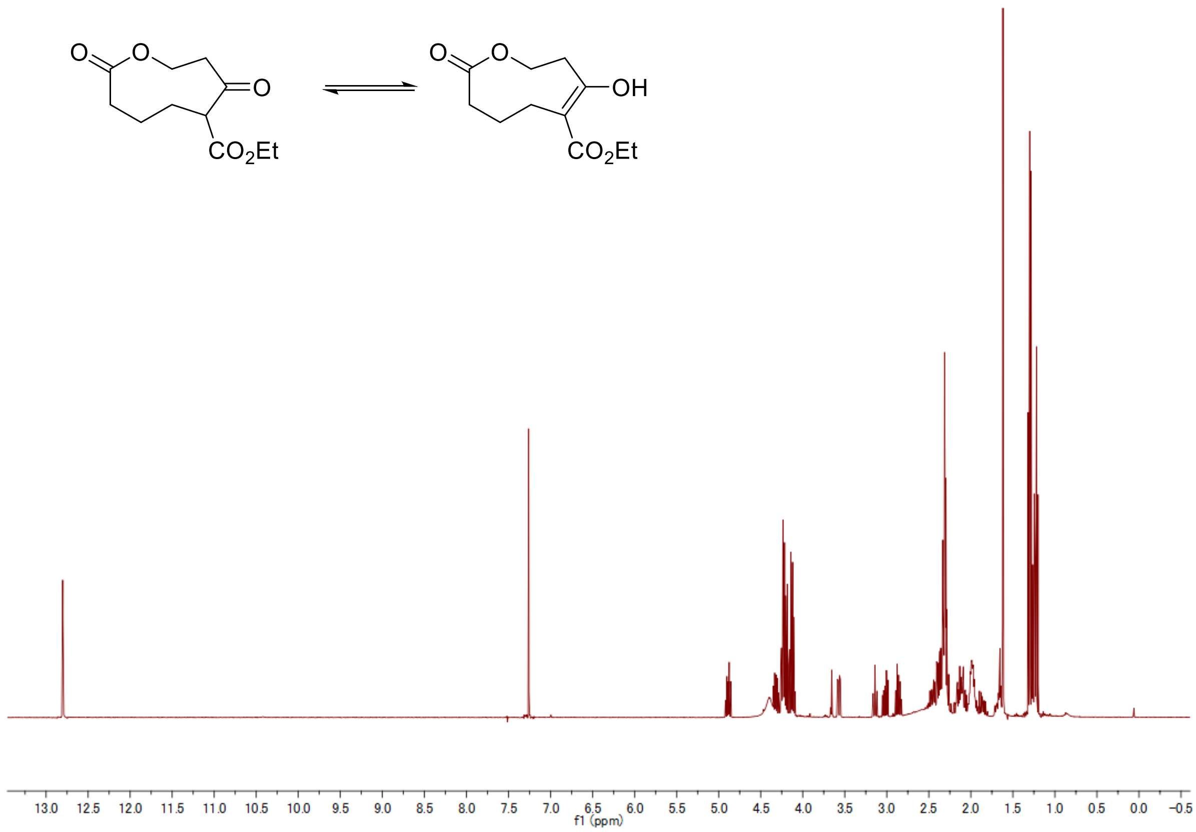

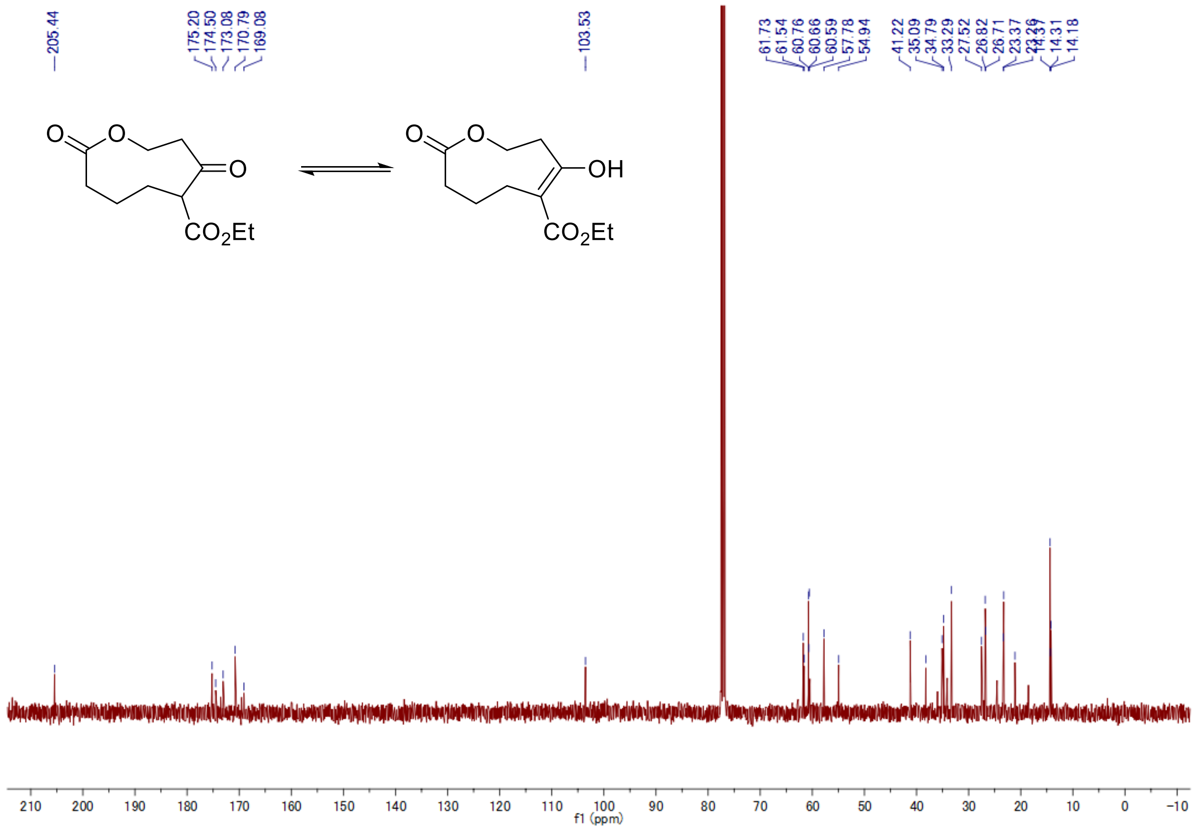

Compound 41RE

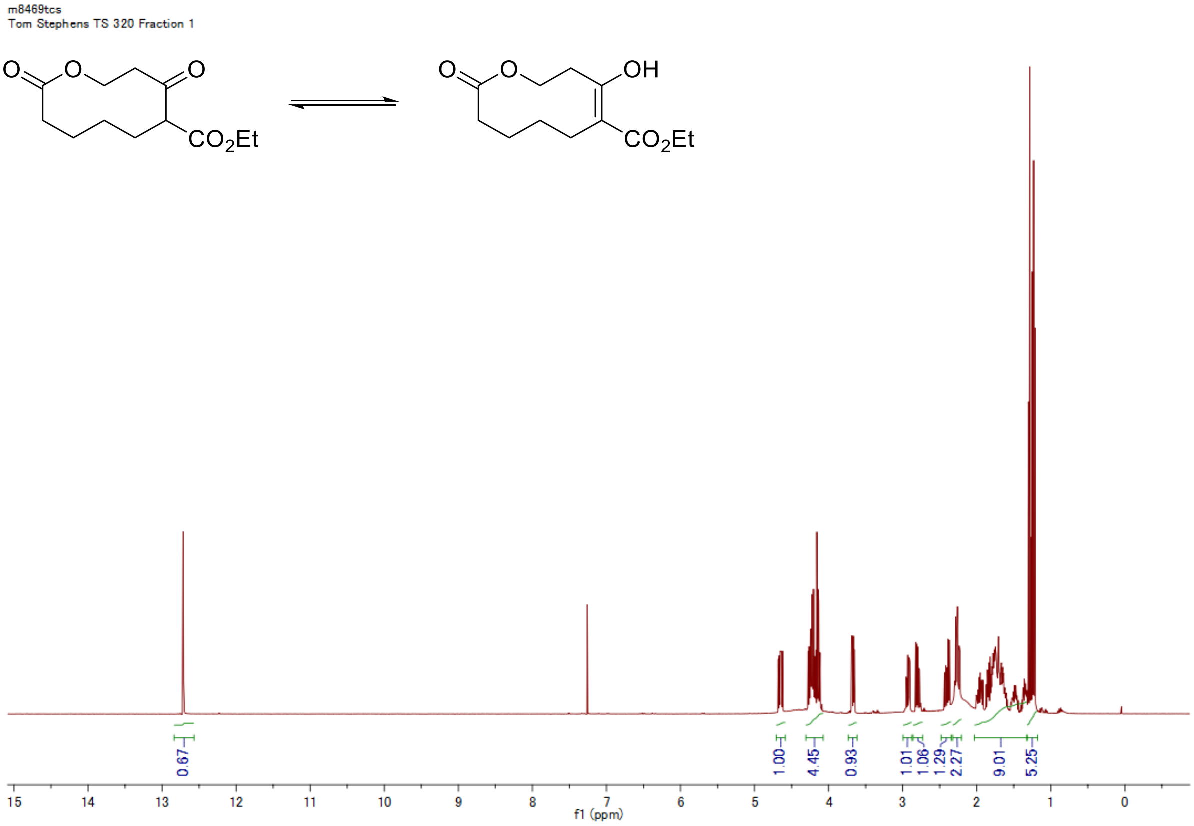

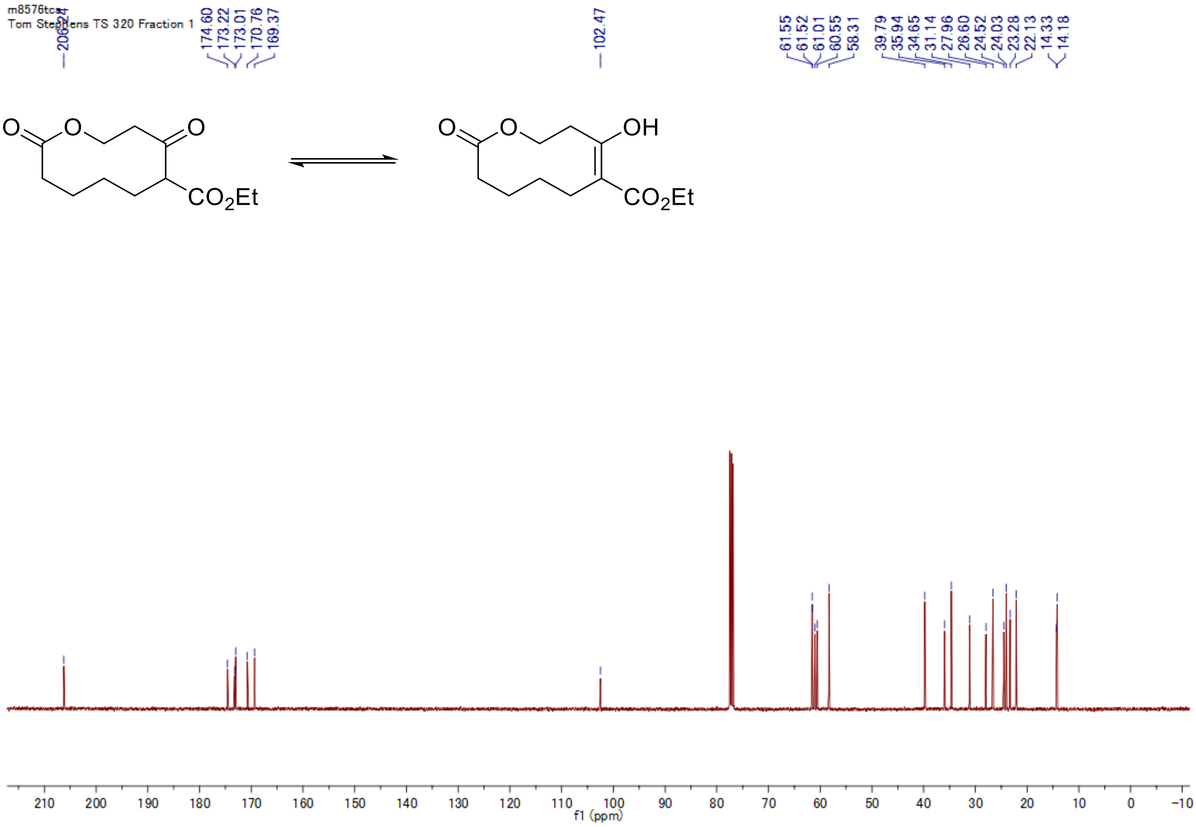

Compound 39

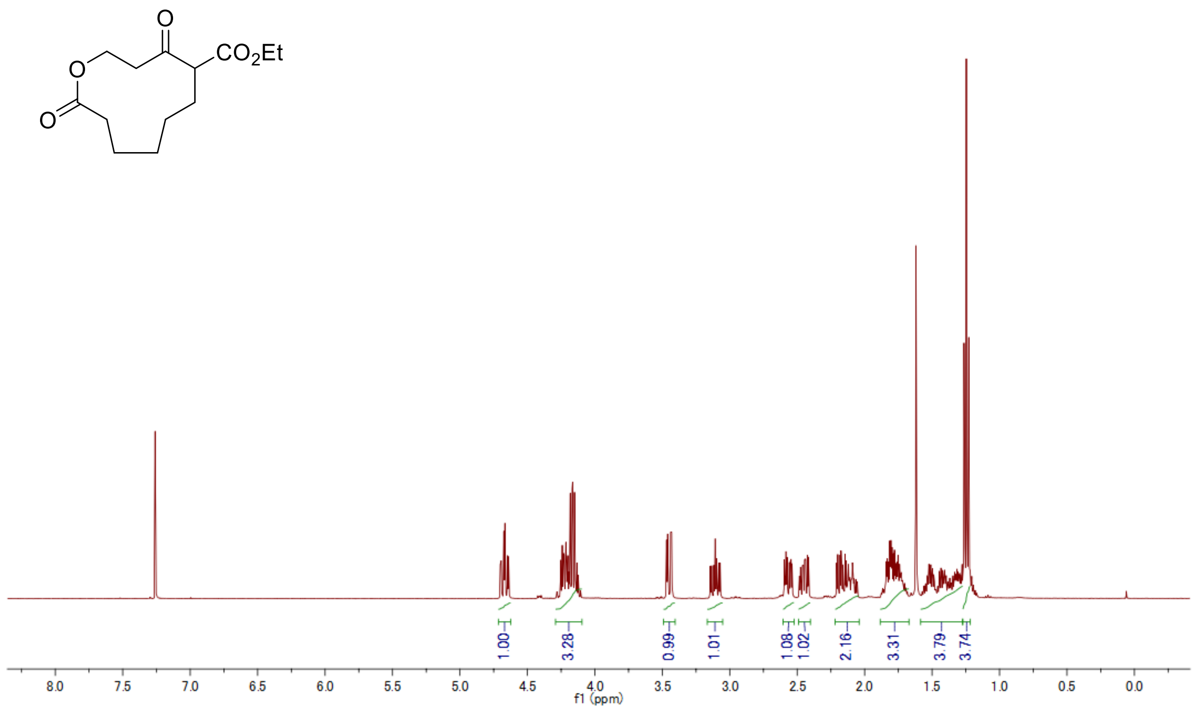

CO2Et
1.00
3.28
0.99
1.01
1.08
1.02
2.16
3.31
3.79
3.74
f1 (ppm)

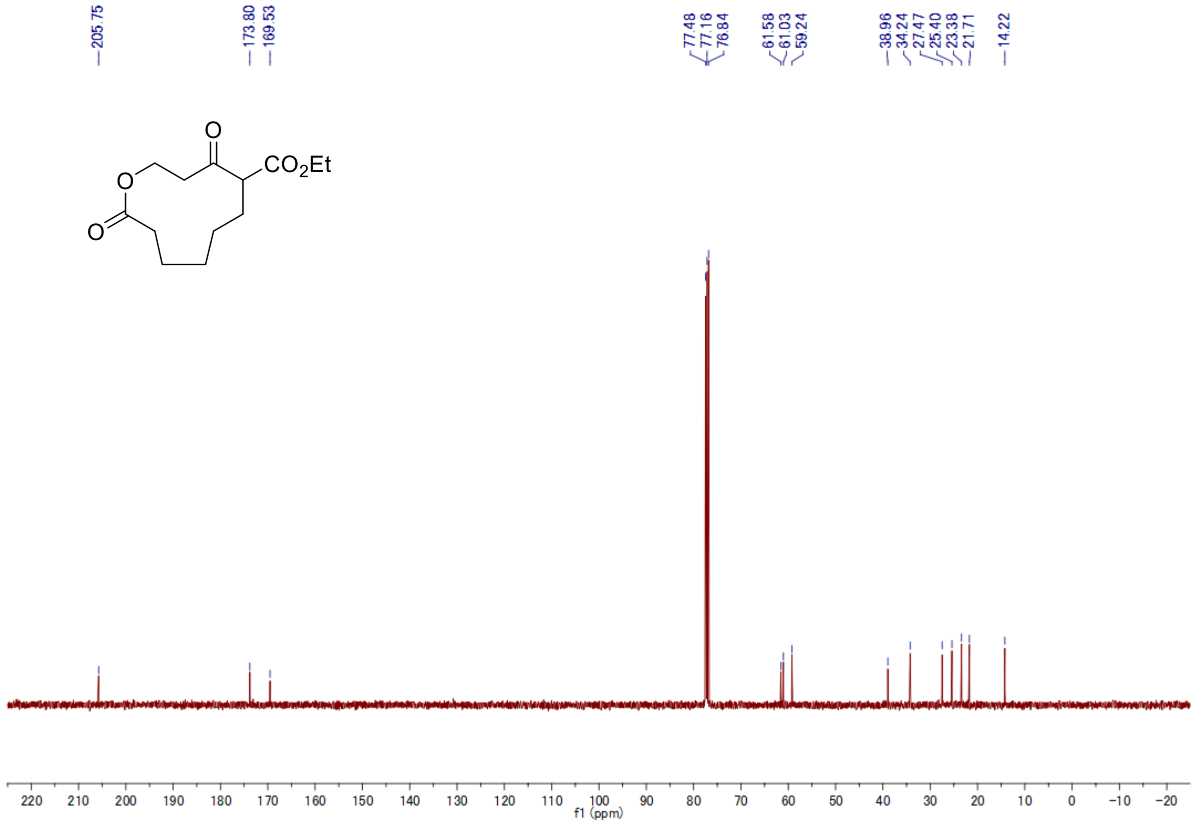

205.75
173.80
169.53
77.48
77.16
76.84
61.58
61.03
59.24
38.96
34.24
27.47
25.40
23.38
21.71
14.22
CO2Et
f1 (ppm)

Compound 43

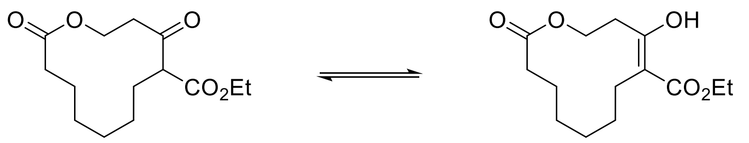

O
O
O
CO2Et
O
O
OH
CO2Et

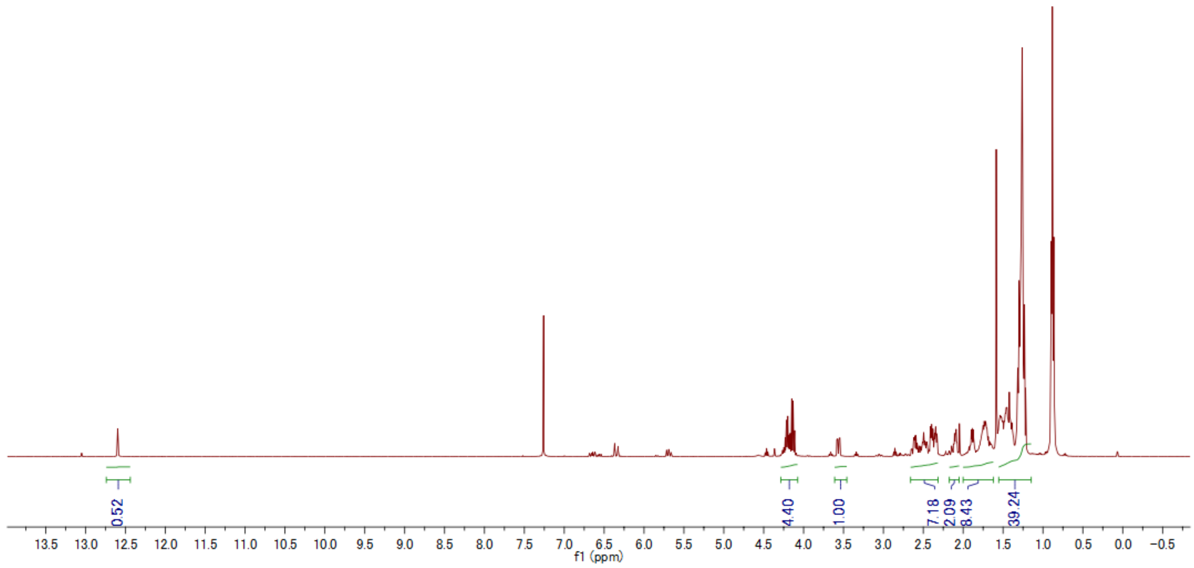

0.52
4.40
1.00
7.18
2.09
8.43
39.24
13.5 13.0 12.5 12.0 11.5 11.0 10.5 10.0 9.5 9.0 8.5 8.0 7.5 7.0 6.5 6.0 5.5 5.0 4.5 4.0 3.5 3.0 2.5 2.0 1.5 1.0 0.5 0.0 −0.5
f1 (ppm)

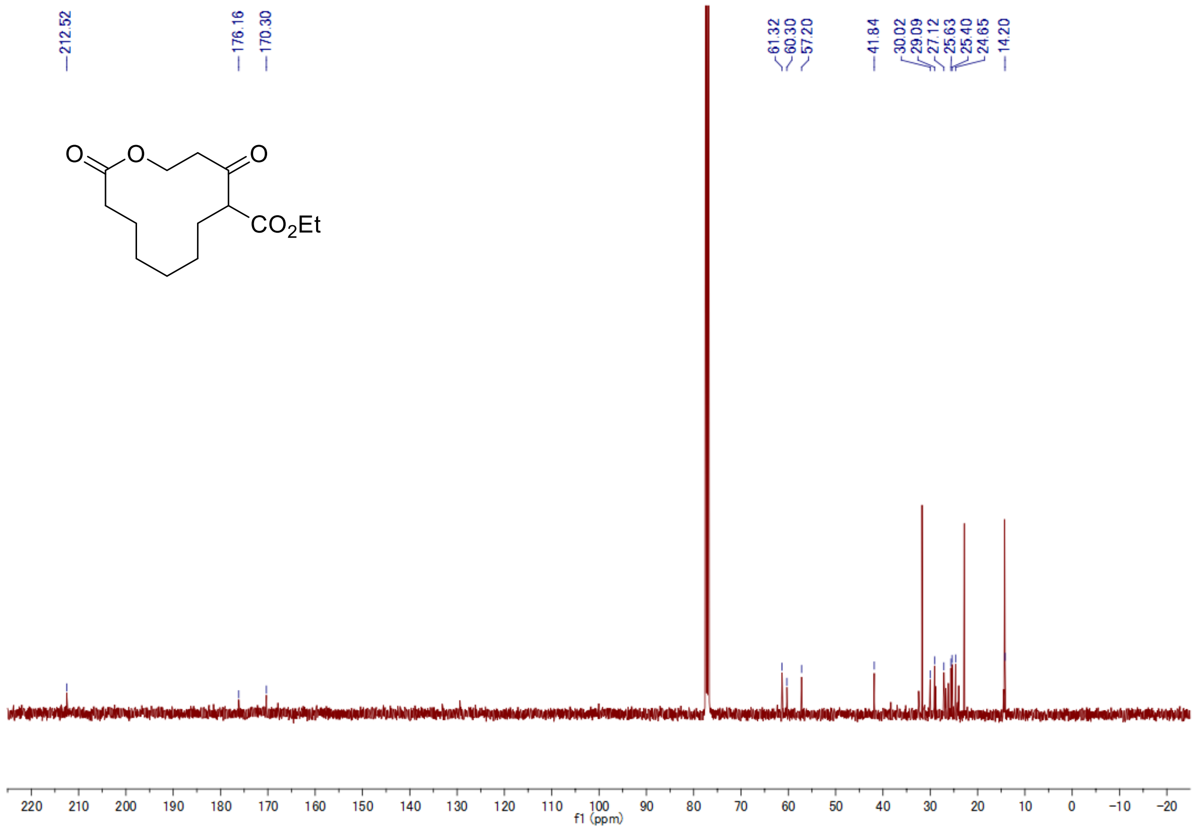

212.52
176.16
170.30
61.32
60.30
57.20
41.84
30.02
29.09
27.12
25.63
25.40
24.65
14.20
O
O
O
CO2Et
220 210 200 190 180 170 160 150 140 130 120 110 100 90 80 70 60 50 40 30 20 10 0 −10 −20
f1 (ppm)

Compound 53RE

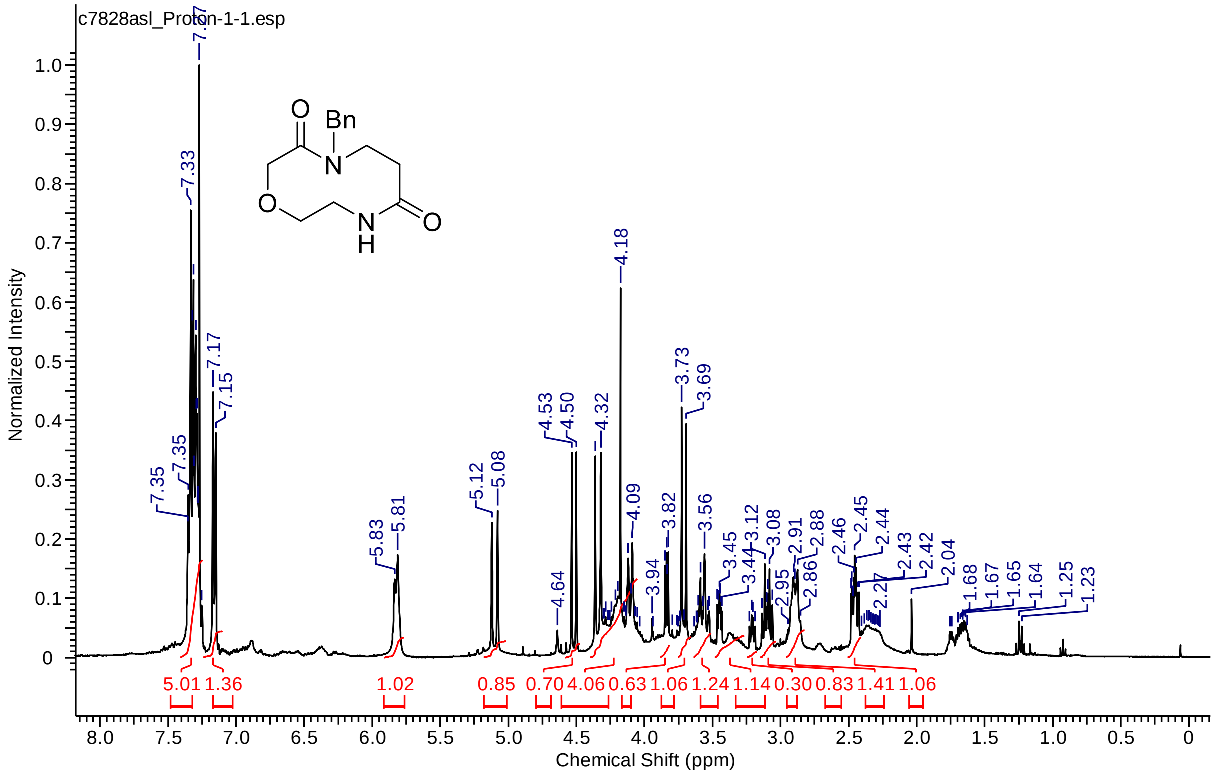

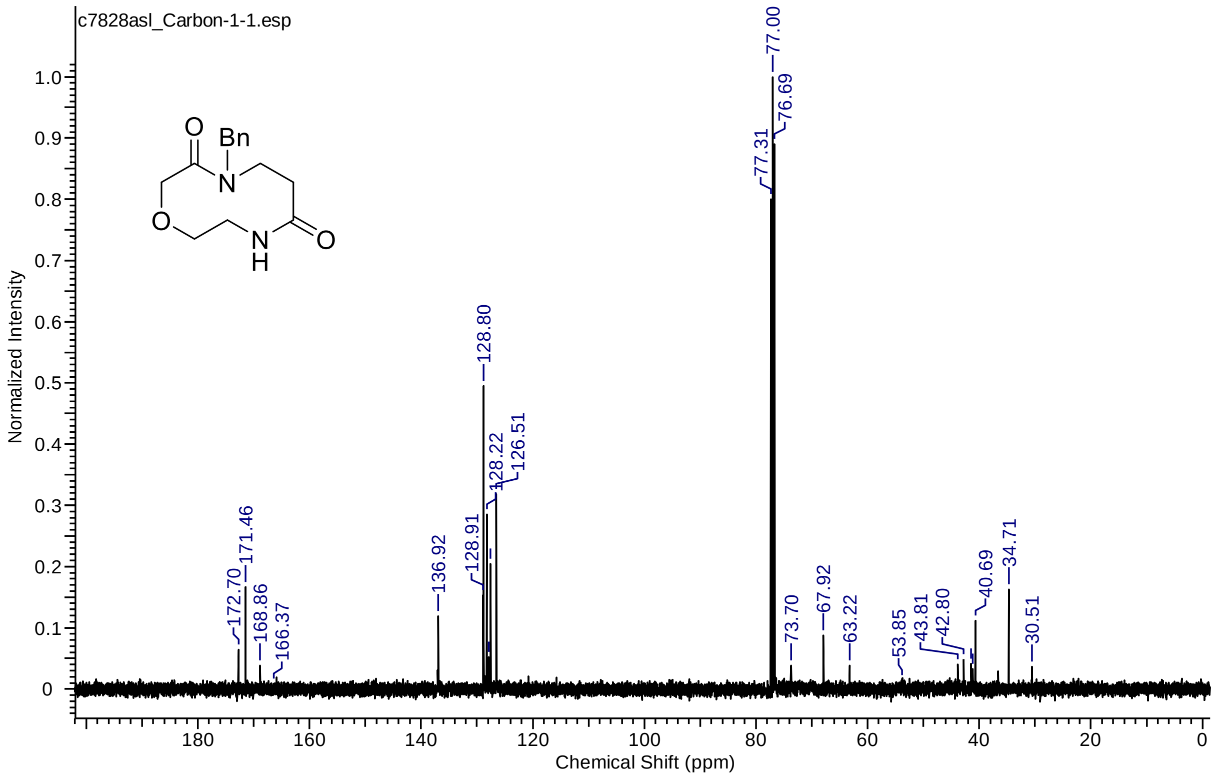

Compound 54RO

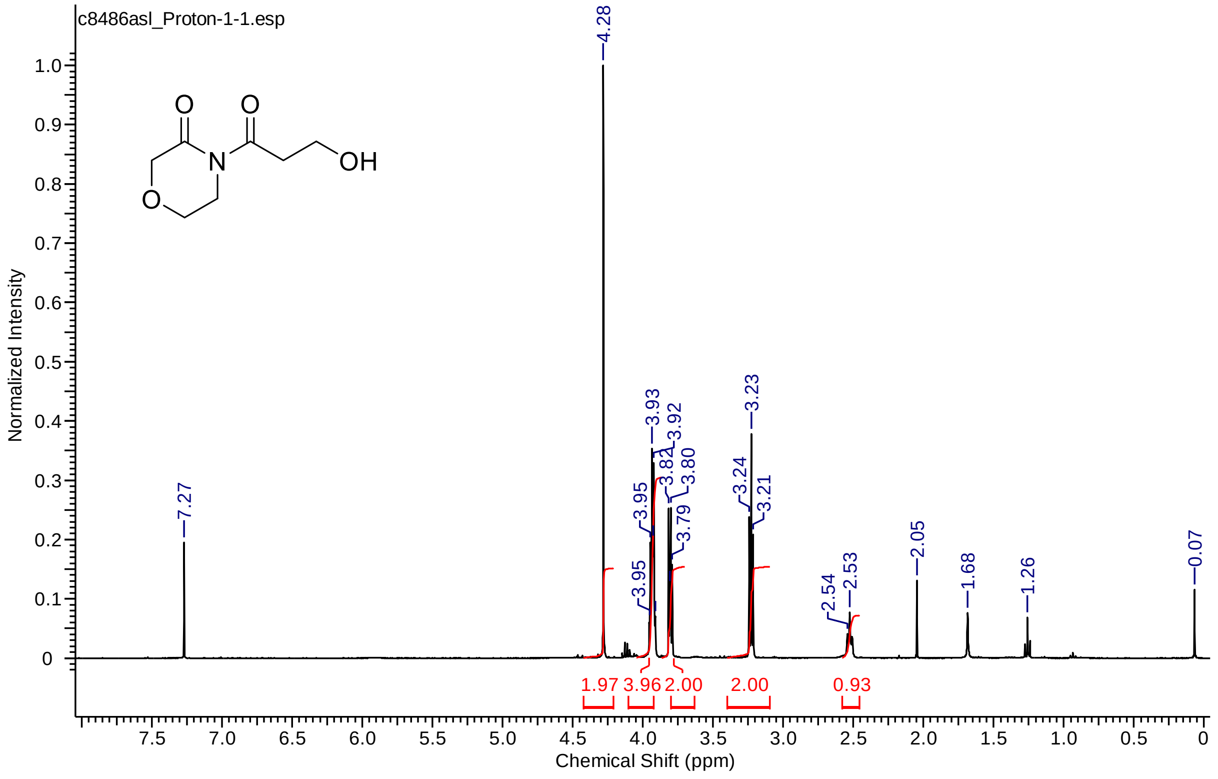

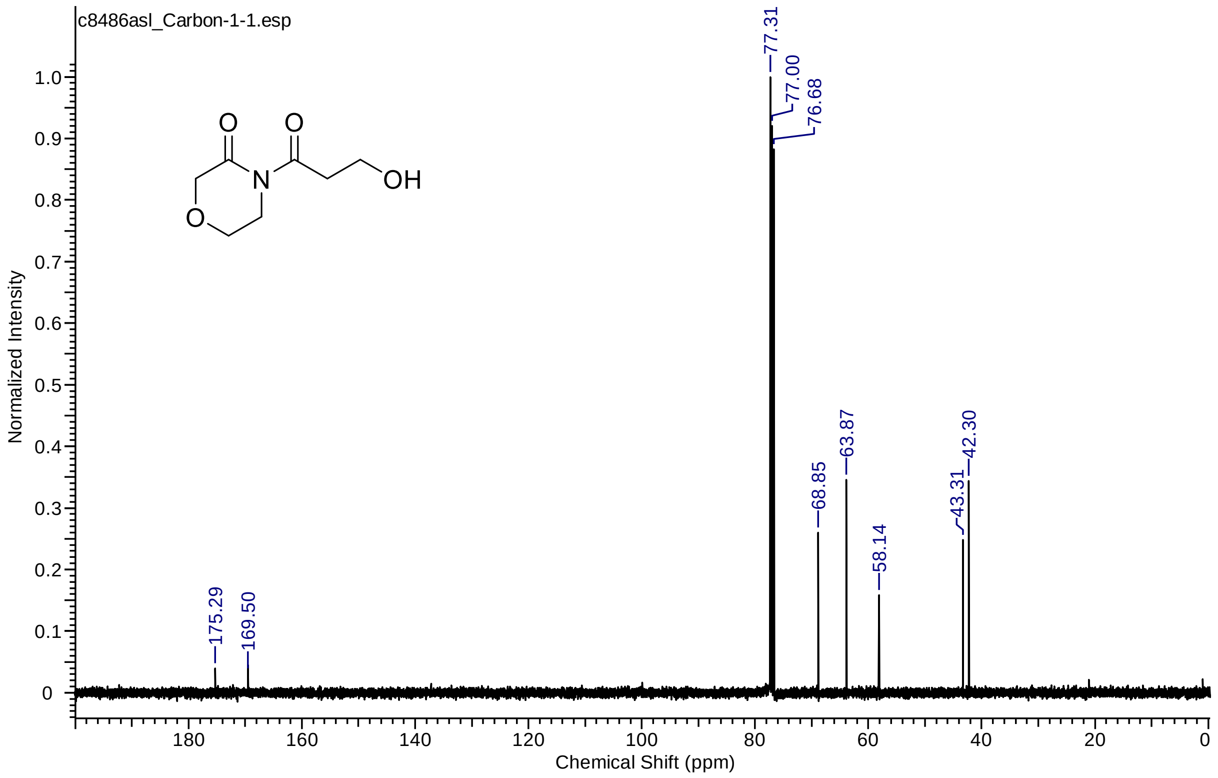

Compound 54RO and 54RE

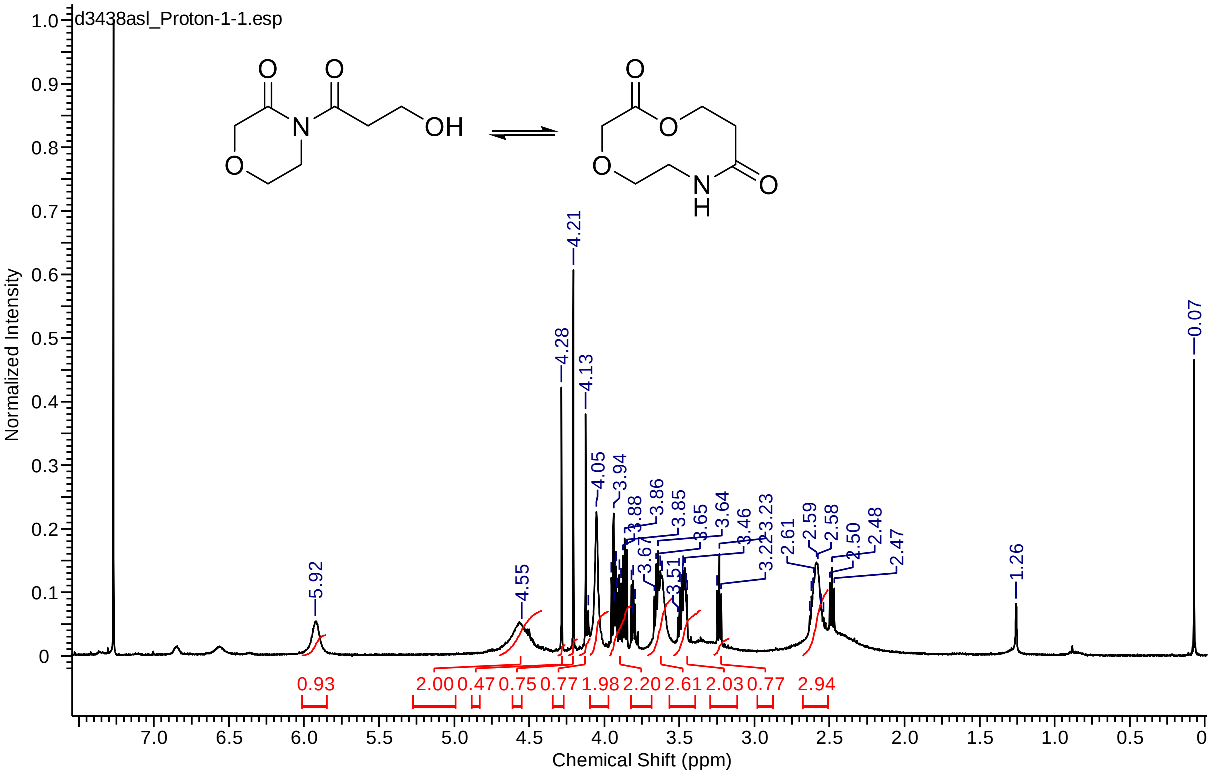

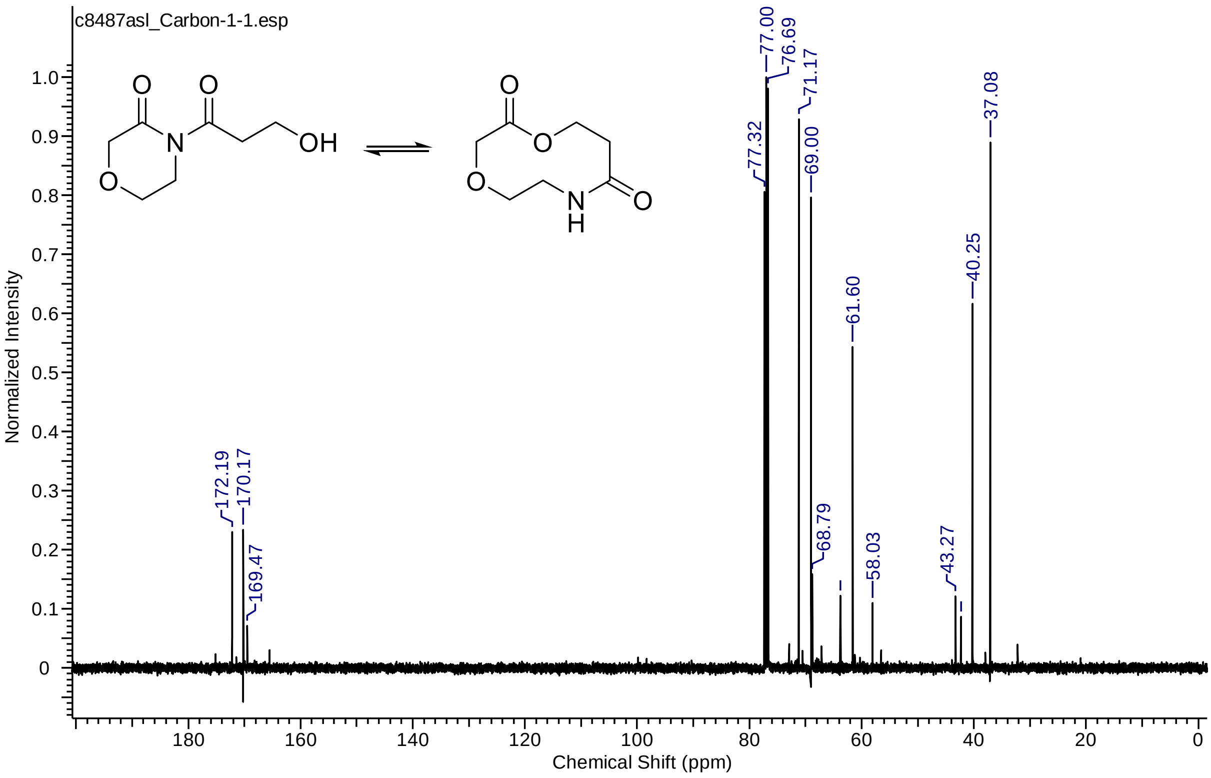

Compound 56$_{RE}$

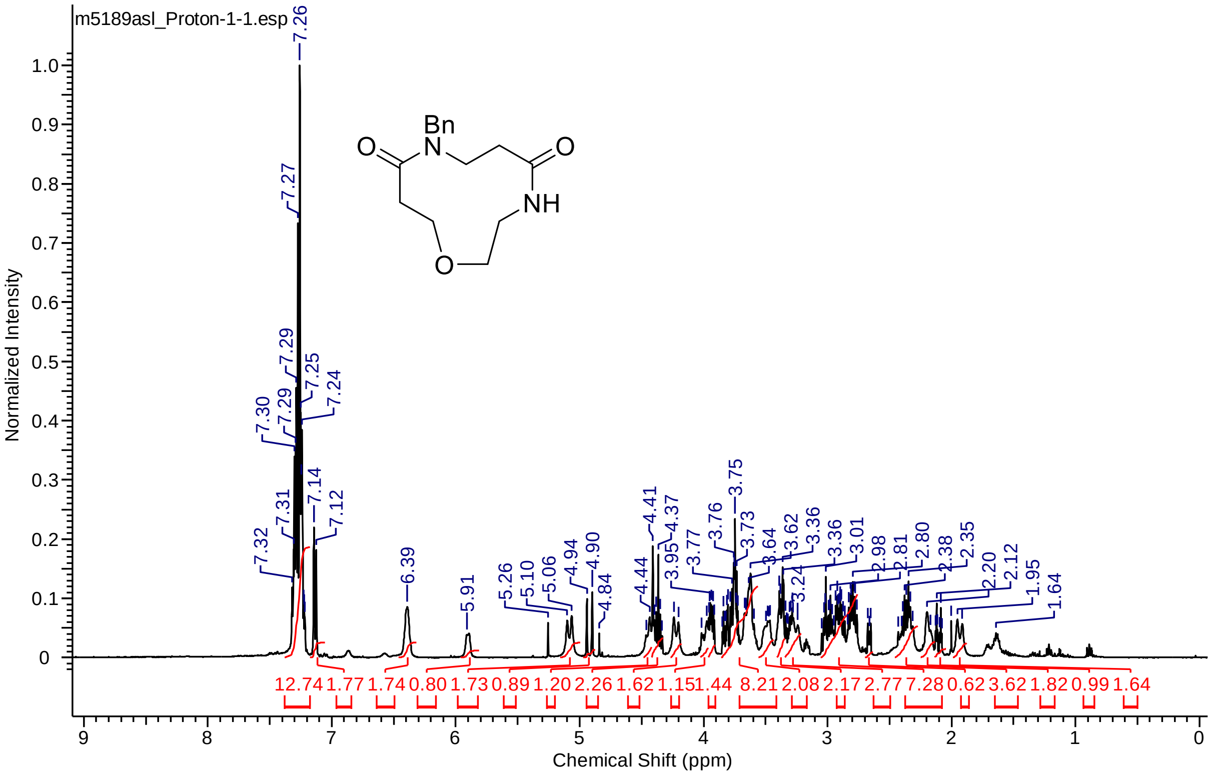

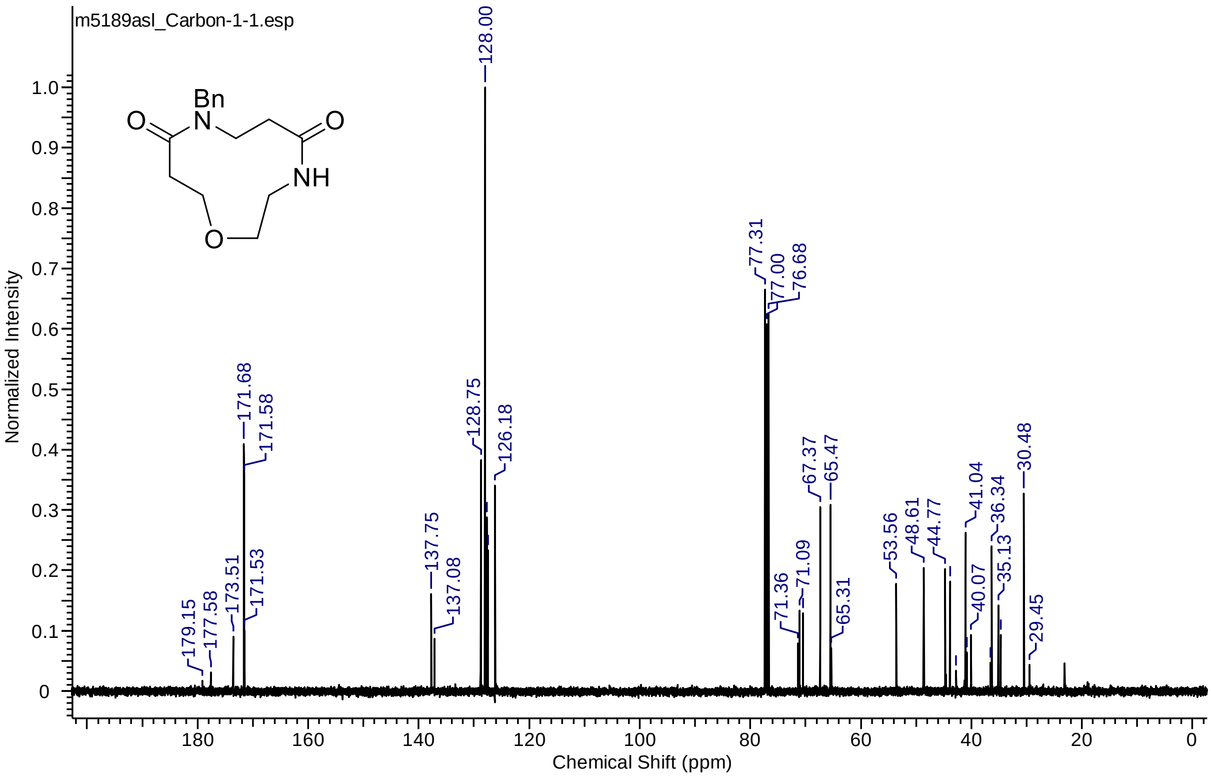

Compound 59RE

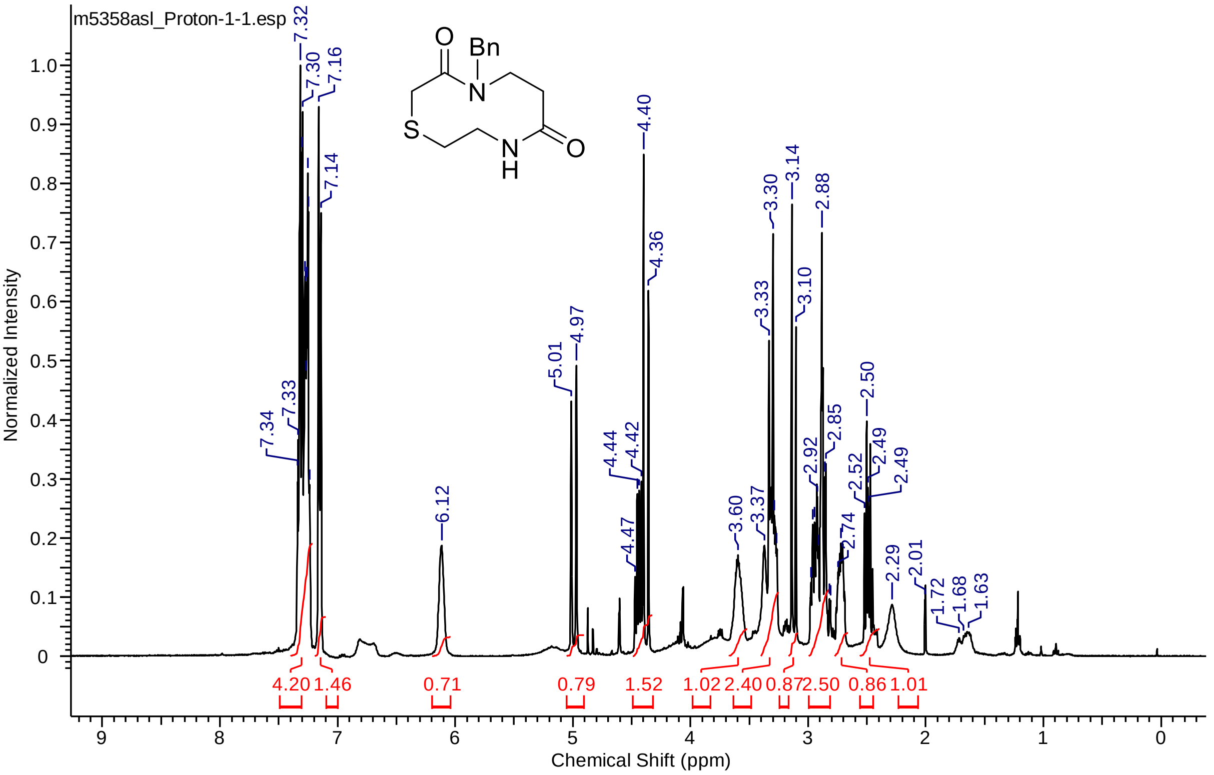

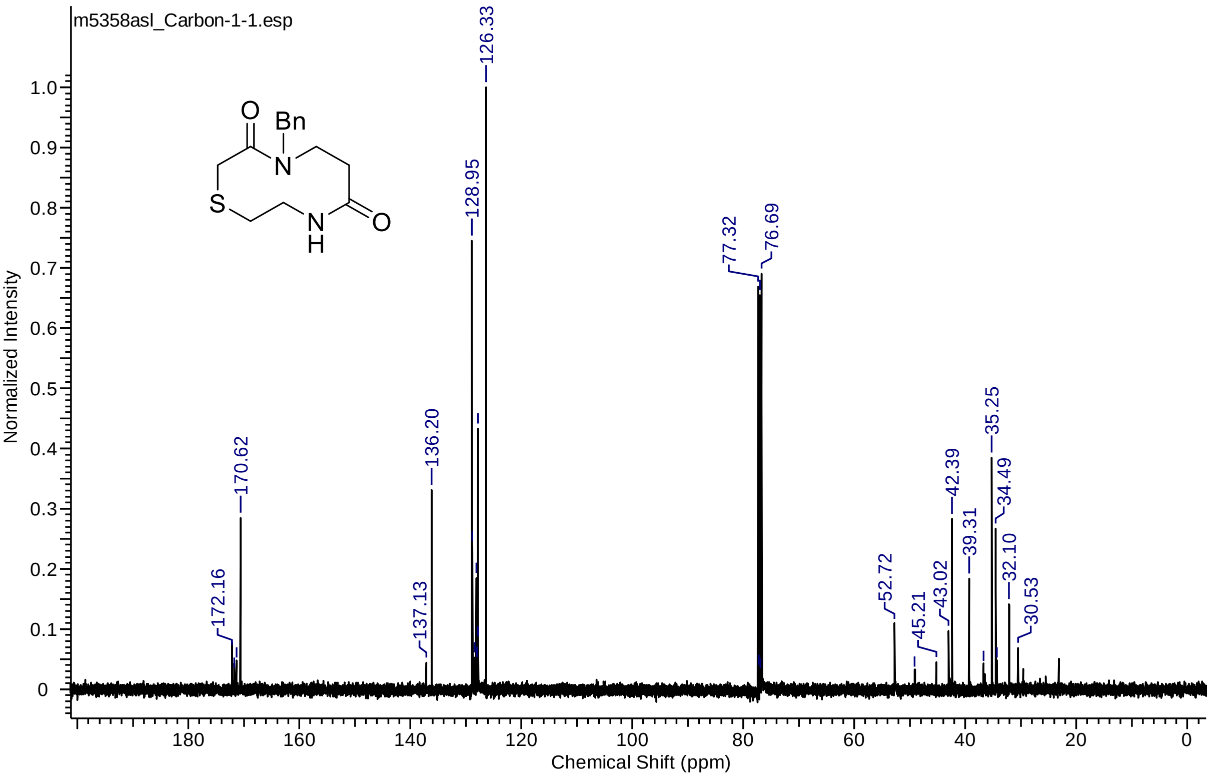

Compound 61RE

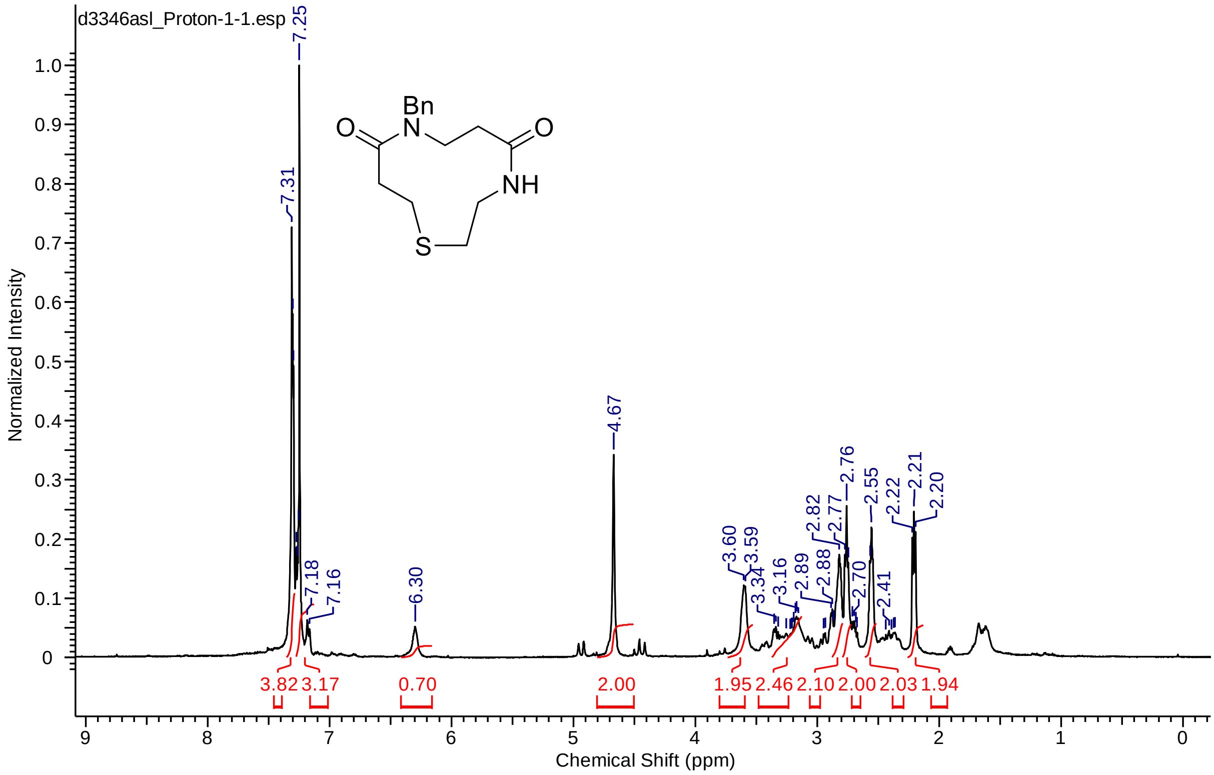

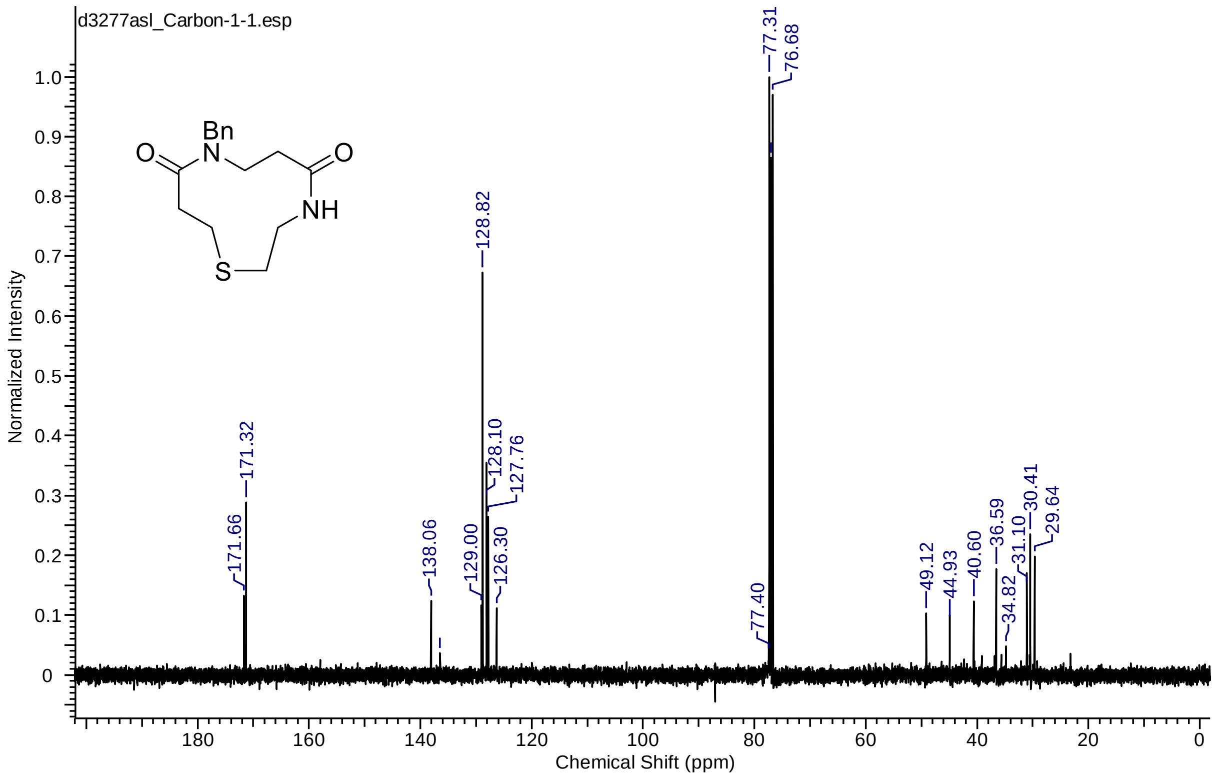

Compound 63RE

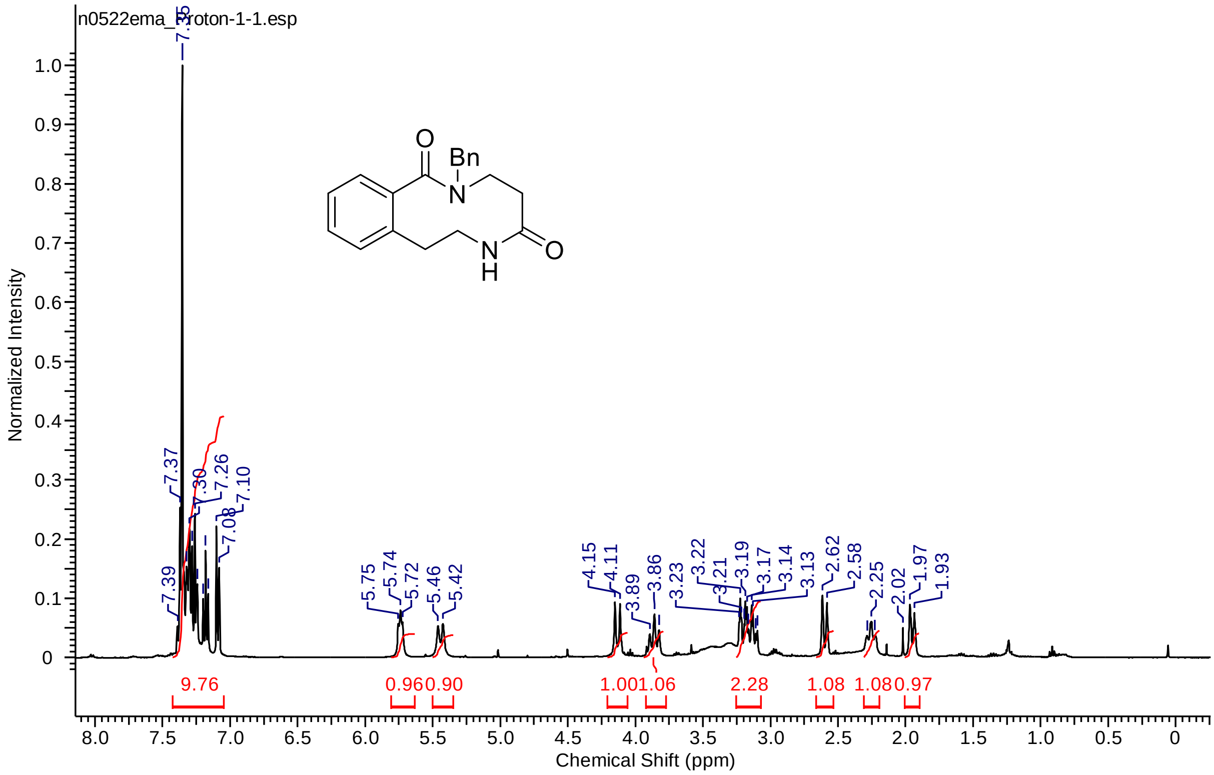

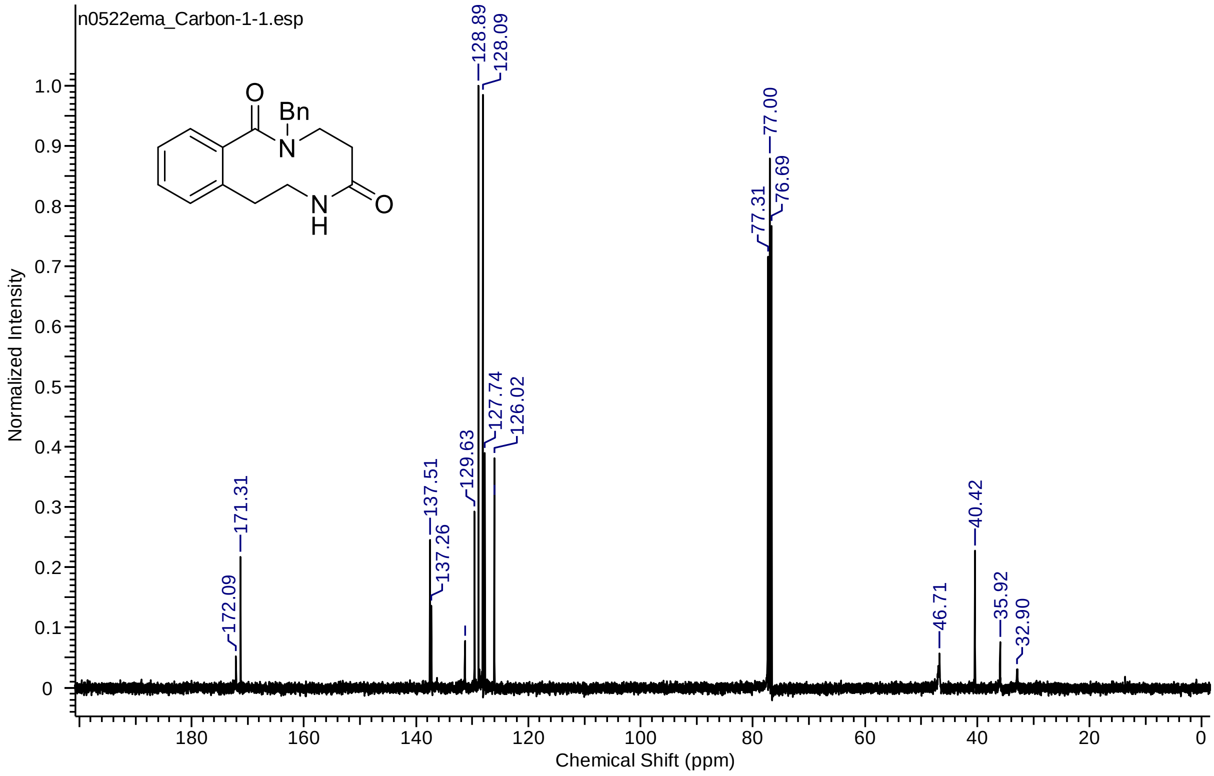

Compound 64RO

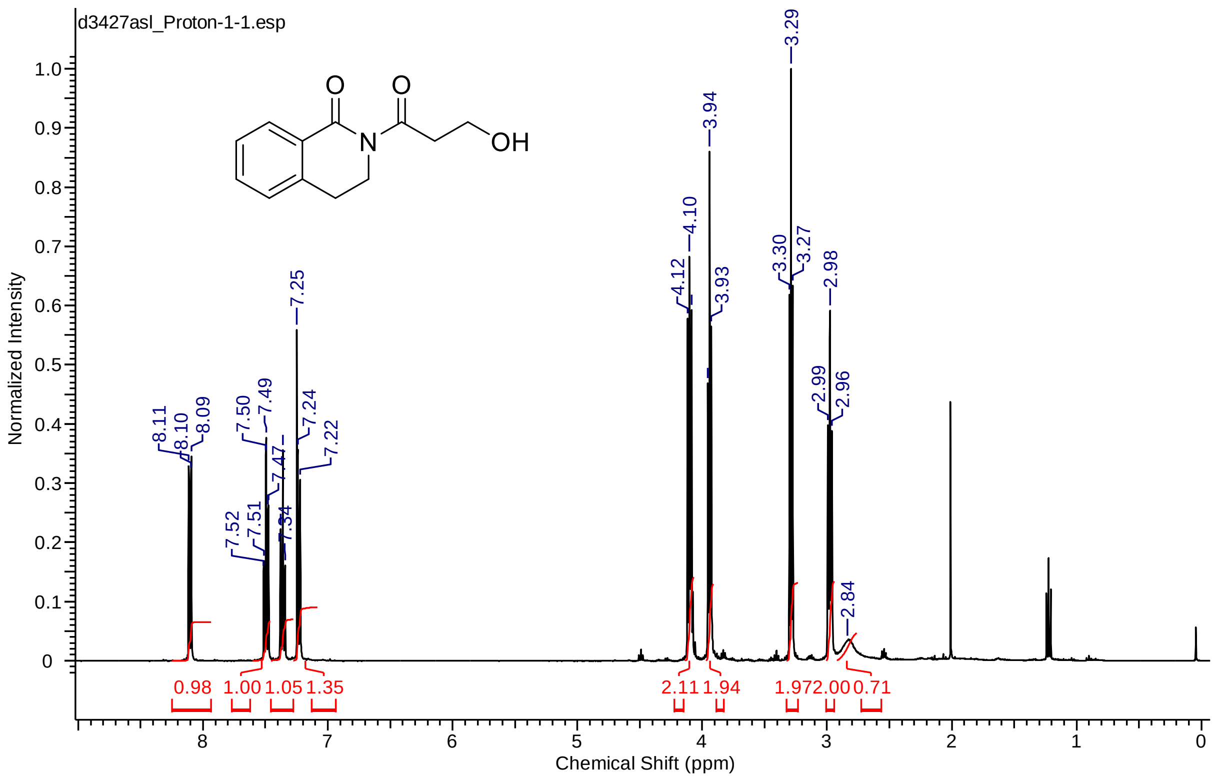
d3427asl_Proton-1-1.esp
Normalized Intensity
Chemical Shift (ppm)
OH
N
O
O
8.11
8.10
8.09
7.52
7.51
7.50
7.49
7.47
7.34
7.25
7.24
7.22
4.12
4.10
3.94
3.93
3.30
3.29
3.27
2.99
2.98
2.96
2.84
0.98
1.00
1.05
1.35
2.11
1.94
1.97
2.00
0.71

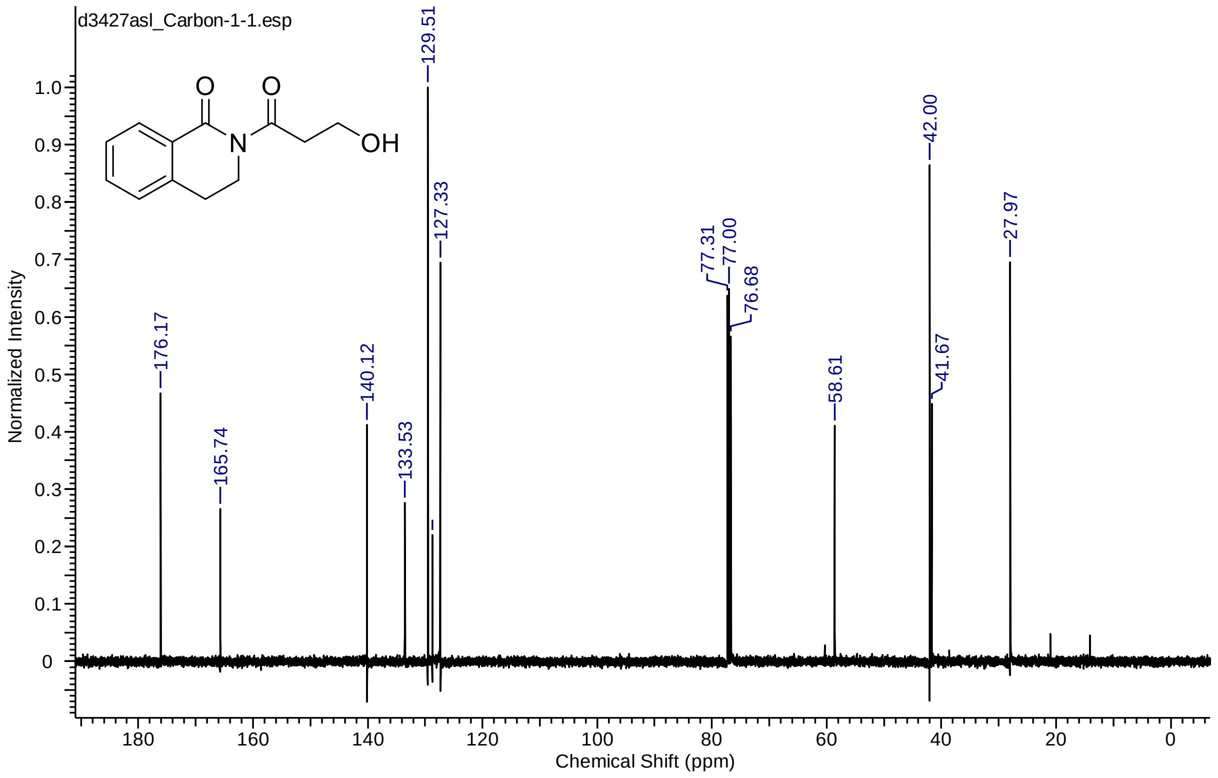
d3427asl_Carbon-1-1.esp
Normalized Intensity
Chemical Shift (ppm)
OH
N
O
O
176.17
165.74
140.12
133.53
129.51
127.33
77.31
77.00
76.68
58.61
42.00
41.67
27.97

Compound 66$_{RE}$

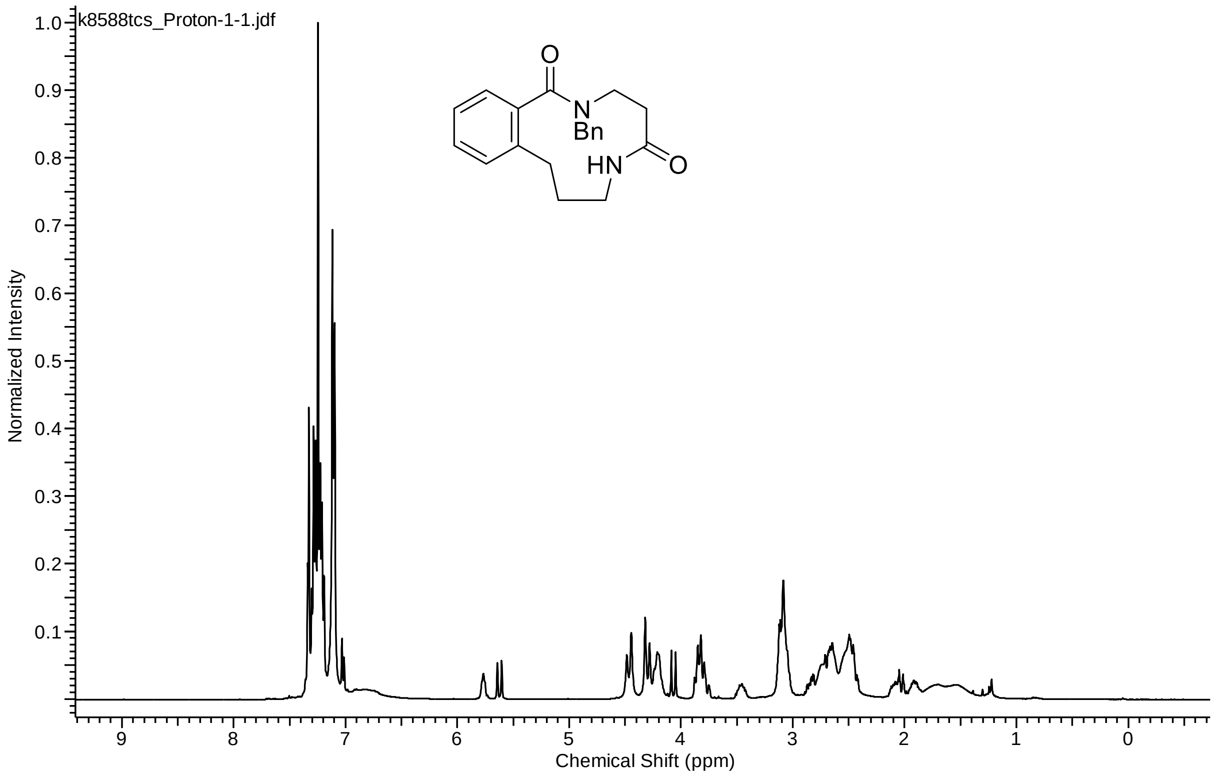

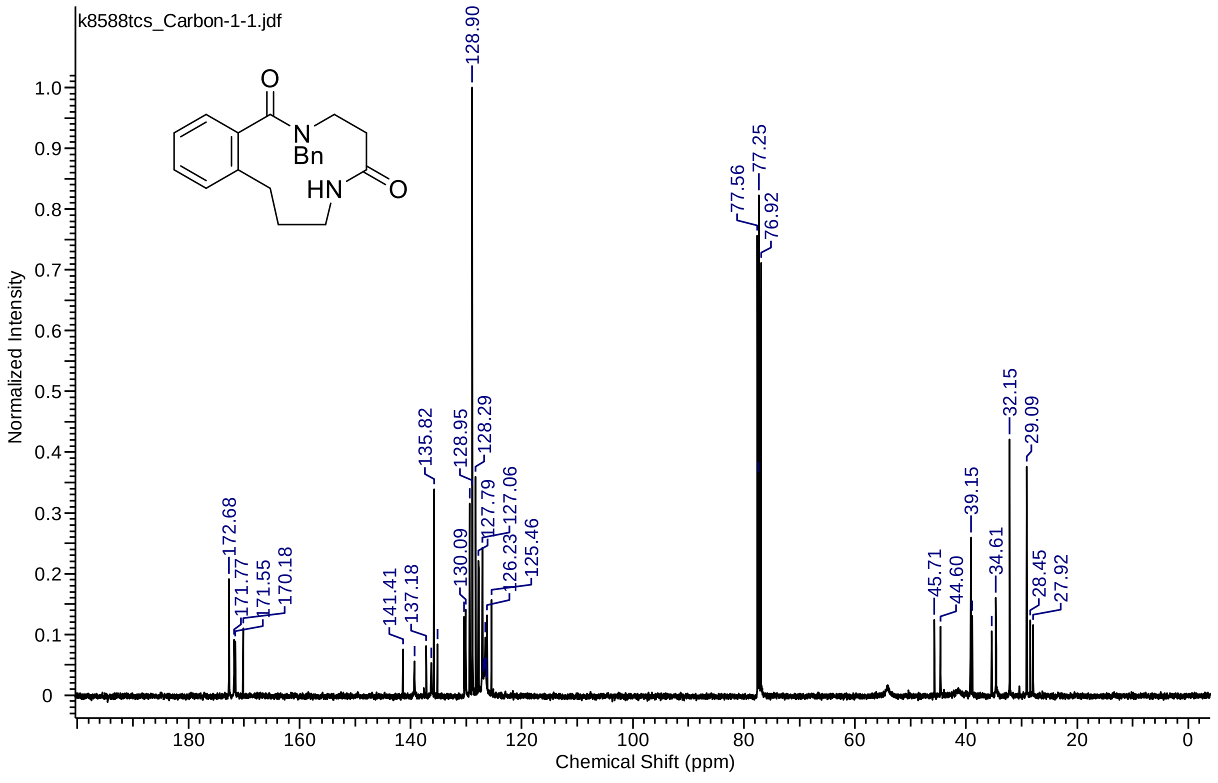

Compound 69$_{RE}$

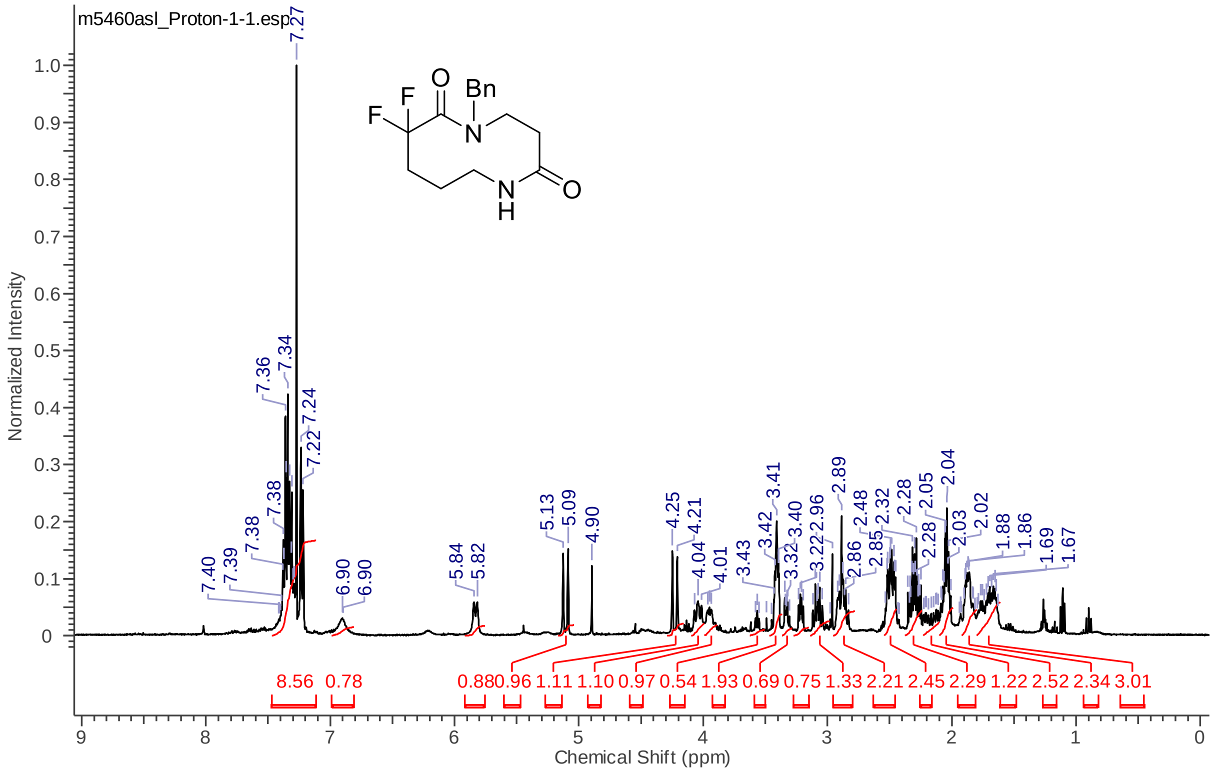
m5460asl_Proton-1-1.esp
Normalized Intensity
Chemical Shift (ppm)
O
F
F
Bn
N
N
H
O
8.56
0.78

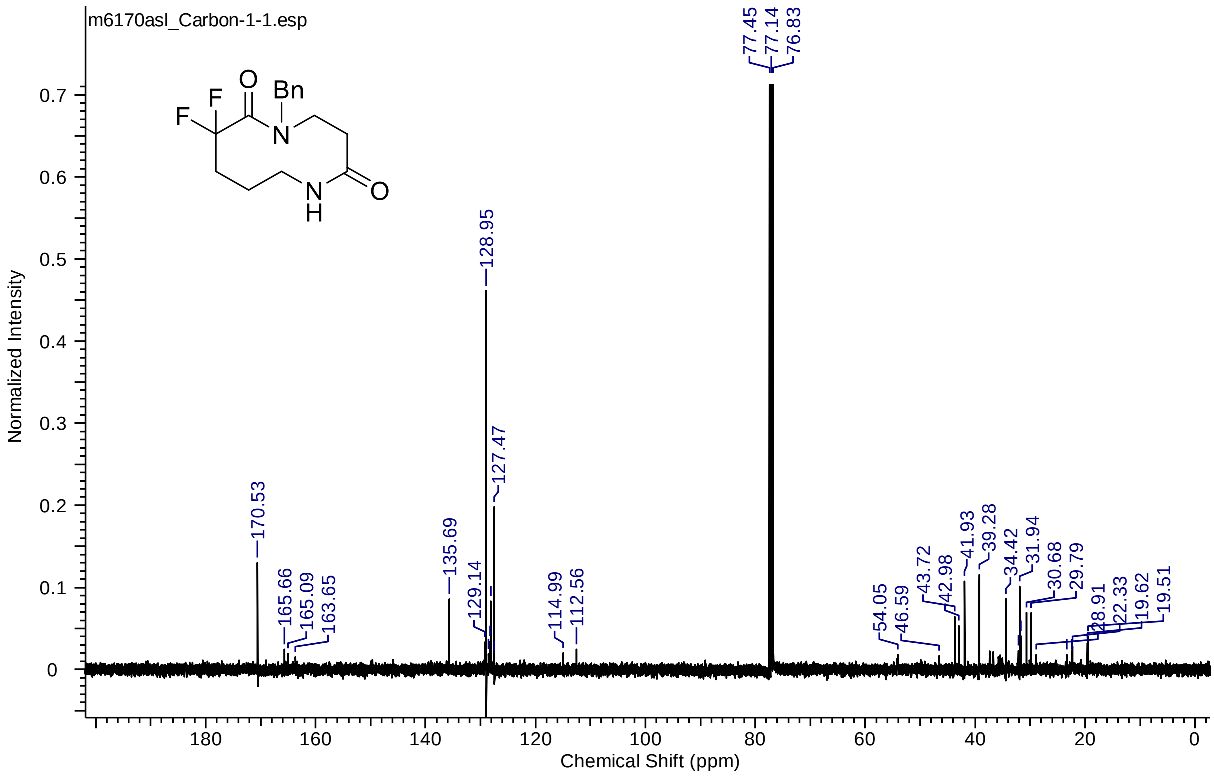
m6170asl_Carbon-1-1.esp
Normalized Intensity
Chemical Shift (ppm)
170.53
135.69
128.95
127.47
77.45
77.14
76.83

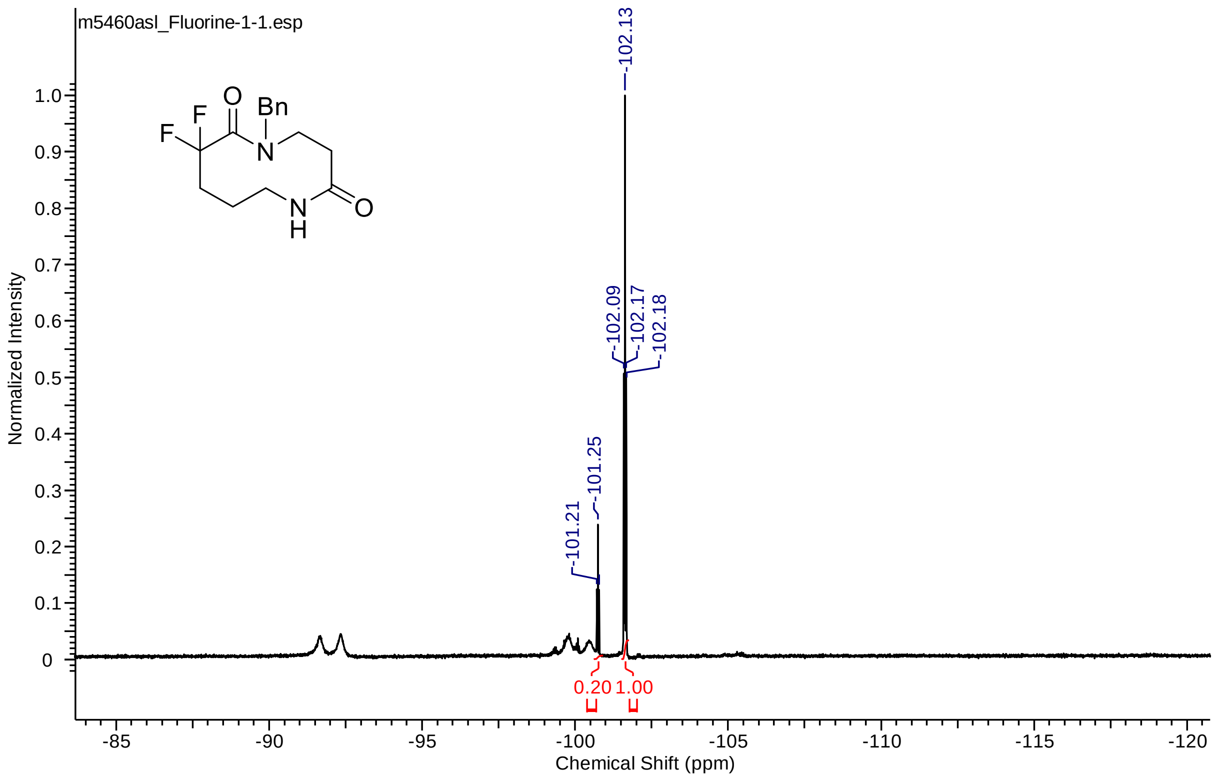
m5460asl_Fluorine-1-1.esp
F
F
O
Bn
N
N
H
O
-102.13
-102.09
-102.17
-102.18
-101.21
-101.25
0.20
1.00
Normalized Intensity
Chemical Shift (ppm)
1.0
0.9
0.8
0.7
0.6
0.5
0.4
0.3
0.2
0.1
0
-85
-90
-95
-100
-105
-110
-115
-120

Compound 71RE

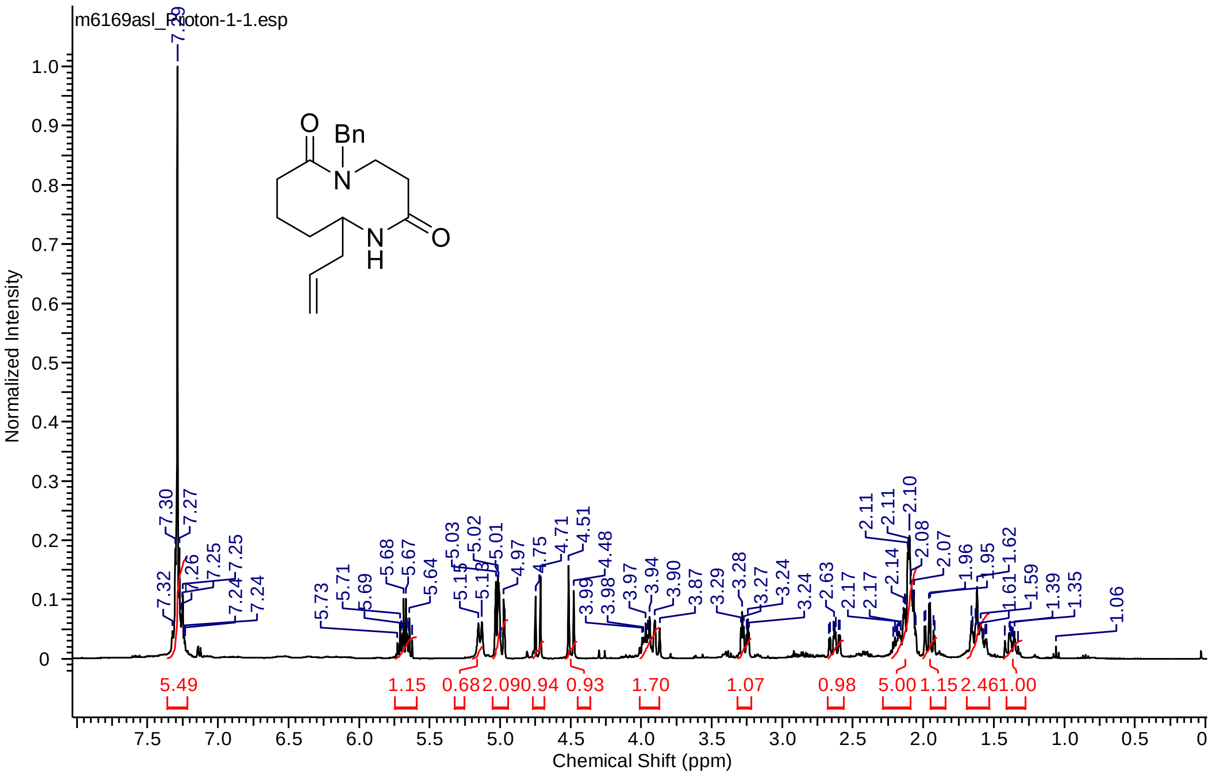

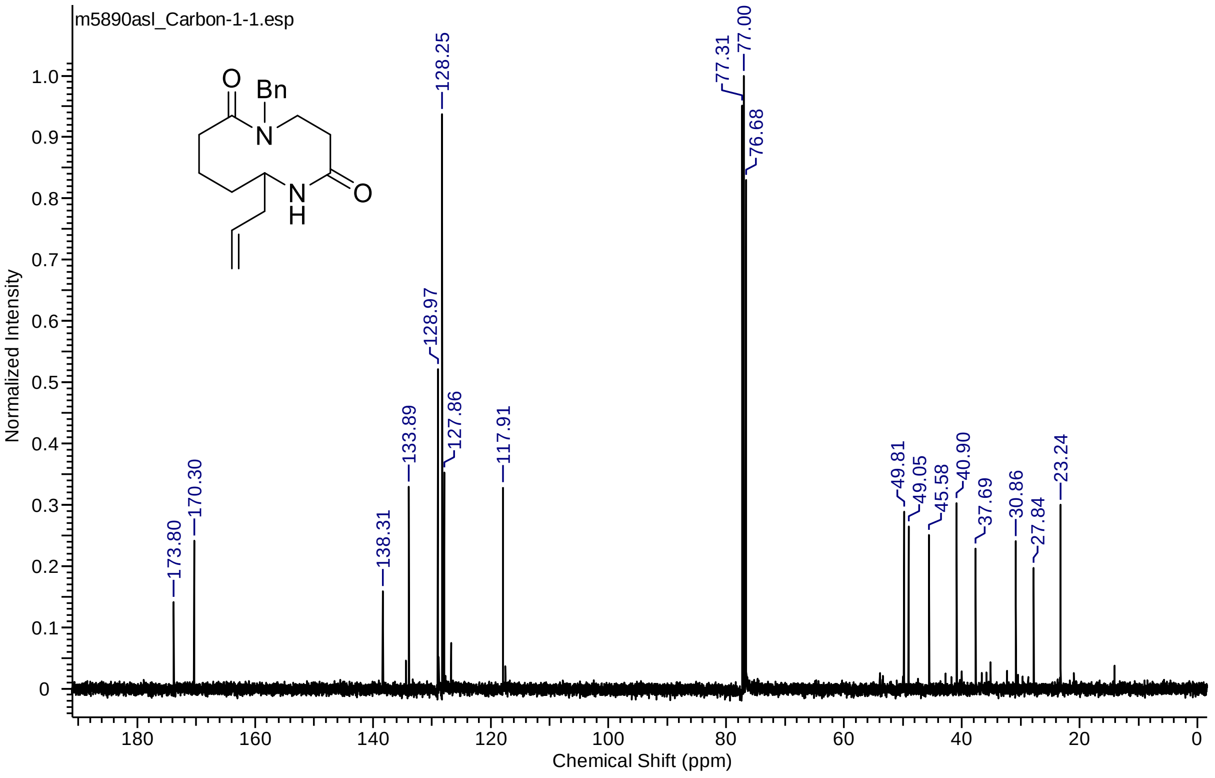

Compound 72RE

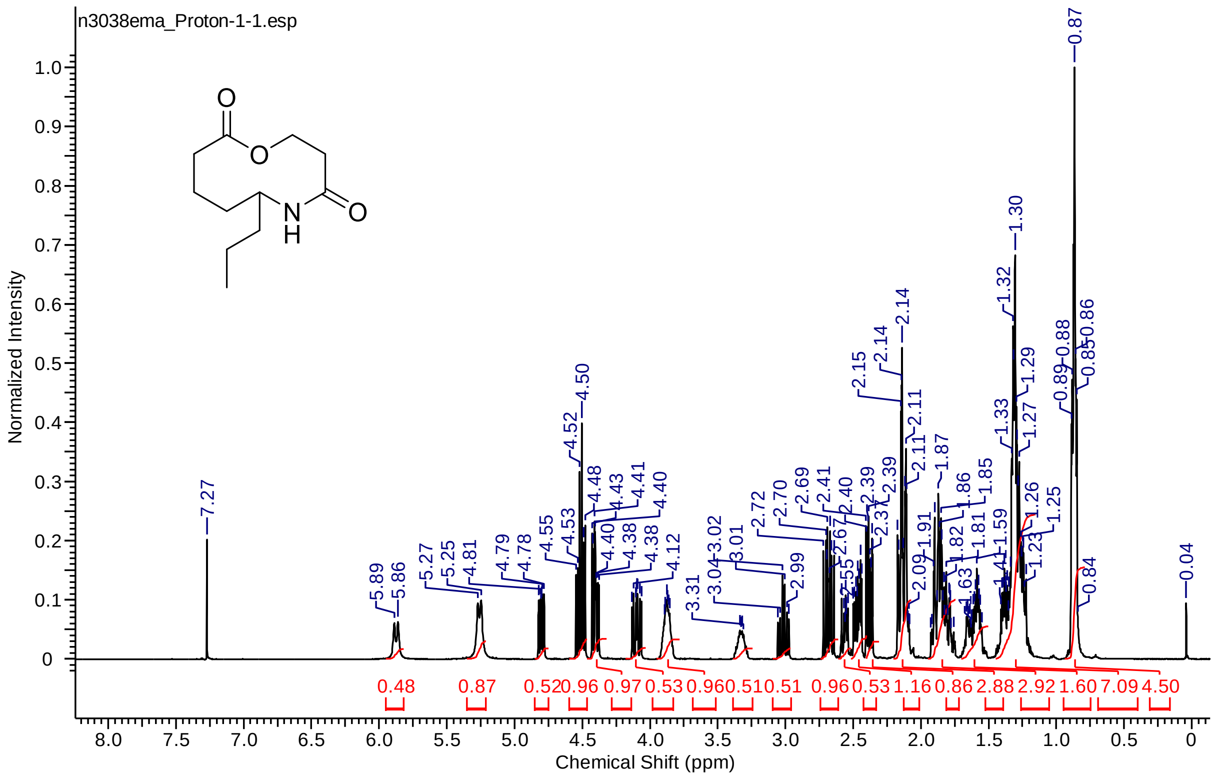

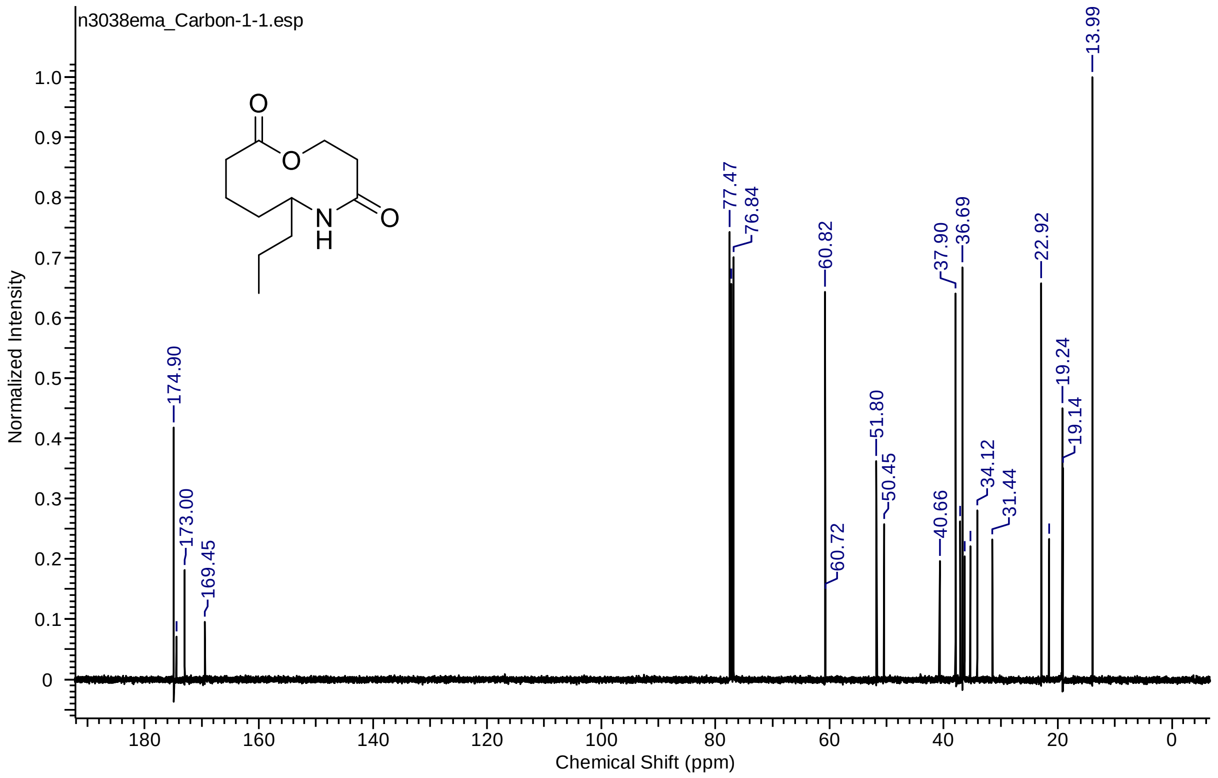

Compound 78

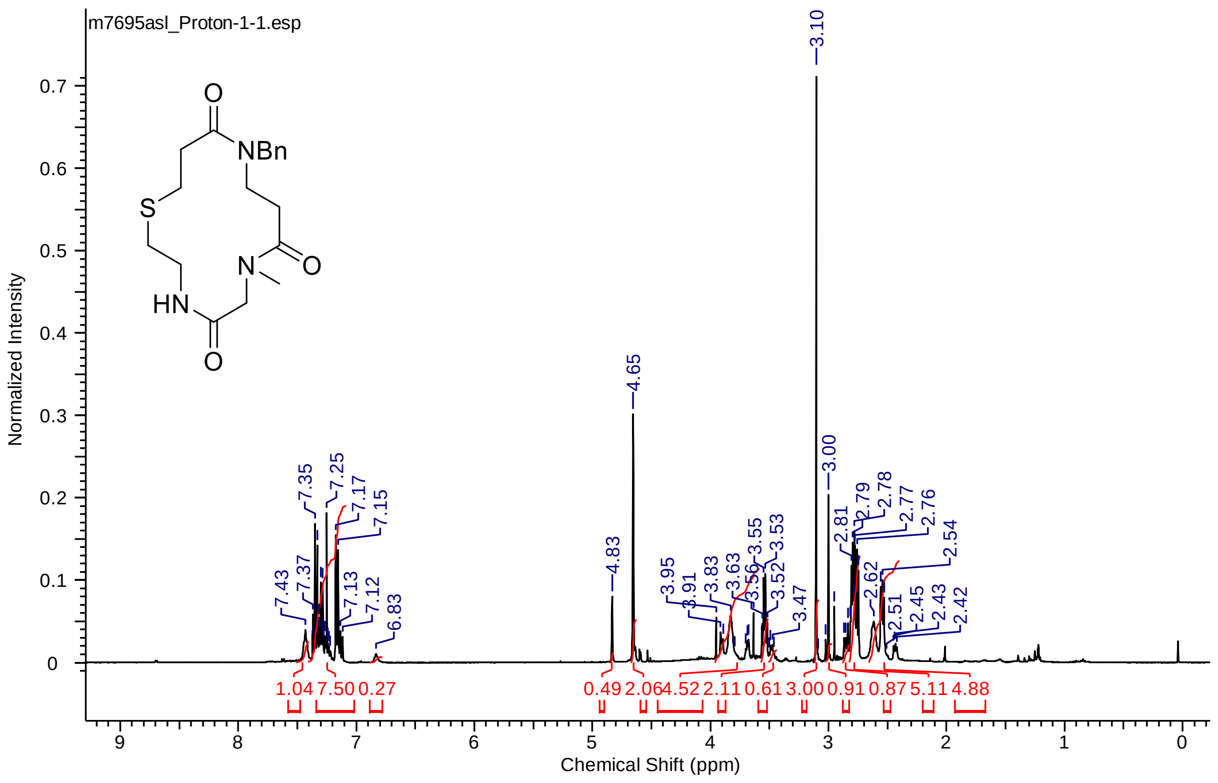

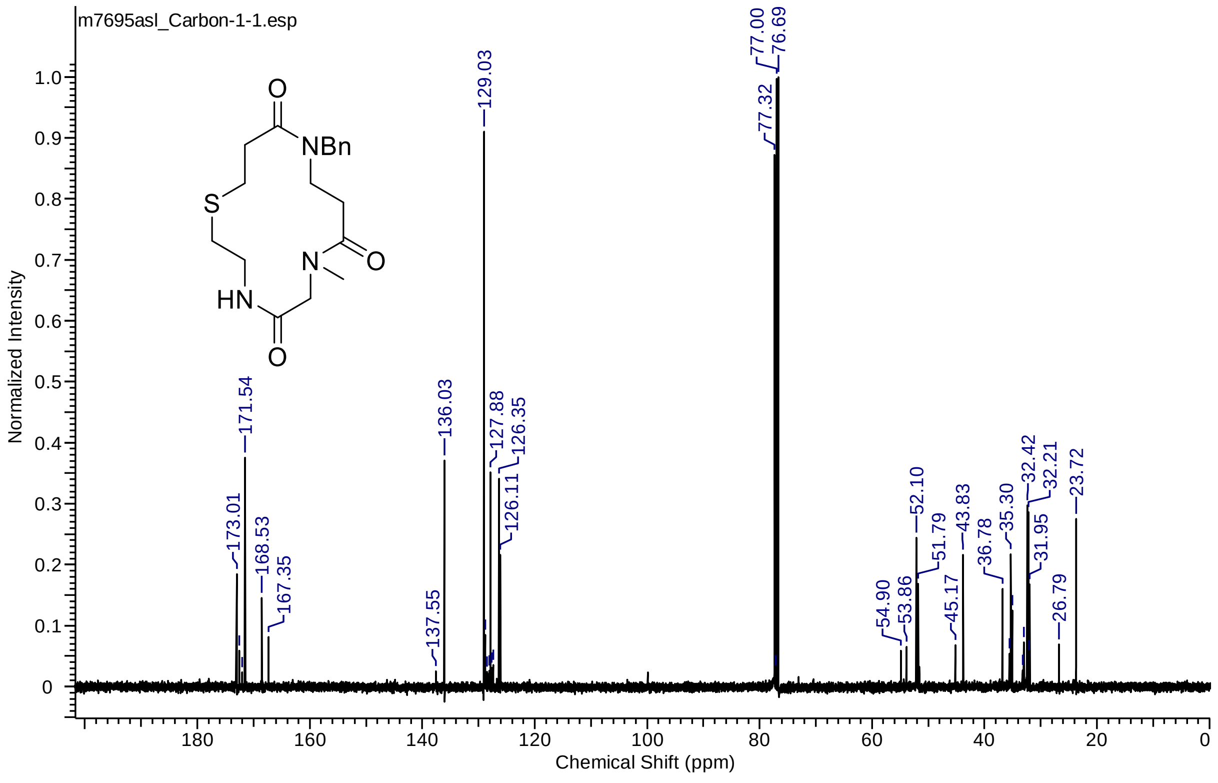

Compound 79

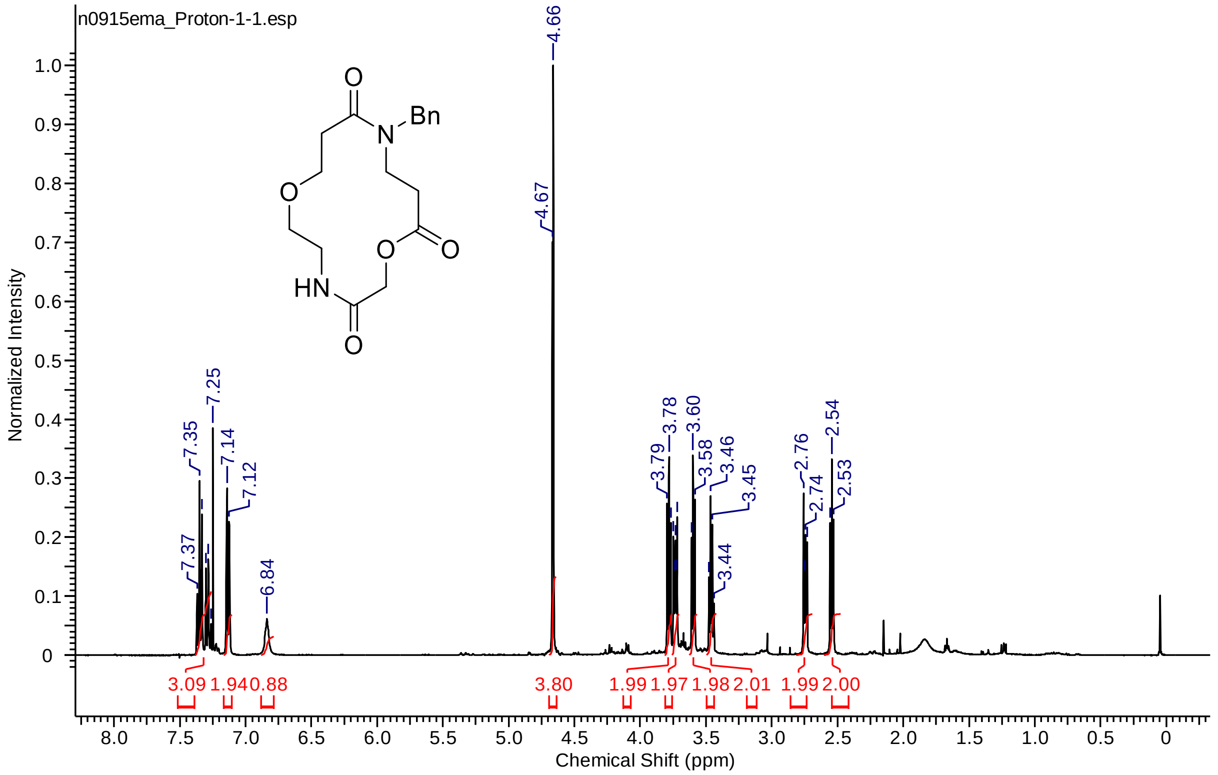
n0915ema_Proton-1-1.esp
Normalized Intensity
Chemical Shift (ppm)
7.37
7.35
7.25
7.14
7.12
6.84
4.67
4.66
3.79
3.78
3.60
3.58
3.46
3.45
3.44
2.76
2.74
2.54
2.53
3.09
1.94
0.88
3.80
1.99
1.97
1.98
2.01
1.99
2.00
O
N
Bn
O
O
O
HN
O

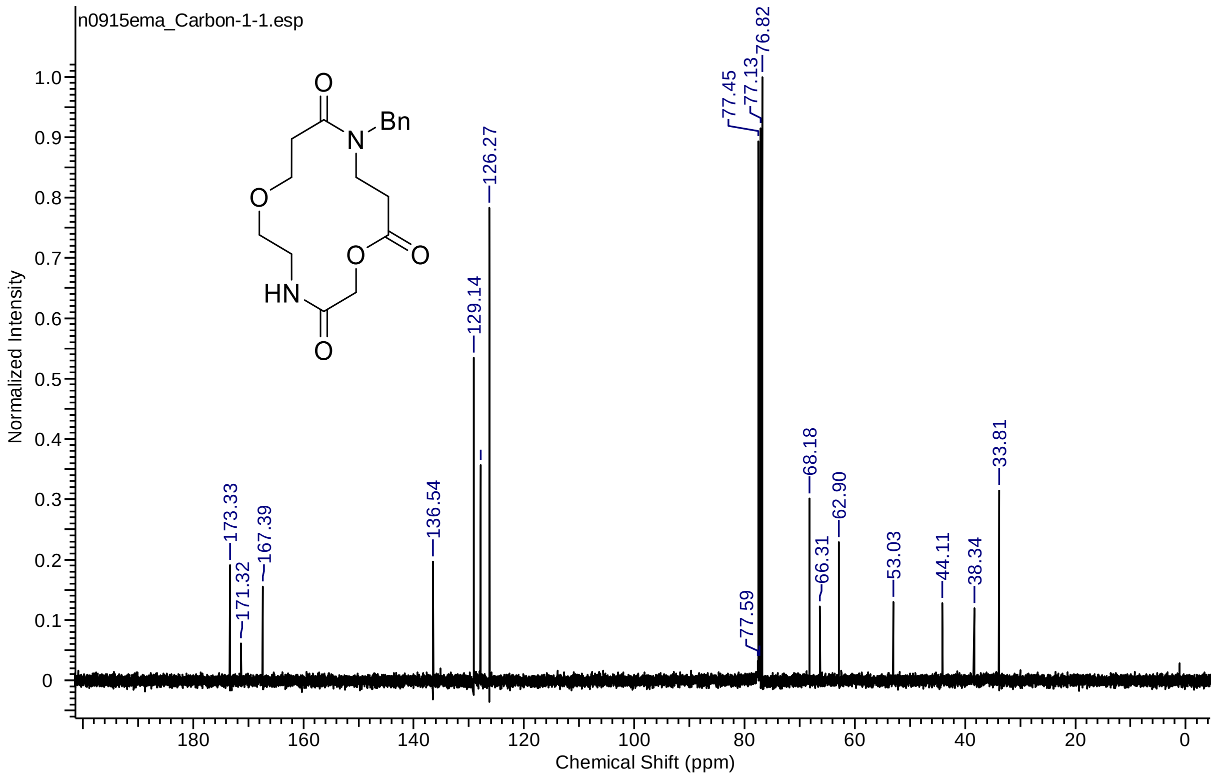
n0915ema_Carbon-1-1.esp
Normalized Intensity
Chemical Shift (ppm)
173.33
171.32
167.39
136.54
129.14
126.27
77.45
77.13
76.82
77.59
68.18
66.31
62.90
53.03
44.11
38.34
33.81
O
N
Bn
O
O
O
HN
O

Compound 80

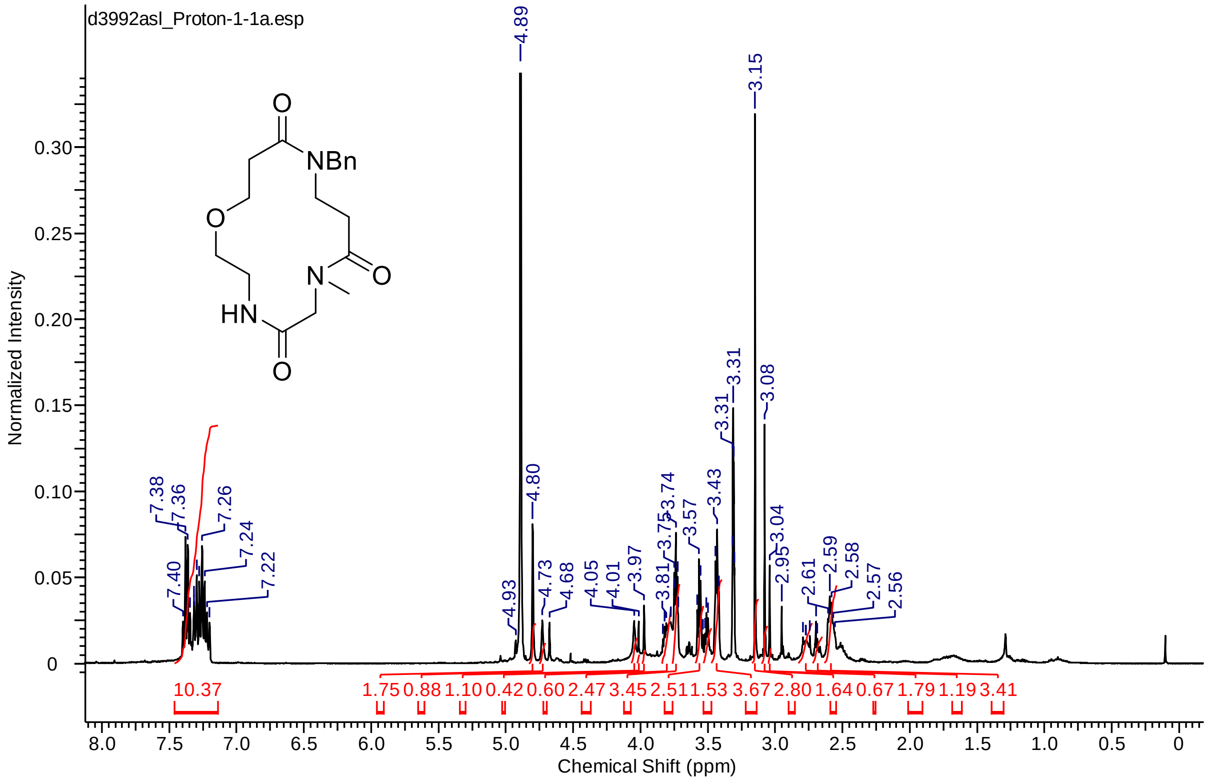

d3992asl_Proton-1-1a.esp
Normalized Intensity
Chemical Shift (ppm)
O
NBn
O
N
O
HN
O
4.89
3.15
3.31
3.31
3.08
7.38
7.36
7.26
7.24
7.22
7.40
4.80
4.93
4.73
4.68
4.05
4.01
3.97
3.81
3.75
3.74
3.57
3.43
3.04
2.95
2.61
2.59
2.58
2.57
2.56
10.37
1.75
0.88
1.10
0.42
0.60
2.47
3.45
2.51
1.53
3.67
2.80
1.64
0.67
1.79
1.19
3.41

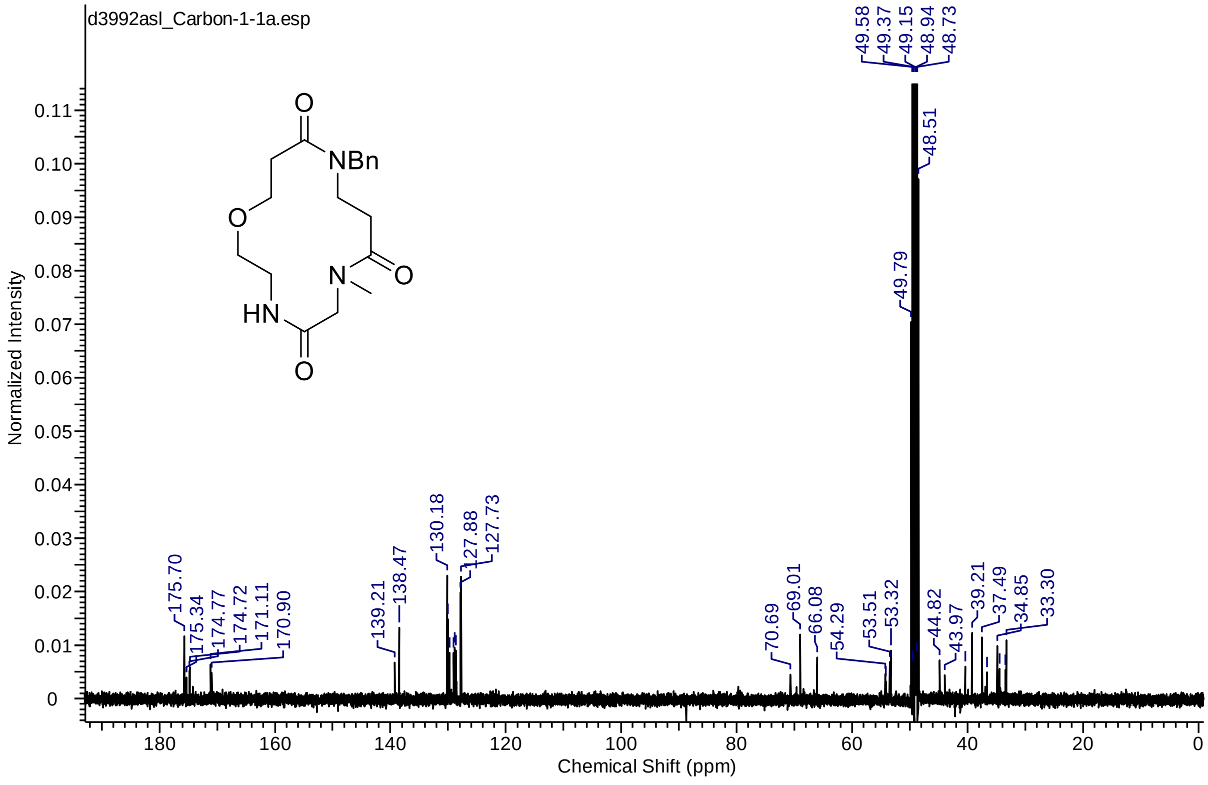

d3992asl_Carbon-1-1a.esp
Normalized Intensity
Chemical Shift (ppm)
O
NBn
O
N
O
HN
O
49.58
49.37
49.15
48.94
48.73
48.51
49.79
175.70
175.34
174.77
174.72
171.11
170.90
139.21
138.47
130.18
127.88
127.73
70.69
69.01
66.08
54.29
53.51
53.32
44.82
43.97
39.21
37.49
34.85
33.30

Computational studies

Spartan Calculations Method

The imides, cyclols and ring expanded products in the selected systems were initially built using Spartan'14[10] and optimised using Density Functional Theory (DFT)/B3LYP/6-31G*[11] in vacuum. Conformational searches of the optimised structures were performed at Molecular Mechanics Force Field (MMFF) level.[12] All the generated structures were retained and their energies were calculated using DFT/B3LYP/6-31G*. The lowest energy geometry in each case was selected, fully optimised and determined to be minima by the absence of negative vibrational modes, in vacuum using DFT/B3LYP/6-31G*. The final optimisations and frequency calculations were also done in vacuum using DFT/B3LYP/6-31G*. XYZ coordinates of the final optimised structures are reported.

Gaussian Calculations Method

All calculations were performed using the Gaussian 09, Revision D.01 package.[13]

The structures from the Spartan calculations were reoptimized using the stated functional (B3LYP, BP86, PBE0, M06 and M06-2X) and basis set (6-31G*, SV(P) or def2-TZVPP). All minima were confirmed as such by the absence of imaginary frequencies. The SCF energies were corrected for their zero-point energies, thermal energies and entropies (obtained from the frequency calculations). For the D3(BJ)-PBE(0)/def2-TZVPP//BP86/SV(P) energies, single-point calculations on the BP86/SV(P) optimised geometries were performed using the hybrid PBE0 functional and the flexible def2-TZVPP basis set, the thermodynamic corrections supplied from the BP86/SV(P) calculations, and dispersion effects were modelled with Grimme's D3 method with additional Becke-Johnson damping.[14] No symmetry constraints were applied during optimisations. Where used, solvent corrections were applied with the Polarisable Continuum Model (PCM) using the integral equation formalism variant (IEFPCM).[15] Energies and xyz coordinates are reported.

Gaussian and Spartan B3LYP/6-31G* Comparison

Due to using Gaussian to perform the thorough method assessment, whilst the initial B3LYP/6-31G* calculations were performed in Spartan, it was deemed necessary to compare the relative energies from each software using the same methodology to confirm the numbers were comparable. The structures were reoptimized and the relative energies for compounds **17-20** are reported below (Table S1). Pleasingly the relative energies are within the margin of error expected in calculations such as these, demonstrating that differences in the relative energies aren't caused by using different software packages.

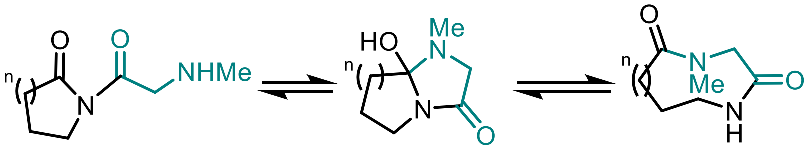

		RO (kcal/mol)	RC (kcal/mol)	RE (kcal/mol)	Yield RE (%)
**17** (n=1)	**Spartan B3LYP/6-31G***	0.0	16.5	1.9	0
	**Gaussian B3LYP/6-31G***	0.0	15.8	2.2	
**18** (n=2)	**Spartan B3LYP/6-31G***	0.0	3.9	2.1	0
	**Gaussian B3LYP/6-31G***	0.0	1.3	-0.3	
**19** (n=3)	**Spartan B3LYP/6-31G***	0.0	6.4	0.7	0
	**Gaussian B3LYP/6-31G***	0.0	6.1	0.3	
**20** (n=4)	**Spartan B3LYP/6-31G***	7.5	14.5	0.0	82
	**Gaussian B3LYP/6-31G***	8.2	14.4	0.0	

Table S1. Relative free energies (ΔG°) of the lowest energy geometries of $\mathbf{17_{RO}}$/$\mathbf{17_{RC}}$/$\mathbf{17_{RE}}$ at DFT/B3LYP/6-31G* in vacuum in kca8l/mol, using both Spartan and Gaussian.

Isomers 17_{RO}/17_{RC}/17_{RE}

Spartan Calculations

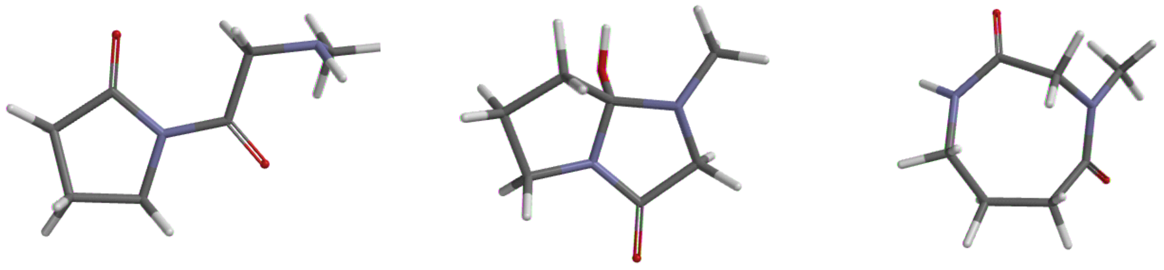

17_{RO}
ΔG° = 0.0 kcal/mol

17_{RC}
ΔG° = 16.5 kcal/mol

17_{RE}
ΔG° = 1.9 kcal/mol

17_{RO}	Calculated energy (au)	Calculated energy (kcal/mol)	Relative energy (kcal/mol)
M0003	-533.933949	-335043.55	0.00
M0002	-533.933541	-335043.30	0.26
M0004	-533.932052	-335042.36	1.19
M0009	-533.931573	-335042.06	1.49
M0007	-533.931013	-335041.71	1.84
M0005	-533.930778	-335041.56	1.99
M0006	-533.930536	-335041.41	2.14
M0013	-533.928395	-335040.07	3.49
M0008	-533.928373	-335040.05	3.50
M0014	-533.928185	-335039.94	3.62
M0010	-533.928052	-335039.85	3.70
M0011	-533.927945	-335039.79	3.77
M0012	-533.927803	-335039.70	3.86
M0015	-533.927631	-335039.59	3.96
M0016	-533.923379	-335036.92	6.63
M0017	-533.923015	-335036.69	6.86
**17_{Rc}**	**Calculated energy (au)**	**Calculated energy (kcal/mol)**	**Relative energy (kcal/mol)**
M0006	-533.912864	-335030.32	0.00
M0003	-533.912142	-335029.87	0.45
M0004	-533.912142	-335029.87	0.45
M0009	-533.910942	-335029.12	1.21
M0001	-533.910868	-335029.07	1.25
M0005	-533.909303	-335028.09	2.23
M0002	-533.905991	-335026.01	4.31
M0007	-533.903578	-335024.50	5.83
M0008	-533.903578	-335024.50	5.83
M0010	-533.902780	-335023.99	6.33
**17_{RE}**	**Calculated energy (au)**	**Calculated energy (kcal/mol)**	**Relative energy (kcal/mol)**
M0001	-533.934615	-335043.97	0.00
M0002	-533.932162	-335042.43	1.54

M0003	-533.922900	-335036.62	7.35
M0004	-533.914759	-335031.51	12.46

Table S2. Energies calculated at DFT/B3LYP/6-31G* in vacuum for the conformers found for $\mathbf{17_{RO}}$/$\mathbf{17_{RC}}$/$\mathbf{17_{RE}}$.

Isomer	ΔG° (au)	ΔG° (kcal/mol)	ΔG° (kcal/mol)
$\mathbf{17_{RO}}$	-533.783075	-334948.88	0.00
$\mathbf{17_{RC}}$	-533.756817	-334932.40	16.5
$\mathbf{17_{RE}}$	-533.780095	-334947.01	1.9

Table S3. Relative free energies (ΔG°) of the lowest energy geometries of $\mathbf{17_{RO}}$/$\mathbf{17_{RC}}$/$\mathbf{17_{RE}}$ at DFT/B3LYP/6-31G* in vacuum in kcal/mol.

XYZ Coordinates

17$_{RO}$

C	1.36799800	0.95801900	0.02426000
C	2.82289900	0.68904100	0.32478900
C	2.99162400	-0.76531300	-0.07836100
C	1.62838400	-1.36299300	0.18841000
N	0.70887800	-0.27718900	-0.05277300
H	2.99629400	0.83595900	1.39519300
H	3.47199200	1.34707600	-0.25790900
H	3.22079800	-0.82473900	-1.14970900
H	3.79350600	-1.27071300	0.46675300
H	1.52084600	-1.67294800	1.23424900
H	1.41339800	-2.22067900	-0.45656100
O	0.88576500	2.07203200	-0.09425800
C	-0.64841100	-0.53116300	-0.21481500
O	-1.08972900	-1.68554800	-0.20583100
C	-1.56570800	0.68677700	-0.42565900
H	-1.43238300	1.41486000	0.37999900
H	-1.28740700	1.15092200	-1.37753500
N	-2.98686300	0.28087000	-0.51883700
H	-3.01617000	-0.61714800	-1.01060100
C	-3.57379700	0.09326000	0.80324400
H	-3.57882500	1.03160800	1.36723100
H	-3.04794400	-0.66973400	1.38543300
H	-4.61442400	-0.22787500	0.69424200

17_{RC}

C	0.07433100	-0.65920900	0.11160100
N	-0.65413300	0.54828800	0.27721100
C	-2.05332300	0.43080300	-0.04727900
C	-2.25008800	-1.07327700	-0.21943900
C	-0.87100600	-1.54242200	-0.70588900
H	-2.66374400	0.85652100	0.75345100
H	-2.24944500	0.97223300	-0.97906900
H	-3.05442700	-1.31546000	-0.91928900
H	-2.48190600	-1.53782800	0.74657100
H	-0.78363700	-1.33325200	-1.77964900
H	-0.72095200	-2.61361200	-0.54204900
O	0.27713300	-1.17674800	1.40419100
H	0.56734600	-2.09251700	1.28399100
C	0.12607300	1.66144100	0.07145100
O	-0.23706100	2.82353000	0.01204100
N	1.30333000	-0.26395500	-0.53977900
C	1.53433500	1.13232600	-0.11259900
H	2.05150300	1.69466800	-0.89423900
H	2.06337400	1.21009800	0.84368100
C	2.45442300	-1.11153100	-0.30365900
H	3.30685100	-0.76527800	-0.89852900
H	2.24678600	-2.13986100	-0.61892900
H	2.76483300	-1.12908900	0.74708100

17_{RE}

C	1.66190300	1.55028600	0.10014100
H	1.77024300	1.54044600	1.18939100
H	2.46786800	2.17678000	-0.29879900
C	0.32270800	2.14400700	-0.32667900
H	0.13620600	1.94106900	-1.38823900
H	0.40636600	3.23338700	-0.22119900
C	-0.86463600	1.70332700	0.52541100
H	-0.61859700	1.62521500	1.58806100
H	-1.63534000	2.48460300	0.47139100
C	-1.52219600	0.43655200	0.01373100
N	-1.00065600	-0.78499200	0.40618100
O	-2.49555500	0.52838000	-0.73720900
C	0.29320300	-0.93808300	1.06861100
H	0.30538500	-1.88275300	1.62395100
H	0.46538900	-0.15055400	1.80239100
C	1.40456300	-0.96626200	0.02530100
O	1.79924400	-2.01614500	-0.48191900
N	1.90845200	0.22229400	-0.41855900
H	2.60645100	0.11160900	-1.14615900
C	-1.56015600	-1.98774800	-0.19529900
H	-0.99036800	-2.23396200	-1.09594900
H	-2.60851400	-1.84348900	-0.47167900
H	-1.50150200	-2.81382800	0.51923100

Gaussian Calculations

17_{RO}

B3LYP/6-31G*

SCF Done: E(RB3LYP) = -533.939535034

Zero-point correction= 0.195633
Thermal correction to Gibbs Free Energy= 0.158153

C	1.35999200	0.98468900	0.03603500
C	2.83074900	0.70611100	0.33290100
C	3.02723000	-0.76522000	-0.05681800
C	1.63597900	-1.38968100	0.15411200
N	0.71471800	-0.25786500	-0.05470000
H	2.99056800	0.87109100	1.40705100
H	3.46468400	1.41582700	-0.20379000
H	3.30989200	-0.83813000	-1.11270300
H	3.79696800	-1.27289000	0.53000600
H	1.50658100	-1.78719200	1.16816100
H	1.39931700	-2.19146000	-0.54698000
O	0.83636500	2.07397300	-0.08462100
C	-0.65437000	-0.51122300	-0.22958100
O	-1.04526900	-1.66803200	-0.21385000
C	-1.60873000	0.66394200	-0.48828600
H	-1.43586200	1.45863500	0.24203800
H	-1.33417900	1.10470700	-1.45664700
N	-3.00588200	0.27938200	-0.48908100
H	-3.10178900	-0.57828800	-1.02972300
C	-3.54894700	0.04702500	0.84981200
H	-3.53401300	0.98853500	1.41169900
H	-3.01038600	-0.71541900	1.43645200
H	-4.59381400	-0.26742600	0.75961400

B3LYP/6-31G* with Solvent Correction

SCF Done: E(RB3LYP) = -533.948511086

Zero-point correction= 0.195486
Thermal correction to Gibbs Free Energy= 0.158102

C	1.36126000	0.98050800	0.03695800
C	2.82787400	0.70442300	0.33541500
C	3.02798300	-0.76535700	-0.05678400
C	1.63850700	-1.39269100	0.14919000
N	0.71297500	-0.25897000	-0.05494300
H	2.98245700	0.86558500	1.41075200
H	3.46389100	1.41435000	-0.19830000
H	3.31423000	-0.83605200	-1.11117700
H	3.79386700	-1.27091400	0.53540300
H	1.50712800	-1.79146400	1.16152800
H	1.40707900	-2.18833400	-0.55998400
O	0.83414600	2.07216200	-0.08535300
C	-0.65141200	-0.50630200	-0.22952200
O	-1.05162900	-1.66308400	-0.22174300
C	-1.60369900	0.67253100	-0.47580900
H	-1.43348500	1.45229200	0.27076400
H	-1.32430500	1.12420200	-1.43707700
N	-3.00534100	0.29418800	-0.48838400
H	-3.10136700	-0.55204600	-1.04732000
C	-3.54898300	0.02973800	0.84703700
H	-3.53336800	0.95660300	1.43232800
H	-3.00637200	-0.74353400	1.41382300
H	-4.59250800	-0.28693600	0.75040200

BP86/6-31G*

SCF Done: E(RB-P86) = -533.939444578

Zero-point correction= 0.189470
Thermal correction to Gibbs Free Energy= 0.151666

C	1.36023800	0.99362700	0.03412100
C	2.83684900	0.71275500	0.33708700
C	3.03597300	-0.76118400	-0.05907400
C	1.64382100	-1.39176000	0.16342400
N	0.71518000	-0.26160300	-0.05742100
H	2.99381300	0.87444900	1.42165300
H	3.47810100	1.43172500	-0.19686300
H	3.31067800	-0.83056200	-1.12663800
H	3.81842500	-1.27341600	0.52369600
H	1.51632600	-1.77885100	1.19200400
H	1.40164400	-2.20964300	-0.53201100
O	0.82672900	2.09014700	-0.08971100
C	-0.66016900	-0.52345300	-0.23533500
O	-1.05108400	-1.69271900	-0.20799700
C	-1.61745200	0.65128500	-0.51711000
H	-1.43084200	1.47610800	0.19097900
H	-1.34999700	1.06589300	-1.51024600
N	-3.02188700	0.26765400	-0.49308100
H	-3.10630700	-0.62815000	-0.99209400
C	-3.53485400	0.07398400	0.87138300
H	-3.51835800	1.04320900	1.40277500
H	-2.97178100	-0.66735700	1.48090000
H	-4.58634800	-0.25670900	0.81403800

PBE0/6-31G*

SCF Done: E(RPBE-PBE) = -533.298475598

Zero-point correction= 0.190196
Thermal correction to Gibbs Free Energy= 0.152500

C	1.36127900	0.99080700	0.03620900
C	2.83430900	0.70468300	0.33634100
C	3.02727800	-0.76498000	-0.06518500
C	1.63708500	-1.39004600	0.16146200
N	0.71371100	-0.26036100	-0.05507400
H	2.99154900	0.86053600	1.42086000
H	3.47727100	1.42380400	-0.19350200
H	3.29556600	-0.83081100	-1.13371500
H	3.81068100	-1.28158700	0.51062400
H	1.51258400	-1.77701200	1.18980300
H	1.38987200	-2.20726600	-0.53206800
O	0.83129200	2.08773300	-0.08585500
C	-0.65974100	-0.51766000	-0.23245200
O	-1.05380200	-1.68473400	-0.20985700
C	-1.61271100	0.65856900	-0.50557800
H	-1.43000900	1.47417000	0.21347100
H	-1.33863700	1.08711400	-1.49007600
N	-3.01409900	0.27622100	-0.49353700
H	-3.09649500	-0.61084800	-1.00581000
C	-3.53097600	0.06197400	0.86232600
H	-3.51429100	1.02148500	1.40943500
H	-2.97215300	-0.68983600	1.46130500
H	-4.58228500	-0.26484000	0.79691000

M06/6-31G* with Solvent Correction

SCF Done: E(RM06) = -533.611702101

Zero-point correction=   0.194716
Thermal correction to Gibbs Free Energy=   0.157513

C	1.35233500	0.97743900	0.03712000
C	2.80536200	0.69600500	0.33763000
C	3.00406300	-0.75580400	-0.07584300
C	1.63162500	-1.38313800	0.15073700
N	0.70754800	-0.26008300	-0.05689500
H	2.94693200	0.83467700	1.42004600
H	3.44849400	1.42068600	-0.17066600
H	3.26548100	-0.81020500	-1.14014800
H	3.78903800	-1.26750800	0.48837400
H	1.50989900	-1.77106600	1.17164300
H	1.39275400	-2.19132500	-0.54601800
O	0.82750200	2.06386800	-0.08348100
C	-0.65346400	-0.51120900	-0.21912500
O	-1.05368500	-1.66133200	-0.19723800
C	-1.58670300	0.66227100	-0.47013200
H	-1.42280700	1.43888100	0.28734600
H	-1.28529300	1.12909800	-1.42061100
N	-2.98008600	0.28908400	-0.49919900
H	-3.07213200	-0.56243700	-1.05030000
C	-3.52121400	0.04348400	0.82968600
H	-3.51317500	0.98111200	1.40165100
H	-2.97028900	-0.71565300	1.41212600
H	-4.56370500	-0.28384300	0.74452600

M06-2X/6-31G* with Solvent Correction

SCF Done: E(RM062X) = -533.719841258

Zero-point correction= 0.197994
Thermal correction to Gibbs Free Energy= 0.161147

C	1.35697300	0.97209100	0.04153900
C	2.81567100	0.68408500	0.34496900
C	3.00269000	-0.77428400	-0.07695200
C	1.61654900	-1.39488600	0.13953300
N	0.70555600	-0.25838800	-0.05492800
H	2.95496400	0.81533800	1.42479900
H	3.45801300	1.40064500	-0.16817500
H	3.26574000	-0.82425600	-1.13692600
H	3.77422500	-1.29282600	0.49288100
H	1.49098600	-1.79268300	1.15191200
H	1.37437700	-2.18396300	-0.57241000
O	0.83608000	2.05943700	-0.08034400
C	-0.65587900	-0.48812800	-0.22542200
O	-1.06849800	-1.63316000	-0.23187300
C	-1.58742700	0.70504300	-0.44202400
H	-1.42782800	1.44452700	0.34731200
H	-1.28639100	1.19777600	-1.37410300
N	-2.98255300	0.32523100	-0.48817000
H	-3.06660000	-0.49388000	-1.08726400
C	-3.50762300	-0.01663500	0.83225900
H	-3.50792500	0.88172300	1.45786300
H	-2.93524600	-0.79956500	1.34992500
H	-4.54171200	-0.35465300	0.73020400

D3(BJ)-PBE0/def2-TZVPP//BP86/SVP with Solvent Correction

SCF Done: E(RB-P86) = -533.564175432
SCF Done: E(RPBE-PBE) = -533.540283055

Zero-point correction= 0.187789
Thermal correction to Gibbs Free Energy= 0.150031

C	1.36490200	0.98927500	0.03976800
C	2.83909800	0.71021700	0.32631900
C	3.03612500	-0.75863200	-0.07664900
C	1.65191600	-1.38926300	0.16754400
N	0.71678500	-0.26327100	-0.04387000
H	2.99755700	0.86897000	1.41710500
H	3.47953400	1.44045300	-0.20511200
H	3.29444000	-0.82485800	-1.15402600
H	3.83312600	-1.27319400	0.49333400
H	1.54095800	-1.77328800	1.20554800
H	1.40238800	-2.21803700	-0.52173700
O	0.82847800	2.08258600	-0.07729900
C	-0.65365800	-0.52198500	-0.22310000
O	-1.05228000	-1.68546600	-0.20009800
C	-1.60984900	0.65292100	-0.49858900
H	-1.41868700	1.47215100	0.22429100
H	-1.31659200	1.08217600	-1.48483400
N	-3.01094200	0.27810500	-0.49653400
H	-3.11453600	-0.58851200	-1.04207800
C	-3.56745000	0.06302100	0.83992800
H	-3.55320300	1.01764100	1.40916800
H	-3.03442800	-0.70452400	1.45580300
H	-4.62753300	-0.25309700	0.75321500

17_{RC}

B3LYP/6-31G*

SCF Done: E(RB3LYP) = -533.917449470

Zero-point correction= 0.196145
Thermal correction to Gibbs Free Energy= 0.161220

C	0.10900400	-0.66579800	0.11004500
N	-0.66763400	0.53963100	0.26991900
C	-2.09831000	0.35405700	0.03668500
C	-2.20842600	-1.15721100	-0.28577800
C	-0.79576500	-1.55122500	-0.76969100
H	-2.68780700	0.63736000	0.91396400
H	-2.41108100	0.99226300	-0.79832600
H	-2.98430800	-1.36442500	-1.02807200
H	-2.45465400	-1.71279600	0.62418300
H	-0.64039600	-1.26900800	-1.81572000
H	-0.59366200	-2.62286900	-0.66686400
O	0.27240900	-1.24138100	1.41579200
H	0.61366800	-2.14302800	1.30204700
C	0.05986300	1.68126400	0.06069800
O	-0.37701700	2.81458200	-0.01453500
N	1.35380800	-0.18957000	-0.48706400
C	1.50953400	1.21169300	-0.08875600
H	2.02433800	1.79913100	-0.85550900
H	2.04692700	1.34366200	0.86756000
C	2.52351200	-1.02846800	-0.29984500
H	3.35025300	-0.62569200	-0.89387500
H	2.32029900	-2.04059700	-0.67044800
H	2.85359800	-1.09592000	0.75086700

B3LYP/6-31G* with Solvent Correction

SCF Done: E(RB3LYP) = -533.927832863

Zero-point correction= 0.196057
Thermal correction to Gibbs Free Energy= 0.161163

C	0.09983100	-0.66823200	0.10979000
N	-0.66308800	0.55524300	0.24754600
C	-2.09865200	0.38208600	0.01558400
C	-2.22529000	-1.13356800	-0.27565900
C	-0.81757700	-1.55639000	-0.75008800
H	-2.68596400	0.69195400	0.88502000
H	-2.40114000	1.00194200	-0.83630900
H	-3.00114200	-1.34305500	-1.01638400
H	-2.48313100	-1.66792400	0.64353500
H	-0.66135600	-1.29678400	-1.80167700
H	-0.62934200	-2.62702000	-0.62526500
O	0.25580300	-1.21162000	1.42952400
H	0.55554500	-2.13154700	1.34255500
C	0.08606900	1.67584100	0.05525300
O	-0.32720600	2.82639200	-0.00690200
N	1.34951500	-0.21303300	-0.49352800
C	1.52706000	1.18455100	-0.09039600
H	2.05747800	1.76459000	-0.85229800
H	2.06368000	1.30695000	0.86759100
C	2.50937300	-1.07044200	-0.30487300
H	3.34374200	-0.67282900	-0.89105200
H	2.29264200	-2.07699100	-0.67953200
H	2.83034900	-1.14601600	0.74704500

BP86/6-31G*

SCF Done: E(RB-P86) = -533.918710671

Zero-point correction= 0.189893
Thermal correction to Gibbs Free Energy= 0.154721

C	0.10855700	-0.67012400	0.10615500
N	-0.66894300	0.54208400	0.27056600
C	-2.10487400	0.35715000	0.03479400
C	-2.21697200	-1.16038100	-0.27953600
C	-0.80433400	-1.55474400	-0.77696400
H	-2.69981100	0.65028100	0.91683900
H	-2.41549900	0.99851100	-0.81122200
H	-3.00775500	-1.37490700	-1.01721000
H	-2.45164700	-1.71530700	0.64469500
H	-0.65200500	-1.26221400	-1.83045700
H	-0.60131300	-2.63616900	-0.68222500
O	0.27062900	-1.24747200	1.42487000
H	0.61725500	-2.15601100	1.29331900
C	0.06560700	1.69145400	0.06104400
O	-0.37479100	2.83494800	-0.01396000
N	1.35808900	-0.19206100	-0.50139900
C	1.52047300	1.21004900	-0.08535200
H	2.04758900	1.80547700	-0.85087100
H	2.05949500	1.33195900	0.88336100
C	2.52713600	-1.03849000	-0.29732300
H	3.36924100	-0.63404700	-0.88510800
H	2.32331000	-2.05724600	-0.67650500
H	2.84686100	-1.10978200	0.76703500

PBE0/6-31G*

SCF Done: E(RPBE-PBE) = -533.279098703

Zero-point correction= 0.190628
Thermal correction to Gibbs Free Energy= 0.155509

C	0.10919500	-0.66834600	0.10609000
N	-0.66870800	0.54013500	0.27058400
C	-2.10006000	0.35338800	0.03299100
C	-2.20905500	-1.16178700	-0.27598400
C	-0.80053500	-1.55080700	-0.77768500
H	-2.69788400	0.64875700	0.91145700
H	-2.40942300	0.99119300	-0.81543100
H	-3.00302800	-1.38164300	-1.00719400
H	-2.43482100	-1.71410400	0.65112100
H	-0.65069200	-1.25329900	-1.82928200
H	-0.59405600	-2.63105900	-0.68756400
O	0.27058000	-1.24621500	1.42079400
H	0.62052900	-2.15185300	1.28651900
C	0.06343600	1.68865900	0.06164800
O	-0.37827900	2.82996600	-0.01568000
N	1.35576000	-0.18878700	-0.49889400
C	1.51608000	1.20885100	-0.08176600
H	2.04456700	1.80613000	-0.84386900
H	2.05190300	1.32986400	0.88803500
C	2.52145500	-1.03404300	-0.29823400
H	3.36358300	-0.62924500	-0.88415100
H	2.31733100	-2.05095500	-0.67994000
H	2.84111200	-1.10872700	0.76520000

M06/6-31G* with Solvent Correction

SCF Done: E(RM06) = -533.598050065

Zero-point correction= 0.195671
Thermal correction to Gibbs Free Energy= 0.161022

C	0.09712000	-0.66217900	0.11213900
N	-0.66587900	0.55353000	0.24595900
C	-2.08831800	0.37646000	-0.01470200
C	-2.20384100	-1.13702500	-0.24433500
C	-0.81136400	-1.54976000	-0.73224900
H	-2.69992800	0.72838200	0.82400600
H	-2.36610800	0.96066600	-0.90401600
H	-2.99983500	-1.38896700	-0.95208900
H	-2.42544000	-1.64036800	0.70497900
H	-0.66670400	-1.28220300	-1.78704300
H	-0.60868500	-2.62127200	-0.61398600
O	0.27100800	-1.18779600	1.41924200
H	0.59065100	-2.09922900	1.33955500
C	0.08750200	1.66967200	0.05762400
O	-0.32171100	2.81390100	-0.01738400
N	1.33099900	-0.20619400	-0.50273500
C	1.51684600	1.16867600	-0.06340400
H	2.08252800	1.76382700	-0.79063900
H	2.02746100	1.24867600	0.91727600
C	2.47697500	-1.06747500	-0.32616600
H	3.31072000	-0.68869400	-0.92910700
H	2.24249600	-2.07916400	-0.68466100
H	2.81311600	-1.13206000	0.72484800

M06-2X/6-31G* with Solvent Correction

SCF Done: E(RM062X) = -533.708685116

Zero-point correction= 0.198556
Thermal correction to Gibbs Free Energy= 0.163852

C	0.10645700	-0.66248400	0.10650300
N	-0.66969200	0.54553600	0.25511400
C	-2.09488300	0.36011100	0.00302700
C	-2.19882900	-1.15893300	-0.24409600
C	-0.79992800	-1.55432500	-0.74767500
H	-2.69827300	0.69089100	0.85120400
H	-2.38217100	0.94703800	-0.87634300
H	-2.99173900	-1.40518600	-0.95210000
H	-2.40751900	-1.67166400	0.69845800
H	-0.66492900	-1.27269100	-1.79556200
H	-0.58814200	-2.62086200	-0.63584600
O	0.28950000	-1.20178300	1.40698800
H	0.62421200	-2.10768800	1.31757300
C	0.06611300	1.66959100	0.05866700
O	-0.35994800	2.80737700	-0.01553000
N	1.33833400	-0.19002600	-0.51000000
C	1.50976100	1.19238900	-0.07115600
H	2.05156700	1.79116700	-0.80742800
H	2.02008800	1.28305300	0.90269900
C	2.49561600	-1.04099300	-0.30947800
H	3.33097600	-0.64782900	-0.89429700
H	2.27811300	-2.04989200	-0.67555900
H	2.80507200	-1.10179000	0.74499500

D3(BJ)-PBE0/def2-TZVPP//BP86/SVP with Solvent Correction

SCF Done: E(RB-P86) = -533.549256956
SCF Done: E(RPBE-PBE) = -533.529000232

Zero-point correction= 0.188533
Thermal correction to Gibbs Free Energy= 0.153373

C	0.08938800	-0.67211800	0.11322000
N	-0.65724400	0.56983100	0.22847200
C	-2.09738600	0.41062600	0.01370100
C	-2.24277900	-1.10733800	-0.27693700
C	-0.84086800	-1.54546900	-0.75979200
H	-2.68615200	0.73062800	0.89847000
H	-2.41030100	1.04615200	-0.84360400
H	-3.04070500	-1.31726100	-1.01570700
H	-2.49819500	-1.64444700	0.65880500
H	-0.67513100	-1.26857300	-1.82089200
H	-0.66078100	-2.63422100	-0.65193100
O	0.22129000	-1.21915100	1.43633800
H	0.43406100	-2.16901700	1.34554800
C	0.11668400	1.68830700	0.05290600
O	-0.27828800	2.85063700	-0.00253000
N	1.35719000	-0.23523000	-0.47175600
C	1.55352700	1.16377300	-0.09162400
H	2.09702600	1.74335100	-0.86816200
H	2.10271000	1.30420800	0.87688500
C	2.49702300	-1.12002100	-0.30398900
H	3.35233100	-0.73600300	-0.89701100
H	2.25557700	-2.13130400	-0.69359100
H	2.83239600	-1.22417100	0.75880100

17$_{RE}$

B3LYP/6-31G*

SCF Done: E(RB3LYP) = -533.938804208

Zero-point correction= 0.197242
Thermal correction to Gibbs Free Energy= 0.197242

C	1.95444100	1.21625400	0.08583100
H	2.09745900	1.20077400	1.17483200
H	2.85802600	1.67374200	-0.32880100
C	0.75259800	2.10996900	-0.26692700
H	0.55821400	2.05461400	-1.34486800
H	1.05899100	3.14302700	-0.05651000
C	-0.55874600	1.85195500	0.50871000
H	-0.34565800	1.74773100	1.58031700
H	-1.20008200	2.72911400	0.39385500
C	-1.41808500	0.69988000	-0.02015300
N	-1.13398100	-0.58021300	0.40680000
O	-2.34858000	0.93952100	-0.78278000
C	0.12056800	-0.94268800	1.06106000
H	-0.03382800	-1.86971400	1.61841300
H	0.41660700	-0.16733100	1.77016700
C	1.20211900	-1.22356800	0.00822100
O	1.34423400	-2.34163400	-0.47260000
N	1.92568400	-0.15006300	-0.43220500
H	2.55386200	-0.38085800	-1.19228300
C	-1.91080800	-1.69352900	-0.13980000
H	-1.31933100	-2.26792000	-0.86134800
H	-2.79161300	-1.28186900	-0.62931400
H	-2.21232600	-2.36211800	0.67475700

B3LYP/6-31G* with Solvent Correction

SCF Done: E(RB3LYP) = -533.951146742

Zero-point correction= 0.197323
Thermal correction to Gibbs Free Energy= 0.161020

C	1.93307400	1.25518200	0.09377300
H	2.06604300	1.24360100	1.18264900
H	2.82735000	1.73058200	-0.31715300
C	0.71343400	2.11488400	-0.27728300
H	0.53131400	2.04637900	-1.35656000
H	0.99427700	3.15537100	-0.07332600
C	-0.59822900	1.84118700	0.49520700
H	-0.38743000	1.74806100	1.56729400
H	-1.24984000	2.70991500	0.37402500
C	-1.43761600	0.67140600	-0.01848100
N	-1.11656600	-0.59896600	0.39252400
O	-2.39490900	0.88775200	-0.76543400
C	0.14440300	-0.93078400	1.05532900
H	0.00485400	-1.85502600	1.62014700
H	0.42137400	-0.14588300	1.75968500
C	1.23370500	-1.19226400	0.00643700
O	1.39243700	-2.31560800	-0.47433200
N	1.94194500	-0.11531900	-0.42316800
H	2.58455700	-0.32267900	-1.17867300
C	-1.87810100	-1.73252800	-0.13253000
H	-1.30770600	-2.26886100	-0.89905600
H	-2.80238600	-1.35408600	-0.56451700
H	-2.10430600	-2.42703100	0.68340300

BP86/6-31G*

SCF Done: E(RB-P86) = -533.939954366

Zero-point correction= 0.191007
Thermal correction to Gibbs Free Energy= 0.191007

C	1.98346600	1.18974500	0.09073400
H	2.11627900	1.17346500	1.19080000
H	2.90631000	1.63695100	-0.31765900
C	0.79234700	2.10173100	-0.27522500
H	0.59935400	2.04082600	-1.36246500
H	1.11757000	3.14031800	-0.07129000
C	-0.52868100	1.86845100	0.50092600
H	-0.31517400	1.76689700	1.58312400
H	-1.16017000	2.76370200	0.38175600
C	-1.41469800	0.72640500	-0.02294800
N	-1.14303000	-0.56353800	0.41213800
O	-2.35813800	0.97648500	-0.78526200
C	0.11135400	-0.93921400	1.07154400
H	-0.05780100	-1.86405400	1.64712100
H	0.42147900	-0.14940200	1.77447600
C	1.18474800	-1.24817900	0.00879100
O	1.29380400	-2.37369000	-0.49271700
N	1.94133400	-0.18317700	-0.42321400
H	2.55451600	-0.42048500	-1.20536100
C	-1.93608500	-1.67301600	-0.12944600
H	-1.31615900	-2.31043900	-0.78456900
H	-2.76734300	-1.23760800	-0.70054700
H	-2.32702000	-2.29105800	0.69972900

PBE0/6-31G*

SCF Done: E(RPBE-PBE) = -533.299557317

Zero-point correction= 0.191743
Thermal correction to Gibbs Free Energy= 0.155543

C	1.99220400	1.16564400	0.09001500
H	2.12519300	1.14950600	1.18932000
H	2.91883300	1.60314800	-0.31798900
C	0.81317200	2.08886100	-0.27476800
H	0.61851200	2.02893700	-1.36098400
H	1.14981600	3.12306400	-0.07290300
C	-0.50700700	1.87097200	0.50026800
H	-0.29494500	1.76929600	1.58178700
H	-1.12908100	2.77157800	0.37963400
C	-1.40291600	0.74008000	-0.02240100
N	-1.14610500	-0.55035700	0.41392900
O	-2.34047500	0.99919300	-0.78672600
C	0.10110600	-0.93834900	1.07217900
H	-0.07711500	-1.86142600	1.64650200
H	0.41984900	-0.15354700	1.77563200
C	1.16724900	-1.25806300	0.00904500
O	1.25921900	-2.38232400	-0.49544100
N	1.93547600	-0.20362100	-0.42132600
H	2.54109800	-0.44690400	-1.20597900
C	-1.94677200	-1.64895100	-0.12809700
H	-1.33317900	-2.29097800	-0.78371200
H	-2.77368000	-1.20607000	-0.69863000
H	-2.34306800	-2.26486800	0.69899000

M06/6-31G* with Solvent Correction

SCF Done: E(RM06) = -533.617543185

Zero-point correction= 0.196416
Thermal correction to Gibbs Free Energy= 0.159956

C	2.09389500	0.87872400	0.06596900
H	2.25025500	0.81863300	1.15341200
H	3.06029800	1.17242900	-0.35806500
C	1.06875600	1.95997000	-0.25702900
H	0.85056000	1.94847200	-1.33530200
H	1.54560100	2.92705100	-0.04667000
C	-0.24474600	1.90799100	0.53241800
H	-0.04116000	1.75987700	1.60198000
H	-0.74456300	2.87815500	0.43493400
C	-1.24895600	0.91049900	-0.00956300
N	-1.19704800	-0.38280000	0.44376400
O	-2.09352300	1.26773700	-0.82326200
C	-0.01721000	-0.93989400	1.08852400
H	-0.31432600	-1.82302600	1.66399000
H	0.41644300	-0.21647300	1.78486700
C	0.95859600	-1.39088900	0.01126800
O	0.89570400	-2.51863700	-0.46291800
N	1.80904800	-0.44784100	-0.46802700
H	2.37054500	-0.76898100	-1.24931400
C	-2.11557000	-1.34344400	-0.14908000
H	-1.74443400	-1.72691400	-1.10953000
H	-3.07977400	-0.85888600	-0.31454100
H	-2.23948500	-2.18638700	0.53847400

M06-2X/6-31G* with Solvent Correction

SCF Done: E(RM062X) = -533.724893353

Zero-point correction= 0.199616
Thermal correction to Gibbs Free Energy= 0.163678

C	2.25050100	0.31518000	0.07176400
H	2.37216700	0.21283400	1.15612900
H	3.25839400	0.35112800	-0.34661700
C	1.53885500	1.63040700	-0.25634200
H	1.33316900	1.67436600	-1.33172500
H	2.24336600	2.43846700	-0.03323100
C	0.24541800	1.91596600	0.52758800
H	0.39812500	1.73033600	1.59559600
H	0.00190000	2.97415500	0.41272500
C	-0.97674600	1.18766400	-0.01223500
N	-1.25998000	-0.06497400	0.46363900
O	-1.69897800	1.73078700	-0.83996700
C	-0.24839500	-0.89682800	1.10073900
H	-0.74836000	-1.67631500	1.67797200
H	0.36036500	-0.30246600	1.78162400
C	0.57268800	-1.58040300	0.00870800
O	0.20885200	-2.64748900	-0.47013200
N	1.63361800	-0.88965000	-0.47418000
H	2.08668000	-1.32991500	-1.26609700
C	-2.38173000	-0.77251300	-0.14356900
H	-2.10545800	-1.21830900	-1.10545600
H	-3.19734300	-0.06856600	-0.30004100
H	-2.70100300	-1.56656500	0.53379100

D3(BJ)-PBE0/def2-TZVPP//BP86/SVP with Solvent Correction

SCF Done: E(RB-P86) = -533.571351041
SCF Done: E(RPBE-PBE) = -533.549372323

Zero-point correction= 0.189901
Thermal correction to Gibbs Free Energy= 0.153878

C	1.88733500	1.33821800	0.09494100
H	2.01664100	1.34008400	1.20050300
H	2.77174900	1.86866200	-0.30998000
C	0.62745200	2.14205600	-0.28186300
H	0.45390800	2.06922600	-1.37760100
H	0.86679100	3.20808300	-0.07930500
C	-0.68062100	1.81794000	0.48048500
H	-0.47207200	1.73838200	1.57004600
H	-1.37406800	2.67132700	0.35171400
C	-1.48743400	0.61411400	-0.02236800
N	-1.08234600	-0.64897900	0.36669200
O	-2.48329800	0.78670100	-0.73945500
C	0.18550600	-0.91302200	1.04884100
H	0.08644100	-1.84606500	1.63749000
H	0.42348600	-0.09356900	1.75271700
C	1.29889200	-1.14330500	0.00859200
O	1.50556000	-2.26539900	-0.47151000
N	1.97232600	-0.03217100	-0.41519000
H	2.63342800	-0.21350900	-1.17748700
C	-1.80452700	-1.82106400	-0.12639200
H	-1.14369700	-2.45014200	-0.75903800
H	-2.66563800	-1.46630400	-0.72009500
H	-2.16454700	-2.43816400	0.72482900

Isomers 18_{RO}/18_{RC}/18_{RE}

Spartan Calculations

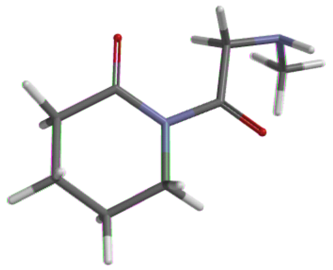

18_{RO}
$\Delta G°$ = 0.0 kcal/mol

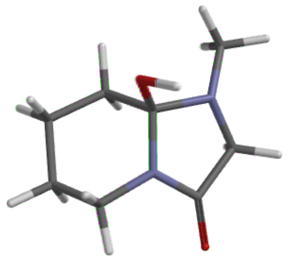

18_{RC}
$\Delta G°$ = 3.9 kcal/mol

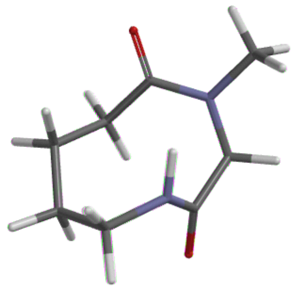

18_{RE}
$\Delta G°$ = 2.1 kcal/mol

18_{RO}	Calculated energy (au)	Calculated energy (kcal/mol)	Relative energy (kcal/mol)
M0002	-573.24174	-359709.1887	0.00
M0005	-573.24058	-359708.4640	0.72
M0003	-573.24051	-359708.4200	0.77
M0001	-573.23990	-359708.0366	1.15
M0012	-573.23857	-359707.2008	1.99
M0009	-573.23841	-359707.1016	2.09
M0004	-573.23809	-359706.9008	2.29
M0007	-573.23777	-359706.7007	2.49
M0008	-573.23700	-359706.2181	2.97
M0010	-573.23613	-359705.6735	3.52
M0015	-573.23572	-359705.4112	3.78
M0006	-573.23529	-359705.1420	4.05
M0014	-573.23506	-359704.9976	4.19
M0013	-573.23443	-359704.6048	4.58
M0011	-573.23395	-359704.3043	4.88
M0017	-573.23081	-359702.3308	6.86
M0016	-573.23028	-359702.0026	7.19
**18_{RC}**	**Calculated energy (au)**	**Calculated energy (kcal/mol)**	**Relative energy (kcal/mol)**
M0001	-573.239237	-359707.6212	0.00
M0004	-573.238487	-359707.1506	0.47
M0003	-573.237261	-359706.3813	1.24
M0002	-573.233602	-359704.0853	3.54
M0005	-573.231453	-359702.7368	4.88
M0011	-573.229976	-359701.8099	5.81
M0006	-573.229445	-359701.4767	6.14
M0009	-573.228786	-359701.0632	6.56
M0007	-573.228187	-359700.6873	6.93
M0010	-573.226962	-359699.9187	7.70
M0008	-573.224033	-359698.0807	9.54

M0012	-573.219744	-359695.3894	12.23
**18_{RE}**	**Calculated energy (au)**	**Calculated energy (kcal/mol)**	**Relative energy (kcal/mol)**
M0001	-573.240546	-359708.4426	0.00
M0002	-573.239455	-359707.7580	0.68
M0003	-573.234248	-359704.4906	3.95

Table S4. Energies calculated at DFT/B3LYP/6-31G* in vacuum for the conformers found for **19_{RO}/19_{RC}/19_{RE}**.

Isomer	ΔG° (au)	ΔG° (kcal/mol)	ΔG° (kcal/mol)
**18_{RO}**	-573.063992	-359597.65	0.0
**18_{RC}**	-573.057732	-359593.73	3.9
**18_{RE}**	-573.060601	-359595.53	2.1

Table S5. Relative free energies (ΔG°) of the lowest energy geometries of **18_{RO}/18_{RC}/18_{RE}** at DFT/B3LYP/6-31G* in vacuum in kcal/mol.

XYZ Coordinates

18_{RO}

C	1.03915400	1.06432500	-0.06919300
C	3.28575500	-0.13650400	0.04846400
C	1.18796200	-1.39826600	-0.25410000
C	2.52481500	-1.37643300	0.46419600
N	0.39845300	-0.17576100	-0.11178200
C	2.47492800	1.08376100	0.42778100
H	3.46519200	-0.14993700	-1.03343000
H	1.34614800	-1.55244600	-1.32958000
H	2.36873400	-1.37603700	1.54986400
H	2.43914300	1.19340400	1.51834900
H	4.26310500	-0.10673200	0.54210900
H	0.60825000	-2.25433900	0.11194700
H	3.09824700	-2.27616600	0.21620700
H	2.96607200	1.97592500	0.02225000
O	0.49406500	2.13942000	-0.31802000
C	-0.96685200	-0.33197100	-0.40971600
O	-1.40865100	-1.38971000	-0.87037200
C	-1.89170500	0.84871600	-0.15223300
H	-1.75736200	1.58046300	-0.95610000
H	-1.63075500	1.30342300	0.81024100
N	-3.29892100	0.42103100	-0.12636200
H	-3.89246200	1.22375300	0.03944200
C	-3.49270100	-0.56274000	0.94996700
H	-2.84989400	-1.43209100	0.77040000
H	-4.54117800	-0.88139600	0.96924200
H	-3.23141200	-0.10771300	1.91220100

18_{RC}

C	-0.26152100	-0.62274200	0.37385900
C	1.94159700	-1.37874500	-0.64893700
C	1.78036400	0.79019000	0.61985500
C	2.46241800	0.05599300	-0.53137200
N	0.33936700	0.67758900	0.51669700
C	0.41166900	-1.39666800	-0.77865700
H	2.24699700	-1.95568800	0.23225600
H	2.08196200	0.35683300	1.57990000
H	2.26624800	0.58617600	-1.47169800
H	0.15030900	-0.94940900	-1.74651300
H	2.39079700	-1.86446000	-1.52240800
H	2.06908800	1.84654300	0.61953400
H	3.54744800	0.05084900	-0.38156300
H	0.07652300	-2.44063000	-0.78999800
O	-0.05808900	-1.30420300	1.58559200
H	-0.57157300	-0.76233400	2.20642300
N	-1.68699700	-0.33613700	0.23109400
C	-2.49014600	-1.27073700	-0.52640800
H	-2.39799800	-2.28164600	-0.11406900
H	-2.22248500	-1.30525700	-1.58868500
H	-3.55201900	-1.00508500	-0.46178400
C	-1.73991600	0.99495200	-0.24289600
C	-0.45274000	1.64613600	-0.04183600
O	-0.20022300	2.79028400	-0.35182600
H	-1.98142900	0.99035100	-1.31194500
H	-2.51430900	1.54467000	0.30422400

18_{RE}

C	1.41250100	0.83970900	-0.25958000
C	-0.85095900	2.01021000	-0.09112000
C	-2.16312900	1.21206100	0.02613000
N	1.47475000	-0.52379100	0.02229000
C	0.20805100	1.38822900	-1.02035000
H	-0.44266800	2.21469000	0.90563000
H	-0.22192900	0.66168000	-1.71169000
H	-1.11704800	2.98522000	-0.52058000
H	-2.43233900	0.80688100	-0.95697000
H	0.61198100	2.18068900	-1.66567000
O	2.26777100	1.62577800	0.15408000
H	-2.96914900	1.90518100	0.29835000
C	-2.15400000	0.08911100	1.07218000
H	-2.13940900	0.51117100	2.08233000
H	-3.05556000	-0.52425900	0.96882000
N	-0.99399000	-0.75488000	0.94884000
C	0.65054900	-1.53597100	-0.66542000
C	-0.81097100	-1.47816000	-0.21517000
O	-1.72099100	-2.00144900	-0.84836000
H	-0.13898000	-0.40180000	1.36764000
C	2.64312000	-1.02589200	0.72956000
H	2.33622900	-1.83742200	1.39597000
H	3.12031000	-0.24611200	1.32908000
H	3.36189900	-1.40099200	-0.00493000
H	0.70479900	-1.35596100	-1.74332000
H	1.03132900	-2.53868100	-0.44572000

Gaussian Calculations

18_{RO}

B3LYP/6-31G*

SCF Done: E(RB3LYP) = -573.244474511

Zero-point correction= 0.224821
Thermal correction to Gibbs Free Energy= 0.185939

C	1.05471600	1.07704000	-0.00233000
C	3.37226700	-0.09264200	-0.11989700
C	1.26575600	-1.44161800	-0.15157600
C	2.65257300	-1.30536600	0.46293900
N	0.46338000	-0.18997700	-0.08570300
C	2.56413600	1.15778600	0.22363300
H	3.45862900	-0.20313400	-1.20941700
H	1.34500300	-1.74419100	-1.20354700
H	2.57315600	-1.20265400	1.55392100
H	2.68585200	1.39573600	1.29028900
H	4.39166600	-0.00190000	0.27204900
H	0.68473400	-2.21664700	0.34865100
H	3.20276500	-2.23318400	0.26788300
H	2.90133900	2.04315100	-0.32328100
O	0.40891800	2.11253000	-0.05694700
C	-0.93576800	-0.40727700	-0.28274800
O	-1.31375900	-1.54382500	-0.49498800
C	-1.91077000	0.76487600	-0.16809800
H	-1.64310500	1.51406800	-0.91663700
H	-1.73522500	1.25697300	0.80642600
N	-3.27986100	0.33291600	-0.38240100
H	-3.83211200	1.15605500	-0.60665600
C	-3.88368400	-0.35968200	0.75202000
H	-3.40672100	-1.33513400	0.87499700
H	-4.94526500	-0.52887600	0.54455500
H	-3.80198200	0.19082000	1.70931800

B3LYP/6-31G* with Solvent Correction

SCF Done: E(RB3LYP) = -573.254313647

Zero-point correction= 0.224720
Thermal correction to Gibbs Free Energy= 0.186002

C	1.36126000	0.98050800	0.03695800
C	2.82787400	0.70442300	0.33541500
C	3.02798300	-0.76535700	-0.05678400
C	1.63850700	-1.39269100	0.14919000
N	0.71297500	-0.25897000	-0.05494300
H	2.98245700	0.86558500	1.41075200
H	3.46389100	1.41435000	-0.19830000
H	3.31423000	-0.83605200	-1.11117700
H	3.79386700	-1.27091400	0.53540300
H	1.50712800	-1.79146400	1.16152800
H	1.40707900	-2.18833400	-0.55998400
O	0.83414600	2.07216200	-0.08535300
C	-0.65141200	-0.50630200	-0.22952200
O	-1.05162900	-1.66308400	-0.22174300
C	-1.60369900	0.67253100	-0.47580900
H	-1.43348500	1.45229200	0.27076400
H	-1.32430500	1.12420200	-1.43707700
N	-3.00534100	0.29418800	-0.48838400
H	-3.10136700	-0.55204600	-1.04732000
C	-3.54898300	0.02973800	0.84703700
H	-3.53336800	0.95660300	1.43232800
H	-3.00637200	-0.74353400	1.41382300
H	-4.59250800	-0.28693600	0.75040200

BP86/6-31G*

SCF Done: E(RB-P86) = -573.241753292

Zero-point correction= 0.217717
Thermal correction to Gibbs Free Energy= 0.178617

C	1.05858400	1.08420300	-0.00168000
C	3.38561600	-0.09528900	-0.12210100
C	1.27363800	-1.44588400	-0.15962800
C	2.65893700	-1.30712900	0.46789600
N	0.46626900	-0.19176500	-0.09133200
C	2.57588600	1.16327400	0.21172900
H	3.47418200	-0.21458500	-1.21971400
H	1.36189800	-1.74235400	-1.22279900
H	2.56948800	-1.19530000	1.56642200
H	2.70587900	1.41793000	1.28315900
H	4.41341200	-0.00377100	0.27345700
H	0.67822600	-2.23020000	0.33126400
H	3.21420000	-2.24471200	0.28301600
H	2.91353800	2.05038800	-0.35075300
O	0.40172400	2.12733400	-0.03994300
C	-0.94285700	-0.41553000	-0.27628900
O	-1.32485300	-1.56675800	-0.46994200
C	-1.91517800	0.76478100	-0.17501400
H	-1.63349000	1.51766300	-0.92966600
H	-1.73771400	1.26803500	0.80651500
N	-3.29006200	0.33344200	-0.39863400
H	-3.83998000	1.17322100	-0.61044500
C	-3.89271200	-0.35188800	0.74928000
H	-3.41158800	-1.33536800	0.87324600
H	-4.96409100	-0.52209100	0.54624100
H	-3.80385300	0.20557400	1.71374100

PBE0/6-31G*

SCF Done: E(RPBE-PBE) = -572.546499107

Zero-point correction=   0.218544
Thermal correction to Gibbs Free Energy=   0.179539

C	1.05749400	1.08236200	0.00036700
C	3.37792300	-0.09763500	-0.12674800
C	1.26834300	-1.44252500	-0.15585600
C	2.65298600	-1.30515700	0.46617500
N	0.46497600	-0.19099600	-0.08953900
C	2.57239700	1.15899000	0.20940100
H	3.46082700	-0.21788600	-1.22376800
H	1.35349100	-1.74281600	-1.21749900
H	2.56685000	-1.19072400	1.56378300
H	2.70532700	1.41214900	1.27988600
H	4.40679600	-0.00756800	0.26346400
H	0.67405300	-2.22525300	0.33785700
H	3.20570400	-2.24316200	0.28206200
H	2.90903300	2.04607400	-0.35200800
O	0.40244500	2.12511600	-0.03565300
C	-0.94111600	-0.41314800	-0.27523900
O	-1.32422400	-1.56285100	-0.46903900
C	-1.91147700	0.76512500	-0.17508400
H	-1.62938800	1.51756000	-0.92921500
H	-1.73339800	1.26944700	0.80510600
N	-3.28303200	0.33490600	-0.39776000
H	-3.83355000	1.17304000	-0.60753500
C	-3.88267700	-0.35373300	0.74495100
H	-3.40282100	-1.33778700	0.86372700
H	-4.95367000	-0.52240200	0.54365500
H	-3.79187600	0.19817500	1.71129400

M06/6-31G* with Solvent Correction

SCF Done: E(RM06) = -572.886356839

Zero-point correction=   0.223855
Thermal correction to Gibbs Free Energy=   0.185347

C	1.04131000	1.07336600	-0.00953800
C	3.33423600	-0.09163100	-0.10856900
C	1.25582800	-1.43762200	-0.12682900
C	2.62216200	-1.28252100	0.49905600
N	0.45396200	-0.19162400	-0.09483200
C	2.53327500	1.15121300	0.22688100
H	3.40499300	-0.21942700	-1.19964000
H	1.34974100	-1.75813000	-1.17488300
H	2.52660200	-1.14514500	1.58737100
H	2.64391700	1.38455300	1.29831500
H	4.35922000	0.00733100	0.26886900
H	0.67001300	-2.20667200	0.38380500
H	3.17669600	-2.21590300	0.34040600
H	2.87677000	2.04251700	-0.31090600
O	0.39341500	2.10303900	-0.08236100
C	-0.92753100	-0.40716300	-0.32856600
O	-1.30015500	-1.53341300	-0.58858300
C	-1.89946200	0.74877200	-0.19362000
H	-1.65504800	1.49841100	-0.95441100
H	-1.70454400	1.24972500	0.77516200
N	-3.26527200	0.30930400	-0.35834300
H	-3.82999000	1.12683500	-0.57048600
C	-3.80011600	-0.34475500	0.82331200
H	-3.30581200	-1.31306000	0.96301500
H	-4.87110400	-0.53299600	0.68807300
H	-3.66657300	0.24323200	1.75232200

M06-2X/6-31G* with Solvent Correction

SCF Done: E(RM062X) = -573.007182078

Zero-point correction= 0.227470
Thermal correction to Gibbs Free Energy= 0.189035

C	1.03892400	1.07198600	-0.01289700
C	3.33331100	-0.09416400	-0.09405600
C	1.24222400	-1.44153500	-0.13877500
C	2.61379300	-1.29726100	0.49514900
N	0.44937100	-0.18947800	-0.10161700
C	2.53149800	1.15032700	0.25976200
H	3.40677900	-0.20309200	-1.18275000
H	1.33682600	-1.75508900	-1.18456100
H	2.51414700	-1.17536400	1.58056300
H	2.62109900	1.35342900	1.33520800
H	4.35118300	-0.00383200	0.29443200
H	0.65347200	-2.20329400	0.37189900
H	3.16524900	-2.22519500	0.31859700
H	2.88303700	2.04535700	-0.25827700
O	0.39365100	2.10042500	-0.10903900
C	-0.92897700	-0.38857300	-0.34926700
O	-1.30811200	-1.50702600	-0.62867700
C	-1.90543600	0.77348500	-0.19970500
H	-1.68588400	1.50794100	-0.97731400
H	-1.69523000	1.27561600	0.75888000
N	-3.27059100	0.30912100	-0.33476600
H	-3.85383000	1.12369300	-0.50461300
C	-3.75446300	-0.37632100	0.85802200
H	-3.22697800	-1.32708000	0.96541200
H	-4.81986600	-0.59100000	0.74569600
H	-3.61102500	0.20555200	1.78384200

D3(BJ)-PBE0/def2-TZVPP//BP86/SVP with Solvent Correction

SCF Done: E(RB-P86) = -572.840483535
SCF Done: E(RPBE-PBE) = -572.806944858

Zero-point correction= 0.215963
Thermal correction to Gibbs Free Energy= 0.176997

C	1.05224900	1.08380600	-0.04189300
C	3.38666800	-0.08131900	-0.01936200
C	1.28793000	-1.43549600	-0.20860300
C	2.63489100	-1.30949800	0.49409100
N	0.46413300	-0.19400000	-0.14148600
C	2.54632100	1.16352600	0.27506600
H	3.55849300	-0.17858600	-1.11428700
H	1.43626200	-1.69549600	-1.28070200
H	2.48528400	-1.23274300	1.59424400
H	2.58927700	1.39828400	1.36387700
H	4.38630700	0.01349400	0.45271500
H	0.67439700	-2.24760300	0.22305000
H	3.20574800	-2.24384400	0.31263200
H	2.91931400	2.07109700	-0.24075300
O	0.39735900	2.11991300	-0.14630000
C	-0.93573600	-0.41750200	-0.33315300
O	-1.32044200	-1.56029400	-0.55861500
C	-1.91880100	0.75015600	-0.19514800
H	-1.67503500	1.49655800	-0.97746700
H	-1.68786300	1.27834200	0.76788500
N	-3.29611600	0.31349900	-0.33248200
H	-3.86561500	1.13245200	-0.57225900
C	-3.85152900	-0.33818400	0.84995100
H	-3.35681300	-1.31859700	1.00796400
H	-4.93282200	-0.53282400	0.69666300
H	-3.74035300	0.25309000	1.79784900

18$_{RC}$

B3LYP/6-31G*

SCF Done: E(RB3LYP) = -573.246802049

Zero-point correction= 0.226019
Thermal correction to Gibbs Free Energy= 0.190308

C	-0.25949600	-0.63210900	0.33658800
C	1.97243500	-1.40418600	-0.60045000
C	1.78349400	0.82449100	0.59219000
C	2.50591000	0.03533100	-0.51000000
N	0.34094700	0.69987800	0.43294600
C	0.44275500	-1.43056700	-0.77888500
H	2.23248500	-1.95276400	0.31283700
H	2.06276100	0.44170300	1.58119200
H	2.35427700	0.54788400	-1.46972600
H	0.17232700	-0.97808100	-1.74230800
H	2.44843800	-1.93032000	-1.43673000
H	2.01469600	1.89161400	0.55302700
H	3.58480900	0.03333700	-0.31233400
H	0.07345600	-2.46106200	-0.77679900
O	-0.07516900	-1.37858800	1.52014700
H	-0.61895800	-0.93155100	2.19233100
N	-1.67316800	-0.30710100	0.16142200
C	-2.53325900	-1.32934500	-0.40573800
H	-2.34241900	-2.28208100	0.09640400
H	-2.40077300	-1.46846000	-1.49347800
H	-3.57925600	-1.05768400	-0.22665200
C	-1.79911400	1.03665800	-0.38323800
C	-0.46785300	1.69886300	-0.03723700
O	-0.17264000	2.87345200	-0.18114700
H	-1.92169500	1.05770500	-1.48206700
H	-2.64137400	1.58660900	0.05234300

B3LYP/6-31G* with Solvent Correction

SCF Done: E(RB3LYP) = -573.254811477

Zero-point correction= 0.225731
Thermal correction to Gibbs Free Energy= 0.190020

C	-0.25097800	-0.63404700	0.33892200
C	1.99082900	-1.38806600	-0.59197100
C	1.77629700	0.85086500	0.57741200
C	2.50768200	0.05805100	-0.51578600
N	0.33415200	0.71052000	0.41291900
C	0.46113400	-1.43551900	-0.76689800
H	2.26422900	-1.92592000	0.32389000
H	2.05341500	0.48007200	1.57103500
H	2.35078400	0.55771500	-1.48117400
H	0.18400800	-0.99850800	-1.73487400
H	2.46992200	-1.91399300	-1.42602900
H	2.00274000	1.91836200	0.53143400
H	3.58545900	0.07119200	-0.31496100
H	0.10400000	-2.47010500	-0.75336100
O	-0.04940500	-1.35418100	1.53730100
H	-0.60445100	-0.91138200	2.20395000
N	-1.66883500	-0.33149300	0.16566900
C	-2.51296900	-1.36578200	-0.41035200
H	-2.31898600	-2.31595100	0.09499300
H	-2.36494200	-1.50316900	-1.49505700
H	-3.56264400	-1.10298800	-0.24301500
C	-1.81481000	1.00916500	-0.38353200
C	-0.49500900	1.68920400	-0.04115900
O	-0.22442600	2.87770400	-0.17909700
H	-1.93678900	1.02358000	-1.48198600
H	-2.66636600	1.54649900	0.04960200

BP86/6-31G*

SCF Done: E(RB-P86) = -573.245245436

Zero-point correction= 0.218881
Thermal correction to Gibbs Free Energy= 0.182912

C	-0.26353900	-0.63761700	0.33942900
C	1.97116100	-1.41390300	-0.60775100
C	1.79259000	0.81515400	0.59977600
C	2.51061000	0.02787800	-0.51367400
N	0.34549600	0.69875300	0.44115800
C	0.43656800	-1.43314700	-0.78637000
H	2.22969400	-1.96952000	0.31293500
H	2.07280400	0.42002200	1.59428300
H	2.35300600	0.55034600	-1.47755200
H	0.16407400	-0.96604500	-1.75299800
H	2.45003300	-1.94551000	-1.45096900
H	2.02535100	1.89243300	0.56710000
H	3.59943500	0.02352800	-0.32011300
H	0.05824800	-2.47026800	-0.79280400
O	-0.08274200	-1.39829900	1.52554800
H	-0.64504100	-0.94027600	2.19113600
N	-1.68475200	-0.29944400	0.17322900
C	-2.54174500	-1.32110900	-0.41454500
H	-2.34730600	-2.28604500	0.08281600
H	-2.40314500	-1.44866300	-1.51324900
H	-3.59800100	-1.05287000	-0.23562900
C	-1.79935700	1.04695200	-0.38496400
C	-0.46075400	1.71056400	-0.03579600
O	-0.15356300	2.89349900	-0.17944100
H	-1.91702300	1.05985200	-1.49510600
H	-2.65010000	1.60762300	0.04395500

PBE0/6-31G*

SCF Done: E(RPBE-PBE) = -572.551735360

Zero-point correction= 0.219724
Thermal correction to Gibbs Free Energy= 0.183816

C	-0.26592700	-0.63560800	0.33861100
C	1.96013300	-1.41603100	-0.61038600
C	1.79101100	0.80360500	0.60377000
C	2.50425800	0.02021500	-0.51122500
N	0.34732000	0.69497900	0.44433400
C	0.42904000	-1.42788200	-0.78950100
H	2.21472100	-1.97472600	0.30865800
H	2.06909100	0.40197700	1.59555100
H	2.34704300	0.54691600	-1.47187100
H	0.15842500	-0.95523200	-1.75301600
H	2.43756600	-1.94698700	-1.45347100
H	2.02894500	1.87927200	0.57686000
H	3.59227200	0.01135600	-0.31936000
H	0.04562900	-2.46219600	-0.80046800
O	-0.08853900	-1.40058300	1.51876100
H	-0.64687600	-0.94126200	2.18519100
N	-1.68232200	-0.29122300	0.17231600
C	-2.54223100	-1.30809400	-0.41004800
H	-2.35022100	-2.27199200	0.08873400
H	-2.40613100	-1.43876900	-1.50785100
H	-3.59656700	-1.03645700	-0.23018800
C	-1.78946900	1.05124400	-0.38613600
C	-0.45174000	1.70879600	-0.03463800
O	-0.13869700	2.88864600	-0.17874400
H	-1.90255100	1.06365800	-1.49604500
H	-2.63889700	1.61616900	0.03790600

M06/6-31G* with Solvent Correction

SCF Done: E(RM06) = -572.895497003

Zero-point correction= 0.225202
Thermal correction to Gibbs Free Energy= 0.189782

C	-0.30024000	-0.62398900	0.35221700
C	1.85497900	-1.46632700	-0.64808400
C	1.82099400	0.68916200	0.63267100
C	2.45928500	-0.07085000	-0.52243900
N	0.37630200	0.66558700	0.49151300
C	0.33624100	-1.39990600	-0.79786800
H	2.10407800	-2.05793000	0.24556200
H	2.08658300	0.22960500	1.59519800
H	2.28489300	0.49328500	-1.45307900
H	0.07743600	-0.88038100	-1.73548600
H	2.28853100	-1.99322100	-1.50808400
H	2.12534300	1.74102800	0.65805300
H	3.54599400	-0.12691300	-0.37626200
H	-0.09970700	-2.40642100	-0.84386600
O	-0.15916400	-1.41033800	1.49687400
H	-0.65665900	-0.95113700	2.19395000
N	-1.68151000	-0.20609200	0.18447800
C	-2.58424300	-1.16493400	-0.40973200
H	-2.46840600	-2.13456200	0.08836100
H	-2.42531800	-1.30098400	-1.49608000
H	-3.61760500	-0.82949100	-0.25978800
C	-1.69855000	1.12072900	-0.39183500
C	-0.34892600	1.69569800	-0.02173800
O	0.02834600	2.84440600	-0.18314900
H	-1.77592800	1.11313500	-1.49853900
H	-2.52346800	1.73748000	-0.01082900

M06-2X/6-31G* with Solvent Correction

SCF Done: E(RM062X) = -573.018235008

Zero-point correction= 0.228471
Thermal correction to Gibbs Free Energy= 0.192997

C	-0.28988700	-0.62054400	0.35044900
C	1.87551300	-1.45392700	-0.63693200
C	1.81115500	0.72533500	0.62264500
C	2.46877100	-0.04572600	-0.52342300
N	0.36643000	0.67944700	0.47356000
C	0.35098100	-1.40330300	-0.79969300
H	2.11864700	-2.02766300	0.26483900
H	2.07456800	0.27889900	1.58746700
H	2.29900300	0.50265300	-1.45878900
H	0.09164800	-0.89509100	-1.73794100
H	2.31802400	-1.98221300	-1.48687700
H	2.10077900	1.77787900	0.63234900
H	3.55000900	-0.09443000	-0.36083600
H	-0.07268300	-2.41150900	-0.83150800
O	-0.11970300	-1.39266100	1.50335500
H	-0.61763300	-0.93693200	2.20301900
N	-1.68447600	-0.23027200	0.19842200
C	-2.55791000	-1.21883800	-0.40507700
H	-2.41912000	-2.17692100	0.10220600
H	-2.37748600	-1.35474000	-1.48335900
H	-3.59646900	-0.90653500	-0.26791600
C	-1.72951100	1.09490300	-0.39391000
C	-0.38042700	1.69506100	-0.02868500
O	-0.02688800	2.85147100	-0.18800400
H	-1.80646900	1.07637100	-1.49525100
H	-2.55588700	1.69776900	-0.00633100

D3(BJ)-PBE0/def2-TZVPP//BP86/SVP with Solvent Correction

SCF Done: E(RB-P86) = -572.846490275
SCF Done: E(RPBE-PBE) = -572.815768599

Zero-point correction= 0.217113
Thermal correction to Gibbs Free Energy= 0.181088

C	-0.23732500	-0.64145200	0.33389800
C	2.02996600	-1.36757400	-0.57274800
C	1.76315700	0.88857700	0.56407500
C	2.52285700	0.09014300	-0.51056300
N	0.32614100	0.71974500	0.38703400
C	0.50135700	-1.44298300	-0.76223600
H	2.30023600	-1.89336100	0.36851900
H	2.04396200	0.53581100	1.58070300
H	2.36854500	0.57964100	-1.49806100
H	0.21808700	-1.01252700	-1.74752700
H	2.53806300	-1.90939100	-1.39835700
H	1.97011300	1.97582500	0.50413500
H	3.61230900	0.12745400	-0.29827600
H	0.15464600	-2.49621000	-0.74343500
O	-0.03068300	-1.34544400	1.54493900
H	-0.61904100	-0.90956000	2.19787800
N	-1.65976700	-0.36424000	0.13887300
C	-2.49822500	-1.41852100	-0.40253000
H	-2.27185300	-2.37360900	0.11242100
H	-2.38057000	-1.57210500	-1.50557600
H	-3.56506800	-1.18070500	-0.20908500
C	-1.84665400	0.98197300	-0.37770000
C	-0.53479000	1.69556300	-0.04416900
O	-0.28588700	2.89483600	-0.17001700
H	-1.98827500	1.02370500	-1.49045800
H	-2.71527400	1.50700000	0.07823600

18$_{RE}$

B3LYP/6-31G*

SCF Done: E(RB3LYP) = -573.247628404

Zero-point correction= 0.226137
Thermal correction to Gibbs Free Energy= 0.188625

C	1.24372100	0.95378400	-0.22513200
C	-1.18812900	1.86690800	-0.25764600
C	-2.37399600	0.91910300	0.02918600
N	1.65299600	-0.36171300	-0.06857600
C	0.00309800	1.26640300	-1.06954000
H	-0.79973300	2.30949800	0.66873300
H	-0.33535700	0.42664400	-1.68239700
H	-1.58704100	2.70557000	-0.83885300
H	-2.64937900	0.40327900	-0.89801800
H	0.36086600	2.03389700	-1.76423300
O	1.85706900	1.87893700	0.29859300
H	-3.24507200	1.52270300	0.31706500
C	-2.15657400	-0.13457000	1.13184400
H	-2.15713500	0.33231000	2.12236000
H	-2.96454700	-0.87095700	1.11082500
N	-0.87503700	-0.83074400	0.99415300
C	0.89922700	-1.51381000	-0.55771500
C	-0.58809500	-1.51187700	-0.15610600
O	-1.42788000	-2.05283600	-0.86099600
H	-0.08645300	-0.36017800	1.41808300
C	2.92063600	-0.62576100	0.60948300
H	2.76700100	-1.25634900	1.49440200
H	3.34481200	0.32893100	0.91682400
H	3.61917200	-1.13710000	-0.06440200
H	0.92136400	-1.60496400	-1.64983300
H	1.37295000	-2.41596600	-0.15662800

B3LYP/6-31G* with Solvent Correction

SCF Done: E(RB3LYP) = -573.258312813

Zero-point correction= 0.225923
Thermal correction to Gibbs Free Energy= 0.188252

C	1.26720900	0.94217100	-0.23185100
C	-1.15796200	1.88014500	-0.22795200
C	-2.36598300	0.95377400	0.03357100
N	1.63987000	-0.37281800	-0.04697400
C	0.01967600	1.27207300	-1.05701300
H	-0.76590400	2.28525700	0.71364000
H	-0.33292400	0.43791800	-1.66863500
H	-1.53539300	2.74307700	-0.78670700
H	-2.64597300	0.46301700	-0.90602700
H	0.37313900	2.03643600	-1.75688500
O	1.91682600	1.86855400	0.25852900
H	-3.22516000	1.57041000	0.32727700
C	-2.17684400	-0.12007800	1.12130800
H	-2.17326500	0.33298900	2.11696500
H	-2.99931700	-0.83989300	1.08755100
N	-0.90364300	-0.83314300	0.97995200
C	0.88050700	-1.51476600	-0.55519400
C	-0.60704100	-1.50617600	-0.16540300
O	-1.44594700	-2.04340900	-0.88379300
H	-0.11447200	-0.39465100	1.43678900
C	2.90243200	-0.66773900	0.63282700
H	2.72965300	-1.31797000	1.49768500
H	3.33885500	0.27136600	0.96711800
H	3.59707700	-1.17058300	-0.05022100
H	0.91597100	-1.58933100	-1.64765000
H	1.34512900	-2.42390500	-0.16139700

BP86/6-31G*

SCF Done: E(RB-P86) = -573.246278842

Zero-point correction= 0.219035
Thermal correction to Gibbs Free Energy= 0.181363

C	1.24595600	0.95590200	-0.22911000
C	-1.19111300	1.87586200	-0.27172100
C	-2.37871000	0.92788200	0.02860200
N	1.65957700	-0.36912400	-0.07380700
C	0.00096800	1.25984200	-1.07889600
H	-0.79565800	2.33400800	0.65547500
H	-0.34544900	0.40445800	-1.68383300
H	-1.59401800	2.71509200	-0.86664900
H	-2.65540900	0.39597600	-0.90039600
H	0.36242100	2.02146500	-1.79307700
O	1.86506300	1.88919500	0.30075200
H	-3.25927600	1.53593700	0.31328000
C	-2.15436100	-0.11923400	1.14257100
H	-2.15125000	0.35618700	2.13922900
H	-2.96438200	-0.86748100	1.12885600
N	-0.86758100	-0.81452700	0.99869200
C	0.89872400	-1.52367000	-0.56282500
C	-0.59462400	-1.51977300	-0.15040200
O	-1.44751000	-2.08307400	-0.84164100
H	-0.06451000	-0.31794100	1.38973700
C	2.92864700	-0.62896000	0.61191200
H	2.77521300	-1.27561500	1.49627900
H	3.33569700	0.34038800	0.93275800
H	3.64551600	-1.12633100	-0.06789700
H	0.91339300	-1.61153200	-1.66518100
H	1.38039100	-2.43514200	-0.16645100

PBE0/6-31G*

SCF Done: E(RPBE-PBE) = -572.551773662

Zero-point correction= 0.219823
Thermal correction to Gibbs Free Energy= 0.182205

C	1.23566100	0.95690600	-0.22873100
C	-1.19866100	1.86593000	-0.28053600
C	-2.37514400	0.91268000	0.02950800
N	1.66148000	-0.36311500	-0.08524100
C	-0.00464600	1.25631200	-1.08191700
H	-0.80509500	2.33503500	0.64114200
H	-0.34544600	0.40247700	-1.69092700
H	-1.60980600	2.69593100	-0.88100700
H	-2.65021100	0.37233600	-0.89420400
H	0.35746300	2.02050400	-1.79181100
O	1.84162000	1.89053800	0.31259200
H	-3.25897500	1.51484600	0.31232200
C	-2.13986200	-0.12354500	1.14714200
H	-2.13394800	0.35853700	2.13963100
H	-2.94685600	-0.87428500	1.14282800
N	-0.85426900	-0.81252800	1.00031000
C	0.90343700	-1.51766200	-0.56703400
C	-0.58507800	-1.51765600	-0.14740900
O	-1.44004300	-2.08008500	-0.83447000
H	-0.05291300	-0.31526400	1.39070000
C	2.92219200	-0.61743500	0.60921400
H	2.76386500	-1.25332600	1.49977400
H	3.32962100	0.35363400	0.92237100
H	3.64120000	-1.12368000	-0.06015600
H	0.91096800	-1.60689500	-1.66862900
H	1.38965200	-2.42715600	-0.17390600

M06/6-31G* with Solvent Correction

SCF Done: E(RM06) = -572.894651638

Zero-point correction= 0.225055
Thermal correction to Gibbs Free Energy= 0.187554

C	1.24558600	0.94353800	-0.24107500
C	-1.15715400	1.84617300	-0.22823700
C	-2.35225400	0.92175700	0.02737000
N	1.64301700	-0.36212400	-0.07548000
C	0.00402100	1.24764500	-1.06204300
H	-0.75703300	2.23347500	0.72209200
H	-0.34609100	0.40268200	-1.66741600
H	-1.53386100	2.72542600	-0.76536000
H	-2.62300500	0.41715600	-0.91137500
H	0.34812300	2.00762600	-1.77541300
O	1.86564000	1.87245600	0.26584100
H	-3.22316900	1.53124100	0.30728600
C	-2.15034300	-0.12518600	1.12070700
H	-2.15248600	0.34377800	2.11193800
H	-2.96299800	-0.86084400	1.10355200
N	-0.87246000	-0.81619900	0.98473600
C	0.88509000	-1.50430400	-0.55768700
C	-0.58599200	-1.49085200	-0.15845100
O	-1.43100800	-2.01935700	-0.86622000
H	-0.08615400	-0.37943800	1.45126700
C	2.87929900	-0.64585700	0.63569500
H	2.68038500	-1.23047600	1.54407500
H	3.34501100	0.30072900	0.91337700
H	3.56489200	-1.21629400	-0.00366900
H	0.91053500	-1.59739500	-1.65228900
H	1.35538300	-2.41167500	-0.15749600

M06-2X/6-31G* with Solvent Correction

SCF Done: E(RM062X) = -573.015725115

Zero-point correction= 0.228520
Thermal correction to Gibbs Free Energy= 0.191129

C	1.21678900	0.95032300	-0.23790100
C	-1.20422900	1.82132800	-0.25294900
C	-2.37603100	0.86446700	0.02391300
N	1.66568700	-0.33812700	-0.10253600
C	-0.02636200	1.23077600	-1.07720100
H	-0.81221400	2.23547600	0.68436200
H	-0.35861900	0.37581700	-1.67143300
H	-1.60413700	2.67122900	-0.81260500
H	-2.62614900	0.32961300	-0.89901200
H	0.31349200	1.98887500	-1.78924600
O	1.79415800	1.88964400	0.29935700
H	-3.26049100	1.45070200	0.29665000
C	-2.13008800	-0.15586700	1.14154700
H	-2.12870800	0.33383500	2.11833700
H	-2.91668600	-0.91423300	1.14611800
N	-0.83259200	-0.80987400	0.99366100
C	0.92023400	-1.49660400	-0.56861200
C	-0.55231600	-1.48399200	-0.14942600
O	-1.40467200	-2.01221700	-0.84785200
H	-0.05252900	-0.36033200	1.45502900
C	2.90407300	-0.58985200	0.62403400
H	2.70251600	-1.08393300	1.58109900
H	3.39560400	0.36248500	0.80886300
H	3.55815700	-1.23142700	0.02727800
H	0.93190000	-1.59459800	-1.65882400
H	1.39789800	-2.39039200	-0.15695300

D3(BJ)-PBE0/def2-TZVPP//BP86/SVP with Solvent Correction

SCF Done: E(RB-P86) = -572.847799770
SCF Done: E(RPBE-PBE) = -572.817982749

Zero-point correction= 0.217120
Thermal correction to Gibbs Free Energy= 0.179585

C	1.24181600	0.95809000	-0.22775700
C	-1.20787100	1.86577400	-0.27592600
C	-2.38785700	0.91879700	0.04201200
N	1.65942900	-0.35768200	-0.06423300
C	-0.00745300	1.25661100	-1.07076800
H	-0.82039400	2.34778900	0.64930600
H	-0.34427200	0.40239700	-1.69105200
H	-1.62010800	2.69580700	-0.88602600
H	-2.68463100	0.39300800	-0.89130300
H	0.35429900	2.02616500	-1.78442600
O	1.86147900	1.89926400	0.28642300
H	-3.26727300	1.52930000	0.34173300
C	-2.15053800	-0.13106000	1.14822500
H	-2.13598400	0.34498900	2.14980500
H	-2.97487400	-0.87388700	1.14949600
N	-0.86833200	-0.82009200	0.98207200
C	0.90965300	-1.51197200	-0.56038200
C	-0.58113200	-1.52157900	-0.15668800
O	-1.42777000	-2.08309600	-0.85683500
H	-0.06436600	-0.37708500	1.43529100
C	2.92692400	-0.61874800	0.61961600
H	2.77483500	-1.26680200	1.50899300
H	3.34510500	0.35096300	0.94372500
H	3.64703300	-1.12119700	-0.06160200
H	0.92945600	-1.60156900	-1.66851400
H	1.39257200	-2.43028800	-0.16699400

Isomers 19_{RO}/19_{RC}/19_{RE}

Spartan Calculations

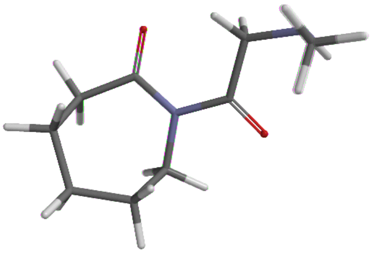

19_{RO}
ΔG ° = 0.0 kcal/mol

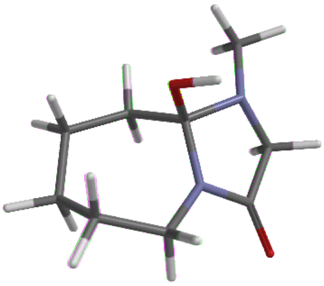

19_{RC}
ΔG ° = 6.37 kcal/mol

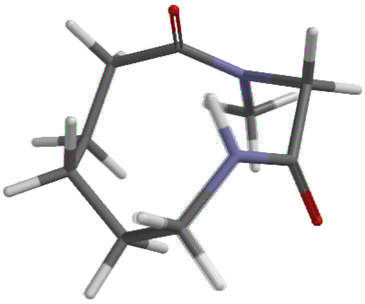

19_{RE}
ΔG ° = 0.74 kcal/mol

19_{RO}	Calculated energy (au)	Calculated energy (kcal/mol)	Relative energy (kcal/mol)
M0002	-612.5522	-384376.5055	0.00
M0003	-612.5522	-384376.5055	0.00
M0006	-612.551561	-384376.1045	0.40
M0001	-612.550335	-384375.3352	1.17
M0004	-612.549429	-384374.7667	1.74
M0005	-612.549429	-384374.7667	1.74
M0022	-612.548932	-384374.4548	2.05
M0023	-612.548932	-384374.4548	2.05
M0021	-612.548813	-384374.3802	2.13
M0009	-612.548426	-384374.1373	2.37
M0008	-612.548369	-384374.1015	2.40
M0011	-612.548075	-384373.9171	2.59
M0024	-612.546729	-384373.0724	3.43
M0018	-612.546552	-384372.9614	3.54
M0019	-612.546552	-384372.9614	3.54
M0012	-612.546433	-384372.8867	3.62
M0016	-612.546149	-384372.7085	3.80
M0035	-612.546069	-384372.6583	3.85
M0036	-612.546069	-384372.6583	3.85
M0038	-612.546055	-384372.6495	3.86
M0013	-612.54602	-384372.6276	3.88
M0014	-612.54602	-384372.6276	3.88
M0007	-612.546004	-384372.6175	3.89
M0010	-612.545944	-384372.5799	3.93
M0027	-612.545763	-384372.4663	4.04
M0020	-612.54573	-384372.4456	4.06
M0032	-612.545515	-384372.3107	4.19
M0017	-612.54492	-384371.9367	4.57
M0041	-612.542964	-384370.7099	5.80
M0049	-612.542572	-384370.4639	6.04
M0048	-612.542265	-384370.2713	6.23

M0053	-612.542203	-384370.2324	6.27
M0054	-612.542203	-384370.2324	6.27
M0033	-612.542148	-384370.1979	6.31
M0037	-612.54195	-384370.0736	6.43
M0045	-612.541744	-384369.9444	6.56
M0015	-612.541684	-384369.9067	6.60
M0029	-612.540944	-384369.4424	7.06
M0030	-612.540944	-384369.4424	7.06
M0031	-612.539348	-384368.4409	8.06
M0025	-612.539202	-384368.3493	8.16
M0026	-612.539202	-384368.3493	8.16
M0028	-612.539198	-384368.3467	8.16
M0056	-612.537646	-384367.3729	9.13
M0052	-612.537306	-384367.1595	9.35
M0039	-612.537042	-384366.9939	9.51
M0046	-612.536639	-384366.741	9.76
M0042	-612.536362	-384366.5672	9.94
M0043	-612.536362	-384366.5672	9.94
M0047	-612.536329	-384366.5464	9.96
M0044	-612.535627	-384366.1059	10.40
M0040	-612.533669	-384364.8773	11.63
M0034	-612.532696	-384364.2667	12.24
M0057	-612.53269	-384364.263	12.24
M0050	-612.532381	-384364.0691	12.44
M0051	-612.532381	-384364.0691	12.44
M0058	-612.531633	-384363.5997	12.91
M0055	-612.530589	-384362.9446	13.56
**19_{RC}**	**Calculated energy (au)**	**Calculated energy (kcal/mol)**	**Relative energy (kcal/mol)**
M0002	-612.118922	-384104.6236	0.00
M0017	-612.117102	-384103.4815	1.14
M0001	-612.115837	-384102.6877	1.94
M0016	-612.115712	-384102.6093	2.01
M0004	-612.115308	-384102.3558	2.27
M0003	-612.115282	-384102.3395	2.28
M0007	-612.114457	-384101.8218	2.80
M0018	-612.11358	-384101.2715	3.35
M0009	-612.112972	-384100.8899	3.73
M0005	-612.112715	-384100.7287	3.89
M0024	-612.112208	-384100.4105	4.21
M0006	-612.111301	-384099.8414	4.78
M0021	-612.110886	-384099.581	5.04
M0013	-612.110293	-384099.2089	5.41
M0015	-612.11007	-384099.0689	5.55
M0010	-612.10946	-384098.6862	5.94
M0008	-612.109149	-384098.491	6.13
M0020	-612.108977	-384098.3831	6.24

M0012	-612.107926	-384097.7236	6.90
M0014	-612.107396	-384097.391	7.23
M0019	-612.106404	-384096.7685	7.86
M0011	-612.105915	-384096.4617	8.16
M0022	-612.104419	-384095.5229	9.10
M0027	-612.102893	-384094.5654	10.06
M0023	-612.101606	-384093.7578	10.87
M0025	-612.101308	-384093.5708	11.05
M0026	-612.099772	-384092.6069	12.02
**19_{RE}**	**Calculated energy (au)**	**Calculated energy (kcal/mol)**	**Relative energy (kcal/mol)**
M0001	-612.558562	-384380.4977	0.00
M0003	-612.556865	-384379.4328	1.06
M0002	-612.556396	-384379.1385	1.36
M0006	-612.55463	-384378.0303	2.47
M0005	-612.554544	-384377.9764	2.52
M0004	-612.553025	-384377.0232	3.47
M0007	-612.552199	-384376.5049	3.99
M0009	-612.548827	-384374.3889	6.11
M0010	-612.547437	-384373.5167	6.98
M0008	-612.547388	-384373.486	7.01
M0011	-612.545201	-384372.1136	8.38

Table S6. Energies calculated at DFT/B3LYP/6-31G* in vacuum for the conformers found for **19_{RO}/19_{RC}/19_{RE}**.

Isomer	ΔG° (au)	ΔG° (kcal/mol)	ΔG° (kcal/mol)
**19_{RO}**	-612.346843	-384247.64	0.00
**19_{RC}**	-612.336689	-384241.27	6.37
**19_{RE}**	-612.345663	-384246.90	0.74

Table S7. Relative free energies (ΔG°) of the lowest energy geometries of **19_{RO}/19_{RC}/19_{RE}** at DFT/B3LYP/6-31G* in vacuum) in kcal/mol.

XYZ Coordinates

19_{RO}

H	-2.39961700	2.35774600	0.44505900
C	-2.21797000	1.33681800	0.08427100
H	-2.88191000	1.18857400	-0.77321000
C	-2.51825900	0.37817700	1.23305000
H	-3.38129500	0.76709900	1.78761500
H	-1.67859000	0.34232700	1.93775800
C	-2.86707000	-1.03336100	0.76799900
C	-1.71771300	-1.77492500	0.08985600
H	-2.09884800	-2.74690200	-0.24640800
H	-0.92588900	-1.98017500	0.82054900
C	-1.14898300	-1.03008600	-1.11492500
H	-0.61242600	-1.74472000	-1.74886400
H	-1.95438700	-0.62225000	-1.73643200
N	-0.24944000	0.05123800	-0.71488600
C	-0.76645000	1.29151900	-0.36135700
O	-0.13028800	2.34159900	-0.45120800
C	1.11870100	-0.25290100	-0.67865500
O	1.55537500	-1.33855200	-1.08331100
C	2.08548100	0.80237900	-0.10005700
H	2.25565400	1.55718700	-0.87435900
H	1.66683900	1.28163000	0.78970800
N	3.39578300	0.19590700	0.23632800
H	3.61810700	-0.46938100	-0.51094100
C	3.34101000	-0.53325700	1.49758500
H	2.60481100	-1.34279700	1.47631400
H	3.10791900	0.13855000	2.32998400
H	4.32020600	-0.97797200	1.70076100
H	-3.19277400	-1.61933700	1.63614700
H	-3.72537300	-0.99016000	0.08577500

19_{RC}

H	3.37732200	1.19950300	0.66339700
C	2.50193600	0.54813000	0.54656700
H	2.53863000	-0.15110400	1.39010800
C	2.62538100	-0.21219100	-0.76967100
C	1.84137000	-1.52231800	-0.77117900
C	0.31671800	-1.37961000	-0.82007700
H	-0.06731800	-2.40720200	-0.79797100
H	0.03219200	-0.95326800	-1.79091100
C	-0.31803800	-0.56641700	0.32558100
C	1.25954100	1.42530200	0.63760700
O	0.10716600	-1.03442800	1.57476700
H	-0.58517300	-0.68368500	2.15849300
N	0.01028000	0.82438900	0.22064400
N	-1.77462300	-0.54044000	0.35197200
H	2.14989000	-2.10261500	-1.65026800
H	2.12639100	-2.12411900	0.10081900
H	1.12541200	1.75581900	1.67454500
H	1.41317800	2.33486600	0.04424900
C	-1.04220000	1.57000300	-0.28197100
O	-1.05106400	2.76108000	-0.54423100
C	-2.18938800	0.60722900	-0.46317900
C	-2.46632400	-1.76251900	-0.00817200
H	-3.54104700	-1.65235200	0.17528200
H	-2.12788600	-2.59933200	0.61232600
H	-2.33777700	-2.03298100	-1.06187700
H	-3.11510600	1.04522000	-0.08127400
H	-2.27748300	0.36945900	-1.52883000
H	2.33263100	0.41763800	-1.61775100
H	3.68375700	-0.46236800	-0.91596500

19$_{RE}$

H	-0.67588900	1.46886900	-1.52714600
C	-0.60630600	2.13115100	-0.65713900
H	-0.85408300	3.13606600	-1.02255000
C	0.83297300	2.16579600	-0.10092400
C	1.80540200	1.19714100	-0.80751300
C	2.34848300	0.06820800	0.07849300
H	2.95831400	0.50103300	0.88190800
H	3.02439700	-0.58174200	-0.48465600
C	-1.67929800	1.74332300	0.37181500
N	-1.49497800	0.40156700	0.87459600
N	0.50255400	-1.62235500	-0.04605900
H	1.35741300	0.78582400	-1.71647100
H	2.67252800	1.77840800	-1.14801500
H	-1.65532200	2.42332800	1.22938000
H	-2.67025200	1.80868800	-0.09068300
C	-1.71924600	-0.69841800	0.06169700
O	-2.41662100	-0.67255600	-0.94875200
C	-0.85605000	-1.90919500	0.43968100
C	0.75297900	-1.90636000	-1.45174000
H	0.42266900	-2.92552300	-1.67326100
H	1.81516000	-1.83331000	-1.68967800
H	0.19352800	-1.20018700	-2.07081100
H	-0.79378300	0.28089500	1.60658200
C	1.25328600	-0.75057200	0.72929700
O	0.96770800	-0.53889000	1.91320000
H	-0.86716000	-2.04976800	1.52547900
H	-1.24678500	-2.81594900	-0.03163800
H	1.21442800	3.18176900	-0.27178900
H	0.84977500	2.03223100	0.98600500

Gaussian Calculations

19_{RO}

B3LYP/6-31G*

SCF Done: E(RB3LYP) = -612.559565227

Zero-point correction= 0.254366
Thermal correction to Gibbs Free Energy= 0.214224

H	-2.46570600	2.41366400	-0.03215700
C	-2.27951200	1.34851200	-0.18707400
H	-2.77365100	1.06818600	-1.12719400
C	-2.87360600	0.52986600	0.98161300
H	-3.82990700	0.98985800	1.25852000
H	-2.22271500	0.62855700	1.86104400
C	-3.11785000	-0.95050000	0.66356800
C	-1.86532100	-1.74206600	0.26894400
H	-2.16216300	-2.76755900	0.01355300
H	-1.17406300	-1.82304200	1.11797400
C	-1.10005600	-1.16128100	-0.92861700
H	-0.43541200	-1.91581600	-1.34490700
H	-1.79037800	-0.85285600	-1.72109900
N	-0.22175300	-0.01853500	-0.58780400
C	-0.76419900	1.25078800	-0.33033300
O	-0.06956200	2.24559600	-0.19947800
C	1.17248800	-0.29784100	-0.55071900
O	1.56407700	-1.41687000	-0.84365400
C	2.19254900	0.78783900	-0.15630700
H	2.20745800	1.53762500	-0.95602800
H	1.85697600	1.32748300	0.73205100
N	3.52428000	0.25909100	0.06310400
H	3.74786400	-0.37665800	-0.70003900
C	3.67133700	-0.46190700	1.32701900
H	2.97945600	-1.31116300	1.45401500
H	3.51681600	0.23588100	2.15888400
H	4.69501600	-0.84320000	1.40233500
H	-3.59069500	-1.43055400	1.52987600
H	-3.84768400	-1.01456900	-0.15743400

B3LYP/6-31G* with Solvent Correction

SCF Done: E(RB3LYP) = -612.567683397

Zero-point correction= 0.254059
Thermal correction to Gibbs Free Energy= 0.213834

H	-2.46457600	2.41055400	0.02341600
C	-2.27424400	1.34950700	-0.15298600
H	-2.78733800	1.08318400	-1.08637200
C	-2.83814700	0.50864800	1.01598400
H	-3.78677200	0.96538800	1.32126500
H	-2.16638800	0.59077000	1.88109300
C	-3.09192100	-0.96498800	0.67627000
C	-1.84871700	-1.75034100	0.24272700
H	-2.15187900	-2.76856000	-0.03192200
H	-1.14266400	-1.85006000	1.07745600
C	-1.10789400	-1.14371600	-0.95679800
H	-0.45435300	-1.88761000	-1.40681800
H	-1.81107000	-0.81311600	-1.72706300
N	-0.22194200	-0.00616000	-0.60490000
C	-0.76565000	1.25936900	-0.33508500
O	-0.06795300	2.25854600	-0.22558600
C	1.16544700	-0.28487700	-0.56738200
O	1.56451700	-1.39953100	-0.87765900
C	2.18249100	0.79161700	-0.14307700
H	2.21399900	1.54747500	-0.93598500
H	1.83379200	1.31704600	0.74865600
N	3.51292200	0.25831800	0.09251300
H	3.74587500	-0.36592800	-0.67787200
C	3.63000900	-0.49188100	1.34571300
H	2.92914600	-1.33717200	1.43763200
H	3.45915700	0.18695400	2.18960000
H	4.64874800	-0.88337400	1.43337800
H	-3.54551800	-1.45918000	1.54453600
H	-3.83777300	-1.01361900	-0.13053000

BP86/6-31G*

SCF Done: E(RB-P86) = -612.554288530

Zero-point correction= 0.246394
Thermal correction to Gibbs Free Energy= 0.206029

H	-2.48095000	2.42482800	-0.09795300
C	-2.28902900	1.34738600	-0.22453200
H	-2.76287500	1.04216500	-1.17881500
C	-2.91798500	0.55114100	0.94797300
H	-3.88903400	1.02096800	1.19188400
H	-2.28570000	0.66880200	1.84975800
C	-3.15347000	-0.93917200	0.65030400
C	-1.88565800	-1.73626600	0.30131200
H	-2.17300300	-2.77918400	0.06682000
H	-1.20489000	-1.79074000	1.17254800
C	-1.09689600	-1.18413600	-0.90057100
H	-0.41139700	-1.95232700	-1.28626500
H	-1.77762800	-0.89398100	-1.72142300
N	-0.21929100	-0.03103400	-0.57390200
C	-0.76508200	1.24824500	-0.32472900
O	-0.06191300	2.24833600	-0.17166400
C	1.18515700	-0.31014200	-0.54016800
O	1.58028700	-1.44223200	-0.82853300
C	2.20687300	0.78799000	-0.16690500
H	2.20530000	1.53884800	-0.97979600
H	1.87552000	1.34150700	0.72667100
N	3.55110600	0.26694800	0.04032900
H	3.74685900	-0.40191000	-0.71609500
C	3.70233400	-0.43998600	1.31990800
H	2.99842300	-1.28849100	1.47130700
H	3.56073300	0.27956300	2.14735100
H	4.73176100	-0.83177200	1.39278900
H	-3.65369500	-1.40911700	1.51845900
H	-3.86659000	-1.01975600	-0.19620300

PBE0/6-31G*

SCF Done: E(RPBE-PBE) = -611.804824417

Zero-point correction= 0.247334

Thermal correction to Gibbs Free Energy= 0.207039

H	-2.47938500	2.41972500	-0.07876400
C	-2.28628200	1.34432500	-0.21341200
H	-2.76721400	1.04448800	-1.16482300
C	-2.90160000	0.54072900	0.95671100
H	-3.87042600	1.00623700	1.21286600
H	-2.26171200	0.65374800	1.85268300
C	-3.13617100	-0.94527700	0.65279600
C	-1.87232400	-1.73669100	0.29084500
H	-2.15894500	-2.77766500	0.05190200
H	-1.18564100	-1.79548500	1.15612600
C	-1.09405300	-1.17522000	-0.90964800
H	-0.41044700	-1.93940600	-1.30508200
H	-1.78046500	-0.88163200	-1.72344200
N	-0.21982700	-0.02568300	-0.58062700
C	-0.76577000	1.25008500	-0.32614700
O	-0.06460400	2.25036700	-0.17619500
C	1.18126400	-0.30450100	-0.54301700
O	1.57764300	-1.43486000	-0.83170400
C	2.19979200	0.78951000	-0.16208300
H	2.20131200	1.54441900	-0.97027900
H	1.86514500	1.33958300	0.73188000
N	3.53946000	0.26763400	0.04877800
H	3.73915400	-0.39407600	-0.71127000
C	3.68066800	-0.44917800	1.31969500
H	2.97622300	-1.29847000	1.45807400
H	3.53267600	0.26190400	2.15223200
H	4.70858300	-0.84141100	1.39775400
H	-3.62962200	-1.42061400	1.52056400
H	-3.85412500	-1.02175300	-0.18873400

M06/6-31G* with Solvent Correction

SCF Done: E(RM06) = -612.168270073

Zero-point correction= 0.252906
Thermal correction to Gibbs Free Energy= 0.212717

H	-2.42924500	2.40803600	0.19090000
C	-2.24108200	1.35653400	-0.05053400
H	-2.83325200	1.12941900	-0.94972000
C	-2.67787800	0.45179000	1.11040700
H	-3.57973800	0.89264900	1.55515800
H	-1.90931100	0.47320000	1.89963400
C	-2.98074400	-0.98738700	0.71441400
C	-1.79076900	-1.75519200	0.15731000
H	-2.11921200	-2.75801700	-0.14939300
H	-1.02335300	-1.90350100	0.93362700
C	-1.13686600	-1.07710500	-1.03693900
H	-0.51512900	-1.78663900	-1.58571700
H	-1.88780400	-0.69298800	-1.73831500
N	-0.23840000	0.03015400	-0.65977700
C	-0.76279700	1.29334300	-0.35526200
O	-0.06474500	2.28822500	-0.29395500
C	1.13109000	-0.28796900	-0.58233900
O	1.50011800	-1.41841500	-0.84734800
C	2.15154900	0.76896400	-0.17209100
H	2.21031400	1.50854000	-0.98268500
H	1.79773700	1.33062100	0.70051300
N	3.45392000	0.21232200	0.10686800
H	3.68327500	-0.44868400	-0.63309800
C	3.50448500	-0.48447600	1.38326800
H	2.76002900	-1.29320200	1.49070400
H	3.34083700	0.23682900	2.19542100
H	4.50054100	-0.91998400	1.52309000
H	-3.38633100	-1.52296100	1.58400000
H	-3.78289800	-0.98013700	-0.04273500

M06-2X/6-31G* with Solvent Correction

SCF Done: E(RM062X) = -612.301830051

Zero-point correction= 0.257146
Thermal correction to Gibbs Free Energy= 0.217496

H	-2.44631000	2.39096500	0.22252900
C	-2.25130600	1.34829100	-0.03362300
H	-2.84192100	1.11826400	-0.92835500
C	-2.66239400	0.42132400	1.12709700
H	-3.56422800	0.84248200	1.58185400
H	-1.88572100	0.44242200	1.90236000
C	-2.95437400	-1.02176100	0.71315100
C	-1.75752800	-1.77242000	0.13117700
H	-2.08174000	-2.76756200	-0.19178500
H	-0.98388500	-1.92219900	0.89458800
C	-1.12351400	-1.06202600	-1.06396400
H	-0.50287000	-1.75144700	-1.63179800
H	-1.88848000	-0.67222100	-1.74078900
N	-0.23548600	0.04794900	-0.67157300
C	-0.76867400	1.30018800	-0.34733700
O	-0.07654600	2.29724000	-0.27496800
C	1.13588100	-0.24754500	-0.60395400
O	1.52690300	-1.35765200	-0.91565100
C	2.14507700	0.80592500	-0.12549600
H	2.22440700	1.57164100	-0.90328600
H	1.76686200	1.31774600	0.76256600
N	3.44240400	0.23190700	0.15985200
H	3.69507300	-0.37428900	-0.61823600
C	3.43733900	-0.56926800	1.38138600
H	2.68302600	-1.36987000	1.38608500
H	3.24845900	0.08760400	2.23667000
H	4.42198200	-1.02298000	1.51816400
H	-3.33886000	-1.57046700	1.57956500
H	-3.76011700	-1.01203700	-0.03375500

D3(BJ)-PBE0/def2-TZVPP//BP86/SVP with Solvent Correction

SCF Done: E(RB-P86) = -612.123939622
SCF Done: E(RPBE-PBE) = -612.080813674

Zero-point correction= 0.244341
Thermal correction to Gibbs Free Energy= 0.204127

H	-2.47729000	2.42866200	-0.09402300
C	-2.28597300	1.34607800	-0.22185700
H	-2.74972000	1.04880600	-1.18894200
C	-2.93370300	0.55119100	0.93809800
H	-3.91073500	1.02772500	1.16563200
H	-2.31621900	0.67252900	1.85618000
C	-3.16778100	-0.93670500	0.64172700
C	-1.90046000	-1.73269700	0.30005700
H	-2.18736700	-2.77853100	0.05654000
H	-1.22549500	-1.79692200	1.18217900
C	-1.09933800	-1.18105100	-0.89135100
H	-0.41500000	-1.95852800	-1.27519000
H	-1.77316700	-0.89009500	-1.72395200
N	-0.21819300	-0.03315900	-0.56282400
C	-0.76554000	1.24512100	-0.31751800
O	-0.06067500	2.24194100	-0.17071800
C	1.18038500	-0.31195100	-0.52862100
O	1.58059800	-1.43869500	-0.81638200
C	2.20173000	0.78326500	-0.15393000
H	2.17293900	1.54700600	-0.96236600
H	1.86494300	1.32966700	0.74975700
N	3.54799200	0.27542400	0.03326000
H	3.76850500	-0.35622000	-0.74866500
C	3.74897200	-0.44389500	1.29135700
H	3.07072200	-1.32012400	1.44888800
H	3.60326800	0.25077800	2.14693800
H	4.79352500	-0.81499300	1.34345400
H	-3.67611400	-1.40798400	1.51060800
H	-3.88053100	-1.01973500	-0.21106400

19$_{RC}$

B3LYP/6-31G*

SCF Done: E(RB3LYP) = -612.553308271

Zero-point correction= 0.255024
Thermal correction to Gibbs Free Energy= 0.217653

H	3.35327400	1.28756000	0.65587300
C	2.49673400	0.61187800	0.53493200
H	2.49499700	-0.04936000	1.40835000
C	2.67943300	-0.23302300	-0.73631000
C	1.88140100	-1.54557500	-0.72600300
C	0.35375800	-1.40987900	-0.79536300
H	-0.07724200	-2.41685900	-0.77485900
H	0.06222500	-0.95333000	-1.75053400
C	-0.31641200	-0.58454400	0.32154400
C	1.25310100	1.50430900	0.55430800
O	0.10274500	-1.13035100	1.55085400
H	-0.49659300	-0.74761800	2.21606300
N	-0.00837200	0.84922300	0.20266800
N	-1.78508600	-0.56001500	0.27240000
H	2.19510100	-2.15817600	-1.58169500
H	2.14499900	-2.11366300	0.17459200
H	1.15548700	1.96481200	1.54725200
H	1.36555200	2.32860400	-0.15774300
C	-1.06390700	1.59085800	-0.25255300
O	-1.06609700	2.79323700	-0.46412000
C	-2.22062900	0.62134300	-0.45383200
C	-2.48457500	-1.79737500	-0.02303400
H	-3.53800900	-1.68067200	0.25480300
H	-2.06535800	-2.60637900	0.58183800
H	-2.44651000	-2.09109500	-1.08665100
H	-3.15086400	1.03699700	-0.05078900
H	-2.37273800	0.44789900	-1.53587400
H	2.42084400	0.36238700	-1.62437700
H	3.74243600	-0.48661000	-0.83773500

B3LYP/6-31G* with Solvent Correction

SCF Done: E(RB3LYP) = -612.561214317

Zero-point correction= 0.254694
Thermal correction to Gibbs Free Energy= 0.217279

H	3.35777500	1.27860400	0.65016100
C	2.50060000	0.60385900	0.53187100
H	2.50108400	-0.05680900	1.40550800
C	2.67642000	-0.24022900	-0.74059800
C	1.87532100	-1.55064300	-0.72832200
C	0.34752600	-1.41213800	-0.79104100
H	-0.08607500	-2.41771200	-0.76789100
H	0.05359100	-0.95798200	-1.74619400
C	-0.32023500	-0.58597800	0.32572400
C	1.25986200	1.49991000	0.55687400
O	0.09933400	-1.12301200	1.55762000
H	-0.49483100	-0.73595800	2.22584200
N	-0.00622900	0.85178000	0.20236300
N	-1.78787400	-0.55469300	0.27657900
H	2.18230700	-2.16218000	-1.58678900
H	2.14498200	-2.12151800	0.16900700
H	1.16292500	1.95490400	1.55185000
H	1.37914700	2.32407600	-0.15383400
C	-1.05596800	1.58753100	-0.25304600
O	-1.05838400	2.79695700	-0.46595600
C	-2.21404000	0.62484400	-0.45905900
C	-2.49302400	-1.79022600	-0.02485200
H	-3.54798500	-1.66458000	0.24125000
H	-2.08635600	-2.60073100	0.58610500
H	-2.44368100	-2.08391000	-1.08683900
H	-3.14713300	1.04344400	-0.06568100
H	-2.35728500	0.44702700	-1.54116300
H	2.41545400	0.35622000	-1.62717000
H	3.73843300	-0.49563100	-0.84538700

BP86/6-31G*

SCF Done: E(RB-P86) = -612.548811008

Zero-point correction= 0.246975
Thermal correction to Gibbs Free Energy= 0.209285

H	3.37691700	1.26587000	0.66913200
C	2.50784400	0.59315800	0.53801800
H	2.50506000	-0.09032800	1.40641500
C	2.67506300	-0.23450500	-0.75215600
C	1.87430500	-1.55097400	-0.74659700
C	0.34210600	-1.41074900	-0.80222400
H	-0.09819700	-2.42443200	-0.78750600
H	0.04125600	-0.93929700	-1.75818600
C	-0.31966100	-0.58944200	0.32896400
C	1.26534600	1.49395400	0.58316900
O	0.09751600	-1.15318400	1.56075100
H	-0.52050800	-0.76223700	2.22062000
N	-0.00135200	0.85034300	0.21621900
N	-1.79802700	-0.54939500	0.28681800
H	2.18347800	-2.16424300	-1.61523600
H	2.14595900	-2.13025300	0.15613200
H	1.17087300	1.93388800	1.59641100
H	1.37283900	2.34399800	-0.11488000
C	-1.05377500	1.60497800	-0.25495500
O	-1.04277300	2.81716600	-0.47375200
C	-2.21874900	0.63333300	-0.45983300
C	-2.49773800	-1.78846900	-0.03014300
H	-3.56126000	-1.67429800	0.24579800
H	-2.07679800	-2.60926300	0.57433100
H	-2.45421200	-2.07350500	-1.10648600
H	-3.15952100	1.06052500	-0.06810000
H	-2.36270300	0.44706100	-1.55166200
H	2.40063800	0.37795300	-1.63530500
H	3.74544900	-0.48763400	-0.87419300

PBE0/6-31G*

SCF Done: E(RPBE-PBE) = -611.801130423

Zero-point correction= 0.247918
Thermal correction to Gibbs Free Energy= 0.210288

H	3.37191700	1.25799400	0.67284200
C	2.50282700	0.58755300	0.53942800
H	2.49925600	-0.09852800	1.40483200
C	2.66816400	-0.23437200	-0.75090800
C	1.86751100	-1.54729900	-0.74975900
C	0.33884900	-1.40535900	-0.80487400
H	-0.10292300	-2.41748000	-0.79359300
H	0.03816500	-0.93016300	-1.75807600
C	-0.31974000	-0.58812700	0.32817500
C	1.26344400	1.48743800	0.58750600
O	0.09653600	-1.15575100	1.55508800
H	-0.51904200	-0.76414400	2.21524900
N	-0.00038700	0.84833500	0.21911600
N	-1.79469600	-0.54575700	0.28518600
H	2.17650600	-2.15823100	-1.61872300
H	2.13764000	-2.12869100	0.15113000
H	1.17000300	1.92418900	1.60141500
H	1.37195000	2.33965800	-0.10685900
C	-1.04902300	1.60309000	-0.25433500
O	-1.03589600	2.81373900	-0.47347200
C	-2.21175900	0.63350400	-0.46109400
C	-2.49453900	-1.78169200	-0.02587200
H	-3.55659000	-1.66689800	0.25208300
H	-2.07258200	-2.60049000	0.57933100
H	-2.45389700	-2.06974800	-1.10066000
H	-3.15291800	1.06161800	-0.07335900
H	-2.35225300	0.44726300	-1.55261700
H	2.39365600	0.38103800	-1.63087200
H	3.73717200	-0.48775600	-0.87477000

M06/6-31G* with Solvent Correction

SCF Done: E(RM06) = -612.170680418

Zero-point correction= 0.253966
Thermal correction to Gibbs Free Energy= 0.216939

H	3.39053100	1.16179500	0.63816400
C	2.50766800	0.51791500	0.52129100
H	2.52593600	-0.18542000	1.36546600
C	2.59588100	-0.26532400	-0.78542800
C	1.79794600	-1.56492900	-0.76585100
C	0.28237700	-1.41600900	-0.77659100
H	-0.16470400	-2.41885000	-0.73606500
H	-0.04215900	-0.95874800	-1.72590700
C	-0.33423700	-0.58311300	0.34434300
C	1.29716900	1.42965200	0.63137800
O	0.06678400	-1.13456300	1.55982000
H	-0.49500500	-0.71802500	2.23502700
N	0.03270000	0.83455000	0.22374700
N	-1.79069700	-0.49348200	0.28853200
H	2.07433800	-2.17113600	-1.64054400
H	2.09577800	-2.15082000	0.11710000
H	1.20851300	1.80649900	1.66212900
H	1.42652400	2.30848700	-0.01460200
C	-0.97922700	1.60110300	-0.25989300
O	-0.94000700	2.80192100	-0.47819100
C	-2.15142200	0.67514200	-0.48136700
C	-2.52897100	-1.69592000	-0.02141800
H	-3.58612000	-1.53561800	0.22426100
H	-2.16224500	-2.52458600	0.59528900
H	-2.46996100	-1.98794700	-1.08658300
H	-3.08840800	1.13192200	-0.13651000
H	-2.26084700	0.46941300	-1.56633100
H	2.26804700	0.36848200	-1.62706600
H	3.64845000	-0.51289600	-0.98159800

M06-2X/6-31G* with Solvent Correction

SCF Done: E(RM062X) = -612.305987604

Zero-point correction= 0.257814
Thermal correction to Gibbs Free Energy= 0.220810

H	3.37777000	1.16957900	0.65968300
C	2.49764700	0.53026400	0.53214500
H	2.49297800	-0.17409800	1.37005700
C	2.59860900	-0.25111000	-0.78183200
C	1.80746300	-1.56168300	-0.76379300
C	0.28566400	-1.41866300	-0.78897400
H	-0.15628500	-2.41904900	-0.74051100
H	-0.03380800	-0.96456000	-1.73615300
C	-0.32716400	-0.57894600	0.33632700
C	1.28361300	1.44980200	0.62121500
O	0.09792100	-1.11936300	1.55080900
H	-0.46056700	-0.70893600	2.23318800
N	0.02667000	0.84028900	0.20593800
N	-1.78871000	-0.50342500	0.30551000
H	2.09390700	-2.16640200	-1.63151700
H	2.09549400	-2.13423900	0.12596500
H	1.18193200	1.83217900	1.64444800
H	1.42001600	2.31527700	-0.03430900
C	-0.99538700	1.59544700	-0.26474900
O	-0.96921600	2.79756900	-0.47803800
C	-2.16762000	0.65557900	-0.48051400
C	-2.50561300	-1.72565200	-0.00443200
H	-3.56081700	-1.58347300	0.24451600
H	-2.11638100	-2.53921500	0.61287700
H	-2.43822100	-2.01425900	-1.06508800
H	-3.10024300	1.10657400	-0.13008900
H	-2.27226800	0.44001300	-1.55864400
H	2.26753800	0.37878100	-1.61950200
H	3.65032200	-0.49209500	-0.96958800

D3(BJ)-PBE0/def2-TZVPP//BP86/SVP with Solvent Correction

SCF Done: E(RB-P86) = -612.122570286
SCF Done: E(RPBE-PBE) = -612.082742307

Zero-point correction= 0.244866
Thermal correction to Gibbs Free Energy= 0.207091

H	3.37036600	1.28666900	0.67403000
C	2.50337300	0.60468400	0.53945600
H	2.50054700	-0.07212900	1.41990500
C	2.69064600	-0.23562000	-0.73689100
C	1.88615100	-1.54690400	-0.74088800
C	0.35760400	-1.40515500	-0.80412600
H	-0.08692000	-2.42223300	-0.79531100
H	0.05745500	-0.93287300	-1.76531500
C	-0.31898400	-0.59120100	0.32449000
C	1.25784600	1.49835600	0.57229100
O	0.09529300	-1.14128900	1.55487600
H	-0.51470700	-0.76083200	2.22331800
N	-0.00827300	0.85502800	0.21212900
N	-1.78885700	-0.56201400	0.25618800
H	2.20243400	-2.16425200	-1.61001600
H	2.15349000	-2.13329800	0.16555700
H	1.16375100	1.95321400	1.58549600
H	1.37097000	2.34929500	-0.13355100
C	-1.06330100	1.59757000	-0.25336800
O	-1.06001800	2.81070400	-0.47092400
C	-2.22366400	0.62648300	-0.45586300
C	-2.49959000	-1.79878800	-0.01579400
H	-3.56432700	-1.67908700	0.27526100
H	-2.07543600	-2.61491100	0.60272700
H	-2.47790000	-2.11439100	-1.08982200
H	-3.16697200	1.05282900	-0.04948500
H	-2.38268100	0.46834900	-1.55656600
H	2.43988000	0.37375800	-1.63594200
H	3.76727200	-0.49307700	-0.83596700

19$_{\mathbf{RE}}$

B3LYP/6-31G*

SCF Done: E(RB3LYP) = -612.562882091

Zero-point correction= 0.256078
Thermal correction to Gibbs Free Energy= 0.218090

H	-0.88260600	1.35807800	-1.51532300
C	-0.91136300	2.04187400	-0.65884100
H	-1.29739900	2.99199600	-1.05017100
C	0.52309300	2.25162300	-0.08553900
C	1.65562800	1.45666200	-0.80424700
C	2.36672600	0.36947300	0.05108300
H	2.91644100	0.84219400	0.86912400
H	3.09505900	-0.16361400	-0.56971400
C	-1.95994900	1.49164500	0.33003800
N	-1.60286000	0.18006400	0.86992400
N	0.72426600	-1.52099900	-0.01804200
H	1.25566300	0.99453600	-1.71457500
H	2.43747700	2.14686300	-1.14270100
H	-2.09199300	2.17183200	1.17858900
H	-2.92393800	1.39871900	-0.17710200
C	-1.60238600	-0.92686800	0.06726200
O	-2.23813500	-1.02163600	-0.97810500
C	-0.57639400	-1.99525000	0.49135300
C	0.98659400	-1.82561800	-1.42220800
H	1.02003600	-2.91326500	-1.55344000
H	1.94710000	-1.41765400	-1.73304000
H	0.19518900	-1.42780700	-2.06837300
H	-0.90877700	0.17067900	1.61193000
C	1.36032000	-0.55374700	0.72050200
O	1.03342700	-0.33874700	1.89490700
H	-0.50784200	-2.11997200	1.57256800
H	-0.82442300	-2.94541700	0.01600800
H	0.76555600	3.31844500	-0.14011000
H	0.53867800	2.00522700	0.98233600

B3LYP/6-31G* with Solvent Correction

SCF Done: E(RB3LYP) = -612.571684157

Zero-point correction= 0.255660
Thermal correction to Gibbs Free Energy= 0.217432

H	-0.65758100	1.49183400	-1.52168500
C	-0.59215000	2.16281800	-0.65662300
H	-0.83761700	3.16261200	-1.03637600
C	0.85771500	2.15942500	-0.08343500
C	1.86103800	1.20308100	-0.79686400
C	2.39994400	0.02000800	0.05715200
H	3.01099900	0.40464700	0.87782500
H	3.04422000	-0.61086100	-0.56352100
C	-1.70997900	1.76404900	0.32833500
N	-1.54455300	0.40931500	0.85913700
N	0.49190600	-1.59388200	-0.03484600
H	1.40351300	0.80775500	-1.71105900
H	2.73985200	1.76841500	-1.12706700
H	-1.73994800	2.44945900	1.18159000
H	-2.67873900	1.81736700	-0.17522700
C	-1.73875900	-0.68956900	0.07479800
O	-2.44325500	-0.70136300	-0.93464000
C	-0.86072700	-1.89191700	0.47054600
C	0.72368800	-1.93418900	-1.43811700
H	0.56477000	-3.00889600	-1.57463300
H	1.74682900	-1.70282700	-1.72766300
H	0.03228500	-1.39756800	-2.09801100
H	-0.84042000	0.29668000	1.58344400
C	1.26813800	-0.75110800	0.71504600
O	0.97917300	-0.51240200	1.89971100
H	-0.81392300	-2.04076000	1.54975700
H	-1.23599300	-2.79359300	-0.01457600
H	1.25394100	3.17832500	-0.14546800
H	0.83554500	1.92391100	0.98704100

BP86/6-31G*

SCF Done: E(RB-P86) = -612.558624308

Zero-point correction= 0.248006
Thermal correction to Gibbs Free Energy= 0.209698

H	-0.54900400	1.46287100	-1.51427200
C	-0.43088800	2.17040000	-0.67217100
H	-0.60263100	3.17609200	-1.10144100
C	1.01525100	2.07987600	-0.08750700
C	1.96371500	1.06538400	-0.80394900
C	2.40438700	-0.16376800	0.05044900
H	3.05306700	0.16992700	0.87704100
H	2.99655100	-0.85620000	-0.57517800
C	-1.58276500	1.88404300	0.32087000
N	-1.52577300	0.52652000	0.87618400
N	0.37008100	-1.64067800	-0.01572300
H	1.48146600	0.70742400	-1.73305600
H	2.89100200	1.57410000	-1.12650800
H	-1.56563400	2.59328100	1.16767900
H	-2.55008800	1.99635200	-0.19646300
C	-1.78063600	-0.56081300	0.07164600
O	-2.43493400	-0.51697400	-0.97910100
C	-1.00289900	-1.82809200	0.50479800
C	0.54933500	-1.99207200	-1.42627900
H	0.38896100	-3.07819200	-1.55619200
H	1.56767100	-1.75184100	-1.75963700
H	-0.18206200	-1.45656200	-2.05982000
H	-0.81770000	0.35818400	1.60368600
C	1.20576100	-0.82601000	0.72210700
O	0.92007300	-0.52144800	1.90183800
H	-0.94880200	-1.95270600	1.59695800
H	-1.45087500	-2.71729000	0.03650400
H	1.47719000	3.08214700	-0.13215900
H	0.97205100	1.82520400	0.98794000

PBE0/6-31G*

SCF Done: E(RPBE-PBE) = -611.810519732

Zero-point correction=   0.248939
Thermal correction to Gibbs Free Energy=   0.210740

H	-0.55432800	1.44945300	-1.50769500
C	-0.43660100	2.16018400	-0.66920600
H	-0.61176200	3.16241000	-1.10240900
C	1.00731000	2.07642400	-0.08731900
C	1.95838200	1.06825000	-0.80181700
C	2.40058500	-0.15827600	0.04906100
H	3.04774600	0.17579800	0.87559300
H	2.99435800	-0.84784800	-0.57659200
C	-1.58467900	1.87508700	0.32392300
N	-1.52694600	0.52136600	0.87889700
N	0.37161100	-1.63535300	-0.01846800
H	1.47973700	0.71136000	-1.73220300
H	2.88379900	1.58010300	-1.12141000
H	-1.56726000	2.58552300	1.16858700
H	-2.55170300	1.98667200	-0.19276600
C	-1.77520900	-0.56379200	0.07285600
O	-2.42139400	-0.51808800	-0.98139000
C	-0.99592000	-1.82722200	0.50392600
C	0.54673800	-1.97760400	-1.42791400
H	0.37881800	-3.06086300	-1.56492100
H	1.56555900	-1.74262100	-1.76100800
H	-0.18131900	-1.43421000	-2.05770800
H	-0.82460300	0.35495400	1.61013400
C	1.20644600	-0.82333800	0.72015200
O	0.92352200	-0.52385800	1.90015600
H	-0.93882000	-1.95197700	1.59532000
H	-1.44276100	-2.71672800	0.03678100
H	1.46405100	3.07998000	-0.13268600
H	0.96650600	1.82319300	0.98788000

M06/6-31G* with Solvent Correction

SCF Done: E(RM06) = -612.178761963

Zero-point correction= 0.254671
Thermal correction to Gibbs Free Energy= 0.217121

H	-0.59908000	1.35570800	-1.46807900
C	-0.50255500	2.09433000	-0.65721900
H	-0.69935500	3.06947800	-1.12457000
C	0.92650700	2.06426800	-0.07184900
C	1.91208000	1.11398100	-0.78750800
C	2.39695200	-0.08346800	0.05281300
H	3.02652100	0.27252100	0.87666300
H	3.01344100	-0.74809300	-0.56548500
C	-1.62255200	1.81320700	0.34281500
N	-1.53209900	0.46494000	0.89202600
N	0.41434300	-1.57050700	-0.05709200
H	1.45769100	0.74567600	-1.71890300
H	2.80983400	1.66309700	-1.10015800
H	-1.59337900	2.51905200	1.18253100
H	-2.59854700	1.92012800	-0.14423600
C	-1.75431800	-0.61143800	0.08821300
O	-2.43303500	-0.57519800	-0.93072400
C	-0.92940200	-1.83926500	0.46263100
C	0.59317500	-1.84323300	-1.47331500
H	0.35706900	-2.89646900	-1.66776400
H	1.62663600	-1.66768100	-1.77711600
H	-0.07429400	-1.22357400	-2.08965700
H	-0.84031700	0.31992400	1.62446200
C	1.23883300	-0.79421100	0.70911300
O	0.97943400	-0.57800800	1.89622500
H	-0.87179900	-2.00770200	1.54138400
H	-1.33795000	-2.72500900	-0.03299200
H	1.33714500	3.08155600	-0.09499200
H	0.88716800	1.80097900	0.99618700

M06-2X/6-31G* with Solvent Correction

SCF Done: E(RM062X) = -612.311079786

Zero-point correction= 0.258724
Thermal correction to Gibbs Free Energy= 0.221282

C	2.25050100	0.31518000	0.07176400
H	2.37216700	0.21283400	1.15612900
H	3.25839400	0.35112800	-0.34661700
C	1.53885500	1.63040700	-0.25634200
H	1.33316900	1.67436600	-1.33172500
H	2.24336600	2.43846700	-0.03323100
C	0.24541800	1.91596600	0.52758800
H	0.39812500	1.73033600	1.59559600
H	0.00190000	2.97415500	0.41272500
C	-0.97674600	1.18766400	-0.01223500
N	-1.25998000	-0.06497400	0.46363900
O	-1.69897800	1.73078700	-0.83996700
C	-0.24839500	-0.89682800	1.10073900
H	-0.74836000	-1.67631500	1.67797200
H	0.36036500	-0.30246600	1.78162400
C	0.57268800	-1.58040300	0.00870800
O	0.20885200	-2.64748900	-0.47013200
N	1.63361800	-0.88965000	-0.47418000
H	2.08668000	-1.32991500	-1.26609700
C	-2.38173000	-0.77251300	-0.14356900
H	-2.10545800	-1.21830900	-1.10545600
H	-3.19734300	-0.06856600	-0.30004100
H	-2.70100300	-1.56656500	0.53379100

D3(BJ)-PBE0/def2-TZVPP//BP86/SVP with Solvent Correction

SCF Done: E(RB-P86) = -612.129828880
SCF Done: E(RPBE-PBE) = -612.091781932

Zero-point correction= 0.245568
Thermal correction to Gibbs Free Energy= 0.207091

H	-0.47520500	1.56957300	-1.55192700
C	-0.34299100	2.22869800	-0.66572000
H	-0.48405900	3.26362700	-1.04597200
C	1.10004700	2.06474700	-0.09423900
C	1.99355700	0.99501900	-0.79587500
C	2.39163100	-0.24290200	0.06096000
H	3.05515500	0.06970200	0.89144100
H	2.96389100	-0.95709400	-0.56615400
C	-1.50722900	1.93897300	0.30954800
N	-1.47640700	0.57650200	0.84101100
N	0.31323700	-1.64010800	-0.02568200
H	1.49017600	0.64973300	-1.72510700
H	2.94295000	1.46040500	-1.13521900
H	-1.48657000	2.64167300	1.16901400
H	-2.47294400	2.08614400	-0.21548800
C	-1.81641600	-0.50544300	0.07059700
O	-2.53708600	-0.44852500	-0.93273600
C	-1.06450800	-1.79466900	0.48038300
C	0.52908400	-2.04982300	-1.41173100
H	0.30474800	-3.13186500	-1.51977300
H	1.57862500	-1.88689900	-1.71055500
H	-0.13494900	-1.49109600	-2.10626900
H	-0.77192300	0.37874500	1.56919600
C	1.17325700	-0.86975500	0.72471400
O	0.88669700	-0.56153800	1.90210000
H	-1.03219100	-1.93174800	1.57828900
H	-1.53983800	-2.67001900	0.00143900
H	1.61561800	3.04472300	-0.16098800
H	1.05322100	1.84106500	0.99403500

Isomers 20_{RO}/20_{RC}/20_{RE}

Spartan Calculations

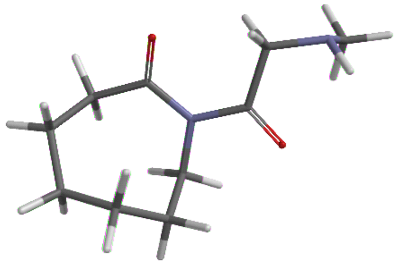

20_{RO}
ΔG ° = 7.3 kcal/mol

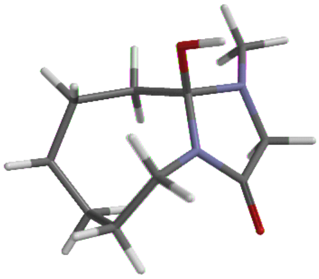

20_{RC}
ΔG ° = 14.1 kcal/mol

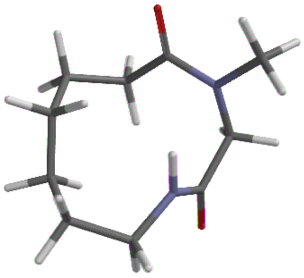

20_{RE}
ΔG ° = 0.00 kcal/mol

20_{RO}	Calculated energy (au)	Calculated energy (kcal/mol)	Relative energy (kcal/mol)
M0005	-651.861759	-409043.2538	0.00
M0001	-651.859793	-409042.0201	1.23
M0002	-651.859793	-409042.0201	1.23
M0006	-651.859186	-409041.6392	1.61
M0010	-651.859124	-409041.6003	1.65
M0007	-651.85897	-409041.5037	1.75
M0008	-651.85897	-409041.5037	1.75
M0020	-651.858928	-409041.4773	1.78
M0011	-651.857755	-409040.7413	2.51
M0003	-651.857642	-409040.6704	2.58
M0009	-651.857473	-409040.5643	2.69
M0027	-651.856967	-409040.2468	3.01
M0004	-651.856831	-409040.1615	3.09
M0012	-651.856661	-409040.0548	3.20
M0030	-651.856136	-409039.7253	3.53
M0015	-651.855993	-409039.6356	3.62
M0021	-651.855797	-409039.5126	3.74
M0022	-651.855674	-409039.4354	3.82
M0023	-651.855674	-409039.4354	3.82
M0024	-651.855134	-409039.0966	4.16
M0025	-651.855134	-409039.0966	4.16
M0031	-651.855131	-409039.0947	4.16
M0036	-651.854881	-409038.9378	4.32
M0018	-651.854806	-409038.8908	4.36
M0019	-651.854806	-409038.8908	4.36
M0052	-651.854692	-409038.8192	4.43
M0043	-651.853952	-409038.3549	4.90
M0063	-651.853613	-409038.1422	5.11
M0028	-651.853557	-409038.107	5.15
M0013	-651.85344	-409038.0336	5.22
M0016	-651.853391	-409038.0029	5.25
M0033	-651.852962	-409037.7337	5.52

M0037	-651.852667	-409037.5485	5.71
M0038	-651.852667	-409037.5485	5.71
M0040	-651.852649	-409037.5372	5.72
M0041	-651.852649	-409037.5372	5.72
M0044	-651.852625	-409037.5222	5.73
M0045	-651.852625	-409037.5222	5.73
M0047	-651.852615	-409037.5159	5.74
M0035	-651.852596	-409037.504	5.75
M0017	-651.851942	-409037.0936	6.16
M0014	-651.851802	-409037.0058	6.25
M0049	-651.851657	-409036.9148	6.34
M0091	-651.85133	-409036.7096	6.54
M0065	-651.851205	-409036.6311	6.62
M0066	-651.850915	-409036.4492	6.80
M0067	-651.850915	-409036.4492	6.80
M0077	-651.850652	-409036.2841	6.97
M0078	-651.850652	-409036.2841	6.97
M0029	-651.850509	-409036.1944	7.06
M0056	-651.849852	-409035.7821	7.47
M0034	-651.849478	-409035.5474	7.71
M0060	-651.849401	-409035.4991	7.75
M0061	-651.849401	-409035.4991	7.75
M0026	-651.848669	-409035.0398	8.21
M0032	-651.848337	-409034.8315	8.42
M0053	-651.848328	-409034.8258	8.43
M0085	-651.848307	-409034.8126	8.44
M0086	-651.848307	-409034.8126	8.44
M0087	-651.848307	-409034.8126	8.44
M0092	-651.847689	-409034.4248	8.83
M0088	-651.847666	-409034.4104	8.84
M0071	-651.847637	-409034.3922	8.86
M0082	-651.847576	-409034.3539	8.90
M0050	-651.847519	-409034.3182	8.94
M0046	-651.847205	-409034.1211	9.13
M0062	-651.847057	-409034.0283	9.23
M0057	-651.846796	-409033.8645	9.39
M0048	-651.846682	-409033.793	9.46
M0064	-651.846545	-409033.707	9.55
M0074	-651.846172	-409033.4729	9.78
M0054	-651.846133	-409033.4485	9.81
M0039	-651.845585	-409033.1046	10.15
M0042	-651.845291	-409032.9201	10.33
M0083	-651.845262	-409032.9019	10.35
M0058	-651.845226	-409032.8793	10.37
M0069	-651.845158	-409032.8366	10.42
M0073	-651.844498	-409032.4225	10.83

M0068	-651.844081	-409032.1608	11.09
M0075	-651.844034	-409032.1313	11.12
M0084	-651.843599	-409031.8584	11.40
M0080	-651.843234	-409031.6293	11.62
M0076	-651.842749	-409031.325	11.93
M0055	-651.842597	-409031.2296	12.02
M0079	-651.842363	-409031.0828	12.17
M0051	-651.842194	-409030.9767	12.28
M0070	-651.842118	-409030.929	12.32
M0059	-651.840036	-409029.6226	13.63
M0081	-651.838906	-409028.9135	14.34
M0089	-651.838282	-409028.522	14.73
M0090	-651.837536	-409028.0538	15.20
M0072	-651.835054	-409026.4964	16.76
**20_{RC}**	**Calculated energy (au)**	**Calculated energy (kcal/mol)**	**Relative energy (kcal/mol)**
M0001	-651.853682	-409038.1855	0.00
M0013	-651.852481	-409037.4318	0.75
M0003	-651.851386	-409036.7447	1.44
M0002	-651.851275	-409036.6751	1.51
M0007	-651.849246	-409035.4019	2.78
M0026	-651.848001	-409034.6206	3.56
M0020	-651.847921	-409034.5704	3.62
M0018	-651.84757	-409034.3502	3.84
M0005	-651.847203	-409034.1199	4.07
M0014	-651.846863	-409033.9065	4.28
M0009	-651.846578	-409033.7277	4.46
M0023	-651.846529	-409033.6969	4.49
M0016	-651.845736	-409033.1993	4.99
M0006	-651.84569	-409033.1705	5.01
M0015	-651.844748	-409032.5794	5.61
M0008	-651.844193	-409032.2311	5.95
M0010	-651.842839	-409031.3815	6.80
M0012	-651.841983	-409030.8443	7.34
M0004	-651.841953	-409030.8255	7.36
M0022	-651.841781	-409030.7176	7.47
M0029	-651.841574	-409030.5877	7.60
M0019	-651.841071	-409030.2721	7.91
M0021	-651.840815	-409030.1114	8.07
M0011	-651.839927	-409029.5542	8.63
M0017	-651.839922	-409029.5511	8.63
M0024	-651.838927	-409028.9267	9.26
M0025	-651.838454	-409028.6299	9.56
M0027	-651.838108	-409028.4128	9.77
M0028	-651.837434	-409027.9898	10.20
**20_{RE}**	**Calculated energy (au)**	**Calculated energy (kcal/mol)**	**Relative energy (kcal/mol)**

M0003	-651.876835	-409052.714	0.00
M0001	-651.875829	-409052.0827	0.63
M0005	-651.875321	-409051.7639	0.95
M0002	-651.874599	-409051.3109	1.40
M0007	-651.873945	-409050.9005	1.81
M0008	-651.872986	-409050.2987	2.42
M0014	-651.872716	-409050.1293	2.58
M0006	-651.872515	-409050.0032	2.71
M0017	-651.871756	-409049.5269	3.19
M0019	-651.871347	-409049.2702	3.44
M0011	-651.87117	-409049.1592	3.55
M0004	-651.871036	-409049.0751	3.64
M0012	-651.870474	-409048.7224	3.99
M0009	-651.870317	-409048.6239	4.09
M0013	-651.870242	-409048.5769	4.14
M0022	-651.869207	-409047.9274	4.79
M0023	-651.868913	-409047.7429	4.97
M0020	-651.868269	-409047.3388	5.38
M0024	-651.867731	-409047.0012	5.71
M0010	-651.867729	-409046.9999	5.71
M0018	-651.867494	-409046.8525	5.86
M0016	-651.867415	-409046.8029	5.91
M0015	-651.867387	-409046.7853	5.93
M0021	-651.866762	-409046.3932	6.32

Table S8. Energies calculated at DFT/B3LYP/6-31G* in vacuum for the conformers found for **20_{RO}/20_{RC}/20_{RE}**.

Isomer	ΔG° (au)	ΔG° (kcal/mol)	ΔG° (kcal/mol)
**20_{RO}**	-651.627638	-408896.34	7.31
**20_{RC}**	-651.616773	-408889.53	14.13
**20_{RE}**	-651.639291	-408903.66	0.00

Table S9. Relative free energies (ΔG°) of the lowest energy geometries of **20_{RO}/20_{RC}/20_{RE}** at DFT/B3LYP/6-31G* in vacuum in kcal/mol.

XYZ Coordinates

20_{RO}

H	-1.96429400	0.57796800	-1.61965100
C	-2.49660900	1.15943400	-0.85787600
H	-3.10252300	1.88764200	-1.41298300
C	-1.50577700	1.96001600	-0.01176700
H	-2.05685700	2.72610400	0.54834000
H	-0.83974800	2.50305600	-0.69336400
C	-0.67333300	1.15086400	0.98391900
H	0.02894200	1.83209300	1.48179900
H	-1.31010400	0.77537900	1.78610000
N	0.08429500	0.06551300	0.35999200
C	-0.49558200	-1.17398600	0.10380300
C	-3.44247700	0.26743200	-0.05315700
H	-4.42659400	0.30846300	-0.53859600
H	-3.59093800	0.65434600	0.96146600
C	-3.03313100	-1.20682300	-0.01524100
C	-1.79375000	-1.53008500	0.81066500
H	-3.87288600	-1.76983300	0.41206100
H	-2.90699000	-1.57627000	-1.04068200
H	-1.75948400	-2.61811700	0.96267200
H	-1.84837200	-1.12132000	1.82148200
O	-0.02723900	-1.98949300	-0.69316900
C	1.33643900	0.43679800	-0.15578000
O	1.66921300	1.62503500	-0.26319200
C	2.30908300	-0.67874600	-0.59159100
H	2.22946000	-1.55488900	0.05881800
H	2.05515600	-0.96168800	-1.61801100
N	3.70865300	-0.18899200	-0.59246300
H	3.69407600	0.75534300	-0.99052300
C	4.25219900	-0.11229500	0.75769700
H	5.28009900	0.26091800	0.71281300
H	4.28351600	-1.10140300	1.22588000
H	3.67874500	0.56657200	1.39652500

20_{RC}

H	3.27994400	0.53817800	-1.52239900
C	2.39730200	0.22657900	-0.94769500
H	1.54409600	0.45869600	-1.59421100
C	2.35336300	1.08646100	0.31322000
H	2.35803800	2.13543100	-0.01127200
H	3.27781400	0.94994700	0.88759700
C	1.16054700	0.88126200	1.24586000
H	1.33665100	0.05007900	1.93381300
H	1.06482700	1.77696700	1.87242800
N	-0.10668800	0.70458700	0.56485700
C	-0.77533800	-0.55657800	0.43914900
C	2.50569200	-1.28011800	-0.73306400
H	3.42973600	-1.50310000	-0.18513600
H	2.62924300	-1.73960800	-1.72293400
C	1.34320100	-1.98971900	-0.03527700
C	-0.06040100	-1.56319900	-0.49268000
H	1.45699700	-3.06067000	-0.25340500
H	1.45012900	-1.91793100	1.05109800
H	-0.65410600	-2.48745500	-0.50511900
H	-0.03045300	-1.19882700	-1.52620100
O	-0.89236300	-1.10228200	1.72722000
H	-1.59838300	-0.55263700	2.10476400
N	-2.11517900	-0.15400200	0.03165000
C	-0.71559900	1.69357800	-0.18482100
O	-0.31940100	2.83102100	-0.37534700
C	-1.96406200	1.07186800	-0.76183100
H	-2.81881600	1.73834700	-0.62149200
H	-1.78972500	0.87400300	-1.82507700
C	-2.93702900	-1.15139000	-0.62487300
H	-3.94991400	-0.76188500	-0.77712000
H	-3.03774800	-2.04534300	0.00003000
H	-2.54719800	-1.45066600	-1.60381800

20$_{RE}$

H	3.02741200	1.94488400	0.46451500
C	2.23138400	1.24208900	0.18688300
H	1.99135900	0.68644100	1.09932600
C	2.77111300	0.29143800	-0.89292700
H	3.86632100	0.26767500	-0.83383800
H	2.52709400	0.67415600	-1.89150900
C	2.26771300	-1.15059500	-0.75523000
H	2.50153500	-1.71146900	-1.66608300
C	-1.74176200	0.79289200	0.08792000
C	1.03860600	2.06824600	-0.31896700
H	0.51289300	1.55197500	-1.12904700
H	1.43363000	2.99054300	-0.76439500
C	0.03980400	2.45277000	0.77949600
C	-0.85159800	1.28663400	1.22614300
H	0.57874700	2.85224000	1.64673700
H	-0.59331700	3.26535700	0.40218000
H	-1.52087200	1.62603200	2.02559300
H	-0.22783200	0.50367600	1.65375400
O	-2.38155800	1.60401200	-0.58483500
H	2.78603000	-1.63903400	0.07682100
N	0.83650800	-1.22584700	-0.55798200
N	-1.78398000	-0.56975500	-0.18330200
C	-1.23822200	-1.60234300	0.70819900
H	-1.74932900	-2.55240000	0.51753700
H	-1.43256900	-1.31195600	1.74571500
C	0.27013500	-1.83438700	0.54138200
O	0.90223200	-2.52451800	1.33687000
H	0.21884000	-0.73792700	-1.19764200
C	-2.61542500	-1.04102000	-1.28074200
H	-2.85099700	-0.24085700	-1.98720500
H	-2.08484900	-1.83409300	-1.81601700
H	-3.54767700	-1.43632500	-0.86667800

Gaussian Calculations

20_{RO}

B3LYP/6-31G*

SCF Done: E(RB3LYP) = -651.868215391

Zero-point correction= 0.283557
Thermal correction to Gibbs Free Energy= 0.242366

H	-1.89937200	0.55987000	-1.63755700
C	-2.47336900	1.17550500	-0.93138600
H	-3.02006600	1.89538800	-1.55278200
C	-1.51188500	1.98311100	-0.04394400
H	-2.08317000	2.73611500	0.51746000
H	-0.80771200	2.53294900	-0.67814700
C	-0.71046800	1.17370400	0.98276400
H	0.00707300	1.82716400	1.47938000
H	-1.36771400	0.76252500	1.74886800
N	0.06336800	0.06415900	0.38659300
C	-0.52060300	-1.19328100	0.16147300
C	-3.48738000	0.28302300	-0.17954100
H	-4.44075000	0.29818300	-0.72246900
H	-3.70554300	0.71477500	0.80859800
C	-3.09245200	-1.19652300	-0.02560800
C	-1.85091100	-1.52089900	0.83739100
H	-3.93989300	-1.73586300	0.41617000
H	-2.93561800	-1.63437700	-1.02031100
H	-1.81212400	-2.60563000	0.97223700
H	-1.94686700	-1.07309600	1.83123200
O	0.00093500	-2.02775700	-0.56228000
C	1.35879900	0.41193900	-0.07606600
O	1.71078800	1.58011300	-0.01402200
C	2.32783000	-0.65551300	-0.61974700
H	2.27286900	-1.56876900	-0.02236300
H	1.97975900	-0.94321900	-1.61824800
N	3.69991400	-0.18991500	-0.68254100
H	3.70499900	0.73146100	-1.11597200
C	4.34909400	-0.09275200	0.62429600
H	5.36885300	0.28217800	0.48847500
H	4.42114100	-1.09429100	1.06526800
H	3.83544500	0.56621200	1.34442600

B3LYP/6-31G* with Solvent Correction

SCF Done: E(RB3LYP) = -651.876069111

Zero-point correction= 0.283330
Thermal correction to Gibbs Free Energy= 0.242206

H	-1.92507900	0.55893700	-1.64070000
C	-2.49227300	1.17103200	-0.92567800
H	-3.04946200	1.89022800	-1.53814000
C	-1.52347200	1.98149700	-0.04894700
H	-2.09063300	2.73179400	0.51915500
H	-0.82952100	2.53397800	-0.69259200
C	-0.71263900	1.17573000	0.97238400
H	0.00221100	1.82931100	1.47179600
H	-1.36309700	0.75826700	1.73946800
N	0.06606600	0.06971100	0.36957500
C	-0.51544700	-1.18957500	0.15203700
C	-3.49267500	0.27455700	-0.16122200
H	-4.45171900	0.28410800	-0.69370900
H	-3.70037400	0.70484300	0.82923700
C	-3.08897600	-1.20268300	-0.01253700
C	-1.83624100	-1.52218500	0.83793500
H	-3.92632200	-1.74605600	0.44195900
H	-2.94425000	-1.63970000	-1.00960200
H	-1.79431700	-2.60659700	0.97440800
H	-1.92295100	-1.07508400	1.83196700
O	0.00738200	-2.02219700	-0.57830600
C	1.35928100	0.41448800	-0.08456000
O	1.71839400	1.58297800	-0.02265100
C	2.33034700	-0.65430400	-0.62043800
H	2.26986500	-1.56460200	-0.01954700
H	1.98901000	-0.93716800	-1.62230800
N	3.70749100	-0.19381900	-0.67494300
H	3.71560800	0.72750900	-1.10940000
C	4.34150100	-0.08886000	0.64183700
H	5.36083100	0.29105200	0.51807600
H	4.40871800	-1.08708900	1.09026900
H	3.81393600	0.57058600	1.35001700

BP86/6-31G*

SCF Done: E(RB-P86) = -651.859950236

Zero-point correction= 0.274624
Thermal correction to Gibbs Free Energy= 0.233021

H	-1.89344800	0.53120400	-1.64112900
C	-2.47832500	1.16429800	-0.94470500
H	-3.02347400	1.87916500	-1.58909700
C	-1.52083200	1.98696400	-0.06003700
H	-2.10006500	2.75056900	0.49696500
H	-0.80782900	2.53729700	-0.70019700
C	-0.72027200	1.18479600	0.97984100
H	0.00441600	1.84655300	1.47745600
H	-1.38555600	0.76920700	1.75125200
N	0.06001100	0.07259600	0.38662500
C	-0.52323600	-1.19599100	0.16275000
C	-3.49854300	0.28050800	-0.18185600
H	-4.46299000	0.29696200	-0.72392300
H	-3.71216800	0.72345000	0.81289400
C	-3.10604600	-1.20285700	-0.01923000
C	-1.85746200	-1.52061100	0.84452000
H	-3.95935500	-1.74493800	0.43107900
H	-2.94945700	-1.65084000	-1.01987100
H	-1.81602900	-2.61358100	0.98872000
H	-1.95469000	-1.06204100	1.84460900
O	0.00994500	-2.04010600	-0.56111000
C	1.36866000	0.42318500	-0.06386500
O	1.73179500	1.59867700	0.02757300
C	2.33105400	-0.64308400	-0.63489300
H	2.27327200	-1.57741100	-0.05247800
H	1.97006000	-0.91378600	-1.64480500
N	3.71180600	-0.18126600	-0.69870300
H	3.70168300	0.77084400	-1.08815100
C	4.35864800	-0.12355100	0.61972400
H	5.38587900	0.26174600	0.49790500
H	4.43347300	-1.14789700	1.02902100
H	3.83776400	0.51368100	1.36910600

PBE0/6-31G*

SCF Done: E(RPBE-PBE) = -651.056479017

Zero-point correction= 0.275671
Thermal correction to Gibbs Free Energy= 0.234184

H	-1.87274300	0.53073200	-1.63752000
C	-2.45935500	1.16644800	-0.94616800
H	-2.99690700	1.88174300	-1.59467100
C	-1.50638900	1.98474200	-0.05806300
H	-2.08567200	2.74952300	0.49512200
H	-0.78777400	2.53200100	-0.69317300
C	-0.71579800	1.18026000	0.98337500
H	0.00885300	1.83880600	1.48414300
H	-1.38589200	0.76879900	1.75168900
N	0.05944800	0.06811000	0.39357600
C	-0.52678200	-1.19632300	0.16838100
C	-3.48554400	0.28910900	-0.19039600
H	-4.44608800	0.30952400	-0.73712200
H	-3.70258100	0.73337700	0.80200800
C	-3.10168800	-1.19293500	-0.02470700
C	-1.86215700	-1.51535400	0.84403500
H	-3.95964400	-1.73061400	0.41947100
H	-2.94064600	-1.64157800	-1.02347900
H	-1.82540900	-2.60776100	0.98706800
H	-1.96300400	-1.05816400	1.84339200
O	0.00348900	-2.04105100	-0.55465700
C	1.36392300	0.41654300	-0.06132900
O	1.72761000	1.59111000	0.02476800
C	2.32321100	-0.64952900	-0.62928300
H	2.26860800	-1.58036900	-0.04185700
H	1.95803000	-0.92772900	-1.63494400
N	3.69990400	-0.18751900	-0.70060700
H	3.68730600	0.75926300	-1.09965300
C	4.34672100	-0.11444700	0.61289100
H	5.37322500	0.26868400	0.48626800
H	4.42197800	-1.13298400	1.03420900
H	3.82725600	0.53105700	1.35496200

M06/6-31G* with Solvent Correction

SCF Done: E(RM06) = -651.446308049

Zero-point correction= 0.282221
Thermal correction to Gibbs Free Energy= 0.241503

H	-1.83860700	0.48525400	-1.60725700
C	-2.42506600	1.13326500	-0.93451700
H	-2.95832700	1.82500800	-1.60067500
C	-1.48633600	1.96812800	-0.06919400
H	-2.06951900	2.73215300	0.46811100
H	-0.77805300	2.50929000	-0.71194100
C	-0.70084800	1.18636300	0.97171800
H	0.01409800	1.84204600	1.47506800
H	-1.36226200	0.78292300	1.74232900
N	0.06870300	0.07579900	0.38643600
C	-0.52962800	-1.16822200	0.15086200
C	-3.44023300	0.28260200	-0.15956100
H	-4.40644300	0.31012900	-0.68203700
H	-3.62990600	0.73278800	0.82958900
C	-3.06584000	-1.18797100	0.00618000
C	-1.82694700	-1.49601700	0.85710800
H	-3.91298700	-1.72194900	0.45780400
H	-2.91485400	-1.63841200	-0.98805100
H	-1.77994100	-2.58132600	1.00646500
H	-1.91193600	-1.03775600	1.84905500
O	-0.04418500	-1.98046900	-0.61712600
C	1.35225600	0.41528300	-0.08398600
O	1.71671800	1.57634300	-0.03336500
C	2.29731700	-0.65911600	-0.61371500
H	2.22678900	-1.57010500	-0.00706200
H	1.94661200	-0.94929300	-1.61316300
N	3.67124400	-0.21755800	-0.66690000
H	3.69352700	0.70105900	-1.10602000
C	4.28144600	-0.11942400	0.65019500
H	5.30865000	0.24982200	0.55119900
H	4.33024100	-1.12003300	1.10078000
H	3.74629600	0.54437400	1.35244000

M06-2X/6-31G* with Solvent Correction

SCF Done: E(RM062X) = -651.592377993

Zero-point correction= 0.286675
Thermal correction to Gibbs Free Energy= 0.246197

H	-1.80470300	0.47152200	-1.58802300
C	-2.39046800	1.14115300	-0.94297400
H	-2.90912700	1.82338500	-1.62394500
C	-1.44677200	1.98032000	-0.07747400
H	-2.02287900	2.75379500	0.44540900
H	-0.71876200	2.49445600	-0.71329700
C	-0.69247200	1.17807200	0.97966600
H	0.02189300	1.81508200	1.50043400
H	-1.37922700	0.77734800	1.72373600
N	0.06846400	0.06230800	0.39413100
C	-0.53705500	-1.17384300	0.14729000
C	-3.42502100	0.31815900	-0.15068600
H	-4.39176700	0.36991100	-0.66208800
H	-3.58538900	0.77022900	0.83820400
C	-3.08407600	-1.16716800	0.01022700
C	-1.84069300	-1.50447500	0.85377300
H	-3.93529100	-1.67753300	0.47250500
H	-2.95122200	-1.61573600	-0.98234200
H	-1.80289200	-2.58906100	0.98111000
H	-1.91128000	-1.05973000	1.84839000
O	-0.05139100	-1.98080500	-0.62481100
C	1.34457300	0.39347200	-0.09233200
O	1.71037300	1.55393500	-0.05574500
C	2.29664700	-0.69098700	-0.61529300
H	2.22269300	-1.59020900	0.00122800
H	1.95788400	-0.97901400	-1.61470300
N	3.67070000	-0.23781800	-0.65811300
H	3.68719600	0.66752000	-1.12384700
C	4.24721400	-0.07975200	0.67453000
H	5.26572100	0.30522800	0.58255400
H	4.30088200	-1.06086900	1.15729400
H	3.67901000	0.59750000	1.32933200

D3(BJ)-PBE0/def2-TZVPP//BP86/SVP with Solvent Correction

SCF Done: E(RB-P86) = -651.401523810
SCF Done: E(RPBE-PBE) = -651.350917078

Zero-point correction= 0.272269
Thermal correction to Gibbs Free Energy= 0.230735

H	-1.89961400	0.56156500	-1.67404400
C	-2.48722900	1.17644200	-0.95453700
H	-3.03392400	1.90836000	-1.58673300
C	-1.53531500	1.98251000	-0.05198000
H	-2.11948400	2.74119900	0.51583400
H	-0.82304700	2.55271500	-0.68573400
C	-0.73657100	1.17554800	0.98306400
H	-0.01961800	1.84259900	1.49653400
H	-1.40464500	0.75232700	1.75488300
N	0.05380400	0.07121500	0.39354000
C	-0.52162300	-1.20226800	0.18530900
C	-3.50882500	0.27342200	-0.22358000
H	-4.46043400	0.27429100	-0.79739700
H	-3.76512300	0.71678400	0.76641600
C	-3.10328300	-1.20191800	-0.04631100
C	-1.86995200	-1.51046200	0.83882000
H	-3.96274000	-1.75287300	0.39257300
H	-2.93109400	-1.65793300	-1.04727500
H	-1.83484100	-2.60628800	1.00019200
H	-1.98004700	-1.03727200	1.83556300
O	0.03249800	-2.05866000	-0.50307400
C	1.36731300	0.42074300	-0.02822100
O	1.74030700	1.58696200	0.08958200
C	2.32538700	-0.63450800	-0.62021800
H	2.27972800	-1.57261300	-0.03116900
H	1.92212200	-0.91579200	-1.61797500
N	3.69570900	-0.16828200	-0.72718600
H	3.68444700	0.77348700	-1.14221900
C	4.41045600	-0.11191800	0.54823000
H	5.43629800	0.27710800	0.38163700
H	4.51076800	-1.13684100	0.96684300
H	3.93007400	0.52668500	1.33207200

20_{RC}

B3LYP/6-31G*

SCF Done: E(RB3LYP) = -651.861781949

Zero-point correction= 0.284170
Thermal correction to Gibbs Free Energy= 0.245804

H	3.29745700	0.54943600	-1.56516100
C	2.43441000	0.23933000	-0.96116700
H	1.55531400	0.45033300	-1.58353000
C	2.38267700	1.14725500	0.27521900
H	2.39260300	2.18555900	-0.07415000
H	3.28491500	1.00953000	0.88759500
C	1.15414700	0.96748500	1.19165600
H	1.28270800	0.13969100	1.89088300
H	1.02057200	1.87844900	1.78558000
N	-0.09411300	0.72373900	0.47713200
C	-0.76689800	-0.58289900	0.43114800
C	2.54855800	-1.27716800	-0.73440100
H	3.48846500	-1.49498300	-0.20743100
H	2.64751700	-1.74381900	-1.72496400
C	1.40185400	-1.98906700	0.01600600
C	-0.03296600	-1.62828900	-0.43737800
H	1.54479100	-3.06704200	-0.12846200
H	1.48584400	-1.83572100	1.09575500
H	-0.64960700	-2.53199600	-0.41308900
H	-0.03206700	-1.27391300	-1.47592800
O	-0.86387900	-1.15961200	1.71640300
H	-1.49300700	-0.59693300	2.20142000
N	-2.10193300	-0.19520700	-0.03905100
C	-0.76979700	1.71503900	-0.17450300
O	-0.42246400	2.88147700	-0.27563800
C	-2.02114600	1.06813700	-0.75482700
H	-2.90121500	1.69850600	-0.58483600
H	-1.89921100	0.95286400	-1.84840700
C	-2.95686600	-1.21657400	-0.61537000
H	-3.98097300	-0.83056400	-0.66584700
H	-2.96288300	-2.09725500	0.03306700
H	-2.66200600	-1.52627900	-1.63347500

B3LYP/6-31G* with Solvent Correction

SCF Done: E(RB3LYP) = -651.869585692

Zero-point correction= 0.283838
Thermal correction to Gibbs Free Energy= 0.245468

H	3.29749800	0.55536300	-1.56854000
C	2.43485300	0.24354100	-0.96497900
H	1.55466500	0.45155700	-1.58700200
C	2.38397100	1.14999700	0.27284600
H	2.40753300	2.18913700	-0.07468900
H	3.28173100	1.00431900	0.88907600
C	1.15214900	0.97760400	1.18656700
H	1.27919500	0.15247800	1.88827200
H	1.01814200	1.88804000	1.78086200
N	-0.09686500	0.73012000	0.47173500
C	-0.76596100	-0.58432700	0.43471500
C	2.55231900	-1.27229800	-0.73741700
H	3.49531000	-1.48766300	-0.21534900
H	2.64403100	-1.73979500	-1.72797500
C	1.41072400	-1.98235900	0.02157100
C	-0.02648500	-1.62783000	-0.42976400
H	1.55528900	-3.06056700	-0.11803800
H	1.50235000	-1.82113300	1.09974700
H	-0.63991400	-2.53352000	-0.40299100
H	-0.02912900	-1.27772800	-1.46924800
O	-0.85442500	-1.14823000	1.72643800
H	-1.48417500	-0.58660700	2.21309100
N	-2.10291900	-0.20614100	-0.03431400
C	-0.78191800	1.70776500	-0.17663400
O	-0.44613400	2.88425700	-0.28034900
C	-2.02716900	1.05493300	-0.75567700
H	-2.91226600	1.67970100	-0.59105400
H	-1.90142200	0.93511600	-1.84776800
C	-2.94840300	-1.23364500	-0.61998700
H	-3.97233200	-0.85022200	-0.68220400
H	-2.95917000	-2.11370600	0.02884300
H	-2.63885600	-1.54112400	-1.63313800

BP86/6-31G*

SCF Done: E(RB-P86) = -651.854603132

Zero-point correction= 0.275239
Thermal correction to Gibbs Free Energy= 0.236511

H	3.29171600	0.56524500	-1.58781600
C	2.43327000	0.24527100	-0.96603700
H	1.53554200	0.45098500	-1.58088000
C	2.38742100	1.15569200	0.27384600
H	2.39533900	2.20309300	-0.07753300
H	3.29818700	1.01709300	0.88967900
C	1.15468900	0.97504400	1.19346200
H	1.27910000	0.13977200	1.90046500
H	1.01499800	1.89540100	1.78956800
N	-0.09402800	0.72769300	0.47376100
C	-0.76400500	-0.58929200	0.43304000
C	2.55995200	-1.27421500	-0.73855200
H	3.51089600	-1.48817600	-0.21053100
H	2.65900800	-1.74583500	-1.73712600
C	1.41437800	-1.99009600	0.01811100
C	-0.02482400	-1.63335100	-0.44009900
H	1.56273200	-3.07781000	-0.11805000
H	1.49587500	-1.82695000	1.10646500
H	-0.64620400	-2.54558300	-0.42119300
H	-0.02324600	-1.27073500	-1.48600700
O	-0.85461200	-1.17019800	1.72761400
H	-1.50028700	-0.59643400	2.20021500
N	-2.11228600	-0.20272000	-0.02835200
C	-0.78254400	1.72490800	-0.17664500
O	-0.44082500	2.90485200	-0.27189100
C	-2.03408000	1.06060700	-0.75811300
H	-2.92675400	1.69134000	-0.59530900
H	-1.90805200	0.93490200	-1.86074700
C	-2.95207000	-1.23639400	-0.61972100
H	-3.98963800	-0.86126500	-0.67263400
H	-2.94818600	-2.12751900	0.02998400
H	-2.64645300	-1.53860700	-1.64796100

PBE0/6-31G*

SCF Done: E(RPBE-PBE) = -651.052906628

Zero-point correction= 0.276301
Thermal correction to Gibbs Free Energy= 0.237672

H	3.28432900	0.55546500	-1.58168300
C	2.42576800	0.23742600	-0.96098900
H	1.52956000	0.45093000	-1.57404000
C	2.38608000	1.14056800	0.28045400
H	2.39552000	2.18868600	-0.06612500
H	3.29705000	0.99654800	0.89281800
C	1.15670500	0.96021700	1.19940800
H	1.27821000	0.12173300	1.90209500
H	1.02282700	1.87753000	1.80030000
N	-0.09052000	0.72403500	0.48122500
C	-0.76774200	-0.58515300	0.43310500
C	2.54451000	-1.28067400	-0.74125400
H	3.49334200	-1.50129900	-0.21431000
H	2.64220000	-1.74711800	-1.74122100
C	1.39759800	-1.99477800	0.00886000
C	-0.03679400	-1.62820300	-0.44446800
H	1.54055900	-3.08110300	-0.13562500
H	1.48005800	-1.84146700	1.09787000
H	-0.66369500	-2.53570000	-0.42826700
H	-0.03523200	-1.26171100	-1.48822000
O	-0.86421000	-1.17171000	1.72111800
H	-1.50202900	-0.59298700	2.19609000
N	-2.10882700	-0.18685300	-0.02931300
C	-0.76393800	1.72411500	-0.17600100
O	-0.40917000	2.89853700	-0.27446600
C	-2.01649000	1.07033200	-0.76004100
H	-2.90361600	1.70938200	-0.60443800
H	-1.88566200	0.94087000	-1.86098500
C	-2.95665700	-1.21160100	-0.61527200
H	-3.99046400	-0.82856500	-0.66748400
H	-2.95899400	-2.10052200	0.03627600
H	-2.65572600	-1.51905400	-1.64245700

M06/6-31G* with Solvent Correction

SCF Done: E(RM06) = -651.448846566

Zero-point correction= 0.282919
Thermal correction to Gibbs Free Energy= 0.244920

H	3.20980300	0.49015000	-1.59188100
C	2.36228400	0.18385000	-0.96147100
H	1.46364800	0.46392100	-1.53491500
C	2.41018400	1.01035700	0.31719200
H	2.51211500	2.06660300	0.03338800
H	3.30996800	0.75417100	0.89796700
C	1.19293700	0.87887300	1.23940000
H	1.28365700	0.03230500	1.92736800
H	1.10876000	1.78117000	1.86004300
N	-0.06489100	0.71098300	0.53409500
C	-0.80095300	-0.55651000	0.45688700
C	2.42369900	-1.33158200	-0.80388300
H	3.38419700	-1.60462500	-0.33806400
H	2.44542300	-1.76869200	-1.81515900
C	1.29743700	-2.00825200	-0.01909300
C	-0.12231600	-1.60001900	-0.43140900
H	1.40455700	-3.09202000	-0.16399200
H	1.42706400	-1.85673300	1.06041800
H	-0.77472800	-2.48285800	-0.41713700
H	-0.12969700	-1.22518000	-1.46792600
O	-0.94012000	-1.14272000	1.71639200
H	-1.51467000	-0.54397800	2.22236500
N	-2.09455000	-0.08832200	-0.02885800
C	-0.64638500	1.71289400	-0.17059900
O	-0.21960000	2.85046000	-0.29422300
C	-1.89586400	1.12756500	-0.78529400
H	-2.74682700	1.81657800	-0.70491200
H	-1.71392200	0.94683100	-1.86564100
C	-2.98217400	-1.05959600	-0.62447600
H	-3.98132600	-0.61635400	-0.71782800
H	-3.06328200	-1.93596500	0.02894600
H	-2.66397800	-1.39134400	-1.63057200

M06-2X/6-31G* with Solvent Correction

SCF Done: E(RM062X) = -651.596746621

Zero-point correction= 0.287149
Thermal correction to Gibbs Free Energy= 0.249105

H	3.19836200	0.47319300	-1.60046300
C	2.35679000	0.17327500	-0.96549600
H	1.45338200	0.44585500	-1.52662400
C	2.41313700	1.01111100	0.31185400
H	2.48925000	2.06361500	0.02168500
H	3.31716000	0.76872800	0.88396400
C	1.19880300	0.86304900	1.24441600
H	1.29173700	0.00667300	1.91338800
H	1.11596800	1.75631600	1.87210300
N	-0.05892200	0.70614800	0.53423500
C	-0.80741600	-0.55156300	0.45221500
C	2.41966300	-1.34671300	-0.79474400
H	3.36943600	-1.61378200	-0.31375200
H	2.45032400	-1.78846900	-1.79909300
C	1.27622300	-2.01948100	-0.01882700
C	-0.14056100	-1.59800500	-0.45014500
H	1.37573900	-3.09958600	-0.16780200
H	1.38894600	-1.86755300	1.05838700
H	-0.79865200	-2.47177700	-0.44400200
H	-0.13377100	-1.21108300	-1.47762900
O	-0.93731100	-1.14552400	1.71081800
H	-1.50538200	-0.54936900	2.22791300
N	-2.10589400	-0.06738600	-0.01497900
C	-0.62595800	1.71312500	-0.16812300
O	-0.17970600	2.84337200	-0.29259800
C	-1.89570100	1.14833000	-0.77995100
H	-2.72966900	1.84908200	-0.68249900
H	-1.72344900	0.96483900	-1.85560800
C	-2.98862300	-1.04264800	-0.62579900
H	-3.98275200	-0.59879200	-0.72659900
H	-3.07014200	-1.91539600	0.02695600
H	-2.65477500	-1.36949700	-1.62328300

D3(BJ)-PBE0/def2-TZVPP//BP86/SVP with Solvent Correction

SCF Done: E(RB-P86) = -651.400916875
SCF Done: E(RPBE-PBE) = -651.353285872

Zero-point correction= 0.272875
Thermal correction to Gibbs Free Energy= 0.234115

H	3.31419900	0.58925900	-1.58404700
C	2.44896800	0.26294200	-0.96638300
H	1.55531900	0.45462000	-1.60183500
C	2.37809900	1.18063200	0.26443000
H	2.38806400	2.23163300	-0.09449900
H	3.28698900	1.05671000	0.89466100
C	1.14086500	0.99537000	1.17352400
H	1.27041900	0.16108100	1.88943200
H	0.99599700	1.91688900	1.77744700
N	-0.10342100	0.73507600	0.45600200
C	-0.75568700	-0.59554200	0.43024600
C	2.58734800	-1.25090100	-0.72505200
H	3.54209100	-1.45265200	-0.18862800
H	2.70005000	-1.73229700	-1.72287800
C	1.44608700	-1.97198000	0.02881900
C	0.00541300	-1.63571100	-0.42834600
H	1.60699800	-3.06340300	-0.10058200
H	1.52360100	-1.80659200	1.12309800
H	-0.60730400	-2.55910100	-0.39765100
H	-0.00454300	-1.28850900	-1.48414700
O	-0.83611300	-1.15238300	1.72920200
H	-1.48418700	-0.59328000	2.20894700
N	-2.09540200	-0.24168100	-0.05547400
C	-0.82044800	1.71511800	-0.17753000
O	-0.50862900	2.90393300	-0.26653100
C	-2.06156100	1.03391100	-0.74977300
H	-2.96836300	1.65096300	-0.56540500
H	-1.94656300	0.94460300	-1.86334100
C	-2.94318200	-1.28174900	-0.60932900
H	-3.99112800	-0.91702300	-0.64501500
H	-2.92520400	-2.17164800	0.05116900
H	-2.66214400	-1.59995300	-1.64542400

20$_{RE}$

B3LYP/6-31G*

SCF Done: E(RB3LYP) = -651.881797360

Zero-point correction= 0.283773
Thermal correction to Gibbs Free Energy= 0.242957

H	2.98147900	1.96034800	0.52400300
C	2.21860300	1.25505300	0.17240700
H	1.94295000	0.65838500	1.05101200
C	2.82806700	0.31754500	-0.89479400
H	3.92242400	0.29631600	-0.81584400
H	2.60915700	0.71139600	-1.89732000
C	2.34652000	-1.14327000	-0.78641000
H	2.66118000	-1.71118700	-1.67140800
C	-1.78658200	0.73853900	0.07856900
C	1.00256400	2.06156600	-0.33991200
H	0.48663100	1.50637600	-1.13478600
H	1.36752700	2.97571700	-0.82627500
C	-0.03352100	2.44268400	0.73030700
C	-0.89213000	1.25476400	1.20996400
H	0.46948000	2.88836400	1.59852600
H	-0.71164800	3.19944900	0.32380500
H	-1.56822600	1.60018900	2.00322200
H	-0.26352400	0.47947700	1.65221100
O	-2.44282800	1.52838200	-0.59250000
H	2.79061800	-1.62444900	0.08846700
N	0.90452200	-1.27546800	-0.60450300
N	-1.81357700	-0.62270100	-0.18076400
C	-1.17124600	-1.61844500	0.67304000
H	-1.59855600	-2.59619500	0.42795100
H	-1.38252300	-1.45182000	1.73316900
C	0.35964500	-1.74234400	0.55263700
O	0.99729800	-2.25643000	1.46400700
H	0.28286200	-0.79614400	-1.24205100
C	-2.73241900	-1.11320900	-1.20856900
H	-3.04219600	-0.26811700	-1.82156600
H	-2.23341700	-1.86279400	-1.83279000
H	-3.62360200	-1.57103100	-0.75894400

B3LYP/6-31G* with Solvent Correction

SCF Done: E(RB3LYP) = -651.892444347

Zero-point correction= 0.283611
Thermal correction to Gibbs Free Energy= 0.242746

H	2.96970400	1.99288100	0.51344900
C	2.21450800	1.27616000	0.16909500
H	1.94623100	0.68430100	1.05362300
C	2.83235700	0.33799700	-0.89247100
H	3.92637300	0.32565900	-0.81182900
H	2.60936500	0.72174500	-1.89728000
C	2.36372000	-1.12601100	-0.77598600
H	2.67671400	-1.69490500	-1.65988600
C	-1.79964500	0.72435000	0.08560000
C	0.98790800	2.06500300	-0.34570300
H	0.47917200	1.49801700	-1.13662400
H	1.34235400	2.98081500	-0.83636900
C	-0.04840500	2.44017100	0.72691500
C	-0.90306400	1.24984500	1.20926200
H	0.45422700	2.88529000	1.59524100
H	-0.72754200	3.19934600	0.32561600
H	-1.57573000	1.59155500	2.00670400
H	-0.26887300	0.47854700	1.64917600
O	-2.48335000	1.51237100	-0.57038900
H	2.81631900	-1.59871200	0.09862100
N	0.92156500	-1.26272700	-0.59068600
N	-1.80414800	-0.62671200	-0.18883100
C	-1.16116300	-1.62214000	0.66666500
H	-1.58869100	-2.59937200	0.42268400
H	-1.37761100	-1.45136900	1.72457600
C	0.36825600	-1.74367400	0.54837300
O	1.00169200	-2.27078300	1.46361700
H	0.30097600	-0.81744000	-1.25409000
C	-2.71041900	-1.12661600	-1.22655300
H	-2.96180400	-0.30529100	-1.89523500
H	-2.21950600	-1.92462300	-1.79116200
H	-3.63464500	-1.52357800	-0.78759500

BP86/6-31G*

SCF Done: E(RB-P86) = -651.87409237

Zero-point correction= 0.274952
Thermal correction to Gibbs Free Energy= 0.233959

H	2.99925600	1.94700900	0.55605000
C	2.22562400	1.24948700	0.18458200
H	1.93554400	0.63570500	1.05912600
C	2.83109800	0.31891800	-0.89694500
H	3.93440600	0.28622200	-0.81926400
H	2.61363500	0.73063800	-1.90287000
C	2.33409800	-1.14281900	-0.80451000
H	2.64145300	-1.71036900	-1.70406900
C	-1.77876500	0.75412800	0.07858800
C	1.01560000	2.07485400	-0.32345500
H	0.48970100	1.52522600	-1.12958400
H	1.39133100	2.99631600	-0.80873400
C	-0.02313700	2.45376300	0.75069600
C	-0.87968100	1.25530000	1.21983400
H	0.48149700	2.90097200	1.62865800
H	-0.71049000	3.21502800	0.34239200
H	-1.55617900	1.58754500	2.03123800
H	-0.24037400	0.46626800	1.64776800
O	-2.42260200	1.55701300	-0.61033800
H	2.77583900	-1.64547600	0.07295500
N	0.88861300	-1.26225800	-0.61167600
N	-1.82697400	-0.61800200	-0.17121600
C	-1.18333700	-1.62065300	0.68327500
H	-1.63257400	-2.60202300	0.44975800
H	-1.38206800	-1.44085900	1.75377000
C	0.35161000	-1.76662600	0.54381000
O	0.99868800	-2.33003300	1.43412200
H	0.25665800	-0.73832500	-1.21954700
C	-2.74362100	-1.10199500	-1.20980000
H	-3.08080600	-0.23107000	-1.78931200
H	-2.22924800	-1.82154000	-1.87197700
H	-3.62467200	-1.60141900	-0.76283100

PBE0/6-31G*

SCF Done: E(RPBE-PBE) = -651.071405372

Zero-point correction= 0.275967
Thermal correction to Gibbs Free Energy= 0.235057

H	2.98897200	1.93971300	0.55729900
C	2.21652600	1.24356400	0.18369700
H	1.92657400	0.62817500	1.05618400
C	2.82141400	0.31756500	-0.89744400
H	3.92372300	0.28517200	-0.82030900
H	2.60380400	0.73136100	-1.90145400
C	2.32580800	-1.14176500	-0.80830200
H	2.63267500	-1.70663400	-1.70861400
C	-1.77447600	0.75049100	0.07659400
C	1.00971000	2.06858900	-0.32269800
H	0.48318600	1.52058600	-1.12838600
H	1.38646600	2.98844600	-0.80798200
C	-0.02661900	2.44887400	0.74874400
C	-0.88238700	1.25430500	1.21855200
H	0.47751900	2.89772800	1.62487200
H	-0.71331500	3.20857600	0.33857100
H	-1.56235900	1.58951400	2.02455900
H	-0.24543900	0.46843900	1.65366900
O	-2.40937900	1.55137700	-0.62064200
H	2.76973000	-1.64533100	0.06679000
N	0.88453500	-1.26360400	-0.61327200
N	-1.82707200	-0.62059400	-0.16429700
C	-1.18015000	-1.61725000	0.68772300
H	-1.62538300	-2.60021300	0.45670400
H	-1.37757300	-1.43771100	1.75776600
C	0.35252000	-1.75655500	0.54748400
O	1.00400200	-2.30414700	1.44256000
H	0.25194700	-0.74813500	-1.22569000
C	-2.72871400	-1.10710800	-1.20888800
H	-3.07532100	-0.23630600	-1.78203400
H	-2.20252900	-1.81282900	-1.87575100
H	-3.60368800	-1.62326400	-0.77132200

M06/6-31G* with Solvent Correction

SCF Done: E(RM06) = -651.466466239

Zero-point correction= 0.282347
Thermal correction to Gibbs Free Energy= 0.242183

H	2.84378800	2.02824400	0.53101400
C	2.12105200	1.28914700	0.15724700
H	1.85984500	0.67167600	1.03184400
C	2.77317300	0.39832400	-0.90612100
H	3.86939500	0.44113900	-0.84419700
H	2.51602200	0.77578000	-1.90876500
C	2.37298900	-1.07098000	-0.78377800
H	2.70871900	-1.64047300	-1.66076400
C	-1.80296800	0.67700300	0.07873300
C	0.88704000	2.03426000	-0.36091900
H	0.37249800	1.42922400	-1.12581300
H	1.22009900	2.93664900	-0.89506900
C	-0.12329400	2.41763300	0.71545100
C	-0.94742500	1.22610300	1.20748200
H	0.39460900	2.87344100	1.57249700
H	-0.81751200	3.17110500	0.32226500
H	-1.63967400	1.55691100	1.99532400
H	-0.29696900	0.47353200	1.66727200
O	-2.47526200	1.43949500	-0.60627300
H	2.84484300	-1.51833400	0.09858100
N	0.94383500	-1.26038100	-0.59493200
N	-1.77552600	-0.67464500	-0.16683800
C	-1.10065900	-1.62960600	0.69274600
H	-1.48713500	-2.62925500	0.45458500
H	-1.33132200	-1.46487700	1.75211000
C	0.41903000	-1.68032700	0.57997000
O	1.07952000	-2.09838600	1.52348700
H	0.30732900	-0.88283500	-1.28769500
C	-2.59390700	-1.21795300	-1.24003000
H	-2.93772200	-0.39545100	-1.86908400
H	-2.00304300	-1.91900100	-1.84385400
H	-3.46618700	-1.75280100	-0.84026800

M06-2X/6-31G* with Solvent Correction

SCF Done: E(RM062X) = -651.610933588

Zero-point correction= 0.286821
Thermal correction to Gibbs Free Energy= 0.246546

H	2.89827900	1.95315800	0.52321700
C	2.16611500	1.22676100	0.15467000
H	1.88977700	0.61204300	1.02193900
C	2.80343200	0.32192600	-0.91294000
H	3.89535200	0.31818100	-0.82974900
H	2.57244400	0.71501300	-1.91114100
C	2.33554200	-1.13574000	-0.80224500
H	2.62355300	-1.70450800	-1.69180100
C	-1.77733700	0.70453100	0.07765100
C	0.94297200	1.99209700	-0.37537100
H	0.41440500	1.38441500	-1.12277600
H	1.29391600	2.87790500	-0.91774000
C	-0.05688500	2.40937300	0.70571400
C	-0.89356000	1.22528500	1.20706800
H	0.47231400	2.86007300	1.55334700
H	-0.74265700	3.16256700	0.30816600
H	-1.56950500	1.55694300	2.00395900
H	-0.24947400	0.45753100	1.63997700
O	-2.43214000	1.49042600	-0.59728800
H	2.79955800	-1.60952500	0.06507300
N	0.90087400	-1.26232300	-0.59147900
N	-1.79546000	-0.64256800	-0.17480100
C	-1.14918000	-1.61156000	0.69338200
H	-1.52878100	-2.60394100	0.43343400
H	-1.38972800	-1.44833200	1.74679600
C	0.37820500	-1.65388400	0.59051300
O	1.04018300	-2.04147100	1.54623000
H	0.26391900	-0.91249600	-1.29593800
C	-2.67576500	-1.15192600	-1.22029700
H	-2.90521000	-0.34045300	-1.90769200
H	-2.17251600	-1.95889000	-1.75922700
H	-3.60912000	-1.53827700	-0.79629200

D3(BJ)-PBE0/def2-TZVPP//BP86/SVP with Solvent Correction

SCF Done: E(RB-P86) = -651.420291941
SCF Done: E(RPBE-PBE) = -651.373360170

Zero-point correction= 0.272602
Thermal correction to Gibbs Free Energy= 0.231807

H	3.06663300	1.89578500	0.54739300
C	2.27270400	1.20827300	0.18524500
H	1.97654900	0.61243500	1.07761100
C	2.85492000	0.25238800	-0.88250000
H	3.95884300	0.17364200	-0.78329400
H	2.67792500	0.67680100	-1.89651000
C	2.29923800	-1.18792000	-0.80674000
H	2.57993800	-1.75476900	-1.72177700
C	-1.76279400	0.78777900	0.07539800
C	1.07727000	2.05342400	-0.32021500
H	0.54791500	1.51471900	-1.13850300
H	1.47026600	2.97480000	-0.80357600
C	0.03988800	2.44592300	0.74750400
C	-0.84645400	1.27016000	1.20858600
H	0.54778500	2.88622200	1.63343400
H	-0.62907100	3.23126100	0.33805600
H	-1.51931900	1.61446900	2.02538200
H	-0.22787000	0.46229400	1.64366100
O	-2.38649500	1.60831100	-0.61055200
H	2.73934500	-1.72431900	0.05863000
N	0.85613700	-1.25623000	-0.59883300
N	-1.85887800	-0.57711600	-0.16273400
C	-1.22677300	-1.58789800	0.68287400
H	-1.69543600	-2.56808000	0.45798900
H	-1.41337700	-1.40993700	1.76192900
C	0.29991500	-1.76983100	0.53597700
O	0.93099700	-2.36280700	1.41835000
H	0.23254500	-0.76325500	-1.24432700
C	-2.77560500	-1.04883700	-1.20377900
H	-3.10625700	-0.17440700	-1.79214200
H	-2.26913600	-1.77726300	-1.87031200
H	-3.66796300	-1.54176800	-0.75916000

Isomers $31a_{RO}$/$31a_{RC}$/$31a_{RE}$

Spartan Calculations

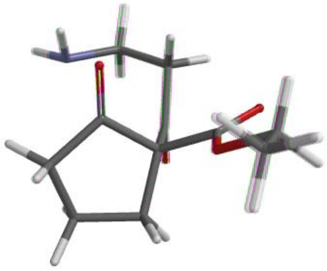

31_{RO}
$\Delta G°$ = 10.0 kcal/mol

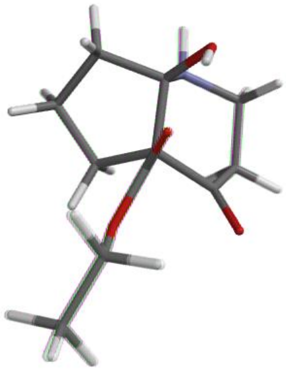

31_{RC}
$\Delta G°$ = 12.6 kcal/mol

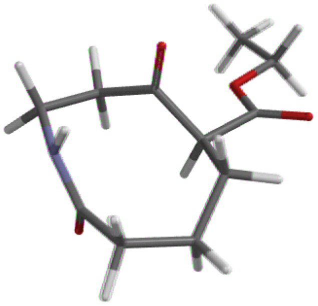

31_{RE}
$\Delta G°$ = 0.0 kcal/mol

31_{RO}	Calculated energy (au)	Calculated energy (kcal/mol)	Relative energy (kcal/mol)
M0010	-785.04963	-492618.6435	0.00
M0011	-785.04963	-492618.6435	0.00
M0015	-785.04824	-492617.7681	0.88
M0016	-785.04824	-492617.7681	0.88
M0018	-785.04780	-492617.4945	1.15
M0019	-785.04780	-492617.4945	1.15
M0020	-785.04772	-492617.4456	1.20
M0021	-785.04772	-492617.4456	1.20
M0031	-785.04756	-492617.3464	1.30
M0032	-785.04756	-492617.3464	1.30
M0023	-785.04740	-492617.2422	1.40
M0054	-785.04721	-492617.1262	1.52
M0043	-785.04716	-492617.0935	1.55
M0044	-785.04716	-492617.0935	1.55
M0055	-785.04703	-492617.0088	1.63
M0058	-785.04686	-492616.9065	1.74
M0060	-785.04683	-492616.8883	1.76
M0013	-785.04678	-492616.8526	1.79
M0014	-785.04678	-492616.8526	1.79
M0082	-785.04608	-492616.4133	2.23
M0079	-785.04605	-492616.3957	2.25
M0080	-785.04605	-492616.3957	2.25
M0003	-785.04598	-492616.3518	2.29
M0004	-785.04598	-492616.3518	2.29
M0040	-785.04578	-492616.2263	2.42
M0041	-785.04578	-492616.2263	2.42

M0062	-785.04572	-492616.1912	2.45
M0061	-785.04554	-492616.0757	2.57
M0048	-785.04551	-492616.0588	2.58
M0049	-785.04551	-492616.0588	2.58
M0005	-785.04478	-492615.6007	3.04
M0098	-785.04465	-492615.5147	3.13
M0001	-785.04434	-492615.3208	3.32
M0002	-785.04434	-492615.3208	3.32
M0008	-785.04420	-492615.2342	3.41
M0097	-785.04413	-492615.1941	3.45
M0100	-785.04406	-492615.1445	3.50
M0009	-785.04391	-492615.0529	3.59
M0006	-785.04388	-492615.0341	3.61
M0007	-785.04388	-492615.0341	3.61
M0085	-785.04374	-492614.9469	3.70
M0012	-785.04363	-492614.8797	3.76
M0087	-785.04315	-492614.5773	4.07
M0088	-785.04294	-492614.4430	4.20
M0092	-785.04254	-492614.1907	4.45
M0091	-785.04234	-492614.0684	4.58
M0093	-785.04227	-492614.0244	4.62
M0059	-785.04203	-492613.8732	4.77
M0074	-785.04189	-492613.7835	4.86
M0094	-785.04186	-492613.7690	4.87
M0024	-785.04177	-492613.7100	4.93
M0068	-785.04175	-492613.7000	4.94
M0069	-785.04175	-492613.7000	4.94
M0084	-785.04167	-492613.6479	5.00
M0029	-785.04163	-492613.6241	5.02
M0030	-785.04163	-492613.6241	5.02
M0064	-785.04157	-492613.5820	5.06
M0051	-785.04145	-492613.5099	5.13
M0072	-785.04142	-492613.4904	5.15
M0073	-785.04142	-492613.4904	5.15
M0045	-785.04141	-492613.4835	5.16
M0077	-785.04137	-492613.4603	5.18
M0078	-785.04137	-492613.4603	5.18
M0070	-785.04117	-492613.3329	5.31
M0071	-785.04116	-492613.3248	5.32
M0034	-785.04115	-492613.3229	5.32
M0090	-785.04114	-492613.3172	5.33
M0042	-785.04100	-492613.2281	5.42
M0086	-785.04099	-492613.2225	5.42
M0050	-785.04081	-492613.1076	5.54
M0065	-785.04063	-492612.9953	5.65
M0056	-785.04045	-492612.8830	5.76

M0057	-785.04045	-492612.8830	5.76
M0017	-785.04032	-492612.8021	5.84
M0027	-785.04020	-492612.7224	5.92
M0039	-785.04014	-492612.6872	5.96
M0025	-785.04010	-492612.6609	5.98
M0026	-785.04010	-492612.6609	5.98
M0053	-785.04004	-492612.6226	6.02
M0038	-785.03993	-492612.5536	6.09
M0083	-785.03987	-492612.5153	6.13
M0022	-785.03974	-492612.4362	6.21
M0046	-785.03959	-492612.3421	6.30
M0033	-785.03956	-492612.3264	6.32
M0036	-785.03955	-492612.3183	6.33
M0037	-785.03955	-492612.3183	6.33
M0047	-785.03949	-492612.2775	6.37
M0035	-785.03934	-492612.1877	6.46
M0028	-785.03885	-492611.8752	6.77
M0075	-785.03884	-492611.8734	6.77
M0052	-785.03871	-492611.7912	6.85
M0076	-785.03864	-492611.7441	6.90
M0063	-785.03862	-492611.7322	6.91
M0067	-785.03858	-492611.7077	6.94
M0081	-785.03795	-492611.3149	7.33
M0066	-785.03787	-492611.2659	7.38
M0095	-785.03684	-492610.6146	8.03
M0096	-785.03684	-492610.6146	8.03
M0089	-785.03549	-492609.7675	8.88
M0099	-785.03321	-492608.3386	10.30
**31_{RC}**	**Calculated energy (au)**	**Calculated energy (kcal/mol)**	**Relative energy (kcal/mol)**
M0001	-785.04830	-492617.8070	0.00
M0003	-785.04812	-492617.6959	0.11
M0002	-785.04761	-492617.3778	0.43
M0018	-785.04753	-492617.3219	0.49
M0006	-785.04713	-492617.0747	0.73
M0012	-785.04694	-492616.9523	0.85
M0025	-785.04688	-492616.9147	0.89
M0010	-785.04688	-492616.9141	0.89
M0024	-785.04661	-492616.7478	1.06
M0047	-785.04464	-492615.5122	2.29
M0056	-785.04445	-492615.3936	2.41
M0007	-785.04440	-492615.3629	2.44
M0017	-785.04418	-492615.2255	2.58
M0026	-785.04394	-492615.0705	2.74
M0055	-785.04391	-492615.0529	2.75
M0015	-785.04391	-492615.0523	2.75
M0030	-785.04375	-492614.9506	2.86

M0013	-785.04355	-492614.8245	2.98
M0021	-785.04296	-492614.4593	3.35
M0038	-785.04287	-492614.3990	3.41
M0042	-785.04279	-492614.3526	3.45
M0036	-785.04267	-492614.2723	3.53
M0060	-785.04245	-492614.1355	3.67
M0045	-785.04240	-492614.1060	3.70
M0057	-785.04215	-492613.9460	3.86
M0041	-785.04206	-492613.8945	3.91
M0048	-785.04161	-492613.6096	4.20
M0005	-785.04152	-492613.5538	4.25
M0062	-785.04136	-492613.4540	4.35
M0037	-785.04126	-492613.3913	4.42
M0064	-785.04124	-492613.3762	4.43
M0016	-785.04123	-492613.3687	4.44
M0019	-785.04099	-492613.2187	4.59
M0008	-785.04087	-492613.1459	4.66
M0063	-785.04066	-492613.0167	4.79
M0027	-785.04045	-492612.8817	4.93
M0023	-785.04037	-492612.8347	4.97
M0014	-785.03999	-492612.5944	5.21
M0049	-785.03986	-492612.5103	5.30
M0020	-785.03962	-492612.3609	5.45
M0058	-785.03958	-492612.3390	5.47
M0028	-785.03897	-492611.9512	5.86
M0054	-785.03885	-492611.8803	5.93
M0032	-785.03881	-492611.8514	5.96
M0004	-785.03851	-492611.6625	6.14
M0065	-785.03840	-492611.5966	6.21
M0066	-785.03824	-492611.4962	6.31
M0067	-785.03814	-492611.4329	6.37
M0009	-785.03800	-492611.3450	6.46
M0011	-785.03800	-492611.3437	6.46
M0033	-785.03747	-492611.0124	6.79
M0022	-785.03709	-492610.7721	7.03
M0029	-785.03692	-492610.6667	7.14
M0031	-785.03674	-492610.5569	7.25
M0034	-785.03672	-492610.5418	7.27
M0043	-785.03643	-492610.3592	7.45
M0039	-785.03639	-492610.3316	7.48
M0059	-785.03575	-492609.9344	7.87
M0051	-785.03568	-492609.8898	7.92
M0061	-785.03555	-492609.8095	8.00
M0068	-785.03512	-492609.5365	8.27
M0046	-785.03501	-492609.4700	8.34
M0050	-785.03499	-492609.4550	8.35

M0035	-785.03460	-492609.2134	8.59
M0053	-785.03456	-492609.1870	8.62
M0040	-785.03438	-492609.0709	8.74
M0044	-785.03434	-492609.0496	8.76
M0052	-785.03324	-492608.3581	9.45
**31_{RE}**	**Calculated energy (au)**	**Calculated energy (kcal/mol)**	**Relative energy (kcal/mol)**
M0003	-785.07315	-492633.4029	0.00
M0021	-785.07298	-492633.2943	0.11
M0022	-785.07298	-492633.2943	0.11
M0023	-785.07294	-492633.2692	0.13
M0025	-785.07293	-492633.2642	0.14
M0009	-785.07292	-492633.2573	0.15
M0010	-785.07271	-492633.1236	0.28
M0029	-785.07259	-492633.0496	0.35
M0013	-785.07258	-492633.0421	0.36
M0014	-785.07250	-492632.9931	0.41
M0028	-785.07248	-492632.9806	0.42
M0015	-785.07247	-492632.9756	0.43
M0016	-785.07247	-492632.9756	0.43
M0002	-785.07236	-492632.9046	0.50
M0026	-785.07235	-492632.8965	0.51
M0008	-785.07218	-492632.7898	0.61
M0005	-785.07209	-492632.7377	0.67
M0017	-785.07060	-492631.8028	1.60
M0018	-785.07060	-492631.8028	1.60
M0004	-785.07059	-492631.7927	1.61
M0011	-785.07048	-492631.7262	1.68
M0019	-785.07044	-492631.7011	1.70
M0020	-785.07044	-492631.7011	1.70
M0012	-785.07024	-492631.5769	1.83
M0032	-785.07012	-492631.5022	1.90
M0033	-785.07008	-492631.4752	1.93
M0034	-785.07008	-492631.4752	1.93
M0024	-785.07004	-492631.4476	1.96
M0001	-785.06968	-492631.2267	2.18
M0035	-785.06967	-492631.2204	2.18
M0036	-785.06967	-492631.2204	2.18
M0006	-785.06951	-492631.1175	2.29
M0007	-785.06933	-492631.0014	2.40
M0037	-785.06728	-492629.7182	3.68
M0045	-785.06712	-492629.6184	3.78
M0057	-785.06706	-492629.5820	3.82
M0041	-785.06692	-492629.4917	3.91
M0063	-785.06674	-492629.3775	4.03
M0027	-785.06666	-492629.3260	4.08
M0065	-785.06660	-492629.2902	4.11

M0066	-785.06660	-492629.2902	4.11
M0051	-785.06655	-492629.2614	4.14
M0031	-785.06638	-492629.1541	4.25
M0038	-785.06629	-492629.0982	4.30
M0039	-785.06629	-492629.0982	4.30
M0056	-785.06628	-492629.0926	4.31
M0053	-785.06627	-492629.0869	4.32
M0030	-785.06624	-492629.0637	4.34
M0046	-785.06612	-492628.9897	4.41
M0064	-785.06608	-492628.9677	4.44
M0052	-785.06605	-492628.9451	4.46
M0084	-785.06592	-492628.8661	4.54
M0044	-785.06588	-492628.8403	4.56
M0088	-785.06587	-492628.8334	4.57
M0043	-785.06581	-492628.7945	4.61
M0049	-785.06572	-492628.7418	4.66
M0067	-785.06572	-492628.7406	4.66
M0048	-785.06556	-492628.6376	4.77
M0090	-785.06513	-492628.3666	5.04
M0075	-785.06433	-492627.8639	5.54
M0060	-785.06426	-492627.8257	5.58
M0083	-785.06421	-492627.7924	5.61
M0050	-785.06420	-492627.7868	5.62
M0086	-785.06417	-492627.7660	5.64
M0077	-785.06414	-492627.7479	5.66
M0081	-785.06409	-492627.7177	5.69
M0073	-785.06403	-492627.6763	5.73
M0089	-785.06395	-492627.6274	5.78
M0094	-785.06384	-492627.5596	5.84
M0085	-785.06369	-492627.4648	5.94
M0095	-785.06361	-492627.4146	5.99
M0087	-785.06344	-492627.3105	6.09
M0069	-785.06339	-492627.2753	6.13
M0070	-785.06339	-492627.2753	6.13
M0078	-785.06318	-492627.1429	6.26
M0054	-785.06312	-492627.1072	6.30
M0097	-785.06296	-492627.0080	6.39
M0071	-785.06295	-492627.0018	6.40
M0076	-785.06288	-492626.9591	6.44
M0099	-785.06288	-492626.9591	6.44
M0059	-785.06284	-492626.9315	6.47
M0079	-785.06276	-492626.8844	6.52
M0080	-785.06276	-492626.8838	6.52
M0082	-785.06253	-492626.7369	6.67
M0096	-785.06251	-492626.7275	6.68
M0100	-785.06249	-492626.7131	6.69

M0040	-785.06215	-492626.4972	6.91
M0091	-785.06206	-492626.4439	6.96
M0047	-785.06198	-492626.3950	7.01
M0055	-785.06148	-492626.0762	7.33
M0061	-785.06142	-492626.0417	7.36
M0093	-785.06122	-492625.9149	7.49
M0042	-785.06112	-492625.8528	7.55
M0058	-785.06090	-492625.7129	7.69
M0062	-785.06086	-492625.6878	7.72
M0098	-785.06000	-492625.1475	8.26
M0068	-785.05981	-492625.0295	8.37
M0074	-785.05960	-492624.8959	8.51
M0072	-785.05920	-492624.6455	8.76
M0092	-785.05829	-492624.0751	9.33

Table S10. Energies calculated at DFT/B3LYP/6-31G* in vacuum for the conformers found for $\mathbf{31_{RO}/31_{RC}/31_{RE}}$.

Isomer	ΔG° (au)	ΔG° (kcal/mol)	ΔG° (kcal/mol)
$\mathbf{31_{RO}}$	-784.828711	-492480.02	10.0
$\mathbf{31_{RC}}$	-784.82455	-492477.41	12.6
$\mathbf{31_{RE}}$	-784.84467	-492490.03	0.0

Table S11. Relative free energies (ΔG°) of the lowest energy geometries of $\mathbf{31_{RO}/31_{RC}/31_{RE}}$ at DFT/B3LYP/6-31G* in vacuum in kcal/mol.

XYZ Coordinates

31_{RO}

C	-0.25592900	0.91794100	-0.88193100
C	-0.55927000	2.38816100	-0.63507100
C	-0.71590000	2.52234100	0.88650900
C	0.17441100	1.39963100	1.46498900
C	-0.01892900	0.21327100	0.49135900
H	-1.43073000	2.68913000	-1.22266100
H	0.30251000	2.95870100	-1.00835100
H	-0.42245000	3.50811100	1.25817900
H	-1.75780000	2.35238000	1.17569900
H	-0.10666900	1.12188100	2.48256900
H	1.22485100	1.70627100	1.45719900
O	-0.18542900	0.36879100	-1.96109100
C	-1.28087900	-0.63246000	0.87141900
O	-1.84944900	-0.42798000	1.92429900
C	1.17832100	-0.72948900	0.41794900
O	1.14562100	-1.91329900	0.69012900
O	2.28907100	-0.08156900	0.02289900
C	3.49383100	-0.88298800	-0.11908100
H	3.25887100	-1.74643800	-0.74737100
C	-1.78135900	-1.68823000	-0.10284100
H	-1.12056900	-1.77243900	-0.96823100
H	-1.76238800	-2.64667000	0.42644900
C	-3.21669900	-1.36989000	-0.54718100
H	-3.85106900	-1.28804000	0.34087900
H	-3.59328800	-2.21164000	-1.15011100
N	-3.25170900	-0.07715000	-1.24815100
H	-4.20728900	0.18906000	-1.46850100
H	-2.73580900	-0.12461000	-2.12414100
H	3.78003100	-1.25148800	0.87082900
C	4.56130100	0.00083200	-0.73369100
H	4.77400100	0.86652200	-0.09883100
H	4.25136100	0.35997200	-1.71947100
H	5.48648100	-0.57291800	-0.85158100

31_{RC}

C	1.42024000	-0.71729100	-0.38574100
C	1.21047000	-2.11833100	0.20690900
C	0.74739000	-1.87115100	1.63993900
C	0.18701000	-0.44211200	1.67087900
C	0.22810000	0.07061800	0.21081900
H	2.10947000	-2.74478100	0.16715900
H	0.44327000	-2.66795100	-0.35301100
H	-0.00879000	-2.60544200	1.93650900
H	1.58473000	-1.96039100	2.34121900
H	0.80376000	0.17286900	2.33682900
H	-0.81637000	-0.44239200	2.10910900
C	0.50951000	1.56168900	0.14712900
O	-0.37906000	2.38994800	-0.06925100
C	-1.06864000	-0.27004200	-0.51184100
O	-1.11638000	-0.82060200	-1.60849100
O	-2.16623000	0.10242800	0.20168900
C	-3.41787000	-0.12654200	-0.45665100
H	-3.54538000	-1.19879200	-0.64221100
C	1.90544000	2.03498900	0.46967900
H	2.01191000	3.07638900	0.14700900
H	-3.44174000	0.41895800	-1.40662100
C	-4.52603000	0.37246800	0.44952900
H	-4.39887000	1.43911800	0.66219900
H	-4.50103000	-0.14911200	1.41205900
H	-5.50590000	0.21818800	-0.01076100
N	2.72404000	-0.23657100	0.07910900
O	1.48812000	-0.78109100	-1.79138100
H	0.60903000	-1.05879100	-2.12654100
H	2.04618000	2.00307900	1.55513900
C	2.92225000	1.17349900	-0.24831100
H	3.42360000	-0.78079100	-0.43040100
H	2.87011000	1.33349900	-1.33218100
H	3.92892000	1.47011900	0.06612900

31$_{RE}$

C	3.08331000	1.34227200	-0.19947000
C	1.78118900	2.14390200	-0.07013000
C	0.70393900	1.51747100	0.84146000
C	-0.21459000	0.50464100	0.10917000
H	3.75813900	1.82692200	-0.91384000
H	3.60792000	1.32055200	0.76278000
H	2.04683900	3.12533200	0.34318000
H	1.36006900	2.33633100	-1.06449000
H	0.10730900	2.34463100	1.24379000
H	1.19246000	1.06451100	1.71265000
C	-0.07901900	-0.88078900	0.73327000
O	-0.36686900	-1.09288900	1.91325000
C	-1.66526000	0.94522000	0.07480000
O	-2.05903100	2.08988000	0.25579000
O	-2.47026000	-0.09592000	-0.28714000
C	-3.86309000	0.23257900	-0.36784000
H	-4.01724000	0.99638900	-1.13808000
C	0.49579100	-1.98485900	-0.13285000
H	-0.07959900	-2.90184900	0.03455000
H	-4.21242000	0.60719900	0.60082000
C	-4.62389900	-1.02643100	-0.73489000
H	-4.46076900	-1.80760100	0.01499000
H	-4.27071900	-1.42510100	-1.69167000
H	-5.69642900	-0.82657200	-0.80956000
N	2.70694100	-0.97468800	0.34910000
H	0.35483100	-1.72488900	-1.18722000
C	1.97151100	-2.21230800	0.18172000
H	0.07535000	0.44247100	-0.94595000
C	2.81838000	-0.06423800	-0.67859000
O	2.57704000	-0.33034800	-1.85003000
H	2.86970000	-0.66140800	1.29994000
H	2.07874100	-2.77017800	1.11811000
H	2.44062100	-2.79445800	-0.61860000

Gaussian Calculations

31$_{RO}$

M06-2X/6-31G* with Solvent Correction

SCF Done: E(RM062X) = -784.743510316

Zero-point correction= 0.281591
Thermal correction to Gibbs Free Energy= 0.237406

C	-0.29960300	0.89942200	-0.86008700
C	-0.69434000	2.33114200	-0.55314900
C	-0.88281400	2.36054500	0.96465100
C	0.11735500	1.31083300	1.48056000
C	-0.03051000	0.15927600	0.47675400
H	-1.57250600	2.60936900	-1.13930700
H	0.14249700	2.96949000	-0.86486400
H	-0.70773800	3.34727500	1.39725800
H	-1.90064000	2.04735900	1.21715500
H	-0.09629100	0.97933600	2.49738200
H	1.13782900	1.70323200	1.43658700
O	-0.16447500	0.39911700	-1.95086000
C	-1.23362700	-0.74675800	0.83064800
O	-1.76634000	-0.65525600	1.91217100
C	1.18586700	-0.73430900	0.32676700
O	1.17655600	-1.93541900	0.47421800
O	2.27018900	-0.02978600	0.00574400
C	3.48401500	-0.78680500	-0.17671300
H	3.31438200	-1.52548900	-0.96440600
C	-1.74187000	-1.71588800	-0.21755700
H	-1.08145200	-1.73235700	-1.08783600
H	-1.76026200	-2.71685000	0.22314000
C	-3.15390200	-1.27590100	-0.60888300
H	-3.78191200	-1.30300500	0.28633600
H	-3.57570500	-1.98256200	-1.33656800
N	-3.10437500	0.11180200	-1.08309100
H	-4.05036900	0.46256000	-1.21660000
H	-2.65847800	0.13939800	-1.99924300
H	3.70160200	-1.32108700	0.75188100
C	4.57317100	0.19710900	-0.53928800
H	4.71189900	0.93022700	0.25918900
H	4.32277500	0.72561700	-1.46233800
H	5.51510000	-0.33636300	-0.68853200

31_{RC}

M06-2X/6-31G* with Solvent Correction

SCF Done: E(RM062X) = -784.751531934

Zero-point correction= 0.284257
Thermal correction to Gibbs Free Energy= 0.241812

C	1.43744900	-0.71787600	-0.44589100
C	1.15404500	-2.17274900	-0.05182300
C	0.57560900	-2.09788700	1.37647700
C	0.20667800	-0.60913100	1.62514300
C	0.24455300	0.04590000	0.22584700
H	2.06215500	-2.77844400	-0.12306600
H	0.42539100	-2.58507000	-0.75694300
H	-0.30608000	-2.73677500	1.47277200
H	1.30219800	-2.43374100	2.11899800
H	0.95872600	-0.13954100	2.25938000
H	-0.76729800	-0.48184400	2.09788200
C	0.50755500	1.55140600	0.21936600
O	-0.31627900	2.33787700	-0.19881800
C	-1.06241300	-0.18910000	-0.52603000
O	-1.14709000	-0.55196800	-1.68478200
O	-2.12382000	0.06601500	0.22485300
C	-3.40549200	-0.03054800	-0.42905200
H	-3.52707000	-1.04972100	-0.80502400
C	1.87975500	1.97387500	0.69225900
H	2.00113800	3.03829900	0.48149900
H	-3.40593500	0.65194000	-1.28296200
C	-4.45622300	0.33206500	0.59562300
H	-4.30017600	1.34932900	0.96260700
H	-4.42256700	-0.35669500	1.44346800
H	-5.44803000	0.27450900	0.14056400
N	2.65272500	-0.28540100	0.20938600
O	1.54892100	-0.49145500	-1.83129900
H	0.66154200	-0.63116300	-2.21064200
H	1.94978100	1.81871800	1.77462700
C	2.95400200	1.12977000	-0.00518900
H	3.41691500	-0.85887200	-0.14335600
H	2.99277900	1.39354600	-1.07020400
H	3.93050200	1.34522900	0.43469900

31RE

M06-2X/6-31G* with Solvent Correction

SCF Done: E(RM062X) = -784.765316785

Zero-point correction= 0.283611
Thermal correction to Gibbs Free Energy= 0.239284

C	3.13616000	1.15822700	-0.28188300
C	1.94082000	2.12820200	-0.30821600
C	0.75984600	1.74674100	0.59803500
C	-0.14513200	0.63107900	0.03684400
H	3.88314100	1.47930000	-1.01271200
H	3.60233600	1.16368800	0.70883500
H	2.30647800	3.11550300	-0.00967800
H	1.58800300	2.22017200	-1.34191500
H	0.13494700	2.63231100	0.74044000
H	1.11405400	1.45529200	1.59426300
C	-0.03299800	-0.70057500	0.80156400
O	-0.13975700	-0.71549000	2.00879700
C	-1.62298300	1.00630800	0.07957700
O	-2.08328700	2.07173800	0.40719200
O	-2.36604100	-0.03681400	-0.31192100
C	-3.79277000	0.16530900	-0.31881300
H	-4.02257200	0.99117800	-0.99742600
C	0.27601300	-1.96299100	0.01686600
H	-0.36079600	-2.77521400	0.37695100
H	-4.10717700	0.45337700	0.68781700
C	-4.42536700	-1.13362000	-0.76518000
H	-4.16726700	-1.94304500	-0.07770800
H	-4.08554900	-1.40318700	-1.76819700
H	-5.51271700	-1.02656500	-0.78347500
N	2.58612900	-1.11937700	0.36876700
H	0.09615200	-1.80749900	-1.04729000
C	1.75974000	-2.31652100	0.26614700
H	0.08344300	0.44691500	-1.01864800
C	2.66198000	-0.23033000	-0.65401500
O	2.26333100	-0.50585000	-1.78183100
H	2.86567500	-0.81827700	1.29235600
H	1.86098700	-2.85344500	1.20974200
H	2.13212800	-2.95450900	-0.53818300

Isomer $32_{RO}/32_{RC}/32_{RE}$

Spartan Calculations

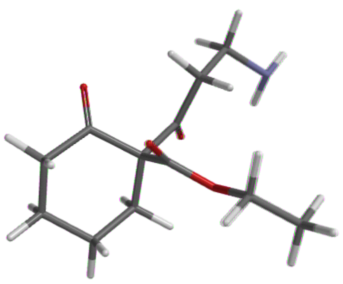

32_{RO}
$\Delta G° = 8.1$ kcal/mol

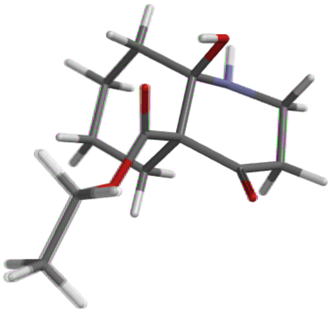

32_{RC}
$\Delta G° = 9.7$ kcal/mol

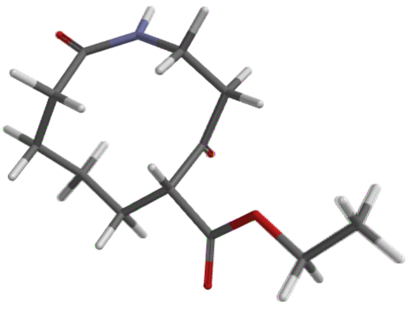

32_{RE}
$\Delta G° = 0.0$ kcal/mol

32_{RO}	Calculated energy (au)	Calculated energy (kcal/mol)	Relative energy (kcal/mol)
M0008	-824.36596	-517289.6412	0.00
M0009	-824.36596	-517289.6412	0.00
M0006	-824.36547	-517289.3343	0.31
M0007	-824.36547	-517289.3343	0.31
M0042	-824.36477	-517288.8957	0.75
M0068	-824.36470	-517288.8467	0.79
M0040	-824.36450	-517288.7212	0.92
M0061	-824.36441	-517288.6654	0.98
M0062	-824.36441	-517288.6654	0.98
M0001	-824.36432	-517288.6089	1.03
M0002	-824.36432	-517288.6089	1.03
M0039	-824.36426	-517288.5719	1.07
M0096	-824.36421	-517288.5393	1.10
M0050	-824.36414	-517288.4985	1.14
M0027	-824.36410	-517288.4696	1.17
M0093	-824.36383	-517288.3058	1.34
M0017	-824.36360	-517288.1584	1.48
M0060	-824.36359	-517288.1502	1.49
M0010	-824.36348	-517288.0818	1.56
M0011	-824.36348	-517288.0818	1.56
M0058	-824.36346	-517288.0730	1.57
M0033	-824.36316	-517287.8854	1.76
M0034	-824.36316	-517287.8854	1.76
M0037	-824.36311	-517287.8522	1.79
M0003	-824.36293	-517287.7379	1.90
M0064	-824.36279	-517287.6514	1.99
M0059	-824.36270	-517287.5917	2.05
M0087	-824.36260	-517287.5321	2.11
M0044	-824.36252	-517287.4800	2.16

M0046	-824.36249	-517287.4650	2.18
M0047	-824.36249	-517287.4650	2.18
M0051	-824.36237	-517287.3891	2.25
M0056	-824.36236	-517287.3809	2.26
M0063	-824.36232	-517287.3545	2.29
M0089	-824.36225	-517287.3125	2.33
M0018	-824.36209	-517287.2102	2.43
M0004	-824.36207	-517287.2014	2.44
M0005	-824.36207	-517287.2014	2.44
M0054	-824.36202	-517287.1676	2.47
M0029	-824.36201	-517287.1613	2.48
M0038	-824.36195	-517287.1243	2.52
M0036	-824.36191	-517287.0954	2.55
M0041	-824.36186	-517287.0659	2.58
M0088	-824.36182	-517287.0421	2.60
M0016	-824.36180	-517287.0308	2.61
M0022	-824.36175	-517286.9969	2.64
M0028	-824.36173	-517286.9849	2.66
M0049	-824.36171	-517286.9755	2.67
M0084	-824.36150	-517286.8400	2.80
M0052	-824.36140	-517286.7810	2.86
M0053	-824.36140	-517286.7810	2.86
M0024	-824.36121	-517286.6605	2.98
M0014	-824.36120	-517286.6517	2.99
M0025	-824.36104	-517286.5539	3.09
M0078	-824.36092	-517286.4754	3.17
M0035	-824.36091	-517286.4717	3.17
M0091	-824.36079	-517286.3932	3.25
M0031	-824.36070	-517286.3405	3.30
M0030	-824.36069	-517286.3336	3.31
M0074	-824.36035	-517286.1171	3.52
M0075	-824.36023	-517286.0431	3.60
M0012	-824.36022	-517286.0399	3.60
M0086	-824.35990	-517285.8385	3.80
M0019	-824.35978	-517285.7607	3.88
M0020	-824.35978	-517285.7607	3.88
M0057	-824.35969	-517285.7030	3.94
M0083	-824.35963	-517285.6703	3.97
M0067	-824.35935	-517285.4896	4.15
M0069	-824.35915	-517285.3635	4.28
M0055	-824.35875	-517285.1156	4.53
M0015	-824.35857	-517285.0020	4.64
M0090	-824.35799	-517284.6394	5.00
M0065	-824.35784	-517284.5465	5.09
M0026	-824.35764	-517284.4178	5.22
M0023	-824.35763	-517284.4103	5.23

M0066	-824.35756	-517284.3664	5.27
M0048	-824.35748	-517284.3156	5.33
M0076	-824.35738	-517284.2578	5.38
M0077	-824.35738	-517284.2578	5.38
M0070	-824.35732	-517284.2183	5.42
M0099	-824.35727	-517284.1857	5.46
M0072	-824.35714	-517284.1066	5.53
M0079	-824.35709	-517284.0752	5.57
M0080	-824.35709	-517284.0752	5.57
M0085	-824.35669	-517283.8217	5.82
M0045	-824.35656	-517283.7439	5.90
M0021	-824.35644	-517283.6648	5.98
M0032	-824.35615	-517283.4848	6.16
M0097	-824.35601	-517283.3988	6.24
M0043	-824.35587	-517283.3091	6.33
M0098	-824.35582	-517283.2739	6.37
M0092	-824.35566	-517283.1785	6.46
M0094	-824.35509	-517282.8202	6.82
M0071	-824.35483	-517282.6539	6.99
M0082	-824.35440	-517282.3835	7.26
M0073	-824.35396	-517282.1124	7.53
M0081	-824.35333	-517281.7133	7.93
M0100	-824.35296	-517281.4830	8.16
M0095	-824.35193	-517280.8361	8.81
**32_{RC}**	**Calculated energy (au)**	**Calculated energy (kcal/mol)**	**Relative energy (kcal/mol)**
M0001	-824.37207	-517293.4708	0.00
M0003	-824.37177	-517293.2876	0.18
M0002	-824.37127	-517292.9738	0.50
M0010	-824.37010	-517292.2346	1.24
M0011	-824.36963	-517291.9409	1.53
M0015	-824.36783	-517290.8152	2.66
M0008	-824.36727	-517290.4600	3.01
M0004	-824.36717	-517290.4017	3.07
M0021	-824.36708	-517290.3446	3.13
M0012	-824.36623	-517289.8068	3.66
M0006	-824.36616	-517289.7679	3.70
M0016	-824.36564	-517289.4385	4.03
M0018	-824.36561	-517289.4178	4.05
M0005	-824.36533	-517289.2421	4.23
M0009	-824.36505	-517289.0708	4.40
M0037	-824.36469	-517288.8411	4.63
M0007	-824.36449	-517288.7143	4.76
M0041	-824.36432	-517288.6102	4.86
M0039	-824.36363	-517288.1803	5.29
M0042	-824.36319	-517287.9030	5.57
M0051	-824.36180	-517287.0282	6.44

M0058	-824.36122	-517286.6662	6.80
M0055	-824.36105	-517286.5608	6.91
M0036	-824.36029	-517286.0820	7.39
M0031	-824.36023	-517286.0443	7.43
M0054	-824.35941	-517285.5273	7.94
M0028	-824.35926	-517285.4350	8.04
M0032	-824.35906	-517285.3070	8.16
M0060	-824.35897	-517285.2512	8.22
M0019	-824.35854	-517284.9813	8.49
M0029	-824.35851	-517284.9644	8.51
M0059	-824.35835	-517284.8665	8.60
M0025	-824.35817	-517284.7498	8.72
M0024	-824.35791	-517284.5904	8.88
M0014	-824.35764	-517284.4160	9.05
M0020	-824.35737	-517284.2497	9.22
M0023	-824.35727	-517284.1894	9.28
M0017	-824.35683	-517283.9115	9.56
M0013	-824.35659	-517283.7583	9.71
M0046	-824.35658	-517283.7546	9.72
M0049	-824.35640	-517283.6404	9.83
M0057	-824.35640	-517283.6391	9.83
M0030	-824.35631	-517283.5814	9.89
M0033	-824.35630	-517283.5776	9.89
M0022	-824.35610	-517283.4540	10.02
M0026	-824.35573	-517283.2181	10.25
M0034	-824.35557	-517283.1195	10.35
M0048	-824.35553	-517283.0951	10.38
M0056	-824.35552	-517283.0913	10.38
M0027	-824.35538	-517282.9978	10.47
M0061	-824.35504	-517282.7901	10.68
M0035	-824.35365	-517281.9173	11.55
M0038	-824.35329	-517281.6863	11.78
M0040	-824.35320	-517281.6349	11.84
M0047	-824.35269	-517281.3111	12.16
M0050	-824.35237	-517281.1147	12.36
M0043	-824.35172	-517280.7024	12.77
M0045	-824.35154	-517280.5895	12.88
M0052	-824.35141	-517280.5123	12.96
M0044	-824.35136	-517280.4759	12.99
**32_{RE}**	**Calculated energy (au)**	**Calculated energy (kcal/mol)**	**Relative energy (kcal/mol)**
M0040	-824.38468	-517301.3854	0.00
M0027	-824.38409	-517301.0177	0.37
M0032	-824.38394	-517300.9249	0.46
M0013	-824.38386	-517300.8740	0.51
M0014	-824.38385	-517300.8671	0.52
M0033	-824.38354	-517300.6695	0.72

M0011	-824.38328	-517300.5101	0.88
M0017	-824.38318	-517300.4429	0.94
M0015	-824.38315	-517300.4235	0.96
M0020	-824.38308	-517300.3846	1.00
M0001	-824.38304	-517300.3576	1.03
M0004	-824.38290	-517300.2723	1.11
M0002	-824.38282	-517300.2202	1.17
M0003	-824.38253	-517300.0382	1.35
M0006	-824.38251	-517300.0219	1.36
M0005	-824.38234	-517299.9209	1.46
M0034	-824.38209	-517299.7583	1.63
M0036	-824.38174	-517299.5412	1.84
M0025	-824.38113	-517299.1572	2.23
M0030	-824.38099	-517299.0725	2.31
M0019	-824.38060	-517298.8265	2.56
M0029	-824.38053	-517298.7794	2.61
M0021	-824.38050	-517298.7619	2.62
M0009	-824.38040	-517298.7016	2.68
M0023	-824.38035	-517298.6671	2.72
M0031	-824.38022	-517298.5849	2.80
M0022	-824.38005	-517298.4826	2.90
M0010	-824.37996	-517298.4230	2.96
M0007	-824.37991	-517298.3935	2.99
M0026	-824.37984	-517298.3483	3.04
M0028	-824.37981	-517298.3308	3.05
M0056	-824.37959	-517298.1927	3.19
M0066	-824.37946	-517298.1130	3.27
M0018	-824.37945	-517298.1055	3.28
M0052	-824.37928	-517297.9976	3.39
M0068	-824.37921	-517297.9530	3.43
M0057	-824.37901	-517297.8313	3.55
M0058	-824.37886	-517297.7321	3.65
M0041	-824.37880	-517297.6939	3.69
M0065	-824.37866	-517297.6098	3.78
M0043	-824.37865	-517297.6010	3.78
M0045	-824.37842	-517297.4611	3.92
M0044	-824.37807	-517297.2358	4.15
M0048	-824.37802	-517297.2044	4.18
M0049	-824.37770	-517297.0074	4.38
M0035	-824.37767	-517296.9879	4.40
M0008	-824.37759	-517296.9390	4.45
M0016	-824.37754	-517296.9089	4.48
M0072	-824.37750	-517296.8831	4.50
M0047	-824.37737	-517296.7990	4.59
M0012	-824.37727	-517296.7338	4.65
M0087	-824.37725	-517296.7269	4.66

M0050	-824.37720	-517296.6930	4.69
M0071	-824.37719	-517296.6892	4.70
M0051	-824.37712	-517296.6447	4.74
M0070	-824.37708	-517296.6177	4.77
M0042	-824.37631	-517296.1352	5.25
M0037	-824.37618	-517296.0536	5.33
M0024	-824.37608	-517295.9927	5.39
M0046	-824.37585	-517295.8446	5.54
M0079	-824.37556	-517295.6614	5.72
M0062	-824.37549	-517295.6206	5.76
M0053	-824.37511	-517295.3840	6.00
M0084	-824.37499	-517295.3050	6.08
M0063	-824.37481	-517295.1945	6.19
M0055	-824.37476	-517295.1644	6.22
M0082	-824.37448	-517294.9862	6.40
M0038	-824.37391	-517294.6292	6.76
M0076	-824.37391	-517294.6273	6.76
M0064	-824.37390	-517294.6229	6.76
M0039	-824.37356	-517294.4108	6.97
M0077	-824.37325	-517294.2125	7.17
M0054	-824.37317	-517294.1648	7.22
M0060	-824.37304	-517294.0795	7.31
M0059	-824.37288	-517293.9835	7.40
M0061	-824.37249	-517293.7362	7.65
M0080	-824.37245	-517293.7149	7.67
M0092	-824.37205	-517293.4582	7.93
M0097	-824.37168	-517293.2292	8.16
M0075	-824.37136	-517293.0271	8.36
M0069	-824.37133	-517293.0077	8.38
M0088	-824.37126	-517292.9638	8.42
M0089	-824.37100	-517292.8044	8.58
M0086	-824.37096	-517292.7787	8.61
M0093	-824.37078	-517292.6638	8.72
M0094	-824.37065	-517292.5848	8.80
M0095	-824.37063	-517292.5710	8.81
M0081	-824.36956	-517291.9014	9.48
M0085	-824.36890	-517291.4822	9.90
M0067	-824.36880	-517291.4195	9.97
M0073	-824.36850	-517291.2331	10.15
M0078	-824.36719	-517290.4130	10.97
M0091	-824.36692	-517290.2398	11.15
M0083	-824.36665	-517290.0710	11.31
M0099	-824.36654	-517290.0032	11.38
M0074	-824.36642	-517289.9279	11.46
M0098	-824.36632	-517289.8658	11.52
M0100	-824.36593	-517289.6236	11.76

M0096	-824.36567	-517289.4604	11.93
M0090	-824.36483	-517288.9296	12.46

Table S12. Energies calculated at DFT/B3LYP/6-31G* in vacuum for the conformers found for **32_{RO}/32_{RC}/32_{RE}**.

Isomer	ΔG° (au)	ΔG° (kcal/mol)	ΔG° (kcal/mol)
**32_{RO}**	-824.117432	-517133.69	8.1
**32_{RC}**	-824.114985	-517132.15	9.7
**32_{RE}**	-824.130391	-517141.82	0.0

Table S13. Relative free energies (ΔG°) of the lowest energy geometries of **32_{RO}/32_{RC}/32_{RE}** at DFT/B3LYP/6-31G* in vacuum in kcal/mol.

XYZ Coordinates

32$_{RO}$

C	-1.44446000	-1.30068800	-0.66720700
C	-0.53856100	-0.34072500	0.15959200
O	-1.00643000	-2.33851200	-1.12813900
C	0.83942300	-1.03939800	0.41100900
O	1.07043000	-1.53382600	1.49803600
C	-0.31587500	0.94831100	-0.64099400
O	-0.69706000	1.14230800	-1.77925900
O	0.38281800	1.84524400	0.07612700
C	0.71744400	3.09881400	-0.58485100
H	-0.21493300	3.59743700	-0.86610100
C	1.83235200	-1.08580200	-0.73419100
H	1.30654800	-1.49664200	-1.60398200
H	2.11594700	-0.05473400	-0.98410600
C	3.09358000	-1.89555500	-0.43075400
H	3.67702200	-1.96627700	-1.35503600
H	2.80139100	-2.91933800	-0.15012900
H	1.26689800	2.86662700	-1.50196000
C	1.54087000	3.92036100	0.38656000
H	2.46179800	3.39801000	0.66169700
H	0.97669000	4.13559000	1.29899800
H	1.81209800	4.87280800	-0.08107700
C	-1.24350400	-0.03928800	1.51297000
H	-1.24272400	-0.96710900	2.09358400
H	-0.63594200	0.67827100	2.07007200
C	-2.67870200	0.46937000	1.32929100
H	-3.11946600	0.66123600	2.31458200
H	-2.67738000	1.43197400	0.79867000
C	-3.53328900	-0.54629200	0.56100500
H	-3.63589900	-1.46067500	1.16022000
H	-4.54598400	-0.16029600	0.39882100
C	-2.89922100	-0.89894500	-0.80151500
H	-3.42582400	-1.71874100	-1.29748600
H	-2.93283800	-0.02271900	-1.46098600
N	3.92452700	-1.22369300	0.57656500
H	3.48511900	-1.30663200	1.48995000
H	4.83739500	-1.66562900	0.63871100

32_{RC}

C	1.39493900	0.40842100	0.61496000
C	0.19912000	-0.18012900	-0.19079000
C	0.47623000	-1.65806900	-0.43458000
O	-0.40248000	-2.51873000	-0.33825000
C	-1.09039000	-0.02531000	0.61463000
O	-1.12057100	0.16912000	1.82712000
O	-2.20203000	-0.13254000	-0.16410000
C	-3.44347000	-0.06865000	0.54807000
H	-3.52969100	0.90040900	1.05198000
C	1.84940000	-2.04056900	-0.93104000
H	1.97019000	-3.12600900	-0.84615000
H	-3.48775000	-0.87598000	1.28752000
C	-4.57025000	-0.22749100	-0.45356000
H	-4.48445000	-1.18289100	-0.98173000
H	-4.52520100	0.56074900	-1.21238000
H	-5.54335000	-0.18450100	0.04347000
N	2.68124000	0.09889100	-0.02589000
O	1.48234000	-0.09044900	1.94171000
H	0.62562000	0.08913100	2.38529000
H	1.92419000	-1.77791900	-1.99132000
C	2.91593000	-1.34374900	-0.11212000
C	1.29033900	1.95068100	0.70949000
H	0.44430900	2.22810100	1.35169000
H	2.16914900	2.36060100	1.22606000
C	1.13265900	2.63347100	-0.64679000
H	2.05755900	2.53046100	-1.22687000
H	0.97457900	3.70802100	-0.49957000
C	-0.03204100	2.05096000	-1.43749000
H	-0.05592100	2.50091000	-2.43669000
H	-0.97680100	2.32030000	-0.95064000
C	0.07470900	0.53106000	-1.56602000
H	-0.78999100	0.15513000	-2.12719000
H	0.94266900	0.30357100	-2.19766000
H	2.97628000	-1.78588900	0.88990000
H	3.89166000	-1.51129800	-0.58179000
H	3.40715900	0.49190100	0.57694000

32_{RE}

C	-3.14802000	-0.73381100	-0.37197100
C	-2.65575000	1.72849900	-0.58387100
C	-1.76277000	1.81306900	0.65933900
N	-2.29986900	-1.75219100	-0.06121100
C	-2.70093000	0.37874900	-1.29581100
H	-2.36110100	2.50309900	-1.30210100
H	-3.67810000	1.97911900	-0.27057100
H	-1.97474000	0.99144900	1.35060900
O	-4.27025000	-0.72970100	0.13381900
H	-2.06516100	2.72398900	1.19447900
C	-0.25577000	1.94801000	0.40087900
H	-0.09569100	2.73228000	-0.35067100
H	0.20030000	2.33118000	1.32484900
C	0.49918000	0.67357000	-0.01965100
C	-0.97948900	-2.07414000	-0.56774100
H	-1.01455900	-3.13712000	-0.83357100
H	-0.76900900	-1.53759000	-1.49272100
C	0.11675100	-1.87293000	0.46763900
H	1.07866100	-2.19772000	0.05828900
H	-0.09080900	-2.51093000	1.33613900
C	0.27255000	-0.45100000	0.97875900
O	0.30774000	-0.23908000	2.19428900
H	-1.75588000	0.17363900	-1.78940100
H	-3.44041000	0.42718900	-2.10547100
H	0.15875000	0.37245000	-1.00984100
C	1.97815000	0.97437000	-0.17895100
O	2.45613000	2.09298100	-0.31283100
O	2.69919000	-0.18081900	-0.25227100
C	4.11128000	0.01185100	-0.41307100
H	4.30669000	0.53043100	-1.35820100
H	4.50239000	0.60300100	0.42251900
C	4.77277100	-1.35224900	-0.43014100
H	5.85554100	-1.25957800	-0.55195100
H	4.37550100	-1.96299900	-1.24763100
H	4.56599100	-1.89230900	0.49977900
H	-2.72947900	-2.41521100	0.57743900

Gaussian Calculations

32$_{RO}$

M06-2X/6-31G* with Solvent Correction

SCF Done: E(RM062X) = -824.037445315

Zero-point correction= 0.310643
Thermal correction to Gibbs Free Energy= 0.263807

C	-1.10558500	-1.53885100	-0.64535500
C	-0.42786800	-0.42868300	0.18423400
O	-0.46850200	-2.48156400	-1.06235500
C	1.04133300	-0.84125500	0.42748500
O	1.35867500	-1.33863200	1.48474600
C	-0.44435100	0.85802800	-0.63557700
O	-0.71587500	0.91829100	-1.81328400
O	-0.07424500	1.90433200	0.09944200
C	0.00691400	3.16820000	-0.59442000
H	-0.97677700	3.39298500	-1.01472100
C	2.01851500	-0.62360200	-0.70457900
H	1.54528800	-0.97169100	-1.62955600
H	2.17154600	0.46020700	-0.80775800
C	3.36048300	-1.30428200	-0.46904200
H	3.95474500	-1.21888500	-1.38342800
H	3.18359100	-2.37633100	-0.29350900
H	0.71578300	3.06124400	-1.42006500
C	0.45191700	4.20257700	0.41381100
H	1.43000400	3.94279400	0.82581400
H	-0.26704900	4.27652100	1.23337800
H	0.52703800	5.17851500	-0.07186900
C	-1.18818500	-0.26100300	1.51535200
H	-1.05321800	-1.18208500	2.09175200
H	-0.72287700	0.54797200	2.08423500
C	-2.67593000	-0.00284400	1.27991400
H	-3.17898800	0.12684400	2.24290300
H	-2.81059300	0.93561600	0.72378400
C	-3.31039100	-1.16048400	0.50843000
H	-3.24820100	-2.07324300	1.11329400
H	-4.37111700	-0.96949500	0.32259900
C	-2.59717000	-1.39254300	-0.83485400
H	-2.96337400	-2.28566900	-1.34603200
H	-2.75994100	-0.52671800	-1.48814700
N	4.08799000	-0.63950400	0.61431000
H	3.61388300	-0.86141000	1.48761800
H	5.01581200	-1.05159300	0.68875400

32_{RC}

M06-2X/6-31G* with Solvent Correction

SCF Done: E(RM062X) = -824.056389535

Zero-point correction= 0.314200
Thermal correction to Gibbs Free Energy= 0.271914

C	1.42543100	0.39781800	0.62440700
C	0.20916300	-0.14992100	-0.19692600
C	0.45445900	-1.64509800	-0.43991400
O	-0.37234700	-2.48184600	-0.14527600
C	-1.09037300	-0.05264200	0.60198000
O	-1.16291200	0.04246800	1.81301100
O	-2.16257200	-0.10920800	-0.17591800
C	-3.43812700	-0.11035700	0.49646200
H	-3.51844600	0.80541900	1.08799100
C	1.79710800	-1.98647600	-1.04361600
H	1.91231700	-3.07219500	-1.03893100
H	-3.46433600	-0.96307300	1.17986700
C	-4.50394200	-0.19767300	-0.57218600
H	-4.39003300	-1.11480200	-1.15514500
H	-4.44303300	0.65873400	-1.24841400
H	-5.49212100	-0.20237700	-0.10574100
N	2.62478500	0.12981700	-0.13896700
O	1.51787900	-0.28182700	1.86703200
H	0.65077100	-0.18591700	2.30205200
H	1.80708900	-1.63809800	-2.08294200
C	2.92596600	-1.29671900	-0.26747700
C	1.28387800	1.90778900	0.83924300
H	0.42104900	2.07421100	1.49533900
H	2.16956700	2.25287800	1.38580200
C	1.10220900	2.67673800	-0.47128700
H	2.01716400	2.59335400	-1.06834300
H	0.94870900	3.73928100	-0.25658700
C	-0.07607800	2.12300300	-1.27545500
H	-0.16177300	2.64675800	-2.23297300
H	-1.01480500	2.30257900	-0.73285800
C	0.08776000	0.62296200	-1.53196800
H	-0.75383100	0.22754100	-2.10599200
H	1.00207700	0.45915300	-2.10716700
H	3.04462700	-1.78698700	0.70658300
H	3.86356800	-1.40018100	-0.81891600
H	3.40283700	0.60177100	0.31876200

32_{RE}

M06-2X/6-31G* with Solvent Correction

SCF Done: E(RM062X) = -824.056997205

Zero-point correction= 0.312604
Thermal correction to Gibbs Free Energy= 0.266697

C	-3.02384600	-0.79319200	-0.33521200
C	-2.61724500	1.59460600	-0.94001100
C	-1.83023000	1.89288800	0.34183600
N	-2.20432400	-1.78651500	0.10735100
C	-2.55435500	0.14147200	-1.43692300
H	-2.28277700	2.25932200	-1.74506800
H	-3.67062500	1.82212500	-0.75131900
H	-2.09028100	1.15973300	1.11586900
O	-4.12874300	-0.63111400	0.17186300
H	-2.18138100	2.85831000	0.72257700
C	-0.30669300	2.00175200	0.21546000
H	-0.05074800	2.70271000	-0.58717900
H	0.09043500	2.42976100	1.14015700
C	0.45750600	0.69852100	-0.03038500
C	-0.87549800	-2.18052800	-0.32645300
H	-0.86157800	-3.26393400	-0.47517300
H	-0.66536700	-1.73660700	-1.29926500
C	0.21071500	-1.81485600	0.69537900
H	1.19102600	-2.06274900	0.27288600
H	0.06948800	-2.38541100	1.61722300
C	0.19452900	-0.34669100	1.06877700
O	-0.00117000	0.01898200	2.20675500
H	-1.56764100	-0.10506400	-1.82557100
H	-3.25810800	0.01946700	-2.26696500
H	0.23692200	0.25825100	-1.00695400
C	1.96056600	0.96658600	-0.03744400
O	2.47742400	2.02367000	0.22762900
O	2.64370200	-0.13446300	-0.37044900
C	4.08007000	-0.00946700	-0.38372700
H	4.35381500	0.75373200	-1.11710700
H	4.40787300	0.33133900	0.60179800
C	4.64241600	-1.36824400	-0.73571800
H	5.73384800	-1.32034900	-0.75902600
H	4.28797100	-1.68999500	-1.71813800
H	4.34390500	-2.11260900	0.00707200
H	-2.61381300	-2.32611400	0.86287800

Isomer $33_{RO}/33_{RC}/33_{RE}$

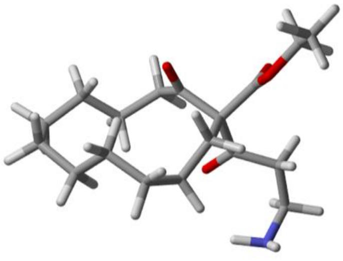

33_{RO}
ΔG ° = 36.6 kcal/mol

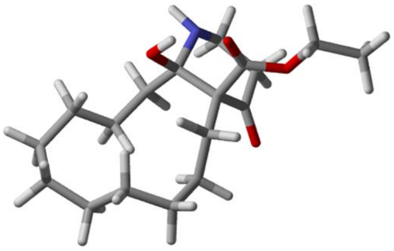

33_{RC}
ΔG ° = 45.4 kcal/mol

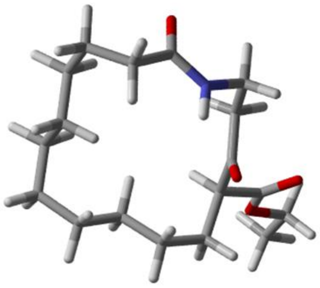

33_{RE}
ΔG ° = 0.0 kcal/mol

Gaussian Calculations

33_{RO}

B3LYP/6-31G*

SCF Done: E(RB3LYP) = -1060.22755152

Zero-point correction= 0.480135
Thermal correction to Gibbs Free Energy= 0.423565

H	-4.30537400	-0.58720400	2.25747800
C	-3.75911300	-0.13197100	1.42004500
H	-3.32418200	-0.97511600	0.86834100
C	-2.64120000	0.74590400	2.01613300
H	-3.09994000	1.46094200	2.71274900
H	-2.17385600	1.35357800	1.23089000
C	-1.55585500	-0.06387500	2.75252200
H	-1.00233100	0.58969600	3.44058300
H	-2.04277700	-0.82224400	3.38116300
C	-0.56365500	-0.75500600	1.79595500
H	-1.12051300	-1.17397200	0.95214800
H	-0.10707700	-1.62076500	2.29296200
C	0.55919200	0.18674200	1.29346800
C	-4.75779500	0.62360300	0.50559500
H	-4.69190900	1.70146400	0.71286600
H	-5.78082900	0.33370400	0.77901400
H	0.27433100	1.23362100	1.42628000
C	-4.61383000	0.40214700	-1.01496000
H	-4.77894400	-0.66193500	-1.23997500
H	-5.44271100	0.93591200	-1.49802300
C	-3.28090100	0.86473600	-1.65363000
C	0.93255600	0.04908900	-0.21929700
C	-0.16396300	0.80841400	-1.06965100
C	-2.28011500	-0.27515500	-1.91481100
C	-0.84879900	0.20508800	-2.27858900
H	-0.91358200	1.00111200	-3.02959500

H	-0.27403300	-0.62385300	-2.69067800
H	-2.21658400	-0.94951400	-1.05540600
H	-2.64444300	-0.89231800	-2.74618400
H	-3.48608400	1.35571800	-2.61467300
H	-2.81777900	1.63530500	-1.02841200
C	1.12971500	-1.44725800	-0.53432800
O	0.19107700	-2.14030400	-0.88896800
C	2.50205500	-2.05539000	-0.27897600
H	2.91591500	-1.62839800	0.64742800
H	3.16469300	-1.70755100	-1.08382200
C	2.49349600	-3.59368700	-0.21881300
H	2.11668200	-3.98250200	-1.17021100
H	3.53057300	-3.93800300	-0.12489100
N	1.70782600	-4.20770700	0.84936400
H	1.99582300	-3.84795200	1.75839800
H	0.72742600	-3.96341700	0.72725400
O	-0.42590400	1.94244900	-0.71091900
C	2.20391500	0.82394700	-0.60692500
O	2.69314700	0.74058700	-1.71708200
O	2.67958700	1.61184000	0.36494200
C	3.81966400	2.43950400	0.01329700
H	3.54618500	3.05136400	-0.85121800
H	4.64421900	1.78554700	-0.28707900
C	4.16203200	3.27875000	1.22858500
H	5.01932400	3.92198800	1.00253000
H	4.42283100	2.64589600	2.08295500
H	3.31855100	3.91516500	1.51343100
H	1.46396700	0.03760900	1.89065400

M06-2X/6-31G* with Solvent Correction

SCF Done: E(RM062X) = -1059.76966473

Zero-point correction= 0.485627
Thermal correction to Gibbs Free Energy= 0.430912

H	-3.12388700	2.66577100	1.95811300
C	-2.68188500	2.24539200	1.04735800
H	-2.35380400	1.23468900	1.32915100
C	-1.47556800	3.11091100	0.65498200
H	-1.76575300	4.16632700	0.72133600
H	-1.23344100	2.94759900	-0.40138500
C	-0.20814700	2.89482900	1.49717800
H	0.57588200	3.55042200	1.09877100
H	-0.38822600	3.21507400	2.53018800
C	0.32046700	1.44446300	1.50913500
H	-0.23827200	0.85921500	2.24446000
H	1.36654200	1.44389900	1.83450900
C	0.18129400	0.83218700	0.11354200
C	-3.76143100	2.12566100	-0.05363200
H	-3.66652200	2.96549100	-0.75542600
H	-4.75175500	2.22437100	0.40359200
H	-0.88005700	0.73700100	-0.09776700
C	-3.74012900	0.79677100	-0.82794500
H	-3.73076000	-0.01679800	-0.08791300
H	-4.68537900	0.68883900	-1.37104000
C	-2.56583200	0.59644000	-1.81517200
C	0.83599200	-0.52181900	-0.23655900
C	0.72476800	-0.65299400	-1.78171400
C	-1.94554000	-0.80515700	-1.70530700
C	-0.61323000	-1.00268400	-2.44175800
H	-0.61791100	-0.47048700	-3.39891700
H	-0.49476500	-2.06520400	-2.68664400
H	-1.84529600	-1.07761500	-0.64707100
H	-2.66336100	-1.53016800	-2.10639000
H	-2.91356800	0.74470800	-2.84388600
H	-1.79947700	1.36306000	-1.66327500
C	0.22440500	-1.79720900	0.36915500
O	0.29109500	-2.82453100	-0.27444000
C	-0.41767700	-1.75439600	1.73537900
H	-1.17253400	-0.95551400	1.74338700
H	0.35504800	-1.45128200	2.45617200
C	-1.06286300	-3.08688300	2.12021400
H	-0.30831600	-3.87757300	2.07736800
H	-1.40533700	-3.02321800	3.15737400
N	-2.18985600	-3.49286000	1.28376700
H	-2.89023400	-2.75258200	1.26673200

H	-1.86140000	-3.60358200	0.32659000
O	1.70435200	-0.49548300	-2.47362300
C	2.31852000	-0.53403000	0.13773200
O	2.88899600	-1.48904200	0.61188600
O	2.89787300	0.63678900	-0.10650800
C	4.30644900	0.71638500	0.18716000
H	4.82761600	-0.02726000	-0.42156000
H	4.45599500	0.46244800	1.24004600
C	4.74654400	2.12768600	-0.12961700
H	5.81344800	2.23678000	0.07990700
H	4.19672300	2.84826000	0.48155900
H	4.57341600	2.35709500	-1.18379600
H	0.57905000	1.55507200	-0.60984900

33_{RC}

B3LYP/6-31G*

SCF Done: E(RB3LYP) = -1060.22166765

Zero-point correction= 0.483587
Thermal correction to Gibbs Free Energy= 0.431760

H	-4.87271600	2.23550200	0.73525500
C	-4.19580700	1.44520500	0.38371700
H	-3.90177100	0.89480000	1.28606300
C	-2.97463600	2.11949700	-0.27516600
H	-3.35200300	2.90541800	-0.94356300
H	-2.48053000	1.39915000	-0.93796500
C	-1.93758600	2.73460800	0.69044500
H	-1.43129100	3.56760100	0.18170600
H	-2.45950500	3.18332100	1.54715000
C	-0.84773600	1.77142500	1.21682400
H	-1.29920900	0.93014600	1.74631700
H	-0.24775300	2.28681800	1.97065100
C	0.07759400	1.31761500	0.06417000
C	-4.95980000	0.50583300	-0.58389500
H	-4.79691200	0.85605500	-1.61377000
H	-6.03847200	0.61122500	-0.40651000
H	-0.49531700	1.21413900	-0.86156100
C	-4.65008000	-1.00743300	-0.51406200
H	-4.98810600	-1.38966200	0.46149600
H	-5.29319800	-1.49480400	-1.25991000
C	-3.19788200	-1.48045400	-0.74801900
C	0.94367700	0.01324500	0.13341800
C	0.23875800	-1.33090700	-0.38881500
C	-2.30813000	-1.29118100	0.48976900
C	-0.90055400	-1.91189100	0.48669000
H	-0.99190000	-2.96349700	0.17732900
H	-0.55256100	-1.93530400	1.52660600
H	-2.23629700	-0.23385300	0.71723000
H	-2.83419700	-1.73906100	1.34636600
H	-3.22156200	-2.55012700	-1.00273900
H	-2.75947700	-0.97565000	-1.61477100
C	1.57593200	-0.29074000	1.50878800
O	1.37158500	0.39553700	2.49045100
C	2.06016100	0.32677000	-0.88848600
O	1.92669200	0.28953200	-2.10022600
O	3.17498300	0.79418800	-0.30539300
C	4.23067000	1.24793000	-1.19428900
H	3.82348100	2.03019500	-1.84164200
H	4.53164000	0.40993500	-1.82988500

C	5.36698900	1.75307500	-0.32674200
H	6.18259300	2.11427200	-0.96248900
H	5.75817000	0.95633800	0.31400200
H	5.03554900	2.57781200	0.31176500
O	-0.28280000	-1.16814500	-1.67924200
H	0.43165300	-0.75809000	-2.21343300
N	1.36924300	-2.29103000	-0.47642100
H	1.04388300	-3.08777500	-1.02162000
C	1.92349900	-2.73088500	0.80552000
H	2.73433900	-3.43768400	0.59813000
H	1.19550400	-3.26196300	1.44021200
C	2.47992900	-1.52018300	1.57287700
H	2.65463400	-1.75620600	2.62611700
H	3.43747200	-1.22466600	1.12975100
H	0.76547800	2.15475900	-0.11253700

M06-2X/6-31G* with Solvent Correction

SCF Done: E(RM062X) = -1059.78250183

Zero-point correction= 0.489228
Thermal correction to Gibbs Free Energy= 0.438533

H	-3.95801200	2.28472500	1.32067300
C	-3.50466400	1.63560000	0.56221300
H	-2.82510900	0.97745700	1.11583200
C	-2.70431700	2.50000100	-0.41966400
H	-3.34370000	3.31623400	-0.77877700
H	-2.47256200	1.89849000	-1.30815600
C	-1.40012300	3.08830000	0.13619400
H	-0.91452900	3.65418300	-0.66909700
H	-1.62602000	3.80957200	0.93129100
C	-0.41363600	2.03702800	0.67827500
H	-0.71801400	1.70876500	1.67463500
H	0.57920300	2.48960800	0.79052000
C	-0.35607900	0.82440400	-0.25070700
C	-4.58913300	0.78334700	-0.13729900
H	-4.59688600	1.02302700	-1.20931200
H	-5.58166600	1.05759100	0.23714000
H	-1.31572400	0.32367800	-0.22366500
C	-4.41991600	-0.73496900	0.01772000
H	-4.49168900	-0.99297500	1.08350500
H	-5.27511500	-1.22004300	-0.46616500
C	-3.10540200	-1.31908400	-0.55305900
C	0.74381900	-0.23978700	-0.02919200
C	0.32394900	-1.62668600	-0.65319100
C	-2.11638400	-1.72773500	0.54573000
C	-0.79682900	-2.37830200	0.10462000
H	-1.02146100	-3.23917100	-0.53991700
H	-0.35336800	-2.79034600	1.01591700
H	-1.90802400	-0.89538200	1.22797500
H	-2.62440800	-2.47813700	1.16613500
H	-3.32474900	-2.21491100	-1.14678400
H	-2.64887300	-0.61341500	-1.25791900
C	1.11898800	-0.59380800	1.40956600
O	0.46055900	-0.25393500	2.36897400
C	1.94432300	0.30955200	-0.79945500
O	2.08629800	0.23245800	-2.00251000
O	2.75821000	1.02017500	-0.01919200
C	3.85269300	1.69341800	-0.67774100
H	3.43713300	2.36484300	-1.43341800
H	4.46324200	0.94218400	-1.18513300
C	4.62427500	2.43573500	0.38941800
H	5.46509600	2.96415500	-0.06648000

H	5.01493400	1.74104400	1.13725800
H	3.98402500	3.16656600	0.88968200
O	-0.10528000	-1.46195600	-1.97476300
H	0.62075000	-0.99281200	-2.43078600
N	1.56124100	-2.42805900	-0.66978600
H	1.37832900	-3.26900500	-1.21541900
C	2.09936800	-2.79118700	0.64218400
H	3.06554200	-3.27767000	0.48583600
H	1.46790500	-3.50592000	1.18788400
C	2.30238400	-1.54062700	1.51925900
H	2.43009900	-1.82115600	2.56639100
H	3.19711400	-1.00612200	1.18787000
H	-0.25033800	1.16622700	-1.28830500

33_{RE}

B3LYP/6-31G*

SCF Done: E(RB3LYP) = -1060.28642169

Zero-point correction= 0.481319
Thermal correction to Gibbs Free Energy= 0.424183

H	3.30762000	3.04826100	-1.92831800
C	2.65635100	2.60388700	-1.16253800
H	1.85376200	2.09790800	-1.71751900
C	2.06282900	3.74183100	-0.31078800
H	1.51113400	4.42310900	-0.97430500
H	2.88564600	4.33408900	0.11412000
C	-1.98889600	0.00675500	0.28841000
C	3.45418400	1.55304300	-0.37662200
H	2.80618800	1.10537500	0.38833200
H	4.27330100	2.04570900	0.16948600
C	4.02259700	0.44514200	-1.27462600
H	3.20353500	-0.03508100	-1.82622200
H	4.67183700	0.90104500	-2.03536600
C	4.82701800	-0.64314700	-0.54385500
C	4.04051800	-1.44753200	0.50402400
H	4.71227000	-2.18717100	0.96132700
H	3.69231900	-0.80490000	1.32217600
H	5.21073400	-1.34937400	-1.28773000
H	5.69763600	-0.19079800	-0.04965400
C	-0.99247400	-1.06465600	0.76271200
O	-0.60429100	-1.09996500	1.91706800
C	-3.33538500	-0.66003200	0.00904200
O	-3.63577800	-1.79039200	0.33402600
O	-4.16534300	0.17811200	-0.64030200
C	-5.49231400	-0.33334200	-0.92541200
H	-5.96493600	-0.62345100	0.01801100
H	-5.39390200	-1.23461600	-1.53853400
C	-6.25877300	0.76405800	-1.63811800
H	-5.76461200	1.04327400	-2.57410100
H	-6.33967500	1.65704300	-1.01020000
H	-7.27020800	0.41547100	-1.87322700
N	1.77366300	-2.35016800	0.69723700
C	0.56080800	-3.02351700	0.26072600
H	0.14951800	-3.58777000	1.10327300
H	0.84914500	-3.72530700	-0.52403900
C	-0.49564400	-2.05425200	-0.28041900
H	-0.08954600	-1.49836100	-1.13688800
H	-1.36587200	-2.60891900	-0.65609200
C	2.87163000	-2.22271100	-0.10642000

O	2.93186300	-2.72104400	-1.22762200
H	1.72110300	-1.83908500	1.56697400
C	1.14016400	3.30394400	0.84120800
H	1.71741500	2.71900200	1.57044700
H	0.80340700	4.20003200	1.38111800
C	-0.08920000	2.48757600	0.41531500
H	0.24048800	1.63676700	-0.19591400
H	-0.73247800	3.09944300	-0.23538400
C	-0.88923700	1.96951400	1.61800700
H	-1.18423000	2.82127400	2.24682200
H	-0.23927800	1.34216200	2.23709000
C	-2.16878200	1.18366200	1.27927200
H	-2.59575500	0.79129900	2.20940600
H	-2.91274200	1.85938200	0.84254100
H	-1.63344800	0.40033800	-0.67314700

M06-2X/6-31G* with Solvent Correction

SCF Done: E(RM062X) = -1059.82887457

Zero-point correction= 0.485933
Thermal correction to Gibbs Free Energy= 0.429628

H	3.30919900	2.83925600	-1.97884100
C	2.60799700	2.45446000	-1.22714400
H	1.82640700	1.92437400	-1.78818100
C	2.00272600	3.64215200	-0.47099000
H	1.44436000	4.26887000	-1.17831500
H	2.81868600	4.26456000	-0.08168500
C	-2.02933400	0.07395200	0.15046200
C	3.33993000	1.45526900	-0.33032100
H	2.61420100	0.96137400	0.33118600
H	4.04107600	1.99302400	0.32545000
C	4.09751400	0.40053000	-1.13609400
H	3.38462000	-0.17298200	-1.74359400
H	4.76734900	0.90630200	-1.84310300
C	4.92401100	-0.56971500	-0.28826500
C	4.09767800	-1.36471800	0.72683800
H	4.74363000	-2.08700200	1.23957700
H	3.67840600	-0.71031800	1.49795000
H	5.42930600	-1.28018900	-0.94970100
H	5.70309500	-0.01786600	0.25092200
C	-0.96579900	-0.92599200	0.59483000
O	-0.62268700	-1.00356300	1.75700300
C	-3.32610000	-0.69302300	-0.05644600
O	-3.47221300	-1.87303500	0.17355000
O	-4.29486700	0.09937200	-0.51321400
C	-5.57761000	-0.52668700	-0.71819900
H	-5.90794000	-0.95878800	0.23005700
H	-5.45506100	-1.33993800	-1.43850300
C	-6.52171700	0.54303100	-1.21873200
H	-6.16186100	0.96542400	-2.16012300
H	-6.61522800	1.34805000	-0.48565400
H	-7.51083100	0.11036200	-1.38777400
N	1.79322400	-2.15141900	0.65498900
C	0.62843400	-2.81288500	0.09896800
H	0.14468600	-3.40765400	0.87890700
H	0.98464200	-3.48449900	-0.68244800
C	-0.36530700	-1.81065800	-0.47670400
H	0.12896200	-1.16960500	-1.21985700
H	-1.18679200	-2.32009800	-0.99478000
C	2.99784400	-2.15319100	0.03385000
O	3.20167700	-2.75276000	-1.01824200
H	1.65935200	-1.61431800	1.50095000

C	1.08436900	3.25807800	0.69232300
H	1.66575500	2.74200500	1.46817400
H	0.69992300	4.17315000	1.16049500
C	-0.09558500	2.37469200	0.29065000
H	0.28674800	1.45817000	-0.18245100
H	-0.70019500	2.88714400	-0.47229500
C	-0.96216900	2.00122800	1.49191800
H	-1.25342000	2.91535400	2.02392600
H	-0.36058900	1.41048300	2.19040100
C	-2.24054600	1.23208700	1.14158900
H	-2.68867600	0.83613100	2.05933300
H	-2.97186900	1.91059800	0.69148200
H	-1.74380400	0.47680100	-0.83040400

Isomer $40_{RO}/40_{RC}/40_{RE}$

Spartan Calculations

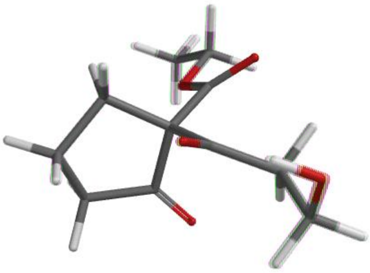

40_{RO}
ΔG ° = 9.3 kcal/mol

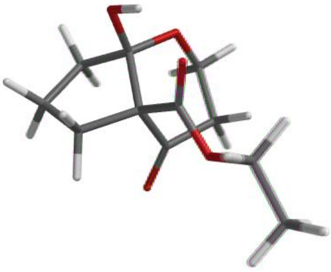

40_{RC}
ΔG ° = 11.8 kcal/mol

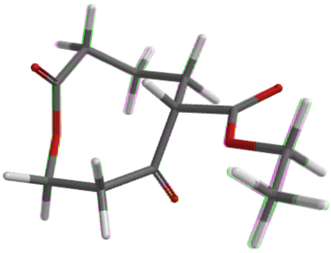

40_{RE}
ΔG ° = 0.0 kcal/mol

40_{RO}	Calculated energy (au)	Calculated energy (kcal/mol)	Relative energy (kcal/mol)
M0008	-804.91501	-505084.1675	0.00
M0003	-804.91467	-505083.9542	0.21
M0002	-804.91466	-505083.9504	0.22
M0016	-804.91449	-505083.8444	0.32
M0009	-804.91417	-505083.6417	0.53
M0007	-804.91415	-505083.6304	0.54
M0011	-804.91415	-505083.6298	0.54
M0017	-804.91372	-505083.3568	0.81
M0014	-804.91346	-505083.1955	0.97
M0020	-804.91308	-505082.9590	1.21
M0059	-804.91164	-505082.0535	2.11
M0018	-804.91151	-505081.9713	2.20
M0010	-804.91120	-505081.7786	2.39
M0022	-804.91120	-505081.7774	2.39
M0004	-804.91115	-505081.7491	2.42
M0001	-804.91096	-505081.6268	2.54
M0034	-804.91092	-505081.6029	2.56
M0037	-804.91056	-505081.3751	2.79
M0038	-804.91053	-505081.3569	2.81
M0041	-804.91049	-505081.3300	2.84
M0013	-804.91047	-505081.3224	2.85
M0005	-804.91034	-505081.2390	2.93
M0047	-804.91018	-505081.1392	3.03
M0046	-804.91014	-505081.1129	3.05
M0025	-804.91012	-505081.1016	3.07
M0019	-804.91009	-505081.0796	3.09
M0043	-804.91004	-505081.0520	3.12
M0052	-804.90983	-505080.9164	3.25
M0050	-804.90967	-505080.8154	3.35
M0023	-804.90949	-505080.7043	3.46
M0027	-804.90932	-505080.5983	3.57

M0056	-804.90930	-505080.5876	3.58
M0031	-804.90922	-505080.5337	3.63
M0051	-804.90914	-505080.4866	3.68
M0045	-804.90907	-505080.4383	3.73
M0024	-804.90897	-505080.3812	3.79
M0028	-804.90895	-505080.3680	3.80
M0029	-804.90878	-505080.2595	3.91
M0036	-804.90865	-505080.1779	3.99
M0021	-804.90862	-505080.1559	4.01
M0032	-804.90845	-505080.0511	4.12
M0026	-804.90840	-505080.0229	4.14
M0030	-804.90839	-505080.0141	4.15
M0033	-804.90830	-505079.9551	4.21
M0039	-804.90813	-505079.8491	4.32
M0078	-804.90808	-505079.8221	4.35
M0044	-804.90789	-505079.7035	4.46
M0006	-804.90789	-505079.6985	4.47
M0035	-804.90785	-505079.6765	4.49
M0012	-804.90785	-505079.6746	4.49
M0015	-804.90748	-505079.4424	4.73
M0090	-804.90711	-505079.2103	4.96
M0064	-804.90682	-505079.0296	5.14
M0070	-804.90674	-505078.9775	5.19
M0094	-804.90629	-505078.6963	5.47
M0042	-804.90598	-505078.5025	5.67
M0075	-804.90586	-505078.4246	5.74
M0068	-804.90583	-505078.4096	5.76
M0076	-804.90516	-505077.9854	6.18
M0061	-804.90515	-505077.9829	6.18
M0048	-804.90507	-505077.9283	6.24
M0085	-804.90495	-505077.8580	6.31
M0040	-804.90461	-505077.6403	6.53
M0063	-804.90456	-505077.6120	6.56
M0066	-804.90450	-505077.5750	6.59
M0072	-804.90445	-505077.5417	6.63
M0069	-804.90412	-505077.3359	6.83
M0067	-804.90397	-505077.2399	6.93
M0077	-804.90394	-505077.2242	6.94
M0082	-804.90386	-505077.1740	6.99
M0096	-804.90371	-505077.0780	7.09
M0071	-804.90358	-505076.9939	7.17
M0049	-804.90344	-505076.9105	7.26
M0074	-804.90340	-505076.8841	7.28
M0053	-804.90330	-505076.8214	7.35
M0055	-804.90328	-505076.8057	7.36
M0083	-804.90320	-505076.7605	7.41

M0054	-804.90318	-505076.7473	7.42
M0087	-804.90313	-505076.7141	7.45
M0091	-804.90307	-505076.6764	7.49
M0065	-804.90286	-505076.5447	7.62
M0057	-804.90284	-505076.5302	7.64
M0073	-804.90278	-505076.4926	7.67
M0084	-804.90277	-505076.4901	7.68
M0058	-804.90269	-505076.4386	7.73
M0060	-804.90265	-505076.4129	7.75
M0095	-804.90262	-505076.3953	7.77
M0062	-804.90258	-505076.3690	7.80
M0080	-804.90244	-505076.2780	7.89
M0092	-804.90237	-505076.2378	7.93
M0097	-804.90229	-505076.1863	7.98
M0099	-804.90218	-505076.1180	8.05
M0081	-804.90201	-505076.0132	8.15
M0079	-804.90194	-505075.9692	8.20
M0098	-804.90192	-505075.9554	8.21
M0089	-804.90175	-505075.8494	8.32
M0100	-804.90150	-505075.6925	8.48
M0088	-804.90148	-505075.6806	8.49
M0086	-804.90136	-505075.6040	8.56
M0093	-804.90071	-505075.1955	8.97
**40_{RC}**	**Calculated energy (au)**	**Calculated energy (kcal/mol)**	**Relative energy (kcal/mol)**
M0016	-804.91671	-505085.2349	0.00
M0019	-804.91647	-505085.0862	0.15
M0018	-804.91634	-505085.0027	0.23
M0037	-804.91572	-505084.6156	0.62
M0040	-804.91520	-505084.2886	0.95
M0041	-804.91507	-505084.2058	1.03
M0012	-804.91469	-505083.9655	1.27
M0020	-804.91461	-505083.9190	1.32
M0032	-804.91447	-505083.8287	1.41
M0025	-804.91437	-505083.7647	1.47
M0014	-804.91432	-505083.7333	1.50
M0005	-804.91428	-505083.7113	1.52
M0004	-804.91427	-505083.7063	1.53
M0015	-804.91421	-505083.6661	1.57
M0024	-804.91410	-505083.5952	1.64
M0006	-804.91395	-505083.5017	1.73
M0038	-804.91393	-505083.4936	1.74
M0039	-804.91382	-505083.4195	1.82
M0036	-804.91370	-505083.3480	1.89
M0001	-804.91352	-505083.2344	2.00
M0044	-804.91335	-505083.1265	2.11
M0042	-804.91328	-505083.0832	2.15

M0003	-804.91313	-505082.9859	2.25
M0002	-804.91312	-505082.9828	2.25
M0007	-804.91255	-505082.6226	2.61
M0033	-804.91236	-505082.5084	2.73
M0009	-804.91236	-505082.5046	2.73
M0010	-804.91225	-505082.4375	2.80
M0008	-804.91114	-505081.7378	3.50
M0011	-804.91087	-505081.5703	3.66
M0013	-804.91076	-505081.5019	3.73
M0043	-804.91062	-505081.4153	3.82
M0046	-804.91036	-505081.2522	3.98
M0047	-804.91027	-505081.1969	4.04
M0023	-804.91018	-505081.1392	4.10
M0045	-804.91004	-505081.0488	4.19
M0026	-804.91004	-505081.0488	4.19
M0028	-804.91004	-505081.0488	4.19
M0030	-804.91001	-505081.0319	4.20
M0049	-804.90996	-505080.9974	4.24
M0048	-804.90980	-505080.8995	4.34
M0017	-804.90959	-505080.7677	4.47
M0027	-804.90957	-505080.7564	4.48
M0029	-804.90953	-505080.7294	4.51
M0034	-804.90948	-505080.7012	4.53
M0031	-804.90939	-505080.6447	4.59
M0021	-804.90916	-505080.4948	4.74
M0022	-804.90901	-505080.4050	4.83
M0035	-804.90895	-505080.3630	4.87
**40_{RE}**	**Calculated energy (au)**	**Calculated energy (kcal/mol)**	**Relative energy (kcal/mol)**
M0012	-804.93905	-505099.2539	0.00
M0001	-804.93871	-505099.0424	0.21
M0005	-804.93858	-505098.9590	0.29
M0002	-804.93850	-505098.9069	0.35
M0006	-804.93827	-505098.7657	0.49
M0007	-804.93814	-505098.6829	0.57
M0036	-804.93813	-505098.6747	0.58
M0025	-804.93810	-505098.6552	0.60
M0034	-804.93796	-505098.5693	0.68
M0027	-804.93794	-505098.5586	0.70
M0031	-804.93767	-505098.3904	0.86
M0010	-804.93687	-505097.8859	1.37
M0028	-804.93660	-505097.7134	1.54
M0021	-804.93656	-505097.6920	1.56
M0035	-804.93655	-505097.6845	1.57
M0014	-804.93636	-505097.5659	1.69
M0038	-804.93632	-505097.5421	1.71
M0004	-804.93612	-505097.4122	1.84

M0009	-804.93591	-505097.2829	1.97
M0016	-804.93581	-505097.2220	2.03
M0018	-804.93516	-505096.8148	2.44
M0003	-804.93487	-505096.6290	2.62
M0008	-804.93469	-505096.5167	2.74
M0013	-804.93410	-505096.1496	3.10
M0032	-804.93404	-505096.1076	3.15
M0015	-804.93395	-505096.0555	3.20
M0011	-804.93393	-505096.0386	3.22
M0017	-804.93382	-505095.9708	3.28
M0039	-804.93380	-505095.9601	3.29
M0020	-804.93373	-505095.9143	3.34
M0042	-804.93370	-505095.8949	3.36
M0022	-804.93364	-505095.8604	3.39
M0037	-804.93357	-505095.8177	3.44
M0024	-804.93325	-505095.6119	3.64
M0041	-804.93300	-505095.4594	3.79
M0043	-804.93289	-505095.3860	3.87
M0026	-804.93222	-505094.9699	4.28
M0054	-804.93193	-505094.7861	4.47
M0053	-804.93191	-505094.7754	4.48
M0033	-804.93179	-505094.6976	4.56
M0029	-804.93161	-505094.5853	4.67
M0044	-804.93136	-505094.4303	4.82
M0030	-804.93136	-505094.4284	4.83
M0047	-804.93135	-505094.4196	4.83
M0059	-804.93127	-505094.3694	4.88
M0060	-804.93118	-505094.3180	4.94
M0069	-804.93110	-505094.2634	4.99
M0049	-804.93076	-505094.0494	5.20
M0019	-804.93066	-505093.9910	5.26
M0023	-804.93026	-505093.7369	5.52
M0040	-804.93012	-505093.6503	5.60
M0046	-804.93009	-505093.6334	5.62
M0051	-804.92947	-505093.2431	6.01
M0045	-804.92944	-505093.2217	6.03
M0056	-804.92941	-505093.2035	6.05
M0050	-804.92935	-505093.1684	6.09
M0048	-804.92924	-505093.0987	6.16
M0052	-804.92920	-505093.0755	6.18
M0068	-804.92879	-505092.8138	6.44
M0078	-804.92872	-505092.7687	6.49
M0067	-804.92813	-505092.4016	6.85
M0061	-804.92805	-505092.3514	6.90
M0081	-804.92795	-505092.2886	6.97
M0071	-804.92791	-505092.2660	6.99

M0072	-804.92789	-505092.2516	7.00
M0065	-804.92784	-505092.2190	7.03
M0092	-804.92772	-505092.1456	7.11
M0074	-804.92759	-505092.0634	7.19
M0094	-804.92754	-505092.0288	7.23
M0066	-804.92749	-505092.0012	7.25
M0087	-804.92732	-505091.8927	7.36
M0099	-804.92720	-505091.8205	7.43
M0055	-804.92666	-505091.4798	7.77
M0091	-804.92653	-505091.3944	7.86
M0057	-804.92644	-505091.3392	7.91
M0077	-804.92629	-505091.2463	8.01
M0096	-804.92624	-505091.2131	8.04
M0070	-804.92615	-505091.1598	8.09
M0079	-804.92613	-505091.1434	8.11
M0075	-804.92583	-505090.9602	8.29
M0084	-804.92571	-505090.8830	8.37
M0083	-804.92537	-505090.6697	8.58
M0063	-804.92529	-505090.6163	8.64
M0100	-804.92503	-505090.4557	8.80
M0086	-804.92501	-505090.4457	8.81
M0090	-804.92484	-505090.3390	8.91
M0080	-804.92473	-505090.2656	8.99
M0062	-804.92431	-505090.0058	9.25
M0058	-804.92418	-505089.9255	9.33
M0073	-804.92394	-505089.7711	9.48
M0064	-804.92389	-505089.7416	9.51
M0089	-804.92253	-505088.8882	10.37
M0093	-804.92232	-505088.7564	10.50
M0097	-804.92209	-505088.6090	10.64
M0082	-804.92203	-505088.5763	10.68
M0098	-804.92191	-505088.4992	10.75
M0088	-804.92176	-505088.4025	10.85
M0085	-804.92144	-505088.2036	11.05
M0095	-804.92120	-505088.0530	11.20
M0076	-804.92097	-505087.9093	11.34

Table S14. Energies calculated at DFT/B3LYP/6-31G* in vacuum for the conformers found for **40_{RO}/40_{RC}/40_{RE}**.

Isomer	ΔG° (au)	ΔG° (kcal/mol)	ΔG° (kcal/mol)
**40_{RO}**	-804.708316	-504954.47	9.3
**40_{RC}**	-804.704337	-504951.97	11.8
**40_{RE}**	-804.723193	-504963.80	0.0

Table S15. Relative free energies (ΔG°) of the lowest energy geometries of **40_{RO}/40_{RC}/40_{RE}** at DFT/B3LYP/6-31G* in vacuum in kcal/mol.

XYZ Coordinates

40_{RO}

C	-0.21655100	1.12429100	0.98067900
C	-0.54532000	2.57877100	0.77825900
C	-0.06178000	2.80978100	-0.64237100
C	-0.32146100	1.48773100	-1.35617100
C	0.00244900	0.38566100	-0.31935100
H	-0.01747000	3.21019100	1.49599900
H	-1.62727000	2.71391100	0.86348900
H	-0.57825000	3.64667100	-1.12171100
H	1.01159000	3.03437000	-0.64373100
H	0.27801900	1.40866100	-2.26968100
H	-1.37374100	1.45106100	-1.66756100
O	-0.13900100	0.60002100	2.08654900
C	1.45768900	-0.06129000	-0.39305100
O	2.30510900	0.60153000	-1.00266100
C	-0.96936200	-0.74841900	-0.52517100
O	-0.70240200	-1.78401900	-1.12116100
O	-2.20141200	-0.44917800	-0.02516100
C	-3.18193200	-1.48271800	-0.19120100
H	-2.84063200	-2.39894800	0.30332900
C	1.86852800	-1.32259000	0.35304900
H	1.16075800	-1.51157000	1.16505900
H	1.83403800	-2.15676000	-0.35496100
C	3.26276800	-1.16937100	0.94230900
H	3.47066800	-1.99571100	1.62874900
H	3.37258900	-0.22980100	1.49397900
H	-3.33803200	-1.67352800	-1.25871100
C	-4.47632200	-1.00970700	0.44046900
H	-4.81780100	-0.08146700	-0.02952100
H	-4.32937200	-0.79262700	1.50367900
H	-5.25944200	-1.76617700	0.33905900
O	4.24907800	-1.21122100	-0.08287100
H	4.05113800	-0.46818100	-0.68977100

40_{RC}

C	1.48414000	-0.48070100	-0.56600000
C	2.43713900	-1.09620200	0.46591000
C	1.53946800	-2.01585100	1.28365000
C	0.13655900	-1.40293000	1.21997000
C	0.21392000	-0.19868000	0.25503000
H	2.88775000	-0.34507200	1.12201000
H	3.25684900	-1.65376300	-0.00012000
H	1.52469800	-3.02116100	0.84641000
H	1.89606800	-2.11256100	2.31402000
H	-0.19725100	-1.12001000	2.22402000
H	-0.56785200	-2.16493900	0.86312000
C	0.40646100	1.12838000	0.96964000
O	0.10837200	1.30752000	2.15017000
C	-1.02126000	-0.12115900	-0.61316000
O	-1.00905000	-0.04669900	-1.83611000
O	-2.15171000	-0.09867800	0.14621000
C	-3.37353000	-0.00820700	-0.59796000
H	-3.46070000	-0.86881700	-1.27045000
C	0.93182200	2.26467900	0.13391000
H	0.18836300	2.50063000	-0.63403000
H	-3.38194900	0.91950300	-1.18083000
C	-4.52664000	-0.00657600	0.38615000
H	-4.44039900	0.83664400	1.07945000
H	-4.51759100	-0.91878600	0.99187000
H	-5.48515000	0.06162500	-0.13581000
O	2.04591100	0.62830800	-1.24780000
O	1.27101900	-1.49146100	-1.54940000
H	0.68316900	-1.08102000	-2.21521000
H	1.08398300	3.14912900	0.75965000
C	2.23647200	1.80570800	-0.47274000
H	2.62202300	2.58021800	-1.14380000
H	3.00433200	1.63567700	0.28879000

40_{RE}

C	2.90483100	1.19186700	-0.64093900
C	2.12492200	1.76003800	0.55331100
C	0.60358200	1.91012900	0.36809100
C	-0.20497900	0.66145000	-0.03582900
H	2.63644200	1.73434700	-1.55405900
H	3.98153100	1.31861600	-0.48309900
H	2.32046100	1.17361700	1.45863100
H	2.52529300	2.76083700	0.76206100
H	0.43325300	2.69104900	-0.38557900
H	0.19629200	2.31300900	1.30562100
C	0.03417000	-0.51883100	0.89981100
O	-0.06294000	-0.41128100	2.12273100
C	-1.68471900	0.98124100	-0.11741900
O	-2.16011800	2.10881100	-0.13286900
O	-2.41117000	-0.16523800	-0.25847900
C	-3.82621000	0.04076300	-0.36056900
H	-4.04818900	0.63695300	-1.25259900
C	0.41731800	-1.84281100	0.26035100
H	-0.18458200	-2.64322000	0.70377100
H	-4.19049900	0.56030300	0.53275100
C	-4.49292100	-1.31635700	-0.47181900
H	-4.26068200	-1.93258700	0.40309100
H	-4.12254200	-1.85699700	-1.34910900
H	-5.57849100	-1.21246600	-0.55294900
O	2.70280900	-0.94097300	0.32365100
H	0.14623900	-1.81497100	-0.80035900
C	1.90752800	-2.13072200	0.42366100
H	0.08474100	0.38672900	-1.05533900
C	2.64807000	-0.27783300	-0.85900900
O	2.32228900	-0.77284300	-1.92765900
H	2.08848800	-2.53555200	1.42542100
H	2.25572800	-2.88108300	-0.29504900

Gaussian Calculations

40$_{RO}$

M06-2X/6-31G* with Solvent Correction

SCF Done: E(RM062X) = -804.601454413

Zero-point correction= 0.268276
Thermal correction to Gibbs Free Energy= 0.222423

C	-0.26593900	1.11414200	1.03979400
C	-0.53415800	2.58054800	0.76652600
C	-0.03599200	2.79908500	-0.66250000
C	-0.34604700	1.46817800	-1.36932500
C	-0.02559900	0.38523300	-0.32092400
H	-0.07589600	3.20622000	1.53496600
H	-1.62292600	2.71457100	0.82550400
H	-0.51974700	3.64532600	-1.15346500
H	1.04389100	2.97289200	-0.66602600
H	0.24052700	1.32507100	-2.27763400
H	-1.40980000	1.41910900	-1.62042600
O	-0.22529200	0.56551600	2.11123500
C	1.45633100	-0.02781900	-0.35124600
O	2.23741500	0.60591200	-1.03456100
C	-0.94767400	-0.81882800	-0.42199700
O	-0.62266500	-1.92552300	-0.77902800
O	-2.19601500	-0.47526700	-0.09731300
C	-3.18365600	-1.52259300	-0.18845100
H	-2.88124300	-2.33879900	0.47270500
C	1.91073500	-1.16484500	0.53322700
H	1.30194600	-1.17831900	1.44379900
H	1.69979700	-2.09641200	-0.00369300
C	3.40226800	-1.07260000	0.84850300
H	3.67143700	-1.86600800	1.55019200
H	3.61729500	-0.11034300	1.33540200
H	-3.19329500	-1.90004000	-1.21437900
C	-4.51048500	-0.91997200	0.21405800
H	-4.78095700	-0.10013000	-0.45600900
H	-4.46522400	-0.53789300	1.23675300
H	-5.29122200	-1.68275400	0.16209800
O	4.20395800	-1.25414000	-0.30000600
H	3.96749600	-0.52764400	-0.89839400

40_{RC}

M06-2X/6-31G* with Solvent Correction

SCF Done: E(RM062X) = -804.613309952

Zero-point correction= 0.271391
Thermal correction to Gibbs Free Energy= 0.228568

C	1.54665300	-0.39453800	-0.59219200
C	2.70350500	-0.24483700	0.39811700
C	2.22683700	-0.99523800	1.65975500
C	0.68476200	-1.14971600	1.53691100
C	0.29481600	-0.26533400	0.34394400
H	2.88048200	0.81095400	0.62260300
H	3.61498800	-0.65585100	-0.04041600
H	2.69638500	-1.97844400	1.72579500
H	2.48888300	-0.44094600	2.56320600
H	0.15586600	-0.83312700	2.43718000
H	0.41893100	-2.18651500	1.31743900
C	0.18401300	1.21034900	0.74020800
O	0.25489300	1.58299300	1.88913200
C	-0.98757500	-0.71959900	-0.32433100
O	-1.06014700	-1.54715900	-1.21053800
O	-2.05346400	-0.12335100	0.20341900
C	-3.33751100	-0.51082000	-0.33297800
H	-3.46182800	-1.58601100	-0.18100900
C	0.12347700	2.14026700	-0.44751400
H	-0.77332300	1.91985500	-1.03764400
H	-3.33321100	-0.31566600	-1.40850500
C	-4.38623900	0.29876900	0.39485300
H	-4.22667900	1.36808900	0.23618100
H	-4.35481200	0.09495100	1.46773700
H	-5.37803800	0.03567100	0.01913000
O	1.50574800	0.52504000	-1.66402600
O	1.64413300	-1.66103400	-1.14005500
H	0.78136800	-1.86214200	-1.55031100
H	0.08620900	3.17703500	-0.10746100
C	1.36843500	1.89001700	-1.31565900
H	1.28524000	2.43156200	-2.25925200
H	2.26319300	2.25277000	-0.79481900

40_{RE}

M06-2X/6-31G* with Solvent Correction

SCF Done: E(RM062X) = -804.624502966

Zero-point correction= 0.270806
Thermal correction to Gibbs Free Energy= 0.226862

C	2.88410200	1.07149700	-0.76084300
C	2.19433200	1.77685900	0.42030300
C	0.67873300	1.97364700	0.28310200
C	-0.12138200	0.71340300	-0.05861000
H	2.65181100	1.54596400	-1.71805800
H	3.96995400	1.11739100	-0.61933400
H	2.41214500	1.21841600	1.33483700
H	2.64957800	2.76487800	0.53800300
H	0.46680800	2.71288800	-0.49653400
H	0.29003200	2.38396800	1.21995200
C	0.04686700	-0.42868600	0.95803400
O	0.04076300	-0.19660000	2.14522500
C	-1.61073400	1.02295000	-0.13408100
O	-2.10198600	2.12198900	-0.07696900
O	-2.31507900	-0.10749300	-0.27698300
C	-3.74594900	0.04293100	-0.35903600
H	-3.97913500	0.67162500	-1.22267100
C	0.25560400	-1.83491900	0.40748400
H	-0.31433100	-2.54282800	1.01439900
H	-4.09248300	0.55821100	0.54064000
C	-4.33338100	-1.34467700	-0.48630700
H	-4.07243600	-1.95361300	0.38288700
H	-3.96058200	-1.83998100	-1.38628300
H	-5.42230800	-1.27986200	-0.55092200
O	2.53458000	-0.98195000	0.33493000
H	-0.09270400	-1.89025900	-0.62547800
C	1.75225200	-2.17557600	0.49850600
H	0.14599600	0.34154900	-1.05426500
C	2.49468100	-0.38596600	-0.87195100
O	2.13277100	-0.95479800	-1.87492500
H	2.00894200	-2.52510300	1.49762100
H	2.03958000	-2.92118500	-0.24463000

Isomer 41_{RO}/41_{RC}/41_{RE}

Spartan Calculations

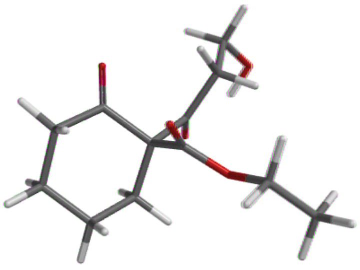

41$_{RO}$
$\Delta G°$ = 10.8 kcal/mol

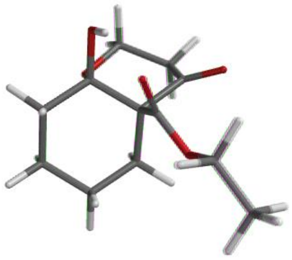

41$_{RC}$
$\Delta G°$ = 10.3 kcal/mol

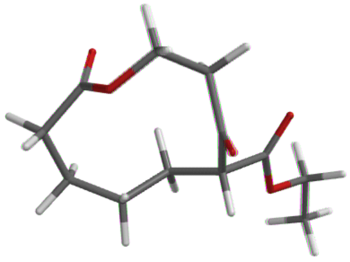

41$_{RE}$
$\Delta G°$ = 0.0 kcal/mol

41_{RO}	Calculated energy (au)	Calculated energy (kcal/mol)	Relative energy (kcal/mol)
M0003	-844.23995	-529760.5711	0.00
M0006	-844.23966	-529760.3885	0.18
M0007	-844.23937	-529760.2053	0.37
M0011	-844.23903	-529759.9932	0.58
M0005	-844.23898	-529759.9593	0.61
M0010	-844.23885	-529759.8771	0.69
M0016	-844.23546	-529757.7493	2.82
M0019	-844.23495	-529757.4305	3.14
M0020	-844.23485	-529757.3709	3.20
M0027	-844.23478	-529757.3213	3.25
M0021	-844.23473	-529757.2924	3.28
M0026	-844.23416	-529756.9335	3.64
M0033	-844.23409	-529756.8915	3.68
M0035	-844.23365	-529756.6147	3.96
M0031	-844.23349	-529756.5118	4.06
M0041	-844.23346	-529756.4930	4.08
M0038	-844.23321	-529756.3380	4.23
M0001	-844.23236	-529755.8040	4.77
M0004	-844.23207	-529755.6208	4.95
M0008	-844.23190	-529755.5154	5.06
M0002	-844.23181	-529755.4620	5.11
M0009	-844.23162	-529755.3409	5.23
M0012	-844.23149	-529755.2619	5.31
M0015	-844.23145	-529755.2336	5.34
M0014	-844.23116	-529755.0498	5.52
M0017	-844.23080	-529754.8264	5.74
M0013	-844.23072	-529754.7762	5.79
M0054	-844.23041	-529754.5816	5.99
M0067	-844.23028	-529754.5032	6.07
M0023	-844.23011	-529754.3959	6.18
M0065	-844.22990	-529754.2641	6.31

M0036	-844.22930	-529753.8839	6.69
M0044	-844.22925	-529753.8525	6.72
M0030	-844.22920	-529753.8243	6.75
M0046	-844.22877	-529753.5507	7.02
M0045	-844.22845	-529753.3549	7.22
M0018	-844.22829	-529753.2513	7.32
M0025	-844.22797	-529753.0505	7.52
M0022	-844.22795	-529753.0405	7.53
M0024	-844.22722	-529752.5793	7.99
M0061	-844.22715	-529752.5341	8.04
M0074	-844.22714	-529752.5285	8.04
M0029	-844.22704	-529752.4689	8.10
M0077	-844.22697	-529752.4218	8.15
M0028	-844.22686	-529752.3547	8.22
M0069	-844.22679	-529752.3101	8.26
M0068	-844.22651	-529752.1319	8.44
M0032	-844.22609	-529751.8740	8.70
M0050	-844.22600	-529751.8169	8.75
M0058	-844.22571	-529751.6305	8.94
M0097	-844.22539	-529751.4303	9.14
M0034	-844.22531	-529751.3839	9.19
M0085	-844.22523	-529751.3287	9.24
M0048	-844.22521	-529751.3212	9.25
M0051	-844.22521	-529751.3205	9.25
M0056	-844.22506	-529751.2277	9.34
M0062	-844.22503	-529751.2057	9.37
M0095	-844.22487	-529751.1059	9.47
M0055	-844.22485	-529751.0915	9.48
M0099	-844.22481	-529751.0702	9.50
M0060	-844.22481	-529751.0695	9.50
M0037	-844.22481	-529751.0689	9.50
M0098	-844.22478	-529751.0513	9.52
M0040	-844.22465	-529750.9647	9.61
M0080	-844.22380	-529750.4326	10.14
M0092	-844.22367	-529750.3529	10.22
M0052	-844.22351	-529750.2494	10.32
M0089	-844.22339	-529750.1772	10.39
M0087	-844.22317	-529750.0367	10.53
M0063	-844.22307	-529749.9752	10.60
M0081	-844.22299	-529749.9231	10.65
M0066	-844.22290	-529749.8716	10.70
M0090	-844.22277	-529749.7869	10.78
M0091	-844.22276	-529749.7813	10.79
M0079	-844.22267	-529749.7235	10.85
M0082	-844.22266	-529749.7217	10.85
M0043	-844.22258	-529749.6715	10.90

M0084	-844.22251	-529749.6275	10.94
M0093	-844.22235	-529749.5240	11.05
M0094	-844.22226	-529749.4694	11.10
M0049	-844.22216	-529749.4067	11.16
M0088	-844.22214	-529749.3954	11.18
M0039	-844.22199	-529749.3012	11.27
M0057	-844.22179	-529749.1745	11.40
M0042	-844.22138	-529748.9134	11.66
M0078	-844.22009	-529748.1065	12.46
M0083	-844.21999	-529748.0437	12.53
M0064	-844.21985	-529747.9527	12.62
M0047	-844.21981	-529747.9320	12.64
M0086	-844.21959	-529747.7934	12.78
M0072	-844.21954	-529747.7620	12.81
M0059	-844.21945	-529747.7036	12.87
M0053	-844.21935	-529747.6446	12.93
M0071	-844.21912	-529747.5003	13.07
M0075	-844.21694	-529746.1324	14.44
M0070	-844.21690	-529746.1054	14.47
M0076	-844.21678	-529746.0288	14.54
M0073	-844.21659	-529745.9077	14.66
M0100	-844.21482	-529744.8002	15.77
**41_{RC}**	**Calculated energy (au)**	**Calculated energy (kcal/mol)**	**Relative energy (kcal/mol)**
M0003	-844.23300	-529756.2100	0.00
M0006	-844.23191	-529755.5242	0.69
M0040	-844.23185	-529755.4827	0.73
M0048	-844.23135	-529755.1746	1.04
M0026	-844.23069	-529754.7599	1.45
M0060	-844.23055	-529754.6720	1.54
M0007	-844.23054	-529754.6632	1.55
M0013	-844.23042	-529754.5892	1.62
M0005	-844.23005	-529754.3532	1.86
M0028	-844.22982	-529754.2146	2.00
M0008	-844.22921	-529753.8312	2.38
M0016	-844.22879	-529753.5632	2.65
M0022	-844.22852	-529753.3963	2.81
M0055	-844.22848	-529753.3731	2.84
M0002	-844.22843	-529753.3379	2.87
M0001	-844.22839	-529753.3116	2.90
M0004	-844.22822	-529753.2055	3.00
M0027	-844.22817	-529753.1786	3.03
M0050	-844.22816	-529753.1723	3.04
M0019	-844.22798	-529753.0600	3.15
M0009	-844.22746	-529752.7305	3.48
M0033	-844.22744	-529752.7173	3.49
M0014	-844.22739	-529752.6853	3.52

M0038	-844.22723	-529752.5862	3.62
M0011	-844.22711	-529752.5090	3.70
M0015	-844.22698	-529752.4318	3.78
M0024	-844.22696	-529752.4149	3.80
M0012	-844.22681	-529752.3258	3.88
M0052	-844.22680	-529752.3195	3.89
M0036	-844.22645	-529752.0955	4.11
M0021	-844.22638	-529752.0541	4.16
M0062	-844.22633	-529752.0202	4.19
M0020	-844.22619	-529751.9349	4.28
M0010	-844.22606	-529751.8495	4.36
M0054	-844.22596	-529751.7874	4.42
M0079	-844.22593	-529751.7736	4.44
M0017	-844.22582	-529751.7039	4.51
M0071	-844.22582	-529751.6989	4.51
M0037	-844.22571	-529751.6349	4.58
M0023	-844.22557	-529751.5452	4.66
M0025	-844.22549	-529751.4925	4.72
M0044	-844.22530	-529751.3776	4.83
M0049	-844.22515	-529751.2810	4.93
M0035	-844.22512	-529751.2634	4.95
M0031	-844.22505	-529751.2201	4.99
M0039	-844.22504	-529751.2120	5.00
M0042	-844.22493	-529751.1455	5.06
M0018	-844.22485	-529751.0921	5.12
M0045	-844.22480	-529751.0620	5.15
M0070	-844.22457	-529750.9189	5.29
M0069	-844.22437	-529750.7909	5.42
M0041	-844.22436	-529750.7872	5.42
M0075	-844.22422	-529750.6955	5.51
M0043	-844.22420	-529750.6849	5.53
M0064	-844.22398	-529750.5481	5.66
M0051	-844.22392	-529750.5123	5.70
M0093	-844.22390	-529750.4966	5.71
M0061	-844.22343	-529750.2048	6.01
M0083	-844.22329	-529750.1151	6.09
M0046	-844.22314	-529750.0216	6.19
M0058	-844.22295	-529749.8986	6.31
M0047	-844.22284	-529749.8302	6.38
M0034	-844.22279	-529749.8007	6.41
M0063	-844.22265	-529749.7110	6.50
M0057	-844.22263	-529749.7028	6.51
M0098	-844.22225	-529749.4606	6.75
M0082	-844.22205	-529749.3383	6.87
M0056	-844.22191	-529749.2504	6.96
M0067	-844.22176	-529749.1569	7.05

M0094	-844.22165	-529749.0847	7.13
M0074	-844.22161	-529749.0603	7.15
M0066	-844.22143	-529748.9442	7.27
M0029	-844.22117	-529748.7810	7.43
M0030	-844.22098	-529748.6650	7.55
M0080	-844.22097	-529748.6593	7.55
M0095	-844.22096	-529748.6505	7.56
M0053	-844.22094	-529748.6367	7.57
M0065	-844.22080	-529748.5514	7.66
M0068	-844.22068	-529748.4736	7.74
M0032	-844.22047	-529748.3462	7.86
M0059	-844.22023	-529748.1918	8.02
M0089	-844.22004	-529748.0738	8.14
M0091	-844.21988	-529747.9734	8.24
M0099	-844.21950	-529747.7388	8.47
M0090	-844.21950	-529747.7363	8.47
M0088	-844.21947	-529747.7162	8.49
M0092	-844.21933	-529747.6315	8.58
M0078	-844.21914	-529747.5097	8.70
M0087	-844.21913	-529747.5041	8.71
M0073	-844.21870	-529747.2355	8.97
M0081	-844.21859	-529747.1621	9.05
M0076	-844.21831	-529746.9902	9.22
M0085	-844.21818	-529746.9086	9.30
M0077	-844.21813	-529746.8785	9.33
M0086	-844.21802	-529746.8050	9.40
M0072	-844.21782	-529746.6821	9.53
M0097	-844.21694	-529746.1292	10.08
M0084	-844.21637	-529745.7703	10.44
M0100	-844.21621	-529745.6711	10.54
M0096	-844.21578	-529745.4032	10.81
**41_{RE}**	**Calculated energy (au)**	**Calculated energy (kcal/mol)**	**Relative energy (kcal/mol)**
M0001	-844.25720	-529771.3949	0.00
M0022	-844.25709	-529771.3252	0.07
M0028	-844.25675	-529771.1075	0.29
M0029	-844.25623	-529770.7856	0.61
M0034	-844.25620	-529770.7649	0.63
M0032	-844.25573	-529770.4731	0.92
M0016	-844.25572	-529770.4643	0.93
M0021	-844.25563	-529770.4091	0.99
M0024	-844.25537	-529770.2466	1.15
M0025	-844.25529	-529770.1913	1.20
M0002	-844.25495	-529769.9799	1.42
M0027	-844.25494	-529769.9755	1.42
M0007	-844.25489	-529769.9435	1.45
M0017	-844.25475	-529769.8550	1.54

M0031	-844.25471	-529769.8286	1.57
M0006	-844.25431	-529769.5776	1.82
M0005	-844.25417	-529769.4904	1.90
M0015	-844.25417	-529769.4892	1.91
M0011	-844.25407	-529769.4289	1.97
M0010	-844.25388	-529769.3110	2.08
M0039	-844.25384	-529769.2865	2.11
M0045	-844.25381	-529769.2683	2.13
M0014	-844.25377	-529769.2375	2.16
M0004	-844.25369	-529769.1917	2.20
M0012	-844.25365	-529769.1629	2.23
M0019	-844.25345	-529769.0399	2.36
M0054	-844.25304	-529768.7832	2.61
M0048	-844.25297	-529768.7368	2.66
M0037	-844.25291	-529768.7004	2.69
M0067	-844.25261	-529768.5096	2.89
M0013	-844.25258	-529768.4958	2.90
M0046	-844.25250	-529768.4431	2.95
M0063	-844.25242	-529768.3942	3.00
M0043	-844.25240	-529768.3810	3.01
M0020	-844.25232	-529768.3283	3.07
M0053	-844.25223	-529768.2718	3.12
M0056	-844.25216	-529768.2317	3.16
M0059	-844.25211	-529768.1965	3.20
M0018	-844.25207	-529768.1714	3.22
M0047	-844.25203	-529768.1457	3.25
M0003	-844.25201	-529768.1388	3.26
M0042	-844.25192	-529768.0823	3.31
M0009	-844.25187	-529768.0478	3.35
M0057	-844.25180	-529768.0058	3.39
M0061	-844.25175	-529767.9725	3.42
M0051	-844.25173	-529767.9574	3.44
M0049	-844.25156	-529767.8545	3.54
M0052	-844.25145	-529767.7842	3.61
M0041	-844.25138	-529767.7397	3.66
M0026	-844.25122	-529767.6393	3.76
M0055	-844.25093	-529767.4598	3.94
M0030	-844.25091	-529767.4448	3.95
M0066	-844.25079	-529767.3695	4.03
M0008	-844.25071	-529767.3230	4.07
M0036	-844.25064	-529767.2753	4.12
M0060	-844.25059	-529767.2471	4.15
M0075	-844.25055	-529767.2170	4.18
M0033	-844.25043	-529767.1417	4.25
M0070	-844.25026	-529767.0407	4.35
M0038	-844.25011	-529766.9447	4.45

M0023	-844.24954	-529766.5870	4.81
M0050	-844.24954	-529766.5864	4.81
M0058	-844.24913	-529766.3291	5.07
M0062	-844.24899	-529766.2419	5.15
M0068	-844.24878	-529766.1107	5.28
M0084	-844.24855	-529765.9645	5.43
M0073	-844.24853	-529765.9494	5.45
M0077	-844.24840	-529765.8729	5.52
M0074	-844.24838	-529765.8610	5.53
M0085	-844.24802	-529765.6307	5.76
M0078	-844.24801	-529765.6250	5.77
M0097	-844.24794	-529765.5798	5.82
M0082	-844.24778	-529765.4832	5.91
M0035	-844.24775	-529765.4619	5.93
M0040	-844.24773	-529765.4493	5.95
M0086	-844.24755	-529765.3357	6.06
M0064	-844.24727	-529765.1626	6.23
M0044	-844.24709	-529765.0496	6.35
M0069	-844.24699	-529764.9887	6.41
M0065	-844.24655	-529764.7095	6.69
M0080	-844.24627	-529764.5319	6.86
M0071	-844.24555	-529764.0833	7.31
M0083	-844.24544	-529764.0111	7.38
M0087	-844.24540	-529763.9898	7.41
M0100	-844.24460	-529763.4865	7.91
M0090	-844.24424	-529763.2612	8.13
M0091	-844.24421	-529763.2405	8.15
M0076	-844.24415	-529763.2054	8.19
M0093	-844.24409	-529763.1640	8.23
M0095	-844.24377	-529762.9644	8.43
M0098	-844.24354	-529762.8207	8.57
M0088	-844.24309	-529762.5365	8.86
M0072	-844.24305	-529762.5158	8.88
M0079	-844.24305	-529762.5132	8.88
M0081	-844.24299	-529762.4769	8.92
M0092	-844.24297	-529762.4618	8.93
M0096	-844.24246	-529762.1437	9.25
M0094	-844.24238	-529762.0922	9.30
M0089	-844.24232	-529762.0533	9.34

Table S16. Energies calculated at DFT/B3LYP/6-31G* in vacuum for the conformers found for **41_{RO}/41_{RC}/41_{RE}**.

Isomer	ΔG° (au)	ΔG° (kcal/mol)	ΔG° (kcal/mol)
**41$_{RO}$**	-843.996996	-529608.11	10.8
**41$_{RC}$**	-843.997766	-529608.60	10.3
**41$_{RE}$**	-844.014157	-529618.88	0.0

Table S17. Relative free energies (ΔG°) of the lowest energy geometries of **41$_{RO}$/41$_{RC}$/41$_{RE}$** at DFT/B3LYP/6-31G* in vacuum in kcal/mol.

XYZ Coordinates

41RO

C	-1.19864800	-1.37499200	-0.66492000
C	-0.47245900	-0.31080100	0.15385000
O	-0.59239600	-2.28870100	-1.22923000
C	0.96113200	-0.77541900	0.44952000
O	1.30342200	-1.16177800	1.57288000
C	-0.43954100	1.01759900	-0.57887000
O	-1.01196100	1.24607800	-1.63556000
O	0.30837800	1.93356000	0.09836000
C	0.39301600	3.22228000	-0.52618000
H	-0.60933500	3.65638900	-0.61087000
C	1.97763200	-0.72861700	-0.68539000
H	1.46374200	-0.84199800	-1.64385000
H	2.46130000	0.25344300	-0.65797000
C	3.00268300	-1.84201600	-0.53715000
H	3.60748300	-1.91600500	-1.44596000
H	2.53251400	-2.81446700	-0.35726000
H	0.83880600	3.12096100	-1.52189000
C	1.26629500	4.10862200	0.33961000
H	2.26502500	3.67373300	0.45177000
H	0.84438400	4.20041100	1.34592000
H	1.36233300	5.10707200	-0.09590000
O	3.88909300	-1.55675500	0.54009000
H	3.33800300	-1.54800500	1.35041000
C	-1.24383900	-0.13067200	1.48576000
H	-1.13157800	-1.02965200	2.10765000
H	-0.82162100	0.69661900	2.07034000
C	-2.74056000	0.11633600	1.28991000
H	-3.22477000	0.21053500	2.26869000
H	-2.90527100	1.06243600	0.76129000
C	-3.37764800	-1.03239500	0.51859000
H	-3.30345700	-1.95099500	1.11419000
H	-4.44466800	-0.83350700	0.36891000
C	-2.70146800	-1.24462400	-0.82513000
H	-3.08693600	-2.16013500	-1.28744000
H	-2.91779900	-0.41788400	-1.50798000

41$_{RC}$

C	1.38906900	0.37918200	0.62928000
C	0.19317000	-0.17440000	-0.18201000
C	0.45711200	-1.65549000	-0.41565000
O	-0.43071700	-2.50644100	-0.32644000
C	-1.09711000	0.01481800	0.60819000
O	-1.13599100	0.26574800	1.80944000
O	-2.20207000	-0.12301300	-0.17519000
C	-3.44978000	-0.02329500	0.52217000
H	-3.53753200	0.96824500	0.97995000
C	1.83189200	-2.05386800	-0.89510000
H	1.95602400	-3.13752800	-0.80420000
H	-3.50284900	-0.79544500	1.29773000
C	-4.56810000	-0.22553600	-0.48112000
H	-4.48032900	-1.20453600	-0.96379000
H	-4.51413100	0.52667400	-1.27510000
H	-5.54543000	-0.15728700	0.00467000
O	2.62318000	0.06356300	-0.04201000
O	1.45471000	-0.14232800	1.95407000
H	0.64680900	0.15115100	2.42543000
H	1.90820200	-1.78833800	-1.95421000
C	2.88340100	-1.33758600	-0.08067000
C	1.35436700	1.91758200	0.73006000
H	2.27017700	2.27354300	1.21998000
H	0.52905700	2.24272000	1.37501000
C	1.23449600	2.59676100	-0.63181000
H	2.15800700	2.44414300	-1.20354000
H	1.12554500	3.67858100	-0.49430000
C	0.05121700	2.05972000	-1.42716000
H	0.04773600	2.51126000	-2.42590000
H	-0.88525300	2.36100900	-0.94301000
C	0.10725900	0.53729000	-1.55705000
H	-0.76379100	0.19108900	-2.12714000
H	0.97545900	0.28242100	-2.17765000
H	2.95187200	-1.74074600	0.93605000
H	3.86557200	-1.47825500	-0.54378000

41$_{RE}$

C	2.97237000	0.35611900	-0.70102100
C	2.00334100	2.47679000	0.28666900
C	0.91544100	1.70849000	1.05466900
O	2.85268000	-0.63890100	0.21724900
C	3.26551100	1.65734900	0.00214900
H	2.30489100	3.34434000	0.88678900
H	1.59625100	2.87507000	-0.65061100
H	0.38569100	2.41193000	1.70982900
O	2.77761000	0.24015900	-1.90138100
H	1.37293000	0.96686000	1.71805900
C	-0.12497000	1.07749000	0.11501900
H	-0.88185900	1.84290000	-0.10304100
H	0.31761000	0.84384000	-0.85722100
C	-0.80114000	-0.17066000	0.70340900
C	2.35316000	-1.90607000	-0.24269100
H	2.86826000	-2.20668100	-1.16237100
H	2.61351900	-2.64186100	0.52597900
C	0.84387000	-1.86753000	-0.44398100
H	0.46025900	-2.86877000	-0.66705100
H	0.59163000	-1.23369000	-1.29703100
C	0.14693000	-1.35970000	0.80655900
O	0.33843000	-1.90709000	1.89692900
H	3.80208100	1.44750900	0.93554900
H	3.95993100	2.23150900	-0.62171100
H	-1.13915000	0.04553100	1.72631900
C	-2.03249000	-0.52847900	-0.09604100
O	-2.12241000	-1.48182900	-0.85711100
O	-3.02751000	0.37313100	0.14137900
C	-4.24135000	0.11768100	-0.57691100
H	-4.04508000	0.14339100	-1.65460100
H	-4.63682000	-0.86477800	-0.29595100
C	-5.24209000	1.19677200	-0.21266100
H	-5.43240000	1.19784200	0.86570900
H	-6.18881000	1.04458200	-0.73841100
H	-4.85082900	2.18719200	-0.46782100

Gaussian Calculations

41$_{RO}$

M06-2X/6-31G* with Solvent Correction

SCF Done: E(RM062X) = -843.900006954

Zero-point correction= 0.297990
Thermal correction to Gibbs Free Energy= 0.249943

C	-0.92817400	-1.59281100	-0.64927400
C	-0.39468200	-0.41430000	0.19123200
O	-0.17476600	-2.44987500	-1.05703500
C	1.10253400	-0.66457100	0.44886900
O	1.47035900	-1.08973100	1.52365500
C	-0.53533300	0.87169800	-0.61945200
O	-0.88118100	0.92717100	-1.77673400
O	-0.18423200	1.93140400	0.10738300
C	-0.22054700	3.20470400	-0.57256500
H	-1.23929400	3.37394100	-0.93086100
C	2.06460700	-0.38479400	-0.68289400
H	1.57868000	-0.61754000	-1.63590800
H	2.27333800	0.69480100	-0.67491900
C	3.37029200	-1.15856100	-0.51863200
H	4.00816200	-0.98639000	-1.38892200
H	3.14339800	-2.23315700	-0.47487200
H	0.44187400	3.14847400	-1.44086400
C	0.22217300	4.25369900	0.42161900
H	1.23662600	4.04879000	0.77212200
H	-0.44926600	4.27603300	1.28341400
H	0.21061000	5.23753400	-0.05373500
O	4.09935200	-0.73814300	0.61493900
H	3.53168500	-0.93734800	1.37668700
C	-1.18966900	-0.33023900	1.50985600
H	-0.96029200	-1.22788000	2.09354400
H	-0.82792000	0.52803500	2.08201800
C	-2.69279100	-0.24417700	1.24718900
H	-3.22406600	-0.16736100	2.20063600
H	-2.92429000	0.66956300	0.68173100
C	-3.17774200	-1.47057300	0.47335800
H	-3.02025900	-2.36725000	1.08495200
H	-4.25061400	-1.40389100	0.27174000
C	-2.42321900	-1.62569700	-0.85860500
H	-2.67296400	-2.55962900	-1.36704000
H	-2.67637600	-0.78960900	-1.52159600

41$_{RC}$

M06-2X/6-31G* with Solvent Correction

SCF Done: E(RM062X) = -843.919382712

Zero-point correction= 0.301281
Thermal correction to Gibbs Free Energy= 0.258808

C	1.40894800	0.38210700	0.64543900
C	0.20222800	-0.15783600	-0.19858000
C	0.47764000	-1.64030200	-0.47435300
O	-0.34006700	-2.49994800	-0.22922100
C	-1.09830500	-0.09810200	0.59674900
O	-1.16356300	-0.08532800	1.81193900
O	-2.16992500	-0.09109800	-0.18159700
C	-3.44539600	-0.12799000	0.49151700
H	-3.51503100	0.74355800	1.14766600
C	1.84203700	-1.92988400	-1.05584400
H	2.00975500	-3.00888500	-1.03585900
H	-3.47797600	-1.02733100	1.11174200
C	-4.51272100	-0.12784200	-0.57910500
H	-4.40845900	-1.00212400	-1.22605000
H	-4.44332300	0.77387700	-1.19266700
H	-5.50066300	-0.15566100	-0.11291600
O	2.59031300	0.18452300	-0.08608100
O	1.53065000	-0.33672400	1.84463600
H	0.65139700	-0.32037000	2.26796800
H	1.84615400	-1.59889200	-2.10086900
C	2.93328200	-1.18164300	-0.27729900
C	1.28328600	1.88230400	0.88941600
H	2.17518100	2.19329500	1.44174500
H	0.41596500	2.04154600	1.54154700
C	1.12077700	2.67042000	-0.41171200
H	2.04064600	2.58074700	-1.00082300
H	0.98255300	3.73235300	-0.18563700
C	-0.05834300	2.14203700	-1.23129000
H	-0.13582200	2.68347700	-2.17951800
H	-0.99872700	2.31558900	-0.69019300
C	0.10013700	0.64646300	-1.51494400
H	-0.73306800	0.26484300	-2.11013300
H	1.02118900	0.49256200	-2.08480600
H	3.10086200	-1.66139600	0.69007800
H	3.86868800	-1.17698800	-0.83865400

41_{RE}

M06-2X/6-31G* with Solvent Correction

SCF Done: E(RM062X) = -843.921486735

Zero-point correction= 0.300324
Thermal correction to Gibbs Free Energy= 0.254976

C	2.76751200	0.43202200	-0.84305100
C	2.01037000	2.44843400	0.48447700
C	1.01929100	1.56790700	1.26390900
O	2.77044100	-0.64011100	-0.03133100
C	3.20928200	1.66887200	-0.09781900
H	2.41873400	3.22954800	1.13179500
H	1.48262800	2.95915500	-0.32956900
H	0.56979000	2.14046700	2.08158800
O	2.40635000	0.39280400	-1.99726200
H	1.55742600	0.73401100	1.73080900
C	-0.10634600	1.04406000	0.35747900
H	-0.87731200	1.81338600	0.25642200
H	0.27160400	0.86365400	-0.65558900
C	-0.76855600	-0.24505500	0.88393800
C	2.24169500	-1.86986400	-0.55377900
H	2.63757100	-2.04225100	-1.55626900
H	2.60480000	-2.63384900	0.13292100
C	0.71933100	-1.83703800	-0.55983100
H	0.33639800	-2.84284900	-0.76117800
H	0.34740400	-1.18334800	-1.35077600
C	0.20562100	-1.41585700	0.81254100
O	0.60955000	-1.95625000	1.81821100
H	3.87330400	1.36399000	0.71462400
H	3.76398800	2.30313400	-0.79293700
H	-1.04781400	-0.10794100	1.93562600
C	-2.02889700	-0.59710000	0.11457300
O	-2.23230100	-1.66907900	-0.41032800
O	-2.90053000	0.41178400	0.10086800
C	-4.14176900	0.16197700	-0.58783000
H	-3.91632100	-0.10005800	-1.62503900
H	-4.63140600	-0.69639700	-0.12001300
C	-4.97196700	1.42149700	-0.48352500
H	-5.17302600	1.66528500	0.56253900
H	-5.92618700	1.27721800	-0.99623900
H	-4.45306700	2.26453500	-0.94646500

Isomer $42_{RO}/42_{RC}/42_{RE}$

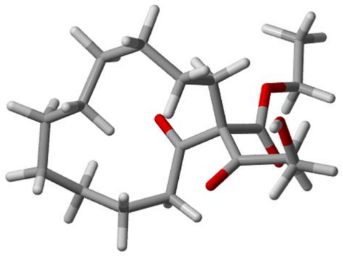

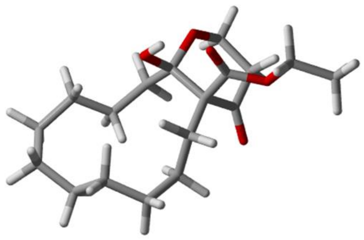

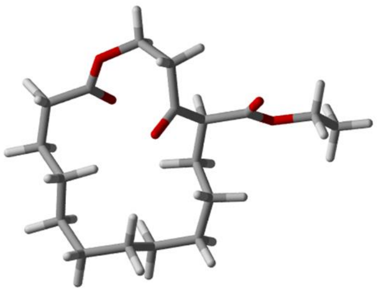

42_{RO}	42_{RC}	42_{RE}
ΔG ° = 35.2 kcal/mol	ΔG ° = 39.8 kcal/mol	ΔG ° = 0.0 kcal/mol

Gaussian Calculations

42_{RO}

B3LYP/6-31G*

SCF Done: E(RB3LYP) = -1080.09006732

Zero-point correction=   0.467369
Thermal correction to Gibbs Free Energy=   0.410885

H	3.70678800	-1.38987300	-1.41471300
C	3.20518400	-0.43156100	-1.21338000
H	2.50712700	-0.63363300	-0.39957300
C	2.39497500	0.00717800	-2.44167700
H	3.06865200	0.16839400	-3.29555600
H	1.94072800	0.98166400	-2.22192600
C	1.29869300	-0.99342800	-2.85778700
H	0.76477400	-0.59778900	-3.73370900
H	1.77658000	-1.92597000	-3.19099900
C	0.28606900	-1.35389600	-1.75306200
H	0.83467700	-1.71606400	-0.87979700
H	-0.31265800	-2.21134000	-2.07850100
C	-0.68085800	-0.20784400	-1.34966700
C	4.23758100	0.60567800	-0.73174100
H	3.87167700	1.61488700	-0.96660900
H	5.16937300	0.48355800	-1.30120400
H	-0.35364600	0.74809900	-1.76500900
C	4.55254400	0.55667700	0.77886600
H	4.79537600	-0.47238500	1.08289800
H	5.46677000	1.14080600	0.94873500
C	3.43969700	1.13695100	1.68794200
C	-0.83622200	0.03560400	0.18764800
C	0.38218300	0.88528800	0.72809700
C	2.34679900	0.14922900	2.17923200
C	0.90351800	0.72397800	2.14816100
H	0.90776800	1.72754300	2.59172400

H	0.23846600	0.09541700	2.73997700
H	2.35848400	-0.78401700	1.61032700
H	2.55308800	-0.14198000	3.21637900
H	3.90950300	1.58416700	2.57363300
H	2.96487900	1.96516500	1.15183000
C	-1.03727700	-1.32040500	0.88457200
O	-0.09693800	-1.92910400	1.36084200
C	-2.44294600	-1.92461600	0.89614600
H	-3.06861500	-1.48420800	0.11175300
H	-2.90068500	-1.64860200	1.85462900
C	-2.42266600	-3.43769400	0.74035800
H	-1.67814100	-3.85911500	1.42751000
H	-3.41030300	-3.84854400	1.00056800
O	0.83239500	1.74440900	-0.00667300
C	-2.01882100	0.95160700	0.54227700
O	-2.47118400	1.02152000	1.66995400
O	-2.45027500	1.69229900	-0.48572900
C	-3.49891100	2.65065300	-0.18790500
H	-3.14433200	3.31463000	0.60619900
H	-4.36832700	2.10706000	0.19473900
C	-3.80520700	3.39877900	-1.47050700
H	-4.59377900	4.13675400	-1.28760000
H	-4.14893500	2.71359200	-2.25188600
H	-2.91800600	3.92436300	-1.83688400
O	-2.09743200	-3.72101800	-0.61986300
H	-1.82127600	-4.64752900	-0.67731200
H	-1.67455400	-0.40297000	-1.76331000

M06-2X/6-31G* with Solvent Correction

SCF Done: E(RM062X) = -1079.63587033

Zero-point correction= 0.472627
Thermal correction to Gibbs Free Energy= 0.418080

H	3.49934100	-1.32359800	-1.26453500
C	2.99627400	-0.35168100	-1.15664000
H	2.26231500	-0.48558900	-0.35833500
C	2.26104300	0.01095300	-2.44604300
H	2.98960700	0.16270700	-3.25368500
H	1.76538300	0.97716000	-2.29086300
C	1.23232900	-1.03573000	-2.88672100
H	0.70115400	-0.67202500	-3.77591800
H	1.75851900	-1.94968800	-3.19159700
C	0.22027400	-1.41225800	-1.79890100
H	0.76597300	-1.80003900	-0.93450200
H	-0.38979200	-2.25207700	-2.14312000
C	-0.72226300	-0.25892100	-1.38131200
C	4.00949100	0.71465000	-0.73545600
H	3.58354300	1.70686600	-0.93876000
H	4.90079600	0.62746700	-1.37009100
H	-0.41772100	0.68561500	-1.84061900
C	4.41901900	0.66655900	0.74318700
H	4.66727600	-0.36416600	1.03315800
H	5.34226700	1.24596000	0.86017900
C	3.36514500	1.24804100	1.70440300
C	-0.80452400	0.01688300	0.14341300
C	0.37906100	0.91263700	0.63166200
C	2.26682700	0.27755300	2.19783900
C	0.83611100	0.84682400	2.07563300
H	0.82691000	1.87583900	2.45284100
H	0.13685300	0.26011700	2.67192000
H	2.31132600	-0.68094600	1.67414400
H	2.42978100	0.04414600	3.25490700
H	3.87888100	1.65163800	2.58400300
H	2.89691100	2.10577800	1.20928500
C	-0.91799100	-1.31779600	0.88047800
O	0.06176100	-1.85297900	1.35275100
C	-2.28328100	-1.98688300	0.92316700
H	-2.91803200	-1.61546500	0.11043600
H	-2.76174500	-1.69008300	1.86347800
C	-2.16279400	-3.49475100	0.83596300
H	-1.45518500	-3.84446500	1.59643400
H	-3.14010500	-3.95594200	1.02911700
O	0.83868700	1.72716100	-0.13900100
C	-2.00336500	0.88788800	0.52543300

O	-2.45714900	0.91048500	1.64905400
O	-2.43674500	1.64952800	-0.47120200
C	-3.51071000	2.55797400	-0.14823200
H	-3.17417400	3.21417300	0.65876200
H	-4.35821100	1.97319800	0.21897900
C	-3.84242900	3.32153300	-1.40997900
H	-4.65361500	4.02596900	-1.21012800
H	-4.16180200	2.63873000	-2.20106900
H	-2.97252100	3.88211700	-1.76026100
O	-1.70394900	-3.79414200	-0.47147600
H	-1.42849400	-4.72093100	-0.49386300
H	-1.73557500	-0.46373000	-1.73868000

42$_{RC}$

B3LYP/6-31G*

SCF Done: E(RB3LYP) = -1080.09108377

Zero-point correction= 0.470843
Thermal correction to Gibbs Free Energy= 0.419160

H	4.84333600	2.28135200	-0.69160400
C	4.17667600	1.47343100	-0.36110200
H	3.90411700	0.93219700	-1.27596500
C	2.93506900	2.11621400	0.29073300
H	3.28982000	2.89019800	0.98479600
H	2.43657100	1.37393300	0.92568400
C	1.91085200	2.73997300	-0.68374500
H	1.40103600	3.57379700	-0.18008700
H	2.44324200	3.18796900	-1.53421700
C	0.82609000	1.77787300	-1.22078400
H	1.28390300	0.92655000	-1.72790700
H	0.24320800	2.28460900	-1.99389900
C	-0.12426500	1.34224900	-0.08122300
C	4.94614100	0.53297800	0.60063900
H	4.77313500	0.86991800	1.63330900
H	6.02426900	0.65409300	0.43026300
H	0.42501400	1.28231100	0.86268700
C	4.65574400	-0.98313600	0.51277500
H	5.00022400	-1.35037200	-0.46628200
H	5.30337900	-1.47083500	1.25430100
C	3.20864900	-1.47437500	0.74045400
C	-0.95128200	0.01383900	-0.14003600
C	-0.20427900	-1.30955900	0.38397100
C	2.32504600	-1.29710000	-0.50349300
C	0.91248900	-1.90635400	-0.49898700
H	0.98094800	-2.95375500	-0.17757000
H	0.56422800	-1.92138800	-1.53860200
H	2.26445300	-0.24282300	-0.75058800
H	2.84912700	-1.76446300	-1.35044100
H	3.24139000	-2.54292200	0.99711500
H	2.76111400	-0.97255700	1.60392100
C	-1.57289900	-0.31856900	-1.50981700
O	-1.37755100	0.34871200	-2.50592000
C	-2.07786100	0.29729700	0.87920800
O	-1.96765500	0.20270100	2.08976200
O	-3.18185800	0.78760500	0.29560100
C	-4.25068300	1.21741800	1.18277300
H	-3.84975700	1.97668600	1.86061300
H	-4.56424400	0.36081900	1.78662000

C	-5.37102900	1.75471700	0.31413600
H	-6.19527600	2.09776900	0.94868100
H	-5.75456200	0.98081000	-0.35834400
H	-5.02695500	2.59877300	-0.29152000
O	0.31046700	-1.12882800	1.65402700
H	-0.41901600	-0.76733400	2.20259500
O	-1.26580700	-2.29288800	0.50328600
C	-1.85246000	-2.73052200	-0.71606500
H	-2.63722700	-3.43412400	-0.42460300
H	-1.12514500	-3.28529700	-1.32245000
C	-2.44715300	-1.56830200	-1.53387500
H	-2.61530700	-1.85486000	-2.57620500
H	-3.41432400	-1.28197100	-1.10500100
H	-0.84052700	2.16406200	0.04727900

M06-2X/6-31G* with Solvent Correction

SCF Done: E(RM062X) = -1079.64619751

Zero-point correction= 0.476450
Thermal correction to Gibbs Free Energy= 0.425008

H	4.81794000	-2.15580800	0.59687900
C	4.04199000	-1.46809700	0.24155200
H	3.72652300	-0.91123500	1.12976200
C	2.86121800	-2.29774500	-0.29413100
H	3.25661800	-3.17189800	-0.82506600
H	2.33299700	-1.71534600	-1.05977100
C	1.86276300	-2.76795800	0.77579800
H	1.29360000	-3.62027500	0.38121400
H	2.41924400	-3.14863400	1.64179800
C	0.84838500	-1.71229900	1.25919100
H	1.36107600	-0.83825700	1.66745500
H	0.27470700	-2.12874400	2.08932400
C	-0.12632900	-1.34519700	0.12363800
C	4.64355000	-0.49544000	-0.80681400
H	4.08592500	-0.58529800	-1.74943200
H	5.66792400	-0.80559300	-1.03951800
H	0.39353900	-1.35043500	-0.84014700
C	4.67652900	0.99080500	-0.40548800
H	5.07410500	1.07475800	0.61607300
H	5.38948400	1.51180400	-1.05552100
C	3.32944400	1.72367800	-0.48140600
C	-0.92841500	-0.01453200	0.13438500
C	-0.16899500	1.27991400	-0.37642700
C	2.27950100	1.07358800	0.41371800
C	0.93715300	1.80068600	0.54794800
H	1.05679000	2.86385000	0.30399400
H	0.59469800	1.75156500	1.58789400
H	2.09581200	0.07506300	0.02343300
H	2.70995900	0.95074900	1.41507400
H	3.47516400	2.77119400	-0.18578400
H	2.96331100	1.73151300	-1.51523600
C	-1.57378700	0.33521500	1.48062100
O	-1.43282300	-0.34824000	2.46916000
C	-2.04555200	-0.29195600	-0.88215500
O	-1.92698700	-0.19148200	-2.08619500
O	-3.14604800	-0.76474500	-0.30382500
C	-4.21911100	-1.15674500	-1.18805200
H	-3.84729000	-1.94510000	-1.84750600
H	-4.48962800	-0.29479800	-1.80296300
C	-5.36237100	-1.62547600	-0.31725600
H	-6.19888200	-1.93727600	-0.94739800

H	-5.70320500	-0.82053900	0.33881600
H	-5.05529800	-2.47373100	0.29906800
O	0.39755500	1.08137500	-1.62143900
H	-0.30923500	0.73323600	-2.19974400
O	-1.18623700	2.27467700	-0.53209100
C	-1.75951600	2.73891700	0.67840600
H	-2.51120000	3.47489400	0.38832500
H	-1.00849300	3.25357100	1.28797800
C	-2.40998500	1.60266300	1.47905800
H	-2.60122600	1.90007600	2.51242400
H	-3.36839800	1.33864300	1.01759500
H	-0.85908400	-2.16076900	0.07058000

42RE

B3LYP/6-31G*

SCF Done: E(RB3LYP) = -1080.14870832

Zero-point correction= 0.469166
Thermal correction to Gibbs Free Energy= 0.413411

H	4.00066800	2.67250700	1.57169200
C	3.48435500	2.82199900	0.61256900
H	3.84658200	3.78313700	0.21958300
C	1.97111100	2.92712000	0.88427100
H	1.80934400	3.69368300	1.65617700
H	1.61324500	1.98575900	1.31658600
C	-1.47651900	-0.45717700	-0.16193500
C	3.91253300	1.70299700	-0.35654300
H	5.00120100	1.76366600	-0.50016900
H	3.47158600	1.88034700	-1.34737300
C	3.54173700	0.28808400	0.10871800
H	4.00811200	0.08917200	1.08539200
H	2.46073700	0.22586900	0.27347100
C	3.95693400	-0.80544500	-0.88575300
C	3.43869600	-2.19836100	-0.50047200
H	3.89790400	-2.96776400	-1.13779000
H	3.69670200	-2.45812000	0.53170900
H	3.57699600	-0.55921700	-1.88448600
H	5.05134500	-0.84566500	-0.96271200
C	-0.58093500	-1.14527300	0.88177900
O	0.28373900	-0.54405200	1.49001700
C	-2.90832500	-0.39440400	0.36164600
O	-3.27573900	-0.79416100	1.44841200
O	-3.73550000	0.15664600	-0.54788700
C	-5.12455300	0.27922500	-0.15338400
H	-5.17614900	0.86430700	0.76994700
H	-5.51570000	-0.71967700	0.06498600
C	-5.86440800	0.94619600	-1.29711900
H	-6.92327600	1.05636000	-1.03896600
H	-5.79303000	0.34886100	-2.21169100
H	-5.45424100	1.94024100	-1.50152200
O	1.42483200	-3.27449500	0.15177600
C	0.00300100	-3.50047600	0.07670700
H	-0.12768000	-4.55842200	0.31616000
H	-0.33681500	-3.31575300	-0.94497200
C	-0.76120900	-2.64532400	1.09934400
H	-1.82149300	-2.91477500	1.04680200
H	-0.39316100	-2.87529500	2.10330000
C	1.93816400	-2.33202500	-0.67499100

O	1.26681400	-1.70679600	-1.47206500
C	1.12968300	3.28749800	-0.34976200
H	1.46597300	4.25996400	-0.73823200
H	1.32139300	2.56677000	-1.15463600
C	-0.38689500	3.36672000	-0.08891900
H	-0.88280900	3.70782100	-1.00957400
H	-0.57836000	4.14410000	0.66451200
C	-1.05357600	2.06013400	0.38456200
H	-2.11603400	2.26775200	0.57504700
H	-0.62159900	1.74462400	1.33798000
C	-0.93996200	0.91468600	-0.63321900
H	-1.47808300	1.18853700	-1.54853200
H	0.10335000	0.75301100	-0.91761000
H	-1.49887300	-1.11598800	-1.04209300

M06-2X/6-31G* with Solvent Correction

SCF Done: E(RM062X) = -1079.68972168

Zero-point correction= 0.474169
Thermal correction to Gibbs Free Energy= 0.419974

H	3.85611900	2.59401200	1.63532300
C	3.35912100	2.80243600	0.67887700
H	3.71254600	3.78963300	0.35289200
C	1.84483200	2.85971600	0.90959200
H	1.63087000	3.58071300	1.71025900
H	1.49350400	1.88602700	1.27252800
C	-1.37789600	-0.50396600	-0.17956700
C	3.80297800	1.74658000	-0.34112500
H	4.89739400	1.77252500	-0.42684200
H	3.41486500	1.99600900	-1.33750000
C	3.36073800	0.33281100	0.03162600
H	3.72640300	0.09293900	1.04094700
H	2.26675900	0.28650800	0.08684300
C	3.84809200	-0.73122500	-0.94946100
C	3.38280800	-2.13118900	-0.55506400
H	3.82469700	-2.89423800	-1.20816900
H	3.68098800	-2.38066100	0.46776900
H	3.47750400	-0.50246700	-1.95532800
H	4.94304700	-0.72338900	-0.99933000
C	-0.49427800	-1.15724200	0.88014000
O	0.38747800	-0.55025500	1.44926200
C	-2.79149100	-0.39354200	0.35895300
O	-3.12786300	-0.67453000	1.48779500
O	-3.62857500	0.08151000	-0.56665800
C	-4.98637100	0.29208500	-0.13519600
H	-4.97638800	0.96975300	0.72321500
H	-5.40050800	-0.66485100	0.19428200
C	-5.74436900	0.86936100	-1.30957900
H	-6.78462400	1.04762700	-1.02608700
H	-5.72832800	0.17877900	-2.15638400
H	-5.30202600	1.81808100	-1.62355000
O	1.42393400	-3.25468300	0.14314600
C	0.01355600	-3.50187900	0.11584700
H	-0.10041800	-4.55500800	0.37429800
H	-0.36584800	-3.33310800	-0.89424500
C	-0.71072200	-2.63445500	1.14886800
H	-1.77731500	-2.87685100	1.11958500
H	-0.31963700	-2.85739800	2.14439100
C	1.88455200	-2.28379400	-0.66444800
O	1.16935800	-1.64181800	-1.40192800
C	1.05501300	3.26266700	-0.33607900

H	1.40278300	4.24731500	-0.67646600
H	1.27332000	2.56804600	-1.15658900
C	-0.45938300	3.32090900	-0.10752500
H	-0.94948300	3.64141400	-1.03634500
H	-0.67885900	4.09168400	0.64246400
C	-1.08231600	1.99667900	0.35100900
H	-2.16336600	2.14697800	0.47683400
H	-0.68834400	1.71067800	1.33138900
C	-0.84717300	0.85984900	-0.64494600
H	-1.32125000	1.11081900	-1.60055600
H	0.22144800	0.72544800	-0.83857500
H	-1.40666400	-1.18361900	-1.04152200

Isomer 53_{RE}/53_{RC}/53_{RE}

Spartan Calculations

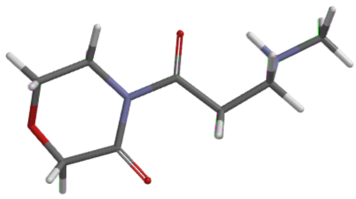

53_{RO}
ΔG ° = 0.00 kcal/mol

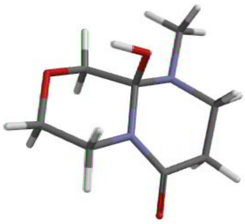

53_{RC}
ΔG ° = 9.7 kcal/mol

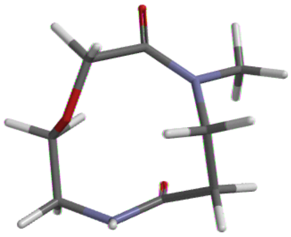

53_{RE}
ΔG ° = 0.04 kcal/mol

53_{RO}	Calculated energy (au)	Calculated energy (kcal/mol)	Relative energy (kcal/mol)
M0021	-648.44584	-406899.7659	0.00
M0022	-648.44584	-406899.7659	0.00
M0016	-648.44518	-406899.3523	0.41
M0003	-648.44324	-406898.1306	1.64
M0004	-648.44324	-406898.1306	1.64
M0043	-648.44275	-406897.8244	1.94
M0001	-648.44262	-406897.7447	2.02
M0002	-648.44262	-406897.7447	2.02
M0038	-648.44237	-406897.5884	2.18
M0039	-648.44237	-406897.5884	2.18
M0006	-648.44173	-406897.1824	2.58
M0007	-648.44173	-406897.1824	2.58
M0008	-648.44141	-406896.9848	2.78
M0009	-648.44141	-406896.9848	2.78
M0010	-648.44098	-406896.7150	3.05
M0011	-648.44098	-406896.7150	3.05
M0005	-648.44090	-406896.6648	3.10
M0045	-648.44089	-406896.6585	3.11
M0014	-648.44086	-406896.6371	3.13
M0015	-648.44086	-406896.6371	3.13
M0052	-648.44082	-406896.6127	3.15
M0020	-648.44080	-406896.6045	3.16
M0012	-648.44076	-406896.5782	3.19
M0013	-648.44076	-406896.5782	3.19
M0027	-648.44067	-406896.5204	3.25
M0033	-648.44044	-406896.3767	3.39
M0037	-648.44044	-406896.3736	3.39
M0030	-648.44027	-406896.2669	3.50
M0031	-648.44027	-406896.2669	3.50

M0041	-648.44009	-406896.1533	3.61
M0042	-648.43951	-406895.7900	3.98
M0018	-648.43941	-406895.7317	4.03
M0019	-648.43941	-406895.7317	4.03
M0024	-648.43927	-406895.6400	4.13
M0025	-648.43927	-406895.6400	4.13
M0029	-648.43919	-406895.5886	4.18
M0034	-648.43913	-406895.5560	4.21
M0035	-648.43913	-406895.5560	4.21
M0044	-648.43912	-406895.5484	4.22
M0028	-648.43874	-406895.3106	4.46
M0017	-648.43869	-406895.2805	4.49
M0036	-648.43860	-406895.2190	4.55
M0023	-648.43842	-406895.1073	4.66
M0046	-648.43834	-406895.0558	4.71
M0047	-648.43834	-406895.0558	4.71
M0026	-648.43833	-406895.0527	4.71
M0032	-648.43806	-406894.8820	4.88
M0040	-648.43777	-406894.7000	5.07
M0050	-648.43754	-406894.5582	5.21
M0051	-648.43754	-406894.5582	5.21
M0048	-648.43647	-406893.8843	5.88
M0049	-648.43624	-406893.7393	6.03
M0053	-648.43561	-406893.3440	6.42
M0054	-648.43561	-406893.3440	6.42
**53_{RC}**	**Calculated energy (au)**	**Calculated energy (kcal/mol)**	**Relative energy (kcal/mol)**
M0001	-648.43407	-406892.3758	0.00
M0004	-648.42986	-406889.7397	2.64
M0003	-648.42938	-406889.4366	2.94
M0006	-648.42929	-406889.3788	3.00
M0002	-648.42897	-406889.1780	3.20
M0005	-648.42860	-406888.9459	3.43
M0008	-648.42483	-406886.5802	5.80
M0011	-648.42449	-406886.3662	6.01
M0012	-648.42381	-406885.9383	6.44
M0013	-648.42381	-406885.9383	6.44
M0009	-648.42356	-406885.7833	6.59
M0014	-648.42336	-406885.6553	6.72
M0007	-648.42311	-406885.4984	6.88
M0010	-648.42083	-406884.0683	8.31
M0016	-648.42021	-406883.6805	8.70
M0015	-648.41936	-406883.1484	9.23
**53_{RE}**	**Calculated energy (au)**	**Calculated energy (kcal/mol)**	**Relative energy (kcal/mol)**
M0001	-648.45184	-406903.5315	0.00
M0003	-648.45045	-406902.6599	0.87

M0002	-648.44947	-406902.0424	1.49
M0004	-648.44657	-406900.2208	3.31
M0005	-648.44589	-406899.7953	3.74
M0010	-648.44409	-406898.6677	4.86
M0007	-648.44393	-406898.5636	4.97
M0006	-648.44360	-406898.3596	5.17
M0008	-648.44295	-406897.9480	5.58
M0011	-648.44199	-406897.3481	6.18
M0009	-648.43749	-406894.5269	9.00

Table S18. Energies calculated at DFT/B3LYP/6-31G* in vacuum for the conformers found for $\mathbf{53_{RO}/53_{RC}/53_{RE}}$.

Isomer	ΔG° (au)	ΔG° (kcal/mol)	ΔG° (kcal/mol)
$\mathbf{53_{RO}}$	-648.264647	-406786.07	0.00
$\mathbf{53_{RC}}$	-648.249198	-406776.37	9.69
$\mathbf{53_{RE}}$	-648.264584	-406786.03	0.04

Table S19. Relative free energies (ΔG°) of the lowest energy geometries of $\mathbf{53_{RO}/53_{RC}/53_{RE}}$ at DFT/B3LYP/6-31G* in vacuum in kcal/mol.

XYZ Coordinates

53_{RO}

C	1.54459100	1.04355000	-0.06350000
O	3.70547100	-0.24832000	-0.50042000
C	1.68107100	-1.41754000	-0.02966000
C	3.14582100	-1.25391000	0.33534000
N	0.90435100	-0.18760000	0.13310000
C	3.07473100	0.99485000	-0.25488000
H	1.58877100	-1.70465000	-1.08474000
H	3.27836100	-0.98082000	1.38901000
H	3.52454100	1.43160000	0.64487000
H	1.24606100	-2.21771000	0.57960000
H	3.68839100	-2.18683000	0.15439000
H	3.31844100	1.64990000	-1.09807000
O	0.98662100	2.13981000	-0.01517000
C	-0.48675900	-0.37371000	0.19931000
O	-0.98292900	-1.50306000	0.11731000
C	-1.37030900	0.85532000	0.35410000
H	-1.28857900	1.47429000	-0.54597000
H	-1.01307900	1.42384000	1.22021000
C	-2.84206900	0.51245000	0.60205000
H	-2.92443900	-0.20965000	1.42375000
H	-3.35712900	1.43020000	0.91062000
N	-3.48616900	-0.01445000	-0.60699000
H	-3.00626900	-0.88318000	-0.86430000
C	-4.89025900	-0.31556000	-0.35949000
H	-5.44508900	0.58637000	-0.08169000
H	-5.00781900	-1.06610000	0.42897000
H	-5.34365900	-0.71305000	-1.27276000

53_{RC}

C	0.04660000	-0.63167000	0.12019200
O	2.51874000	-0.96552800	0.04683200
C	1.62259800	1.29025100	0.09506200
C	2.70832900	0.36598300	-0.44609800
N	0.27283800	0.78298000	-0.15905800
C	1.25305100	-1.47817900	-0.42554800
H	1.72516800	1.39301200	1.18237200
H	2.70756900	0.34980300	-1.54242800
H	1.26674100	-1.49990900	-1.52231800
H	1.73890700	2.28755200	-0.34345800
H	3.69370800	0.71033400	-0.11726800
H	1.20288200	-2.50754900	-0.05701800
O	0.01788000	-0.76304000	1.53389200
H	0.94877000	-0.70286900	1.80917200
N	-1.22846000	-1.04667100	-0.46940800
C	-1.53386800	-2.45911200	-0.22601800
H	-0.81553800	-3.11593100	-0.72604800
H	-2.50933800	-2.71896300	-0.65449800
H	-1.55823800	-2.70984200	0.84038200
C	-2.32185100	-0.22613300	0.05858200
H	-3.28401000	-0.57137400	-0.34094800
C	-0.75410300	1.70569900	-0.06954800
O	-0.55845400	2.90737900	0.12370200
H	-2.39473100	-0.29353300	1.15197200
C	-2.13672200	1.20648800	-0.37409800
H	-2.29820200	1.31194700	-1.45348800
H	-2.86385300	1.84270700	0.14231200

53$_{RE}$

C	1.54098800	0.99070200	-0.10008000
O	-0.77445300	1.61692800	0.79173000
C	-1.44655300	1.56441600	-0.47821000
N	1.63535000	-0.34833800	0.27868000
C	0.60016600	1.93851000	0.67990000
H	0.68528400	2.93860000	0.23651000
H	-0.76590200	1.23952800	-1.27244000
H	1.00415600	2.01919100	1.69557000
O	2.22057700	1.45780300	-1.02015000
H	-1.82301500	2.56081600	-0.72997000
C	-2.60092100	0.56006400	-0.34446000
H	-3.40106200	0.98208300	0.27196000
H	-3.00557100	0.29326400	-1.32561000
N	-2.13453900	-0.63225500	0.35083000
C	0.78168200	-1.02038900	1.26769000
H	1.45752300	-1.53748800	1.96008000
H	0.24666000	-0.28562000	1.87159000
C	-0.19470600	-2.04877100	0.68127000
H	-0.75461600	-2.53295200	1.48993000
H	0.33730500	-2.84318000	0.14657000
C	-1.17407800	-1.40575300	-0.27051000
O	-1.07461700	-1.51114300	-1.48851000
H	-2.01673900	-0.48778500	1.34530000
C	2.53727200	-1.19937600	-0.48742000
H	3.33139100	-0.62033500	-0.96679000
H	1.96372300	-1.71483700	-1.26290000
H	3.00003300	-1.93225500	0.18001000

Gaussian Calculations

53$_{RO}$

M06-2X/6-31G* with Solvent Correction

SCF Done: E(RM062X) = -648.188630653

Zero-point correction= 0.232304
Thermal correction to Gibbs Free Energy= 0.191825

C	1.56033200	1.02820600	-0.02634700
O	3.57142900	-0.28132300	-0.66713500
C	1.62664300	-1.46360400	0.05793800
C	3.11149500	-1.25150700	0.25229200
N	0.89703900	-0.18146400	0.13802500
C	3.05813100	0.97555700	-0.31803300
H	1.43208600	-1.92053100	-0.91671000
H	3.33270400	-0.93558900	1.28263800
H	3.55938800	1.37595700	0.57693700
H	1.22616500	-2.12454000	0.82755200
H	3.64681000	-2.18000800	0.04734900
H	3.24216300	1.66246600	-1.14632500
O	1.03165000	2.11749900	0.07600400
C	-0.49935900	-0.31169700	0.28940100
O	-0.96154300	-1.43661900	0.34360000
C	-1.37639300	0.91732600	0.38244800
H	-1.24082500	1.52176200	-0.51909900
H	-1.02446800	1.53677900	1.21188400
C	-2.84300100	0.54481700	0.55699500
H	-2.96357600	-0.08516700	1.45497700
H	-3.40542300	1.46844500	0.73651700
N	-3.38512600	-0.08080400	-0.64268600
H	-2.89936400	-0.96732900	-0.76289800
C	-4.81003100	-0.35176700	-0.50287300
H	-5.35508200	0.59621200	-0.44189200
H	-5.06348300	-0.94180500	0.39379800
H	-5.16967600	-0.89121500	-1.38277500

53_{RC}

M06-2X/6-31G* with Solvent Correction

SCF Done: E(RM062X) = -648.193438808

Zero-point correction= 0.234316
Thermal correction to Gibbs Free Energy= 0.196938

C	0.00308300	-0.66567500	0.11432500
O	2.35175000	-1.12294500	-0.18876600
C	1.67958300	1.11836300	0.41065100
C	2.66783100	0.25440800	-0.35184700
N	0.31167500	0.77398800	0.02420900
C	1.07008200	-1.40101800	-0.71474700
H	1.80384100	0.96949200	1.49077400
H	2.64728200	0.50758800	-1.42111300
H	0.98801500	-1.08684900	-1.76329900
H	1.83048300	2.17393400	0.18843600
H	3.68025800	0.39464500	0.03136400
H	0.93763800	-2.48036000	-0.63665600
O	0.12018800	-1.07571700	1.46437100
H	1.05545200	-1.29403500	1.61741400
N	-1.31722800	-0.93447400	-0.39160600
C	-1.72975700	-2.32616700	-0.20956500
H	-1.04352300	-3.00719000	-0.71473400
H	-2.71126400	-2.44768800	-0.67318900
H	-1.79644200	-2.61146800	0.84854900
C	-2.30872000	-0.02114200	0.17742800
H	-3.29852000	-0.35333400	-0.14522400
C	-0.59915400	1.78806800	-0.09792600
O	-0.27234800	2.96693000	-0.03884500
H	-2.29385400	-0.04055300	1.27872100
C	-2.03014200	1.37397400	-0.34687500
H	-2.20149100	1.40271600	-1.42866600
H	-2.67256000	2.12550400	0.11665900

53RE

M06-2X/6-31G* with Solvent Correction

SCF Done: E(RM062X) = -648.203976070

Zero-point correction= 0.234205
Thermal correction to Gibbs Free Energy= 0.196167

C	1.49854900	0.98154900	-0.05209500
O	-0.75928400	1.54611500	0.79320200
C	-1.37053600	1.52964500	-0.48906800
N	1.53369900	-0.34420400	0.24904900
C	0.59921200	1.91736800	0.77416700
H	0.73764700	2.91181900	0.33488900
H	-0.66135900	1.16605500	-1.24279300
H	0.93039700	1.94408400	1.81695900
O	2.17375200	1.46170000	-0.95784400
H	-1.69720100	2.53766400	-0.77492600
C	-2.55461500	0.56563300	-0.40510200
H	-3.37178100	0.99907000	0.17470700
H	-2.92276500	0.34012100	-1.40768700
N	-2.14440400	-0.66564400	0.26112300
C	0.77814300	-1.00769200	1.30235300
H	1.48167900	-1.51260200	1.97453200
H	0.23374100	-0.26201600	1.88111300
C	-0.20719100	-2.05706600	0.74021100
H	-0.78025400	-2.48162100	1.57027900
H	0.33239400	-2.86463200	0.24127900
C	-1.10221400	-1.37336800	-0.27205000
O	-0.85185900	-1.36946200	-1.46946700
H	-2.13091900	-0.59161300	1.27111300
C	2.35056900	-1.21190000	-0.59001700
H	3.16526100	-0.62915400	-1.01548500
H	1.74820800	-1.64063500	-1.39756200
H	2.75752000	-2.01945000	0.02484500

Isomer $54_{RO}/54_{RC}/54_{RE}$

Spartan Calculations

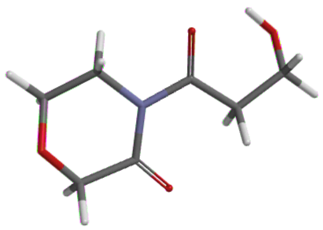

54_{RO}
ΔG ° = 2.9 kcal/mol

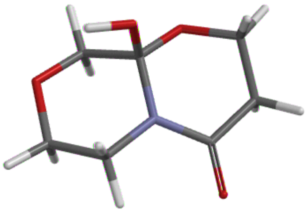

54_{RC}
ΔG ° = 9.2 kcal/mol

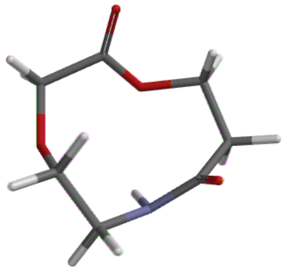

54_{RE}
ΔG ° = 0.0 kcal/mol

54_{RO}	Calculated energy (au)	Calculated energy (kcal/mol)	Relative energy (kcal/mol)
M0002	-629.0034	-394699.6316	0.00
M0001	-629.0031	-394699.4760	0.16
M0009	-628.9992	-394697.0256	2.61
M0010	-628.9988	-394696.7727	2.86
M0007	-628.9982	-394696.3548	3.28
M0003	-628.9980	-394696.2632	3.37
M0006	-628.9976	-394696.0241	3.61
M0011	-628.9975	-394695.9513	3.68
M0013	-628.9967	-394695.4512	4.18
M0004	-628.9963	-394695.1695	4.46
M0018	-628.9961	-394695.0509	4.58
M0012	-628.9959	-394694.9323	4.70
M0020	-628.9958	-394694.8620	4.77
M0021	-628.9948	-394694.2483	5.38
M0023	-628.9948	-394694.2263	5.41
M0015	-628.9945	-394694.0720	5.56
M0005	-628.9941	-394693.7952	5.84
M0024	-628.9939	-394693.6810	5.95
M0008	-628.9939	-394693.6804	5.95
M0022	-628.9935	-394693.4238	6.21
M0017	-628.9935	-394693.4206	6.21
M0014	-628.9935	-394693.4081	6.22
M0025	-628.9929	-394693.0303	6.60
M0016	-628.9924	-394692.7492	6.88
M0019	-628.9921	-394692.5296	7.10
**54_{RC}**	**Calculated energy (au)**	**Calculated energy (kcal/mol)**	**Relative energy (kcal/mol)**
M0001	-628.9980	-394696.2230	0.00
M0002	-628.9939	-394693.6710	2.55
M0003	-628.9907	-394691.6423	4.58
M0004	-628.9903	-394691.3994	4.82

M0005	-628.9902	-394691.3467	4.88
M0006	-628.9874	-394689.6224	6.60
M0007	-628.9869	-394689.3099	6.91
M0008	-628.9812	-394685.6892	10.53
**54_{RC}**	**Calculated energy (au)**	**Calculated energy (kcal/mol)**	**Relative energy (kcal/mol)**
M0001	-629.0138	-394706.1651	0.00
M0002	-629.0118	-394704.8932	1.27
M0006	-629.0089	-394703.1142	3.05
M0005	-629.0075	-394702.2006	3.96
M0003	-629.0068	-394701.7425	4.42
M0008	-629.0060	-394701.2851	4.88
M0007	-629.0041	-394700.0520	6.11
M0004	-629.0024	-394699.0324	7.13
M0010	-628.9975	-394695.9149	10.25
M0009	-628.9941	-394693.7990	12.37

Table S20. Energies calculated at DFT/B3LYP/6-31G* in vacuum for the conformers found for **54_{RO}/54_{RC}/54_{RE}**.

Isomer	ΔG° (au)	ΔG° (kcal/mol)	ΔG° (kcal/mol)
**54_{RO}**	-628.86046	-394609.94	2.9
**54_{RC}**	-628.850408	-394603.63	9.2
**54_{RE}**	-628.865018	-394612.80	0.0

Table S21. Relative free energies (ΔG°) of the lowest energy geometries of **54_{RO}/54_{RC}/54_{RE}** at DFT/B3LYP/6-31G* in vacuum in kcal/mol.

XYZ Coordinates

54_{RO}

C	-1.07204000	1.06323900	-0.03470000
O	-3.29841900	-0.17876300	-0.09695000
C	-1.24430800	-1.38771100	-0.21133000
C	-2.59399800	-1.27407200	0.47576000
N	-0.42778900	-0.18058000	-0.07589000
C	-2.60297000	1.02985800	0.14837000
H	-0.70637700	-2.25278100	0.19209000
H	-3.18251700	-2.18127300	0.30816000
H	-3.02836100	1.78458700	-0.52161000
H	-1.38536800	-1.54589100	-1.28792000
H	-2.49231800	-1.12807200	1.55773000
H	-2.81249100	1.34410800	1.17784000
O	-0.49651100	2.15076000	-0.06757000
C	0.94763100	-0.36601900	-0.27610000
O	1.41049200	-1.46940900	-0.58743000
C	1.86956000	0.82718200	-0.09054000
H	1.68498900	1.25458200	0.90083000
H	1.65871900	1.56265200	-0.87242000
C	3.33606000	0.43222300	-0.20356000
H	3.55894100	-0.06008700	-1.15630000
H	3.96483900	1.32522400	-0.13215000
O	3.70891100	-0.43822600	0.85979000
H	3.29909200	-1.30207700	0.65482000

54_{RC}

C	0.20634000	-0.80612000	0.08571000
O	2.63434000	-0.34567500	-0.05398000
C	1.03079400	1.49499800	0.22755000
C	2.36173300	1.02963600	-0.37379000
N	-0.06866800	0.59667000	-0.12089000
C	1.57266900	-1.18021300	-0.55703000
H	1.09843400	1.51179800	1.32213000
H	2.35723300	1.14197600	-1.46458000
H	1.53392900	-1.10313300	-1.65094000
H	0.80626600	2.51340900	-0.10730000
H	3.18370400	1.63493400	0.02077000
H	1.83130700	-2.21720300	-0.31522000
O	0.24819900	-1.02509000	1.48802000
H	1.11628900	-1.41611200	1.68031000
O	-0.78994200	-1.61756900	-0.51805000
C	-2.08351100	-1.29787600	-0.02143000
H	-2.79368200	-2.00707500	-0.45903000
C	-1.36034700	1.07403200	-0.04127000
O	-1.62837500	2.25115300	0.19897000
H	-2.12566100	-1.43140600	1.06577000
C	-2.44438900	0.10541400	-0.43251000
H	-2.55199900	0.16947500	-1.52139000
H	-3.38865800	0.40486600	0.03267000

54$_{RE}$

C	1.67519200	-0.78832300	0.11824900
O	1.27514300	1.57421500	-0.58927100
C	0.41058200	1.96989200	0.49120900
O	0.66783300	-1.10709700	-0.74476100
C	2.24016700	0.59706900	-0.20247100
H	2.94670700	0.47753200	-1.02993100
H	0.76458800	2.93216300	0.87430900
H	2.79748600	0.96543100	0.66625900
O	2.06018500	-1.49033200	1.04359900
H	0.43335500	1.24355200	1.31182900
C	-1.01855900	2.10857700	-0.05160100
H	-1.70953000	2.39205400	0.74820900
H	-1.05137200	2.86318600	-0.84378100
N	-1.45054400	0.85831500	-0.65820100
C	-0.28220300	-2.07668100	-0.26621100
H	-0.11672900	-3.01163000	-0.81056100
H	-0.16728200	-2.26998000	0.80669900
C	-1.68634500	-1.54796600	-0.54456100
H	-2.45119200	-2.23338900	-0.16640100
H	-1.84680500	-1.42942700	-1.62234100
C	-1.84195000	-0.20365700	0.13357900
O	-2.18840000	-0.09066800	1.30400900
H	-0.94481300	0.61988700	-1.50469100

Gaussian Calculations

54$_{RO}$

M06-2X/6-31G* with Solvent Correction

SCF Done: E(RM062X) = -628.764869784

Zero-point correction= 0.191391
Thermal correction to Gibbs Free Energy= 0.153685

C	-1.09183900	1.04276000	0.06284300
O	-3.21793300	-0.16936600	-0.34733900
C	-1.21588500	-1.44758200	-0.09610300
C	-2.63733800	-1.23522500	0.37623200
N	-0.44688500	-0.18730800	-0.03030900
C	-2.61788200	1.03816300	0.03457100
H	-0.70490400	-2.18400800	0.52553500
H	-3.22972100	-2.12985000	0.17763100
H	-2.92265000	1.81112000	-0.67368400
H	-1.20817800	-1.81096600	-1.12772700
H	-2.66672600	-1.02429300	1.45569200
H	-2.94564400	1.34668200	1.03953400
O	-0.51724100	2.10415300	0.19256600
C	0.94521200	-0.34296100	-0.14890000
O	1.38305600	-1.47497600	-0.27440300
C	1.86384400	0.85777000	-0.12766500
H	1.76773600	1.35939600	0.84002200
H	1.53421100	1.58481900	-0.87496500
C	3.30739800	0.42938300	-0.37339300
H	3.39080700	-0.03863800	-1.36350900
H	3.94266700	1.31874400	-0.36988700
O	3.79763500	-0.42613700	0.63792700
H	3.28541000	-1.24508700	0.54800700

54_{RC}

M06-2X/6-31G* with Solvent Correction

SCF Done: E(RM062X) = -628.769925577

Zero-point correction= 0.193291
Thermal correction to Gibbs Free Energy= 0.157964

C	0.22926500	-0.84630100	0.07077000
O	2.54496300	-0.31080800	-0.18287600
C	1.00440600	1.46269200	0.43077400
C	2.26981800	1.08035100	-0.32281500
N	-0.09184400	0.59014300	0.01864300
C	1.50020400	-1.07867100	-0.74321200
H	1.16434800	1.36949200	1.51268700
H	2.16073200	1.32178400	-1.38924400
H	1.31608000	-0.80802300	-1.79069700
H	0.71400700	2.49052200	0.21280300
H	3.13353400	1.61267300	0.07941600
H	1.78233400	-2.13108300	-0.67107300
O	0.44358500	-1.22828600	1.40402700
H	1.38335900	-1.07243300	1.59865500
O	-0.77359800	-1.61122600	-0.47897100
C	-2.05788400	-1.31200400	0.06889300
H	-2.73814100	-2.07001900	-0.31980000
C	-1.36119000	1.09523500	-0.06323200
O	-1.59929900	2.28757300	0.06021500
H	-2.02036400	-1.39447500	1.16018700
C	-2.45047800	0.08611700	-0.37386700
H	-2.61538400	0.10037400	-1.45693000
H	-3.36764200	0.42763600	0.11046200

54_{RE}

M06-2X/6-31G* with Solvent Correction

SCF Done: E(RM062X) = -628.775315091

Zero-point correction= 0.192754
Thermal correction to Gibbs Free Energy= 0.156180

C	1.65475800	-0.75440500	0.11008900
O	1.23163900	1.53489300	-0.59726100
C	0.40045700	1.89750300	0.49929200
O	0.62536200	-1.06488600	-0.68965000
C	2.23304500	0.61162500	-0.25986100
H	2.87644200	0.49799000	-1.13738100
H	0.78290100	2.80900900	0.97565700
H	2.84587400	0.94459800	0.58618900
O	2.05872800	-1.44935600	1.00819600
H	0.38169600	1.09850400	1.25399700
C	-1.01462100	2.09896200	-0.02819200
H	-1.68399000	2.35889900	0.79349000
H	-1.03640700	2.90288400	-0.76764300
N	-1.49721500	0.87817200	-0.65729400
C	-0.29002200	-2.08707800	-0.26017700
H	-0.07984500	-3.00587900	-0.81139600
H	-0.15097300	-2.26357600	0.80837900
C	-1.69078300	-1.54873500	-0.55334700
H	-2.43662100	-2.22633800	-0.13282000
H	-1.84733600	-1.46877800	-1.63275100
C	-1.79909100	-0.19463300	0.13150700
O	-2.05373200	-0.09376600	1.32327200
H	-1.02967000	0.65096700	-1.52699400

Isomer 56_{RO}/56_{RC}/56_{RE}

Spartan Calculations

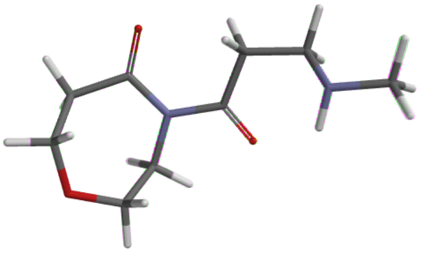

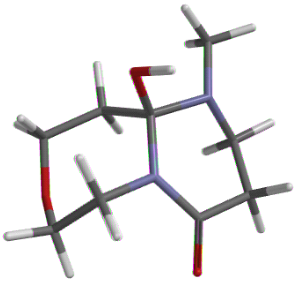

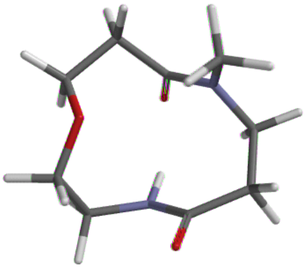

56_{RO} $\Delta G° = 5.5$ kcal/mol

56_{RC} $\Delta G° = 17.9$ kcal/mol

56_{RE} $\Delta G° = 0.0$ kcal/mol

56_{RO}	Calculated energy (au)	Calculated energy (kcal/mol)	Relative energy (kcal/mol)
M0016	-687.75522	-431566.3993	0.00
M0017	-687.75522	-431566.3993	0.00
M0018	-687.75522	-431566.3993	0.00
M0020	-687.75475	-431566.1044	0.29
M0021	-687.75475	-431566.1044	0.29
M0022	-687.75475	-431566.1044	0.29
M0014	-687.75418	-431565.7505	0.65
M0015	-687.75418	-431565.7505	0.65
M0035	-687.75295	-431564.9742	1.43
M0036	-687.75295	-431564.9742	1.43
M0037	-687.75295	-431564.9742	1.43
M0065	-687.75284	-431564.9046	1.49
M0001	-687.75250	-431564.6938	1.71
M0002	-687.75250	-431564.6938	1.71
M0063	-687.75209	-431564.4365	1.96
M0064	-687.75209	-431564.4365	1.96
M0003	-687.75187	-431564.3003	2.10
M0004	-687.75187	-431564.3003	2.10
M0060	-687.75169	-431564.1836	2.22
M0061	-687.75169	-431564.1836	2.22
M0043	-687.75117	-431563.8617	2.54
M0044	-687.75117	-431563.8617	2.54
M0045	-687.75117	-431563.8617	2.54
M0005	-687.75096	-431563.7249	2.67
M0006	-687.75096	-431563.7249	2.67
M0071	-687.75093	-431563.7098	2.69
M0072	-687.75093	-431563.7098	2.69
M0055	-687.75088	-431563.6791	2.72
M0056	-687.75088	-431563.6791	2.72

M0057	-687.75088	-431563.6791	2.72
M0009	-687.75083	-431563.6471	2.75
M0010	-687.75083	-431563.6471	2.75
M0088	-687.75073	-431563.5812	2.82
M0089	-687.75073	-431563.5812	2.82
M0011	-687.75058	-431563.4883	2.91
M0012	-687.75058	-431563.4883	2.91
M0013	-687.75058	-431563.4883	2.91
M0007	-687.75040	-431563.3754	3.02
M0008	-687.75040	-431563.3754	3.02
M0093	-687.75034	-431563.3409	3.06
M0092	-687.75034	-431563.3409	3.06
M0038	-687.75022	-431563.2637	3.14
M0039	-687.75022	-431563.2637	3.14
M0040	-687.75022	-431563.2637	3.14
M0023	-687.74954	-431562.8364	3.56
M0024	-687.74954	-431562.8364	3.56
M0025	-687.74954	-431562.8364	3.56
M0046	-687.74952	-431562.8219	3.58
M0047	-687.74952	-431562.8219	3.58
M0048	-687.74952	-431562.8219	3.58
M0054	-687.74932	-431562.6952	3.70
M0066	-687.74919	-431562.6174	3.78
M0067	-687.74919	-431562.6174	3.78
M0033	-687.74917	-431562.6023	3.80
M0034	-687.74917	-431562.6023	3.80
M0058	-687.74908	-431562.5496	3.85
M0097	-687.74907	-431562.5408	3.86
M0031	-687.74896	-431562.4718	3.93
M0032	-687.74896	-431562.4718	3.93
M0080	-687.74872	-431562.3199	4.08
M0081	-687.74872	-431562.3199	4.08
M0049	-687.74866	-431562.2854	4.11
M0050	-687.74866	-431562.2854	4.11
M0051	-687.74866	-431562.2854	4.11
M0076	-687.74854	-431562.2114	4.19
M0077	-687.74854	-431562.2114	4.19
M0027	-687.74848	-431562.1706	4.23
M0028	-687.74848	-431562.1706	4.23
M0041	-687.74831	-431562.0645	4.33
M0042	-687.74831	-431562.0645	4.33
M0059	-687.74829	-431562.0488	4.35
M0062	-687.74827	-431562.0407	4.36
M0030	-687.74810	-431561.9302	4.47
M0026	-687.74801	-431561.8763	4.52
M0019	-687.74799	-431561.8650	4.53

M0029	-687.74786	-431561.7796	4.62
M0073	-687.74729	-431561.4251	4.97
M0074	-687.74729	-431561.4251	4.97
M0075	-687.74717	-431561.3492	5.05
M0052	-687.74714	-431561.3285	5.07
M0053	-687.74714	-431561.3285	5.07
M0082	-687.74668	-431561.0442	5.36
M0083	-687.74668	-431561.0442	5.36
M0068	-687.74603	-431560.6307	5.77
M0069	-687.74603	-431560.6307	5.77
M0070	-687.74603	-431560.6307	5.77
M0084	-687.74543	-431560.2554	6.14
M0085	-687.74543	-431560.2554	6.14
M0078	-687.74537	-431560.2222	6.18
M0079	-687.74537	-431560.2222	6.18
M0086	-687.74496	-431559.9605	6.44
M0087	-687.74267	-431558.5248	7.87
M0096	-687.74187	-431558.0215	8.38
M0100	-687.74064	-431557.2491	9.15
M0090	-687.73726	-431555.1300	11.27
M0091	-687.73726	-431555.1300	11.27
M0099	-687.73587	-431554.2603	12.14
M0098	-687.73587	-431554.2603	12.14
M0094	-687.73489	-431553.6435	12.76
M0095	-687.73489	-431553.6435	12.76
**56_{RC}**	**Calculated energy (au)**	**Calculated energy (kcal/mol)**	**Relative energy (kcal/mol)**
M0001	-687.74190	-431558.0441	0.00
M0004	-687.73880	-431556.0945	1.95
M0008	-687.73716	-431555.0698	2.97
M0009	-687.73713	-431555.0478	3.00
M0007	-687.73656	-431554.6908	3.35
M0003	-687.73594	-431554.2992	3.74
M0002	-687.73593	-431554.2948	3.75
M0015	-687.73335	-431552.6771	5.37
M0012	-687.73322	-431552.5924	5.45
M0005	-687.73280	-431552.3295	5.71
M0014	-687.73239	-431552.0716	5.97
M0006	-687.73183	-431551.7240	6.32
M0010	-687.72906	-431549.9826	8.06
M0011	-687.72894	-431549.9067	8.14
M0016	-687.72794	-431549.2836	8.76
M0013	-687.72391	-431546.7542	11.29
**56_{RE}**	**Calculated energy (au)**	**Calculated energy (kcal/mol)**	**Relative energy (kcal/mol)**
M0005	-687.77425	-431578.3412	0.00
M0003	-687.77344	-431577.8349	0.51

M0011	-687.77248	-431577.2337	1.11
M0001	-687.77241	-431577.1873	1.15
M0009	-687.77231	-431577.1252	1.22
M0012	-687.77189	-431576.8629	1.48
M0008	-687.77155	-431576.6489	1.69
M0002	-687.77145	-431576.5836	1.76
M0006	-687.77031	-431575.8714	2.47
M0016	-687.76995	-431575.6443	2.70
M0004	-687.76987	-431575.5928	2.75
M0010	-687.76986	-431575.5846	2.76
M0019	-687.76872	-431574.8743	3.47
M0023	-687.76630	-431573.3507	4.99
M0007	-687.76513	-431572.6166	5.72
M0014	-687.76492	-431572.4842	5.86
M0017	-687.76347	-431571.5743	6.77
M0022	-687.76319	-431571.3998	6.94
M0013	-687.76201	-431570.6594	7.68
M0015	-687.76175	-431570.4981	7.84
M0018	-687.76133	-431570.2339	8.11
M0020	-687.76100	-431570.0262	8.32
M0021	-687.76073	-431569.8549	8.49

Table S22. Energies calculated at DFT/B3LYP/6-31G* in vacuum for the conformers found for $\mathbf{56_{RO}/56_{RC}/56_{RE}}$.

Isomer	ΔG° (au)	ΔG° (kcal/mol)	ΔG° (kcal/mol)
$\mathbf{56_{RO}}$	-687.549503	-431437.31	5.5
$\mathbf{56_{RC}}$	-687.529745	-431424.91	17.9
$\mathbf{56_{RE}}$	-687.558325	-431442.85	0.00

Table S23. Relative free energies (ΔG°) of the lowest energy geometries of $\mathbf{56_{RO}/56_{RC}/56_{RE}}$ at DFT/B3LYP/6-31G* in vacuum in kcal/mol.

XYZ Coordinates

56$_{RO}$

H	-2.98254900	2.06424100	0.90578000
C	-2.74667000	1.13366000	0.37483000
H	-3.45971000	1.03042100	-0.44881000
C	-2.82504000	-0.01529000	1.36638000
H	-3.65395000	0.17481100	2.05660000
H	-1.91614000	-0.12206000	1.97038000
O	-3.13421000	-1.24529900	0.71295000
C	-2.03162100	-1.82097000	0.00818000
H	-2.38548100	-2.78432000	-0.37359000
H	-1.21036100	-2.02378000	0.70506000
C	-1.59548000	-0.95368000	-1.17189000
H	-1.04617000	-1.56992000	-1.89225000
H	-2.47004000	-0.55275000	-1.69655000
N	-0.75280000	0.16925000	-0.74858000
C	-1.34435000	1.29466000	-0.17863000
O	-0.82206900	2.40979000	-0.17193000
C	0.62818000	-0.08283100	-0.71800000
O	1.08190000	-1.19232100	-1.01898000
C	1.54800000	1.03562900	-0.24898000
H	1.43442100	1.87664900	-0.94216000
H	1.25216000	1.35367900	0.75694000
C	3.02657000	0.64051900	-0.24099000
H	3.61843000	1.54819900	-0.07157000
H	3.31301000	0.23247900	-1.21808000
N	3.31711000	-0.33061100	0.82176000
H	2.80293000	-1.19071100	0.60638000
C	4.74036000	-0.64474200	0.85980000
H	4.92971000	-1.37875200	1.64912000
H	5.33569000	0.24525800	1.08771000
H	5.08322000	-1.07301200	-0.08776000

56$_{RC}$

H	2.97160000	1.07374000	-0.43991000
C	2.69630000	0.11514000	0.01249000
H	3.61267000	-0.28815000	0.45863000
O	2.31681000	-0.73959000	-1.06524000
C	1.49141100	-1.84128000	-0.71472000
C	0.02407000	-1.44547100	-0.85153000
H	-0.54360900	-2.38030100	-0.93101000
H	-0.09574000	-0.89291100	-1.78998000
C	-0.51120000	-0.62064100	0.33773000
C	1.65501000	0.34003000	1.11828000
O	-0.43190000	-1.42822100	1.49584000
H	-0.95239000	-0.90026100	2.12467000
N	0.28953000	0.55588000	0.63310000
N	-1.91214000	-0.21544100	0.24200000
H	1.70099100	-2.62274000	-1.45388000
H	1.75231100	-2.25929000	0.26358000
H	1.66695000	-0.49785000	1.82275000
H	1.95818000	1.21671000	1.70495000
C	0.04499000	1.77998900	0.03262000
O	0.89425000	2.67041000	-0.04279000
C	-1.36735000	2.06010900	-0.39525000
H	-1.34835000	2.75322900	-1.24344000
H	-1.86947000	2.56075900	0.44140000
C	-2.10841000	0.80659900	-0.78371000
C	-2.83422000	-1.33027100	0.00860000
H	-3.86989000	-0.98400100	0.10595000
H	-2.71010900	-2.12246100	0.75454000
H	-2.72522900	-1.77011100	-0.98875000
H	-3.17250000	1.06207900	-0.86881000
H	-1.79404000	0.46246900	-1.77591000

56_{RE}

H	-3.20244400	-1.33363200	-0.41616900
C	-2.40509500	-0.58724900	-0.50882900
H	-2.70101400	0.09037900	-1.31683900
O	-1.19745900	-1.21726800	-0.94538900
C	-0.75002400	-2.29680900	-0.13081900
C	0.77409700	-2.32547600	-0.18881900
H	1.11273900	-2.37180200	-1.22327900
H	1.16299400	-3.21027000	0.32648100
C	-2.26766900	0.22412700	0.79790100
N	-1.00449900	0.92749000	0.94224100
N	1.80904100	-0.03117300	-0.16879900
H	-1.10117800	-2.20378400	0.90210100
H	-1.15859500	-3.22445700	-0.54474900
H	-3.08478000	0.95046300	0.86810100
H	-2.34543100	-0.44326600	1.66242100
C	-0.59625700	1.84766700	0.01040100
O	-1.36843800	2.33989500	-0.81206900
C	0.85269400	2.29771700	0.07654100
H	0.90215800	3.16779000	0.74123100
H	1.11071900	2.65071800	-0.93038900
C	1.90390700	1.26985100	0.50268100
C	1.99752200	-0.04121500	-1.61265900
H	2.66912800	0.77125300	-1.90544900
H	1.02847700	0.08527500	-2.10235900
H	2.45283100	-0.98226600	-1.92933900
H	2.90408000	1.66889300	0.29352100
H	1.85611200	1.14149900	1.58954100
H	-0.30063900	0.46626900	1.51196100
C	1.27937600	-1.09931500	0.54687100
O	1.10736300	-1.02676200	1.76990100

Gaussian Calculations

56_{RO}

M06-2X/6-31G* with Solvent Correction

SCF Done: E(RM062X) = -687.482604990

Zero-point correction= 0.261833
Thermal correction to Gibbs Free Energy= 0.220181

H	-3.08379500	2.11418700	0.59368300
C	-2.79756900	1.14696300	0.17622000
H	-3.40070100	0.98281900	-0.72350100
C	-3.10740000	0.03596600	1.19222700
H	-4.05116300	0.27413200	1.68705200
H	-2.32070200	-0.00574700	1.96127800
O	-3.29245800	-1.23386600	0.60953500
C	-2.10853600	-1.81196500	0.10659000
H	-2.39304700	-2.80479800	-0.25045100
H	-1.36114700	-1.92880500	0.90654900
C	-1.49263800	-1.02944600	-1.04837900
H	-0.82111800	-1.67926600	-1.60540900
H	-2.27869100	-0.69120200	-1.72793300
N	-0.68514000	0.12645300	-0.62258200
C	-1.32787900	1.28873600	-0.17512000
O	-0.75172300	2.34815100	-0.03598400
C	0.71383600	-0.03245300	-0.67644900
O	1.17390800	-1.08708900	-1.07853200
C	1.62234700	1.08555600	-0.20627700
H	1.47141600	1.95308600	-0.85376200
H	1.31840600	1.40186000	0.79561100
C	3.08267800	0.65359400	-0.20539700
H	3.69058100	1.52667400	0.05901200
H	3.38223400	0.34827200	-1.22225300
N	3.33620600	-0.38361800	0.78723800
H	2.82433200	-1.21200100	0.48961300
C	4.75278500	-0.71888500	0.85510200
H	4.90056800	-1.55357200	1.54510700
H	5.31085700	0.14082300	1.24094300
H	5.19093500	-0.99228600	-0.11938200

56_{RC}

M06-2X/6-31G* with Solvent Correction

SCF Done: E(RM062X) = -687.485321468

Zero-point correction= 0.263917
Thermal correction to Gibbs Free Energy= 0.226306

H	2.97230900	1.01910500	-0.55408000
C	2.70837800	0.07356200	-0.07488400
H	3.61521600	-0.35720700	0.37601400
O	2.22290900	-0.75660600	-1.11112200
C	1.48122400	-1.88429700	-0.68436900
C	-0.01968200	-1.59624800	-0.68173600
H	-0.55957400	-2.54123100	-0.56267400
H	-0.27131100	-1.18710900	-1.66629000
C	-0.53637200	-0.62767200	0.39417900
C	1.68333100	0.32001300	1.03185800
O	-0.49603700	-1.33547800	1.60390900
H	-0.92649900	-0.76305200	2.26288900
N	0.34187800	0.55941500	0.50844900
N	-1.90654700	-0.17844800	0.18038800
H	1.67554100	-2.67731400	-1.41259000
H	1.82511400	-2.24694500	0.29327500
H	1.62016700	-0.53039300	1.70978800
H	1.98766200	1.19517500	1.60933800
C	0.07075600	1.81950700	0.05790200
O	0.90909500	2.71291600	0.11334900
C	-1.32526900	2.08456700	-0.45583400
H	-1.24558200	2.80269500	-1.27464000
H	-1.87636900	2.56674100	0.35929700
C	-2.03897300	0.81509300	-0.87719600
C	-2.84151700	-1.28470000	-0.01010300
H	-3.85744800	-0.90207500	0.11791100
H	-2.65992800	-2.05180100	0.74378800
H	-2.77019300	-1.74111700	-1.00957300
H	-3.10525100	1.01149100	-1.01881800
H	-1.65017500	0.45067000	-1.84348400

56$_{RE}$

M06-2X/6-31G* with Solvent Correction

SCF Done: E(RM062X) = -687.505863057

Zero-point correction= 0.263749
Thermal correction to Gibbs Free Energy= 0.224879

H	0.69477900	-3.32524200	-0.41052100
C	0.09887700	-2.40526300	-0.49400200
H	-0.63989100	-2.53531800	-1.28470000
O	0.89813000	-1.31520400	-0.90371100
C	2.10767100	-1.14918800	-0.18685300
C	2.43931000	0.34233000	-0.20271900
H	2.57962300	0.67804500	-1.22996300
H	3.36027700	0.51850300	0.35835300
C	-0.62577500	-2.14719400	0.84144400
N	-1.10027900	-0.77933200	0.97100600
N	0.36190600	1.70304400	-0.17162200
H	1.99800400	-1.47369300	0.85531600
H	2.91010100	-1.73688900	-0.65149800
H	-1.46325400	-2.84846200	0.91625100
H	0.03356400	-2.33225100	1.69262700
C	-1.89416000	-0.23082400	0.02126100
O	-2.46390700	-0.90918500	-0.82833900
C	-2.08620000	1.28186000	0.06695200
H	-2.92270500	1.50817400	0.73834000
H	-2.40673200	1.56516500	-0.93903600
C	-0.86816700	2.10725800	0.50672000
C	0.36248000	1.80301300	-1.62824700
H	-0.38604700	2.54345400	-1.91658300
H	0.11835600	0.84286200	-2.09344600
H	1.32848800	2.15152700	-1.99570900
H	-1.06185900	3.16292200	0.28964400
H	-0.69005600	2.01926600	1.57787200
H	-0.53415700	-0.16486400	1.54752200
C	1.30392600	1.03121800	0.53374900
O	1.21857200	0.86958500	1.75705200

Isomer $57_{RO}/57_{RC}/57_{RE}$

Spartan Calculations

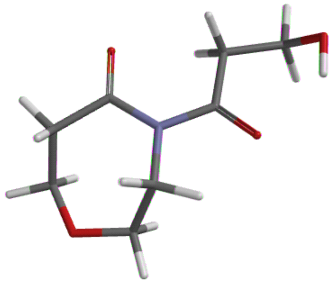

57_{RO}
ΔG ° = 10.4 kcal/mol

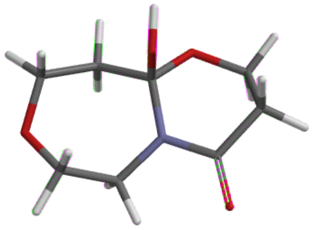

57_{RC}
ΔG ° = 19.6 kcal/mol

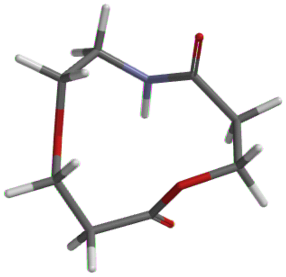

57_{RE}
ΔG ° = 0.0 kcal/mol

57_{RO}	Calculated energy (au)	Calculated energy (kcal/mol)	Relative energy (kcal/mol)
M0003	-668.31340	-419366.6604	0.00
M0001	-668.31260	-419366.1534	0.51
M0026	-668.31141	-419365.4066	1.25
M0025	-668.31099	-419365.1469	1.51
M0002	-668.31021	-419364.6587	2.00
M0005	-668.30899	-419363.8893	2.77
M0009	-668.30822	-419363.4099	3.25
M0011	-668.30760	-419363.0209	3.64
M0017	-668.30744	-419362.9199	3.74
M0032	-668.30735	-419362.8640	3.80
M0016	-668.30702	-419362.6551	4.01
M0006	-668.30700	-419362.6438	4.02
M0008	-668.30691	-419362.5848	4.08
M0014	-668.30667	-419362.4335	4.23
M0042	-668.30646	-419362.3024	4.36
M0012	-668.30574	-419361.8537	4.81
M0010	-668.30539	-419361.6310	5.03
M0015	-668.30507	-419361.4289	5.23
M0019	-668.30471	-419361.2055	5.45
M0004	-668.30450	-419361.0738	5.59
M0023	-668.30449	-419361.0694	5.59
M0018	-668.30386	-419360.6740	5.99
M0020	-668.30382	-419360.6489	6.01
M0024	-668.30382	-419360.6489	6.01
M0007	-668.30381	-419360.6414	6.02
M0021	-668.30354	-419360.4701	6.19
M0027	-668.30351	-419360.4538	6.21
M0034	-668.30296	-419360.1087	6.55
M0022	-668.30292	-419360.0848	6.58
M0013	-668.30265	-419359.9141	6.75

M0033	-668.30263	-419359.9028	6.76
M0039	-668.30132	-419359.0758	7.58
M0037	-668.30003	-419358.2663	8.39
M0031	-668.29845	-419357.2767	9.38
M0038	-668.29831	-419357.1876	9.47
M0030	-668.29749	-419356.6743	9.99
M0036	-668.29735	-419356.5859	10.07
M0029	-668.29704	-419356.3907	10.27
M0028	-668.29652	-419356.0638	10.60
M0040	-668.29438	-419354.7235	11.94
M0043	-668.29394	-419354.4448	12.22
M0041	-668.29102	-419352.6138	14.05
M0035	-668.28807	-419350.7627	15.90
**57_{RC}**	**Calculated energy (au)**	**Calculated energy (kcal/mol)**	**Relative energy (kcal/mol)**
M0002	-668.30600	-419362.0144	0.00
M0007	-668.30372	-419360.5818	1.43
M0003	-668.30193	-419359.4611	2.55
M0001	-668.30020	-419358.3730	3.64
M0006	-668.30002	-419358.2619	3.75
M0004	-668.29936	-419357.8497	4.16
M0005	-668.29889	-419357.5560	4.46
M0008	-668.29605	-419355.7739	6.24
**57_{RE}**	**Calculated energy (au)**	**Calculated energy (kcal/mol)**	**Relative energy (kcal/mol)**
M0002	-668.33782	-419381.9802	0.00
M0005	-668.33549	-419380.5219	1.46
M0001	-668.33523	-419380.3581	1.62
M0006	-668.33431	-419379.7795	2.20
M0004	-668.33320	-419379.0855	2.89
M0007	-668.33318	-419379.0673	2.91
M0013	-668.33178	-419378.1913	3.79
M0016	-668.33157	-419378.0614	3.92
M0003	-668.33047	-419377.3718	4.61
M0008	-668.33038	-419377.3122	4.67
M0015	-668.32979	-419376.9426	5.04
M0014	-668.32966	-419376.8598	5.12
M0012	-668.32910	-419376.5115	5.47
M0011	-668.32873	-419376.2781	5.70
M0019	-668.32866	-419376.2348	5.75
M0017	-668.32804	-419375.8457	6.13
M0018	-668.32786	-419375.7315	6.25
M0010	-668.32710	-419375.2553	6.72
M0009	-668.32645	-419374.8486	7.13
M0020	-668.32548	-419374.2393	7.74
M0022	-668.32526	-419374.1019	7.88
M0021	-668.32395	-419373.2755	8.70

M0023	-668.32326	-419372.8469	9.13

Table S24. Energies calculated at DFT/B3LYP/6-31G* in vacuum for the conformers found for $\mathbf{57_{RO}/57_{RC}/57_{RE}}$.

Isomer	ΔG° (au)	ΔG° (kcal/mol)	ΔG° (kcal/mol)
$\mathbf{57_{RO}}$	-668.145533	-419261.32	10.4
$\mathbf{57_{RC}}$	-668.130837	-419252.10	19.6
$\mathbf{57_{RE}}$	-668.16214	-419271.74	0.00

Table S25. Relative free energies (ΔG°) of the lowest energy geometries of $\mathbf{57_{RO}/57_{RC}/57_{RE}}$ at DFT/B3LYP/6-31G* in vacuum in kcal/mol.

XYZ Coordinates

57$_{RO}$

H	2.74199000	1.02521800	-1.25742000
C	2.31001000	1.28835900	-0.28723000
H	2.57477100	2.33281800	-0.08070000
C	2.83686900	0.42549800	0.84698000
H	2.17895900	0.42529900	1.72418000
H	3.80604000	0.82257800	1.16739000
O	3.08447900	-0.91107200	0.41545000
C	1.90201800	-1.69287100	0.23257000
H	1.34458800	-1.75190100	1.17440000
H	2.24293800	-2.70404100	-0.01307000
C	1.04697900	-1.16815100	-0.91966000
H	1.68053900	-0.85542100	-1.75715000
H	0.40709800	-1.97562000	-1.29227000
N	0.21459900	-0.02866000	-0.51616000
C	0.79671000	1.22965900	-0.36117000
O	0.15887100	2.28213000	-0.33049000
C	-1.08351100	-0.34705900	-0.08903000
O	-1.46076200	-1.52094900	0.00509000
C	-2.02217000	0.78839100	0.28540000
H	-1.57416000	1.36838100	1.09795000
H	-2.16948000	1.41489100	-0.60065000
C	-3.37318100	0.27253200	0.76466000
H	-3.98813000	1.11126200	1.10561000
H	-3.27234100	-0.43111800	1.59798000
O	-4.07778100	-0.36985800	-0.29216000
H	-3.59580200	-1.20187800	-0.47141000

57_{RC}

H	2.58364000	2.34134100	0.14297500
C	2.02679200	1.40556200	0.26017300
H	1.74677800	1.31858100	1.31627100
O	2.93303100	0.36743100	-0.11629100
C	2.50192900	-0.93084000	0.27154500
C	1.29379800	-1.42139300	-0.52254100
H	1.27046900	-2.51343100	-0.39935400
H	1.43403000	-1.21498400	-1.59089100
C	-0.04665600	-0.84924700	-0.02994200
C	0.82105800	1.43891300	-0.67398200
O	-0.12306200	-1.06835800	1.36212900
H	-0.69729600	-1.85144000	1.41508400
N	-0.27167500	0.56098600	-0.26181300
O	-1.12232000	-1.61141300	-0.65281900
H	3.34263200	-1.59999300	0.05543200
H	2.35067800	-0.97099900	1.35586300
H	0.45642900	2.46967100	-0.73207000
H	1.10559100	1.14569400	-1.69109900
C	-1.49546100	1.13033200	0.08722500
O	-1.69150600	2.34389600	0.15276600
C	-2.61262800	0.14266300	0.37452700
H	-3.56760100	0.60408400	0.10372500
H	-2.62605700	-0.10141800	1.44043500
C	-2.42491100	-1.09095400	-0.45399200
H	-3.01538100	-1.88415200	0.01865800
H	-2.83484900	-0.87252500	-1.44669300

57$_{RE}$

H	-0.95041500	1.60039900	-1.30496100
C	-0.89587900	2.12187800	-0.34291100
H	-1.07816900	3.18776000	-0.51059100
O	0.39377900	1.96265500	0.26686000
C	1.44669700	1.75513500	-0.67435100
C	2.43757700	0.76367500	-0.07813000
H	2.80584100	1.15088100	0.87899000
H	3.29087600	0.61272700	-0.74630100
C	-1.94253500	1.53345800	0.61154000
N	-1.59072900	0.16680500	0.97274000
O	1.00245000	-0.97252100	-0.82870100
H	1.07740300	1.39275900	-1.64019100
H	1.93845600	2.71837000	-0.84564100
H	-1.96632000	2.10240900	1.54640000
H	-2.93627500	1.55044800	0.15332000
C	-1.71856900	-0.85160400	0.05073000
O	-2.40514800	-0.76937700	-0.96273100
C	-0.87475100	-2.06555200	0.36386000
H	-1.51631000	-2.95281600	0.34036000
H	-0.47253000	-1.99158600	1.37942000
C	0.25483800	-2.19235300	-0.65473100
H	-0.82374000	0.09442700	1.63175000
C	1.80005400	-0.57781900	0.19767000
O	2.00229700	-1.23447100	1.21091000
H	0.93656000	-3.00779000	-0.38627100
H	-0.16188500	-2.43682900	-1.63822100

Gaussian Calculations

57$_{\mathbf{RO}}$

M06-2X/6-31G* with Solvent Correction

SCF Done: E(RM062X) = -668.058926951

Zero-point correction= 0.220793
Thermal correction to Gibbs Free Energy= 0.181545

H	2.71082900	0.97659300	-1.32183500
C	2.30048400	1.28236700	-0.35309800
H	2.54749400	2.33572000	-0.20733400
C	2.95723200	0.43981600	0.75230900
H	2.36690500	0.50338000	1.67965700
H	3.94899600	0.84910500	0.95512600
O	3.16696900	-0.90625700	0.39406400
C	1.98458200	-1.66874500	0.30692600
H	1.44867200	-1.67023200	1.26879600
H	2.30688200	-2.68968200	0.08814600
C	1.03975600	-1.20240000	-0.79565800
H	1.61091300	-0.95758800	-1.69452300
H	0.34866700	-2.00585000	-1.04174300
N	0.20782800	-0.04642400	-0.41856000
C	0.78396500	1.23302500	-0.36735800
O	0.12275900	2.24758200	-0.30185500
C	-1.13779300	-0.31216100	-0.11813700
O	-1.54348100	-1.46285800	-0.18515600
C	-2.06369000	0.80508000	0.31540300
H	-1.58963100	1.39540800	1.10441700
H	-2.20894700	1.48923600	-0.52548600
C	-3.40239800	0.24015100	0.78193000
H	-4.03233100	1.06688400	1.12031500
H	-3.24383900	-0.43174300	1.63657900
O	-4.10622200	-0.41065300	-0.25500400
H	-3.56243300	-1.18157400	-0.48249800

57$_{RC}$

M06-2X/6-31G* with Solvent Correction

SCF Done: E(RM062X) = -668.058817393

Zero-point correction= 0.222217
Thermal correction to Gibbs Free Energy= 0.185578

H	2.58419600	2.31266800	0.21320300
C	2.03254800	1.37134700	0.28113500
H	1.73569200	1.21381200	1.32877800
O	2.93547400	0.37292400	-0.14333100
C	2.51092700	-0.94003600	0.14753800
C	1.23080100	-1.36477500	-0.56802300
H	1.14892900	-2.45521300	-0.49973900
H	1.27917600	-1.11372200	-1.63216600
C	-0.07749500	-0.83428400	0.02233700
C	0.79283700	1.47308500	-0.60612300
O	-0.08733400	-0.96547700	1.43117800
H	-0.10679500	-1.91727600	1.62516400
N	-0.30181600	0.57436900	-0.24954800
O	-1.08258200	-1.64445800	-0.52786800
H	3.32658300	-1.58973200	-0.17917600
H	2.38134000	-1.06650800	1.23164500
H	0.37305100	2.47692500	-0.53365600
H	1.09648400	1.30596700	-1.64591000
C	-1.52346200	1.11185000	0.08742600
O	-1.75445100	2.30991800	0.07380600
C	-2.57328200	0.06850700	0.40376600
H	-3.55451200	0.52911000	0.27815400
H	-2.46023200	-0.25440200	1.44163900
C	-2.40470900	-1.10833200	-0.54896800
H	-3.08352200	-1.92483600	-0.29301200
H	-2.62552500	-0.78479300	-1.57288900

57$_{RE}$

M06-2X/6-31G* with Solvent Correction

SCF Done: E(RM062X) = -668.079361193

Zero-point correction= 0.222102
Thermal correction to Gibbs Free Energy= 0.184124

H	-0.30780400	1.72473300	-1.28904900
C	-0.04970400	2.24557900	-0.35758100
H	0.11946500	3.30753000	-0.58209200
O	1.09227700	1.65615500	0.23154800
C	2.04864000	1.12391700	-0.66294200
C	2.54802000	-0.18284200	-0.06104100
H	3.07181700	-0.00628100	0.88015300
H	3.23542800	-0.67849200	-0.75539700
C	-1.19495600	2.07401300	0.63257900
N	-1.36177100	0.66908100	0.96590600
O	0.56307500	-1.22613900	-0.81374900
H	1.59265800	0.93061700	-1.64061400
H	2.88135500	1.82425900	-0.80296200
H	-0.99253600	2.62388400	1.55444500
H	-2.12504600	2.44546100	0.19812500
C	-1.87692200	-0.18863900	0.04524400
O	-2.47699000	0.18753800	-0.95330300
C	-1.59740300	-1.65634200	0.31034500
H	-2.51489800	-2.22446200	0.13516700
H	-1.26455000	-1.82743300	1.33490900
C	-0.54075400	-2.13979000	-0.68425200
H	-0.69806000	0.28580700	1.62761200
C	1.39481000	-1.11677700	0.23453400
O	1.22993000	-1.70981000	1.27557500
H	-0.15984700	-3.12209100	-0.39526800
H	-0.96231000	-2.18376500	-1.68825500

Isomer 59_{RO}/59_{RC}/59_{RE}

Spartan Calculations

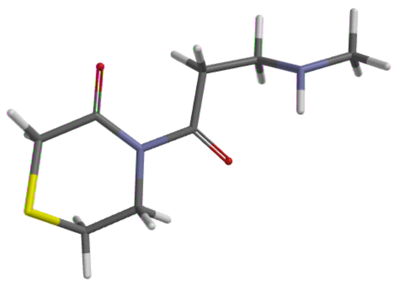

59_{RO}
$\Delta G° = 6.7$ kcal/mol

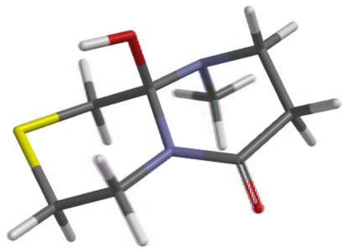

59_{RC}
$\Delta G° = 18.4$ kcal/mol

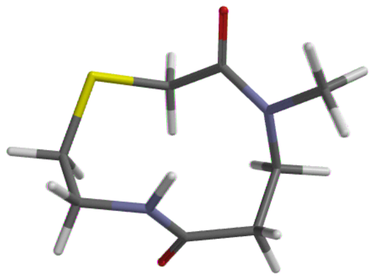

59_{RE}
$\Delta G° = 0.0$ kcal/mol

59_{RO}	Calculated energy (au)	Calculated energy (kcal/mol)	Relative energy (kcal/mol)
M0027	-971.42149	-609566.9869	0.00
M0011	-971.42093	-609566.6317	0.36
M0041	-971.41924	-609565.5725	1.41
M0040	-971.41912	-609565.4953	1.49
M0038	-971.41895	-609565.3936	1.59
M0002	-971.41890	-609565.3572	1.63
M0001	-971.41854	-609565.1320	1.85
M0033	-971.41823	-609564.9381	2.05
M0004	-971.41789	-609564.7228	2.26
M0005	-971.41778	-609564.6576	2.33
M0006	-971.41772	-609564.6193	2.37
M0014	-971.41751	-609564.4869	2.50
M0021	-971.41748	-609564.4681	2.52
M0035	-971.41733	-609564.3733	2.61
M0008	-971.41723	-609564.3112	2.68
M0003	-971.41719	-609564.2855	2.70
M0013	-971.41695	-609564.1355	2.85
M0031	-971.41667	-609563.9573	3.03
M0007	-971.41652	-609563.8663	3.12
M0051	-971.41644	-609563.8186	3.17
M0009	-971.41593	-609563.4954	3.49
M0032	-971.41587	-609563.4603	3.53
M0012	-971.41584	-609563.4377	3.55
M0017	-971.41572	-609563.3624	3.62
M0018	-971.41572	-609563.3624	3.62
M0016	-971.41551	-609563.2344	3.75
M0029	-971.41540	-609563.1641	3.82
M0052	-971.41529	-609563.0970	3.89
M0050	-971.41527	-609563.0807	3.91

M0039	-971.41500	-609562.9112	4.08
M0030	-971.41490	-609562.8472	4.14
M0015	-971.41469	-609562.7186	4.27
M0045	-971.41468	-609562.7092	4.28
M0019	-971.41417	-609562.3942	4.59
M0020	-971.41397	-609562.2643	4.72
M0028	-971.41392	-609562.2323	4.75
M0044	-971.41389	-609562.2153	4.77
M0047	-971.41386	-609562.1965	4.79
M0048	-971.41284	-609561.5577	5.43
M0049	-971.41252	-609561.3588	5.63
M0034	-971.41251	-609561.3507	5.64
M0046	-971.41243	-609561.2967	5.69
M0026	-971.41189	-609560.9585	6.03
M0010	-971.40806	-609558.5551	8.43
M0024	-971.40614	-609557.3554	9.63
M0022	-971.40611	-609557.3309	9.66
M0042	-971.40473	-609556.4674	10.52
M0023	-971.40471	-609556.4536	10.53
M0037	-971.40361	-609555.7659	11.22
M0025	-971.40333	-609555.5889	11.40
M0043	-971.40305	-609555.4145	11.57
M0036	-971.40285	-609555.2871	11.70
M0053	-971.40180	-609554.6308	12.36
**59_{RC}**	**Calculated energy (au)**	**Calculated energy (kcal/mol)**	**Relative energy (kcal/mol)**
M0004	-971.40903	-609559.1657	0.00
M0002	-971.40881	-609559.0264	0.14
M0005	-971.40706	-609557.9295	1.24
M0003	-971.40700	-609557.8944	1.27
M0008	-971.40592	-609557.2129	1.95
M0001	-971.40430	-609556.1976	2.97
M0006	-971.40394	-609555.9724	3.19
M0009	-971.39983	-609553.3946	5.77
M0007	-971.39974	-609553.3369	5.83
**59_{RE}**	**Calculated energy (au)**	**Calculated energy (kcal/mol)**	**Relative energy (kcal/mol)**
M0002	-971.43745	-609576.9980	0.00
M0001	-971.43670	-609576.5274	0.47
M0003	-971.43384	-609574.7371	2.26
M0004	-971.43352	-609574.5351	2.46
M0006	-971.43303	-609574.2238	2.77
M0005	-971.43248	-609573.8812	3.12
M0007	-971.42877	-609571.5551	5.44

Table S26. Energies calculated at DFT/B3LYP/6-31G* in vacuum for the conformers found for **59_{RO}/59_{RC}/59_{RE}**.

Isomer	ΔG° (au)	ΔG° (kcal/mol)	ΔG° (kcal/mol)
**59_{RO}**	-971.246915	-609457.44	6.7
**59_{RC}**	-971.228242	-609445.72	18.4
**59_{RE}**	-971.257548	-609464.11	0.0

Table S27. Relative free energies (ΔG°) of the lowest energy geometries of **59_{RO}/59_{RC}/59_{RE}** at DFT/B3LYP/6-31G* in vacuum in kcal/mol.

XYZ Coordinates

59$_{RO}$

C	1.23063000	1.07021100	-0.12837900
S	3.75893100	-0.14367800	-0.20390900
C	1.37109100	-1.41226900	-0.12149900
C	2.74899100	-1.44860800	0.51510100
N	0.60979100	-0.16948900	0.05223100
C	2.69034000	1.22149200	0.27314100
H	1.44084100	-1.59937900	-1.20175900
H	2.68856100	-1.30929800	1.59950100
H	2.73456000	1.36321200	1.35792100
H	0.79719100	-2.24552900	0.30306100
H	3.22834100	-2.41386800	0.32553100
H	3.08406000	2.12797200	-0.19815900
O	0.62636000	2.08103100	-0.49642900
C	-0.78410900	-0.33520900	-0.04002900
O	-1.28516900	-1.41124900	-0.38390900
C	-1.66505000	0.86182100	0.29741100
H	-1.70876000	1.53333100	-0.56658900
H	-1.21834000	1.39637100	1.14370100
C	-3.08487000	0.45148100	0.70035100
H	-3.04153900	-0.35964900	1.43772100
H	-3.56717000	1.31313100	1.17731100
N	-3.88204900	0.04529100	-0.46156900
H	-3.40702900	-0.74482900	-0.91137900
C	-5.21788900	-0.37441000	-0.06104900
H	-5.18307900	-1.23147000	0.61939100
H	-5.78739900	-0.67131000	-0.94713900
H	-5.76165000	0.44425000	0.42124100

59$_{RC}$

C	0.00441000	-0.64441100	0.32536000
S	2.81930000	-0.38592800	0.17645000
C	0.97205800	1.62519000	-0.18288000
C	2.28845800	1.08785100	-0.73402000
N	-0.17583100	0.70435900	-0.20991000
C	1.31012100	-1.35934000	-0.15867000
H	1.09953800	1.96511000	0.85369000
H	2.18843900	0.83170100	-1.79406000
H	1.30386100	-1.54223000	-1.23714000
H	0.71795700	2.51741000	-0.76886000
H	3.06876800	1.85038200	-0.64792000
H	1.42806100	-2.31982000	0.35524000
O	0.10452000	-0.49675100	1.73758000
H	1.02227000	-0.22713000	1.91028000
N	-1.16144900	-1.47915200	0.02686000
C	-1.32255900	-1.84988200	-1.37768000
H	-0.54543800	-2.55365200	-1.69147000
H	-1.30582000	-0.99035200	-2.05598000
H	-2.26901800	-2.38171300	-1.53193000
C	-2.37436000	-0.87943300	0.57565000
H	-3.23673900	-1.53659400	0.40619000
C	-1.41020200	1.34103800	-0.06173000
O	-1.53198300	2.56896800	-0.08677000
H	-2.29730000	-0.80349300	1.66780000
C	-2.64566100	0.48181700	-0.01405000
H	-3.03317100	0.40004600	-1.03597000
H	-3.40312200	0.99403600	0.58990000

59RE

C	-1.05017000	-1.36885000	-0.20058100
S	1.68869100	-1.80629900	-0.16585100
C	2.57261000	-0.20862900	-0.03746100
N	-1.87278000	-0.24811000	-0.06484100
C	0.19202000	-1.29350000	-1.09187100
H	0.36360000	-0.32134900	-1.54461100
H	2.52252000	0.31028100	-0.99978100
H	0.03940100	-2.00483000	-1.91052100
O	-1.31645900	-2.41551000	0.39957900
H	3.62739000	-0.44805800	0.13584900
C	2.08670000	0.67873100	1.11355900
H	2.18604000	0.16115100	2.07390900
H	2.71052900	1.57906100	1.15614900
N	0.70614000	1.10226100	0.99111900
C	-1.79123000	0.98495000	-0.86139100
H	-1.25331000	0.80449000	-1.79484100
H	-2.81601100	1.24481900	-1.15485100
C	-1.18195100	2.15569000	-0.09457100
H	-1.61843100	2.26610000	0.90393900
H	-1.37440100	3.09067000	-0.63362100
C	0.31234900	2.00038000	0.02744900
O	1.09303900	2.58909100	-0.71248100
H	0.00033000	0.53850000	1.45369900
C	-3.04203000	-0.36770100	0.80097900
H	-2.94563000	-1.19029100	1.51431900
H	-3.92045000	-0.54403100	0.17297900
H	-3.17657000	0.56013900	1.36361900

Gaussian Calculations

59$_{RO}$

M06-2X/6-31G* with Solvent Correction

SCF Done: E(RM062X) = -971.157652742

Zero-point correction= 0.228640
Thermal correction to Gibbs Free Energy= 0.186316

C	1.23989700	1.12704200	0.13366500
S	3.51175200	-0.28948000	-0.81995300
C	1.30852900	-1.41536400	0.36159000
C	2.81613200	-1.34483800	0.47572200
N	0.61405500	-0.10463300	0.32201200
C	2.73983000	1.18795700	-0.12624300
H	1.02805900	-1.96856700	-0.53860200
H	3.13414200	-0.96580800	1.45192200
H	3.22372300	1.44580700	0.82265300
H	0.90550100	-1.95958300	1.21791200
H	3.21804000	-2.35244300	0.35440300
H	2.89001500	2.02465500	-0.80933000
O	0.64251100	2.18315800	0.19799100
C	-0.79193000	-0.24401800	0.41706700
O	-1.23983900	-1.37067900	0.53154200
C	-1.71048200	0.95915100	0.36779600
H	-1.52274500	1.51955600	-0.55213900
H	-1.45061500	1.63402300	1.18719500
C	-3.17394200	0.54338100	0.44562600
H	-3.34978900	-0.03483100	1.36875600
H	-3.77690600	1.45593600	0.51980900
N	-3.59594400	-0.17237500	-0.75206100
H	-3.07672000	-1.04774700	-0.77512700
C	-5.01911200	-0.48269200	-0.71118500
H	-5.32890700	-1.02481700	0.19798100
H	-5.28891500	-1.08706100	-1.58099200
H	-5.59460100	0.44807100	-0.75533500

59$_{RC}$

M06-2X/6-31G* with Solvent Correction

SCF Done: E(RM062X) = -971.163072769

Zero-point correction= 0.230792
Thermal correction to Gibbs Free Energy= 0.193504

C	0.01546400	-0.66267200	0.33272300
S	2.78107500	-0.43499000	0.08085500
C	0.97405800	1.66225300	0.03356000
C	2.25385800	1.16275300	-0.61262800
N	-0.16507600	0.74822600	-0.13349600
C	1.24114600	-1.28050900	-0.36196900
H	1.14525100	1.84695600	1.10314500
H	2.12680300	1.04889500	-1.69363200
H	1.13060700	-1.24356000	-1.44716000
H	0.68429100	2.60926000	-0.42073000
H	3.05897800	1.87751800	-0.43007500
H	1.31781200	-2.32090100	-0.03828000
O	0.19274800	-0.66796300	1.72576600
H	1.10910700	-0.39276600	1.90620600
N	-1.14558200	-1.47562000	0.06648200
C	-1.33589200	-1.84209800	-1.33727600
H	-0.61067800	-2.60114800	-1.63719600
H	-1.26284600	-0.99329500	-2.03382400
H	-2.33078100	-2.28243500	-1.43633000
C	-2.33997800	-0.85898000	0.64406800
H	-3.17861800	-1.55046800	0.52805000
C	-1.40135300	1.36226600	-0.10886300
O	-1.52006100	2.57733500	-0.19480900
H	-2.16539700	-0.72097100	1.71309500
C	-2.63056100	0.47771300	-0.02798200
H	-2.99054900	0.32604500	-1.05296600
H	-3.39852900	1.04914000	0.49765700

59RE

M06-2X/6-31G* with Solvent Correction

SCF Done: E(RM062X) = -971.180449083

Zero-point correction= 0.229881
Thermal correction to Gibbs Free Energy= 0.190005

C	-1.07519800	-1.29655800	-0.19904700
S	1.64879900	-1.76219000	-0.17961100
C	2.60515800	-0.22171100	-0.01891500
N	-1.91334700	-0.23099700	-0.06791100
C	0.16464300	-1.17321200	-1.07834700
H	0.37609300	-0.17573200	-1.46202500
H	2.58193900	0.31721400	-0.96811600
H	0.01560400	-1.84108900	-1.93124500
O	-1.29332700	-2.35811100	0.37414900
H	3.63628100	-0.53823500	0.16264400
C	2.16034500	0.69305400	1.12168700
H	2.28671900	0.18753400	2.08055000
H	2.79542500	1.58608300	1.11593300
N	0.76936500	1.10446300	1.02629100
C	-1.84724400	1.01506200	-0.82109400
H	-1.34581600	0.86049900	-1.77840100
H	-2.87410500	1.31142800	-1.05356500
C	-1.15734700	2.14535100	-0.03817300
H	-1.59367300	2.21995300	0.96331800
H	-1.31182000	3.09246200	-0.55925400
C	0.33580800	1.89294100	0.01648300
O	1.09088200	2.30531300	-0.86056800
H	0.10711400	0.76394400	1.70914200
C	-3.07362700	-0.38905300	0.80144000
H	-2.82881300	-1.07538600	1.61004100
H	-3.93081000	-0.78557500	0.24607400
H	-3.34271100	0.58481600	1.21716500

Isomer 61_{RO}/61_{RC}/61_{RE}

Spartan Calculations

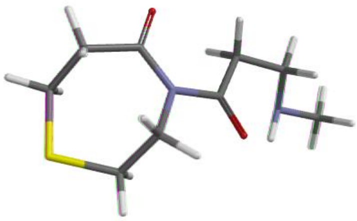

61_{RO}
ΔG ° = 10.9 kcal/mol

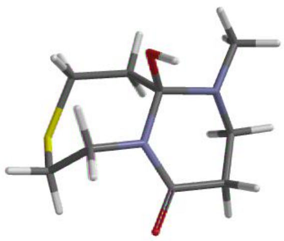

61_{RC}
ΔG ° = 20.3 kcal/mol

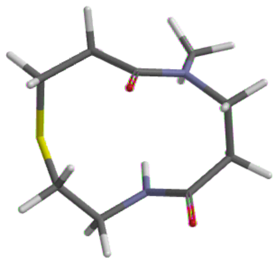

61_{RE}
ΔG ° = 0.0 kcal/mol

61_{RO}	Calculated energy (au)	Calculated energy (kcal/mol)	Relative energy (kcal/mol)
M0015	-1010.73464	-634235.9866	0.00
M0017	-1010.73461	-634235.9678	0.02
M0018	-1010.73461	-634235.9678	0.02
M0013	-1010.73371	-634235.4030	0.58
M0088	-1010.73343	-634235.2273	0.76
M0089	-1010.73343	-634235.2273	0.76
M0095	-1010.73307	-634235.0014	0.99
M0047	-1010.73255	-634234.6751	1.31
M0001	-1010.73193	-634234.2861	1.70
M0045	-1010.73183	-634234.2233	1.76
M0002	-1010.73183	-634234.2233	1.76
M0003	-1010.73183	-634234.2233	1.76
M0038	-1010.73152	-634234.0288	1.96
M0007	-1010.73093	-634233.6586	2.33
M0008	-1010.73093	-634233.6586	2.33
M0051	-1010.73083	-634233.5958	2.39
M0052	-1010.73083	-634233.5958	2.39
M0060	-1010.73074	-634233.5394	2.45
M0061	-1010.73074	-634233.5394	2.45
M0048	-1010.73051	-634233.3950	2.59
M0004	-1010.73045	-634233.3574	2.63
M0034	-1010.73044	-634233.3511	2.64
M0035	-1010.73029	-634233.2570	2.73
M0030	-1010.73019	-634233.1942	2.79
M0005	-1010.73014	-634233.1629	2.82
M0006	-1010.73014	-634233.1629	2.82
M0014	-1010.73008	-634233.1252	2.86
M0009	-1010.73000	-634233.0750	2.91
M0010	-1010.73000	-634233.0750	2.91
M0011	-1010.72985	-634232.9809	3.01
M0012	-1010.72985	-634232.9809	3.01

M0019	-1010.72957	-634232.8052	3.18
M0020	-1010.72957	-634232.8052	3.18
M0049	-1010.72946	-634232.7362	3.25
M0036	-1010.72944	-634232.7236	3.26
M0037	-1010.72944	-634232.7236	3.26
M0076	-1010.72930	-634232.6358	3.35
M0041	-1010.72926	-634232.6107	3.38
M0042	-1010.72926	-634232.6107	3.38
M0080	-1010.72922	-634232.5856	3.40
M0081	-1010.72922	-634232.5856	3.40
M0028	-1010.72918	-634232.5605	3.43
M0029	-1010.72918	-634232.5605	3.43
M0069	-1010.72911	-634232.5165	3.47
M0077	-1010.72890	-634232.3848	3.60
M0073	-1010.72875	-634232.2906	3.70
M0074	-1010.72875	-634232.2906	3.70
M0065	-1010.72866	-634232.2342	3.75
M0043	-1010.72864	-634232.2216	3.77
M0027	-1010.72842	-634232.0836	3.90
M0033	-1010.72841	-634232.0773	3.91
M0046	-1010.72839	-634232.0647	3.92
M0031	-1010.72832	-634232.0208	3.97
M0032	-1010.72832	-634232.0208	3.97
M0021	-1010.72825	-634231.9769	4.01
M0022	-1010.72825	-634231.9769	4.01
M0016	-1010.72808	-634231.8702	4.12
M0059	-1010.72785	-634231.7259	4.26
M0025	-1010.72781	-634231.7008	4.29
M0026	-1010.72781	-634231.7008	4.29
M0024	-1010.72776	-634231.6694	4.32
M0044	-1010.72774	-634231.6569	4.33
M0023	-1010.72764	-634231.5941	4.39
M0053	-1010.72714	-634231.2804	4.71
M0054	-1010.72714	-634231.2804	4.71
M0057	-1010.72713	-634231.2741	4.71
M0058	-1010.72713	-634231.2741	4.71
M0091	-1010.72696	-634231.1674	4.82
M0092	-1010.72696	-634231.1674	4.82
M0039	-1010.72668	-634230.9917	4.99
M0040	-1010.72668	-634230.9917	4.99
M0066	-1010.72667	-634230.9854	5.00
M0098	-1010.72660	-634230.9415	5.05
M0050	-1010.72565	-634230.3454	5.64
M0070	-1010.72552	-634230.2638	5.72
M0062	-1010.72533	-634230.1446	5.84
M0064	-1010.72520	-634230.0630	5.92

M0075	-1010.72493	-634229.8936	6.09
M0063	-1010.72462	-634229.6991	6.29
M0090	-1010.72011	-634226.8690	9.12
M0096	-1010.72001	-634226.8063	9.18
M0099	-1010.71804	-634225.5701	10.42
M0055	-1010.71742	-634225.1811	10.81
M0056	-1010.71742	-634225.1811	10.81
M0071	-1010.71602	-634224.3026	11.68
M0072	-1010.71602	-634224.3026	11.68
M0067	-1010.71591	-634224.2335	11.75
M0068	-1010.71591	-634224.2335	11.75
M0087	-1010.71479	-634223.5307	12.46
M0082	-1010.71477	-634223.5182	12.47
M0083	-1010.71477	-634223.5182	12.47
M0085	-1010.71475	-634223.5056	12.48
M0086	-1010.71475	-634223.5056	12.48
M0097	-1010.71459	-634223.4052	12.58
M0078	-1010.71443	-634223.3048	12.68
M0079	-1010.71443	-634223.3048	12.68
M0093	-1010.71395	-634223.0036	12.98
M0100	-1010.71284	-634222.3071	13.68
M0094	-1010.71248	-634222.0812	13.91
M0084	-1010.71197	-634221.7612	14.23
**61_{RC}**	**Calculated energy (au)**	**Calculated energy (kcal/mol)**	**Relative energy (kcal/mol)**
M0001	-1010.72181	-634227.9358	0.00
M0002	-1010.71771	-634225.3630	2.57
M0003	-1010.71375	-634222.8781	5.06
M0005	-1010.71302	-634222.4201	5.52
M0011	-1010.71293	-634222.3636	5.57
M0008	-1010.71287	-634222.3259	5.61
M0006	-1010.71087	-634221.0709	6.86
M0004	-1010.70957	-634220.2552	7.68
M0007	-1010.70872	-634219.7218	8.21
M0009	-1010.70869	-634219.7030	8.23
M0012	-1010.70719	-634218.7617	9.17
M0010	-1010.70650	-634218.3288	9.61
**61_{RE}**	**Calculated energy (au)**	**Calculated energy (kcal/mol)**	**Relative energy (kcal/mol)**
M0001	-1010.75490	-634248.6998	0.00
M0004	-1010.75429	-634248.3170	0.38
M0003	-1010.75288	-634247.4322	1.27
M0002	-1010.75272	-634247.3318	1.37
M0010	-1010.75161	-634246.6353	2.06
M0011	-1010.75129	-634246.4345	2.27
M0005	-1010.75038	-634245.8635	2.84
M0008	-1010.75017	-634245.7317	2.97

M0007	-1010.74985	-634245.5309	3.17
M0013	-1010.74966	-634245.4117	3.29
M0006	-1010.74872	-634244.8218	3.88
M0012	-1010.74840	-634244.6210	4.08
M0009	-1010.74840	-634244.6210	4.08
M0021	-1010.74685	-634243.6484	5.05
M0015	-1010.74672	-634243.5668	5.13
M0016	-1010.74624	-634243.2656	5.43
M0014	-1010.74610	-634243.1778	5.52
M0020	-1010.74568	-634242.9142	5.79
M0022	-1010.74395	-634241.8286	6.87
M0018	-1010.74319	-634241.3517	7.35
M0017	-1010.74317	-634241.3392	7.36
M0019	-1010.74312	-634241.3078	7.39
M0023	-1010.74089	-634239.9085	8.79
M0025	-1010.73992	-634239.2998	9.40
M0024	-1010.73789	-634238.0260	10.67

Table S28. Energies calculated at DFT/B3LYP/6-31G* in vacuum for the conformers found for **61_{RO}/61_{RC}/61_{RE}**.

Isomer	ΔG° (au)	ΔG° (kcal/mol)	ΔG° (kcal/mol)
**61_{RO}**	-1010.53005	-634107.61	10.9
**61_{RC}**	-1010.51516	-634098.26	20.3
**61_{RE}**	-1010.54754	-634118.58	0.00

Table S29. Relative free energies (ΔG°) of the lowest energy geometries of **61_{RO}/61_{RC}/61_{RE}** at DFT/B3LYP/6-31G* in vacuum in kcal/mol.

XYZ Coordinates

61$_{RO}$

H	-3.15527100	1.37611900	0.40404000
C	-2.41608100	1.40177000	-0.40276000
H	-2.60485100	2.33265000	-0.95484000
C	-2.53611000	0.25254000	-1.39146000
H	-1.59326000	0.04415000	-1.90838000
H	-3.27143000	0.52082900	-2.15767000
S	-3.15523000	-1.27673100	-0.62241000
C	-1.75097000	-1.69867000	0.46238000
H	-0.90613900	-2.01534000	-0.15768000
H	-2.07083900	-2.56802000	1.04643000
C	-1.36669000	-0.57299000	1.42171000
H	-2.25110000	-0.07756000	1.83861000
H	-0.84656000	-1.01400000	2.28123000
N	-0.48143000	0.42295100	0.81186000
C	-1.01257100	1.52750000	0.15618000
O	-0.42636100	2.60675100	0.06379000
C	0.88575000	0.09899100	0.80592000
O	1.29468000	-0.95410900	1.30688000
C	1.84452900	1.06271200	0.12102000
H	1.49517900	1.26561100	-0.89756000
H	1.84723900	1.99647200	0.69426000
C	3.28490000	0.54868200	0.06894000
H	3.62307000	0.26742200	1.07378000
H	3.92364900	1.36897200	-0.28046000
N	3.41399000	-0.59047800	-0.84924000
H	2.87243000	-1.36810800	-0.45934000
C	4.80593000	-1.01222700	-0.95552000
H	5.43021000	-0.21424700	-1.37023000
H	5.20950000	-1.31570700	0.01600000
H	4.87526100	-1.86984700	-1.63179000

61_{RC}

H	2.60170900	1.64481000	-0.05235000
C	2.50557900	0.66461000	0.42519000
H	3.39766000	0.54758000	1.05076000
S	2.62882000	-0.56663000	-0.91703000
C	1.46475000	-1.84317000	-0.35673000
C	0.01516000	-1.47925000	-0.67518000
H	-0.50976000	-2.44095100	-0.74455000
H	-0.04389000	-1.02071000	-1.66893000
C	-0.69050000	-0.61775100	0.39460000
C	1.27156900	0.63656000	1.33291000
O	-0.64777000	-1.31929100	1.62037000
H	-1.27727000	-0.80358100	2.15232000
N	-0.02561100	0.65032000	0.64815000
N	-2.10642000	-0.35382100	0.14751000
H	1.72097000	-2.74006000	-0.93261000
H	1.63165000	-2.10065000	0.69313000
H	1.33256000	-0.20469000	2.03047000
H	1.30859900	1.53294000	1.96671000
C	-0.35907100	1.81813900	-0.01706000
O	0.36905900	2.81348000	-0.02095000
C	-1.72699100	1.90523900	-0.63114000
H	-1.66905100	2.52383900	-1.53349000
H	-2.37451100	2.41765900	0.09079000
C	-2.29553000	0.55501900	-0.97983000
C	-2.91217000	-1.56138100	-0.05275000
H	-3.97715000	-1.30092200	-0.05813000
H	-2.78167000	-2.27784100	0.76516000
H	-2.69212000	-2.06760100	-0.99893000
H	-3.36459100	0.68884900	-1.18987000
H	-1.84713000	0.17030900	-1.90307000

61$_{\mathbf{RE}}$

H	2.62525600	2.19223600	0.70311900
C	1.80887000	1.53807100	0.38093900
H	1.33498300	1.13346800	1.27757900
S	2.55499800	0.20275500	-0.63842100
C	2.36922700	-1.23039600	0.48985900
C	1.12774200	-2.05217300	0.14702900
H	1.21222800	-3.04315300	0.60906900
H	1.07907300	-2.19935400	-0.93474100
C	0.80097500	2.34960500	-0.43891100
N	-0.37076000	1.57129700	-0.80547100
N	-1.30609100	-1.53973800	-0.04058100
H	3.25194100	-1.85822000	0.32621900
H	2.40000500	-0.90745500	1.53482900
H	0.48657000	3.22493300	0.13980900
H	1.25279300	2.71490700	-1.36783100
C	-1.52210000	1.59418000	-0.04936100
O	-1.70950500	2.36639900	0.88516900
C	-2.56140400	0.58062400	-0.47286100
H	-2.48040300	0.38579400	-1.54800100
H	-3.54402700	1.03928800	-0.31004100
C	-2.45308600	-0.70510500	0.34135900
C	-1.41033600	-2.28343900	-1.28790100
H	-1.06522000	-1.64732700	-2.10761100
H	-2.44933500	-2.57788500	-1.46112100
H	-0.80293100	-3.19101500	-1.24629100
H	-2.40107800	-0.46562500	1.40949900
H	-3.35759200	-1.30833100	0.20073900
H	-0.22181500	0.80221800	-1.44524100
C	-0.13747200	-1.42857100	0.70600900
O	-0.11511500	-0.85843100	1.79734900

Gaussian Calculations

61_{RO}

M06-2X/6-31G* with Solvent Correction

SCF Done: E(RM062X) = -1010.45369938

Zero-point correction= 0.258114
Thermal correction to Gibbs Free Energy= 0.215607

H	-3.14041300	1.32707500	0.59062500
C	-2.47209900	1.43486800	-0.26935800
H	-2.69448900	2.39508500	-0.73919600
C	-2.70936900	0.30076100	-1.28138000
H	-1.83766000	0.17244500	-1.93125800
H	-3.55135300	0.57103600	-1.92263000
S	-3.19039800	-1.30620700	-0.57834500
C	-1.71352600	-1.71806600	0.39912500
H	-0.87706200	-1.97511500	-0.25756000
H	-1.98958700	-2.61677600	0.95673600
C	-1.28806800	-0.62148300	1.37221500
H	-2.15526400	-0.14326200	1.82940900
H	-0.68920600	-1.06039100	2.16896500
N	-0.44461200	0.41025800	0.74924700
C	-1.01930100	1.54671800	0.16117500
O	-0.38283500	2.55660200	-0.05569400
C	0.93757400	0.14642200	0.75682900
O	1.33040700	-0.89186200	1.26090000
C	1.90198200	1.11962500	0.10976900
H	1.55613500	1.35558800	-0.90086900
H	1.87321300	2.05988400	0.66627800
C	3.31515200	0.55402300	0.06529600
H	3.66103200	0.33329800	1.08920700
H	3.97577200	1.33128500	-0.33595600
N	3.40129800	-0.60410700	-0.81602000
H	2.84610600	-1.34407500	-0.39050400
C	4.77454800	-1.07569400	-0.94173700
H	5.37168700	-0.31871200	-1.46110900
H	5.26283300	-1.28181300	0.02536900
H	4.79589800	-1.99026700	-1.53983200

61_{RC}

M06-2X/6-31G* with Solvent Correction

SCF Done: E(RM062X) = -1010.45871712

Zero-point correction= 0.260233
Thermal correction to Gibbs Free Energy= 0.221589

H	2.67024500	1.58712200	-0.08071800
C	2.56179300	0.60572600	0.38424600
H	3.43914200	0.41569900	1.00836100
S	2.50834900	-0.59711900	-0.98930500
C	1.42745200	-1.90443200	-0.31842200
C	-0.06991000	-1.64480200	-0.48811500
H	-0.59605400	-2.59186900	-0.32737600
H	-0.25150100	-1.33643600	-1.52271000
C	-0.72507400	-0.61858200	0.45087900
C	1.32010200	0.58620300	1.27039400
O	-0.75252800	-1.22036200	1.71684700
H	-1.28245400	-0.63204200	2.28294200
N	0.05832800	0.63591900	0.53702400
N	-2.09500600	-0.28484400	0.07880200
H	1.68856200	-2.79252700	-0.89976800
H	1.69205200	-2.12147700	0.71990500
H	1.30228000	-0.30475900	1.89542200
H	1.36249100	1.45777000	1.92962900
C	-0.28857000	1.85048300	0.01630700
O	0.43658900	2.83026200	0.15198200
C	-1.61759200	1.95653100	-0.69337400
H	-1.47748600	2.60385300	-1.56204100
H	-2.29973000	2.46761100	-0.00506500
C	-2.18540100	0.60362000	-1.07300900
C	-2.94682300	-1.46060400	-0.08515600
H	-3.98950800	-1.13273500	-0.07184800
H	-2.78699500	-2.14793100	0.74661000
H	-2.76741600	-1.99473500	-1.03085800
H	-3.24132300	0.69894900	-1.33988400
H	-1.66749900	0.19583500	-1.95763500

61RE

M06-2X/6-31G* with Solvent Correction

SCF Done: E(RM062X) = -1010.47925127

Zero-point correction= 0.259108
Thermal correction to Gibbs Free Energy= 0.218061

H	2.56067900	2.22614700	0.68576800
C	1.76242100	1.56310500	0.34319800
H	1.25138300	1.13134500	1.20596800
S	2.50480300	0.19582800	-0.62706700
C	2.37251900	-1.24022300	0.49944500
C	1.12959500	-2.08582800	0.24450000
H	1.18871900	-3.01146900	0.83306000
H	1.09390600	-2.37211700	-0.80750600
C	0.76044900	2.34948300	-0.50054400
N	-0.41324200	1.57468200	-0.84882500
N	-1.25984900	-1.51133500	-0.04015700
H	3.27004500	-1.83896400	0.33142800
H	2.38988200	-0.87880400	1.52928200
H	0.41668700	3.21456900	0.07114900
H	1.22762900	2.71335100	-1.42045100
C	-1.50723600	1.54036700	-0.04444900
O	-1.62546200	2.25035100	0.94654500
C	-2.58615200	0.55071200	-0.45081300
H	-2.55559400	0.34264700	-1.52621900
H	-3.55115500	1.00782800	-0.21785900
C	-2.44901000	-0.75747700	0.34186100
C	-1.31886000	-2.30217800	-1.26528600
H	-0.76310700	-1.83938300	-2.08837000
H	-2.36429900	-2.38658300	-1.56532600
H	-0.93422000	-3.31146800	-1.10272400
H	-2.38144300	-0.53613500	1.40721400
H	-3.32609300	-1.38676300	0.16895600
H	-0.32381300	0.88483400	-1.58250200
C	-0.12571000	-1.34718800	0.69136900
O	-0.09860200	-0.64539600	1.70075200

Isomer 63_{RO}/63_{RC}/63_{RE}

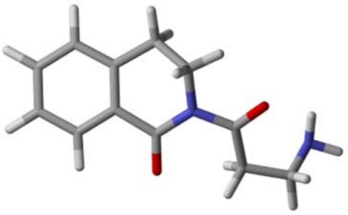

63_{RO}
ΔG ° = 0.0 kcal/mol

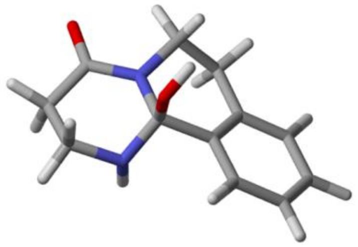

63_{RC}
ΔG ° = 15.9 kcal/mol

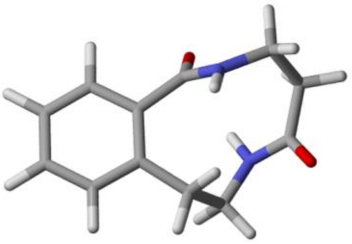

63_{RE}
ΔG ° = 2.5 kcal/mol

Gaussian Calculations

63_{RO}

B3LYP/6-31G*

SCF Done: E(RB3LYP) = -725.689737420

Zero-point correction= 0.249246
Thermal correction to Gibbs Free Energy= 0.207739

H	2.20360800	-2.51856400	-0.33979400
C	2.59781900	-1.52999600	-0.13002600
C	3.53391700	1.03731600	0.43358600
C	1.69991500	-0.45575200	-0.05860400
C	3.95775600	-1.31931700	0.07108500
C	4.42550700	-0.03244300	0.35505300
C	2.16651600	0.83896900	0.22583400
H	4.65261900	-2.15191500	0.00924300
H	5.48737800	0.13818000	0.51139800
H	3.90140000	2.03715000	0.65239400
C	1.15809900	1.95728700	0.30092700
C	-0.00298600	1.67923900	-0.64605300
N	-0.60665700	0.35765600	-0.36611800
C	0.24341400	-0.75582200	-0.23428600
H	1.61975100	2.91610200	0.03858200
H	0.77740200	2.05781400	1.32776200
H	0.34338300	1.70149400	-1.68748800
H	-0.79742500	2.41585600	-0.54436600
O	-0.16432300	-1.90850400	-0.24903500
C	-2.02256700	0.29229700	-0.39775000
O	-2.64895400	1.30089700	-0.69344600
C	-2.72905400	-0.99852400	-0.02317100
H	-2.48560500	-1.76106900	-0.76966700
H	-2.32460800	-1.37333300	0.92236000
C	-4.24333900	-0.80758000	0.08790000
H	-4.68183800	-1.79007600	0.29879300

H	-4.63747100	-0.47928200	-0.88819300
N	-4.58283700	0.08779400	1.20015500
H	-4.34451200	1.03773900	0.91968400
H	-5.59138100	0.07856000	1.34391600

M06-2X/6-31G* with Solvent Correction

SCF Done: E(RM062X) = -725.397292256

Zero-point correction= 0.251861
Thermal correction to Gibbs Free Energy= 0.210496

H	2.19678500	-2.51235000	-0.35152900
C	2.58520300	-1.52231700	-0.13787200
C	3.51378000	1.04294800	0.42825900
C	1.68983100	-0.45283700	-0.05100500
C	3.94371900	-1.30675400	0.04980600
C	4.40628000	-0.02101300	0.33542900
C	2.14859500	0.83852600	0.23303900
H	4.64170600	-2.13402800	-0.02429800
H	5.46785200	0.15280000	0.48171800
H	3.87657500	2.04335300	0.64717700
C	1.13741700	1.94854100	0.31932200
C	-0.01147700	1.67151500	-0.63494000
N	-0.61080600	0.35222500	-0.35592500
C	0.23348900	-0.75326200	-0.21156500
H	1.59633900	2.90885100	0.06759600
H	0.75167200	2.02860100	1.34348700
H	0.33923300	1.69219300	-1.67291500
H	-0.80298000	2.40911100	-0.52919400
O	-0.17258000	-1.90146600	-0.20901100
C	-2.01386500	0.26826900	-0.42174600
O	-2.64698700	1.25145400	-0.76478000
C	-2.71480500	-1.01391800	-0.02122000
H	-2.49223100	-1.77937600	-0.76963800
H	-2.29469500	-1.37709200	0.92070000
C	-4.21688500	-0.79699600	0.11029800
H	-4.67196600	-1.76304400	0.34860200
H	-4.62116400	-0.47480700	-0.86008400
N	-4.51173400	0.12979400	1.20590200
H	-4.26275200	1.06621300	0.89259200
H	-5.51775000	0.14933400	1.35945000

63_{RC}

B3LYP/6-31G*

SCF Done: E(RB3LYP) = -725.667804122

Zero-point correction= 0.250189
Thermal correction to Gibbs Free Energy= 0.211063

N	-1.14990200	0.63229600	0.15764500
C	1.13013000	-0.32653400	0.07446800
C	0.71453800	2.16162300	-0.18050600
C	1.63554100	0.96042900	-0.17166300
C	-0.55220700	1.88306000	0.62294800
C	-0.35133600	-0.60203600	0.34970200
H	1.23367800	3.03903400	0.22440800
H	0.43019400	2.41197700	-1.21249100
C	3.01042200	1.11364800	-0.40005700
C	3.86982700	0.01890000	-0.38504900
C	3.36293700	-1.25817400	-0.12777800
C	2.00168100	-1.42536800	0.10556600
H	3.40520000	2.11013800	-0.58648000
H	4.93246500	0.16092000	-0.56352400
H	4.02751900	-2.11751900	-0.10524500
H	1.58879000	-2.40710000	0.31182000
O	-0.52427600	-1.08303200	1.68563600
H	-0.21040700	-0.39092800	2.28854900
H	-1.30248000	2.66361600	0.49904400
H	-0.31460900	1.81650100	1.69667500
C	-2.48628400	0.63131600	-0.19627100
O	-3.17280300	1.64560900	-0.15664600
N	-0.80659600	-1.69171800	-0.48168200
C	-2.25039000	-1.91880100	-0.36288900
H	-2.44879800	-2.20445300	0.67296900
H	-2.51819100	-2.77126000	-0.99551200
C	-3.05532400	-0.67338100	-0.74404400
H	-3.08133300	-0.55561100	-1.83720500
H	-4.09791200	-0.73440100	-0.41677100
H	-0.54922000	-1.49366100	-1.44645000

M06-2X/6-31G* with Solvent Correction

SCF Done: E(RM062X) = -725.389148288

Zero-point correction= 0.253054
Thermal correction to Gibbs Free Energy= 0.213839

N	-1.15257600	0.61527500	0.20523500
C	1.11916900	-0.32539200	0.08477200
C	0.68627500	2.15534400	-0.14470100
C	1.60910200	0.96025500	-0.16825700
C	-0.54699200	1.85035200	0.69139300
C	-0.35300400	-0.61467400	0.37315700
H	1.21296000	3.02671900	0.25727000
H	0.37082100	2.40851800	-1.16457900
C	2.97516500	1.12185400	-0.42384400
C	3.83877500	0.03415300	-0.42440500
C	3.34624700	-1.24387300	-0.15518200
C	1.99319500	-1.41756500	0.10181600
H	3.35832900	2.12084200	-0.61636700
H	4.89577300	0.18147300	-0.62361100
H	4.01605800	-2.09782200	-0.14366200
H	1.58940800	-2.40196500	0.31541700
O	-0.51016300	-1.10496200	1.69241300
H	-0.17137600	-0.43544200	2.30593600
H	-1.29686100	2.63581800	0.61928700
H	-0.27088900	1.74003500	1.74900900
C	-2.45360100	0.63364300	-0.22017700
O	-3.12583700	1.65802900	-0.22094700
N	-0.80360400	-1.69029700	-0.47095100
C	-2.24630900	-1.90094700	-0.35769100
H	-2.45847200	-2.15415300	0.68345900
H	-2.52210500	-2.76086900	-0.97227200
C	-3.02148600	-0.65846400	-0.77874300
H	-2.99940000	-0.55013400	-1.87076200
H	-4.07326800	-0.70321100	-0.48580500
H	-0.54893700	-1.47732200	-1.43385200

63$_{RE}$

B3LYP/6-31G*

SCF Done: E(RB3LYP) = -725.689755734

Zero-point correction= 0.251084
Thermal correction to Gibbs Free Energy= 0.211734

H	-2.27209400	-2.45537100	0.34560700
C	-2.41849900	-1.39948700	0.13906000
C	-2.73485200	1.30849700	-0.31053300
C	-1.28576200	-0.60246900	-0.08501800
C	-3.70044200	-0.86138500	0.10489300
C	-3.85741600	0.50557700	-0.12306800
C	-1.42864200	0.79012300	-0.30081700
H	-4.56519400	-1.49963500	0.26280000
H	-4.84913400	0.94930100	-0.14763400
H	-2.86859100	2.37605100	-0.46888900
C	-0.28523400	1.78029300	-0.48092800
N	0.91413500	-1.22573200	-0.98654400
C	0.03582700	-1.31847300	0.05124000
H	-0.72236600	2.73802500	-0.78629000
H	0.39033700	1.49463500	-1.29373900
O	0.30265200	-1.96287100	1.06786800
H	0.66323200	-0.64091100	-1.77104500
C	0.59847600	2.06280100	0.76342300
H	-0.02154600	2.26538200	1.64173600
H	1.20092300	2.95097300	0.55699600
C	2.32761400	-1.57664700	-0.82311200
H	2.41455900	-2.64729900	-0.61738000
N	1.51353200	0.97492800	1.08930900
H	1.15306700	0.21980000	1.65997800
C	2.53386200	0.66679400	0.22571500
O	2.93057000	1.44722000	-0.63473000
H	2.81274000	-1.36696800	-1.77882300
C	3.02942600	-0.77008100	0.30555600
H	2.80834200	-1.22439900	1.27400600
H	4.11013600	-0.79199800	0.13975500

M06-2X/6-31G* with Solvent Correction

SCF Done: E(RM062X) = -725.406120829

Zero-point correction= 0.253736
Thermal correction to Gibbs Free Energy= 0.214846

H	-2.23217000	-2.46267900	0.35866200
C	-2.38571700	-1.40795600	0.15119800
C	-2.71646100	1.29137100	-0.31881200
C	-1.26550200	-0.60614900	-0.09027400
C	-3.66788500	-0.87609100	0.12573900
C	-3.83095800	0.48622400	-0.11176900
C	-1.41285400	0.77714300	-0.31896400
H	-4.52819800	-1.51478900	0.29687100
H	-4.82368000	0.92511100	-0.12885800
H	-2.85418600	2.35651000	-0.48446800
C	-0.27371700	1.76322700	-0.50532300
N	0.92927600	-1.18553600	-0.99817300
C	0.06048800	-1.30646400	0.03013500
H	-0.70665100	2.71135600	-0.83892200
H	0.42239200	1.45501300	-1.29067000
O	0.33989300	-1.95290900	1.03919000
H	0.65062000	-0.65938100	-1.81462200
C	0.55858600	2.05359500	0.76096500
H	-0.09122500	2.24101700	1.61872100
H	1.16167500	2.94694100	0.58505000
C	2.33671300	-1.53641000	-0.83441000
H	2.43422700	-2.61114800	-0.66219300
N	1.46265500	0.96435200	1.09864200
H	1.11225500	0.21913000	1.68885500
C	2.48468600	0.66408800	0.25130200
O	2.87603700	1.44757600	-0.60690200
H	2.83394500	-1.28766300	-1.77250700
C	2.99876400	-0.75862300	0.32806900
H	2.75088100	-1.22612100	1.28208700
H	4.08229800	-0.76607300	0.19326100

Isomer 64_{RO}/64_{RC}/64_{RE}

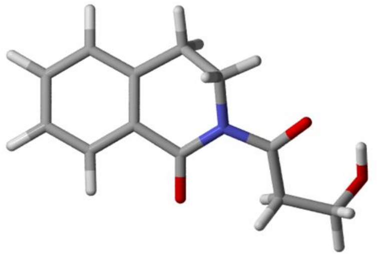

64_{RO}
$\Delta G°$ = 0.0 kcal/mol

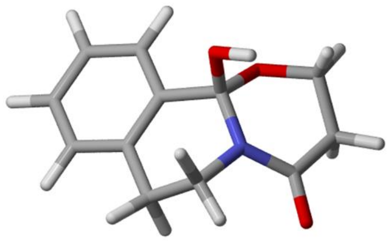

64_{RC}
$\Delta G°$ = 13.9 kcal/mol

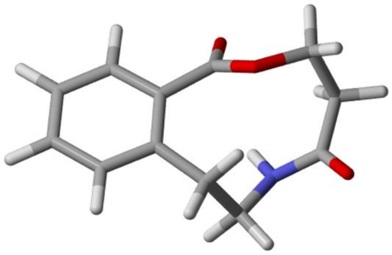

64_{RE}
$\Delta G°$ = 3.3 kcal/mol

Gaussian Calculations

64_{RO}

B3LYP/6-31G*

SCF Done: E(RB3LYP) = -745.556627474

Zero-point correction=   0.236669
Thermal correction to Gibbs Free Energy=   0.195354

H	2.20127100	-2.52209300	-0.32626400
C	2.59903600	-1.53065800	-0.13795800
C	3.54650100	1.04378200	0.37402400
C	1.70186500	-0.45662600	-0.05110000
C	3.96376800	-1.31572400	0.02091200
C	4.43716700	-0.02542900	0.27904600
C	2.17415100	0.84141400	0.20789400
H	4.65804800	-2.14768500	-0.05352800
H	5.50279400	0.14820900	0.40269600
H	3.91843100	2.04596700	0.57334400
C	1.16589600	1.95797300	0.30329700
C	-0.01790600	1.67336800	-0.61272600
N	-0.61418700	0.35128500	-0.31304300
C	0.24256400	-0.76093100	-0.17987500
H	1.61796700	2.91632800	0.02363500
H	0.81163400	2.06397800	1.33897700
H	0.30242400	1.68854200	-1.66237400
H	-0.80971500	2.41018700	-0.49495500
O	-0.16566400	-1.91261100	-0.15551100
C	-2.02508700	0.28296100	-0.31237500
O	-2.66221300	1.30037000	-0.56706400
C	-2.73350000	-1.01099100	0.04395600
H	-2.43756000	-1.79528000	-0.65900100
H	-2.38056200	-1.35287500	1.02370400
C	-4.25577000	-0.82785500	0.05100900
H	-4.71801600	-1.77597200	0.34433400
H	-4.60181900	-0.59178300	-0.96698900

O	-4.69781000	0.14228000	0.98346600
H	-4.35221100	0.98546400	0.64396900

M06-2X/6-31G* with Solvent Correction

SCF Done: E(RM062X) = -745.260021534

Zero-point correction= 0.239443
Thermal correction to Gibbs Free Energy= 0.198432

H	2.18861800	-2.51610800	-0.34130700
C	2.58281800	-1.52458900	-0.14594700
C	3.52485600	1.04494700	0.37816300
C	1.69031900	-0.45390500	-0.04638300
C	3.94534200	-1.30752400	0.00663100
C	4.41464000	-0.01984900	0.27115200
C	2.15559800	0.83932300	0.21745400
H	4.64136400	-2.13542100	-0.07795300
H	5.47946700	0.15480900	0.39028000
H	3.89291900	2.04668400	0.58173600
C	1.14602900	1.94922600	0.32125000
C	-0.02195800	1.66834800	-0.60829900
N	-0.61721300	0.35005200	-0.31307800
C	0.23129200	-0.75629400	-0.16783100
H	1.59865500	2.90896300	0.05601400
H	0.78175200	2.03352100	1.35293800
H	0.30878200	1.68158000	-1.65283200
H	-0.81085800	2.40698300	-0.49162000
O	-0.17658000	-1.90193900	-0.13161300
C	-2.01674300	0.26804300	-0.34212500
O	-2.66124600	1.26432700	-0.63283500
C	-2.71831800	-1.02177900	0.02897100
H	-2.45259100	-1.79813500	-0.69287600
H	-2.34305300	-1.36930500	0.99642600
C	-4.22839300	-0.81153600	0.07157700
H	-4.70529600	-1.74933000	0.36796400
H	-4.59308200	-0.55136900	-0.93076600
O	-4.61471300	0.16115200	1.02110000
H	-4.27875300	1.00198800	0.67265200

64_{RC}

B3LYP/6-31G*

SCF Done: E(RB3LYP) = -745.537629536

Zero-point correction= 0.237478
Thermal correction to Gibbs Free Energy= 0.198432

N	-1.20636300	0.56459600	0.34679000
C	1.09285300	-0.34520900	0.10668400
C	0.62738600	2.15144000	0.04383600
C	1.55831100	0.96277700	-0.09293400
C	-0.61008400	1.79854000	0.86866100
C	-0.35631700	-0.62541100	0.49235700
H	1.15678800	2.99344200	0.50664000
H	0.30494700	2.49149400	-0.95044900
C	2.90515200	1.15053600	-0.43119300
C	3.77206400	0.06926500	-0.56443200
C	3.30084300	-1.22925900	-0.35509600
C	1.96588800	-1.43206800	-0.01807100
H	3.27326400	2.16240900	-0.58660300
H	4.81364300	0.23837200	-0.82468500
H	3.97121600	-2.07870600	-0.45269000
H	1.58504200	-2.43461700	0.14431400
O	-0.33442100	-1.00874300	1.85779800
H	-1.24909100	-0.98270300	2.18635900
H	-1.37117200	2.57603100	0.81109600
H	-0.33914400	1.63271400	1.91670700
C	-2.36837300	0.63887000	-0.39711300
O	-2.96310800	1.69309000	-0.57812300
O	-0.80504900	-1.67203000	-0.33148100
C	-2.21663500	-1.87449800	-0.30940100
H	-2.57434900	-2.02233500	0.72101200
H	-2.39640700	-2.80236600	-0.85725600
C	-2.88271500	-0.67583800	-0.96579600
H	-2.66859100	-0.67396400	-2.04156900
H	-3.97122600	-0.68533900	-0.85096600

M06-2X/6-31G* with Solvent Correction

SCF Done: E(RM062X) = -745.253224790

Zero-point correction= 0.240485
Thermal correction to Gibbs Free Energy= 0.201665

N	-1.21155800	0.53940000	0.39724600
C	1.07454200	-0.35031800	0.11576800
C	0.59237700	2.13744900	0.09769300
C	1.52475200	0.95881200	-0.07739500
C	-0.60649100	1.74813200	0.95278400
C	-0.36481300	-0.64938900	0.51045300
H	1.13013300	2.97466800	0.55425100
H	0.23244500	2.47943900	-0.88105900
C	2.86135700	1.15851300	-0.43639400
C	3.72995700	0.08574700	-0.59405300
C	3.27248800	-1.21616400	-0.38876600
C	1.94727000	-1.42975200	-0.03265200
H	3.21866400	2.17413700	-0.58713900
H	4.76465600	0.26326300	-0.87067700
H	3.94656100	-2.05861500	-0.50610000
H	1.57196900	-2.43570300	0.12604100
O	-0.33316700	-1.06014700	1.85255700
H	-1.24309600	-1.05731100	2.19241700
H	-1.36800100	2.52601400	0.96632100
H	-0.29481900	1.52757500	1.97788700
C	-2.30500900	0.65688400	-0.42359800
O	-2.83574600	1.73417900	-0.64671700
O	-0.81527900	-1.66789100	-0.32986600
C	-2.22634100	-1.83883100	-0.32149900
H	-2.59790500	-1.95280200	0.70545100
H	-2.42714600	-2.76674900	-0.85748900
C	-2.84219500	-0.63479400	-1.00567800
H	-2.59527800	-0.64377800	-2.07245000
H	-3.93110600	-0.61279300	-0.91593900

64$_{RE}$

B3LYP/6-31G*

SCF Done: E(RB3LYP) = -745.555002786

Zero-point correction= 0.238434
Thermal correction to Gibbs Free Energy= 0.199041

H	-2.32076300	-2.40353400	0.39925800
C	-2.43959900	-1.34598500	0.18712600
C	-2.67236800	1.36330500	-0.30145800
C	-1.28910300	-0.59729100	-0.11311300
C	-3.69865800	-0.75842800	0.21235600
C	-3.81319100	0.60935000	-0.03556100
C	-1.38845500	0.79641900	-0.35392000
H	-4.57772200	-1.35896500	0.42748900
H	-4.78681600	1.09185900	-0.01744900
H	-2.77352800	2.43148800	-0.47757300
C	-0.22102800	1.73759300	-0.59991900
O	0.89408200	-1.01998700	-0.99427400
C	-0.01360300	-1.38033600	-0.06117600
H	-0.62524700	2.67815900	-0.99227200
H	0.45664400	1.34316800	-1.36002000
O	0.18780500	-2.26806300	0.74820900
C	0.62336700	2.09295200	0.65438800
H	-0.01989900	2.39679600	1.48604300
H	1.28053900	2.92885500	0.40532100
C	2.24258800	-1.52169500	-0.80905400
H	2.21771900	-2.60694800	-0.69027300
N	1.47423000	0.99476600	1.10122700
H	1.02871000	0.25917900	1.63455600
C	2.56022600	0.63908500	0.33504600
O	3.10931700	1.41501300	-0.43555900
H	2.74703600	-1.25132600	-1.73700400
C	2.92739400	-0.83925400	0.39334500
H	2.60805000	-1.31164500	1.32628400
H	4.01061500	-0.95044900	0.29168100

M06-2X/6-31G* with Solvent Correction

SCF Done: E(RM062X) = -745.264617273

Zero-point correction=   0.241120
Thermal correction to Gibbs Free Energy=   0.202245

H	-2.24102200	-2.42844100	0.40178100
C	-2.38150800	-1.37398100	0.18683100
C	-2.66915700	1.32335700	-0.31886100
C	-1.25354700	-0.59955700	-0.10899300
C	-3.65108800	-0.81455700	0.19983700
C	-3.79225000	0.54653400	-0.05671300
C	-1.37786700	0.78265400	-0.35922100
H	-4.51796100	-1.43137300	0.41199800
H	-4.77529500	1.00699500	-0.04704800
H	-2.78986000	2.38805400	-0.49953700
C	-0.22634500	1.74097000	-0.58218300
O	0.91760500	-1.00046100	-1.00575400
C	0.04078800	-1.34638900	-0.05334300
H	-0.63723900	2.67653400	-0.97432800
H	0.47945300	1.36196900	-1.32459200
O	0.27521100	-2.19910100	0.77646600
C	0.56223500	2.07535000	0.70178800
H	-0.11405100	2.31683800	1.52510400
H	1.19986800	2.94116800	0.51344700
C	2.26381000	-1.48176000	-0.82406600
H	2.25680100	-2.56787500	-0.72080500
N	1.42051600	0.97462500	1.11442700
H	1.02763300	0.25618200	1.70924300
C	2.48568800	0.64785400	0.32844000
O	2.96511400	1.42796800	-0.48186800
H	2.77337100	-1.19020100	-1.74148100
C	2.91903600	-0.80365600	0.39052000
H	2.60805900	-1.28995700	1.31704800
H	4.00442800	-0.87043700	0.29320400

Isomer 66_{RO}/66_{RC}/66_{RE}

Spartan Calculations

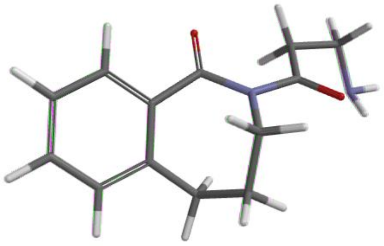

66_{RO}
$\Delta G°$ = 5.9 kcal/mol

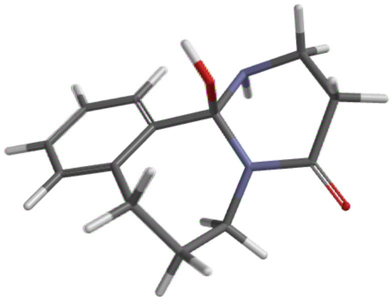

66_{RC}
$\Delta G°$ = 24.2 kcal/mol

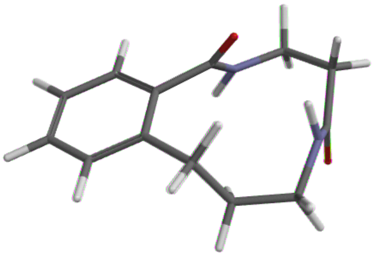

66_{RE}
$\Delta G°$ = 0.0 kcal/mol

66_{RO}	Calculated energy (au)	Calculated energy (kcal/mol)	Relative energy (kcal/mol)
M0010	-764.98912	-480030.6753	0.00
M0017	-764.98900	-480030.6000	0.08
M0018	-764.98900	-480030.6000	0.08
M0011	-764.98829	-480030.1526	0.52
M0012	-764.98829	-480030.1526	0.52
M0019	-764.98786	-480029.8847	0.79
M0016	-764.98737	-480029.5734	1.10
M0005	-764.98720	-480029.4667	1.21
M0001	-764.98619	-480028.8367	1.84
M0002	-764.98619	-480028.8367	1.84
M0009	-764.98579	-480028.5813	2.09
M0008	-764.98553	-480028.4182	2.26
M0020	-764.98551	-480028.4094	2.27
M0003	-764.98537	-480028.3178	2.36
M0004	-764.98537	-480028.3178	2.36
M0026	-764.98506	-480028.1226	2.55
M0006	-764.98501	-480028.0919	2.58
M0022	-764.98469	-480027.8955	2.78
M0023	-764.98454	-480027.7957	2.88
M0007	-764.98398	-480027.4487	3.23
M0013	-764.98378	-480027.3188	3.36
M0021	-764.98303	-480026.8501	3.83
M0025	-764.98298	-480026.8181	3.86
M0031	-764.98264	-480026.6072	4.07
M0014	-764.98252	-480026.5288	4.15
M0015	-764.98252	-480026.5288	4.15
M0027	-764.98195	-480026.1749	4.50
M0028	-764.98195	-480026.1749	4.50
M0024	-764.98122	-480025.7168	4.96
M0044	-764.96852	-480017.7463	12.93

M0037	-764.96819	-480017.5399	13.14
M0032	-764.96623	-480016.3112	14.36
M0033	-764.96623	-480016.3112	14.36
M0036	-764.96610	-480016.2259	14.45
M0029	-764.96600	-480016.1669	14.51
M0030	-764.96600	-480016.1669	14.51
M0041	-764.96590	-480016.1004	14.57
M0034	-764.96441	-480015.1685	15.51
M0035	-764.96441	-480015.1685	15.51
M0040	-764.96353	-480014.6144	16.06
M0038	-764.96300	-480014.2800	16.40
M0039	-764.96212	-480013.7272	16.95
M0042	-764.96155	-480013.3695	17.31
**66_{RC}**	**Calculated energy (au)**	**Calculated energy (kcal/mol)**	**Relative energy (kcal/mol)**
M0002	-764.96136	-480013.2528	0.00
M0003	-764.96102	-480013.0394	0.21
M0001	-764.96068	-480012.8280	0.42
M0004	-764.95681	-480010.4002	2.85
M0006	-764.95522	-480009.4018	3.85
M0005	-764.95277	-480007.8638	5.39
**66_{RE}**	**Calculated energy (au)**	**Calculated energy (kcal/mol)**	**Relative energy (kcal/mol)**
M0001	-765.00261	-480039.1359	0.00
M0004	-765.00168	-480038.5523	0.58
M0002	-765.00120	-480038.2543	0.88
M0003	-765.00101	-480038.1338	1.00
M0005	-764.99920	-480036.9961	2.14
M0006	-764.99686	-480035.5309	3.60
M0009	-764.99526	-480034.5263	4.61
M0007	-764.99495	-480034.3280	4.81
M0010	-764.99337	-480033.3403	5.80
M0008	-764.99308	-480033.1546	5.98
M0011	-764.99111	-480031.9184	7.22
M0013	-764.98936	-480030.8259	8.31
M0012	-764.98871	-480030.4180	8.72

Table S30. Energies calculated at DFT/B3LYP/6-31G* in vacuum for the conformers found for **66_{RO}/66_{RC}/66_{RE}**.

**Isomer**	**ΔG° (au)**	**ΔG° (kcal/mol)**	**ΔG° (kcal/mol)**
**66_{RO}**	-764.761326	-479887.73	5.9
**66_{RC}**	-764.732155	-479869.43	24.2
**66_{RE}**	-764.770719	-479893.63	0.00

Table S31. Relative free energies (ΔG°) of the lowest energy geometries of **66_{RO}/66_{RC}/66_{RE}** at DFT/B3LYP/6-31G* in vacuum in kcal/mol.

XYZ Coordinates

66_{RO}

C	-1.63842000	-0.59877000	-0.01462000
C	-2.03948000	0.63381000	0.54314000
C	-1.03262000	1.69065000	0.91757000
C	-0.35254000	2.30621000	-0.30400000
H	0.52888900	2.86350000	0.03608000
H	-1.02020100	3.03703000	-0.77685000
C	0.05898000	1.27576000	-1.35499000
H	-0.81097000	0.97888000	-1.95431000
H	0.75999000	1.73573000	-2.06125000
N	0.64623000	0.07983000	-0.75511000
C	-0.20293900	-0.93415000	-0.28133000
O	0.13775100	-2.10689000	-0.14258000
C	2.03679000	0.10320100	-0.56510000
O	2.72110000	1.06670100	-0.92724000
C	2.67463100	-1.08548900	0.13959000
H	2.17174100	-1.25529900	1.09812000
H	2.54108100	-1.96522900	-0.49964000
C	4.17444100	-0.91211900	0.37610000
H	4.68733000	-0.64671800	-0.55522000
H	4.59100100	-1.86606900	0.71751000
N	4.46238000	0.09974100	1.39493000
H	5.47336000	0.17893200	1.50301000
H	-1.50630000	2.48915000	1.50200000
H	-0.28614000	1.24484000	1.58785000
H	4.16103000	1.00716100	1.02715000
C	-3.40553000	0.86832900	0.76316000
C	-4.35389000	-0.10660100	0.44913000
C	-3.95188900	-1.32963100	-0.08185000
C	-2.59864900	-1.57809100	-0.30886000
H	-3.74249000	1.81116900	1.19011000
H	-5.40923000	0.08529900	0.62926000
H	-4.69067900	-2.09268100	-0.31455000
H	-2.29278900	-2.53984000	-0.71712000

66_{RC}

H	0.81281700	3.21236000	-0.72995500
C	0.20650100	2.53885600	-0.11104000
H	-0.52065000	3.17204200	0.41229300
C	1.09908500	1.86932500	0.93162300
C	1.82666900	0.66026200	0.39170400
C	1.11265500	-0.51527300	0.03286200
C	-0.41230200	-0.60629200	0.22797900
C	-0.52431500	1.57455600	-1.04155800
O	-0.61451600	-0.63783400	1.62264000
H	-0.20493900	-1.47896000	1.88158000
N	-1.18251200	0.47857400	-0.34240700
N	-0.97989700	-1.80625800	-0.35126000
H	-1.26086800	2.14885000	-1.61575900
H	0.15304800	1.15033200	-1.79269600
C	-2.56213900	0.47871400	-0.23617300
O	-3.28059300	1.39813100	-0.62593300
C	-3.15377800	-0.74248800	0.41336300
H	-4.20481800	-0.83250000	0.11758400
H	-3.12666800	-0.60150200	1.49934800
C	-2.39387700	-1.99233500	-0.01044100
H	-2.46992500	-2.74059700	0.78739300
H	-2.89120700	-2.43965200	-0.88009000
H	1.81390200	2.61053500	1.31125000
H	0.50978200	1.57780400	1.80841500
H	-0.91228900	-1.66003500	-1.35768800
C	1.85764900	-1.61103900	-0.45273700
C	3.24418000	-1.53970100	-0.60573300
C	3.92368500	-0.37588000	-0.26696300
C	3.22087600	0.71781700	0.23135700
H	1.36562000	-2.54646400	-0.71297300
H	3.79283500	-2.39823800	-0.98505200
H	5.00282200	-0.32109200	-0.38460100
H	3.76894500	1.61939200	0.49749800

66$_{RE}$

H	-0.23836000	1.34546000	-1.23345900
C	-0.24351000	2.13214000	-0.46894900
H	0.07366900	3.05296000	-0.97422900
C	0.75565000	1.82703000	0.65866100
C	1.77412000	0.77943000	0.26969100
C	1.47667000	-0.59828000	0.25621100
C	-1.67000000	2.32277000	0.05402100
N	-2.22722000	1.07238000	0.52864100
N	-0.54539000	-1.71209000	-0.40220900
H	-1.68954100	3.03281000	0.88699100
H	-2.30739100	2.71382000	-0.74625900
C	-2.77489000	0.15283900	-0.34407900
O	-3.05642000	0.39436900	-1.51359900
C	-2.94000000	-1.22166100	0.26049100
H	-3.95579000	-1.57448100	0.04951100
H	-2.85886000	-1.16940100	1.35191100
C	-1.91166000	-2.19923000	-0.30234900
H	-1.89236000	-3.10488000	0.31376100
H	-2.20305000	-2.49710000	-1.31594900
H	-1.83084000	0.68854000	1.38500100
C	0.13270000	-1.10487000	0.62953100
O	-0.32358000	-0.92190000	1.75012100
H	1.27185900	2.75767000	0.93021100
H	0.25933000	1.51715000	1.58421100
H	-0.12327000	-1.69796000	-1.32400900
C	2.44050000	-1.53739000	-0.13756900
C	3.71034000	-1.11167000	-0.52242900
C	4.01672000	0.24764000	-0.51925900
C	3.05763000	1.18597000	-0.12889900
H	2.20708000	-2.59945000	-0.13318900
H	4.46175000	-1.83797000	-0.82209900
H	5.00733000	0.58074000	-0.82059900
H	3.32113000	2.24202000	-0.13942900

Gaussian Calculations

66_{RO}

M06-2X/6-31G* with Solvent Correction

SCF Done: E(RM062X) = -764.685625667

Zero-point correction= 0.281300
Thermal correction to Gibbs Free Energy= 0.238916

C	-1.64755400	-0.58316900	-0.01667900
C	-2.02500200	0.65945500	0.52775500
C	-0.99456300	1.70304500	0.89135900
C	-0.30356700	2.30742400	-0.34210200
H	0.59615800	2.84370200	-0.02500300
H	-0.96310800	3.02804200	-0.83696700
C	0.06137200	1.22133600	-1.35029400
H	-0.82308200	0.89081200	-1.89672600
H	0.79174000	1.58096900	-2.07288500
N	0.64270700	0.03462200	-0.69825500
C	-0.21479500	-0.97348900	-0.23741300
O	0.15913200	-2.11278100	-0.03316900
C	2.03865600	0.01040000	-0.56011900
O	2.68862300	0.94165500	-1.00417400
C	2.70274600	-1.14084300	0.16790300
H	2.18685700	-1.31501700	1.11693300
H	2.56849600	-2.05010800	-0.42435400
C	4.17862000	-0.85546900	0.41331900
H	4.68433800	-0.71609500	-0.55301700
H	4.61523200	-1.74084500	0.88515200
N	4.33933600	0.27689400	1.32870900
H	5.32216600	0.36108100	1.57970700
H	-1.46610300	2.49452200	1.47998800
H	-0.23109400	1.24653900	1.53171700
H	4.11293900	1.12452900	0.81179100
C	-3.38331000	0.89607000	0.74147700
C	-4.34377900	-0.06279700	0.42679100
C	-3.95792500	-1.29424000	-0.09707100
C	-2.60851500	-1.55419400	-0.30562300
H	-3.68949500	1.84652500	1.16993800
H	-5.39405900	0.15083500	0.59931900
H	-4.70134800	-2.04713200	-0.33697600
H	-2.28028100	-2.51112800	-0.69886100

66_{RC}

M06-2X/6-31G* with Solvent Correction

SCF Done: E(RM062X) = -764.675101677

Zero-point correction= 0.283360
Thermal correction to Gibbs Free Energy= 0.243889

H	0.94774000	3.20273400	-0.67327000
C	0.27928200	2.57767400	-0.07032400
H	-0.44587000	3.23981700	0.41401800
C	1.07757900	1.82473700	1.00789400
C	1.79822700	0.62828100	0.43293900
C	1.08883400	-0.51869900	0.01863600
C	-0.42084500	-0.61616200	0.26507900
C	-0.44369600	1.61292200	-1.00743600
O	-0.67157700	-0.60563500	1.66049500
H	-0.23818800	-1.39703800	2.02080300
N	-1.13916100	0.54290400	-0.29224800
N	-0.96532600	-1.85280700	-0.26152100
H	-1.19831400	2.13403300	-1.59510400
H	0.26951800	1.15710100	-1.70088500
C	-2.50929800	0.51423600	-0.31459400
O	-3.18095000	1.41290600	-0.80701300
C	-3.14802600	-0.69860600	0.33010400
H	-4.17411700	-0.74988600	-0.03734500
H	-3.18319300	-0.49857900	1.40427800
C	-2.38661400	-2.02064700	0.08007300
H	-2.46474900	-2.64767300	0.97156200
H	-2.85004700	-2.57144400	-0.74072700
H	1.79916900	2.49967100	1.47731600
H	0.38668700	1.48683500	1.78419000
H	-0.85612900	-1.83314100	-1.27356700
C	1.78068400	-1.59721300	-0.52925300
C	3.16439900	-1.54882300	-0.69752700
C	3.86684400	-0.41669000	-0.30541800
C	3.18034200	0.65925800	0.25573900
H	1.23646800	-2.49138500	-0.81053400
H	3.68478500	-2.39869400	-1.12750400
H	4.94440700	-0.36805700	-0.42849300
H	3.72718900	1.54524300	0.56831600

66$_{RE}$

M06-2X/6-31G* with Solvent Correction

SCF Done: E(RM062X) = -764.703220282

Zero-point correction= 0.282477
Thermal correction to Gibbs Free Energy= 0.241589

H	-0.24722300	1.23549700	-1.18387400
C	-0.21209200	2.08932500	-0.49651500
H	0.16232200	2.94191600	-1.07298500
C	0.74636400	1.78867700	0.67942300
C	1.79288700	0.76443600	0.31078300
C	1.46046000	-0.59867600	0.21248900
C	-1.64775000	2.35467000	-0.03641900
N	-2.25389200	1.14346600	0.49884500
N	-0.60676000	-1.67239500	-0.39944400
H	-1.68072700	3.12841600	0.73637800
H	-2.26294300	2.68274900	-0.87636000
C	-2.71349100	0.17662800	-0.33754100
O	-2.88247400	0.35409200	-1.53916600
C	-2.96214300	-1.17167800	0.31412400
H	-3.97280400	-1.50314100	0.06005900
H	-2.87715400	-1.10834700	1.39909900
C	-1.95903500	-2.19790600	-0.24114500
H	-1.94118200	-3.07629000	0.41150000
H	-2.27828600	-2.51738500	-1.23357100
H	-1.95785000	0.83458900	1.41721600
C	0.08479400	-1.07963000	0.59868700
O	-0.37420800	-0.94167300	1.73185100
H	1.23423900	2.71328900	1.00272300
H	0.17896000	1.41279300	1.53578100
H	-0.19100800	-1.69078600	-1.32055500
C	2.40503500	-1.53031600	-0.21872700
C	3.69939000	-1.12606500	-0.53911500
C	4.04029200	0.21886500	-0.43978800
C	3.08998600	1.15116900	-0.02505400
H	2.12870200	-2.57920000	-0.28711600
H	4.43283800	-1.85764300	-0.86215900
H	5.04493000	0.54673300	-0.68828400
H	3.35701800	2.20297400	0.03764600

Isomer $67_{RO}/67_{RC}/67_{RE}$

Spartan Calculations

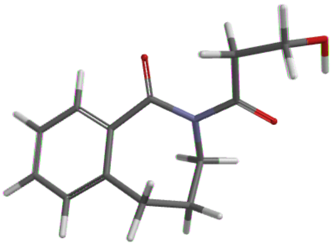

67_{RO}
$\Delta G° = 3.9$ kcal/mol

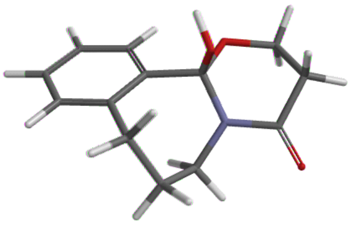

67_{RC}
$\Delta G° = 17.7$ kcal/mol

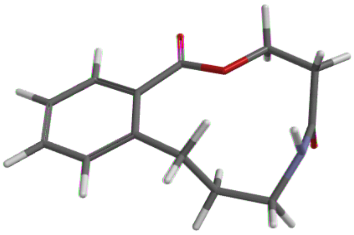

67_{RE}
$\Delta G° = 0.0$ kcal/mol

67_{RO}	Calculated energy (au)	Calculated energy (kcal/mol)	Relative energy (kcal/mol)
M0002	-784.85636	-492497.3628	0.00
M0001	-784.85577	-492496.9969	0.37
M0004	-784.85341	-492495.5123	1.85
M0006	-784.85224	-492494.7793	2.58
M0008	-784.85112	-492494.0772	3.29
M0012	-784.85065	-492493.7835	3.58
M0010	-784.85023	-492493.5187	3.84
M0014	-784.85001	-492493.3813	3.98
M0007	-784.84989	-492493.3053	4.06
M0009	-784.84988	-492493.3022	4.06
M0022	-784.84874	-492492.5812	4.78
M0003	-784.84798	-492492.1075	5.26
M0013	-784.84795	-492492.0867	5.28
M0023	-784.84794	-492492.0842	5.28
M0017	-784.84793	-492492.0736	5.29
M0019	-784.84734	-492491.7046	5.66
M0011	-784.84732	-492491.6958	5.67
M0024	-784.84691	-492491.4335	5.93
M0016	-784.84675	-492491.3369	6.03
M0005	-784.84647	-492491.1580	6.20
M0018	-784.84630	-492491.0501	6.31
M0020	-784.84620	-492490.9905	6.37
M0021	-784.84565	-492490.6441	6.72
M0015	-784.84442	-492489.8723	7.49
M0025	-784.84230	-492488.5414	8.82
M0029	-784.83986	-492487.0090	10.35
M0032	-784.83723	-492485.3593	12.00
M0030	-784.83602	-492484.6013	12.76
M0041	-784.83600	-492484.5919	12.77
M0026	-784.83538	-492484.2035	13.16

M0033	-784.83516	-492484.0604	13.30
M0037	-784.83102	-492481.4651	15.90
M0038	-784.83055	-492481.1676	16.20
M0034	-784.83047	-492481.1193	16.24
M0028	-784.82978	-492480.6888	16.67
M0039	-784.82968	-492480.6261	16.74
M0036	-784.82962	-492480.5853	16.78
M0035	-784.82949	-492480.5075	16.86
M0031	-784.82931	-492480.3901	16.97
M0040	-784.82877	-492480.0551	17.31
M0027	-784.82706	-492478.9820	18.38
M0042	-784.82511	-492477.7578	19.60
**67_{Rc}**	**Calculated energy (au)**	**Calculated energy (kcal/mol)**	**Relative energy (kcal/mol)**
M0001	-784.83633	-492484.7939	0.00
M0002	-784.82818	-492479.6817	5.11
**67_{RE}**	**Calculated energy (au)**	**Calculated energy (kcal/mol)**	**Relative energy (kcal/mol)**
M0008	-784.86642	-492503.6779	0.00
M0004	-784.86371	-492501.9761	1.70
M0011	-784.86351	-492501.8513	1.83
M0007	-784.86289	-492501.4654	2.21
M0001	-784.86279	-492501.3988	2.28
M0006	-784.86274	-492501.3675	2.31
M0003	-784.86226	-492501.0650	2.61
M0002	-784.86111	-492500.3446	3.33
M0014	-784.86070	-492500.0893	3.59
M0010	-784.85951	-492499.3400	4.34
M0012	-784.85823	-492498.5393	5.14
M0009	-784.85797	-492498.3762	5.30
M0005	-784.85756	-492498.1183	5.56
M0017	-784.85688	-492497.6891	5.99
M0016	-784.85616	-492497.2379	6.44
M0020	-784.85470	-492496.3268	7.35
M0013	-784.85445	-492496.1686	7.51
M0015	-784.85334	-492495.4734	8.20
M0019	-784.85295	-492495.2236	8.45
M0018	-784.84913	-492492.8278	10.85

Table S32. Energies calculated at DFT/B3LYP/6-31G* in vacuum for the conformers found for **67_{RO}/67_{RC}/67_{RE}**.

Isomer	ΔG° (au)	ΔG° (kcal/mol)	ΔG° (kcal/mol)
**67_{RO}**	-784.640525	-492361.93	3.9
**67_{RC}**	-784.618628	-492348.19	17.7
**67_{RE}**	-784.646771	-492365.85	0.0

Table S33. Relative free energies (ΔG°) of the lowest energy geometries of **67_{RO}/67_{RC}/67_{RE}** at DFT/B3LYP/6-31G* in vacuum in kcal/mol.

XYZ Coordinates

67$_{RO}$

C	1.64118100	-0.61815900	-0.02043000
C	2.17427100	0.58528200	0.48766000
C	1.27826100	1.68781100	0.98929000
C	0.48433100	2.35041100	-0.13498000
H	1.12268000	3.06027100	-0.67548000
H	-0.32547000	2.93851100	0.31389000
C	-0.09268900	1.35658100	-1.14233000
H	-0.83969900	1.86294100	-1.76473000
H	0.68954100	1.02358100	-1.83614000
N	-0.67477900	0.18271100	-0.49333000
C	0.16824100	-0.87206900	-0.10208000
O	-0.21612800	-2.01664900	0.12557000
C	-2.04345900	0.25662100	-0.19845000
O	-2.71261900	1.26158000	-0.46630000
C	-2.69776900	-0.93732000	0.47713000
H	-2.67743800	-1.77788000	-0.22442000
H	-2.14245900	-1.18192900	1.38781000
C	-4.14067900	-0.64398000	0.86829000
H	-4.22176900	0.25425000	1.48981000
H	-4.54716900	-1.48544000	1.43796000
H	0.59792100	1.27269100	1.74434000
H	1.86129100	2.45429100	1.51471000
C	3.56738100	0.74517200	0.53426000
C	4.41442100	-0.27547800	0.09880000
C	3.88346100	-1.47007800	-0.38196000
C	2.50115100	-1.64443800	-0.43721000
H	4.00597100	1.66432200	0.91841000
H	5.49295100	-0.14172800	0.14429000
H	4.54517200	-2.26842800	-0.70930000
H	2.09421200	-2.58406800	-0.80692000
O	-4.95986900	-0.47509000	-0.28374000
H	-4.65197900	0.34877000	-0.71309000

67$_{RC}$

H	0.73994100	3.21535800	-0.82325200
C	0.17707000	2.53235200	-0.17408900
H	-0.55426900	3.15149500	0.36015900
C	1.13225900	1.92158700	0.85124100
C	1.83141600	0.67717200	0.35486400
C	1.09654400	-0.50687800	0.07565100
C	-0.42067500	-0.57558900	0.32412000
C	-0.54891900	1.53247400	-1.07087600
O	-0.63221100	-0.42591300	1.72095800
H	0.16735000	-0.78146100	2.14006500
N	-1.19683600	0.44706700	-0.35053400
O	-0.90985000	-1.86769900	-0.02692400
H	-1.29475300	2.08161800	-1.65799500
H	0.13106400	1.09572400	-1.81238500
C	-2.57321500	0.42933200	-0.23334000
O	-3.31125300	1.34577900	-0.58682700
C	-3.12186800	-0.84236600	0.34551800
H	-4.17539700	-0.94507400	0.06690400
H	-3.05057400	-0.81278900	1.43606100
C	-2.30935900	-1.96025700	-0.27135400
H	-2.64827400	-2.92881400	0.11026000
H	-2.44873600	-1.98155500	-1.35924400
H	1.86710000	2.68374500	1.14103800
H	0.59703100	1.69104800	1.77895700
C	1.80195500	-1.62612500	-0.40825700
C	3.18238800	-1.57991700	-0.61112900
C	3.88929800	-0.41551800	-0.33289900
C	3.21933100	0.70765400	0.14654500
H	1.27922000	-2.55436300	-0.63388400
H	3.70452300	-2.45643100	-0.98699800
H	4.96407300	-0.38066700	-0.49226800
H	3.78871500	1.61185000	0.35267900

67_{RE}

H	-0.58096900	-0.88243100	1.28873900
C	-0.52066900	-1.79795100	0.68873900
H	-0.04705900	-2.55230100	1.32911900
C	0.34848100	-1.57456100	-0.55692100
C	1.61777100	-0.80046000	-0.28222100
C	1.67553000	0.59049000	-0.02611100
C	-1.93799900	-2.26216200	0.32556900
N	-2.61870900	-1.26347200	-0.47549100
O	-0.57363000	1.11041900	-0.62897100
H	-1.90668800	-3.18801200	-0.25751100
H	-2.51536900	-2.44485200	1.23806900
C	-3.05950000	-0.09691200	0.11877900
O	-3.36746000	-0.00759300	1.30221900
C	-2.99577000	1.10451800	-0.79493100
H	-3.90737000	1.69586700	-0.66367100
H	-2.94684000	0.78599800	-1.84164100
C	-1.75627100	1.91159800	-0.42352100
H	-1.68678100	2.79646800	-1.06535100
H	-1.82664100	2.23176800	0.62354900
H	-2.35697900	-1.21461200	-1.45128100
C	0.51436000	1.53411900	0.05994900
O	0.57795900	2.59082900	0.67314900
H	0.59597100	-2.55994100	-0.97543100
H	-0.18678900	-1.07695100	-1.37046100
C	2.92891000	1.18684000	0.22465900
C	4.10612000	0.43935100	0.21469900
C	4.05180100	-0.92362900	-0.03871100
C	2.82264100	-1.53626000	-0.28184100
H	2.99358900	2.25394000	0.43694900
H	5.05950000	0.92307100	0.41146900
H	4.96484100	-1.51393900	-0.04324100
H	2.81116100	-2.60888000	-0.47092100

Gaussian Calculations

67$_{RO}$

M06-2X/6-31G* with Solvent Correction

SCF Done: E(RM062X) = -784.548613593

Zero-point correction= 0.268977
Thermal correction to Gibbs Free Energy= 0.227092

C	1.65448200	-0.60183500	-0.04264100
C	2.14163300	0.60430000	0.49693700
C	1.20280600	1.68358100	0.98433300
C	0.43175600	2.36097900	-0.16037800
H	1.07842200	3.06507800	-0.69435400
H	-0.40670000	2.92886000	0.25438000
C	-0.07917700	1.32793500	-1.16041200
H	-0.85473000	1.74535900	-1.80017500
H	0.73307700	0.97320500	-1.79646800
N	-0.65566700	0.14836300	-0.49023400
C	0.19107300	-0.91761700	-0.14316000
O	-0.22249400	-2.04286400	0.05454800
C	-2.02799000	0.17777800	-0.22983400
O	-2.66989900	1.16209900	-0.56838900
C	-2.69263700	-0.97424900	0.49457100
H	-2.70667000	-1.84346400	-0.16943300
H	-2.09331000	-1.26625000	1.36178100
C	-4.11270000	-0.59885600	0.90928200
H	-4.08497500	0.27180100	1.57919000
H	-4.54908500	-1.43276500	1.46508300
H	0.48054600	1.24289400	1.68116800
H	1.76587200	2.43173100	1.54878300
C	3.52372000	0.76974900	0.58911500
C	4.40208200	-0.22360000	0.16201900
C	3.90897300	-1.41909800	-0.35474000
C	2.53504800	-1.60851400	-0.44401300
H	3.91507700	1.69121400	1.01155900
H	5.47310600	-0.06519300	0.24140600
H	4.58884000	-2.19894900	-0.68126800
H	2.12418500	-2.53643500	-0.82896700
O	-4.95410900	-0.36247700	-0.19982900
H	-4.57638900	0.41700000	-0.63817000

67$_{RC}$

M06-2X/6-31G* with Solvent Correction

SCF Done: E(RM062X) = -784.540986203

Zero-point correction= 0.270213
Thermal correction to Gibbs Free Energy= 0.230426

H	0.84800300	3.18981800	-0.81417100
C	0.21855900	2.55996000	-0.17533300
H	-0.52541800	3.20944200	0.29702400
C	1.07147400	1.88807700	0.91857700
C	1.79030700	0.66208700	0.40501300
C	1.08227800	-0.50888700	0.06596000
C	-0.41986400	-0.58853900	0.33428500
C	-0.48231900	1.54185300	-1.07293200
O	-0.66968900	-0.43357100	1.72128000
H	-0.24384100	-1.18801600	2.16020500
N	-1.15609900	0.47619800	-0.33216500
O	-0.87012800	-1.85173900	-0.06283400
H	-1.25062800	2.02590700	-1.67568700
H	0.23845400	1.07642100	-1.75076900
C	-2.52646700	0.46746100	-0.24890500
O	-3.22373400	1.38015300	-0.66230800
C	-3.08463500	-0.81922900	0.32272300
H	-4.12857400	-0.89735900	0.01423900
H	-3.03588100	-0.78899100	1.41273500
C	-2.28277800	-1.99737100	-0.22763500
H	-2.56905500	-2.92881200	0.26600000
H	-2.48017400	-2.10128600	-1.30030200
H	1.80065700	2.60430000	1.30831500
H	0.41873300	1.60004900	1.74526400
C	1.75666600	-1.60704500	-0.46136000
C	3.13720900	-1.56527800	-0.65302800
C	3.84503400	-0.41668300	-0.32172700
C	3.16836300	0.68625800	0.19705600
H	1.19557600	-2.49629500	-0.72166400
H	3.65120200	-2.42942000	-1.06173500
H	4.91991700	-0.37288600	-0.46812400
H	3.71918400	1.58900000	0.44856600

67RE

M06-2X/6-31G* with Solvent Correction

SCF Done: E(RM062X) = -784.560018415

Zero-point correction= 0.270146
Thermal correction to Gibbs Free Energy= 0.229010

H	-0.59717900	-0.96976200	1.22248200
C	-0.52407900	-1.86161900	0.59111900
H	-0.02208000	-2.63533400	1.18315400
C	0.30957700	-1.54921200	-0.66231500
C	1.58169300	-0.79715000	-0.34063400
C	1.66069800	0.58407900	-0.05496900
C	-1.94777500	-2.31377000	0.24740600
N	-2.66467700	-1.25969400	-0.46345500
O	-0.65196500	1.07766600	-0.47388200
H	-1.94222000	-3.20509700	-0.38646200
H	-2.50305500	-2.54671000	1.15793600
C	-2.95512000	-0.10301400	0.19279500
O	-3.05297700	-0.03719500	1.40992300
C	-3.02055000	1.14561700	-0.67151900
H	-3.90752800	1.72946700	-0.41432000
H	-3.05022000	0.90915100	-1.73884100
C	-1.77944800	1.96249500	-0.33215000
H	-1.65297600	2.82319500	-0.99270500
H	-1.82160400	2.30916200	0.70246000
H	-2.48102200	-1.18006600	-1.45429800
C	0.52028600	1.55993500	-0.03236100
O	0.63380300	2.70258700	0.35367900
H	0.58056500	-2.49053700	-1.15359900
H	-0.28822600	-0.97510700	-1.37108500
C	2.89759800	1.16046000	0.26389400
C	4.05962900	0.40463400	0.30829600
C	3.99134400	-0.95825400	0.03446300
C	2.76792700	-1.53732700	-0.28255100
H	2.92228200	2.22296500	0.47862600
H	5.00630000	0.87376700	0.55481900
H	4.88707500	-1.57099200	0.06518100
H	2.72105900	-2.60195400	-0.49576500

Isomer 69_{RO}/69_{RC}/69_{RE}

Spartan Calculations

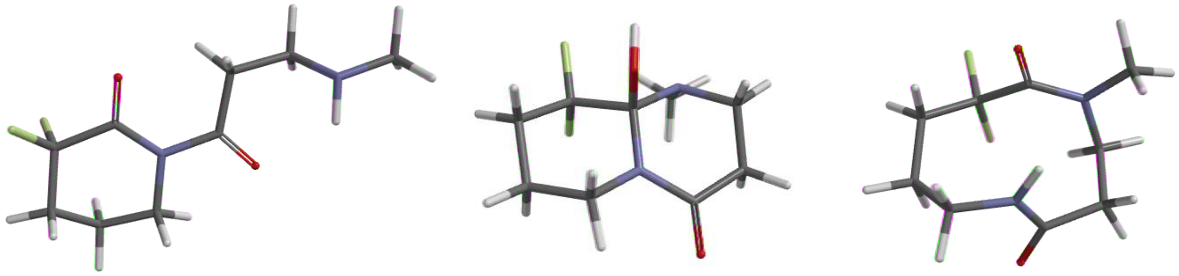

69_{RO}
ΔG ° = 3.9 kcal/mol

69_{RC}
ΔG ° = 11.2 kcal/mol

69_{RE}
ΔG ° = 0.0 kcal/mol

69_{RO}	Calculated energy (au)	Calculated energy (kcal/mol)	Relative energy (kcal/mol)
M0023	-811.02173	-508916.1343	0.00
M0017	-811.02080	-508915.5507	0.58
M0044	-811.01948	-508914.7237	1.41
M0048	-811.01915	-508914.5185	1.62
M0004	-811.01896	-508914.3980	1.74
M0001	-811.01833	-508914.0027	2.13
M0003	-811.01804	-508913.8195	2.31
M0007	-811.01785	-508913.6990	2.44
M0009	-811.01777	-508913.6526	2.48
M0002	-811.01753	-508913.5007	2.63
M0026	-811.01751	-508913.4875	2.65
M0038	-811.01727	-508913.3388	2.80
M0011	-811.01713	-508913.2510	2.88
M0005	-811.01688	-508913.0903	3.04
M0008	-811.01686	-508913.0771	3.06
M0010	-811.01640	-508912.7916	3.34
M0015	-811.01628	-508912.7144	3.42
M0018	-811.01625	-508912.6937	3.44
M0012	-811.01619	-508912.6611	3.47
M0014	-811.01613	-508912.6197	3.51
M0006	-811.01596	-508912.5130	3.62
M0013	-811.01593	-508912.4986	3.64
M0016	-811.01574	-508912.3775	3.76
M0034	-811.01571	-508912.3593	3.78
M0027	-811.01567	-508912.3342	3.80
M0053	-811.01559	-508912.2815	3.85
M0032	-811.01559	-508912.2796	3.85
M0030	-811.01550	-508912.2288	3.91
M0019	-811.01532	-508912.1127	4.02
M0050	-811.01502	-508911.9219	4.21

M0037	-811.01499	-508911.9056	4.23
M0046	-811.01492	-508911.8636	4.27
M0036	-811.01488	-508911.8397	4.29
M0043	-811.01481	-508911.7914	4.34
M0020	-811.01469	-508911.7186	4.42
M0047	-811.01453	-508911.6201	4.51
M0060	-811.01445	-508911.5693	4.57
M0045	-811.01445	-508911.5667	4.57
M0024	-811.01444	-508911.5592	4.58
M0041	-811.01429	-508911.4663	4.67
M0031	-811.01404	-508911.3076	4.83
M0035	-811.01403	-508911.3013	4.83
M0049	-811.01400	-508911.2863	4.85
M0033	-811.01397	-508911.2687	4.87
M0028	-811.01384	-508911.1865	4.95
M0025	-811.01381	-508911.1626	4.97
M0021	-811.01354	-508910.9970	5.14
M0058	-811.01349	-508910.9637	5.17
M0040	-811.01343	-508910.9267	5.21
M0022	-811.01305	-508910.6864	5.45
M0052	-811.01297	-508910.6406	5.49
M0055	-811.01285	-508910.5640	5.57
M0029	-811.01284	-508910.5590	5.58
M0051	-811.01275	-508910.4981	5.64
M0042	-811.01275	-508910.4981	5.64
M0039	-811.01235	-508910.2496	5.88
M0063	-811.01193	-508909.9886	6.15
M0054	-811.01110	-508909.4627	6.67
M0057	-811.01076	-508909.2513	6.88
M0056	-811.01042	-508909.0373	7.10
M0059	-811.00698	-508906.8806	9.25
M0061	-811.00386	-508904.9240	11.21
M0062	-811.00373	-508904.8387	11.30
**69_{RC}**	**Calculated energy (au)**	**Calculated energy (kcal/mol)**	**Relative energy (kcal/mol)**
M0001	-811.01709	-508913.2233	0.00
M0004	-811.01241	-508910.2854	2.94
M0002	-811.01142	-508909.6635	3.56
M0003	-811.00874	-508907.9856	5.24
M0006	-811.00649	-508906.5750	6.65
M0005	-811.00550	-508905.9494	7.27
M0008	-811.00405	-508905.0407	8.18
M0007	-811.00352	-508904.7094	8.51
M0009	-811.00297	-508904.3618	8.86
**69_{RE}**	**Calculated energy (au)**	**Calculated energy (kcal/mol)**	**Relative energy (kcal/mol)**
M0003	-811.03300	-508923.2100	0.00

M0002	-811.03082	-508921.8377	1.37
M0004	-811.03036	-508921.5490	1.66
M0001	-811.02756	-508919.7952	3.41
M0005	-811.02677	-508919.2988	3.91
M0006	-811.02639	-508919.0572	4.15

Table S34. Energies calculated at DFT/B3LYP/6-31G* in vacuum for the conformers found for **69_{RO}/69_{RC}/69_{RE}**.

Isomer	ΔG° (au)	ΔG° (kcal/mol)	ΔG° (kcal/mol)
**69_{RO}**	-810.838237	-508800.99	3.9
**69_{RC}**	-810.826607	-508793.70	11.2
**69_{RE}**	-810.844483	-508804.91	0.0

Table S35. Relative free energies (ΔG°) of the lowest energy geometries of **69_{RO}/69_{RC}/69_{RE}** at DFT/B3LYP/6-31G* in vacuum in kcal/mol.

XYZ Coordinates

69$_{RO}$

C	-1.14731100	-0.66573000	-0.31122000
C	-3.24468000	0.76634100	-0.33438000
C	-0.99469000	1.79958000	-0.17424000
C	-2.43443000	1.87576000	0.30537000
N	-0.37690000	0.47970000	-0.04700000
C	-2.64134000	-0.56897000	0.04867000
H	-3.26196000	0.87808100	-1.42495000
H	-0.94562900	2.07073000	-1.23694000
H	-2.46797000	1.78187000	1.39769000
H	-4.28267000	0.81834100	0.01241000
H	-0.40141900	2.54006000	0.37555000
H	-2.86136900	2.85207100	0.05115000
O	-0.66987100	-1.71307000	-0.75175000
C	1.02692000	0.48362900	-0.06444000
O	1.66246000	1.51466900	-0.30726000
C	1.74015900	-0.83320100	0.21349000
H	1.73067900	-1.44654100	-0.69378000
H	1.19880900	-1.36208100	1.00643000
C	3.18392900	-0.63302200	0.68347000
H	3.21380000	0.11981800	1.48085000
H	3.54093900	-1.58110200	1.10327000
N	4.06404000	-0.24170200	-0.42265000
H	3.70979000	0.63803800	-0.81331000
C	5.42875000	-0.03380300	0.04206000
H	5.48128000	0.76166700	0.79255000
H	6.06052000	0.25821700	-0.80247000
H	5.84713900	-0.95315300	0.46425000
F	-3.38229100	-1.54634900	-0.54947000
F	-2.81954100	-0.74234900	1.39328000

69_{RC}

C	0.13189000	-0.33531000	0.42142000
C	2.38103800	0.91403200	0.25348000
C	0.17998700	2.11281000	-0.01432000
C	1.64187700	2.04659200	-0.44386000
N	-0.53817200	0.84157900	-0.14187000
C	1.66354000	-0.39491800	-0.02613000
H	2.44646800	1.09675200	1.33197000
H	0.10737700	2.43059000	1.03362000
H	1.69344700	1.89997200	-1.53003000
H	3.40879800	0.85951300	-0.12251000
H	-0.32180400	2.88131000	-0.61411000
H	2.13400600	3.00120200	-0.22682000
O	0.11831900	-0.14204000	1.83552000
H	0.27079000	-1.04499000	2.16759000
N	-0.62722900	-1.56909100	0.17917000
C	-0.46849900	-2.12079100	-1.16484000
H	0.53410200	-2.53935000	-1.29621000
H	-0.65350900	-1.38425100	-1.95391000
H	-1.15185800	-2.96347100	-1.32394000
C	-2.03264900	-1.42066200	0.54958000
H	-2.56756800	-2.36749300	0.40363000
C	-1.92260200	0.95167800	-0.15999000
O	-2.50972300	2.03314700	-0.23696000
H	-2.11320000	-1.21763200	1.62532000
C	-2.71314000	-0.32318300	-0.22650000
H	-2.84365000	-0.59074300	-1.28072000
H	-3.70682100	-0.14137400	0.19823000
F	2.30973100	-1.40200800	0.62535000
F	1.78847000	-0.65774800	-1.35810000

69$_{RE}$

C	-0.74794100	-1.11892300	0.38892000
C	1.80964400	-1.41281900	0.19952000
C	2.51648100	-0.05774700	0.35665000
N	-1.83545500	-0.33452100	-0.02976000
C	0.46835300	-1.38465200	-0.54729000
H	1.70767200	-1.89739100	1.17729000
H	2.48657500	-2.05778800	-0.37657000
H	2.61149300	0.41417500	-0.62828000
O	-0.78476200	-1.65190300	1.50915000
H	3.54238400	-0.25648000	0.69283000
C	1.89435400	0.92163200	1.35301000
H	1.86907100	0.47916200	2.35429000
H	2.50097800	1.83280300	1.39950000
N	0.54402700	1.30135900	1.01621000
C	-1.74217400	0.76583000	-1.00397000
H	-1.13384800	0.47255100	-1.85777000
H	-2.75029600	0.92026300	-1.40821000
C	-1.24617600	2.08587900	-0.40480000
H	-1.79386100	2.34946900	0.50655000
H	-1.41216000	2.89810600	-1.12224000
C	0.23294500	2.03959400	-0.10387000
O	1.06861600	2.56390800	-0.83016000
H	-0.19687800	1.04517700	1.66127000
C	-2.99716500	-0.30081100	0.85119000
H	-3.89604900	-0.08424600	0.26697000
H	-2.84927900	0.47373100	1.60911000
H	-3.13758900	-1.26381400	1.35124000
F	0.31023400	-2.63047500	-1.10746000
F	0.58876900	-0.54925000	-1.61467000

Gaussian Calculations

69_{RO}

M06-2X/6-31G* with Solvent Correction

SCF Done: E(RM062X) = -810.719530330

Zero-point correction= 0.240549
Thermal correction to Gibbs Free Energy= 0.197869

C	-1.13509000	-0.68637500	-0.13592500
C	-3.21321900	0.81467900	-0.49992700
C	-0.96335100	1.82361700	-0.11123800
C	-2.43398500	1.87405300	0.26644000
N	-0.37799800	0.46431800	0.00828600
C	-2.67347000	-0.52319600	-0.08235000
H	-3.07336700	0.92664200	-1.58054100
H	-0.81747800	2.16460900	-1.14193300
H	-2.55408800	1.71222900	1.34224700
H	-4.28456500	0.83607800	-0.28330100
H	-0.37649800	2.47516300	0.53404200
H	-2.80951400	2.87418300	0.03889300
O	-0.68774300	-1.80592500	-0.24512100
C	1.03801100	0.44610200	0.08111500
O	1.62464700	1.50646500	-0.01397400
C	1.77202000	-0.85399200	0.32440300
H	1.67870900	-1.48335700	-0.56512300
H	1.27638300	-1.39899600	1.13253100
C	3.24350700	-0.61401300	0.64161900
H	3.33406700	0.08522200	1.49094600
H	3.67579600	-1.56762200	0.96623100
N	3.98196300	-0.16865500	-0.53323200
H	3.60992300	0.74235800	-0.79327600
C	5.40248200	-0.02353500	-0.24290600
H	5.61460500	0.62964000	0.62008900
H	5.91652900	0.38363900	-1.11726000
H	5.83084400	-1.00824800	-0.02764500
F	-3.20938500	-1.52459600	-0.81387300
F	-3.00903300	-0.76313100	1.22321800

69_{RC}

M06-2X/6-31G* with Solvent Correction

SCF Done: E(RM062X) = -810.731413285

Zero-point correction= 0.242644
Thermal correction to Gibbs Free Energy= 0.204247

C	0.12938000	-0.34430900	0.47783100
C	2.40566100	0.77267900	0.19345200
C	0.28951800	2.12736100	0.21688600
C	1.70201200	2.01809300	-0.34519200
N	-0.48298500	0.89431600	-0.01066600
C	1.55275700	-0.42937600	-0.13128300
H	2.53475000	0.82819700	1.27675000
H	0.32516800	2.32414800	1.29532900
H	1.65881100	1.96640800	-1.43743100
H	3.38389600	0.62141200	-0.27173100
H	-0.26033200	2.93527800	-0.26171500
H	2.26371000	2.91625300	-0.07557300
O	0.29217900	-0.17337100	1.87249300
H	0.59484200	-1.03319500	2.21070200
N	-0.64211900	-1.53824800	0.25468400
C	-0.55082200	-2.20192000	-1.04767700
H	0.44759900	-2.61356000	-1.18851000
H	-0.78472700	-1.55331900	-1.90169700
H	-1.26179200	-3.03131800	-1.03380900
C	-2.02677600	-1.31173400	0.67222800
H	-2.56861900	-2.25842100	0.60785900
C	-1.83617900	1.02562700	-0.23338800
O	-2.34488600	2.11039500	-0.47518500
H	-2.01975800	-0.99856800	1.71928300
C	-2.67432900	-0.23787300	-0.19336600
H	-2.80395200	-0.59032300	-1.22337000
H	-3.66191900	0.05033900	0.17337700
F	2.12953900	-1.57629500	0.33150600
F	1.48183500	-0.54184800	-1.49072700

69$_{RE}$

M06-2X/6-31G* with Solvent Correction

SCF Done: E(RM062X) = -810.737788131

Zero-point correction= 0.241724
Thermal correction to Gibbs Free Energy= 0.201495

C	-0.83954300	-1.04700800	0.34154100
C	1.69977500	-1.51657800	0.19696400
C	2.52798400	-0.23802100	0.35409300
N	-1.82725800	-0.18947200	-0.02719500
C	0.38457900	-1.35810600	-0.55259000
H	1.48234000	-1.98431800	1.16063900
H	2.27546400	-2.23353400	-0.39717900
H	2.63970800	0.24703000	-0.61875900
O	-0.91950400	-1.71775700	1.36425100
H	3.53066600	-0.52564300	0.68682800
C	1.98154700	0.77981900	1.35465800
H	1.94036100	0.34563900	2.35671600
H	2.64708400	1.64881200	1.38719900
N	0.63295800	1.21834400	1.02392500
C	-1.73048700	0.91524200	-0.98458200
H	-1.17677600	0.60840600	-1.86606600
H	-2.74949100	1.14420900	-1.30346400
C	-1.10477100	2.18282900	-0.36760000
H	-1.65129600	2.45607000	0.54220800
H	-1.18937600	3.00517800	-1.08061800
C	0.36989700	1.96935500	-0.07953400
O	1.25436700	2.36383000	-0.82776100
H	-0.10597800	1.04774000	1.69216600
C	-2.93485500	-0.10742300	0.92605800
H	-3.74106100	0.46923600	0.47273600
H	-2.63085900	0.37313100	1.86374600
H	-3.28826500	-1.11086700	1.16256000
F	0.06646500	-2.55690200	-1.13452400
F	0.55285900	-0.48114600	-1.58345300

Isomer $71_{RO}/71_{RC}/71_{RE}$

Spartan Calculations

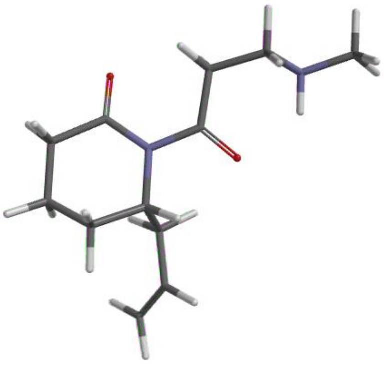

71_{RO}
ΔG ° = 3.5 kcal/mol

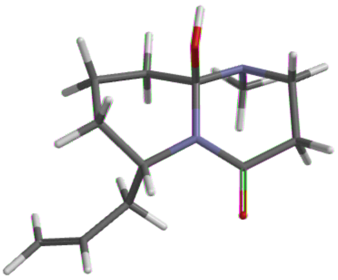

71_{RC}
ΔG ° = 19.6 kcal/mol

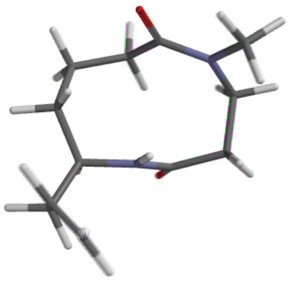

71_{RE}
ΔG ° = 0.0 kcal/mol

71_{RO}	Calculated energy (au)	Calculated energy (kcal/mol)	Relative energy (kcal/mol)
M0032	-729.26261	-457612.2890	0.00
M0033	-729.26261	-457612.2890	0.00
M0043	-729.26143	-457611.5448	0.74
M0045	-729.26082	-457611.1664	1.12
M0065	-729.26029	-457610.8313	1.46
M0062	-729.26022	-457610.7862	1.50
M0044	-729.25985	-457610.5527	1.74
M0001	-729.25969	-457610.4580	1.83
M0002	-729.25969	-457610.4580	1.83
M0003	-729.25856	-457609.7439	2.55
M0004	-729.25856	-457609.7439	2.55
M0055	-729.25855	-457609.7382	2.55
M0056	-729.25855	-457609.7382	2.55
M0052	-729.25830	-457609.5858	2.70
M0007	-729.25817	-457609.5004	2.79
M0008	-729.25817	-457609.5004	2.79
M0006	-729.25804	-457609.4226	2.87
M0078	-729.25803	-457609.4113	2.88
M0073	-729.25776	-457609.2438	3.05
M0066	-729.25748	-457609.0687	3.22
M0005	-729.25724	-457608.9175	3.37
M0079	-729.25724	-457608.9162	3.37
M0080	-729.25724	-457608.9162	3.37
M0013	-729.25695	-457608.7380	3.55
M0081	-729.25676	-457608.6144	3.67
M0019	-729.25657	-457608.4983	3.79
M0016	-729.25652	-457608.4638	3.83

M0017	-729.25644	-457608.4130	3.88
M0025	-729.25632	-457608.3389	3.95
M0085	-729.25609	-457608.1971	4.09
M0024	-729.25588	-457608.0666	4.22
M0011	-729.25583	-457608.0340	4.26
M0012	-729.25583	-457608.0340	4.26
M0014	-729.25558	-457607.8777	4.41
M0015	-729.25558	-457607.8777	4.41
M0074	-729.25548	-457607.8156	4.47
M0029	-729.25498	-457607.4987	4.79
M0030	-729.25498	-457607.4987	4.79
M0037	-729.25491	-457607.4579	4.83
M0031	-729.25486	-457607.4272	4.86
M0095	-729.25472	-457607.3349	4.95
M0096	-729.25472	-457607.3349	4.95
M0038	-729.25440	-457607.1366	5.15
M0042	-729.25439	-457607.1304	5.16
M0034	-729.25435	-457607.1065	5.18
M0036	-729.25422	-457607.0237	5.27
M0026	-729.25380	-457606.7564	5.53
M0027	-729.25380	-457606.7564	5.53
M0069	-729.25370	-457606.6986	5.59
M0057	-729.25322	-457606.3962	5.89
M0089	-729.25287	-457606.1778	6.11
M0067	-729.25253	-457605.9632	6.33
M0093	-729.25220	-457605.7574	6.53
M0077	-729.25218	-457605.7442	6.54
M0047	-729.25212	-457605.7066	6.58
M0063	-729.25185	-457605.5371	6.75
M0048	-729.25181	-457605.5076	6.78
M0022	-729.25173	-457605.4625	6.83
M0023	-729.25173	-457605.4625	6.83
M0068	-729.25168	-457605.4286	6.86
M0058	-729.25120	-457605.1293	7.16
M0009	-729.25104	-457605.0263	7.26
M0018	-729.25101	-457605.0107	7.28
M0098	-729.25081	-457604.8833	7.41
M0035	-729.25062	-457604.7659	7.52
M0060	-729.25052	-457604.7026	7.59
M0061	-729.25052	-457604.7026	7.59
M0071	-729.25052	-457604.7019	7.59
M0010	-729.25047	-457604.6712	7.62
M0039	-729.25029	-457604.5570	7.73
M0086	-729.24992	-457604.3248	7.96
M0099	-729.24979	-457604.2426	8.05
M0083	-729.24968	-457604.1711	8.12

M0020	-729.24945	-457604.0274	8.26
M0070	-729.24942	-457604.0136	8.28
M0051	-729.24907	-457603.7933	8.50
M0021	-729.24887	-457603.6659	8.62
M0076	-729.24783	-457603.0146	9.27
M0082	-729.24754	-457602.8301	9.46
M0028	-729.24728	-457602.6663	9.62
M0054	-729.24709	-457602.5465	9.74
M0049	-729.24701	-457602.4988	9.79
M0091	-729.24673	-457602.3224	9.97
M0053	-729.24618	-457601.9798	10.31
M0084	-729.24598	-457601.8531	10.44
M0040	-729.24584	-457601.7652	10.52
M0041	-729.24584	-457601.7652	10.52
M0072	-729.24546	-457601.5236	10.77
M0092	-729.24356	-457600.3333	11.96
M0088	-729.24335	-457600.2021	12.09
M0059	-729.24314	-457600.0685	12.22
M0075	-729.24275	-457599.8244	12.46
M0046	-729.24158	-457599.0921	13.20
M0094	-729.24099	-457598.7200	13.57
M0064	-729.23942	-457597.7335	14.56
M0087	-729.23417	-457594.4429	17.85
M0097	-729.23240	-457593.3335	18.96
M0100	-729.23134	-457592.6671	19.62
M0090	-729.23069	-457592.2592	20.03
**71_{RC}**	**Calculated energy (au)**	**Calculated energy (kcal/mol)**	**Relative energy (kcal/mol)**
M0005	-729.24300	-457599.9800	0.00
M0008	-729.24199	-457599.3500	0.63
M0014	-729.24191	-457599.2960	0.68
M0020	-729.24164	-457599.1260	0.85
M0006	-729.24133	-457598.9314	1.05
M0001	-729.24079	-457598.5945	1.39
M0011	-729.24055	-457598.4458	1.53
M0036	-729.24043	-457598.3692	1.61
M0002	-729.23958	-457597.8390	2.14
M0039	-729.23940	-457597.7254	2.25
M0025	-729.23934	-457597.6884	2.29
M0019	-729.23927	-457597.6407	2.34
M0012	-729.23919	-457597.5942	2.39
M0016	-729.23908	-457597.5240	2.46
M0066	-729.23902	-457597.4876	2.49
M0013	-729.23874	-457597.3075	2.67
M0017	-729.23864	-457597.2460	2.73
M0031	-729.23848	-457597.1449	2.84
M0004	-729.23828	-457597.0201	2.96

M0090	-729.23826	-457597.0050	2.97
M0040	-729.23804	-457596.8682	3.11
M0053	-729.23795	-457596.8105	3.17
M0048	-729.23765	-457596.6247	3.36
M0037	-729.23764	-457596.6191	3.36
M0034	-729.23758	-457596.5796	3.40
M0026	-729.23736	-457596.4403	3.54
M0047	-729.23715	-457596.3135	3.67
M0009	-729.23704	-457596.2432	3.74
M0003	-729.23703	-457596.2344	3.75
M0023	-729.23683	-457596.1083	3.87
M0054	-729.23682	-457596.1046	3.88
M0049	-729.23656	-457595.9414	4.04
M0041	-729.23639	-457595.8328	4.15
M0027	-729.23631	-457595.7870	4.19
M0044	-729.23615	-457595.6841	4.30
M0022	-729.23598	-457595.5756	4.40
M0007	-729.23593	-457595.5486	4.43
M0010	-729.23568	-457595.3917	4.59
M0050	-729.23561	-457595.3478	4.63
M0030	-729.23557	-457595.3177	4.66
M0038	-729.23540	-457595.2110	4.77
M0042	-729.23531	-457595.1570	4.82
M0028	-729.23530	-457595.1533	4.83
M0059	-729.23525	-457595.1200	4.86
M0078	-729.23505	-457594.9964	4.98
M0015	-729.23470	-457594.7736	5.21
M0072	-729.23465	-457594.7404	5.24
M0056	-729.23463	-457594.7310	5.25
M0055	-729.23429	-457594.5163	5.46
M0058	-729.23420	-457594.4611	5.52
M0018	-729.23411	-457594.4040	5.58
M0035	-729.23409	-457594.3934	5.59
M0021	-729.23402	-457594.3450	5.63
M0064	-729.23398	-457594.3231	5.66
M0067	-729.23394	-457594.2961	5.68
M0065	-729.23390	-457594.2723	5.71
M0086	-729.23389	-457594.2666	5.71
M0057	-729.23365	-457594.1141	5.87
M0083	-729.23364	-457594.1060	5.87
M0063	-729.23351	-457594.0269	5.95
M0060	-729.23350	-457594.0200	5.96
M0024	-729.23275	-457593.5513	6.43
M0075	-729.23273	-457593.5406	6.44
M0074	-729.23265	-457593.4860	6.49
M0033	-729.23254	-457593.4189	6.56

M0046	-729.23253	-457593.4113	6.57
M0032	-729.23236	-457593.3046	6.68
M0029	-729.23233	-457593.2896	6.69
M0088	-729.23226	-457593.2406	6.74
M0085	-729.23218	-457593.1955	6.78
M0080	-729.23210	-457593.1428	6.84
M0061	-729.23154	-457592.7895	7.19
M0071	-729.23135	-457592.6740	7.31
M0045	-729.23128	-457592.6282	7.35
M0068	-729.23117	-457592.5579	7.42
M0094	-729.23100	-457592.4500	7.53
M0051	-729.23033	-457592.0346	7.95
M0087	-729.23022	-457591.9618	8.02
M0052	-729.23003	-457591.8419	8.14
M0077	-729.22996	-457591.8024	8.18
M0098	-729.22960	-457591.5753	8.40
M0079	-729.22951	-457591.5144	8.47
M0081	-729.22938	-457591.4347	8.55
M0089	-729.22917	-457591.3023	8.68
M0084	-729.22916	-457591.2992	8.68
M0076	-729.22893	-457591.1523	8.83
M0043	-729.22877	-457591.0557	8.92
M0069	-729.22860	-457590.9478	9.03
M0062	-729.22855	-457590.9158	9.06
M0073	-729.22846	-457590.8568	9.12
M0070	-729.22836	-457590.7978	9.18
M0095	-729.22819	-457590.6867	9.29
M0091	-729.22792	-457590.5185	9.46
M0093	-729.22782	-457590.4558	9.52
M0100	-729.22708	-457589.9914	9.99
M0092	-729.22642	-457589.5767	10.40
M0096	-729.22558	-457589.0521	10.93
M0099	-729.22466	-457588.4716	11.51
M0097	-729.22308	-457587.4852	12.49
**71_{RE}**	**Calculated energy (au)**	**Calculated energy (kcal/mol)**	**Relative energy (kcal/mol)**
M0001	-729.27467	-457619.8561	0.00
M0006	-729.27452	-457619.7626	0.09
M0011	-729.27435	-457619.6527	0.20
M0003	-729.27377	-457619.2907	0.57
M0008	-729.27354	-457619.1432	0.71
M0015	-729.27335	-457619.0259	0.83
M0016	-729.27304	-457618.8351	1.02
M0013	-729.27262	-457618.5684	1.29
M0005	-729.27238	-457618.4172	1.44
M0010	-729.27226	-457618.3400	1.52
M0004	-729.27218	-457618.2930	1.56

M0007	-729.27178	-457618.0426	1.81
M0017	-729.27172	-457618.0068	1.85
M0022	-729.27167	-457617.9698	1.89
M0009	-729.27133	-457617.7571	2.10
M0012	-729.27102	-457617.5644	2.29
M0019	-729.27052	-457617.2494	2.61
M0014	-729.27001	-457616.9325	2.92
M0030	-729.26983	-457616.8158	3.04
M0024	-729.26965	-457616.7029	3.15
M0025	-729.26944	-457616.5705	3.29
M0023	-729.26939	-457616.5410	3.32
M0020	-729.26926	-457616.4632	3.39
M0018	-729.26923	-457616.4431	3.41
M0028	-729.26915	-457616.3923	3.46
M0042	-729.26914	-457616.3872	3.47
M0021	-729.26898	-457616.2837	3.57
M0032	-729.26892	-457616.2442	3.61
M0047	-729.26866	-457616.0860	3.77
M0029	-729.26864	-457616.0691	3.79
M0031	-729.26859	-457616.0415	3.81
M0052	-729.26842	-457615.9317	3.92
M0043	-729.26841	-457615.9298	3.93
M0037	-729.26837	-457615.9022	3.95
M0034	-729.26835	-457615.8871	3.97
M0026	-729.26822	-457615.8106	4.05
M0040	-729.26821	-457615.8030	4.05
M0041	-729.26788	-457615.5972	4.26
M0038	-729.26788	-457615.5966	4.26
M0033	-729.26775	-457615.5144	4.34
M0027	-729.26762	-457615.4284	4.43
M0045	-729.26757	-457615.3995	4.46
M0036	-729.26736	-457615.2653	4.59
M0049	-729.26699	-457615.0337	4.82
M0059	-729.26666	-457614.8310	5.03
M0035	-729.26652	-457614.7426	5.11
M0057	-729.26631	-457614.6095	5.25
M0046	-729.26604	-457614.4388	5.42
M0054	-729.26601	-457614.4200	5.44
M0051	-729.26601	-457614.4188	5.44
M0048	-729.26598	-457614.4037	5.45
M0056	-729.26595	-457614.3849	5.47
M0063	-729.26593	-457614.3723	5.48
M0060	-729.26583	-457614.3058	5.55
M0050	-729.26574	-457614.2506	5.61
M0039	-729.26571	-457614.2324	5.62
M0055	-729.26547	-457614.0837	5.77

M0070	-729.26545	-457614.0711	5.78
M0044	-729.26512	-457613.8597	6.00
M0058	-729.26510	-457613.8496	6.01
M0062	-729.26454	-457613.4989	6.36
M0069	-729.26356	-457612.8858	6.97
M0064	-729.26289	-457612.4622	7.39
M0061	-729.26202	-457611.9182	7.94
M0067	-729.26185	-457611.8077	8.05
M0068	-729.26180	-457611.7814	8.07
M0076	-729.26163	-457611.6697	8.19
M0075	-729.26112	-457611.3515	8.50
M0072	-729.26089	-457611.2104	8.65
M0074	-729.26075	-457611.1213	8.73
M0065	-729.26063	-457611.0434	8.81
M0073	-729.26034	-457610.8640	8.99
M0066	-729.26016	-457610.7479	9.11
M0071	-729.26016	-457610.7473	9.11

Table S36. Energies calculated at DFT/B3LYP/6-31G* in vacuum for the conformers found for $\mathbf{71_{RO}/71_{RC}/71_{RE}}$.

Isomer	ΔG° (au)	ΔG° (kcal/mol)	ΔG° (kcal/mol)
$\mathbf{71_{RO}}$	-729.002548	-457449.10	3.5
$\mathbf{71_{RC}}$	-728.976902	-457433.01	19.6
$\mathbf{71_{RE}}$	-729.008097	-457452.58	0.0

Table S37. Relative free energies (ΔG°) of the lowest energy geometries of $\mathbf{71_{RO}/71_{RC}/71_{RE}}$ at DFT/B3LYP/6-31G* in vacuum in kcal/mol.

XYZ Coordinates

71_{RO}

C	0.65357900	-1.82141000	-0.39920000
C	3.14133900	-1.39953100	0.24950000
C	1.38427000	0.39018000	0.59225000
C	2.58488000	-0.34064100	1.19870000
N	0.32314000	-0.57375000	0.16413000
C	2.03529900	-2.39615000	-0.10420000
H	3.98546900	-1.92812100	0.70553000
H	0.91800000	1.00425000	1.36432000
H	3.34690000	0.40508900	1.44563000
H	2.29229900	-3.02750000	-0.95860000
H	3.52769000	-0.92585100	-0.66012000
H	2.27626000	-0.81270000	2.14069000
H	1.87021900	-3.07925000	0.74197000
O	-0.14647100	-2.48016900	-1.04578000
C	-1.00576000	-0.07613900	0.24313000
O	-1.16958000	1.08089100	0.61461000
C	-2.18701000	-0.97541900	-0.06927000
H	-2.21007000	-1.15836900	-1.14812000
H	-2.02911100	-1.95783900	0.38648000
C	-3.52256000	-0.37945800	0.38658000
H	-3.48153000	-0.14962800	1.46816000
H	-4.28646000	-1.15660800	0.25776000
N	-3.92016000	0.77159200	-0.41792000
C	1.74646100	1.32967000	-0.58650000
H	2.23662000	0.77051000	-1.39211000
H	0.80322100	1.71587000	-0.99079000
C	2.60306100	2.49091900	-0.16050000
H	2.19010100	3.11413000	0.63447000
C	3.78753100	2.81449900	-0.68832000
H	4.34475200	3.68208900	-0.34726000
H	4.23423100	2.22877900	-1.48906000
H	-3.23729900	1.50983200	-0.26979000
C	-5.25556900	1.26305300	-0.09417000
H	-5.39557900	1.51822300	0.97309000
H	-6.00125000	0.50285300	-0.35520000
H	-5.47003900	2.15613300	-0.68921000

71RC

C	0.95293000	-0.87501100	-0.15932000
C	-1.26212900	-2.13720200	-0.45048000
C	-1.15550000	0.41181900	-0.62499000
C	-1.60982000	-0.90384200	-1.27624000
N	0.29038000	0.40472900	-0.30985000
C	-0.00434000	-1.94967100	0.40218000
H	-1.13156900	-2.99146100	-1.12593000
H	-1.32730000	1.19783800	-1.37320000
H	-2.68306000	-0.89499200	-1.49680000
H	-0.33185000	-1.68471100	1.41432000
H	-2.10093900	-2.39654200	0.20688000
H	-1.11597000	-0.99163100	-2.25336000
H	0.50533100	-2.91892100	0.47932000
O	1.32496000	-1.30391100	-1.45480000
H	2.04243000	-1.93266100	-1.27327000
N	2.17809000	-0.76207100	0.62183000
C	3.05603000	0.25976900	0.04583000
H	4.00003000	0.30688900	0.60373000
C	0.97517000	1.60073900	-0.41682000
O	0.42477000	2.65935900	-0.73206000
H	3.35525000	-0.03431100	-0.96849000
C	2.42082000	1.62850900	0.00316000
H	2.46209000	2.09931900	0.99247000
H	2.97315000	2.26674900	-0.69549000
C	-1.97382000	0.79595800	0.63303000
H	-1.83876000	0.04495800	1.41971000
H	-1.57818000	1.73118800	1.04983000
C	1.97211000	-0.54822100	2.05451000
H	1.54960000	-1.44049100	2.52750000
H	1.31955000	0.30333900	2.27469000
H	2.92970000	-0.37922100	2.56114000
C	-3.43796000	1.00981800	0.36027000
H	-3.69203000	1.80974800	-0.33347000
C	-4.42231000	0.29950800	0.92543000
H	-5.46174000	0.51078800	0.69227000
H	-4.21998000	-0.50109200	1.62983000

71$_{RE}$

C	1.78729000	1.09400000	0.28409000
C	0.09558900	2.25623900	-1.23347000
C	-1.15785100	1.77137900	-0.47689000
N	2.36145000	-0.14092900	0.58618000
C	1.34817900	1.37509000	-1.14832000
H	0.34055800	3.26146900	-0.86714000
H	1.17827000	0.47667000	-1.73586000
H	-0.16307100	2.37521900	-2.29333000
H	-1.97308100	2.45998800	-0.73442000
H	2.18215900	1.89796000	-1.63276000
O	1.65469900	1.96843000	1.14472000
H	-0.98644100	1.88958900	0.60017000
C	-1.60782000	0.32765800	-0.78356000
H	-1.58298000	0.18574800	-1.87117000
N	-0.68387900	-0.61727100	-0.16660000
C	2.35566100	-1.32504900	-0.29030000
H	2.48136100	-1.01439900	-1.33118000
H	3.24973100	-1.91064900	-0.04399000
C	1.11941100	-2.21537000	-0.12738000
H	0.86213200	-2.37491000	0.92501000
H	1.31343200	-3.20191000	-0.56430000
C	-0.05687900	-1.62131100	-0.86181000
O	-0.34236900	-1.94669100	-2.00870000
H	-0.47038900	-0.48432100	0.81610000
C	-3.05265000	0.04088800	-0.31777000
H	-3.74085000	0.71642700	-0.84087000
H	-3.32649900	-0.97662300	-0.62663000
C	-3.27857000	0.20138700	1.16520000
H	-3.19296000	1.20636800	1.57385000
C	-3.59823900	-0.80552300	1.98816000
H	-3.76282900	-0.62771300	3.04664000
H	-3.70790900	-1.82394300	1.62756000
C	2.77909000	-0.36888900	1.96444000
H	1.99447100	-0.92648000	2.48352000
H	3.70789100	-0.94673900	1.97098000
H	2.95609000	0.56874100	2.49826000

Gaussian Calculations

71$_{RO}$

M06-2X/6-31G* with Solvent Correction

SCF Done: E(RM062X) = -728.956758789

Zero-point correction= 0.318541
Thermal correction to Gibbs Free Energy= 0.272823

C	0.64994900	-1.77374000	-0.38169200
C	3.12575100	-1.29354400	0.22941500
C	1.33816500	0.42009500	0.63695000
C	2.54862500	-0.29732600	1.22360600
N	0.29795700	-0.54978200	0.20456700
C	2.04731300	-2.31239700	-0.12080900
H	3.99330600	-1.80888300	0.65034200
H	0.87236300	1.02311200	1.41812300
H	3.28931300	0.45657700	1.50497300
H	2.30542200	-2.92341600	-0.98812600
H	3.47230200	-0.77415500	-0.67043500
H	2.24475300	-0.81957700	2.13882300
H	1.91597600	-3.00374500	0.72310500
O	-0.14109300	-2.43957600	-1.02079700
C	-1.02510600	-0.07041000	0.25428100
O	-1.21555500	1.08242200	0.60544800
C	-2.18777200	-0.99184900	-0.05576100
H	-2.20050900	-1.19209600	-1.13042000
H	-2.02197700	-1.95800700	0.42803000
C	-3.51676300	-0.38187100	0.37542300
H	-3.47028300	-0.09662500	1.44101200
H	-4.28745900	-1.15675900	0.28651900
N	-3.90404600	0.72958900	-0.48441200
C	1.67963600	1.34830600	-0.54399100
H	2.05647200	0.75933200	-1.38841400
H	0.74305600	1.81757900	-0.86708000
C	2.67691700	2.40509700	-0.16893600
H	2.38761800	3.06938100	0.64644300
C	3.86160100	2.56384300	-0.75392000
H	4.55007600	3.34549000	-0.44825600
H	4.18008200	1.91787300	-1.56929000
H	-3.20437200	1.45829500	-0.35954600
C	-5.20551000	1.26392700	-0.10696100
H	-5.27608100	1.55236200	0.95542700
H	-5.97667800	0.50980400	-0.29777900
H	-5.43440300	2.14126500	-0.71738800

71_{RC}

M06-2X/6-31G* with Solvent Correction

SCF Done: E(RM062X) = -728.955626824

Zero-point correction= 0.320728
Thermal correction to Gibbs Free Energy= 0.279233

C	0.97760500	-0.88181100	-0.16115400
C	-1.23178900	-2.12788800	-0.30704400
C	-1.16879000	0.39496500	-0.64152000
C	-1.57507500	-0.95102800	-1.23474800
N	0.27821300	0.40072900	-0.35056600
C	0.02671400	-1.85699900	0.53676400
H	-1.09112100	-3.02763600	-0.91167100
H	-1.33164800	1.17900400	-1.38731900
H	-2.64923800	-0.92816400	-1.44027800
H	-0.25335300	-1.43943000	1.50683700
H	-2.06756900	-2.33135700	0.37052500
H	-1.05619700	-1.07151400	-2.18812500
H	0.58150000	-2.78078200	0.73356300
O	1.28548800	-1.35693000	-1.46170600
H	1.83596900	-2.14728200	-1.33279600
N	2.22417800	-0.73579000	0.56413800
C	3.06195000	0.27659800	-0.08506200
H	4.03151200	0.30070700	0.41957600
C	0.90989700	1.61169700	-0.36708400
O	0.29549500	2.65409800	-0.57369800
H	3.22878100	-0.03963900	-1.11608500
C	2.39602800	1.64850400	-0.05654900
H	2.48625300	2.11123500	0.93364200
H	2.85711300	2.34090300	-0.76557000
C	-1.97434800	0.77803500	0.61532500
H	-1.85792400	0.01309600	1.39178600
H	-1.54803900	1.71233900	0.99991100
C	2.06925200	-0.48923200	1.99679400
H	1.72693900	-1.39346300	2.50349400
H	1.36675700	0.32695800	2.22933400
H	3.04788300	-0.22731900	2.40629600
C	-3.43132400	0.96997900	0.31078600
H	-3.67029700	1.75339500	-0.40978900
C	-4.41584300	0.24948100	0.84274900
H	-5.45647600	0.42397900	0.58724300
H	-4.21110400	-0.54077000	1.56211300

71$_{\mathbf{RE}}$

M06-2X/6-31G* with Solvent Correction

SCF Done: E(RM062X) = -728.975553569

Zero-point correction= 0.320049
Thermal correction to Gibbs Free Energy= 0.276046

C	1.69511500	1.11176500	0.31828400
C	0.00671800	2.21451000	-1.19650800
C	-1.20493800	1.68259500	-0.42175000
N	2.20820300	-0.10600000	0.67100300
C	1.28189800	1.36027000	-1.12696500
H	0.23749400	3.21166700	-0.81122400
H	1.15630400	0.44950800	-1.71037300
H	-0.26088800	2.33048000	-2.25316700
H	-2.04421700	2.36850400	-0.59101200
H	2.10216600	1.91931000	-1.59483700
O	1.57027300	2.01692700	1.14043700
H	-0.98625800	1.71826500	0.65304300
C	-1.66159700	0.26322500	-0.78856500
H	-1.62334400	0.12581800	-1.87334300
N	-0.73949300	-0.72132100	-0.23551200
C	2.38722200	-1.24575300	-0.21849400
H	2.60937800	-0.90937900	-1.23308500
H	3.26655500	-1.79405300	0.13176800
C	1.17507200	-2.20131300	-0.23930000
H	0.89127600	-2.46709600	0.78413900
H	1.44580700	-3.11237000	-0.77698500
C	0.04529000	-1.52551200	-0.98885400
O	-0.04458800	-1.60374000	-2.21153600
H	-0.61419700	-0.72064600	0.77011700
C	-3.09393700	-0.03611400	-0.30360900
H	-3.78063100	0.65308000	-0.80895300
H	-3.35292100	-1.05494400	-0.61073500
C	-3.22745300	0.09599900	1.18850000
H	-3.26163100	1.10802600	1.59115700
C	-3.26165900	-0.93909900	2.02580700
H	-3.33118400	-0.80450600	3.10089700
H	-3.23306200	-1.96232400	1.65702300
C	2.57393700	-0.34181900	2.06053200
H	2.03000400	-1.20649800	2.45663100
H	3.64775100	-0.54012800	2.14160400
H	2.32114900	0.54051500	2.64321700

Isomer 72_{RO}/72_{RC}/72_{RE}

Spartan Calculations

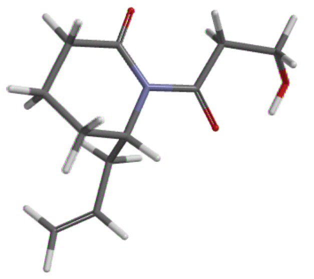

72_{RO}
ΔG ° = 4.8 kcal/mol

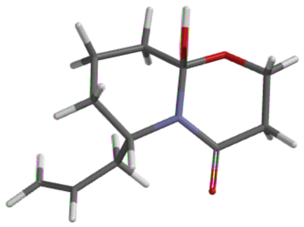

72_{RC}
ΔG ° = 14.5 kcal/mol

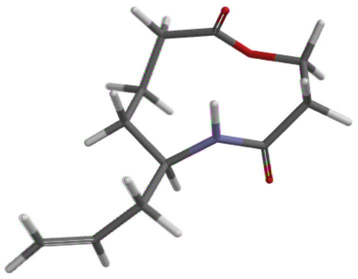

72_{RE}
ΔG ° = 0.0 kcal/mol

72_{RO}	Calculated energy (au)	Calculated energy (kcal/mol)	Relative energy (kcal/mol)
M0001	-709.82087	-445412.5959	0.00
M0002	-709.82026	-445412.2100	0.39
M0004	-709.81968	-445411.8473	0.75
M0007	-709.81916	-445411.5204	1.08
M0006	-709.81913	-445411.5034	1.09
M0005	-709.81894	-445411.3842	1.21
M0009	-709.81800	-445410.7956	1.80
M0008	-709.81745	-445410.4518	2.14
M0018	-709.81730	-445410.3564	2.24
M0016	-709.81693	-445410.1255	2.47
M0015	-709.81674	-445410.0031	2.59
M0023	-709.81671	-445409.9868	2.61
M0003	-709.81630	-445409.7283	2.87
M0017	-709.81624	-445409.6912	2.90
M0022	-709.81564	-445409.3160	3.28
M0013	-709.81487	-445408.8278	3.77
M0035	-709.81379	-445408.1532	4.44
M0026	-709.81375	-445408.1281	4.47
M0031	-709.81303	-445407.6776	4.92
M0036	-709.81295	-445407.6249	4.97
M0019	-709.81276	-445407.5050	5.09
M0040	-709.81275	-445407.4987	5.10
M0044	-709.81228	-445407.2070	5.39
M0028	-709.81223	-445407.1731	5.42
M0051	-709.81200	-445407.0319	5.56
M0062	-709.81191	-445406.9748	5.62
M0010	-709.81179	-445406.8982	5.70

M0033	-709.81130	-445406.5914	6.00
M0038	-709.81117	-445406.5104	6.09
M0034	-709.81117	-445406.5073	6.09
M0047	-709.81095	-445406.3705	6.23
M0032	-709.81087	-445406.3178	6.28
M0074	-709.81075	-445406.2425	6.35
M0055	-709.81038	-445406.0122	6.58
M0067	-709.81038	-445406.0103	6.59
M0065	-709.81036	-445406.0003	6.60
M0057	-709.81031	-445405.9670	6.63
M0049	-709.81029	-445405.9589	6.64
M0075	-709.81016	-445405.8779	6.72
M0056	-709.81003	-445405.7945	6.80
M0048	-709.80999	-445405.7706	6.83
M0054	-709.80989	-445405.7053	6.89
M0042	-709.80981	-445405.6526	6.94
M0061	-709.80973	-445405.6056	6.99
M0063	-709.80948	-445405.4474	7.15
M0060	-709.80942	-445405.4079	7.19
M0052	-709.80941	-445405.4029	7.19
M0027	-709.80935	-445405.3684	7.23
M0024	-709.80934	-445405.3634	7.23
M0020	-709.80928	-445405.3201	7.28
M0081	-709.80918	-445405.2617	7.33
M0021	-709.80914	-445405.2360	7.36
M0066	-709.80899	-445405.1387	7.46
M0071	-709.80888	-445405.0691	7.53
M0068	-709.80840	-445404.7691	7.83
M0045	-709.80838	-445404.7603	7.84
M0014	-709.80796	-445404.4930	8.10
M0080	-709.80778	-445404.3832	8.21
M0073	-709.80760	-445404.2690	8.33
M0039	-709.80743	-445404.1617	8.43
M0037	-709.80735	-445404.1102	8.49
M0070	-709.80724	-445404.0450	8.55
M0030	-709.80719	-445404.0105	8.59
M0025	-709.80690	-445403.8323	8.76
M0079	-709.80688	-445403.8185	8.78
M0089	-709.80687	-445403.8084	8.79
M0083	-709.80680	-445403.7689	8.83
M0012	-709.80647	-445403.5574	9.04
M0041	-709.80620	-445403.3899	9.21
M0050	-709.80617	-445403.3729	9.22
M0011	-709.80613	-445403.3466	9.25
M0046	-709.80610	-445403.3284	9.27
M0092	-709.80601	-445403.2706	9.33

M0072	-709.80554	-445402.9782	9.62
M0059	-709.80536	-445402.8603	9.74
M0088	-709.80517	-445402.7467	9.85
M0077	-709.80516	-445402.7366	9.86
M0064	-709.80479	-445402.5070	10.09
M0043	-709.80474	-445402.4725	10.12
M0069	-709.80468	-445402.4367	10.16
M0091	-709.80452	-445402.3388	10.26
M0078	-709.80435	-445402.2271	10.37
M0084	-709.80433	-445402.2171	10.38
M0058	-709.80370	-445401.8230	10.77
M0085	-709.80304	-445401.4095	11.19
M0029	-709.80295	-445401.3536	11.24
M0090	-709.80187	-445400.6728	11.92
M0053	-709.80146	-445400.4149	12.18
M0086	-709.80113	-445400.2116	12.38
M0076	-709.80092	-445400.0748	12.52
M0093	-709.80026	-445399.6625	12.93
M0100	-709.80020	-445399.6274	12.97
M0082	-709.79994	-445399.4649	13.13
M0094	-709.79630	-445397.1808	15.42
M0087	-709.79459	-445396.1052	16.49
M0095	-709.79240	-445394.7285	17.87
M0097	-709.79087	-445393.7728	18.82
M0098	-709.78902	-445392.6082	19.99
M0099	-709.78875	-445392.4425	20.15
M0096	-709.78861	-445392.3553	20.24
**72_{RO}**	**Calculated energy (au)**	**Calculated energy (kcal/mol)**	**Relative energy (kcal/mol)**
M0004	-709.81168	-445406.8261	0.00
M0014	-709.81016	-445405.8729	0.95
M0017	-709.80935	-445405.3671	1.46
M0002	-709.80862	-445404.9091	1.92
M0001	-709.80842	-445404.7823	2.04
M0008	-709.80804	-445404.5451	2.28
M0025	-709.80803	-445404.5388	2.29
M0024	-709.80776	-445404.3700	2.46
M0007	-709.80747	-445404.1893	2.64
M0016	-709.80721	-445404.0211	2.80
M0023	-709.80718	-445404.0048	2.82
M0009	-709.80707	-445403.9377	2.89
M0029	-709.80702	-445403.9057	2.92
M0030	-709.80641	-445403.5216	3.30
M0028	-709.80623	-445403.4087	3.42
M0015	-709.80621	-445403.3980	3.43
M0011	-709.80601	-445403.2732	3.55
M0031	-709.80581	-445403.1445	3.68

M0012	-709.80571	-445403.0843	3.74
M0010	-709.80541	-445402.8916	3.93
M0041	-709.80536	-445402.8640	3.96
M0046	-709.80513	-445402.7159	4.11
M0022	-709.80498	-445402.6231	4.20
M0045	-709.80495	-445402.6086	4.22
M0018	-709.80482	-445402.5246	4.30
M0032	-709.80471	-445402.4568	4.37
M0003	-709.80466	-445402.4248	4.40
M0020	-709.80455	-445402.3576	4.47
M0044	-709.80439	-445402.2554	4.57
M0036	-709.80429	-445402.1888	4.64
M0006	-709.80427	-445402.1807	4.65
M0005	-709.80399	-445402.0031	4.82
M0047	-709.80399	-445402.0025	4.82
M0021	-709.80360	-445401.7571	5.07
M0013	-709.80343	-445401.6492	5.18
M0038	-709.80337	-445401.6140	5.21
M0026	-709.80321	-445401.5111	5.31
M0037	-709.80311	-445401.4515	5.37
M0027	-709.80309	-445401.4358	5.39
M0039	-709.80233	-445400.9608	5.87
M0034	-709.80229	-445400.9338	5.89
M0035	-709.80203	-445400.7713	6.05
M0040	-709.80149	-445400.4325	6.39
M0019	-709.80112	-445400.1997	6.63
M0043	-709.80044	-445399.7730	7.05
M0042	-709.79903	-445398.8938	7.93
M0033	-709.79840	-445398.4929	8.33
**72_{RC}**	**Calculated energy (au)**	**Calculated energy (kcal/mol)**	**Relative energy (kcal/mol)**
M0013	-709.83386	-445420.7484	0.00
M0004	-709.83367	-445420.6279	0.12
M0009	-709.83361	-445420.5884	0.16
M0003	-709.83345	-445420.4880	0.26
M0011	-709.83316	-445420.3048	0.44
M0027	-709.83278	-445420.0720	0.68
M0007	-709.83256	-445419.9295	0.82
M0006	-709.83238	-445419.8185	0.93
M0022	-709.83215	-445419.6716	1.08
M0005	-709.83214	-445419.6704	1.08
M0019	-709.83181	-445419.4601	1.29
M0015	-709.83171	-445419.3949	1.35
M0002	-709.83103	-445418.9732	1.78
M0001	-709.83102	-445418.9669	1.78
M0032	-709.83095	-445418.9199	1.83
M0018	-709.83080	-445418.8295	1.92

M0012	-709.83058	-445418.6858	2.06
M0016	-709.83056	-445418.6777	2.07
M0010	-709.83022	-445418.4643	2.28
M0024	-709.83007	-445418.3714	2.38
M0028	-709.83003	-445418.3413	2.41
M0017	-709.82999	-445418.3187	2.43
M0030	-709.82972	-445418.1487	2.60
M0025	-709.82948	-445417.9981	2.75
M0008	-709.82906	-445417.7326	3.02
M0026	-709.82901	-445417.7006	3.05
M0041	-709.82899	-445417.6900	3.06
M0020	-709.82898	-445417.6824	3.07
M0039	-709.82895	-445417.6661	3.08
M0042	-709.82870	-445417.5111	3.24
M0014	-709.82826	-445417.2313	3.52
M0049	-709.82814	-445417.1553	3.59
M0035	-709.82808	-445417.1202	3.63
M0038	-709.82807	-445417.1139	3.63
M0043	-709.82806	-445417.1045	3.64
M0031	-709.82797	-445417.0531	3.70
M0044	-709.82782	-445416.9558	3.79
M0033	-709.82774	-445416.9081	3.84
M0023	-709.82757	-445416.7989	3.95
M0034	-709.82750	-445416.7550	3.99
M0029	-709.82742	-445416.7042	4.04
M0053	-709.82708	-445416.4921	4.26
M0045	-709.82703	-445416.4620	4.29
M0040	-709.82698	-445416.4306	4.32
M0021	-709.82676	-445416.2919	4.46
M0036	-709.82627	-445415.9813	4.77
M0047	-709.82626	-445415.9807	4.77
M0054	-709.82621	-445415.9487	4.80
M0055	-709.82613	-445415.8959	4.85
M0037	-709.82612	-445415.8878	4.86
M0059	-709.82584	-445415.7127	5.04
M0048	-709.82539	-445415.4335	5.31
M0046	-709.82520	-445415.3117	5.44
M0068	-709.82519	-445415.3061	5.44
M0057	-709.82487	-445415.1040	5.64
M0061	-709.82475	-445415.0300	5.72
M0052	-709.82472	-445415.0087	5.74
M0050	-709.82465	-445414.9672	5.78
M0069	-709.82452	-445414.8876	5.86
M0051	-709.82437	-445414.7890	5.96
M0058	-709.82408	-445414.6089	6.14
M0056	-709.82379	-445414.4289	6.32

M0067	-709.82366	-445414.3454	6.40
M0060	-709.82308	-445413.9808	6.77
M0079	-709.82288	-445413.8578	6.89
M0065	-709.82288	-445413.8541	6.89
M0091	-709.82286	-445413.8440	6.90
M0080	-709.82285	-445413.8352	6.91
M0070	-709.82282	-445413.8170	6.93
M0084	-709.82271	-445413.7493	7.00
M0074	-709.82267	-445413.7273	7.02
M0064	-709.82249	-445413.6125	7.14
M0083	-709.82222	-445413.4443	7.30
M0073	-709.82212	-445413.3790	7.37
M0062	-709.82200	-445413.3056	7.44
M0081	-709.82194	-445413.2667	7.48
M0092	-709.82180	-445413.1795	7.57
M0086	-709.82170	-445413.1193	7.63
M0088	-709.82162	-445413.0666	7.68
M0085	-709.82161	-445413.0596	7.69
M0072	-709.82141	-445412.9323	7.82
M0066	-709.82140	-445412.9291	7.82
M0076	-709.82118	-445412.7930	7.96
M0063	-709.82111	-445412.7490	8.00
M0090	-709.82067	-445412.4679	8.28
M0087	-709.82059	-445412.4215	8.33
M0094	-709.82049	-445412.3594	8.39
M0075	-709.82045	-445412.3324	8.42
M0077	-709.82014	-445412.1347	8.61
M0071	-709.81993	-445412.0086	8.74
M0082	-709.81984	-445411.9471	8.80
M0078	-709.81971	-445411.8693	8.88
M0089	-709.81920	-445411.5480	9.20
M0096	-709.81905	-445411.4507	9.30
M0099	-709.81887	-445411.3422	9.41
M0098	-709.81827	-445410.9669	9.78
M0097	-709.81794	-445410.7580	9.99
M0095	-709.81779	-445410.6657	10.08
M0093	-709.81719	-445410.2874	10.46
M0100	-709.81127	-445406.5700	14.18

Table S38. Energies calculated at DFT/B3LYP/6-31G* in vacuum for the conformers found for **72_{RO}/72_{RC}/72_{RE}**.

Isomer	ΔG° (au)	ΔG° (kcal/mol)	ΔG° (kcal/mol)
**72_{RO}**	-709.598571	-445273.10	4.8
**72_{RC}**	-709.583065	-445263.37	14.5
**72_{RE}**	-709.606218	-445277.90	0.0

Table S39. Relative free energies (ΔG°) of the lowest energy geometries of **72_{RO}/72_{RC}/72_{RE}** at DFT/B3LYP/6-31G* in vacuum in kcal/mol.

XYZ Coordinates

72$_{RO}$

C	0.00342100	1.62888900	-0.43511000
C	-2.42480900	1.69670000	0.36063000
C	-1.01623900	-0.36084000	0.63154000
C	-2.06639900	0.54780000	1.28259000
N	0.17505100	0.40610900	0.21517000
C	-1.17421900	2.47664000	0.01369000
H	-3.14941900	2.35748000	0.84967000
H	-0.70257000	-1.07458000	1.40591000
H	-2.96652900	-0.01222000	1.55660000
H	-1.40913900	3.19163000	-0.78320000
H	-2.90381900	1.32512000	-0.55166000
H	-1.65811900	0.95413000	2.21752000
H	-0.83830900	3.05453000	0.88331000
O	0.78179100	2.08148900	-1.27343000
C	1.38835100	-0.29723100	0.21716000
O	1.44287000	-1.50869100	0.45755000
C	2.65975100	0.47305900	-0.10477000
H	2.72480100	0.58368900	-1.19200000
H	2.62012100	1.45491900	0.37752000
C	3.89354100	-0.26415200	0.40127000
H	3.79998000	-0.53938200	1.45720000
H	4.77590100	0.37432800	0.29435000
C	-1.56844000	-1.18331000	-0.56489000
H	-1.85646000	-0.51487000	-1.38549000
H	-0.76858000	-1.81287000	-0.97488000
C	-2.70764000	-2.09448000	-0.19827000
H	-2.49463000	-2.87947000	0.52582000
C	-3.93654000	-2.01122000	-0.72330000
H	-4.71431000	-2.70967900	-0.42793000
H	-4.19666000	-1.25826900	-1.46102000
O	4.13191000	-1.44585200	-0.35569000
H	3.35515000	-2.02395200	-0.20854000

72$_{RC}$

C	1.16420000	0.89291000	-0.10075000
C	-1.00385100	2.24823000	-0.00550000
C	-0.99874000	-0.24417000	0.61137000
C	-1.33141000	1.18205000	1.06160000
N	0.42892000	-0.33365000	0.20435000
C	0.25964900	1.89269000	-0.82332000
H	-0.87544100	3.21542000	0.49134000
H	-1.11313000	-0.92348000	1.46136000
H	-2.39437000	1.22134900	1.31851000
H	-0.00239100	1.44177000	-1.78534000
H	-1.84567100	2.37039000	-0.69504000
H	-0.76612100	1.39313000	1.97297000
H	0.85957900	2.78067000	-1.05135000
O	1.62141900	1.40673000	1.15349000
H	2.07449900	2.24525100	0.97958000
O	2.26091000	0.64755100	-0.94329000
C	3.16372000	-0.33978900	-0.42018000
H	3.99691000	-0.37829900	-1.12497000
C	1.02916000	-1.57028000	0.23806000
O	0.42576000	-2.57343000	0.61478000
H	3.53988000	-0.01199900	0.55504000
C	2.44424000	-1.67587900	-0.31321000
H	2.34218000	-2.13564900	-1.30402000
H	2.99328000	-2.38359900	0.31445000
C	-1.91670000	-0.76539000	-0.52504000
H	-1.88795000	-0.08497000	-1.38422000
H	-1.50983000	-1.72892000	-0.85356000
C	-3.33786000	-0.96972100	-0.07755000
H	-3.47454000	-1.65224100	0.76295000
C	-4.41503000	-0.40703100	-0.63462000
H	-5.41819000	-0.61584100	-0.27401000
H	-4.33012000	0.27403900	-1.47913000

72RE

C	1.89976900	1.26679100	0.38128000
C	-0.07825200	1.80321000	-1.11304000
C	-1.24236100	1.24958900	-0.26590000
O	2.52673900	0.48480200	-0.53456000
C	1.10466800	2.34686100	-0.30350000
H	-0.47032200	2.64529000	-1.69838000
H	1.78812800	2.89966100	-0.95893000
H	0.25766900	1.05652000	-1.84182000
H	-2.17117200	1.61213900	-0.72346000
H	0.75907700	3.05929100	0.45426000
O	1.90226900	1.07862100	1.59091000
H	-1.21031200	1.67304900	0.74575000
C	-1.30907000	-0.29600100	-0.19794000
H	-1.34367000	-0.68653100	-1.22280000
N	-0.11413000	-0.80973000	0.45850000
C	3.10626000	-0.74901800	-0.06969000
H	3.56015000	-1.21401700	-0.95213000
H	3.92416000	-0.52838700	0.62586000
C	2.11062100	-1.73352800	0.54603000
H	1.97317100	-1.51617800	1.61145000
H	2.49522100	-2.75783800	0.49535000
C	0.77675100	-1.66235900	-0.15951000
O	0.55857100	-2.24339900	-1.21591000
H	0.14706000	-0.40098000	1.35224000
C	-2.55790000	-0.79290200	0.56074000
H	-2.49836900	-1.88346200	0.67365000
H	-2.55598000	-0.38390200	1.57971000
C	-3.85374000	-0.47937200	-0.13576000
H	-3.99112000	-0.90602300	-1.12802000
C	-4.83401100	0.26251700	0.39489000
H	-5.75210100	0.44427600	-0.15606000
H	-4.74972100	0.70027700	1.38473000

Gaussian Calculations

72$_{RO}$

M06-2X/6-31G* with Solvent Correction

SCF Done: E(RM062X) = -709.533557864

Zero-point correction= 0.277726
Thermal correction to Gibbs Free Energy= 0.234812

C	-0.00071500	1.70238100	-0.38138700
C	-2.49841400	1.61326300	0.30491300
C	-1.00123300	-0.37700400	0.62360500
C	-2.06603200	0.50981500	1.25928500
N	0.16909100	0.42790900	0.18535200
C	-1.27927800	2.45562100	-0.05521800
H	-3.26057300	2.24972800	0.76253800
H	-0.61848900	-1.06723500	1.37741800
H	-2.91257300	-0.12207900	1.54228900
H	-1.45995600	3.12920200	-0.89524500
H	-2.94720700	1.18095500	-0.59583600
H	-1.66116800	0.95106000	2.17801700
H	-1.01023100	3.08670900	0.80326900
O	0.86306800	2.23174000	-1.05041900
C	1.39893700	-0.24548300	0.18970400
O	1.42023600	-1.42695500	0.50611000
C	2.68701700	0.48956100	-0.12002300
H	2.74059000	0.66178200	-1.19879700
H	2.67685800	1.47882700	0.34479400
C	3.89265400	-0.32593500	0.34196000
H	3.82138400	-0.51466000	1.42237300
H	4.79926400	0.25886000	0.16590600
C	-1.51481600	-1.20666400	-0.56813100
H	-1.81340400	-0.54279700	-1.38805800
H	-0.67389000	-1.81064800	-0.92840400
C	-2.65904900	-2.10008100	-0.18798300
H	-2.45829900	-2.82672600	0.60028300
C	-3.86960900	-2.04778400	-0.73776200
H	-4.66666700	-2.71735000	-0.43018500
H	-4.10179300	-1.33505600	-1.52613900
O	4.03924800	-1.53157000	-0.37825000
H	3.24532300	-2.04780400	-0.16497900

72$_{RC}$

M06-2X/6-31G* with Solvent Correction

SCF Done: E(RM062X) = -709.533821645

Zero-point correction= 0.279492
Thermal correction to Gibbs Free Energy= 0.239352

C	1.10331200	0.91948300	-0.09683800
C	-1.13368200	2.11939700	0.00479200
C	-0.96393300	-0.33451400	0.64167100
C	-1.36137700	1.06562800	1.10087700
N	0.45480100	-0.34578600	0.23522100
C	0.12760900	1.82890000	-0.83235600
H	-1.05810300	3.10532000	0.47115200
H	-1.05108000	-1.03274600	1.48012700
H	-2.41547600	1.04712400	1.39182700
H	-0.11945100	1.33205900	-1.77493400
H	-2.00014700	2.15621100	-0.66329700
H	-0.77492200	1.31874000	1.98660900
H	0.66277800	2.74826200	-1.09108000
O	1.51763900	1.48567500	1.12733500
H	1.90671500	2.35197800	0.92266800
O	2.19936700	0.72532500	-0.93629000
C	3.14944400	-0.18342200	-0.38524600
H	4.01465700	-0.16097500	-1.04902800
C	1.10228200	-1.54765100	0.21485900
O	0.54492300	-2.58574400	0.54959800
H	3.45346700	0.16118400	0.60775400
C	2.52199600	-1.56391500	-0.32207800
H	2.45896900	-1.99705100	-1.32667400
H	3.10485500	-2.25169400	0.29489900
C	-1.84721900	-0.86006200	-0.50736100
H	-1.77589900	-0.19019600	-1.37244800
H	-1.44652100	-1.83515200	-0.80653100
C	-3.28226800	-1.00383600	-0.09321300
H	-3.47506700	-1.68702200	0.73512900
C	-4.29947000	-0.35490500	-0.65469800
H	-5.32242100	-0.49179500	-0.31801100
H	-4.14155000	0.33358300	-1.48230800

72RE

M06-2X/6-31G* with Solvent Correction

SCF Done: E(RM062X) = -709.546257948

Zero-point correction= 0.278896
Thermal correction to Gibbs Free Energy= 0.237060

C	1.88766600	1.24662900	0.37997200
C	-0.11483500	1.75540000	-1.10218100
C	-1.28333100	1.20107600	-0.26241000
O	2.48688400	0.46073600	-0.52774600
C	1.07199000	2.31978900	-0.29544200
H	-0.48436200	2.57152200	-1.72936800
H	1.73726600	2.86146200	-0.97571800
H	0.25756900	0.98206600	-1.78462800
H	-2.23442400	1.53910900	-0.68581000
H	0.72565500	3.01243400	0.47508400
O	1.98469800	1.07280800	1.57544300
H	-1.24598000	1.60961600	0.75867500
C	-1.33522000	-0.32851600	-0.17550000
H	-1.33523600	-0.75012200	-1.18634700
N	-0.12730600	-0.83533600	0.45759300
C	3.12555100	-0.73263000	-0.04827300
H	3.57789800	-1.16030600	-0.94203600
H	3.90341700	-0.46742100	0.67111400
C	2.11926200	-1.71509200	0.57134900
H	1.98953300	-1.50729800	1.63461400
H	2.49967700	-2.73201100	0.45247500
C	0.80353500	-1.59079900	-0.18194500
O	0.64528100	-2.06401200	-1.29888500
H	0.12058200	-0.44607500	1.35971600
C	-2.57966000	-0.82672100	0.57773400
H	-2.49069500	-1.91562800	0.67693700
H	-2.58439800	-0.40493900	1.59150200
C	-3.86123300	-0.48365400	-0.12549800
H	-3.97209000	-0.88316100	-1.13449300
C	-4.83497800	0.25975200	0.39332900
H	-5.74508400	0.47549900	-0.15764500
H	-4.75556900	0.67494600	1.39549200

Isomers $27v_{RO}/_{RC}/_{RE}$

Spartan Calculations

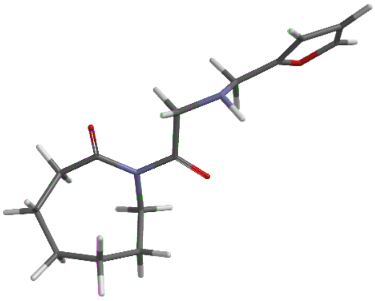

$27v_{RO}$
$\Delta G° = 8.1$ kcal/mol

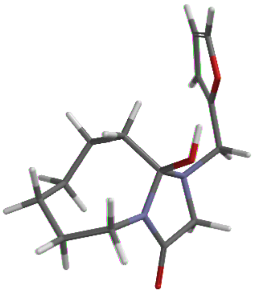

$27v_{RC}$
$\Delta G° = 16.7$ kcal/mol

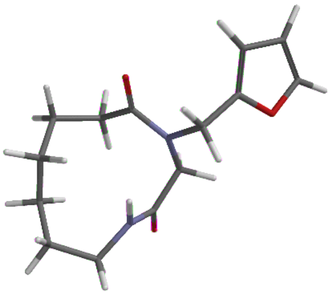

$27v_{RE}$
$\Delta G° = 0.0$ kcal/mol

$27v_{RO}$	Calculated energy (au)	Calculated energy (kcal/mol)	Relative energy (kcal/mol)
M0003	-880.681955	-552627.9268	0.00
M0010	-880.68164	-552627.7291	0.20
M0022	-880.681139	-552627.4147	0.51
M0028	-880.68101	-552627.3338	0.59
M0012	-880.680549	-552627.0445	0.88
M0016	-880.680367	-552626.9303	1.00
M0021	-880.678915	-552626.0192	1.91
M0018	-880.678733	-552625.905	2.02
M0041	-880.678581	-552625.8096	2.12
M0059	-880.678464	-552625.7362	2.19
M0044	-880.678325	-552625.6489	2.28
M0065	-880.678193	-552625.5661	2.36
M0030	-880.678111	-552625.5147	2.41
M0045	-880.67799	-552625.4387	2.49
M0001	-880.677937	-552625.4055	2.52
M0002	-880.677902	-552625.3835	2.54
M0075	-880.677803	-552625.3214	2.61
M0004	-880.677732	-552625.2768	2.65
M0020	-880.677442	-552625.0949	2.83
M0082	-880.67719	-552624.9367	2.99
M0006	-880.677155	-552624.9148	3.01
M0063	-880.676983	-552624.8068	3.12
M0066	-880.676931	-552624.7742	3.15
M0093	-880.676891	-552624.7491	3.18
M0024	-880.676707	-552624.6336	3.29
M0064	-880.676653	-552624.5998	3.33
M0009	-880.676196	-552624.313	3.61
M0038	-880.676139	-552624.2772	3.65
M0049	-880.675994	-552624.1862	3.74
M0025	-880.675991	-552624.1844	3.74

M0043	-880.675929	-552624.1454	3.78
M0055	-880.675836	-552624.0871	3.84
M0019	-880.675828	-552624.0821	3.84
M0008	-880.675716	-552624.0118	3.91
M0035	-880.675696	-552623.9992	3.93
M0048	-880.675597	-552623.9371	3.99
M0017	-880.675352	-552623.7834	4.14
M0027	-880.675237	-552623.7112	4.22
M0072	-880.674981	-552623.5506	4.38
M0096	-880.674829	-552623.4552	4.47
M0061	-880.674669	-552623.3548	4.57
M0056	-880.674627	-552623.3284	4.60
M0005	-880.674503	-552623.2506	4.68
M0031	-880.674465	-552623.2268	4.70
M0015	-880.674246	-552623.0894	4.84
M0089	-880.674211	-552623.0674	4.86
M0014	-880.674171	-552623.0423	4.88
M0011	-880.674052	-552622.9676	4.96
M0032	-880.674027	-552622.9519	4.97
M0007	-880.67392	-552622.8848	5.04
M0023	-880.673728	-552622.7643	5.16
M0037	-880.673568	-552622.6639	5.26
M0094	-880.673442	-552622.5849	5.34
M0034	-880.673157	-552622.406	5.52
M0033	-880.673064	-552622.3477	5.58
M0042	-880.67298	-552622.295	5.63
M0013	-880.672876	-552622.2297	5.70
M0057	-880.672741	-552622.145	5.78
M0070	-880.672671	-552622.1011	5.83
M0026	-880.672465	-552621.9718	5.95
M0076	-880.672219	-552621.8174	6.11
M0069	-880.672066	-552621.7214	6.21
M0054	-880.671858	-552621.5909	6.34
M0073	-880.671812	-552621.562	6.36
M0052	-880.671796	-552621.552	6.37
M0039	-880.671617	-552621.4397	6.49
M0051	-880.671481	-552621.3543	6.57
M0029	-880.671322	-552621.2546	6.67
M0060	-880.671299	-552621.2401	6.69
M0036	-880.671061	-552621.0908	6.84
M0068	-880.670946	-552621.0186	6.91
M0062	-880.670714	-552620.873	7.05
M0078	-880.670431	-552620.6955	7.23
M0074	-880.670326	-552620.6296	7.30
M0053	-880.670198	-552620.5492	7.38
M0099	-880.670064	-552620.4652	7.46

M0067	-880.669909	-552620.3679	7.56
M0087	-880.669902	-552620.3635	7.56
M0040	-880.66976	-552620.2744	7.65
M0058	-880.669623	-552620.1884	7.74
M0097	-880.669508	-552620.1163	7.81
M0088	-880.669249	-552619.9537	7.97
M0092	-880.669186	-552619.9142	8.01
M0081	-880.669182	-552619.9117	8.02
M0100	-880.668592	-552619.5415	8.39
M0085	-880.668255	-552619.33	8.60
M0080	-880.668153	-552619.266	8.66
M0091	-880.66813	-552619.2516	8.68
M0077	-880.668045	-552619.1982	8.73
M0079	-880.667789	-552619.0376	8.89
M0071	-880.667314	-552618.7395	9.19
M0090	-880.666986	-552618.5337	9.39
M0047	-880.666831	-552618.4365	9.49
M0050	-880.666567	-552618.2708	9.66
M0046	-880.665851	-552617.8215	10.11
M0086	-880.665688	-552617.7192	10.21
M0084	-880.665493	-552617.5969	10.33
M0083	-880.664727	-552617.1162	10.81
M0098	-880.664693	-552617.0949	10.83
M0095	-880.664075	-552616.7071	11.22

**27v_{RC}**	**Calculated energy (au)**	**Calculated energy (kcal/mol)**	**Relative energy (kcal/mol)**
M0008	-880.688293	-552631.9039	0.00
M0042	-880.688183	-552631.8348	0.07
M0043	-880.688176	-552631.8304	0.07
M0013	-880.687354	-552631.3146	0.59
M0004	-880.687006	-552631.0963	0.81
M0001	-880.68603	-552630.4838	1.42
M0019	-880.685937	-552630.4255	1.48
M0020	-880.685922	-552630.4161	1.49
M0007	-880.685772	-552630.3219	1.58
M0003	-880.685755	-552630.3113	1.59
M0009	-880.685696	-552630.2742	1.63
M0023	-880.685507	-552630.1556	1.75
M0011	-880.68529	-552630.0195	1.88
M0031	-880.685288	-552630.0182	1.89
M0002	-880.685271	-552630.0076	1.90
M0014	-880.684964	-552629.8149	2.09
M0015	-880.684537	-552629.547	2.36
M0025	-880.684046	-552629.2389	2.66
M0034	-880.683812	-552629.092	2.81
M0022	-880.683703	-552629.0236	2.88
M0040	-880.6837	-552629.0218	2.88

M0005	-880.683681	-552629.0098	2.89
M0012	-880.68366	-552628.9967	2.91
M0010	-880.683467	-552628.8755	3.03
M0006	-880.683327	-552628.7877	3.12
M0036	-880.683308	-552628.7758	3.13
M0028	-880.683193	-552628.7036	3.20
M0039	-880.68317	-552628.6892	3.21
M0041	-880.683049	-552628.6132	3.29
M0033	-880.682638	-552628.3553	3.55
M0055	-880.682412	-552628.2135	3.69
M0047	-880.682406	-552628.2098	3.69
M0027	-880.682307	-552628.1476	3.76
M0037	-880.6823	-552628.1433	3.76
M0029	-880.682242	-552628.1069	3.80
M0017	-880.682219	-552628.0924	3.81
M0021	-880.682032	-552627.9751	3.93
M0024	-880.681633	-552627.7247	4.18
M0035	-880.681206	-552627.4568	4.45
M0057	-880.681032	-552627.3476	4.56
M0098	-880.680936	-552627.2873	4.62
M0016	-880.680476	-552626.9987	4.91
M0085	-880.680361	-552626.9265	4.98
M0061	-880.680269	-552626.8688	5.04
M0038	-880.680205	-552626.8286	5.08
M0026	-880.680012	-552626.7075	5.20
M0048	-880.679953	-552626.6705	5.23
M0069	-880.679872	-552626.6197	5.28
M0018	-880.679867	-552626.6165	5.29
M0050	-880.679717	-552626.5224	5.38
M0044	-880.679645	-552626.4772	5.43
M0032	-880.679136	-552626.1578	5.75
M0068	-880.679018	-552626.0838	5.82
M0075	-880.678974	-552626.0562	5.85
M0030	-880.678778	-552625.9332	5.97
M0056	-880.678681	-552625.8723	6.03
M0049	-880.678549	-552625.7895	6.11
M0079	-880.678413	-552625.7042	6.20
M0065	-880.678124	-552625.5228	6.38
M0063	-880.678123	-552625.5222	6.38
M0074	-880.678116	-552625.5178	6.39
M0096	-880.678052	-552625.4776	6.43
M0077	-880.677883	-552625.3716	6.53
M0094	-880.67759	-552625.1877	6.72
M0082	-880.676988	-552624.81	7.09
M0053	-880.676978	-552624.8037	7.10
M0045	-880.676955	-552624.7893	7.11

M0072	-880.676838	-552624.7158	7.19
M0090	-880.676529	-552624.5219	7.38
M0086	-880.676098	-552624.2515	7.65
M0046	-880.674097	-552622.9959	8.91
M0066	-880.674015	-552622.9444	8.96
M0070	-880.673503	-552622.6231	9.28
M0062	-880.673298	-552622.4945	9.41
M0073	-880.671708	-552621.4968	10.41
M0054	-880.671042	-552621.0789	10.83
M0099	-880.670665	-552620.8423	11.06
M0059	-880.670316	-552620.6233	11.28
M0060	-880.670205	-552620.5536	11.35
M0058	-880.669901	-552620.3629	11.54
M0083	-880.669845	-552620.3277	11.58
M0052	-880.669516	-552620.1213	11.78
M0091	-880.669348	-552620.0159	11.89
M0076	-880.669136	-552619.8828	12.02
M0051	-880.669073	-552619.8433	12.06
M0097	-880.66907	-552619.8414	12.06
M0080	-880.66894	-552619.7599	12.14
M0078	-880.668313	-552619.3664	12.54
M0088	-880.668108	-552619.2378	12.67
M0095	-880.667499	-552618.8556	13.05
M0084	-880.667492	-552618.8512	13.05
M0064	-880.667134	-552618.6266	13.28
M0089	-880.667031	-552618.562	13.34
M0081	-880.66516	-552617.3879	14.52
M0093	-880.665144	-552617.3779	14.53
M0087	-880.66439	-552616.9047	15.00
M0092	-880.663847	-552616.564	15.34
M0071	-880.663824	-552616.5496	15.35
M0100	-880.662897	-552615.9679	15.94
M0067	-880.662497	-552615.7169	16.19
**27VRE**	**Calculated energy (au)**	**Calculated energy (kcal/mol)**	**Relative energy (kcal/mol)**
M0009	-880.703212	-552641.2655	0.00
M0003	-880.703157	-552641.231	0.03
M0005	-880.702786	-552640.9982	0.27
M0002	-880.702595	-552640.8784	0.39
M0019	-880.702243	-552640.6575	0.61
M0001	-880.702137	-552640.591	0.67
M0004	-880.701961	-552640.4805	0.79
M0037	-880.701912	-552640.4498	0.82
M0016	-880.701897	-552640.4404	0.83
M0012	-880.701826	-552640.3958	0.87
M0015	-880.701577	-552640.2396	1.03
M0010	-880.701539	-552640.2157	1.05

M0021	-880.701442	-552640.1549	1.11
M0008	-880.701428	-552640.1461	1.12
M0006	-880.701223	-552640.0174	1.25
M0011	-880.700827	-552639.7689	1.50
M0027	-880.700304	-552639.4408	1.82
M0024	-880.700245	-552639.4037	1.86
M0036	-880.700149	-552639.3435	1.92
M0020	-880.700058	-552639.2864	1.98
M0032	-880.700035	-552639.272	1.99
M0049	-880.699947	-552639.2167	2.05
M0046	-880.699587	-552638.9908	2.27
M0013	-880.699302	-552638.812	2.45
M0026	-880.699287	-552638.8026	2.46
M0018	-880.698805	-552638.5001	2.77
M0051	-880.698644	-552638.3991	2.87
M0064	-880.698546	-552638.3376	2.93
M0025	-880.69834	-552638.2084	3.06
M0048	-880.698271	-552638.1651	3.10
M0045	-880.698267	-552638.1625	3.10
M0075	-880.697964	-552637.9724	3.29
M0023	-880.697925	-552637.9479	3.32
M0038	-880.697766	-552637.8482	3.42
M0017	-880.697568	-552637.7239	3.54
M0007	-880.697511	-552637.6882	3.58
M0014	-880.697448	-552637.6486	3.62
M0035	-880.697404	-552637.621	3.64
M0055	-880.697254	-552637.5269	3.74
M0028	-880.697183	-552637.4823	3.78
M0076	-880.697069	-552637.4108	3.85
M0039	-880.696667	-552637.1585	4.11
M0053	-880.696529	-552637.0719	4.19
M0033	-880.696456	-552637.0261	4.24
M0060	-880.696455	-552637.0255	4.24
M0029	-880.696299	-552636.9276	4.34
M0052	-880.696197	-552636.8636	4.40
M0043	-880.696163	-552636.8423	4.42
M0047	-880.696149	-552636.8335	4.43
M0034	-880.695757	-552636.5875	4.68
M0057	-880.69572	-552636.5643	4.70
M0069	-880.695716	-552636.5618	4.70
M0065	-880.695696	-552636.5492	4.72
M0059	-880.695516	-552636.4363	4.83
M0056	-880.695447	-552636.393	4.87
M0040	-880.695156	-552636.2104	5.06
M0070	-880.695125	-552636.1909	5.07
M0058	-880.6948	-552635.987	5.28

M0022	-880.694756	-552635.9594	5.31
M0031	-880.694695	-552635.9211	5.34
M0061	-880.694498	-552635.7975	5.47
M0041	-880.694327	-552635.6902	5.58
M0042	-880.694325	-552635.6889	5.58
M0030	-880.694282	-552635.662	5.60
M0054	-880.694247	-552635.64	5.63
M0044	-880.693892	-552635.4172	5.85
M0050	-880.693844	-552635.3871	5.88
M0068	-880.693797	-552635.3576	5.91
M0071	-880.693717	-552635.3074	5.96
M0073	-880.693645	-552635.2622	6.00
M0066	-880.693638	-552635.2578	6.01
M0062	-880.693501	-552635.1719	6.09
M0063	-880.693454	-552635.1424	6.12
M0074	-880.693201	-552634.9836	6.28
M0077	-880.693069	-552634.9008	6.36
M0072	-880.69222	-552634.3681	6.90
M0067	-880.692087	-552634.2846	6.98
M0079	-880.691306	-552633.7945	7.47
M0078	-880.689801	-552632.8501	8.42

Table S40. Energies calculated at DFT/B3LYP/6-31G* in vacuum for the conformers found for **27v$_{RO}$/27v$_{RC}$/27v$_{RE}$**.

Isomer	ΔG° (au)	ΔG° (kcal/mol)	ΔG° (kcal/mol)
**27v$_{RO}$**	-880.411892	-552458.46	8.05
**27v$_{RC}$**	-880.39805	-552449.78	16.74
**27v$_{RE}$**	-880.424722	-552466.51	0.00

Table S41. Relative free energies (ΔG°) of the lowest energy geometries of **27v$_{RO}$/27v$_{RC}$/27v$_{RE}$** at DFT/B3LYP/6-31G* in vacuum in kcal/mol.

XYZ Coordinates

27$_{VRO}$

H	-3.66343000	-0.43990000	1.63211000
C	-4.07151000	-1.20360000	0.95980000
H	-4.64067000	-1.88832000	1.60228000
C	-2.94014000	-2.02003000	0.33387000
H	-3.36353000	-2.93008000	-0.10978000
H	-2.27831000	-2.35608000	1.14141000
C	-2.11841000	-1.30593000	-0.74032000
H	-1.31069000	-1.97668000	-1.06104000
H	-2.72349000	-1.15407000	-1.63515000
N	-1.53111000	-0.04669000	-0.28025000
C	-2.25249000	1.14394000	-0.29945000
C	-5.03833000	-0.58214000	-0.04854000
H	-6.04791000	-0.65060000	0.37796000
H	-5.06628000	-1.15579000	-0.98201000
C	-4.78135000	0.89973000	-0.33187000
C	-3.52283000	1.21336000	-1.13205000
H	-5.63970000	1.27849000	-0.90156000
H	-4.77241000	1.45549000	0.61415000
H	-3.59152000	2.25330000	-1.48127000
H	-3.45631000	0.62773000	-2.05096000
O	-1.92972000	2.14131000	0.34926000
C	-0.29672000	-0.17435000	0.37575000
O	0.14828000	-1.27814000	0.71732000
C	0.51638000	1.10323000	0.66789000
H	0.40764000	1.83883000	-0.13452000
H	0.14472000	1.52824000	1.60561000
N	1.95118000	0.78456000	0.85557000
H	1.99853000	-0.08524000	1.39643000
C	2.61684000	0.53908000	-0.42829000
H	2.56486000	1.43809000	-1.05379000
H	2.12503000	-0.27527000	-0.97294000
C	4.05220000	0.20026000	-0.22330000
O	4.54720000	-0.83522000	-0.96318000
C	5.05145000	0.72691000	0.57083000
H	4.93814000	1.55475000	1.25724000
C	6.21592000	-0.03266000	0.30825000
H	7.19499000	0.08671000	0.74954000
C	5.85675000	-0.97025000	-0.63086000
H	6.38727000	-1.76355000	-1.13831000

27_{VRC}

H	2.39035000	2.82750000	-0.67710000
C	2.84234000	2.23161000	0.12363000
H	3.62651000	2.86390000	0.56079000
C	3.54527000	0.99820000	-0.45450000
H	4.04268000	0.45488000	0.35864000
H	4.34178000	1.34689000	-1.12375000
C	2.65137000	0.04391000	-1.24560000
H	2.17923000	0.55105000	-2.09274000
H	3.28462000	-0.74761000	-1.66486000
N	1.64850000	-0.59625000	-0.41962000
C	0.24740000	-0.29799000	-0.49358000
C	1.82948000	1.92074000	1.22991000
H	2.05042000	0.96102000	1.70948000
H	1.95385000	2.67076000	2.02260000
C	0.36221000	1.98037000	0.79736000
C	-0.02584000	1.22211000	-0.47492000
H	-0.26142000	1.63898000	1.63259000
H	0.09744000	3.03501000	0.64394000
H	0.45460000	1.69695000	-1.33927000
H	-1.08866000	1.41077000	-0.65759000
O	-0.18581000	-0.83801000	-1.72542000
H	-1.09803000	-0.52159000	-1.84623000
N	-0.32833000	-1.01343000	0.64563000
C	1.91375000	-1.69273000	0.36670000
O	2.99267000	-2.23323000	0.54926000
C	0.59855000	-2.12146000	0.95327000
H	0.31609000	-3.06603000	0.47408000
H	0.69286000	-2.25138000	2.03468000
C	-1.68172000	-1.54888000	0.48386000
H	-1.94754000	-2.10889000	1.39094000
H	-1.73939000	-2.27056000	-0.34172000
C	-2.72215000	-0.50072000	0.30549000
O	-3.11832000	-0.25104000	-0.97819000
C	-3.41247000	0.31360000	1.17901000
H	-3.29560000	0.33531000	2.25387000
C	-4.28604000	1.09711000	0.38742000
H	-4.98659000	1.84830000	0.72445000
C	-4.07325000	0.71325000	-0.91576000
H	-4.49623000	1.01604000	-1.86382000

27VRE

H	4.77982000	-0.95559000	-0.39245000
C	3.80007000	-0.53008000	-0.14013000
H	3.42461000	-0.07604000	-1.06298000
C	3.99488000	0.54107000	0.94389000
H	5.03147000	0.89882000	0.91126000
H	3.85444000	0.10275000	1.93957000
C	3.07833000	1.76002000	0.78289000
H	3.10396000	2.36310000	1.69646000
C	-0.11569000	-1.31692000	-0.14262000
C	2.90541000	-1.68090000	0.34535000
H	2.22472000	-1.34781000	1.13588000
H	3.55210000	-2.43562000	0.81147000
C	2.10341000	-2.35750000	-0.77299000
C	0.90933000	-1.52346000	-1.25480000
H	2.76213000	-2.57524000	-1.62199000
H	1.74035000	-3.32396000	-0.40196000
H	0.39486000	-2.05823000	-2.06204000
H	1.27326000	-0.59114000	-1.68257000
O	-0.48618000	-2.27875000	0.53477000
H	3.44675000	2.38592000	-0.03684000
N	1.69775000	1.39587000	0.54621000
N	-0.58755000	-0.03436000	0.10358000
C	-0.35286000	1.11321000	-0.78633000
H	-1.11258000	1.88251000	-0.61390000
H	-0.43526000	0.77533000	-1.82443000
C	1.01934000	1.77333000	-0.59361000
O	1.45411000	2.59133000	-1.40030000
H	1.23567000	0.76080000	1.18649000
C	-1.58872000	0.14479000	1.15424000
H	-1.45411000	-0.60746000	1.93929000
H	-1.43399000	1.12468000	1.61997000
C	-2.95330000	0.02829000	0.57718000
O	-3.60903000	1.19515000	0.30979000
C	-3.74497000	-1.04710000	0.22969000
H	-3.47289000	-2.08984000	0.32961000
C	-4.95079000	-0.50791000	-0.27752000
H	-5.80995000	-1.04805000	-0.64944000
C	-4.81892000	0.85898000	-0.20730000
H	-5.46096000	1.68777000	-0.47159000

Isomers 53_{RO}/53_{RC}/53_{RE}

Spartan Calculations

The calculations below were done with the full structures of compound **53** (*i.e.* with the *N*-Bn protecting group instead of the simplified *N*-methyl group as shown in table **S18** and **S19**). At the same level of theory, both *N*-Bn and *N*-Me afforded very similar results.

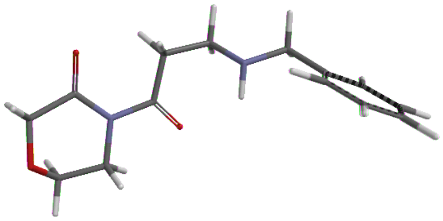

53_{RO}
$\Delta G°$ = 0.0 kcal/mol

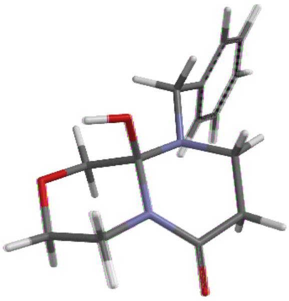

53_{RC}
$\Delta G°$ = 10.5 kcal/mol

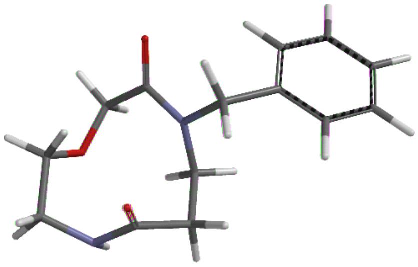

53_{RE}
$\Delta G°$ = 0.4 kcal/mol

53_{RO}	Calculated energy (au)	Calculated energy (kcal/mol)	Relative energy (kcal/mol)
M0023	-879.497702	-551884.808	0.00
M0046	-879.497629	-551884.7622	0.05
M0037	-879.497226	-551884.5093	0.30
M0014	-879.496814	-551884.2508	0.56
M0042	-879.496129	-551883.8209	0.99
M0036	-879.495668	-551883.5317	1.28
M0002	-879.495532	-551883.4463	1.36
M0080	-879.495287	-551883.2926	1.52
M0091	-879.494942	-551883.0761	1.73
M0009	-879.494834	-551883.0083	1.80
M0001	-879.494731	-551882.9437	1.86
M0038	-879.494246	-551882.6394	2.17
M0084	-879.494163	-551882.5873	2.22
M0075	-879.494046	-551882.5139	2.29
M0006	-879.493876	-551882.4072	2.40
M0004	-879.493386	-551882.0997	2.71
M0070	-879.493297	-551882.0439	2.76
M0076	-879.493184	-551881.973	2.84
M0007	-879.493092	-551881.9152	2.89
M0012	-879.493074	-551881.9039	2.90
M0003	-879.493065	-551881.8983	2.91
M0067	-879.493049	-551881.8882	2.92
M0005	-879.492985	-551881.8481	2.96
M0028	-879.492977	-551881.8431	2.96
M0011	-879.492937	-551881.818	2.99
M0010	-879.492862	-551881.7709	3.04
M0093	-879.492815	-551881.7414	3.07

M0016	-879.492782	-551881.7207	3.09
M0033	-879.492776	-551881.7169	3.09
M0057	-879.492699	-551881.6686	3.14
M0096	-879.49264	-551881.6316	3.18
M0035	-879.492594	-551881.6027	3.21
M0072	-879.492436	-551881.5036	3.30
M0090	-879.492419	-551881.4929	3.32
M0100	-879.492322	-551881.4321	3.38
M0051	-879.492214	-551881.3643	3.44
M0083	-879.492176	-551881.3404	3.47
M0031	-879.492162	-551881.3317	3.48
M0078	-879.492108	-551881.2978	3.51
M0065	-879.491994	-551881.2262	3.58
M0059	-879.491974	-551881.2137	3.59
M0018	-879.491925	-551881.1829	3.63
M0025	-879.4917	-551881.0418	3.77
M0043	-879.491679	-551881.0286	3.78
M0027	-879.49163	-551880.9978	3.81
M0061	-879.49157	-551880.9602	3.85
M0013	-879.491524	-551880.9313	3.88
M0071	-879.491515	-551880.9257	3.88
M0019	-879.491494	-551880.9125	3.90
M0086	-879.491476	-551880.9012	3.91
M0040	-879.491453	-551880.8868	3.92
M0044	-879.491432	-551880.8736	3.93
M0024	-879.491432	-551880.8736	3.93
M0069	-879.491382	-551880.8422	3.97
M0056	-879.491194	-551880.7242	4.08
M0049	-879.491099	-551880.6646	4.14
M0032	-879.491013	-551880.6107	4.20
M0017	-879.490936	-551880.5623	4.25
M0015	-879.490917	-551880.5504	4.26
M0053	-879.490915	-551880.5492	4.26
M0098	-879.490864	-551880.5172	4.29
M0008	-879.490842	-551880.5034	4.30
M0047	-879.490767	-551880.4563	4.35
M0062	-879.490747	-551880.4437	4.36
M0085	-879.490652	-551880.3841	4.42
M0041	-879.490638	-551880.3753	4.43
M0088	-879.490523	-551880.3032	4.50
M0020	-879.490504	-551880.2913	4.52
M0094	-879.490406	-551880.2298	4.58
M0099	-879.490385	-551880.2166	4.59
M0081	-879.490307	-551880.1676	4.64
M0045	-879.490255	-551880.135	4.67
M0026	-879.490158	-551880.0741	4.73

M0066	-879.490017	-551879.9857	4.82
M0039	-879.490009	-551879.9806	4.83
M0054	-879.489789	-551879.8426	4.97
M0082	-879.489737	-551879.81	5.00
M0055	-879.489702	-551879.788	5.02
M0052	-879.489587	-551879.7158	5.09
M0030	-879.489579	-551879.7108	5.10
M0022	-879.489482	-551879.65	5.16
M0034	-879.489478	-551879.6474	5.16
M0068	-879.489456	-551879.6336	5.17
M0048	-879.48919	-551879.4667	5.34
M0029	-879.489181	-551879.4611	5.35
M0064	-879.489132	-551879.4303	5.38
M0060	-879.489102	-551879.4115	5.40
M0021	-879.488796	-551879.2195	5.59
M0089	-879.488598	-551879.0952	5.71
M0063	-879.488557	-551879.0695	5.74
M0087	-879.488511	-551879.0407	5.77
M0073	-879.488487	-551879.0256	5.78
M0097	-879.488426	-551878.9873	5.82
M0079	-879.487948	-551878.6874	6.12
M0074	-879.487685	-551878.5223	6.29
M0092	-879.487654	-551878.5029	6.31
M0058	-879.487577	-551878.4546	6.35
M0077	-879.487429	-551878.3617	6.45
M0095	-879.486545	-551877.807	7.00
M0050	-879.485432	-551877.1086	7.70
**53_{RC}**	**Calculated energy (au)**	**Calculated energy (kcal/mol)**	**Relative energy (kcal/mol)**
M0001	-879.48322	-551875.7206	0.00
M0002	-879.481629	-551874.7222	1.00
M0007	-879.479466	-551873.3649	2.36
M0013	-879.479138	-551873.1591	2.56
M0003	-879.47898	-551873.06	2.66
M0005	-879.478919	-551873.0217	2.70
M0014	-879.478752	-551872.9169	2.80
M0010	-879.478468	-551872.7387	2.98
M0008	-879.478435	-551872.718	3.00
M0009	-879.478341	-551872.659	3.06
M0006	-879.478298	-551872.632	3.09
M0031	-879.476684	-551871.6192	4.10
M0024	-879.475811	-551871.0714	4.65
M0020	-879.4757	-551871.0018	4.72
M0018	-879.474976	-551870.5474	5.17
M0016	-879.474456	-551870.2211	5.50
M0032	-879.474359	-551870.1603	5.56
M0011	-879.474256	-551870.0956	5.62

M0023	-879.474034	-551869.9563	5.76
M0022	-879.473838	-551869.8333	5.89
M0017	-879.473513	-551869.6294	6.09
M0004	-879.473257	-551869.4688	6.25
M0029	-879.472746	-551869.1481	6.57
M0025	-879.472499	-551868.9931	6.73
M0015	-879.472289	-551868.8613	6.86
M0019	-879.472266	-551868.8469	6.87
M0026	-879.472008	-551868.685	7.04
M0028	-879.471956	-551868.6524	7.07
M0027	-879.471775	-551868.5388	7.18
M0012	-879.471306	-551868.2445	7.48
M0021	-879.470681	-551867.8523	7.87
M0030	-879.469677	-551867.2223	8.50
M0034	-879.469356	-551867.0209	8.70
M0033	-879.468359	-551866.3953	9.33

**53_{RE}**	**Calculated energy (au)**	**Calculated energy (kcal/mol)**	**Relative energy (kcal/mol)**
M0002	-879.503642	-551888.5354	0.00
M0003	-879.503187	-551888.2498	0.29
M0006	-879.502731	-551887.9637	0.57
M0001	-879.502331	-551887.7127	0.82
M0004	-879.501261	-551887.0413	1.49
M0005	-879.500164	-551886.3529	2.18
M0007	-879.499277	-551885.7963	2.74
M0009	-879.498992	-551885.6175	2.92
M0008	-879.49868	-551885.4217	3.11
M0011	-879.498417	-551885.2567	3.28
M0013	-879.497501	-551884.6819	3.85
M0012	-879.497455	-551884.653	3.88
M0015	-879.496456	-551884.0261	4.51
M0019	-879.49612	-551883.8153	4.72
M0010	-879.495058	-551883.1489	5.39
M0018	-879.494736	-551882.9468	5.59
M0017	-879.493815	-551882.3689	6.17
M0016	-879.493478	-551882.1574	6.38
M0014	-879.492312	-551881.4258	7.11
M0021	-879.490417	-551880.2367	8.30
M0020	-879.487443	-551878.3705	10.16

Table S42. Energies calculated at DFT/B3LYP/6-31G* in vacuum for the conformers found for **53_{RO}/53_{RC}/53_{RE}**.

Isomer	ΔG° (au)	ΔG° (kcal/mol)	ΔG° (kcal/mol)
**53_{RO}**	-879.244777	-551726.10	0.00
**53_{RC}**	-879.228125	-551715.65	10.45
**53_{RE}**	-879.244226	-551725.75	0.35

Table S43. Relative free energies (ΔG°) of the lowest energy geometries of **53_{RO}/53_{RC}/53_{RE}** at DFT/B3LYP/6-31G* in vacuum in kcal/mol.

XYZ Coordinates

53_{RO}

C	3.56077000	-0.69924000	0.62648000
O	5.21230000	1.22751000	0.92858000
C	3.28793000	1.50178000	-0.45259000
C	4.79779000	1.63851000	-0.36835000
N	2.81589000	0.13633000	-0.21660000
C	4.91030000	-0.14139000	1.12428000
H	2.81273000	2.12942000	0.31186000
H	5.30761000	1.03856000	-1.13159000
H	5.69850000	-0.73366000	0.64406000
H	2.95018000	1.84980000	-1.43517000
H	5.09220000	2.68368000	-0.50406000
H	4.96357000	-0.33109000	2.20159000
O	3.24970000	-1.85303000	0.92266000
C	1.50793000	-0.11987000	-0.66131000
O	0.84556000	0.75475000	-1.23105000
C	0.91893000	-1.50008000	-0.41026000
H	0.84523000	-1.67211000	0.66904000
H	1.59409000	-2.24166000	-0.85202000
C	-0.46006000	-1.69060000	-1.04823000
H	-0.42877000	-1.39313000	-2.10358000
H	-0.70804000	-2.75816000	-1.00960000
N	-1.49174000	-0.93461000	-0.33215000
H	-1.26170000	0.06194000	-0.39232000
C	-2.80639000	-1.13117000	-0.94189000
H	-2.78032000	-0.87215000	-2.00794000
H	-3.10585000	-2.18482000	-0.87787000
C	-3.85025000	-0.29060000	-0.24617000
C	-5.77371000	1.27587000	1.06555000
C	-4.33461000	-0.66383000	1.01610000
C	-4.33238000	0.88665000	-0.83577000
C	-5.29200000	1.66351000	-0.18369000
C	-5.29354000	0.11396000	1.66681000
H	-3.95356000	-1.56156000	1.49837000
H	-3.96035000	1.20676000	-1.80618000
H	-5.66065000	2.57363000	-0.64918000
H	-5.66024000	-0.18481000	2.64503000
H	-6.51837000	1.88254000	1.57355000

53_{RC}

C	1.24969000	0.43517000	-0.34533000
O	2.86412000	2.29936000	0.07212000
C	3.61005000	0.00853000	0.32946000
C	3.70216000	1.43798000	0.84932000
N	2.22916000	-0.47320000	0.25144000
C	1.48580000	1.88890000	0.21175000
H	4.02644000	-0.05119000	-0.68357000
H	3.40738000	1.49917000	1.90361000
H	1.19677000	1.96666000	1.26713000
H	4.20522000	-0.65105000	0.97065000
H	4.73058000	1.80306000	0.76732000
H	0.91730000	2.62630000	-0.36179000
O	1.51171000	0.45106000	-1.74073000
H	2.32344000	0.97832000	-1.83741000
N	-0.10569000	-0.05527000	-0.07617000
C	-1.14403000	0.80193000	-0.68438000
H	-1.09309000	0.75843000	-1.78040000
H	-1.00569000	1.85254000	-0.40971000
C	-0.24986000	-1.42841000	-0.58130000
H	-1.27180000	-1.79961000	-0.44224000
C	2.06701000	-1.84593000	0.20712000
O	3.00875000	-2.63513000	0.30429000
H	-0.04075000	-1.49107000	-1.65739000
C	0.65721000	-2.35301000	0.18895000
H	0.32309000	-2.45462000	1.22824000
H	0.64515000	-3.34701000	-0.27153000
C	-2.54966000	0.47437000	-0.21238000
C	-5.15779000	-0.08973000	0.65601000
C	-2.86002000	0.46256000	1.15635000
C	-3.56742000	0.20847000	-1.13992000
C	-4.86399000	-0.07448000	-0.70632000
C	-4.15667000	0.17893000	1.58728000
H	-2.08540000	0.66669000	1.89235000
H	-3.35687000	0.21669000	-2.20694000
H	-5.64572000	-0.28310000	-1.43213000
H	-4.38460000	0.16632000	2.64981000
H	-6.16729000	-0.31122000	0.99210000

53$_{RE}$

C	-0.48528000	-1.58673000	-0.26740000
O	-2.69323000	-1.24924000	0.98788000
C	-3.48858000	-0.97270000	-0.17724000
N	0.05687000	-0.31069000	-0.12178000
C	-1.57362000	-2.07364000	0.71799000
H	-1.07019000	-2.27092000	1.67127000
H	-4.22538000	-1.77082000	-0.31154000
H	-1.94278000	-3.04216000	0.35792000
O	-0.10772000	-2.36163000	-1.15297000
H	-2.86495000	-0.91963000	-1.07608000
C	-4.18063000	0.37984000	0.05117000
H	-4.61188000	0.76277000	-0.87884000
H	-4.97239000	0.27851000	0.80025000
N	-3.22290000	1.33377000	0.59488000
C	-0.24601000	0.61897000	0.97977000
H	-0.87723000	0.13201000	1.72499000
H	0.69558000	0.82955000	1.49970000
C	-0.87880000	1.94730000	0.54548000
H	-0.20328000	2.52765000	-0.09130000
H	-1.08653000	2.56642000	1.42600000
C	-2.16594000	1.71460000	-0.20792000
O	-2.23256000	1.78045000	-1.43110000
H	-3.00173000	1.15661000	1.56650000
C	1.07284000	0.10600000	-1.09643000
H	1.07816000	-0.55365000	-1.97199000
H	0.79785000	1.09329000	-1.48247000
C	2.46463000	0.12827000	-0.50787000
C	5.04064000	0.15760000	0.60218000
C	3.11160000	-1.07091000	-0.17541000
C	3.12766000	1.34282000	-0.27986000
C	4.40938000	1.35594000	0.27319000
C	4.39280000	-1.05551000	0.37717000
H	2.61634000	-2.02426000	-0.35285000
H	2.65129000	2.28681000	-0.53440000
H	4.91780000	2.30128000	0.44414000
H	4.88569000	-1.99115000	0.62792000
H	6.03976000	0.16841000	1.02969000

All the steps in the calculations above were repeated for compound **19** and **20** with the optimisations, energy and frequency calculations done using DFT/EDF2/6-31G* in vacuum.[16] The results are shown in table **S44−S47**.

Isomers 19_{RO}/19_{RC}/19_{RE}

Spartan Calculations

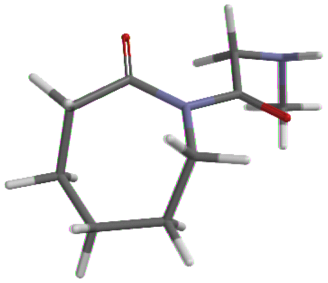

19_{RO}
ΔG ° = 0.46 kcal/mol

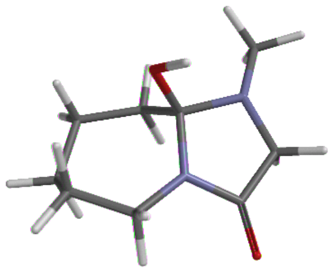

19_{RC}
ΔG ° = 4.36 kcal/mol

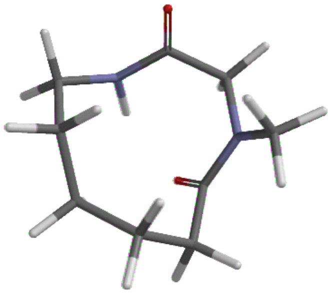

19_{RE}
ΔG ° = 0.00 kcal/mol

19_{RO}	Calculated energy (au)	Calculated energy (kcal/mol)	Relative energy (kcal/mol)
M0002	-612.120469	-384105.5943	0.00
M0004	-612.119854	-384105.2084	0.39
M0001	-612.118366	-384104.2747	1.32
M0003	-612.117479	-384103.7181	1.88
M0008	-612.117451	-384103.7005	1.89
M0017	-612.11716	-384103.5179	2.08
M0016	-612.117013	-384103.4257	2.17
M0009	-612.116767	-384103.2713	2.32
M0006	-612.116765	-384103.27	2.32
M0007	-612.116681	-384103.2173	2.38
M0028	-612.114786	-384102.0282	3.57
M0018	-612.114738	-384101.9981	3.60
M0010	-612.1147	-384101.9743	3.62
M0030	-612.114694	-384101.9705	3.62
M0014	-612.11453	-384101.8676	3.73
M0012	-612.114165	-384101.6385	3.96
M0011	-612.11402	-384101.5476	4.05
M0005	-612.113993	-384101.5306	4.06
M0023	-612.11379	-384101.4032	4.19
M0015	-612.113641	-384101.3097	4.28
M0013	-612.112721	-384100.7324	4.86
M0025	-612.111483	-384099.9556	5.64
M0033	-612.111265	-384099.8188	5.78
M0040	-612.110509	-384099.3444	6.25
M0037	-612.110447	-384099.3055	6.29

M0039	-612.110103	-384099.0896	6.50
M0026	-612.110078	-384099.0739	6.52
M0029	-612.110062	-384099.0639	6.53
M0036	-612.109479	-384098.6981	6.90
M0024	-612.109376	-384098.6334	6.96
M0021	-612.109167	-384098.5023	7.09
M0020	-612.107519	-384097.4682	8.13
M0022	-612.107471	-384097.4381	8.16
M0019	-612.107156	-384097.2404	8.35
M0047	-612.107111	-384097.2122	8.38
M0042	-612.106069	-384096.5583	9.04
M0046	-612.105758	-384096.3631	9.23
M0032	-612.105564	-384096.2414	9.35
M0045	-612.10505	-384095.9189	9.68
M0034	-612.104669	-384095.6798	9.91
M0038	-612.104203	-384095.3874	10.21
M0035	-612.103646	-384095.0379	10.56
M0031	-612.101795	-384093.8764	11.72
M0043	-612.100975	-384093.3618	12.23
M0027	-612.100691	-384093.1836	12.41
M0048	-612.10056	-384093.1014	12.49
M0041	-612.100537	-384093.087	12.51
M0044	-612.098396	-384091.7435	13.85
**19_{RC}**	**Calculated energy (au)**	**Calculated energy (kcal/mol)**	**Relative energy (kcal/mol)**
M0002	-612.118922	-384104.6236	0.00
M0017	-612.117102	-384103.4815	1.14
M0001	-612.115837	-384102.6877	1.94
M0016	-612.115712	-384102.6093	2.01
M0004	-612.115308	-384102.3558	2.27
M0003	-612.115282	-384102.3395	2.28
M0007	-612.114457	-384101.8218	2.80
M0018	-612.11358	-384101.2715	3.35
M0009	-612.112972	-384100.8899	3.73
M0005	-612.112715	-384100.7287	3.89
M0024	-612.112208	-384100.4105	4.21
M0006	-612.111301	-384099.8414	4.78
M0021	-612.110886	-384099.581	5.04
M0013	-612.110293	-384099.2089	5.41
M0015	-612.11007	-384099.0689	5.55
M0010	-612.10946	-384098.6862	5.94
M0008	-612.109149	-384098.491	6.13
M0020	-612.108977	-384098.3831	6.24
M0012	-612.107926	-384097.7236	6.90
M0014	-612.107396	-384097.391	7.23
M0019	-612.106404	-384096.7685	7.86
M0011	-612.105915	-384096.4617	8.16

M0022	-612.104419	-384095.5229	9.10
M0027	-612.102893	-384094.5654	10.06
M0023	-612.101606	-384093.7578	10.87
M0025	-612.101308	-384093.5708	11.05
M0026	-612.099772	-384092.6069	12.02
**19_{RE}**	**Calculated energy (au)**	**Calculated energy (kcal/mol)**	**Relative energy (kcal/mol)**
M0001	-612.128627	-384110.7134	0.00
M0003	-612.126745	-384109.5325	1.18
M0002	-612.126201	-384109.1911	1.52
M0005	-612.124598	-384108.1852	2.53
M0006	-612.124389	-384108.0541	2.66
M0004	-612.123028	-384107.2001	3.51
M0007	-612.122179	-384106.6673	4.05
M0009	-612.117345	-384103.634	7.08
M0008	-612.116958	-384103.3911	7.32
M0010	-612.116823	-384103.3064	7.41
M0011	-612.114656	-384101.9466	8.77

Table S44. Energies calculated at DFT/EDF2/6-31G* in vacuum for the conformers found for **19_{RO}/19_{RC}/19_{RE}**.

Isomer	ΔG° (au)	ΔG° (kcal/mol)	ΔG° (kcal/mol)
**19_{RO}**	-611.924795	-383982.81	0.46
**19_{RC}**	-611.918574	-383978.91	4.36
**19_{RE}**	-611.92553	-383983.27	0.00

Table S45. Relative free energies (ΔG°) of the lowest energy geometries of **19_{RO}/19_{RC}/19_{RE}** at DFT/EDF2/6-31G* in vacuum in kcal/mol.

XYZ Coordinates

19$_{RO}$

H	-2.88191000	1.18857000	0.77321000
C	-2.21797000	1.33682000	-0.08427000
H	-2.39962000	2.35775000	-0.44506000
C	-2.51826000	0.37818000	-1.23305000
H	-1.67859000	0.34233000	-1.93776000
H	-3.38129000	0.76710000	-1.78762000
C	-2.86707000	-1.03336000	-0.76800000
C	-1.71771000	-1.77493000	-0.08986000
H	-0.92589000	-1.98018000	-0.82055000
H	-2.09885000	-2.74690000	0.24641000
C	-1.14898000	-1.03009000	1.11493000
H	-1.95439000	-0.62225000	1.73643000
H	-0.61243000	-1.74472000	1.74886000
N	-0.24944000	0.05124000	0.71489000
C	-0.76645000	1.29152000	0.36136000
O	-0.13029000	2.34160000	0.45121000
C	1.11870000	-0.25290000	0.67865000
O	1.55537000	-1.33855000	1.08331000
C	2.08548000	0.80238000	0.10006000
H	1.66684000	1.28163000	-0.78971000
H	2.25565000	1.55719000	0.87436000
N	3.39578000	0.19591000	-0.23633000
H	3.61811000	-0.46938000	0.51094000
C	3.34101000	-0.53326000	-1.49759000
H	3.10792000	0.13855000	-2.32998000
H	2.60481000	-1.34280000	-1.47631000
H	4.32021000	-0.97797000	-1.70076000
H	-3.72537000	-0.99016000	-0.08577000
H	-3.19277000	-1.61934000	-1.63615000

19_{RC}

H	3.37732000	-1.19950000	-0.66340000
C	2.50194000	-0.54813000	-0.54657000
H	2.53863000	0.15110000	-1.39011000
C	2.62538000	0.21219000	0.76967000
C	1.84137000	1.52232000	0.77118000
C	0.31672000	1.37961000	0.82008000
H	-0.06732000	2.40720000	0.79797000
H	0.03219000	0.95327000	1.79091000
C	-0.31804000	0.56642000	-0.32558000
C	1.25954000	-1.42530000	-0.63761000
O	0.10717000	1.03443000	-1.57477000
H	-0.58517000	0.68368000	-2.15849000
N	0.01028000	-0.82439000	-0.22064000
N	-1.77462000	0.54044000	-0.35197000
H	2.14989000	2.10262000	1.65027000
H	2.12639000	2.12412000	-0.10082000
H	1.12541000	-1.75582000	-1.67454000
H	1.41318000	-2.33487000	-0.04425000
C	-1.04220000	-1.57000000	0.28197000
O	-1.05106000	-2.76108000	0.54423000
C	-2.18939000	-0.60723000	0.46318000
C	-2.46632000	1.76252000	0.00817000
H	-3.54105000	1.65235000	-0.17528000
H	-2.12789000	2.59933000	-0.61233000
H	-2.33778000	2.03298000	1.06188000
H	-3.11511000	-1.04522000	0.08127000
H	-2.27748000	-0.36946000	1.52883000
H	2.33263000	-0.41764000	1.61775000
H	3.68376000	0.46237000	0.91597000

19_{RE}

H	-0.67589000	1.46887000	-1.52715000
C	-0.60631000	2.13115000	-0.65714000
H	-0.85408000	3.13607000	-1.02255000
C	0.83297000	2.16580000	-0.10092000
C	1.80540000	1.19714000	-0.80751000
C	2.34848000	0.06821000	0.07849000
H	2.95831000	0.50103000	0.88191000
H	3.02440000	-0.58174000	-0.48466000
C	-1.67930000	1.74332000	0.37181000
N	-1.49498000	0.40157000	0.87460000
N	0.50255000	-1.62235000	-0.04606000
H	1.35741000	0.78582000	-1.71647000
H	2.67253000	1.77841000	-1.14801000
H	-1.65532000	2.42333000	1.22938000
H	-2.67025000	1.80869000	-0.09068000
C	-1.71925000	-0.69842000	0.06170000
O	-2.41662000	-0.67256000	-0.94875000
C	-0.85605000	-1.90920000	0.43968000
C	0.75298000	-1.90636000	-1.45174000
H	0.42267000	-2.92552000	-1.67326000
H	1.81516000	-1.83331000	-1.68968000
H	0.19353000	-1.20019000	-2.07081000
H	-0.79378000	0.28090000	1.60658000
C	1.25329000	-0.75057000	0.72930000
O	0.96771000	-0.53889000	1.91320000
H	-0.86716000	-2.04977000	1.52548000
H	-1.24678000	-2.81595000	-0.03164000
H	1.21443000	3.18177000	-0.27179000
H	0.84978000	2.03223000	0.98600000

Isomers 20_{RO}/20_{RC}/20_{RE}

Spartan Calculations

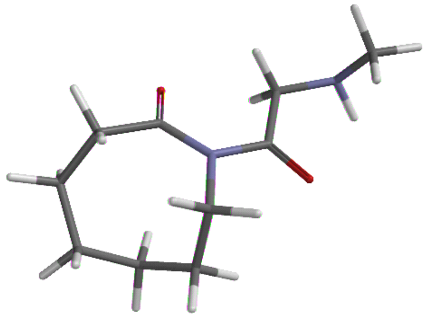

20_{RO}
$\Delta G°$ = 7.9 kcal/mol

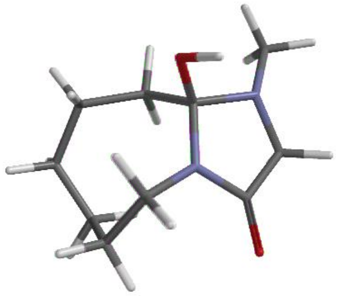

20_{RC}
$\Delta G°$ = 12.4 kcal/mol

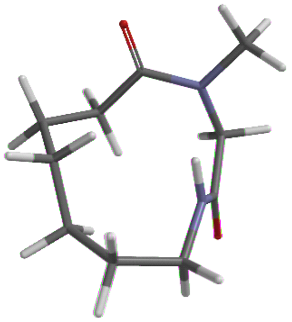

20_{RE}
$\Delta G°$ = 0.0 kcal/mol

20_{RO}	Calculated energy (au)	Calculated energy (kcal/mol)	Relative energy (kcal/mol)
M0007	-651.39478	-408750.2245	0.00
M0008	-651.39478	-408750.2245	0.00
M0009	-651.39478	-408750.2245	0.00
M0001	-651.392576	-408748.8414	1.38
M0002	-651.392576	-408748.8414	1.38
M0027	-651.392491	-408748.7881	1.44
M0028	-651.392491	-408748.7881	1.44
M0029	-651.392491	-408748.7881	1.44
M0013	-651.392178	-408748.5917	1.63
M0010	-651.392051	-408748.512	1.71
M0011	-651.392051	-408748.512	1.71
M0014	-651.390759	-408747.7013	2.52
M0015	-651.390759	-408747.7013	2.52
M0016	-651.390759	-408747.7013	2.52
M0003	-651.390451	-408747.508	2.72
M0004	-651.390451	-408747.508	2.72
M0012	-651.390252	-408747.3831	2.84
M0034	-651.390047	-408747.2545	2.97
M0035	-651.390047	-408747.2545	2.97
M0036	-651.390047	-408747.2545	2.97
M0020	-651.389977	-408747.2106	3.01
M0021	-651.389977	-408747.2106	3.01
M0017	-651.389745	-408747.065	3.16
M0018	-651.389745	-408747.065	3.16
M0019	-651.389745	-408747.065	3.16
M0005	-651.389625	-408746.9897	3.23
M0006	-651.389625	-408746.9897	3.23
M0031	-651.388682	-408746.398	3.83
M0030	-651.38837	-408746.2022	4.02
M0041	-651.387861	-408745.8828	4.34

M0042	-651.387861	-408745.8828	4.34
M0061	-651.387735	-408745.8037	4.42
M0062	-651.387735	-408745.8037	4.42
M0063	-651.387735	-408745.8037	4.42
M0048	-651.387617	-408745.7297	4.49
M0049	-651.387617	-408745.7297	4.49
M0073	-651.386681	-408745.1423	5.08
M0037	-651.386613	-408745.0997	5.12
M0023	-651.386404	-408744.9685	5.26
M0040	-651.385735	-408744.5487	5.68
M0043	-651.385717	-408744.5374	5.69
M0050	-651.385472	-408744.3837	5.84
M0052	-651.385434	-408744.3598	5.86
M0056	-651.385415	-408744.3479	5.88
M0046	-651.385345	-408744.304	5.92
M0047	-651.385345	-408744.304	5.92
M0091	-651.385261	-408744.2513	5.97
M0092	-651.385261	-408744.2513	5.97
M0024	-651.384922	-408744.0386	6.19
M0025	-651.384922	-408744.0386	6.19
M0026	-651.384922	-408744.0386	6.19
M0022	-651.384618	-408743.8478	6.38
M0057	-651.384224	-408743.6006	6.62
M0075	-651.384174	-408743.5692	6.66
M0076	-651.384174	-408743.5692	6.66
M0079	-651.383952	-408743.4299	6.79
M0085	-651.383841	-408743.3602	6.86
M0086	-651.383841	-408743.3602	6.86
M0087	-651.383841	-408743.3602	6.86
M0038	-651.38358	-408743.1965	7.03
M0039	-651.38358	-408743.1965	7.03
M0070	-651.382778	-408742.6932	7.53
M0044	-651.382539	-408742.5432	7.68
M0045	-651.382539	-408742.5432	7.68
M0078	-651.381998	-408742.2037	8.02
M0032	-651.381477	-408741.8768	8.35
M0033	-651.381477	-408741.8768	8.35
M0064	-651.381389	-408741.8216	8.40
M0095	-651.381013	-408741.5857	8.64
M0058	-651.380566	-408741.3052	8.92
M0059	-651.380566	-408741.3052	8.92
M0099	-651.380442	-408741.2274	9.00
M0054	-651.380088	-408741.0052	9.22
M0055	-651.380088	-408741.0052	9.22
M0096	-651.379852	-408740.8571	9.37
M0074	-651.379402	-408740.5748	9.65

M0077	-651.379265	-408740.4888	9.74
M0082	-651.379181	-408740.4361	9.79
M0065	-651.378864	-408740.2372	9.99
M0066	-651.378864	-408740.2372	9.99
M0051	-651.378427	-408739.9629	10.26
M0097	-651.378404	-408739.9485	10.28
M0053	-651.378302	-408739.8845	10.34
M0080	-651.378132	-408739.7778	10.45
M0093	-651.376295	-408738.6251	11.60
M0094	-651.376295	-408738.6251	11.60
M0083	-651.376013	-408738.4482	11.78
M0089	-651.375573	-408738.1721	12.05
M0090	-651.375573	-408738.1721	12.05
M0067	-651.37551	-408738.1325	12.09
M0068	-651.37551	-408738.1325	12.09
M0069	-651.37551	-408738.1325	12.09
M0088	-651.375446	-408738.0924	12.13
M0084	-651.375376	-408738.0484	12.18
M0060	-651.374946	-408737.7786	12.45
M0071	-651.37272	-408736.3818	13.84
M0072	-651.37272	-408736.3818	13.84
M0100	-651.371324	-408735.5058	14.72
M0098	-651.370137	-408734.761	15.46
M0081	-651.367829	-408733.3127	16.91

20_{RC}	Calculated energy (au)	Calculated energy (kcal/mol)	Relative energy (kcal/mol)
M0001	-651.391637	-408748.2522	0.00
M0012	-651.389959	-408747.1993	1.05
M0002	-651.389141	-408746.686	1.57
M0003	-651.38913	-408746.6791	1.57
M0006	-651.387178	-408745.4542	2.80
M0024	-651.385707	-408744.5311	3.72
M0017	-651.385572	-408744.4464	3.81
M0019	-651.385467	-408744.3805	3.87
M0013	-651.384864	-408744.0022	4.25
M0008	-651.384355	-408743.6828	4.57
M0022	-651.384152	-408743.5554	4.70
M0015	-651.38357	-408743.1902	5.06
M0005	-651.383333	-408743.0415	5.21
M0014	-651.382565	-408742.5595	5.69
M0007	-651.38167	-408741.9979	6.25
M0009	-651.380527	-408741.2807	6.97
M0021	-651.379726	-408740.7781	7.47
M0011	-651.379638	-408740.7228	7.53
M0004	-651.379334	-408740.5321	7.72
M0027	-651.379082	-408740.374	7.88
M0018	-651.378735	-408740.1562	8.10

M0020	-651.378623	-408740.0859	8.17
M0010	-651.377376	-408739.3034	8.95
M0016	-651.377375	-408739.3028	8.95
M0023	-651.376197	-408738.5636	9.69
M0025	-651.376047	-408738.4695	9.78
M0026	-651.374998	-408737.8112	10.44
**20_{RE}**	**Calculated energy (au)**	**Calculated energy (kcal/mol)**	**Relative energy (kcal/mol)**
M0003	-651.410997	-408760.4006	0.00
M0001	-651.410196	-408759.898	0.50
M0005	-651.409463	-408759.438	0.96
M0002	-651.408863	-408759.0615	1.34
M0007	-651.408165	-408758.6235	1.78
M0008	-651.407186	-408758.0092	2.39
M0006	-651.406806	-408757.7708	2.63
M0014	-651.406498	-408757.5775	2.82
M0018	-651.405627	-408757.0309	3.37
M0011	-651.405531	-408756.9707	3.43
M0020	-651.405216	-408756.773	3.63
M0004	-651.405208	-408756.768	3.63
M0009	-651.404575	-408756.3708	4.03
M0013	-651.404406	-408756.2648	4.14
M0012	-651.404352	-408756.2309	4.17
M0023	-651.403227	-408755.5249	4.88
M0024	-651.402888	-408755.3122	5.09
M0021	-651.402234	-408754.9018	5.50
M0015	-651.402015	-408754.7644	5.64
M0019	-651.401954	-408754.7261	5.67
M0010	-651.401927	-408754.7092	5.69
M0025	-651.401728	-408754.5843	5.82
M0016	-651.40166	-408754.5417	5.86
M0017	-651.401241	-408754.2787	6.12
M0022	-651.40082	-408754.0146	6.39

Table S46. Energies calculated at DFT/EDF2/6-31G* in vacuum for the conformers found for **20_{RO}/20_{RC}/20_{RE}**.

Isomer	ΔG° (au)	ΔG° (kcal/mol)	ΔG° (kcal/mol)
**20_{RO}**	-651.171645	-408610.21	7.96
**20_{RC}**	-651.164586	-408605.78	12.39
**20_{RE}**	-651.184324	-408618.16	0.00

Table S47. Relative free energies (ΔG°) of the lowest energy geometries of **20_{RO}/20_{RC}/20_{RE}** at DFT/EDF2/6-31G* in vacuum in kcal/mol.

XYZ Coordinates

20_{RO}

H	3.10252000	-1.88764000	1.41298000
C	2.49661000	-1.15943000	0.85788000
H	1.96429000	-0.57797000	1.61965000
C	1.50578000	-1.96002000	0.01177000
H	0.83975000	-2.50306000	0.69336000
H	2.05686000	-2.72610000	-0.54834000
C	0.67333000	-1.15086000	-0.98392000
H	1.31010000	-0.77538000	-1.78610000
H	-0.02894000	-1.83209000	-1.48180000
N	-0.08429000	-0.06551000	-0.35999000
C	0.49558000	1.17399000	-0.10380000
C	3.44248000	-0.26743000	0.05316000
H	3.59094000	-0.65435000	-0.96147000
H	4.42659000	-0.30846000	0.53860000
C	3.03313000	1.20682000	0.01524000
C	1.79375000	1.53008000	-0.81067000
H	2.90699000	1.57627000	1.04068000
H	3.87289000	1.76983000	-0.41206000
H	1.84837000	1.12132000	-1.82148000
H	1.75948000	2.61812000	-0.96267000
O	0.02724000	1.98949000	0.69317000
C	-1.33644000	-0.43680000	0.15578000
O	-1.66921000	-1.62504000	0.26319000
C	-2.30908000	0.67875000	0.59159000
H	-2.05516000	0.96169000	1.61801000
H	-2.22946000	1.55489000	-0.05882000
N	-3.70865000	0.18899000	0.59246000
H	-3.69408000	-0.75534000	0.99052000
C	-4.25220000	0.11230000	-0.75770000
H	-4.28352000	1.10140000	-1.22588000
H	-5.28010000	-0.26092000	-0.71281000
H	-3.67874000	-0.56657000	-1.39653000

20_{RC}

H	3.27994000	-0.53818000	1.52240000
C	2.39730000	-0.22658000	0.94770000
H	1.54410000	-0.45870000	1.59421000
C	2.35336000	-1.08646000	-0.31322000
H	2.35804000	-2.13543000	0.01127000
H	3.27781000	-0.94995000	-0.88760000
C	1.16055000	-0.88126000	-1.24586000
H	1.33665000	-0.05008000	-1.93381000
H	1.06483000	-1.77697000	-1.87243000
N	-0.10669000	-0.70459000	-0.56486000
C	-0.77534000	0.55658000	-0.43915000
C	2.50569000	1.28012000	0.73306000
H	3.42974000	1.50310000	0.18514000
H	2.62924000	1.73961000	1.72293000
C	1.34320000	1.98972000	0.03528000
C	-0.06040000	1.56320000	0.49268000
H	1.45700000	3.06067000	0.25341000
H	1.45013000	1.91793000	-1.05110000
H	-0.65411000	2.48745000	0.50512000
H	-0.03045000	1.19883000	1.52620000
O	-0.89236000	1.10228000	-1.72722000
H	-1.59838000	0.55264000	-2.10476000
N	-2.11518000	0.15400000	-0.03165000
C	-0.71560000	-1.69358000	0.18482000
O	-0.31940000	-2.83102000	0.37535000
C	-1.96406000	-1.07187000	0.76183000
H	-2.81882000	-1.73835000	0.62149000
H	-1.78972000	-0.87400000	1.82508000
C	-2.93703000	1.15139000	0.62487000
H	-3.94991000	0.76189000	0.77712000
H	-3.03775000	2.04534000	-0.00003000
H	-2.54720000	1.45067000	1.60382000

20RE

H	-3.02741000	-1.94488000	0.46452000
C	-2.23138000	-1.24209000	0.18688000
H	-1.99136000	-0.68644000	1.09933000
C	-2.77111000	-0.29144000	-0.89293000
H	-3.86632000	-0.26767000	-0.83384000
H	-2.52709000	-0.67416000	-1.89151000
C	-2.26771000	1.15060000	-0.75523000
H	-2.50154000	1.71147000	-1.66608000
C	1.74176000	-0.79289000	0.08792000
C	-1.03861000	-2.06825000	-0.31897000
H	-0.51289000	-1.55197000	-1.12905000
H	-1.43363000	-2.99054000	-0.76439000
C	-0.03980000	-2.45277000	0.77950000
C	0.85160000	-1.28663000	1.22614000
H	-0.57875000	-2.85224000	1.64674000
H	0.59332000	-3.26536000	0.40218000
H	1.52087000	-1.62603000	2.02559000
H	0.22783000	-0.50368000	1.65375000
O	2.38156000	-1.60401000	-0.58484000
H	-2.78603000	1.63903000	0.07682000
N	-0.83651000	1.22585000	-0.55798000
N	1.78398000	0.56975000	-0.18330000
C	1.23822000	1.60234000	0.70820000
H	1.74933000	2.55240000	0.51754000
H	1.43257000	1.31196000	1.74572000
C	-0.27013000	1.83439000	0.54138000
O	-0.90223000	2.52452000	1.33687000
H	-0.21884000	0.73793000	-1.19764000
C	2.61543000	1.04102000	-1.28074000
H	2.85100000	0.24086000	-1.98720000
H	2.08485000	1.83409000	-1.81602000
H	3.54768000	1.43633000	-0.86668000

References

[1] T. C. Stephens, M. Lodi, A. Steer, Y. Lin, M. Gill, W. P. Unsworth, *Chem. Eur. J.* **2017**, *23*, 13314.

[2] J. A. Marshall, V. H. Audia, *J. Org. Chem.* **1987**, *52*, 1106.

[3] Y. Song, B. Y. Zhu, S. Wang, S. Bauer, R. M. Scarborough, U.S. Patent 7,612,089 B2, **2009**.

[4] H. F. Motiwala, M. Charaschanya, V. W. Day, J. Aube, *J. Org. Chem*, **2016**, *81*, 1593.

[5] K. Khumtaveeporn, H. Alper, *J. Am. Chem. Soc.* **1994**, *116*, 5662.

[6] (a) R. Surmont, G. Verniest, J. W. Thuring, G. Macdonald, F. Deroose, N. D. Kimpe, *J. Org. Chem.* **2010**, *75*, 929; (b) K. Sato, M. Tamura, K. Tamato, M. Omote, A. Ando, I. Kumadaki, *Chem. Pharm. Bull.* **2000**, *48*, 1023.

[7] P. B. Hurley, G. R. Dake, *J. Org. Chem*, **2008**, *73*, 4131.

[8] A. Rene, J. Martinez, F. Cavelier, *Eur. J. Org. Chem*. **2014**, 8142.

[9] C. Kitsiou, J. J. Hindes, P. I'Anson, P. Jackson, T. C. Wilson, E. K. Daly, H. R. Felstead, P. Hearnshaw and W. P. Unsworth, *Angew. Chem. Int. Ed.* **54**, 15794.

[10] Spartan'14 Wavefunction, Inc. Irvine, CA.

[11] (a) A. D. Becke, *J. Chem. Phys*. 1992, **97**, 9173; (b) A. D. Becke, *J. Chem. Phys*. 1993, **98**, 5648; (c) C. Lee, W. Yang, R. G. Parr, *Phys. Rev. B.* 1988, **37**, 785.

[12] T. A. Halgren, *J Comput. Chem*. 1996, **17**, 490.

[13] Gaussian 09, Revision D.01, M. J. Frisch, G. W. Trucks, H. B. Schlegel, G. E. Scuseria, M. A. Robb, J. R. Cheeseman, G. Scalmani, V. Barone, B. Mennucci, G. A. Petersson, H. Nakatsuji, M. Caricato, X. Li, H. P. Hratchian, A. F. Izmaylov, J. Bloino, G. Zheng, J. L. Sonnenberg, M. Hada, M. Ehara, K. Toyota, R. Fukuda, J. Hasegawa, M. Ishida, T. Nakajima, Y. Honda, O. Kitao, H. Nakai, T. Vreven, J. A. Montgomery, Jr., J. E. Peralta, F. Ogliaro, M. Bearpark, J. J. Heyd, E. Brothers, K. N. Kudin, V. N. Staroverov, T. Keith, R. Kobayashi, J. Normand, K. Raghavachari, A. Rendell, J. C. Burant, S. S. Iyengar, J. Tomasi, M. Cossi, N. Rega, J. M. Millam, M. Klene, J. E. Knox, J. B. Cross, V. Bakken, C. Adamo, J. Jaramillo, R. Gomperts, R. E. Stratmann, O. Yazyev, A. J. Austin, R. Cammi, C. Pomelli, J. W. Ochterski, R. L. Martin, K. Morokuma, V. G. Zakrzewski, G. A. Voth, P. Salvador, J. J. Dannenberg, S. Dapprich, A. D. Daniels, O. Farkas, J. B. Foresman, J. V. Ortiz, J. Cioslowski, and D. J. Fox, Gaussian, Inc., Wallingford CT, 2013.

[14] S. Grimme, S. Ehrlich and L. Goerigk, *J. Comp. Chem*., **2011**, *32*, 1456.

[15] For a review on continuum solvation models, see: J. Tomasi, B. Mennucci, and R. Cammi, *Chem. Rev.*, **2005**, *105*, 2999.

[16] C. Y. Lin, M. W. George, P. M. W. Gill, *Aust. J. Chem*. 2004, **57**, 365.
